# Supplementary material for: Insights into the nutritional prevention of macular degeneration based on a comparative topic modeling approach
Source: PeerJ Comput Sci. 2024 Mar 20;10:e1940. doi: 10.7717/peerj-cs.1940 (PMC11042009; doi:10.7717/peerj-cs.1940)
Supplement: Supplemental Information 2 [file peerj-cs-10-1940-s002.zip › positive_result_reports/1-s2.0-S0002916523279095-main.pdf]

Multivitamin-multimineral supplements and eye disease: age-related macular degeneration and cataract[1–4]

Johanna M Seddon

ABSTRACT
The prevalence and effects of age-related macular degeneration (AMD) and cataract are increasing dramatically as the proportion of elderly in our population continues to rise. A multivitamin-multimineral supplement with a combination of vitamin C, vitamin E, β-carotene, and zinc (with cupric oxide) is recommended for AMD but not cataract. Weak support exists for multivitamins or other vitamin supplements from observational studies of cataract. The results of observational studies suggest that a healthy lifestyle with a diet containing foods rich in antioxidants, particularly lutein and zeaxanthin, as well as n−3 fatty acids, appears beneficial for AMD and possibly cataract. The Age-Related Eye Disease Study II will evaluate some of these additional nutrients as dietary supplements in a randomized trial. *Am J Clin Nutr* 2007;85(suppl): 304S–7S.

KEY WORDS AREDS, Age-Related Eye Disease Study, macular degeneration, cataract, antioxidants, eye

INTRODUCTION

The prevalence and effects of age-related macular degeneration (AMD) and cataract are increasing dramatically as the proportion of elderly in our population continues to rise (1–3). In 1984, in the Epidemiology Unit at the Massachusetts Eye and Ear Infirmary (MEEI), we began to explore whether diet or supplements could prevent or slow the progression of AMD and cataract. Our hypothesis involved oxidative mechanisms whereby daily insults such as pollution, smoking, sunlight, and even normal metabolic processes that lead to free radicals and oxidation could damage the retina and could theoretically be blocked by antioxidants such as those that occur in foods or supplements (4, 5). There was also much discussion at that time about the potential effect of vitamins on other chronic diseases such as cancer and heart disease.

THE DIETARY ANCILLARY STUDY

This Dietary Ancillary Study of the Eye Disease Case-Control Study (EDCCS) was designed to evaluate the relation between nutrition and AMD. The main EDCCS was directed by the National Eye Institute, and the Dietary Ancillary Study was directed by the Epidemiology Unit at MEEI, where the dietary questionnaire was designed for use by elderly individuals (6) and the dietary data were analyzed from the 5 centers. In the Dietary Ancillary Study, lutein and zeaxanthin from foods were associated with a decreased risk of AMD, whereas β-carotene from foods was not (7). We also found that specific foods rich in lutein and zeaxanthin also decreased the risk of AMD. Leading the list of foods rich in lutein and zeaxanthin are kale, spinach, and collard greens, which prompted some lay press at the time to tout the benefits of Popeye's message about eating spinach. This finding also made sense biologically because lutein and zeaxanthin are the carotenoid pigments that are present in the center of the retina, called the macula (8). They may act as free radical scavengers and can filter the damaging rays of blue light. However, the results regarding supplement use were not strong in this Dietary Ancillary Study, with a small, nonsignificant association with multivitamins and vitamin C for ≥2 y but no association with vitamins A or E (7).

THE AGE-RELATED EYE DISEASE STUDY

The gold standard for evaluating supplements is a randomized controlled clinical trial, the Age-Related Eye Disease Study (AREDS), which was initiated in 1990 by the National Eye Institute (9, 10). It involved 11 centers in the United States and enrolled ≈5000 patients. Four AMD categories were defined on the basis of fundus photographs of the macula, ranging from no AMD to advanced disease. Cataract was assessed by photography of the lens. The study supplements were vitamin C (500 mg), vitamin E (400 IU), β-carotene (15 mg), and zinc oxide (80 mg) with cupric oxide (2 mg), and the patients were assigned to these 4 treatment groups in a factorial design. The antioxidant-zinc combination group had a 25% reduction in risk of progression to advanced AMD over 5 y and a 19% reduction in loss of ≥3 lines of vision over the same period. Because ≈8 million Americans are at risk of advanced AMD, >300 000 persons could be saved from vision loss over 5 y if they all took these supplements (11). Based on AREDS and the observational studies of foods, many

[1] From the Epidemiology Unit, Massachusetts Eye and Ear Infirmary, Harvard Medical School, Boston, MA.

[2] Supported in part by the Epidemiology Unit Research Fund, Massachusetts Eye and Ear Infirmary; Foundation Fighting Blindness; and the Massachusetts Lions Eye Research Fund.

[3] Presented at the conference "Multivitamin/Mineral Supplements and Chronic Disease Prevention," held at the National Institutes of Health, Bethesda, MD, May 15–17, 2006.

[4] Address reprint requests to J Seddon, Epidemiology Unit, Department of Ophthalmology, Massachusetts Eye and Ear Infirmary, 243 Charles Street, Boston, MA 02114. E-mail: jseddon@earthlink.net.

 © 2007 American Society for Nutrition

TABLE 1
Relations between supplement use and age-related macular degeneration (AMD)[1]

	Subjects and study design	Nutrients	Results
Randomized trials			
Newsome et al, 1988 (12)	151 subjects (early AMD): 95 women, 56 men; duration: 12–24 mo	Zinc sulfate, 100 mg	Delay in visual loss
Stur et al, 1996 (13)	112 subjects with wet AMD in one eye; 21 mo	Zinc sulfate, 200 mg	No effect on fellow eye
AREDS, 2001 (10)	3640 subjects; 6.3 y	Vitamins C, E, and β-carotene; zinc alone; vitamins C, E, and β-carotene plus zinc; placebo	Antioxidants plus zinc, 25% reduction in progression at 5 y
Taylor et al, 2002 (14)	1193 subjects; 4 y	Vitamin E, 500 IU	No effect on early AMD
Richer et al, 2004 (15)	90 subjects randomly assigned; 12 mo	Lutein 10 mg; Lutein plus antioxidants; placebo	Improved visual function with lutein
Prospective cohort studies			
Vandenlandgenberg et al, 1998 (16)	Beaver Dam, 1709 subjects; 5-y incidence of early AMD	Foods and supplements combined	Carotenoids, vitamin E decreased risk of large drusen, zinc decreased risk of macular pigment abnormalities
Christen et al, 1999 (17)	PHS, 279 cases; 12.5 y of follow-up	Supplements, vitamins E and C, multivitamins	No significant effects
Flood et al, 2002 (18)	Australia, 1989 subjects, 192 cases of early AMD	Food and supplements combined	No beneficial effect
Cho et al, 2001 (19)	NHS, HPFS, 195 early AMD, 189 late AMD	Food and supplements	No association with zinc from food or supplements
Cho et al, 2004 (20)	NHS, HPFS, 464 early AMD, 316 wet AMD	Food and supplements	No association with antioxidant nutrients (food and supplements combined) (positive association with fruit, vegetables)
Case-control studies			
Seddon et al, 1994 (7)	Diet Ancillary Study, EDCCS, 356 advanced AMD cases, newly diagnosed, 520 controls	Food and supplements	Vitamins A, C, and E, no association (positive association with lutein and zeaxanthin from foods)
Teikari et al, 1998 (21)	ATBC, smoking males, end of trial exam, 269 cases, early AMD	Vitamin E, β-carotene, both, placebo	No effect

[1] AREDS, Age-Related Eye Disease Study; ATBC, Alpha-Tocopherol, Beta-Carotene Cancer Prevention Trial; EDCCS, Eye Disease Case-Control Study; HPFS, Health Professionals Follow-Up Study; NHS, Nurses' Health Study; PHS, Physicians' Health Study.

clinicians now recommend that eligible patients with intermediate or advanced AMD in one eye take an AREDS-type supplement and consume a diet rich in antioxidants. Nutritional advice based on scientific studies, including foods and supplements, has had a major impact on the management of AMD.

SUPPLEMENT USE AND AMD

Our review of the literature regarding supplement use for both AMD and cataract included randomized trials, cohort studies, and case-control studies. As listed in **Table 1**, there were 5 randomized trials for AMD (10, 12–15), 5 cohort studies (16–20), and 2 case-control studies (7, 21). The largest and most robust study concerning supplements is AREDS, which found a reduction in the rate of progression of AMD with the use of a multivitamin-multimineral combination supplement (9).

SUPPLEMENT USE AND CATARACTS

Regarding the relation between cataract and supplements, a few randomized trials were initially designed to evaluate cataract (2 of these were in China) (10, 22, 23), and 3 other studies evaluated cataract later in the clinical trials designed for other diseases (24–26) (**Table 2**). AREDS found no effect of the multivitamin-multimineral supplement on cataract progression, and all 3 trials that added cataract later in the study also found no effect. The other trials with cataract as the primary outcome found either a beneficial effect only in subgroups or a small decrease in progression. Several cohort studies had mixed results (27–34); some suggest a beneficial effect for vitamin C, multivitamins, and vitamin E, especially for longer use. Of 3 case-control studies (35–37), 2 supported vitamin C use of $\geq$10 y and 1 showed no effect. For cataract, therefore, the results are mixed, and the largest randomized trial done in the United States, AREDS, showed no beneficial effect of supplements containing vitamin C, vitamin E, β-carotene, and zinc with cupric oxide on development or progression of cataract (10).

THERAPEUTIC RECOMMENDATIONS

The therapeutic recommendations at this time include an AREDS-type supplement for persons with certain stages of AMD (intermediate disease or advanced disease in one eye) but not cataract. However, there have been concerns about some of

TABLE 2
Relations between supplement use and cataract[1]

	Subjects and study design	Nutrients	Results
Randomized trials (primary outcomes)			
Sperduto et al, 1993 (22)	China, I, 2141 subjects; II, 3249 subjects	I, multivitamin-multimineral; II, retinol and zinc, riboflavin and niacin, vitamin C and molybdenum, selenium, vitamin E, and β-carotene	I, 36% ↓ nuclear cataracts (age 65–74 y); II, 44% ↓ nuclear cataracts in riboflavin and niacin group (age 65–74 y)
AREDS, 2001 (10)	4629 subjects; 6.3 y follow-up	Vitamins C and E, β-carotene	No effect
Chylack et al, 2002 (23)	297 subjects; 2–4 y follow-up	β-carotene, vitamin C, vitamin E	"Small" progression
Randomized trials (secondary outcomes)			
Teikari et al, 1998 (24)	425 incident cataract extractions, ATBC	Vitamin E, β-carotene, both, placebo	No effect
Christen et al, 2002 (25)	PHS (men), cataract and cataract extractions	β-carotene (50 mg qod)	No effect
Christen et al, 2004 (26)	WHS (women), cataract and cataract surgery	β-carotene, vitamin E, aspirin	No effect of β-carotene
Prospective cohort studies			
Hankinson et al, 1992 (27)	NHS; 8-y follow-up, 493 cataract extractions	Food and supplements	Vitamin C supplements ≥10 y, RR = 0.55
Seddon et al, 1994 (28)	PHS, 370 cataracts, 109 cataract extractions	Food and supplements	Multivitamins ↓ risk, RR = 0.73–0.79, vitamins C and E, no effect
Leske et al, 1998 (29)	744 patients, nuclear changes	Food and supplements	Multivitamins ↓ risk, RR = 0.69; vitamin E ↓ risk, RR = 0.43
Brown et al, 1999 (30)	HPFS (men), 840 cataract extractions; 8-y follow-up	Food and supplements	Retinol and vitamin A, no effect
Chasen-Taber et al, 1999 (31)	NHS (women), 1471 cataract extractions; 12-y follow-up	Food and supplements	Retinol and vitamin A, no effect
Chasen-Taber et al, 1999 (32)	NHS, 1377 cataract extractions; 12-y follow-up	Supplements	No effect, vitamin C ↓ risk (NS) for subgroups
Mares-Perlman et al, 2000 (33)	Beaver Dam, 3039 subjects; 5-y follow-up	Food and supplements	Multivitamins, vitamins C and E >10 y, 60% ↓ risk
Jacques et al, 2005 (34)	NHS, 408 women (subsample)	Diet and supplements 13–15 y before baseline	Vitamin E 10 y, followed progression (nuclear)
Case-control studies			
Jacques et al, 2001 (35)	NHS, 478 women (subsample), nuclear cataracts	Diet and supplements 13–15 y before baseline	Vitamin C, OR = 0.31 for 5th quintile; vitamin C 10 y, OR = 0.36
Taylor et al, 2002 (36)	NHS, 492 women (subsample), cortical and posterior subcapsular cataracts	Diet and supplements 13–15 y before baseline	Vitamin C, OR = 0.43; vitamin C 10 y, OR = 0.40 (only for < 60 y old, cortical cataracts)
Case-control within clinical trial			
Teikari et al, 1997 (37)	1828 evaluated end of trial (5–8 y)	Vitamin E, β-carotene, both, placebo	No effect

[1] AREDS, Age-Related Eye Disease Study; ATBC, Alpha-Tocopherol, Beta-Carotene Cancer Prevention Trial; HPFS, Health Professionals Follow-Up Study; NHS, Nurses' Health Study; OR, odds ratio; PHS, Physicians' Health Study; qod, every other day; RR, relative risk; WHS, Women's Health Study.

the components of the AREDS formula, namely β-carotene supplement use by smokers (38, 39), zinc (40), and vitamin E (41, 42). Observational studies showed that other nutrients derived from foods may also be important. The most consistent evidence relates to lutein and zeaxanthin (7, 8, 43) as well as dietary n−3 fatty acids (44-47). For this reason, the National Eye Institute launched a new randomized clinical trial called AREDS II, which will evaluate lutein and zeaxanthin, n−3 fatty acids, and alternate formulations for the AREDS I-type supplements in a secondary randomization (no β-carotene and a lower dose of zinc).

SUMMARY

In summary, a multivitamin-multimineral supplement with a combination of vitamin C, vitamin E, β-carotene, and zinc (with cupric oxide) is recommended for AMD but not cataract. Observational studies for cataract provide only weak support for multivitamins or other vitamin supplements. The results of observational studies suggest that a healthy lifestyle with a diet containing foods rich in antioxidants, especially lutein and zeaxanthin, and n−3 fatty acids appears beneficial for AMD and possibly cataract. AREDS II will evaluate some of these additional nutrients as dietary supplements in a randomized trial.

The author had no financial interests to declare.

REFERENCES

1. National Advisory Eye Council. Vision research—a national plan: 1999-2003. Vol 1. A report of the National Advisory Eye Council. Bethesda, MD: National Institutes of Health, 1999. (NIH publication 98-4120.)
2. We the people: aging in the United States. Internet: http://www.census.gov/prod/2004pubs/censr-19.pdf (accessed 20 June 2006).

3. Klein R, Klein BEK, Linton KLP. Prevalence of age-related maculopathy. Ophthalmology 1992;99:933–43.
4. Hung S, Seddon J. The relationship between nutritional factors and age-related macular degeneration. In: Bendich A, Deckelbaum RJ, eds. Preventive nutrition, the comprehensive guide for health professional. 2nd ed. Totowa, NJ: Humana Press, 2001:247–69.
5. Seddon JM, Chen CA. Epidemiology of age-related macular degeneration. In: Ryan SJ, ed. Retina. 4th ed. Vol 2. Medical retina. St Louis, MO: CV Mosby, 2006.
6. Ajani U, Willett W, Seddon J, Eye Disorders Case-Control Study Group. Reproducibility of a food frequency questionnaire for use in ocular research. Invest Ophthalmol Vis Sci 1994;35:2725–33.
7. Seddon JM, Ajani UA, Sperduto RD, et al. Dietary carotenoids, vitamins A, C, and E, and advanced age-related macular degeneration. JAMA 1994;272:1413–20.
8. Snodderly DM, Auran JD, Delori FC. The macular pigment. II. Spatial distribution in primate retinas. Invest Ophthalmol Vis Sci 1984;25:674–85.
9. Age-Related Eye Disease Study Research Group. A randomized, placebo-controlled, clinical trial of high-dose supplementation with vitamins C and E, beta carotene and zinc for age-related macular degeneration and vision loss: AREDS report no. 8. Arch Ophthalmol 2001; 119:1417–36.
10. Age-Related Eye Disease Study Research Group. A randomized, placebo-controlled, clinical trial of high-dose supplementation with vitamins C and E and beta carotene for age-related cataract and vision loss: AREDS report no. 9. Arch Ophthalmol 2001;119:1439–52.
11. Age-Related Eye Disease Study Research Group. Potential public health impact of age-related eye disease study results. AREDS report no. 11. Arch Ophthalmol 2003;121:1621–4.
12. Newsome DA, Swartz M, Leone NC, et al. Oral zinc in macular degeneration. Arch Ophthalmol 1988;106:192–8.
13. Stur M, Tittl M, Reitner A, et al. Oral zinc and the second eye in age-related macular degeneration. Invest Ophthalmol Vis Sci 1996;37: 1225–35.
14. Taylor HR, Tikellis G, Robman LD, et al. Vitamin E supplementation and macular degeneration: randomized controlled trial. BMJ 2002;325: 11–4.
15. Richer S, Stiles W, Statkute L, et al. Double-masked, placebo-controlled, randomized trial of lutein and antioxidant supplementation in the intervention of atrophic age-related macular degeneration: the Veterans LAST study (Lutein Antioxidant Supplementation Trial). Optometry 2004;75:216–30.
16. Vandenlangenberg GM, Mares-Perlman JA, Klein R, et al. Associations between antioxidant and zinc intake and the 5-year incidence of early age-related maculopathy in the Beaver Dam Eye Study. Am J Epidemiol 1998;148:204–14.
17. Christen WG, Ajani UA, Glynn RJ, et al. Prospective cohort study of antioxidant vitamin supplement use and the risk of age-related maculopathy. Am J Epidemiol 1999;149:476-84.
18. Flood V, Smith W, Wang JJ, et al. Dietary antioxidant intake and incidence of early age-related maculopathy. Ophthalmology 2002;109:2272–8.
19. Cho E, Seddon JM, Rosner B, et al. Prospective study of intake of fruits, vegetables, vitamins, and carotenoids and risk of age related maculopathy. Arch Ophthalmol 2004;122:883–92.
20. Cho E, Stampfer MJ, Seddon JM, et al. Prospective study of zinc intake and the risk of age-related macular degeneration. Ann Epidemiol 2001; 11:328–36.
21. Teikari JM, Laatikainen L, Virtamo J, et al. Six year supplementation with alpha-tocopherol and beta-carotene and age-related maculopathy. Acta Ophthalmol Scand 1998;76:224–9.
22. Sperduto RD, Hu TS, Milton RC, et al. The Linxian cataract studies. Two nutrition intervention trials. Arch Ophthalmol 1993;111:1246–53.
23. Chylack LT Jr, Brown NP, Bron A, et al. The Roche European American Cataract Trial (REACT): a randomized clinical trial to investigate the efficacy of an oral antioxidant micronutrient mixture to slow progression of age-related cataract. Ophthalmic Epidemiol 2002;9:49–80.
24. Teikari JM, Rautalahti M, Haukka J, et al. Incidence of cataract operations in Finnish male smokers unaffected by alpha-tocopherol or beta carotene supplements. J Epidemiol Community Health 1998;52:468–72.
25. Christen WG, Manson JE, Glynn RJ, et al. A randomized trial of beta carotene and age-related cataract in US physicians. Arch Ophthalmol 2002;120:372–8.
26. Christen W, Glynn R, Sperduto R, et al. Age-related cataract in a randomized trial of beta-carotene in women. Ophthalmic Epidemiol 2004; 11:401–12.
27. Hankinson SE, Stampfer MJ, Seddon JM, et al. Nutrient intake and cataract extraction in women: a prospective study. BMJ 1992;305:335–9.
28. Seddon JM, Christen WG, Manson JE, et al. The use of vitamin supplements and the risk of cataract among US male physicians. Am J Public Health 1994;84:788–92.
29. Leske MC, Chylack LT Jr, He Q, et al. Antioxidant vitamins and nuclear opacities: the longitudinal study of cataract. Ophthalmology 1998;105: 831–6.
30. Brown L, Rimm EB, Seddon JM, et al. A prospective study of carotenoid intake and risk of cataract extraction in US men. Am J Clin Nutr 1999; 70:517–24.
31. Chasan-Taber L, Willett WC, Seddon JM, et al. A prospective study of carotenoid and vitamin A intakes and risk of cataract extraction in US women. Am J Clin Nutr 1999;70:509–16.
32. Chasan-Taber L, Willett WC, Seddon JM, et al. A prospective study of vitamin supplement intake and cataract extraction among US women. Epidemiology 1999;10:679–84.
33. Mares-Perlman JA, Lyle BJ, Klein R, et al. Vitamin supplement use and incident cataracts in a population-based study. Arch Ophthalmol 2000; 118:1556–63.
34. Jacques PF, Taylor A, Moeller S, et al. Long-term nutrient intake and 5-year change in nuclear lens opacities. Arch Ophthalmol 2005;123: 517–26.
35. Jacques PF, Chylack LT Jr, Hankinson SE, et al. Long-term nutrient intake and early age-related nuclear lens opacities. Arch Ophthalmol 2001;119:1009–19.
36. Taylor A, Jacques PF, Chylack LT Jr. Long-term intake of vitamins and carotenoids and odds of early age-related cortical and posterior subcapsular lens opacities. Am J Clin Nutr 2002;75:540–9.
37. Teikari JM, Virtamo J, Rautalahti M, Palmgren J, Liesto K, Heinonen OP. Long-term supplementation with alpha-tocopherol and beta-carotene and age-related cataract. Acta Ophthalmol Scand 1997;75:634–40.
38. The Alpha-Tocopherol, Beta Carotene Cancer Prevention Study Group. The effect of vitamin E and beta carotene on the incidence of lung cancer and other cancers in male smokers. N Engl J Med 1994;330:1029–35.
39. Omenn GS, Goodman GE, Thornquist MD, et al. Effects of a combination of beta carotene and vitamin A on lung cancer and cardiovascular disease. N Engl J Med 1996;334:1150–5.
40. Lovell MA, Smith JL, Markesbery WR. Elevated zinc transporter-6 in mild cognitive impairment, Alzheimer disease, and pick disease. J Neuropathol Exp Neurol 2006;65:489–98.
41. Miller ER, Pastor-Barriuso R, Dalal D, et al. Meta-analysis: high-dosage vitamin E supplementation may increase all-cause mortality. Ann Intern Med 2005;142:37–46.
42. Chew EY, Clemmons T. Vitamin E and the age-related eye disease study supplementation for age-related macular degeneration. Arch Opthalmol 2005;123:395–6.
43. Mares-Perlman JA, Fisher AI, Klein R, et al. Lutein and zeaxanthin in the diet and serum and their relation to age-related maculopathy in the third National Health and Nutrition Examination Survey. Am J Epidemiol 2001;153:424–32.
44. Seddon JM, Rosner B, Sperduto RD, et al. Dietary fat and risk for advanced age-related macular degeneration. Arch Ophthalmol 2001;119:1191–9.
45. Seddon JM, Cote J, Rosner B. Progression of age-related macular degeneration. Association with dietary fat, transunsaturated fat, nuts, and fish intake. Arch Ophthalmol 2003;121:1728–37.
46. Cho E, Hung S, Willett W, et al. Prospective study of dietary fat and the risk of age-related macular degeneration. Am J Clin Nutr 2001;73:209–18.
47. Seddon J, George S, Rosner B. Cigarette smoking, fish consumption, omega-3 fatty acid intake, and associations with age-related macular degeneration. The US twin study of age-related macular degeneration. Arch Ophthalmol 2006;124:995–1001.
