# Supplementary material for: Insights into the nutritional prevention of macular degeneration based on a comparative topic modeling approach
Source: PeerJ Comput Sci. 2024 Mar 20;10:e1940. doi: 10.7717/peerj-cs.1940 (PMC11042009; doi:10.7717/peerj-cs.1940)
Supplement: Supplemental Information 2 [file peerj-cs-10-1940-s002.zip › positive_result_reports/new1.pdf]

Clinical Trials

The Impact of Supplemental Antioxidants on Visual Function in Nonadvanced Age-Related Macular Degeneration: A Head-to-Head Randomized Clinical Trial

Kwadwo Owusu Akuffo,[1] Stephen Beatty,[1] Tunde Peto,[2] Jim Stack,[1] Jim Stringham,[3] David Kelly,[1] Irene Leung,[4] Laura Corcoran,[1] and John M. Nolan[1]

[1]Macular Pigment Research Group, Nutrition Research Centre Ireland, School of Health Sciences, Waterford Institute of Technology, Waterford, Ireland
[2]Centre for Public Health, Queen's University Belfast, Belfast, United Kingdom
[3]Nutritional Neuroscience Laboratory, Department of Physiology and Pharmacology, University of Georgia, Athens, Georgia, United States
[4]NIHR Biomedical Research Centre at Moorfields Eye Hospital NHS Foundation Trust and UCL Institute of Ophthalmology, London, United Kingdom

Correspondence: John M. Nolan, Macular Pigment Research Group, Nutrition Research Centre Ireland, Waterford Institute of Technology, West Campus, Carriganore, Waterford, X91 K236, Ireland; jmnolan@wit.ie.

KOA and SB are joint first authors.

Submitted: November 28, 2016
Accepted: September 10, 2017

Citation: Akuffo KO, Beatty S, Peto T, et al. The impact of supplemental antioxidants on visual function in nonadvanced age-related macular degeneration: a head-to-head randomized clinical trial. *Invest Ophthalmol Vis Sci*. 2017;58:5347–5360. DOI: 10.1167/iovs.16-21192

Purpose. The purpose of this study was to evaluate the impact of supplemental macular carotenoids (including versus not including *meso*-zeaxanthin) in combination with coantioxidants on visual function in patients with nonadvanced age-related macular degeneration.

Methods. In this study, 121 participants were randomly assigned to group 1 (Age-Related Eye Disease Study 2 formulation with a low dose [25 mg] of zinc and an addition of 10 mg *meso*-zeaxanthin; $n = 60$) or group 2 (Age-Related Eye Disease Study 2 formulation with a low dose [25 mg] of zinc; $n = 61$). Visual function was assessed using best-corrected visual acuity, contrast sensitivity (CS), glare disability, retinal straylight, photostress recovery time, reading performance, and the National Eye Institute Visual Function Questionnaire-25. Macular pigment was measured using customized heterochromatic flicker photometry.

Results. There was a statistically significant improvement in the primary outcome measure (letter CS at 6 cycles per degree [6 cpd]) over time ($P = 0.013$), and this observed improvement was statistically comparable between interventions ($P = 0.881$). Statistically significant improvements in several secondary outcome visual function measures (letter CS at 1.2 and 2.4 cpd; mesopic and photopic CS at all spatial frequencies; mesopic glare disability at 1.5, 3, and 6 cpd; photopic glare disability at 1.5, 3, 6, and 12 cpd; photostress recovery time; retinal straylight; mean and maximum reading speed) were also observed over time ($P < 0.05$, for all), and were statistically comparable between interventions ($P > 0.05$, for all). Statistically significant increases in macular pigment at all eccentricities were observed over time ($P < 0.0005$, for all), and the degree of augmentation was statistically comparable between interventions ($P > 0.05$).

Conclusions. Antioxidant supplementation in patients with nonadvanced age-related macular degeneration results in significant increases in macular pigment and improvements in CS and other measures of visual function. (Clinical trial, http://www.isrctn.com/ISRCTN13894787).

Keywords: randomized clinical trial, lutein, zeaxanthin, *meso*-zeaxanthin, macular pigment, age-related macular degeneration, visual function, visual acuity, contrast sensitivity, macular pigment, NEI VFQ-25, photostress recovery time, reading performance, glare disability, retinal straylight

AMD is a multifactorial disease characterized by a spectrum of degenerative changes at the macula, ultimately leading to central vision impairment in many cases. Given the growing and aging world population, the number of people suffering from AMD continues to rise. Wong et al.[1] estimated the prevalence of any AMD (globally) to be 8.7% in those aged 45 to 85 years and predicted that the number of people afflicted with AMD worldwide will be 288 million by 2040. In the Republic of Ireland, the current prevalence of (any) AMD among persons aged 50 years and older is estimated to be 7.2%.[2] Beyond the personal suffering of those afflicted with advanced AMD, which includes loss of central vision and associated adverse clinical events such as increased risk of falls, depression, loneliness, suicide, and so on,[3] the growing prevalence of AMD represents a huge socioeconomic burden to society and to health care providers.[4] To address this challenge, preventive, retarding, and vision-optimizing strategies for nonadvanced AMD need to be explored, and prior work in diseased and nondiseased eyes indicates that the enhancement of ocular nutrition is worth pursuing in this endeavor.[5]

Copyright 2017 The Authors
iovs.arvojournals.org | ISSN: 1552-5783

This work is licensed under a Creative Commons Attribution-NonCommercial-NoDerivatives 4.0 International License.

Downloaded from iovs.arvojournals.org on 08/08/2023

Investigative Ophthalmology & Visual Science

Meso-zeaxanthin (MZ), zeaxanthin (Z), and lutein (L) represent the three constituent carotenoids that make up macular pigment (MP), a yellow pigment found in the macula. Their anatomic (central and prereceptorial location), biochemical (antioxidant and anti-inflammatory), and optical (short-wavelength [blue] light-filtering) properties make these compounds ideal candidates to enhance vision and protect against AMD and its progression.[5] The Age Related Eye Disease Study (AREDS) 2, published in May 2013, examined the role of supplementation with two of MP's constituent macular carotenoids (L and Z, in combination with coantioxidants) in patients with intermediate AMD.[6] The primary outcome measure (POM; progression to advanced AMD) in AREDS2 failed to reveal a beneficial effect of supplemental L and Z.[7] However, secondary analysis, where data were dichotomized to those supplemented with L and Z versus those not supplemented with these macular carotenoids, did demonstrate a beneficial effect in terms of progression to the advanced form of the disease, especially in those with a low dietary intake of these carotenoids.[7] It is important to note that AREDS2 was designed and powered to investigate the impact of supplementation with macular carotenoids plus coantioxidants on AMD morphology and on visual acuity, whereas the current trial (Central Retinal Enrichment Supplementation Trial 2 [CREST] AMD - CREST Report 2) was designed and powered to investigate change in psychophysical (visual) function, in patients with nonadvanced AMD, following supplementation with the macular carotenoids plus coantioxidants.

In terms of assessing visual function in patients with retinal disease (including AMD), a number of studies have examined the impact of supplementation with macular carotenoids.[8] Indeed, recent studies have reported favorable outcomes on visual function (e.g., contrast sensitivity [CS] and glare disability [GD]) in patients with AMD and other retinal diseases, following supplementation with the macular carotenoids using a formulation of MZ:L:Z in a ratio (mg/d) of 10:10:2.[9,10] However, given the exploratory nature of those studies, a double-blind randomized controlled trial (RCT) with appropriate methodology was warranted. Originally, the CREST AMD trial planned a placebo-controlled design, but following publication of AREDS2, the CREST Data Safety and Monitoring Committee (DSMC) recommended that the design be amended to reflect the new standard of care and that, accordingly, the placebo group should be replaced with an AREDS2 formula containing a lower dose of zinc (25 mg). In the amended protocol, we chose a lower zinc dose (25 mg) because the AREDS2 study found no efficacy-lowering effect of reducing zinc from 80 mg to 25 mg on either visual acuity or AMD progression.[7]

In summary, CREST AMD was designed and conducted to investigate the impact of macular carotenoid supplementation with coantioxidants on visual function in patients with nonadvanced AMD during a 2-year period (ISRCTN13894787).[11] We also investigated whether the addition of 10 mg of MZ to a formulation containing standard AREDS2 doses of L and Z and in combination with coantioxidants offered advantages/disadvantages in terms of a wide array of measures of visual function and MP response.

Methods

Trial Design

Details of the CREST design and methodology have been reported elsewhere and are briefly summarized here.[11] Ethical approval was granted by the Research Ethics Committee of the Waterford Institute of Technology (reference number 12/CLS/02), Waterford, Ireland, and the Ethics Committee of the European Research Council (reference number 281096). As explained previously, following the AREDS2 report, the CREST protocol was amended from a placebo-controlled design to a double-blind, head-to-head, RCT (ISRCTN13894787) in which participants were randomly assigned to two parallel groups, each receiving active supplements as follows: group 1, 10 mg/d MZ, 10 mg/d L, and 2 mg/d Z plus 500 mg/d vitamin C, 400 international units (IU)/d of vitamin E, 25 mg/d zinc, and 2 mg/d copper (Macushield Gold [Alliance Pharma PLC & Alliance Pharmaceuticals Ltd, Chippenham Wiltshire, England, UK]; Macuhealth Plus [MacuHealth Limited Partnership, Birmingham, MI, USA]); and group 2, 10 mg/d L, 2 mg/d Z plus 500 mg/d vitamin C, 400 IU/d of vitamin E, 25 mg/d zinc, and 2 mg/d copper (AREDS2 formula with a lower dose of zinc [25 mg] custom prepared for CREST AMD and not commercially available). The group 2 intervention, therefore, represents the standard of care (AREDS2 formula with a lower dose of zinc [25 mg]), whereas group 1 also represents the same standard of care, but with the addition of 10 mg of MZ. All protocol changes were approved by the DSMC and the Research Ethics Committee of the Waterford Institute of Technology, Waterford, Ireland, and the Ethics Committee of the European Research Council (Saint-Josse-ten-Noode, Brussels, Belgium). In addition, protocol changes were published on the International Standard RCT registration website (www.isrctn.com/ISRCTN13894787) and in the published methodology[11] for this project. Participants in each group were instructed to take the study intervention daily with a meal for 2 years. The trial was conducted at the Macular Pigment Research Group, Nutrition Research Centre Ireland (Waterford, Ireland) from November 2013 (first visit of first participant) to May 2016 (last visit of last participant).

Randomization and Intervention

Participants were randomly assigned to intervention groups using block randomization (block size: 4 and randomization ratio 1:1). The randomization sequence was generated by the study statistician (J.S.), and a pharmacist (C.K.) performed random allocation to intervention groups based on this randomization sequence at Whitfield Clinic, Waterford, Ireland. The study investigator (K.O.A.) received, from the pharmacist, a box of supplements for each study participant, labeled only with the participant identification number. Only at study completion, after a masked database review and following direction from the CREST DSMC, was the randomization sequence revealed to the study investigator and other data analysts.

Participants

Inclusion criteria for the trial were as follows: nonadvanced AMD (1 to 8 on the AREDS 11-step severity scale[12] in at least one eye [the study eye], confirmed by the Moorfields Eye Hospital Reading Centre, London, UK, an accredited retinal grading center); best-corrected visual acuity (BCVA) of 6/12 (20/40) or better in the study eye; no more than five diopters spherical equivalent refraction in the study eye; no previous consumption of supplements containing the macular carotenoids (L and/or Z and/or MZ); no retinal pathology other than AMD; and no diabetes mellitus (by self-report). The study eye could be either the right or left eye. If both eyes exhibited nonadvanced AMD, the eye with the best BCVA was chosen as the study eye. However, if each eye had the same BCVA and nonadvanced AMD, the right eye was selected. Each participant provided written informed consent of their willingness to participate in the trial, and the examination procedures

Downloaded from iovs.arvojournals.org on 08/08/2023

Investigative Ophthalmology & Visual Science

adhered to the tenets of the Declaration of Helsinki. Clinical assessment was conducted at baseline and at six monthly intervals during a 2-year period by the study investigator (K.O.A.) who was trained in all aspects of the CREST protocol. Retinal photographs were graded in a masked fashion at the Moorfields Eye Hospital Reading Centre, adhering to the AREDS 11-step severity scale.[12]

Outcomes

The POM was change in CS at 6 cycles per degree (cpd) following 24 months of supplementation (letter CS at 6 cpd). The Test Chart 2000PRO (Thomson Software Solutions, Hatfield, UK) was used to assess the POM. Letter CS (instead of grating CS) at 6 cpd was chosen as our primary outcome measure because this measure is close to the peak contrast sensitivity function, and any improvements in CS is best assessed at this spatial frequency. Furthermore, pilot data were only available on letter CS (but not grating CS), and this informed our choice in the current study. Secondary outcome measures included change in CS at the other spatial frequencies, BCVA, GD, photostress recovery time (PRT), MP, retinal straylight, reading acuity, reading speed, subjective visual function (National Eye Institute Visual Function Questionnaire–25 [NEI VFQ-25]), and AMD morphology. For measuring BCVA and letter CS, a Hewlett-Packard monitor LV916AA2211 (Hewlett-Packard, Palo Alto, CA, USA; resolution 1920 × 1080, luminance 250 cd/m^2, dynamic contrast ratio 3,000,000: 1) was used. Prior to use for vision testing, the device was calibrated in accordance with the instructions manual from Thomson Software Solutions. Furthermore, all vision testing was conducted in the same room during the course of the study.

Compliance and Adverse Event Reporting

Compliance was assessed by contacting participants via telephone, by capsule counting, and by serum carotenoid analysis at the end of the study. Participants were also phoned regularly to ascertain whether they had experienced any unusual signs/symptoms during the course of the study. Potential or perceived adverse events were documented and reported to the DSMC.

Statistical Analysis

A previous report described the sample size/power calculation for this study.[11] Based on an effect size of 0.15 logCS units (one line on a letter CS chart) for the POM, and a two-tailed test at the 5% level of significance, we estimated that 56 participants per intervention group were needed to achieve a power of 80% for the comparison of the two intervention groups. One eye (the study eye) of each participant comprised the unit of analysis. Statistical analysis was performed using IBM SPSS Statistics for Windows, version 22.0 (Armonk, NY, USA). All analyses were conducted as per protocol. However, intention-to-treat (ITT) analysis was also performed, and discrepancies between ITT analyses and per protocol are reported herein. No interim analyses were conducted during the course of the study.

Baseline differences between intervention groups were assessed using independent samples *t*-tests for interval variables and contingency table analyses using the chi-squared tests for categorical variables.

Most of the outcome variables in this study were changes (over time) in interval variables (e.g., CS, MP). To compare the effects of the two intervention groups (on each interval outcome measure, over time), we used repeated measures analysis of variance, with time as a within-participants factor and intervention group as a between-participants factor. In the ITT analysis, the last observation carried forward was used when participant data were missing.

Tests of significance, for all comparisons of intervention groups on interval outcome measures, were two-tailed, and the 5% level of significance was used throughout. We did not correct for multiple tests, as we were anxious to avoid type II errors.

Results

Figure 1 shows the Consolidated Standards of Reporting Trials diagram,[13] summarizing the CREST study design, participant enrolment, randomization, follow-up, and the number of participants included in study analyses. In this study, 121 participants were enrolled at baseline with 98 participants completing final assessment at 24 months. Baseline characteristics (see Table 1) were statistically comparable between interventions, except for letter CS (1.2 and 2.4 cpd) and photopic CS at 3 cpd. Losses to follow-up after 2 years of antioxidant supplementation were statistically comparable between interventions ($P = 0.680$, Pearson chi-square).

Primary Outcome Measure

The repeated measures analysis of change in letter CS at 6 cpd (POM) is presented in Table 2 (as per protocol). There was a statistically significant improvement in the POM during the study period ($P = 0.013$ for time effect), but there was no statistically significant difference between the intervention groups ($P = 0.881$ for the time × group interaction effect). Thus, there is no evidence that the two intervention groups are different with respect to improvement in this measure. Figure 2 graphically illustrates these findings.

Secondary Outcome Measures

Other Visual Function Outcomes From Baseline to 24 Months. Results from the repeated measures analysis, for other visual function variables, are also shown in Table 2. There was a statistically significant improvement ($P < 0.05$, for time effect) in most measures of visual function (75%; 24 of 32 of vision-related outcome measures) during the study period, including CS, PRT, retinal straylight, and GD, and again these improvements were statistically comparable between intervention groups ($P > 0.05$). There was one exception; mesopic GD at 3 cpd ($P = 0.040$ for the time × group interaction effect), which improved to a borderline significantly greater extent in group 2. However, in the subsequent ITT analysis, the disparity between interventions in terms of mesopic GD at 3 cpd was no longer significant ($P = 0.132$ for the time × group interaction effect). Figures 2, 3, and 4 graphically illustrate these findings.

Clinically Significant Contrast Sensitivity Findings

The numbers and proportions of patients exhibiting clinically meaningful changes (one line or more on a letter CS chart) are presented in Table 3, where it is evident (especially for CS at 1.2 and 2.4 cpd, but also for the POM) that the percentage of participants showing a clinically meaningful improvement in CS over time greatly exceeds the percentage showing a clinically significant deterioration, and that this observation is true for each intervention group.

Macular Pigment From Baseline to 24 Months. There was a statistically significant increase in MP for all eccentric-

Downloaded from iovs.arvojournals.org on 08/08/2023

Investigative Ophthalmology & Visual Science

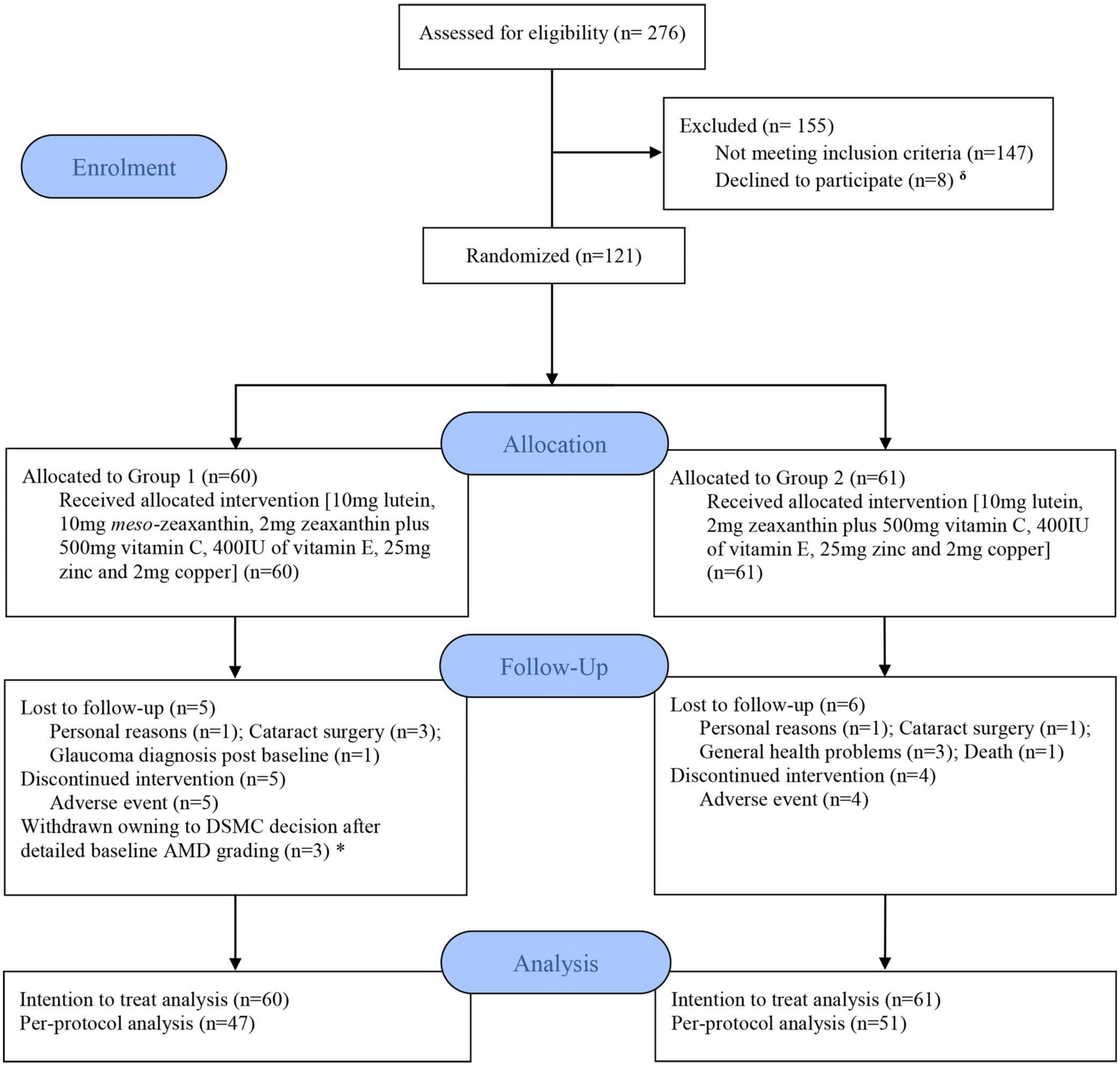

Figure 1. CREST AMD consolidated standards of reporting trials flow diagram. δ, Participants declined to participate either due to personal reasons, transportation difficulties, or cataract surgery; *, Participants were initially enrolled based on nondetail grading of retinal photographs obtained at screening visit, confirming eligibility by the Moorfields Eye Hospital Reading Centre. However, detailed grading of baseline retinal photographs showed some participants had AMD grades > 8 on the AREDS 11-step severity scale and therefore these participants were excluded based on a decision by the DSMC.

ities during the course of the study ($P < 0.0005$, for all time effects), but this increase was statistically comparable between intervention groups ($P > 0.05$ for time × group interaction effect at all retinal eccentricities; Table 4). Figure 5 graphically illustrates these findings.

Serum Carotenoids From Baseline to 24 Months. There was a statistically significant increase in serum concentrations of L, Z, and MZ during the course of the study ($P < 0.0005$, for all time effects; Table 4). The repeated measures analysis of change in serum L concentrations over time did not show significant differences between intervention groups ($P = 0.111$ for the time × group interaction effect). Observed increases in serum Z concentrations were significantly greater in group 2 when compared with group 1 ($P = 0.005$ for the time × group interaction effect). Significant increases in serum MZ concentrations were observed in group 1, but not in group 2 ($P < 0.0005$ for the time × group interaction effect). In terms of observed increases in total (composite) serum macular carotenoid concentrations (i.e., L, Z, and MZ combined), this measure increased significantly over time, and no significant difference between intervention groups ($P = 0.241$ for the time × group interaction effect) was observed. Figure 6 graphically illustrates these findings.

Grade of AMD From Baseline to 24 Months. Table 5 shows, within each intervention group, the transition between these grades from baseline to final study visit at 24 months.

Downloaded from iovs.arvojournals.org on 08/08/2023

Table 1. Baseline Characteristics by Intervention Group in the CREST AMD Study (Per Protocol)

Variables	Group 1, n = 57*	Group 2, n = 61†	Sig.
Demographic, lifestyle, and health			
Age, y	65.09 ± 8.59	64.34 ± 9.50	0.657
Body mass index, kg/m^2	28.27 ± 4.30	27.78 ± 4.57	0.551
Blood pressure, mm Hg			
Systolic	142.07 ± 20.98	138.00 ± 24.35	0.334
Diastolic	82.65 ± 11.21	79.12 ± 9.81	0.070
Sex			
Male	18 (45.0)	22 (55.0)	0.607
Female	39 (50.0)	39 (50.0)	
Education			
Primary	7 (43.8)	9 (56.3)	0.766
Secondary	29 (51.8)	27 (48.2)	
Tertiary	21 (45.7)	25 (54.3)	
Smoking			
Never	29 (50.0)	29 (50.0)	0.933
Past	23 (46.9)	26 (53.1)	
Current	5 (45.5)	6 (54.5)	
AMD family history			
Yes	16 (53.3)	14 (46.7)	0.406
No	31 (44.3)	39 (55.7)	
Cardiovascular disease			
Yes	5 (50.0)	5 (50.0)	0.804
No	50 (47.6)	55 (52.4)	
Hypertension			
Yes	17 (48.6)	18 (51.4)	0.970
No	40 (48.2)	43 (51.8)	
AMD grades			
1-3	13 (43.3)	17 (56.7)	0.528
4-8	44 (50.0)	44 (50.0)	
Diet score	26.90 ± 12.00	26.26 ± 12.03	0.776
Serum carotenoids*			
Serum L, μmol/l	0.35 ± 0.20	0.34 ± 0.22	0.710
Serum Z, μmol/l	0.07 ± 0.05	0.07 ± 0.05	0.639
Serum MZ, μmol/l	0.00 ± 0.01	0.01 ± 0.02	0.205
Macular pigment			
Densitometer*			
0.25°	0.79 ± 0.24	0.72 ± 0.26	0.179
0.5°	0.65 ± 0.22	0.60 ± 0.21	0.204
1.0°	0.45 ± 0.16	0.45 ± 0.17	0.927
1.75°	0.32 ± 0.12	0.31 ± 0.15	0.933
Vision			
Best corrected visual acuity, VAR			
Study eye	100.04 ± 5.83	100.08 ± 5.62	0.965
Fellow eye	94.63 ± 10.95	95.92 ± 12.20	0.549
Letter contrast sensitivity, LogCS			
1.2 cpd	1.77 ± 0.17	1.85 ± 0.16	0.007
2.4 cpd	1.76 ± 0.21	1.83 ± 0.18	0.045
6 cpd, POM	1.49 ± 0.25	1.56 ± 0.21	0.108
9.6 cpd	1.23 ± 0.30	1.32 ± 0.25	0.082
15.15 cpd*	0.86 ± 0.35	0.94 ± 0.29	0.160
Mesopic contrast sensitivity, LogCS			
1.5 cpd	1.53 ± 0.22	1.61 ± 0.21	0.065
3 cpd	1.62 ± 0.23	1.68 ± 0.18	0.106
6 cpd	1.21 ± 0.35	1.33 ± 0.35	0.065
12 cpd	0.78 ± 0.27	0.85 ± 0.28	0.132
18 cpd	0.33 ± 0.12	0.32 ± 0.11	0.749

Downloaded from iovs.arvojournals.org on 08/08/2023

Investigative Ophthalmology & Visual Science

Table 1. Continued

Variables	Group 1, *n* = 57*	Group 2, *n* = 61†	Sig.
Photopic contrast sensitivity, LogCS			
1.5 cpd	1.46 ± 0.19	1.52 ± 0.16	0.061
3 cpd	1.72 ± 0.22	1.80 ± 0.19	0.047
6 cpd	1.58 ± 0.31	1.68 ± 0.31	0.079
12 cpd	1.19 ± 0.38	1.27 ± 0.35	0.279
18 cpd	0.51 ± 0.34	0.62 ± 0.34	0.081
Mesopic glare disability, LogCS			
1.5 cpd	0.91 ± 0.32	0.99 ± 0.29	0.193
3 cpd	1.11 ± 0.37	1.19 ± 0.32	0.241
6 cpd	0.93 ± 0.25	0.93 ± 0.23	0.977
12 cpd	0.66 ± 0.15	0.63 ± 0.11	0.355
18 cpd	0.30 ± 0.00	0.31 ± 0.04	0.336
Photopic glare disability, LogCS			
1.5 cpd	1.40 ± 0.21	1.46 ± 0.17	0.082
3 cpd	1.67 ± 0.22	1.73 ± 0.18	0.130
6 cpd	1.51 ± 0.32	1.58 ± 0.31	0.210
12 cpd	1.11 ± 0.36	1.19 ± 0.36	0.206
18 cpd	0.52 ± 0.35	0.56 ± 0.31	0.583
Retinal Straylight	1.30 ± 0.18	1.33 ± 0.25	0.381
Photostress recovery time, s	15.98 ± 8.72	15.97 ± 7.99	0.996
Reading performance			
Reading acuity, LogRAD	0.12 ± 0.13	0.09 ± 0.12	0.165
Mean reading speed, w/min	154.48 ± 26.82	156.45 ± 27.53	0.694
Maximum reading speed, w/min	199.61 ± 31.58	201.56 ± 34.44	0.749
National Eye Institute Questionnaire-25			
Overall vision score	87.80 ± 9.96	90.38 ± 9.22	0.147

Data displayed are mean ± standard deviation for interval data and percentages, *n* (%), for categorical data; the percentages displayed are row percentages. Sig., significance set at $P < 0.05$. Education, highest level of education; Smoking, Never (<100 cigarettes in lifetime), Past (smoked ≥100 cigarettes in lifetime and none in past year), current (smoked ≥100 cigarettes in lifetime and at least one in the last year). *, $n \neq 57$ in group 1 and/or $n \neq 61$ in group 2 as certain tests/measures were not obtained. VAR, visual acuity rating. VAR = 100 − 50 LogMAR, a score of 100 corresponds with 20/20 (6/6); LogCS, logarithm of contrast sensitivity units. Family history of AMD means having a first degree relative, that is, parent or sibling, with AMD AREDS 11-step scale. Diet score, estimated dietary intake of lutein and zeaxanthin using the "L/Z screener" developed by Professor Elizabeth Johnson, Tufts University. Macular pigment measured using the Macular Densitometer (Macular Metrics Corp.). Serum macular carotenoids analyzed by HPLC. Best-corrected visual acuity measured with the Test Chart 2000 Xpert (Thomson Software Solutions). Letter contrast sensitivity measured using the Test Chart 2000 PRO (Thomson Software Solutions). Mesopic and photopic contrast sensitivity measured using the Functional Vision Analyzer (Stereo Optical Co.). Mesopic and photopic glare disability measured using the Functional Vision Analyzer (Stereo Optical Co.). Retinal straylight measured using the Oculus C-Quant (Oculus GmbH, Wetzler, Germany) and recorded in logarithms (judged reliable when estimated standard deviation (ESD) ≤ 0.08 and Q ≥ 1). Photostress recovery time measured by assessing the time of recovery after a 10-second exposure to a 300-watt tungsten spotlight (ARRI 300 Plus lamp, ARRI Lighting Solutions GmbH, Berlin, Germany) with a low-pass glass dichroic filter. Reading performance assessed using the English version of the standardized Radner reading chart at a distance of 40 cm with reading correction. Reading acuity recorded in logarithm of the reading acuity determination (LogRAD). The following formula was used to calculate the LogRAD-score: logRAD + total number of incorrectly read syllables × 0.005. Reading speed (the time taken to read the number of words in a sentence) was measured in words per minute (w/min) with a stop watch for each standardized sentence (14 words × 60 seconds divided by reading time in seconds). National Eye Institute Visual Function Questionnaire–25 overall vision scores range from zero (worst) to 100 (best).

* Group 1, 10 mg/d MZ, 10 mg/d L, and 2 mg/d Z plus 500 mg/d vitamin C, 400 IU/d of vitamin E, 25 mg/d zinc, and 2 mg/d copper.

† Group 2, 10 mg/d L, 2 mg/d Z plus 500 mg/d vitamin C, 400 IU/d of vitamin E, 25 mg/d zinc, and 2 mg/d copper.

Importantly, no participant from Group 1 (the intervention containing MZ) and only one participant from Group 2 progressed to advanced AMD over the study period.

Compliance

The compliance to study intervention (as measured by capsule counting) was not significantly different between intervention groups during the course of the study ($P = 0.342$ for the time × group interaction effect). In addition, serum carotenoid assessment indicated good compliance to study intervention (see Fig. 6).

Adverse Events

The distribution of potential or perceived adverse events reported during the course of the study is shown in Table 6. Some participants reported more than one adverse event. The proportion of participants experiencing any adverse event was statistically similar between interventions: 15 (26%) of 57 from group 1 and 10 (16%) of 61 from group 2 ($P = 0.187$, Pearson chi-squared test). No serious adverse event relating to the study intervention was reported in either intervention group during the course of the study.

Discussion

This RCT was designed to compare the impact of two different macular carotenoid formulations, in combination with coantioxidants, on visual function in patients with nonadvanced AMD. The AMD disease status of participants was graded using the AREDS 11-step severity scale[12] and included only eyes

Downloaded from iovs.arvojournals.org on 08/08/2023

Investigative Ophthalmology & Visual Science—

TABLE 2. Repeated Measures Analysis of Visual Function Outcomes From Baseline to 24 Months in the CREST AMD Study by Intervention Groups

		Group 1*					Group 2†				Time	Time × Group
		Baseline		24 Months			Baseline		24 Months		Effect	Interaction
Variable	N	Mean	SD	Mean	SD	N	Mean	SD	Mean	SD	Sig.	Sig.
Vision												
Best corrected visual acuity, VAR	46	101.22	5.16	100.91	5.80	51	100.78	5.08	101.31	5.20	0.746	0.233
Letter contrast sensitivity, LogCS												
1.2 cpd	46	1.79	0.17	1.89	0.20	51	1.86	0.14	1.91	0.16	<0.0005	0.058
2.4 cpd	46	1.78	0.22	1.86	0.22	51	1.85	0.16	1.91	0.18	<0.0005	0.582
6 cpd, POM	46	1.53	0.24	1.57	0.29	51	1.58	0.18	1.61	0.23	0.013	0.881
9.6 cpd	46	1.29	0.28	1.31	0.30	51	1.36	0.21	1.38	0.26	0.154	0.925
15.15 cpd	46	0.92	0.33	0.95	0.34	51	0.96	0.27	1.01	0.33	0.082	0.747
Mesopic contrast sensitivity, LogCS												
1.5 cpd	46	1.55	0.22	1.62	0.24	51	1.63	0.21	1.70	0.23	0.007	0.982
3 cpd	46	1.63	0.24	1.76	0.27	51	1.69	0.18	1.84	0.27	<0.0005	0.523
6 cpd	46	1.25	0.35	1.48	0.45	51	1.34	0.34	1.49	0.42	<0.0005	0.228
12 cpd	46	0.81	0.29	0.94	0.36	51	0.87	0.28	0.96	0.35	0.002	0.605
18 cpd	46	0.33	0.13	0.39	0.23	51	0.31	0.08	0.41	0.25	<0.0005	0.369
Photopic contrast sensitivity, LogCS												
1.5 cpd	46	1.47	0.19	1.60	0.23	51	1.53	0.16	1.64	0.21	<0.0005	0.862
3 cpd	46	1.75	0.23	1.84	0.23	51	1.82	0.18	1.91	0.21	<0.0005	0.986
6 cpd	46	1.63	0.28	1.74	0.39	51	1.70	0.29	1.81	0.34	<0.0005	0.934
12 cpd	46	1.25	0.37	1.34	0.43	51	1.30	0.33	1.34	0.37	0.015	0.468
18 cpd	46	0.56	0.36	0.71	0.44	51	0.65	0.34	0.69	0.36	0.008	0.174
Mesopic glare disability, LogCS												
1.5 cpd	46	0.98	0.32	1.08	0.44	51	1.01	0.29	1.20	0.45	<0.0005	0.172
3 cpd	46	1.19	0.36	1.22	0.43	51	1.22	0.30	1.38	0.41	0.001	0.040
6 cpd	46	0.97	0.27	1.05	0.35	51	0.94	0.23	1.09	0.35	<0.0005	0.222
12 cpd	46	0.67	0.16	0.68	0.22	51	0.64	0.12	0.69	0.18	0.133	0.412
18 cpd	46	0.30	0.00	0.32	0.10	51	0.31	0.04	0.31	0.08	0.197	0.486
Photopic glare disability, LogCS												
1.5 cpd	46	1.43	0.21	1.55	0.26	51	1.47	0.18	1.55	0.24	<0.0005	0.364
3 cpd	46	1.70	0.22	1.82	0.25	51	1.74	0.18	1.83	0.24	<0.0005	0.542
6 cpd	46	1.56	0.31	1.65	0.40	51	1.61	0.29	1.70	0.34	0.001	0.987
12 cpd	46	1.18	0.34	1.26	0.41	51	1.23	0.33	1.31	0.38	0.011	0.913
18 cpd	46	0.58	0.37	0.60	0.39	51	0.57	0.31	0.62	0.33	0.179	0.646
Retinal Straylight, Logs	41	1.29	0.18	1.25	0.19	43	1.33	0.20	1.26	0.16	0.004	0.359
Photostress recovery time, s	46	16.93	9.19	12.47	6.79	51	16.00	8.51	10.96	6.05	<0.0005	0.757
Reading performance												
Reading acuity, LogRAD	46	0.09	0.12	0.09	0.08	51	0.07	0.10	0.06	0.10	0.637	0.759
Mean reading speed, w/min	46	154.61	27.11	189.89	26.53	51	158.75	27.00	192.82	28.54	<0.0005	0.765
Maximum reading speed, w/min	46	200.44	32.25	244.00	35.02	51	204.74	33.40	245.38	37.90	<0.0005	0.606
National Eye Institute Questionnaire-25												
Overall vision score	46	89.24	7.95	89.27	9.61	50	90.83	9.66	91.93	7.01	0.408	0.434

N, participants with data at all study visits; Sig., significance set at $P < 0.05$. *P* values obtained from repeated measures analysis of variance. Best-corrected visual acuity measured with the Test Chart 2000 Xpert (Thomson Software Solutions). Letter contrast sensitivity measured using the Test Chart 2000 PRO (Thomson Software Solutions). Mesopic and photopic contrast sensitivity measured using the Functional Vision Analyzer (Stereo Optical Co.). Mesopic and photopic glare disability measured using the Functional Vision Analyzer (Stereo Optical Co.). Retinal straylight measured using Oculus C-Quant (Oculus GmbH) and recorded in logarithms (judged reliable when ESD ≤ 0.08 and Q ≥ 1). Photostress recovery time measured by assessing the time of recovery after a 10-second exposure to a 300-watt tungsten spotlight (ARRI 300 Plus lamp) with a low-pass glass dichroic filter. Reading performance assessed using the English version of the standardized Radner reading chart at a distance of 40 cm with reading correction. Reading acuity recorded in logarithm of the reading acuity determination (LogRAD). The following formula was used to calculate the LogRAD score: logRAD + total number of incorrectly read syllables × 0.005. Reading speed (the time taken to read the number of words in a sentence) was measured in words per minute (w/min) with a stop watch for each standardized sentence (14 words × 60 seconds divided by reading time in seconds). National Eye Institute Visual Function Questionnaire–25 overall vision scores range from zero (worst) to 100 (best).

* Group 1, 10 mg/d MZ, 10 mg/d L, and 2 mg/d Z plus 500 mg/d vitamin C, 400 IU/d of vitamin E, 25 mg/d zinc, and 2 mg/d copper.

† Group 2, 10 mg/d L, 2 mg/d Z plus 500 mg/d vitamin C, 400 IU/d of vitamin E, 25 mg/d zinc, and 2 mg/d copper.

Downloaded from iovs.arvojournals.org on 08/08/2023

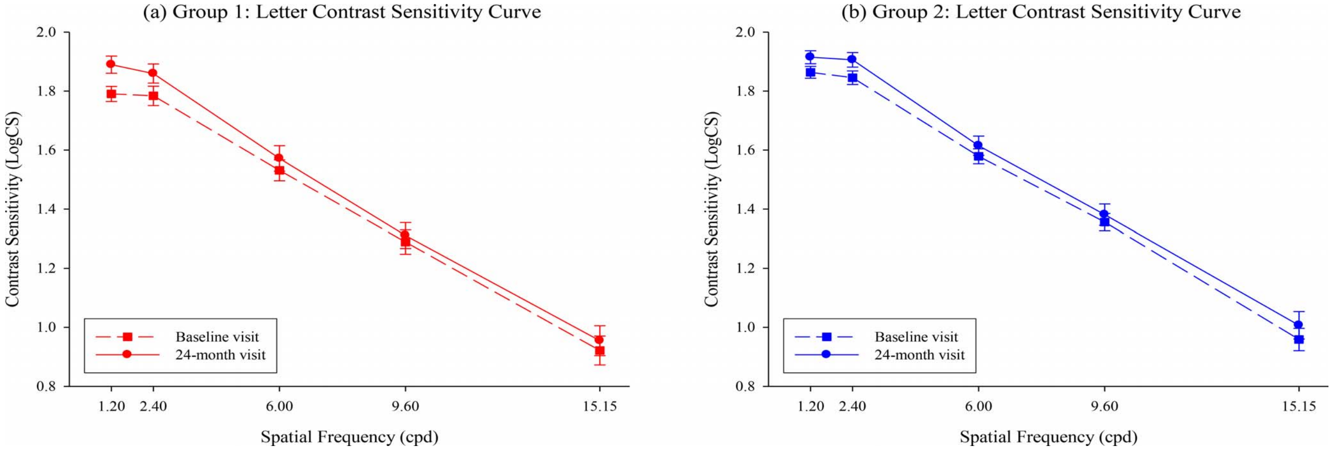

FIGURE 2. Letter contrast sensitivity function using the Test Chart 2000 PRO (Thomson Software Solutions) in the CREST AMD study. Group 1, 10 mg/d MZ, 10 mg/d L, and 2 mg/d Z plus 500 mg/d vitamin C, 400 IU/d of vitamin E, 25 mg/d zinc, and 2 mg/d copper; group 2, 10 mg/d L, 2 mg/d Z plus 500 mg/d vitamin C, 400 IU/d of vitamin E, 25 mg/d zinc, and 2 mg/d copper. *Error bars* represent standard error of mean.

classed as grade 1 to 8 at baseline (referred to as nonadvanced AMD for the purpose of the current study). We did not include eyes with noncentral geographic atrophy (AMD grade 9 on the AREDS 11-step severity scale). Given the biologically plausible rationale that benefits, in terms of vision and in terms of MP augmentation, are more likely to extend to participants with earlier disease (before irreversible damage has occurred, such as in noncentral geographic atrophy [grade 9 AREDS 11-step severity scale]), we purposely recruited eyes at an earlier stage of disease. We report improvements in a range of measures of visual function (i.e., CS, GD, PRT, reading speed) following supplementation with the macular carotenoids in combination

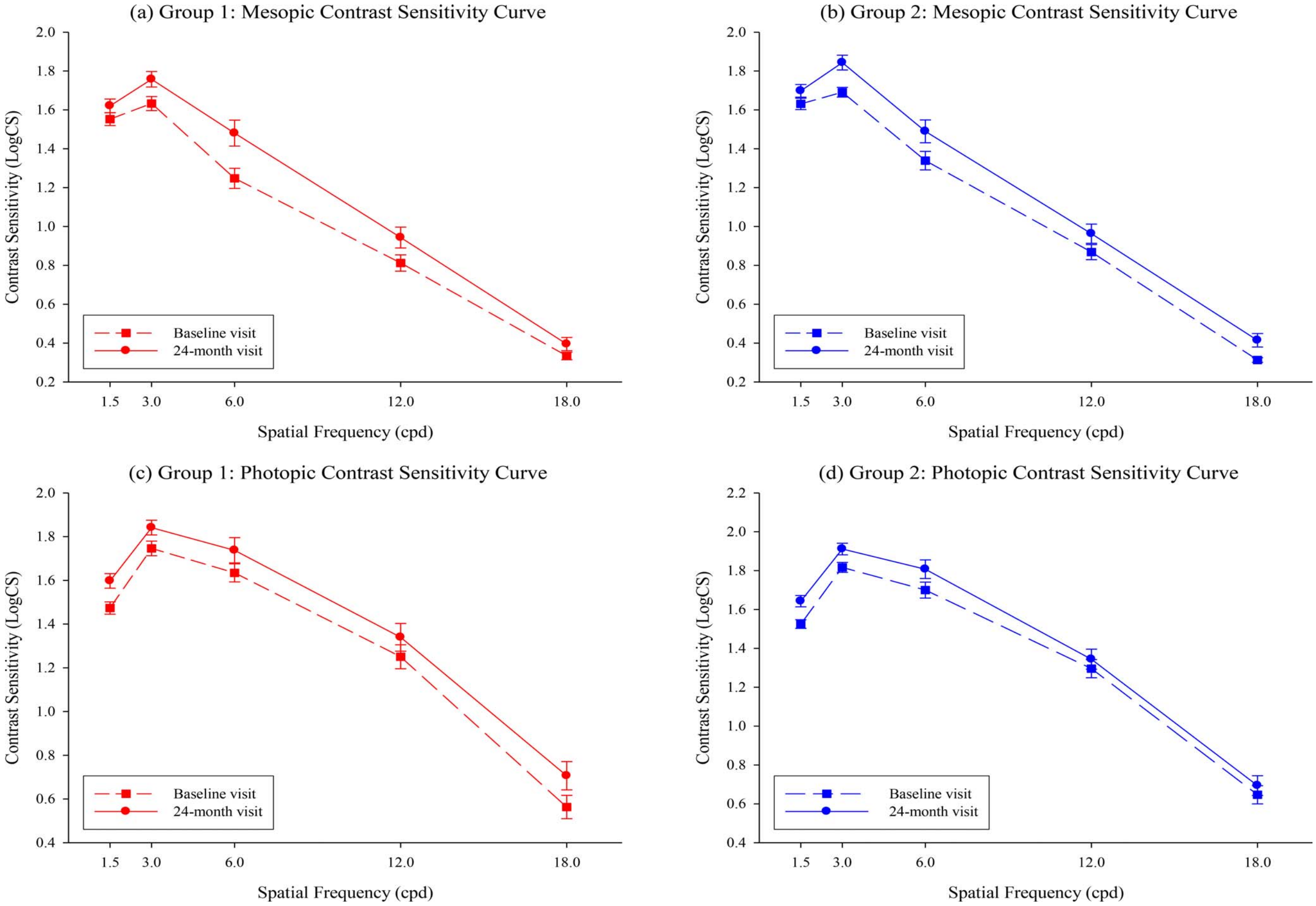

FIGURE 3. Mesopic and photopic contrast sensitivity function using the Functional Vision Analyzer (Stereo Optical Co., Chicago, IL, USA) in the CREST AMD study. Group 1, 10 mg/d MZ, 10 mg/d L, and 2 mg/d Z plus 500 mg/d vitamin C, 400 IU/d of vitamin E, 25 mg/d zinc, and 2 mg/d copper; group 2, 10 mg/d L, 2 mg/d Z plus 500 mg/d vitamin C, 400 IU/d of vitamin E, 25 mg/d zinc, and 2 mg/d copper. *Error bars* represent standard error of mean.

Downloaded from iovs.arvojournals.org on 08/08/2023

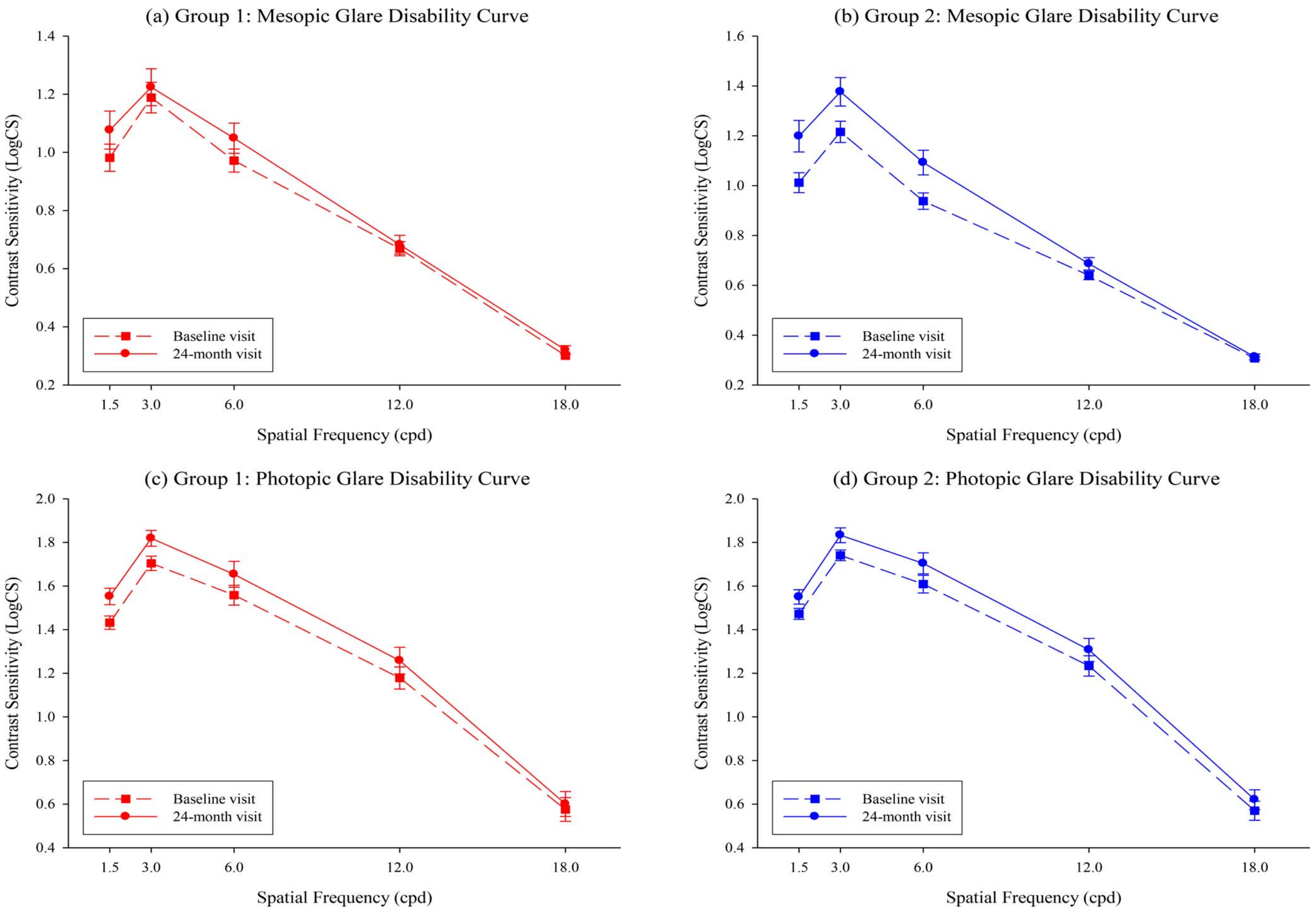

Figure 4. Mesopic and photopic glare disability using the Functional Vision Analyzer (Stereo Optical Co.) in the CREST AMD study. Group 1, 10 mg/d MZ, 10 mg/d L, and 2 mg/d Z plus 500 mg/d vitamin C, 400 IU/d of vitamin E, 25 mg/d zinc, and 2 mg/d copper; group 2, 10 mg/d L, 2 mg/d Z plus 500 mg/d vitamin C, 400 IU/d of vitamin E, 25 mg/d zinc, and 2 mg/d copper. *Error bars* represent standard error of mean.

with coantioxidants, and our results are consistent with previous studies.[9,14,15]

A possible explanation for the role that MP plays in optimizing CS rests on the visibility hypothesis of MP, which posits that this prereceptorial pigment enhances visualization of a target's detail by the absorption of blue haze.[16] Blue haze is a subjective experience and is caused by scattered short-wavelength dominant air light (blue light), which results in a veiling luminance when we view objects at a distance.[16] MP accentuates the luminance of an object relative to its background by attenuating the impact of this scattered (veiling) short-wavelength visible blue light on the

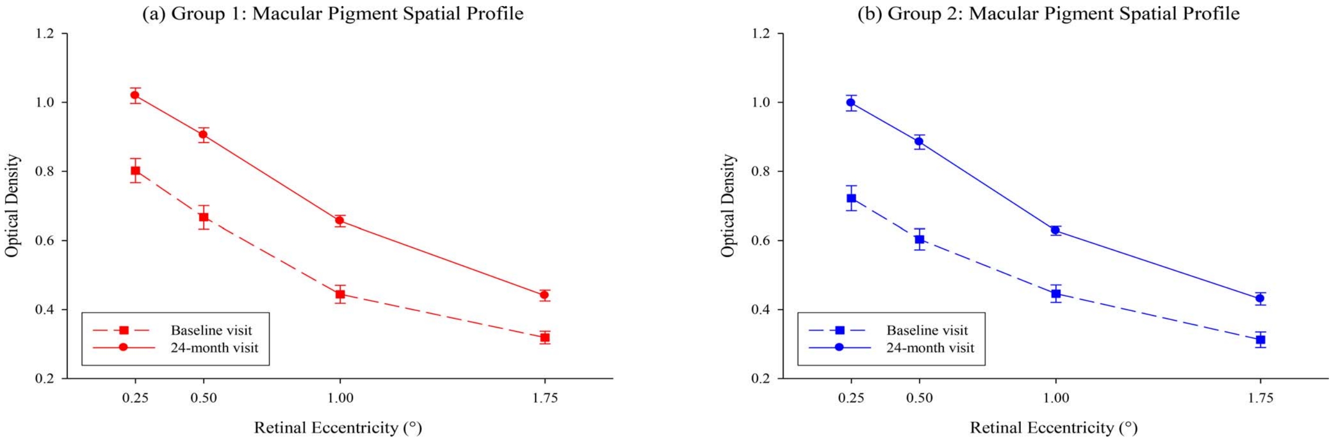

Figure 5. Macular pigment response in the CREST AMD study. Group 1, 10 mg/d MZ, 10 mg/d L, and 2 mg/d Z plus 500 mg/d vitamin C, 400 IU/d of vitamin E, 25 mg/d zinc, and 2 mg/d copper; group 2, 10 mg/d L, 2 mg/d Z plus 500 mg/d vitamin C, 400 IU/d of vitamin E, 25 mg/d zinc, and 2 mg/d copper. Macular pigment measured using a Macular Densitometer (Macular Metrics Corp., Providence, RI, USA). *Error bars* represent standard error of mean.

Investigative Ophthalmology & Visual Science

Downloaded from iovs.arvojournals.org on 08/08/2023

TABLE 3. Change in Contrast Sensitivity of ≥1 Line of CS

Variable	% Showing Clinical Improvement		% Showing Clinical Deterioration	
	Group 1*	Group 2†	Group 1*	Group 2†
Letter CS 1.2 cpd	34.8	19.6	2.2	3.9
Letter CS 2.4 cpd	26.1	21.6	4.3	3.9
Letter CS 6 cpd	26.1	21.6	13	11.8

Clinical significance, which for present purposes we defined as one line or more on a letter CS chart. Letter CS measured using the Test Chart 2000 PRO (Thomson Software Solutions).

* Group 1, 10 mg/d MZ, 10 mg/d L, and 2 mg/d Z plus 500 mg/d vitamin C, 400 IU/d of vitamin E, 25 mg/d zinc, and 2 mg/d copper.

† Group 2, 10 mg/d L, 2 mg/d Z plus 500 mg/d vitamin C, 400 IU/d of vitamin E, 25 mg/d zinc, and 2 mg/d copper.

TABLE 5. Change in AMD Morphology in the CREST AMD Study by Intervention Group

Study Visit	Intervention	Low Risk	High Risk	Advanced AMD	Total
Baseline	Group 1*	13	44	0	57
	Group 2†	17	44	0	61
24 months	Group 1*	11	35	0	46
	Group 2†	11	38	1	50

Low risk, AMD grades 1 to 3 on the AREDS 11-step scale; high risk, AMD grades 4 to 8 on the AREDS 11-step scale; advanced AMD, AMD grades 9 to 11 on the AREDS 11-step scale.

* Group 1, 10 mg/d MZ, 10 mg/d L, and 2 mg/d Z plus 500 mg/d vitamin C, 400 IU/d of vitamin E, 25 mg/d zinc, and 2 mg/d copper.

† Group 2, 10 mg/d L, 2 mg/d Z plus 500 mg/d vitamin C, 400 IU/d of vitamin E, 25 mg/d zinc, and 2 mg/d copper.

just noticeable differences of luminance required for discernibility and, by consequence, extends the visual range.[17] Indeed, the visibility hypothesis has been tested empirically and is supported by two studies that have demonstrated the beneficial effect of MP in this respect under simulated blue haze conditions.[18,19] Beyond this optical effect, the macular carotenoids may also favorably influence lateral inhibitory mechanisms[20] and may thereby have contributed to the observed improvements in CS following supplementation.

Importantly, we believe that the observed improvements in CS in this trial are clinically meaningful. Recently, Maynard et al.[21] demonstrated that, when compared with age-similar healthy eyes, patients with nonadvanced AMD exhibit significantly worse CS (reflecting a deterioration of −0.007 log CS/year), consistent with the findings of a major review by Neelam et al.[22] In terms of visual performance, visual acuity is a measure of the ability to correctly identify targets (of variable size) at 100% contrast, whereas CS is a measure of the ability to detect/identify targets (of variable sizes [spatial frequencies]) at varying contrast (i.e., faintness). Furthermore, CS (but not BCVA) can effectively predict how well patients see targets typical of everyday life, which has important implications for quality of life.[23] Consequently, good visual acuity in the presence of poor CS (e.g., nonadvanced cataract) results in reports of visual complaints,[24] particularly for real-world tasks and targets,[23] but the following question remains: what degree of change in CS will have a clinically meaningful impact for the patient?

For VA, a one-line change (0.1 log MAR) is considered clinically meaningful.[25] For CS, the available data indicate that a 0.1 log change in the percentage threshold contrast required for the detection of a target/pattern is equally (if not more) devastating to visual performance than a deterioration of one line of BCVA.[23,26] In brief, threshold contrast is the contrast required to see the target reliably; the reciprocal of threshold is called sensitivity, which is expressed as a percentage (e.g., see Michelson contrast).[27] For example, for spatial frequencies that are near the peak of the contrast sensitivity function (i.e., 4–6 cycles/degree), younger and middle-aged patients have contrast thresholds of, on average, circa 2.5%. A 0.1 log unit deterioration from this value yields a contrast threshold of 3.2%, which is classed as visual impairment.[28] Moreover, contrast thresholds > 5% are associated with increased risk of driving accidents.[28] Accordingly, a decrease/increase in CS of 0.1 log unit is deemed clinically meaningful; in this study, 19.6% to 34.8% of participants exhibited at least this magnitude of improvement at three spatial frequencies, whereas this

TABLE 4. Repeated Measures Analysis of Macular Pigment and Serum Carotenoid Outcomes From Baseline to 24 Months in the CREST AMD Study by Intervention Group

Variable	Group 1* N	Group 1* Baseline Mean	Group 1* Baseline SD	Group 1* 24 Months Mean	Group 1* 24 Months SD	Group 2† N	Group 2† Baseline Mean	Group 2† Baseline SD	Group 2† 24 Months Mean	Group 2† 24 Months SD	Time Effect Sig.	Time × Group Interaction Sig.
Macular pigment												
0.25°	45	0.80	0.23	1.02	0.15	50	0.72	0.25	1.00	0.16	<0.0005	0.247
0.5°	45	0.67	0.23	0.90	0.14	50	0.60	0.22	0.88	0.15	<0.0005	0.334
1.0°	45	0.44	0.17	0.66	0.11	50	0.45	0.18	0.63	0.09	<0.0005	0.444
1.75°	45	0.32	0.12	0.44	0.10	50	0.31	0.16	0.43	0.13	<0.0005	0.924
Serum carotenoids												
Serum L, μmol/l	41	0.34	0.16	1.40	0.83	47	0.33	0.21	1.72	1.06	<0.0005	0.111
Serum Z, μmol/l	40	0.07	0.04	0.14	0.07	46	0.07	0.05	0.19	0.11	<0.0005	0.005
Serum MZ, μmol/l	40	0.00	0.01	0.10	0.08	46	0.00	0.01	0.00	0.01	<0.0005	<0.0005
Serum TC, μmol/l	40	0.41	0.20	1.65	0.96	46	0.40	0.26	1.91	1.18	<0.0005	0.241

Macular pigment measured using the Macular Densitometer (Macular Metrics Corp.). Serum macular carotenoids analyzed by HPLC. Total carotenoids represent the total (composite) serum macular carotenoid concentrations (i.e., L, Z, and MZ combined). *N*, participants with data at all study visits. Sig., Significance set at $P < 0.05$.

* Group 1, 10 mg/d MZ, 10 mg/d L, and 2 mg/d Z plus 500 mg/d vitamin C, 400 IU/d of vitamin E, 25 mg/d zinc, and 2 mg/d copper.

† Group 2, 10 mg/d L, 2 mg/d Z plus 500 mg/d vitamin C, 400 IU/d of vitamin E, 25 mg/d zinc, and 2 mg/d copper.

Investigative Ophthalmology & Visual Science

Downloaded from iovs.arvojournals.org on 08/08/2023

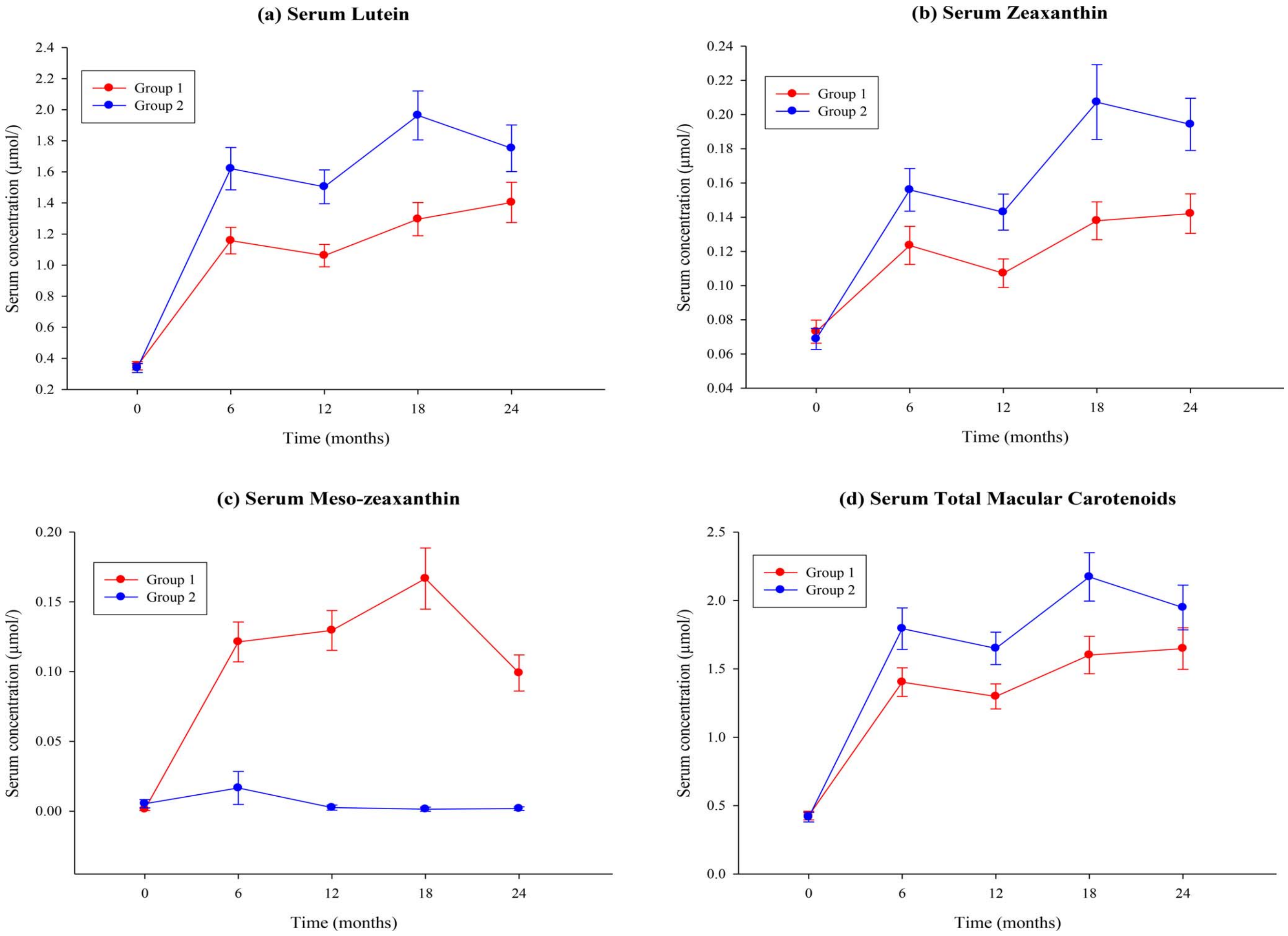

FIGURE 6. Serum carotenoid response in the CREST AMD study. Group 1, 10 mg/d MZ, 10 mg/d L, and 2 mg/d Z plus 500 mg/d vitamin C, 400 IU/d of vitamin E, 25 mg/d zinc, and 2 mg/d copper; group 2, 10 mg/d L, 2 mg/d Z plus 500 mg/d vitamin C, 400 IU/d of vitamin E, 25 mg/d zinc, and 2 mg/d copper. Serum macular carotenoids analyzed by HPLC. Serum total macular carotenoids represent the addition of serum lutein, zeaxanthin, and *meso*-zeaxanthin concentrations obtained at each study visit. *Error bars* represent standard error of mean.

magnitude of deterioration was demonstrable in only 2.2% to 13% of participants at the same three spatial frequencies.

GD, defined as reduction in visual function caused by a glare source, results in retinal contrast loss secondary to retinal straylight.[29,30] Clinically, GD can be measured by assessing the impact of a glare source on visual function (BCVA or CS) or by the measurement of retinal straylight.[30] Of note, the Commission Internationale de l'Eclairage defines GD in terms of retinal straylight.[29] For the purposes of this study, GD was measured using each of these aforementioned methods (i.e., by assessing CS under conditions of glare [in both mesopic and photopic conditions] using the Functional Vision Analyzer and by measuring retinal straylight using the Oculus C-Quant). Mechanisms put forward to explain the observed improvements in CS following MP augmentation in patients with nonadvanced AMD apply also to the observed improvements in GD in this population, but with the possibility of an additional element, which relates the glare hypothesis of MP.[31] The glare hypothesis of MP posits that MP augmentation should improve GD and PRT via its optical (blue light) filtration properties.[31] Of note, the absorption spectrum of MP[32] accounts for one third of the visible spectrum, and wavelengths of light responsible for GD are those in MP's absorption range.[31] Therefore, and given that MP filters short-wavelength light at a prereceptorial level, thereby reducing the adverse impact of retinal straylight (caused by the glare source) that casts a veiling luminance on the retina, the observed improvements in CS under conditions of glare (GD) are unsurprising.[31] Also, improvements in PRT following supplementation may also be explained, at least in part, by the glare hypothesis of MP.[31] In brief, MP attenuates short-wavelength light from the glare source before it reaches the photoreceptors, thereby reducing its impact on photopigment bleaching, and, consequently, reducing the recovery time (i.e., the time it takes for vision to be restored).

The observed improvement in reading speed as a consequence of supplementation may be attributed to visual and/or nonvisual (neurocognitive) factors. In terms of the visual factors, reading speed is a function of both spatial and temporal CS,[33] and we have already discussed the mechanisms whereby antioxidant supplementation resulted in an improvement in two aspects of spatial vision (CS and GD). In terms of temporal vision, it has been shown that MP is positively related to critical flicker fusion frequency and to the full temporal CS function measured at the fovea but not the parafovea.[34] Furthermore, supplemental macular carotenoids have been shown to increase critical flicker fusion frequency thresholds and visual motor reaction time in young healthy participants.[35] Thus, MP could improve reading speed by its effects on temporal vision (i.e., increasing temporal processing speeds). Indeed, Stringham and Stringham[36] have suggested that temporal visual mechanisms compensate for MP's optical

Downloaded from iovs.arvojournals.org on 08/08/2023

TABLE 6. Distribution of Adverse Events in the CREST AMD Study by Intervention Group

Adverse Events	Group 1, $n = 57$*	Group 2, $n = 61$†
Any adverse event	15	10
Ocular		
Watery eyes	1	1
Transient blurred vision	1	0
Gritty eyes	1	0
Ocular pain	1	
Bloodshot eyes	1	0
Nonocular		
Nausea	2	3
Tiredness	2	1
Vomiting	3	0
Itchy skin	1	1
Metallic taste in mouth	1	1
Heat rash	0	2
Irritable bowel syndrome	1	0
Night-time urination	1	0
Headaches	1	0
Weight gain	1	0
Overactive kidney	0	1
Leg cramps	1	0
Knee ache	1	0
Red and swollen arms and legs	0	1
Dizziness	1	0
Neck stiffness	1	0
Abdominal pains	0	1
Pancreatitis	0	1
Palpitations	1	0
Sleep disturbance	1	0
Swollen face	0	1
Hallucinations	0	1
Swollen ankle	0	1
Loss of appetite	0	1

Data expressed as number of participants. Some participants reported more than one adverse event.

* Group 1, 10 mg/d MZ, 10 mg/d L, and 2 mg/d Z plus 500 mg/d vitamin C, 400 IU/d of vitamin E, 25 mg/d zinc, and 2 mg/d copper.

† Group 2, 10 mg/d L, 2 mg/d Z plus 500 mg/d vitamin C, 400 IU/d of vitamin E, 25 mg/d zinc, and 2 mg/d copper.

filtration properties by reducing temporal input from the short-wavelength cone system and increasing temporal processing by the middle/long wavelength cone system. These aspects of temporal vision may be enhanced following supplementation with the macular carotenoids[35] and may lead to subsequent improvements in reading speed.

Vision-related quality of life questionnaires are known to correlate with subjective measures of visual function (e.g., CS and reading speed),[37] and, therefore, it is likely that improvements in these parameters will result in improved quality of life. Scilley et al.[38] reported that persons with nonadvanced AMD have good visual acuity, but are likely to have problems with night driving, near vision tasks, and GD when compared with persons with no retinal disease (age-matched controls with normal retinal health). Visual acuity, CS, and reading speed are known determinants of vision-related quality of life in patients with nonadvanced AMD,[39] reflected in the findings of the Los Angeles Latino Eye Study, where nonadvanced AMD lesions (i.e., soft indistinct drusen and pigmentary abnormalities) were associated with a lower self-reported vision-related quality of life.[40] Therefore, the observed improvements in visual function parameters (in the current study) are likely to impact favourably on quality of life of patients with nonadvanced AMD. However, our vision-related quality of life instrument (NEI VFQ-25) did not show any statistically significant improvements following supplementation with macular carotenoids (in combination with coantioxidants), and we suspect that a larger number of participants will be required to do so with such an instrument. For instance, to detect a two-point difference between interventions in the NEI VFQ-25 overall score (assuming a 5% level of significance, 80% power ,and two-tailed test), the required sample size would be 3136 participants (1568 per intervention group).[41]

Eye care professionals should be aware of the observed visual benefits afforded to patients with nonadvanced AMD as a result of supplementation with macular carotenoids (and coantioxidants) in the short, medium, and long terms, and the indication for recommending such supplements should no longer be limited to risk reduction for disease progression in the long term. Also, and importantly, further augmentation of MP and further improvements in psychophysical function are realized in patients with nonadvanced AMD after 24 months of sustained supplementation, and it may well be that the improvements observed in this study (duration of 24 months) understate the visual improvements that patients can expect.[9]

Given that psychophysical function is compromised in nonadvanced AMD in a way that is commensurate with the stage of nonadvanced AMD and given that AMD is a progressive disease, our findings of visual improvements in a condition where visual deterioration is expected is as interesting as it is welcome. If psychophysical visual function can be improved in a progressive condition (such as nonadvanced AMD), it is tempting to hypothesize that improvements in psychophysical function herald regression of the morphological changes that underpin them. However, longer term studies with larger numbers of patients with nonadvanced AMD, and with regular monitoring of MP and psychophysical function as well as morphological changes, are required to confirm or refute this hypothesis.

It is possible that some of our reported improvements in psychophysical measures of visual function (e.g., reading speed) may be due to learning effects, but given that we had no placebo group (which represents a limitation of our study) it is difficult to ascertain to what level (if any). It is also important to point out that reading speed was not a primary outcome measure in this trial. However, given the long periods of time between study visits, we feel that these learning effects are likely to be minimal. It should also be appreciated that these improvements in for example, reading speed, were observed in patients suffering from a condition associated with progressive visual deterioration and at a time of life when speed of neural processing declines.

Of note, the MP levels reported at baseline may be considered high.[42] This suggests that the current study was representative of a very well-nourished population and this may have, in fact, resulted in understating the benefits of supplementation that may have been seen in a less well-nourished population (as was the case in subgroup analyses of the AREDS2 cohort).[43]

In this study, we measured MP using two devices; namely the Densitometer (Macular Metrics) and the Spectralis HRA-OCT MultiColor (Heidelberg Engineering, GmbH, Heidelberg, Germany). In a previous report, using data from the current study, we found that measures of MP using these two devices are not impressively concordant, although each of these two devices is capable of detecting statistically significant changes in MP over time, within a given eye, following supplementation with MP's constituent carotenoids.[44] Furthermore, another recent study has found that MP measurement using the

Investigative Ophthalmology & Visual Science

Downloaded from iovs.arvojournals.org on 08/08/2023

Spectralis is affected by cataract.[45] Thus, in the current study, which included patients with varying severity of lens opacification, we elected (following advice from the DSMC) to use the MP measures from the Densitometer, which are robust to cataract.[46,47]

The strengths of this study include its randomized, controlled, and double-masked design, the range of parameters of visual function assessed, the fact that MP was measured and monitored using an established and validated technique, and the determination of serological responses and that AMD was graded in a masked fashion by an accredited reading center. Finally, the study was overseen by an independent DSMC.

A study limitation (albeit slight) is the failure to reach the intended sample size of 56 participants per group; actual samples sizes were 51 and 46. However, because we elected to use repeated measures analysis of variance, rather than the independent-samples *t*-tests on which the original sample size calculations had been based, our statistical tests (of time and time × supplement interaction effects) were based on the t-distribution with more than 90 degrees of freedom, that is, these tests were more than adequately powered.

Another study limitation is the absence of a placebo arm. However, as already noted, the original study protocol had a true placebo, but that protocol had to be revised on ethical grounds, following publication of the AREDS2 findings. We did not measure the serum concentrations of any of the co-antioxidants (vitamin C, E, zinc, and copper) in this RCT. We do, however, report serum response of the macular carotenoids, which was important in the assessment of compliance, and that allowed us to investigate whether participants were responding to the nutrients of interest. Assessing the concentrations of the coantioxidants may have yielded insights into the interrelationships/interactions between these compounds and the macular carotenoids, and future studies may consider adopting such an approach. Correction for multiple testing was not performed in the current study. It is therefore possible that some of our reported significant results may be attributable to type 1 errors. However, many of the reported *P* values in this study would still be significant after Bonferroni adjustment.

In summary, supplementation with a formulation that contains the macular carotenoids (with or without MZ), in combination with coantioxidants, results in improvements in contrast sensitivity and other measures of visual function in patients with nonadvanced AMD.

Acknowledgments

The authors thank the Whitfield Clinic Pharmacy, Waterford, Ireland, for their support with randomization and intervention assignment (Catherine Kelly [Chief Pharmacist] and Lisa O'Brien [Pharmacy Technician]). They also thank Moorfields Eye Hospital Reading Centre, London, United Kingdom, for retinal photograph grading where Tunde Peto and Irene Leung have been funded by the National Institute of Health Research Biomedical Research Centre at Moorfields Eye Hospital and University College London Institute of Ophthalmology. They also thank Elizabeth Johnson from Tufts University, Boston, Massachusetts, United States, for permission to use the "L/Z screener" for estimating dietary intake of lutein and zeaxanthin in this study. They also thank the CREST Data Safety and Monitoring Committee (James Loughman [Chairperson and Vision Scientist], Ailbhe Whyte [Medical Ophthalmologist], Michael Harrison [Research Ethics Committee member], and Frank Leonard [Statistician]) for their support and guidance during the Central Retinal Enrichment Supplementation Trials study.

Supported by the European Research Council Grant 281096.

Disclosure: **K.O. Akuffo**, None; **S. Beatty**, None; **T. Peto**, None; **J. Stack**, None; **J. Stringham**, None; **D. Kelly**, None; **I. Leung**, None; **L. Corcoran**, None; **J.M. Nolan**, None

References

1. Wong WL, Su X, Li X, et al. Global prevalence of age-related macular degeneration and disease burden projection for 2020 and 2040: a systematic review and meta-analysis. *Lancet Global Health*. 2014;2:e106–e116.
2. Akuffo KO, Nolan J, Stack J, et al. Prevalence of age-related macular degeneration in the Republic of Ireland. *Br J Ophthalmol*. 2015;99:1037–1044.
3. Williams RA, Brody BL, Thomas RG, Kaplan RM, Brown SI. The psychosocial impact of macular degeneration. *Arch Ophthalmol*. 1998;116:514–520.
4. Brown GC, Brown MM, Sharma S, et al. The burden of age-related macular degeneration: a value-based medicine analysis. *Trans Am Ophthalmol Soc*. 2005;103:173–184.
5. Bernstein PS, Li B, Vachali PP, et al. Lutein, zeaxanthin, and meso-zeaxanthin: the basic and clinical science underlying carotenoid-based nutritional interventions against ocular disease. *Prog Retin Eye Res*. 2016;50:34–66.
6. Chew EY, Clemons T, SanGiovanni JP, et al. The Age-Related Eye Disease Study 2 (AREDS2): study design and baseline characteristics (AREDS2 report number 1). *Ophthalmology*. 2012;119:2282–2289.
7. Age Related Eye Disease Study 2 Research Group. Lutein + zeaxanthin and omega-3 fatty acids for age-related macular degeneration: the Age-Related Eye Disease Study 2 (AREDS2) randomized clinical trial. *JAMA*. 2013;309:2005–2015.
8. Liu R, Wang T, Zhang B, et al. Lutein and zeaxanthin supplementation and association with visual function in age-related macular degeneration. *Invest Ophthalmol Vis Sci*. 2015;56:252–258.
9. Akuffo KO, Nolan JM, Howard AN, et al. Sustained supplementation and monitored response with differing carotenoid formulations in early age-related macular degeneration. *Eye (Lond)*. 2015;29:902–912.
10. Crosby-Nwaobi R, Hykin P, Peto T, Sivaprasad S. An exploratory study evaluating the effects of macular carotenoid supplementation in various retinal diseases. *Clin Ophthalmol*. 2016;10:835–844.
11. Akuffo KO, Beatty S, Stack J, et al. Central Retinal Enrichment Supplementation Trials (CREST): design and methodology of the CREST randomized controlled trials. *Ophthalmic Epidemiol*. 2014;21:111–123.
12. Davis MD, Gangnon RE, Lee LY, et al. The Age-Related Eye Disease Study severity scale for age-related macular degeneration: AREDS report no. 17. *Arch Ophthalmol*. 2005;123:1484–1498.
13. Schulz KF, Altman DG, Moher D. CONSORT 2010 statement: updated guidelines for reporting parallel group randomised trials. *Trials*. 2010;11:32.
14. Ma L, Yan SF, Huang YM, et al. Effect of lutein and zeaxanthin on macular pigment and visual function in patients with early age-related macular degeneration. *Ophthalmology*. 2012;119:2290–2297.
15. Huang YM, Dou HL, Huang FF, Xu XR, Zou ZY, Lin XM. Effect of supplemental lutein and zeaxanthin on serum, macular pigmentation, and visual performance in patients with early age-related macular degeneration. *Biomed Res Int*. 2015;2015:564738.
16. Wooten BR, Hammond BR. Macular pigment: influences on visual acuity and visibility. *Prog Retin Eye Res*. 2002;21:225–240.
17. Hammond BR Jr, Fletcher LM. Influence of the dietary carotenoids lutein and zeaxanthin on visual performance:

Investigative Ophthalmology & Visual Science

Downloaded from iovs.arvojournals.org on 08/08/2023

application to baseball. *Am J Clin Nutr*. 2012;96:1207S–1213S.
18. Fletcher LM, Engles M, Hammond BR Jr. Visibility through atmospheric haze and its relation to macular pigment. *Optom Vis Sci*. 2014;91:1089–1096.
19. Hammond BR Jr, Wooten BR, Engles M, Wong JC. The influence of filtering by the macular carotenoids on contrast sensitivity measured under simulated blue haze conditions. *Vision Res*. 2012;63:58–62.
20. Stringham JM, O'Brien KJ, Stringham NT. Contrast sensitivity and lateral inhibition are enhanced with macular carotenoid supplementation. *Invest Ophthalmol Vis Sci*. 2017;58:2291–2295.
21. Maynard ML, Zele AJ, Feigl B. Mesopic Pelli-Robson contrast sensitivity and MP-1 microperimetry in healthy ageing and age-related macular degeneration. *Acta Ophthalmol*. 2016;94:e772–e778.
22. Neelam K, Nolan J, Chakravarthy U, Beatty S. Psychophysical function in age-related maculopathy. *Surv Ophthalmol*. 2009;54:167–210.
23. Owsley C, Sloane ME. Contrast sensitivity, acuity, and the perception of "real-world" targets. *Br J Ophthalmol*. 1987;71:791–796.
24. Charalampidou S, Loughman J, Nolan J, et al. Prognostic indicators and outcome measures for surgical removal of symptomatic nonadvanced cataract. *Arch Ophthalmol*. 2011;129:1155–1161.
25. Kaiser PK. Prospective evaluation of visual acuity assessment: a comparison of snellen versus ETDRS charts in clinical practice (an AOS thesis). *Trans Am Ophthalmol Soc*. 2009;107:311–324.
26. Leat SJ, Legge GE, Bullimore MA. What is low vision? A re-evaluation of definitions. *Optom Vis Sci*. 1999;76:198–211.
27. Pelli DG, Bex P. Measuring contrast sensitivity. *Vision Res*. 2013;90:10–14.
28. Owsley C, McGwin G Jr. Vision impairment and driving. *Surv Ophthalmol*. 1999;43:535–550.
29. Vos JJ. Disability glare—a state of the art report. *Commission Internationale de l'Eclairage J*. 1984;3:39–53.
30. Mainster MA, Turner PL. Glare's causes, consequences, and clinical challenges after a century of ophthalmic study. *Am J Ophthalmol*. 2012;153:587–593.
31. Stringham JM, Hammond BR Jr. The glare hypothesis of macular pigment function. *Optom Vis Sci*. 2007;84:859–864.
32. Snodderly DM, Brown PK, Delori FC, Auran JD. The macular pigment. I. Absorbance spectra, localization, and discrimination from other yellow pigments in primate retinas. *Invest Ophthalmol Vis Sci*. 1984;25:660–673.
33. Legge GE, Rubin GS, Luebker A. Psychophysics of reading—V. The role of contrast in normal vision. *Vision Res*. 1987;27:1165–1177.
34. Renzi LM, Hammond BR Jr. The relation between the macular carotenoids, lutein and zeaxanthin, and temporal vision. *Ophthalmic Physiol Opt*. 2010;30:351–357.
35. Bovier ER, Hammond BR. A randomized placebo-controlled study on the effects of lutein and zeaxanthin on visual processing speed in young healthy subjects. *Arch Biochem Biophys*. 2015;572:54–57.
36. Stringham NT, Stringham JM. Temporal visual mechanisms may mediate compensation for macular pigment. *Perception*. 2015;44:1400–1415.
37. Orr P, Rentz AM, Margolis MK, et al. Validation of the National Eye Institute Visual Function Questionnaire-25 (NEI VFQ-25) in age-related macular degeneration. *Invest Ophthalmol Vis Sci*. 2011;52:3354–3359.
38. Scilley K, Jackson GR, Cideciyan AV, Maguire MG, Jacobson SG, Owsley C. Early age-related maculopathy and self-reported visual difficulty in daily life. *Ophthalmology*. 2002;109:1235–1242.
39. Maguire M. Baseline characteristics, the 25-Item National Eye Institute Visual Functioning Questionnaire, and their associations in the Complications of Age-Related Macular Degeneration Prevention Trial (CAPT). *Ophthalmology*. 2004;111:1307–1316.
40. Choudhury F, Varma R, Klein R, Gauderman WJ, Azen SP, McKean-Cowdin R. Age-related macular degeneration and quality of life in Latinos: The Los Angeles Latino Eye Study. *JAMA Ophthalmol*. 2016;134:683–690.
41. Mangione CM, Lee PP, Gutierrez PR, Spritzer K, Berry S, Hays RD. Development of the 25-item National Eye Institute Visual Function Questionnaire. *Arch Ophthalmol*. 2001;119:1050–1058.
42. Akuffo KO, Nolan JM, Peto T, et al. Relationship between macular pigment and visual function in subjects with early age-related macular degeneration. *Br J Ophthalmol*. 2016;101:190–197.
43. Chew EY, Clemons TE, SanGiovanni JP, et al. Secondary analyses of the effects of lutein/zeaxanthin on age-related macular degeneration progression: AREDS2 report no. 3. *JAMA Ophthalmol*. 2013;132:142–149.
44. Akuffo KO, Beatty S, Stack J, et al. Concordance of macular pigment measurement using customized heterochromatic flicker photometry and fundus autofluorescence in age-related macular degeneration. *Invest Ophthalmol Vis Sci*. 2015;56:8207–8214.
45. Akuffo KO, Nolan JM, Stack J, et al. the impact of cataract, and its surgical removal, on measures of macular pigment using the Heidelberg Spectralis HRA+OCT multicolor device effect of cataract on Spectralis MP measurement. *Invest Ophthalmol Vis Sci*. 2016;57:2552–2563.
46. Ciulla TA, Hammond BR Jr, Yung CW, Pratt LM. Macular pigment optical density before and after cataract extraction. *Invest Ophthalmol Vis Sci*. 2001;42:1338–1341.
47. Nolan JM, O'Reilly P, Loughman J, et al. Augmentation of macular pigment following implantation of blue light-filtering intraocular lenses at the time of cataract surgery. *Invest Ophthalmol Vis Sci*. 2009;50:4777–4785.

Downloaded from iovs.arvojournals.org on 08/08/2023
