# Supplementary material for: Insights into the nutritional prevention of macular degeneration based on a comparative topic modeling approach
Source: PeerJ Comput Sci. 2024 Mar 20;10:e1940. doi: 10.7717/peerj-cs.1940 (PMC11042009; doi:10.7717/peerj-cs.1940)
Supplement: Supplemental Information 2 [file peerj-cs-10-1940-s002.zip › positive_result_reports/jama_272_18_032.pdf]

Dietary Carotenoids, Vitamins A, C, and E, and Advanced Age-Related Macular Degeneration

Johanna M. Seddon, MD; Umed A. Ajani, MBBS; Robert D. Sperduto, MD; Rita Hiller, MS; Norman Blair, MD; Thomas C. Burton, MD; Marilyn D. Farber, PhD; Evangelos S. Gragoudas, MD; Julia Haller, MD; Dayton T. Miller, PhD; Lawrence A. Yannuzzi, MD; Walter Willett, MD; for the Eye Disease Case-Control Study Group

Objective.—To evaluate the relationships between dietary intake of carotenoids and vitamins A, C, and E and the risk of neovascular age-related macular degeneration (AMD), the leading cause of irreversible blindness among adults.

Design.—The multicenter Eye Disease Case-Control Study.

Setting.—Five ophthalmology centers in the United States.

Patients.—A total of 356 case subjects who were diagnosed with the advanced stage of AMD within 1 year prior to their enrollment, aged 55 to 80 years, and residing near a participating clinical center. The 520 control subjects were from the same geographic areas as case subjects, had other ocular diseases, and were frequency-matched to cases according to age and sex.

Main Outcome Measures.—The relative risk for AMD was estimated according to dietary indicators of antioxidant status, controlling for smoking and other risk factors, by using multiple logistic-regression analyses.

Results.—A higher dietary intake of carotenoids was associated with a lower risk for AMD. Adjusting for other risk factors for AMD, we found that those in the highest quintile of carotenoid intake had a 43% lower risk for AMD compared with those in the lowest quintile (odds ratio, 0.57; 95% confidence interval, 0.35 to 0.92; *P* for trend=.02). Among the specific carotenoids, lutein and zeaxanthin, which are primarily obtained from dark green, leafy vegetables, were most strongly associated with a reduced risk for AMD (*P* for trend=.001). Several food items rich in carotenoids were inversely associated with AMD. In particular, a higher frequency of intake of spinach or collard greens was associated with a substantially lower risk for AMD (*P* for trend<.001). The intake of preformed vitamin A (retinol) was not appreciably related to AMD. Neither vitamin E nor total vitamin C consumption was associated with a statistically significant reduced risk for AMD, although a possibly lower risk for AMD was suggested among those with higher intake of vitamin C, particularly from foods.

Conclusion.—Increasing the consumption of foods rich in certain carotenoids, in particular dark green, leafy vegetables, may decrease the risk of developing advanced or exudative AMD, the most visually disabling form of macular degeneration among older people. These findings support the need for further studies of this relationship.

(*JAMA*. 1994;272:1413-1420)

AGE-RELATED macular degeneration (AMD) is the leading cause of irreversible blindness among persons older than 65 years.[1,2] Activities essential for independent living, including reading, driving, and writing, are most impaired by the loss of central vision due to this disease that affects the macula, the small central part of the retina. The prevalence of AMD and the associated social and economic consequences of blindness from AMD are increasing as the number of older people in our population continues to increase. However, despite the significance of this disease, there are no available means to prevent it, and effective treatment is limited to only a small fraction of patients. Strategies to prevent or retard the onset of AMD are needed so that the burden of blindness and visual impairment due to this disease can be reduced.

See also pp 1439 and 1455.

There is increasing speculation that dietary factors, particularly antioxidants, may prevent or impede the progression of AMD.[3-11] The theory is biologically plausible. The outer retina, rich in polyunsaturated fatty acids, may be altered adversely by free-radical production and oxidation and, conversely, may be protected by nutrients that block this oxidative damage. Antioxidants may also help to maintain the integrity of the choroidal blood vessels that supply the macular region of the retina.

Basic and clinical research suggest that nutritional factors may be associated with AMD. Animals of several species, including primates, that are deprived of nutrients with antioxidant potential are more prone than those not deprived of such nutrients to develop retinal degeneration, an effect that is enhanced by bright light. Animals that are given nutrients that have antioxidant potential are less likely to demon-

From the Epidemiology Unit (Drs Seddon and Ajani) and the Retina Service (Drs Seddon and Gragoudas), the Department of Ophthalmology, Massachusetts Eye and Ear Infirmary, and the Channing Laboratory (Dr Willett), Harvard Medical School, Boston, Mass; the Department of Epidemiology (Drs Seddon and Willett) and the Department of Nutrition (Dr Willett), Harvard School of Public Health, Boston; the National Eye Institute, Bethesda, Md (Dr Sperduto and Ms Hiller); the Department of Ophthalmology, University of Illinois at Chicago (Drs Blair and Farber); the Department of Ophthalmology, Medical College of Wisconsin, Milwaukee (Dr Burton); the Wilmer Eye Institute, Baltimore, Md (Dr Haller); the Division of Environmental Health Laboratory Sciences, Centers for Disease Control and Prevention, Atlanta, Ga (Dr Miller); the Manhattan Eye, Ear, and Throat Hospital, New York, NY (Dr Yannuzzi); and the Brigham and Women's Hospital, Boston, Mass (Dr Willett).

A complete list of the participants in the Eye Disease Case-Control Study Group was published in the following article: The Eye Disease Case-Control Study Group. Risk factors for neovascular age-related macular degeneration. *Arch Ophthalmol.* 1992;110:1701-1708.

Presented in part at the annual meeting of the Association for Research in Vision and Ophthalmology, Sarasota, Fla, May 5, 1993.

Reprint requests to the Epidemiology Unit, Massachusetts Eye and Ear Infirmary, 243 Charles St, Boston, MA 02114 (Dr Seddon).

Downloaded From: https://jamanetwork.com/ by a SCELC - University of Southern California User on 07/17/2023

strate retinal degeneration than those who are not given such nutrients.[3-7] Only a few descriptive and observational epidemiologic studies[9-12] and one small randomized trial[13] have addressed the hypothesis that antioxidants can reduce the occurrence of AMD. The results of most studies,[9,10,12,13] but not all,[11] have supported this hypothesis, but none have evaluated dietary intake among a large number of persons with advanced AMD.

Inverse associations between exudative AMD and blood levels of antioxidant vitamins have been noted previously in the study population evaluated herein.[10] It is important to assess dietary intake in addition to blood values of nutrients for several reasons.[14] First, biochemical markers can be influenced by nondietary factors, such as absorption and other metabolic factors. Second, practically speaking, people choose certain foods to eat, and not specific nutrients. Finally, information obtained from a dietary questionnaire as used in this study reflects long-term dietary intake and, thus, complements the information obtained by blood analyses, which generally reflects short-term intake.

To explore the potential association of dietary intake of antioxidants with risk for AMD, we compared case subjects who had AMD with control subjects who had other eye problems by evaluating their intake of antioxidant nutrients, their use of vitamin supplements, and their consumption of specific food items. Because smoking increases oxidative stress and lipid peroxidation,[15] reduces plasma concentrations of antioxidants,[16,17] and is associated with risk for AMD,[18] we also hypothesized that any observed relationship between the consumption of dietary levels of nutrients and AMD might be modified by cigarette smoking.

METHODS

The Eye Disease Case-Control Study (EDCCS),[10] sponsored by the National Eye Institute, Bethesda, Md, was designed to evaluate the role of potential risk factors for a number of retinal disorders, including advanced AMD. The dietary component was an ancillary study initiated and directed by the principal investigator of the clinical center in Boston (J.M.S.). Institutional review boards at each participating center approved the main and ancillary studies.

Case and Control Subjects

A detailed description of the identification of study subjects is described elsewhere.[10,18] Case and control subjects were selected between May 1986 and December 1990 from patients treated at the following five large ophthalmology centers: the Manhattan Eye, Ear, and Throat Hospital in New York City; the Massachusetts Eye and Ear Infirmary in Boston, Mass; the Medical College of Wisconsin at Milwaukee; the Illinois Eye and Ear Infirmary, University of Illinois at Chicago; and the Wilmer Eye Institute in Baltimore, Md.

Eligible case subjects were people 55 to 80 years of age in whom the advanced or exudative neovascular form of AMD was diagnosed within 1 year of their enrollment into the study and who resided in or near the community in which the clinical center was located as defined by ZIP code listing. They were identified by review of ocular photography lists and screening of medical records. In addition, physicians were asked to refer potential case and control subjects who met the criteria for the study. Diagnostic criteria for AMD included a visual acuity of less than 20/20 in the affected eye or distortion on the Amsler grid, drusen in either eye, and at least one of the following signs of exudative AMD: (1) macular fibrous scar; and/or (2) subretinal hemorrhage or fluorescein angiographic signs of neovascularization with one or more of the following clinical signs involving the macula: a neurosensory detachment, lipid deposits, gray subretinal membrane, or a retinal pigment epithelium detachment. Patients with ocular diseases other than AMD that are associated with subretinal neovascularization and patients with a history of intraocular surgery were excluded from both groups. All cases of AMD were confirmed with an examination by a retina specialist, as well as by fundus photography and fluorescein angiography. Eighty-two percent of the case subjects identified as eligible for the study agreed to participate.

Control subjects were enrolled concurrently with the case subjects and were selected from a similar pool of outpatients who had undergone a complete dilated ocular examination and did not have the diseases under study. Eligible controls were identified from the same general population and geographic area as the case subjects and resided in or near the community in which the clinical center was located as defined by ZIP code listing. Potential controls were identified using the same sources from which the cases were identified. For example, if a general ophthalmology clinic was used to identify cases, then general clinic records were used to screen for controls. When cases were identified from specialty clinics, controls were selected from the same source or from other specialty clinics. The diagnoses among enrolled controls included lid disorders (31%), vitreous disorders (18%), cataract (14%), conjunctivitis (10%), other retinal disorders (8%), corneal problems (7%), and a miscellaneous group of other diagnoses (12%). Control subjects were diagnosed with these disorders within 1 year of their enrollment into the study.

Seventy-eight percent of the control subjects identified as eligible for the study agreed to participate. Reasons for nonparticipation were similarly distributed among cases and controls and included lack of interest, being too busy, or unable to miss work. The cases and controls were frequency-matched on age, sex, and clinic. The balance between cases and controls on these factors was monitored quarterly by the coordinating center.

Data Collection

Data were derived from a standardized interview, a limited physical examination, an ophthalmic examination, and a laboratory analyses of blood specimens. A semiquantitative food-frequency questionnaire (FFQ) was also performed at all five centers. The FFQ contains a list of 60 food items that were selected as the major sources of a variety of nutrients. We modified an extensively validated questionnaire[19] to facilitate its use among elderly subjects with eye disease. This dietary instrument was found to be reliable in this study population.[20] Reproducibility correlation coefficients for carotenoids (r=.61), vitamin C with supplements (r=.66), and vitamin E with supplements (r=.69) were similar to results from other established diet questionnaires.[14]

Questionnaires were mailed to the participants before their study visit. Participants completed the FFQ at home or in the clinic; 58% of cases and 49% of controls completed them mainly in the clinic. On the day of the study visit, we assisted people who had questions about the form. Participants were asked to indicate the average frequency of consumption for each food item during the past year. Each food was specified in a standardized portion size. Options for frequency ranged from "almost never" to "6+ per day," with a total of nine possibilities. The FFQ also included questions about the use of multivitamins and supplements containing vitamins A, C, and E, selenium, iron, zinc, and calcium. Users of individual supplements were asked to report years of use (≤1, 2 to 4, 5 to 9, or 10 or more years). There was also an open-ended question regarding the intake of any other supplements.

We reviewed, coded, and entered the data from all questionnaires into the computer without knowledge of the case or control status of the participant, using data-entry software with a double-en-

Downloaded From: https://jamanetwork.com/ by a SCELC - University of Southern California User on 07/17/2023

try and verification system as a quality control measure. For this FFQ, we used a computer program that was developed at the Channing Laboratory in Boston to generate the intake scores for various micronutrients. The scores were calculated by summing the nutrient contribution of each food item and multiplying it by its reported frequency of use. Nutrient values were primarily derived from US Department of Agriculture sources.[19] We used the published vitamin A activity (in international units) for fruits and vegetables plus one third of the vitamin A activity of dairy foods to estimate the carotenoid value of these foods. Thus, these values represent only carotenoids with provitamin A activity, largely beta carotene intake with smaller contributions from other carotenoids. A recently published database[21] was used to estimate the intake of the specific carotenoids beta carotene, alpha carotene, lutein and zeaxanthin (lutein/zeaxanthin), lycopene, and beta cryptoxanthin.

The FFQ was added as an ancillary study shortly after the onset of the main project. Of the 1036 enrolled subjects, 922 (89%) completed the diet questionnaire. To maintain the quality of data, we excluded 46 of these questionnaires because of the following reasons: inadequate answers (n=17), extreme values (n=24), or missing information (n=5). Therefore, we used a total of 876 observations, including 356 (85%) of the 421 case subjects and 520 (85%) of the 615 control subjects for these dietary analyses. Participants whose responses were not included in the analyses (n=160) did not differ significantly from those included (n=876) with regard to age (median age, 69 years vs 70 years, respectively), sex (61% vs 55% female, respectively), or education (at least high school degree, 72% vs 76%, respectively) (P>.05 for all comparisons).

Analyses

We used the nutrient values adjusted for total energy intake that have been described elsewhere.[22] Since the amount of a nutrient derived from supplements is not correlated with total energy intake, we adjusted only the food component of the nutrient intake and then added it to the supplemented amount in those instances where supplements were used. We computed energy-adjusted intakes as the difference between expected and observed nutrient intake (residuals), using a simple linear regression with the log of the nutrient modeled as the dependent variable and the log of the total energy intake as the independent variable. The residuals were added to a constant value (the predicted log nutrient for the population mean of the log of energy intake) to avoid negative values and exponentiated to return to the original scale. These adjusted nutrients represent the nutrient composition of diet with the total energy intake held constant.

Energy-adjusted nutrient scores were categorized into quintiles of intake on the basis of the distribution of nutrient scores among control subjects. We calculated nutrient intake both including and excluding the contribution from vitamin supplements. Short-term users of vitamin A, C, and E supplements (people who used supplements for 1 year or less) were excluded from the analyses related to total vitamin intake including supplements because a diagnosis of eye disease among these people may have influenced their recent supplement use. Analyses also included the association between the risk for AMD and the intake of specific food items. We evaluated the use of supplements independent of food intake and categorized the current users of vitamin C and vitamin E supplements according to the duration of their use of supplements. There were too few current vitamin A users to categorize their use of supplements by duration, and data on the duration of use were not available for multivitamin users.

Odds ratios (ORs) and 95% confidence intervals (CIs) were calculated using multiple logistic regression. Two-sided P values for the χ^2 test for trend were also calculated. Adjusted ORs were calculated to control initially for age, sex, and clinical center. We also constructed multivariate models to control for factors potentially related to dietary intake and to the risk for AMD. Therefore, in addition to age, sex, clinical center, and education (greater than, less than, or equal to 12 years of school), these models also included smoking status (never smoked, former smoker, current smoker), alcohol intake (grams per day), body mass index, measured systolic blood pressure (mm Hg), and self-reported physical activity level (below average, average, above average).

Stratified analyses were performed to evaluate the possible modification of the effect of antioxidant intake on the risk for AMD by smoking status (never smoked, past smoker, current smoker). The referent was the lowest quintile of intake for each nutrient among nonsmokers.

RESULTS

The average age of participants was 71 years among cases (range, 55 to 80 years) and 68 years among controls (range, 55 to 80 years). Fifty-six percent of case subjects and 55% of control subjects were female. Advanced AMD is uncommon among nonwhites and only six were enrolled in this study. Since genetic and cultural factors may be related to the low prevalence of AMD in nonwhites and there were insufficient numbers of cases to address this possibility, analyses were restricted to whites.

Nutrient and Supplement Intake

The median nutrient value for each quintile and the ORs for advanced AMD according to the quintile of nutrient intake are displayed in Table 1. Higher levels of carotenoid intake were associated with a reduced risk for exudative neovascular AMD. There was a statistically significant, apparently linear trend for a reduction in risk for AMD with increasing amount of carotenoids in the food. Controlling for other potential risk factors, we estimated the risk for AMD to be reduced by 43% among people whose consumption of dietary carotenoids placed them in the highest quintile of dietary carotenoid intake compared with those in the lowest quintile (OR, 0.57; 95% CI, 0.35 to 0.92; P for trend=.02). In an analysis using an alternate classification of smoking, defined as number of cigarettes smoked per day among current smokers, we found that the OR for carotenoid intake remained essentially unchanged (OR, 0.56). When carotenoid fractions were evaluated separately, beta carotene and lutein/zeaxanthin were associated with a statistically significant trend for reduction in risk for AMD with higher intake, with an OR of 0.59 for beta carotene and 0.43 for lutein/zeaxanthin, comparing the highest to the lowest quintile of intake for each (Table 2). We also conducted an analysis with beta carotene and lutein/zeaxanthin, (correlation coefficient, 0.51) in the model simultaneously. In that model, the inverse association between AMD and beta carotene was reduced (OR, 0.91; 95% CI, 0.5 to 1.6), but a clear and strong inverse association with lutein/zeaxanthin remained (OR, 0.44; 95% CI, 0.2 to 0.8; P for trend<.008) (Table 2). A formal test of the difference in slopes between beta carotene and lutein/zeaxanthin was not statistically significant (P=.17).

Total vitamin A, which is composed mainly of carotenoid, was also associated with a reduced risk for AMD in the multivariate model (P for trend=.02) (Table 1). Retinol (preformed vitamin A) intake was not associated with AMD. Total vitamin C intake had no consistent association with AMD. However, the results for vitamin C intake from foods, excluding supplement use, suggested a protective effect that was not statistically significant (OR, 0.83 in the multivariate model), although, the OR was closer to 1.0 when carotenoid intake was also in-

Downloaded From: https://jamanetwork.com/ by a SCELC - University of Southern California User on 07/17/2023

Table 1.—Odds Ratios for Exudative Age-Related Macular Degeneration by Quintile of Energy-Adjusted Nutrient Intake*

Nutrient	Quintiles 1	2	3	4	5	*P* (Trend)
Carotenoids						
Cases/Controls, No.	85/104	73/104	75/104	61/104	62/104	. . .
Median intake, IU	3154	5181	7636	11393	19250	. . .
Adjusted OR†	1.0	0.72	0.71	0.58	0.49	.002
Multivariate OR (95% CI)‡	1.0	0.78 (0.49-1.23)	0.78 (0.49-1.23)	0.65 (0.41-1.05)	0.57 (0.35-0.92)	.02
Total retinol						
Cases/Controls, No.	55/104	71/104	81/104	72/104	77/104	. . .
Median intake, IU	837	1565	2348	5357	10614	. . .
Adjusted OR†	1.0	1.45	1.56	1.23	1.25	.69
Multivariate OR (95% CI)‡	1.0	1.51 (0.92-2.48)	1.60 (0.98-2.60)	1.28 (0.78-2.09)	1.34 (0.82-2.17)	.53
Retinol without supplements						
Cases/Controls, No.	70/104	75/104	57/104	74/104	80/104	. . .
Median intake, IU	627	1278	1699	2287	3789	. . .
Adjusted OR†	1.0	1.14	0.82	1.09	1.13	.69
Multivariate OR (95% CI)‡	1.0	1.16 (0.73-1.84)	0.85 (0.52-1.39)	1.16 (0.72-1.85)	1.04 (0.65-1.67)	.88
Total vitamin A including supplements§						
Cases/Controls, No.	72/104	83/104	65/103	62/103	56/100	. . .
Median intake, IU	5294	8205	11831	16633	25551	. . .
Adjusted OR†	1.0	0.87	0.70	0.65	0.49	.002
Multivariate OR (95% CI)‡	1.0	0.94 (0.60-1.50)	0.75 (0.46-1.21)	0.73 (0.45-1.19)	0.56 (0.35-0.96)	.02
Vitamin A without supplements						
Cases/Controls, No.	72/104	80/104	77/104	63/104	64/104	. . .
Median intake, IU	4690	7127	9792	13541	21262	. . .
Adjusted OR†	1.0	0.97	0.88	0.66	0.61	.01
Multivariate OR (95% CI)‡	1.0	1.14 (0.72-1.82)	0.96 (0.60-1.54)	0.76 (0.47-1.24)	0.71 (0.44-1.16)	.05
Total vitamin C including supplements§						
Cases/Controls, No.	53/104	75/104	83/104	69/102	56/96	. . .
Median intake, mg	64.9	111.9	158.5	268.1	1039.1	. . .
Adjusted OR†	1.0	1.26	1.31	1.06	0.82	.31
Multivariate OR (95% CI)‡	1.0	1.43 (0.88-2.33)	1.39 (0.86-2.26)	1.38 (0.84-2.27)	1.01 (0.60-1.70)	.98
Vitamin C without supplements						
Cases/Controls, No.	75/104	83/104	67/104	62/104	69/104	. . .
Median intake, mg	54	94	123	153	213	. . .
Adjusted OR†	1.0	0.98	0.74	0.71	0.66	.03
Multivariate OR (95% CI)‡	1.0	1.12 (0.70-1.77)	0.84 (0.53-1.35)	0.82 (0.51-1.34)	0.83 (0.52-1.33)	.22
Total vitamin E including supplements§						
Cases/Controls, No.	50/104	71/104	81/104	74/104	50/94	. . .
Median intake, mg‖	3.38	4.81	6.48	20.3	405.5	. . .
Adjusted OR†	1.0	1.33	1.39	1.15	0.87	.45
Multivariate OR (95% CI)‡	1.0	1.52 (0.92-2.50)	1.56 (0.95-2.56)	1.34 (0.81-2.22)	1.07 (0.63-1.84)	.98
Vitamin E without supplements						
Cases/Controls, No.	46/104	62/104	75/104	90/104	83/104	. . .
Median intake, mg‖	3	4	5	6	8	. . .
Adjusted OR†	1.0	1.34	1.47	1.59	1.38	.17
Multivariate OR (95% CI)‡	1.0	1.27 (0.76-2.13)	1.60 (0.96-2.68)	1.72 (1.04-2.84)	1.46 (0.88-2.44)	.09

*OR indicates odds ratio; CI, confidence interval; and ellipses, not applicable.

†Adjusted for age, sex, and clinic.

‡Included terms for age (continuous), sex, clinic (Massachusetts, New York, Illinois, Wisconsin, Maryland), education (>, <, or = 12th grade), systolic blood pressure (mm Hg), self-reported physical activity level (average, below average, above average), alcohol intake (grams per day), body mass index, and smoking status (never smoker, former smoker, current smoker).

§Number of cases and controls varies because of the exclusion of short-term (≤1 y) current users of vitamin supplements.

‖Milligrams of tocopherol equivalents.

cluded in the model (OR, 0.89; 95% CI, 0.55 to 1.44). Increased vitamin E intake, both with and without supplements, was associated with a nonsignificant elevated risk for AMD, but the magnitude of effect did not appear to increase with increasing intake of vitamin E.

When we evaluated vitamin supplement use separately from food intake (Table 3), we noted a nonsignificant inverse association between risk for AMD and multivitamin use (OR, 0.82; 95% CI, 0.57 to 1.18). Overall, no beneficial effects of supplementation with vitamins A or E were noted. When we categorized current users of vitamin C and E by duration of supplement use, we noted a contrast between short-term and long-term users. Short-term use (≤1 year) suggested an adverse effect for both vitamins, whereas long-term use suggested no effect (vitamin E) or a possible small beneficial effect (vitamin C).

Food Intake

Since we noted a statistically significant inverse association between the risk

Downloaded From: https://jamanetwork.com/ by a SCELC - University of Southern California User on 07/17/2023

Table 2.—Odds Ratios for Exudative Age-Related Macular Degeneration by Quintile of Energy-Adjusted Nutrient Intake*

Nutrient	Quintiles 1	2	3	4	5	*P* (Trend)
Alpha carotene						
Median intake, IU	152.5	303.3	499.8	1085	1830	...
Adjusted OR†	1.0	0.94	0.67	0.64	0.69	.03
Multivariate OR (95% CI)‡	1.0	1.01 (0.6-1.6)	0.73 (0.5-1.2)	0.73 (0.4-1.2)	0.79 (0.5-1.3)	.14
Beta carotene						
Median intake, IU	1143	1997	3094	4728	8053	...
Adjusted OR†	1.0	0.79	0.78	0.65	0.51	.004
Multivariate OR (95% CI)‡	1.0	0.86 (0.5-1.4)	0.86 (0.5-1.4)	0.72 (0.4-1.1)	0.59 (0.4-0.96)	.03
Beta cryptoxanthin						
Median intake, IU	17.44	51.12	76.37	118.58	224	...
Adjusted OR†	1.0	0.71	0.93	0.74	0.65	.12
Multivariate OR (95% CI)‡	1.0	0.82 (0.5-1.3)	1.12 (0.7-1.8)	0.93 (0.6-1.5)	0.89 (0.5-1.4)	.84
Lycopene						
Median intake, IU	217.7	509.4	1367	1837	3450	...
Adjusted OR†	1.0	0.94	0.61	0.64	1.06	.71
Multivariate OR (95% CI)‡	1.0	1.0 (0.6-1.6)	0.70 (0.4-1.1)	0.70 (0.4-1.1)	1.16 (0.7-1.8)	.96
Lutein/zeaxanthin						
Median intake, IU	560.8	1211	1708	2487	5757	...
Adjusted OR†	1.0	1.14	0.82	0.74	0.40	<.001
Multivariate OR (95% CI)‡	1.0	1.14 (0.7-1.8)	0.84 (0.5-1.3)	0.77 (0.5-1.2)	0.43 (0.2-0.7)	<.001
**Two-Nutrient Multivariate Model**						
Beta carotene, OR (95% CI)	1.0	0.89 (0.5-1.4)	1.03 (0.6-1.7)	0.97 (0.6-1.6)	0.91 (0.5-1.6)	.80
Lutein/zeaxanthin, OR (95% CI)	1.0	1.16 (0.7-1.8)	0.85 (0.5-1.4)	0.77 (0.5-1.3)	0.44 (0.2-0.8)	.008

*OR indicates odds ratio; CI, confidence interval; and ellipses, not applicable.
†Adjusted for age, sex, and clinic.
‡Included terms for age (continuous), sex, clinic (Massachusetts, New York, Illinois, Wisconsin, Maryland), education (>, <, or=12th grade), systolic blood pressure (mm Hg), self-reported physical activity level (average, below average, above average), alcohol intake (grams per day), body mass index, and smoking status (never smoker, former smoker, current smoker).

Table 3.—Odds Ratios for Exudative Age-Related Macular Degeneration According to Use of Vitamin Supplementation*

Supplements	Never Taken	Taken in Past Only	Currently Taking 0-1 y	Currently Taking 2+ y	*P* (Trend)
Vitamin C					
Cases/Controls, No.	192/288	65/95	20/10	59/96	...
Adjusted OR (95% CI)†	1.00	1.05 (0.71-1.55)	2.29 (1.02-5.1)	0.74 (0.5-1.1)	.36
Multivariate OR (95% CI)‡	1.00	1.14 (0.76-1.71)	2.53 (1.1-5.8)	0.87 (0.6-1.3)	.87
Vitamin E					
Cases/Controls, No.	226/344	40/66	30/10	40/69	...
Adjusted OR (95% CI)†	1.00	1.01 (0.64-1.59)	4.29 (1.96-9.4)	0.83 (0.5-1.3)	.73
Multivariate OR (95% CI)‡	1.00	1.08 (0.68-1.74)	5.06 (2.2-11.5)	0.97 (0.6-1.5)	.28
Vitamin A§					
Cases/Controls, No.	284/415	23/38	29/28		...
Adjusted OR (95% CI)†	1.00	0.92 (0.52-1.63)	1.38 (0.78-2.42)		.38
Multivariate OR (95% CI)‡	1.00	0.96 (0.53-1.72)	1.53 (0.86-2.75)		.22
Multivitamins§					
Cases/Controls, No.	141/188	89/147	115/167		...
Adjusted OR (95% CI)†	1.00	0.75 (0.52-1.08)	0.78 (0.55-1.10)		.14
Multivariate OR (95% CI)‡	1.00	0.77 (0.53-1.12)	0.82 (0.57-1.18)		.27

*Number of cases and controls varies because of missing information. OR indicates odds ratio; CI, confidence interval; and ellipses, not applicable.
†Adjusted for age, sex, and clinic.
‡Included terms for age, sex, clinic, education, systolic blood pressure, self-reported physical activity level, alcohol intake, body mass index, and smoking status.
§Duration of supplementation for current multivitamin users not available and number of current vitamin A users too few to categorize by duration of use.

for AMD and carotenoid intake, we also evaluated individual foods containing carotenoids (Table 4). A strong, inverse association was seen for dietary intake of spinach or collard greens, with a statistically significant trend for a lower risk for AMD with a greater frequency of intake of these vegetables (*P*<.001). Compared with those consuming these greens less than once per month in the multivariate model, the OR for those eating them two to four times per week was 0.54 (95% CI, 0.3 to 0.9); for those eating the greens five or more times per week, the OR was 0.14 (95% CI, 0.01 to 1.2). The intake of other carotenoid-rich foods also tended to be inversely associated with risk for AMD, but these associations were not significant in the multivariate models, and the magnitude of the apparent effect was not as large for these foods.

Because of the strong and clear relationship between spinach and reduced risk for AMD and the likelihood of a correlation between spinach and the other foods, we conducted analyses including spinach plus each one of the other carotenoid-rich foods (two food models). The intake of spinach remained highly significant, but the associations between the consumption of all the other foods and the risk for AMD weakened. A formal test of the difference in slopes between frequency of intake of spinach vs carrots approached statistical significance (*P*=.07). Further, we conducted

Downloaded From: https://jamanetwork.com/ by a SCELC - University of Southern California User on 07/17/2023

Table 4.—Odds Ratios for Exudative Age-Related Macular Degeneration by Frequency of Consumption of Foods Rich in Carotenoids*

Food Item (Serving Size)	Frequency (No. of Servings per Time Period) <1/mo	1-3/mo	1/wk	2-4/wk	5-6+/wk	P (Trend)
Broccoli (0.5 cup)						
Cases/Controls, No.	64/81	96/144	109/152	76/120	8/20	...
Adjusted OR (95% CI)†	1.0	0.82 (0.5-1.3)	0.87 (0.6-1.3)	0.66 (0.4-1.1)	0.47 (0.2-1.2)	.06
Multivariate OR (95% CI)‡	1.0	0.92 (0.6-1.5)	1.06 (0.7-1.7)	0.85 (0.5-1.4)	0.50 (0.2-1.3)	.34
Cabbage, cauliflower, brussels sprouts (0.5 cup)						
Cases/Controls, No.	92/112	115/191	91/130	49/72	7/11	...
Adjusted OR (95% CI)†	1.0	0.82 (0.6-1.2)	0.88 (0.6-1.3)	0.90 (0.6-1.5)	0.63 (0.2-1.8)	.57
Multivariate OR (95% CI)‡	1.0	0.82 (0.5-1.2)	0.90 (0.6-1.4)	1.04 (0.6-1.7)	0.65 (0.2-1.8)	.91
Carrots (whole or cooked, 0.5 cup)						
Cases/Controls, No.	32/48	96/126	106/150	84/142	37/53	...
Adjusted OR (95% CI)†	1.0	1.1 (0.6-1.9)	0.87 (0.5-1.5)	0.74 (0.4-1.3)	0.72 (0.4-1.4)	.06
Multivariate OR (95% CI)‡	1.0	1.21 (0.7-2.1)	1.0 (0.6-1.8)	0.88 (0.5-1.6)	0.89 (0.4-1.8)	.23
Spinach or collard greens (cooked or raw, 0.5 cup)						
Cases/Controls, No.	141/158	118/179	70/110	23/58	1/11	...
Adjusted OR (95% CI)†	1.0	0.81 (0.6-1.1)	0.63 (0.4-0.9)	0.52 (0.3-0.9)	0.12 (0.01-0.09)	<.001
Multivariate OR (95% CI)‡	1.0	0.81 (0.6-1.2)	0.61 (0.4-0.9)	0.54 (0.3-0.9)	0.14 (0.01-1.2)	<.001
Sweet potato (0.5 cup)§						
Cases/Controls, No.	191/276	115/174	32/42	15/20		...
Adjusted, OR (95% CI)	1.0	0.84 (0.6-1.2)	0.84 (0.5-1.4)	0.67 (0.3-1.4)		.18
Multivariate, OR (95% CI)	1.0	0.85 (0.6-1.2)	0.85 (0.5-1.5)	0.64 (0.3-1.4)		.19
Winter squash (0.5 cup)§						
Cases/Controls, No.	221/278	85/158	36/54	12/27		...
Adjusted OR (95% CI)†	1.0	0.69 (0.5-1.0)	0.75 (0.5-1.2)	0.56 (0.3-1.2)		.03
Multivariate OR (95% CI)‡	1.0	0.74 (0.5-1.1)	0.79 (0.5-1.3)	0.60 (0.3-1.3)		.08

*OR indicates odds ratio; CI, confidence interval; and ellipses, not applicable.
†Adjusted for age, sex, and clinic. OR indicates odds ratio; CI, confidence interval.
‡Included terms for age, sex, clinic, education, systolic blood pressure, self-reported physical activity level, alcohol intake, body mass index, and smoking status.
§Highest two categories combined because of five or fewer controls.

an additional analysis in which carotenoid intake was also included in the same models. The association between spinach intake and AMD remained significant (*P* for trend=.007). The effect of overall carotenoid intake was reduced and not significant in the model with spinach, although it maintained an inverse association with risk for AMD.

Effect Modification by Smoking

The inverse trend associated with lutein/zeaxanthin intake (lower risk for AMD with higher intake) was seen for all categories of smoking (Figure) and was somewhat stronger in current smokers. In the highest quintile of intake, there was little apparent effect of cigarette smoking. In a separate analysis (data available on request from authors), we found that persons in the high-risk group (low quintile of intake, current smoker) had almost a sixfold elevated risk for AMD compared with persons in the low-risk group (high quintile of intake, nonsmoker) (OR, 5.89; 95% CI, 2.3 to 15.2). For vitamins C and E, a protective association for higher intake was suggested only among those who had never smoked (data available on request from authors). However, these associations were not statistically significant. Interaction terms for specific nutrients and smoking, when added to multivariate models, were also not statistically significant.

COMMENT

Findings from this multicenter study support the possibility that an increased intake of dietary antioxidants, specifically carotenoids, may reduce the risk for advanced AMD, the form of the disease associated with the most severe visual loss.

After adjusting for other risk factors, individuals consuming the highest levels of carotenoids had a statistically significant 43% lower risk for AMD compared with those who consumed the lowest levels. A significant trend was seen for a lower risk for AMD with increasing amounts of carotenoids in the diet. Of all the carotenoid fractions evaluated, the combination of lutein and zeaxanthin was most strongly associated with AMD.

The apparent beneficial effect was strongest for the intake of spinach or collard greens among all the carotenoid-rich foods consumed. These greens maintained the same magnitude of association with AMD risk in analytic models that included the consumption of other carotenoid-rich foods as well as in analyses that also included carotenoid intake. Spinach and collard greens contain larger amounts of lutein and zeaxanthin than the other carotenoid-rich foods, whereas of foods consumed in the United States, carrots are the most important source of carotenoids with vitamin A activity as well as beta carotene, but are very low in lutein and zeaxanthin.[21] Therefore, the association between carotenoid intake and lower risk for AMD appears to be accounted for primarily by lutein/zeaxanthin rather than carotenoids with provitamin A activity. Although not specifically included in our diet questionnaire, kale, mustard greens, and turnip greens also contain substantial amounts of lutein and zeaxanthin.

Findings regarding vitamin intake other than carotenoids were less consistent. There was a suggestive trend for an inverse association between vitamin A intake and AMD risk, but this association appeared to be entirely due to the carotenoid component. A beneficial effect for high intake of vitamin C, particularly from foods, cannot be ruled out. However, data do not support the supposition that vitamin E offers a protective effect, as the overall results were in the opposite direction. When we evaluated the effect of total vitamin E after controlling for the dietary intake of polyunsaturated fatty acids or linoleic acid, which are found in vitamin E–rich foods like vegetable oils, and carotenoid intake, we found that the ORs were not appreciably changed. Given the lack of an association of AMD risk with high-dose vitamin E with vitamin supplements and the lack of a significant dose relationship, it is unlikely that the sug-

Downloaded From: https://jamanetwork.com/ by a SCELC - University of Southern California User on 07/17/2023

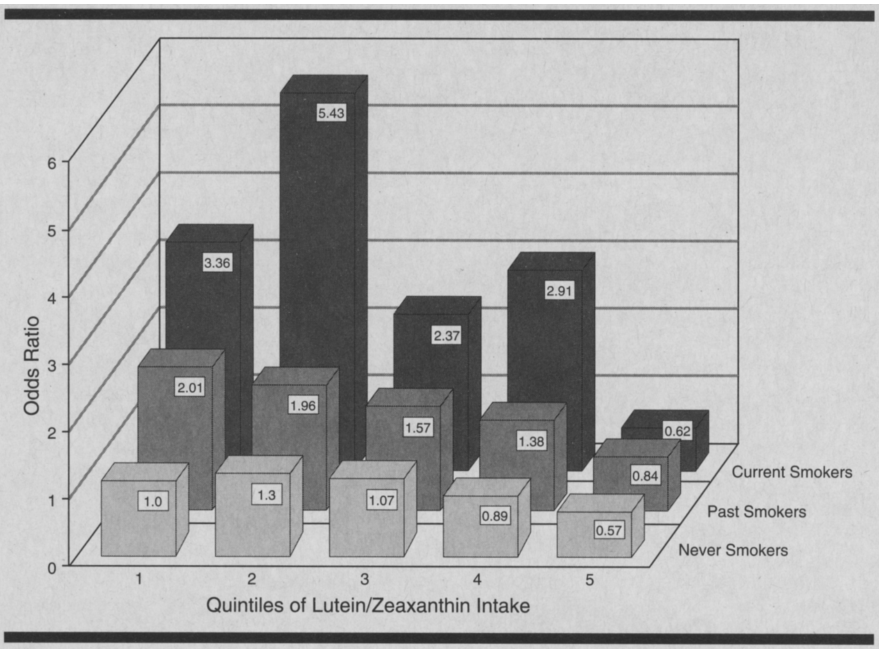

Odds ratio for age-related macular degeneration according to quintile of lutein/zeaxanthin intake and smoking status, adjusted for age, sex, clinic, education, systolic blood pressure, self-reported physical activity, alcohol intake, and body mass index. The referent group consisted of never smokers in the lowest quintile of lutein/zeaxanthin intake.

gestion of a positive association with dietary vitamin E without supplements represents a true or causal association.

Supplementation with vitamin and mineral pills is an increasingly important health topic. Overall, these data do not support a large beneficial effect of consumption of vitamin supplements on the risk for AMD. The adverse effects noted for short-term users of vitamins E and C, in contrast to long-term users, were likely related to the recent diagnosis of AMD that may have led to use of supplements. If duration of intake was known for multivitamins, a stronger association may have been seen for their long-term use as well. In addition, the statistical power to detect an association between individual supplements and AMD was limited because of the smaller number of subjects in these subgroups.

Current smokers had the highest risk for AMD compared with past smokers and nonsmokers, although the excess risk associated with smoking appeared to be reduced among persons with greater intake of lutein and zeaxanthin, particularly with the highest level of intake. However, these subgroup findings are speculative and require further study.

One must consider the possibility of confounding in this observational study. People who consume diets that are rich in certain nutrients may also differ with regard to other health habits or characteristics that are related to AMD risk. It is plausible that the intake of antioxidant-rich foods is indeed protective, but the benefit may result not from their antioxidant properties but rather from some other nutrient that these foods have in common. If this were the case, vitamin supplements would not be helpful. Adjustment in this study for a range of potential risk factors had only a modest effect on the ORs comparing the highest to the lowest quintile of carotenoid intake (from 0.49 to 0.57) (Table 1). Some unmeasured and, therefore, uncontrolled factors might still be confounding this relationship, but they would have to be associated highly with carotenoid intake and a strong risk factor for AMD to explain these results.

The quality of information on exposure and disease in this study is high. The reproducibility and validity of the FFQ has been demonstrated in previous studies.[19-20] Subjects were all examined by retina specialists, and the diagnosis of AMD was confirmed by retinal angiography. Therefore, classification according to the presence or absence of advanced AMD was highly accurate.

The potential for biased selection cannot be ruled out entirely, but it was minimized by the manner in which case and control subjects were selected and the relatively high and equally distributed participation rates. Eighteen percent of case subjects and 22% of control subjects did not participate in the overall study, and reasons for nonparticipation were similarly distributed among cases and control subjects. Eighty-five percent of case subjects and 85% of control subjects were included in the dietary analyses, and there were no significant differences between these participants and nonparticipants with regard to age, sex, or education.

Although case subjects may have selectively recalled worse dietary habits than control subjects, there is no reason to believe that such differential recall would affect some micronutrients, such as carotenoids, more than the others evaluated in this report. We selected case subjects who were diagnosed with AMD no longer than 1 year before their enrollment into this study to decrease the possibility that the diagnosis of eye disease would influence the reporting of a person's behaviors. However, we did find that case subjects were more likely than controls to take supplements within the past year (Table 3). If similar changes occurred in the diet and were reported on the FFQ, then our finding of an inverse association between carotenoid intake and AMD would be an underestimate of the beneficial effect.

These findings regarding dietary intake of micronutrients are generally consistent with a previous report on this study population regarding the blood values of these nutrients.[10] Dietary and blood carotenoid levels had the strongest association with AMD risk in each report (ORs, 0.57 and 0.30 for extreme categories, respectively), which strengthen and confirm our findings. In the previous study, all serum carotenoids except lycopene were significantly inversely associated with risk for AMD, whereas our dietary results suggest that lutein/zeaxanthin are the important carotenoids. Findings regarding vitamin C assessed by dietary and blood levels were also similar (ie, they were suggestive but nonsignificant). For vitamin E, the point estimates were in opposite directions in the two studies, but tests for trend were not statistically significant in either one.

Other than the previous biochemical study,[10] there are few published epidemiologic data with which to compare our findings and none that specifically examine dietary carotenoids. All types of AMD were pooled in a cross-sectional study based on the National Health and Nutition Examination Survey.[9] Researchers found that people with low intake of vitamin A–rich fruits and vegetables had a significantly higher risk for AMD compared with those whose consumption was high. Many of these food items are also rich in carotenoids, but carotenoid intake was not specifically addressed in that study. Plasma levels of vitamins and use of supplements were assessed in another cross-sectional study, although dietary intake was not evaluated.[12] Plasma vitamin E level was inversely associated with the nonadvanced form of AMD, but vitamin supplementation was not. The very few

Downloaded From: https://jamanetwork.com/ by a SCELC - University of Southern California User on 07/17/2023

cases of advanced AMD (n=11) preclude any firm conclusions about that group. In a small case-control study, researchers found no association between levels of serum vitamins A, C, or E and AMD.[11] However, the power of that study to detect an association was low, given that there were only 26 case subjects, 23 control subjects, and the control group consisted of spouses of case subjects.

The theory that nutrition may play a role in the mechanism of development of AMD is biologically plausible. Various insults including normal metabolic processes within cells and possibly photochemical damage from ultraviolet and high-energy visible light can initiate the oxidation process in the eye. These stimuli lead to formation of free radicals and highly reactive singlet oxygen that can initiate lipid peroxidation.[23] Conditions in the outer retina are optimal for these effects because of the high concentrations of polyunsaturated fatty acids in the photoreceptor outer-segment membranes.[8] These insults may lead to damage, incompletely degraded molecules, and impaired function of the retinal pigment epithelium and, theoretically, could lead to degeneration involving the macula. Nutrients with antioxidant properties might help defend the retina against these reactions and might also maintain the normal function of blood vessels that supply the macular region. This latter mechanism would invoke a vascular etiology for AMD, a hypothesis that has been suggested for the advanced form.[18] The antioxidant activity of carotenoids is well established[24]; carotenoids could also serve that function in the retina.[25] On the other hand, the association between carotenoid intake and AMD might not be related to an antioxidant mechanism, given that foods containing vitamins E and C (which have antioxidant potential) were not significantly associated with AMD.

The dominant pigments in the macula are lutein and zeaxanthin, which are selectively accumulated in the retina from plasma.[25,26] Beta carotene and lycopene are virtually absent in the macula.[25] These yellow pigments, lutein and zeaxanthin, can filter out visible blue light, which theoretically can cause photic damage.[25] Ultraviolet light is filtered by the cornea and lens in the anterior aspect of the eye, but visible blue light reaches the retina. Therefore, lutein and zeaxanthin might serve to protect the retina from photic damage or other oxidative insults.

In conclusion, our data are consistent with the hypothesis that increasing dietary intake of foods that are rich in antioxidants, particularly certain carotenoids, may reduce the risk of developing advanced AMD. A significant trend was seen for a decreased risk for AMD among people with greater intake of carotenoids and carotenoid-rich foods, particularly spinach and collard greens. Use of vitamin supplements was not significantly associated with AMD. Although these observational nutritional data do not establish causality,[27] it seems prudent to concur with the recommendation of increasing the consumption of vegetables in the diet and, in particular, to include dark green, leafy vegetables that are rich in lutein and zeaxanthin.

The fastest growing segment of the US population comprises persons older than 65 years, and the prevalence and impact of AMD will continue to increase. Identifying strategies to prevent or retard the onset of AMD will have a major impact on the burden of blindness and visual impairment among the elderly. Prospective observational studies and randomized clinical trials will help clarify the relationship between these potentially important nutritional factors and the incidence of AMD.

This study was supported in part by grant EY09972 from the National Institutes of Health, the Macula Foundation Inc, and Hoffman-LaRoche Inc.

References

1. National Advisory Eye Council, Report of the Retinal and Choroidal Diseases Panel. *Vision Research—A National Plan: 1983-1987.* Bethesda, Md: US Dept of Health and Human Services; 1984. National Institutes of Health publication 83-2471.
2. Klein R, Klein B, Linton KLP. Prevalence of age-related maculopathy: the Beaver Dam Study. *Ophthalmology.* 1992;99:933-943.
3. Organisciak DT, Wang HM, Li Z, Li ZY, Tso MOM. The protective effect of ascorbate in retinal light damage of rats. *Invest Ophthalmol Vis Sci.* 1985;26:1580-1588.
4. Tso MOM, Woodford BJ, Lam KW. Distribution of ascorbate in normal primate retina and after photic injury: a biochemical, morphological correlated study. *Curr Eye Res.* 1984;3:181-191.
5. Ham WT, Mueller HA, Ruffolo JJ, et al. Basic mechanisms underlying the production of photochemical lesions in the mammalian retina. *Curr Eye Res.* 1984;3:165-174.
6. Katz ML, Parker KR, Handelman GJ, Bramel TL, Dratz EA. Effects of antioxidant nutrient deficiency on the retina and retinal pigment epithelium of albino rats: a light and electron microscopic study. *Exp Eye Res.* 1982;34:339-369.
7. Hayes KC. Retinal degeneration in monkeys induced by deficiencies of vitamin E or A. *Invest Ophthalmol Vis Sci.* 1974;13:499-510.
8. Young RW. Solar radiation and age-related macular degeneration. *Surv Ophthalmol.* 1988;32:252-269.
9. Goldberg J, Flowerdew G, Smith E, Brody JA, Tso MOM. Factors associated with age-related macular degeneration: an analysis of data from the First National Health and Nutrition Examination Survey. *Am J Epidemiol.* 1988;128:700-710.
10. The Eye Disease Case-Control Study Group. Antioxidant status and neovascular age-related macular degeneration. *Arch Ophthalmol.* 1993;111:104-109.
11. Blumenkranz MS, Russell SR, Robey MG, Blumenkranz RK, Penneys N. Risk factors in age-related maculopathy complicated by chroroidal neovascularization. *Ophthalmology.* 1986;96:552-558.
12. West S, Vitale S, Hallfrisch J, et al. Are antioxidants or supplements protective of age-related macular degeneration? *Arch Ophthalmol.* 1994;112: 222-227.
13. Newsome DA, Swartz M, Leone NC, Elston RC, Miller E. Oral zinc in macular degeneration. *Arch Ophthalmol.* 1988;106:192-198.
14. Willett W. *Nutritional Epidemiology.* New York, NY: Oxford University Press; 1990.
15. Blackman BC, White P, Tsou W, Finkel D. Peroxidation of plasma and platelet lipids in chronic smokers and insulin-dependent diabetics. *Ann N Y Acad Sci.* 1984;435:385-387.
16. Chow CK, Thacker RR, Changchit C, et al. Lower levels of vitamin C and carotenes in plasma of cigarette smokers. *J Am Coll Nutr.* 1986;5:305-312.
17. Stryker WS, Kaplan LA, Stein EA, Stampfer MJ, Sober A, Willett WC. The relation of diet, cigarette smoking, and alcohol consumption to plasma beta carotene and alpha tocopherol levels. *Am J Epidemiol.* 1988;127:283-291.
18. The Eye Disease Case-Control Study Group. Risk factors for neovascular age-related macular degeneration. *Arch Ophthalmol.* 1992;110:1701-1708.
19. Willett WC, Sampson L, Stampfer MJ, et al. Reproducibility and validity of a semiquantitative food frequency questionnaire. *Am J Epidemiol.* 1985;122:51-65.
20. Ajani U, Willett W, Seddon J, the Eye Disease Case-Control Study Group. Reproducibility of a food frequency questionnaire for use in ocular research. *Invest Ophthalmol Vis Sci.* 1994;35:2725-2733.
21. Mangels AR, Holden JM, Beecher GR, Forman MR, Lanza E. Carotenoid content of fruits and vegetables: an evaluation of analytic data. *J Am Diet Assoc.* 1993;93:284-296.
22. Willett WC, Stampfer MJ. Total energy intake: implications for epidemiologic analyses. *Am J Epidemiol.* 1986;124:17-27.
23. Foote CS. Photosensitized oxidation and singlet oxygen: consequences in biological systems. In: Pryor WA, ed. *Free Radicals in Biology.* New York, NY: Academic Press; 1976;2:85-133.
24. Krinsky NI. Antioxidant function of carotenoids. *Free Radic Biol Med.* 1989;7:617-635.
25. Schalch W. Carotenoids in the retina: a review of their possible role in preventing or limiting damage caused by light and oxygen. In: Emerit I, Chance B, eds. *Free Radicals and Aging.* Basel, Switzerland: Birkhauser Verlag; 1992:280-298.
26. Handleman GJ, Snodderly DM, Adler AJ, Russett MD, Oratz EA. Measurements of carotenoids in human and monkey retinas. In: Packer L, ed. *Methods in Epidemiology.* San Diego, Calif: Academic Press; 1992:220-230.
27. Seddon JM, Hennekens CH. Vitamins, minerals, and macular degeneration: promising but unproven hypotheses. *Arch Ophthalmol.* 1994;112:176-179.

Downloaded From: https://jamanetwork.com/ by a SCELC - University of Southern California User on 07/17/2023
