# Supplementary material for: Insights into the nutritional prevention of macular degeneration based on a comparative topic modeling approach
Source: PeerJ Comput Sci. 2024 Mar 20;10:e1940. doi: 10.7717/peerj-cs.1940 (PMC11042009; doi:10.7717/peerj-cs.1940)
Supplement: Supplemental Information 2 [file peerj-cs-10-1940-s002.zip › positive_result_reports/new3.pdf]

Check for updates

Original research article

EJO European Journal of Ophthalmology

European Journal of Ophthalmology
2020, Vol. 30(2) 376–381
© The Author(s) 2019
Article reuse guidelines:
sagepub.com/journals-permissions
DOI: 10.1177/1120672119836007
journals.sagepub.com/home/ejo
SAGE

Effect of 2-year nutritional supplementation on progression of age-related macular degeneration

Alberto Piatti[1], Antonella Croce[2], Danilo Mazzacane[3], Giovanni Traina[4], Lina Ambrosino[5], Luigi Boni[6], Luca Lisi[7], Maria Caterina Cascella[8] and Alberto Grunberger[9]

Abstract

Purpose: To examine the effect of a long-term nutritional supplementation on age-related macular degeneration progression.

Methods: In this prospective, double-blind, placebo-controlled study, 80 patients with intermediate age-related macular degeneration were randomized (2:1) to receive 1 tablet/day of a nutritional supplement containing a mixture of carotenoids, vitamins and omega-3 fatty acids or placebo. Age-related macular degeneration progression assessed by digital fundus photography (primary outcome) and best-corrected visual acuity were evaluated. Differences between arms were tested using chi-square test or Fisher's exact test.

Results: Seventy-four patients completed the follow-up at 24 months (48 in the treated arm and 26 in the placebo arm). An age-related macular degeneration progression was observed in the 2.1% of patients of the treated arm and in the 15.4% of patients in the placebo arm ($p = 0.05$, Fisher's exact test). Best-corrected visual acuity data alone were not statistically significant among groups.

Conclusion: A clinically meaningful stabilization of intermediate age-related macular degeneration over a period of 2 years may be obtained by treating patients with a mixture of carotenoids, vitamins and omega-3 fatty acids.

Keywords

Carotenoids, omega-3 fatty acids, macular degeneration

Date received: 12 May 2018; accepted: 15 February 2019

Introduction

Age-related macular degeneration (AMD) is the leading cause of visual loss in elderly. It can be distinguished in two forms: the early AMD, characterized by drusen and pigmentary changes, and the late AMD, including geographic atrophy and choroidal neovascularization (CNV). The prevalence of both early and late AMD remained stable during the last decade, affecting 13.2% and 3.0%, respectively, in the age category >70 years.[1] AMD is multifactorial disease involving several factors, including familiarity, age and lifestyle.[2] Medical treatment is limited to the neovascular form of AMD. The treatment of dry AMD represents a major challenge as there is no approved therapy available for these patients,[3] for which the only way to delay its progression is a lifestyle change and the use of nutritional supplements with high anti-oxidant activity.[4,5] The age-related eye disease study (AREDS) is the most important large-scale interventional study conducted in AMD patients. This study demonstrated that in

[1]ASL TO5, Moncalieri, Italy
[2]ASL Città di Torino, Torino, Italy
[3]ASST Pavia and Melegnano, Pavia, Italy
[4]ASL Toscana Nord Ovest, Pisa, Italy
[5]ASL Napoli 1 Centro, Napoli, Italy
[6]ASL Toscana Sud Est, Arezzo, Italy
[7]ASL Roma 2, Rome, Italy
[8]ASL di Bari, presidio F. Iaia, Bari, Italy
[9]ATS Sardegna, SASSL Nuoro, Italy

Corresponding author:
Alberto Piatti, ASL TO5 di Moncalieri, Via Vittime di Bologna 20, 10024 Moncalieri (TO), Italy.
Email: piatti.alberto@aslto5.piemonte.it

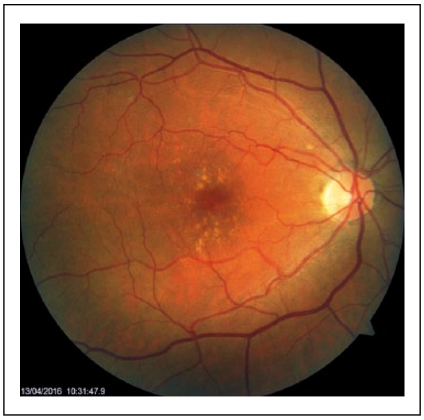

Figure 1. Ocular fundus photography of intermediate AMD.

patients with intermediate or advanced AMD, a combination of vitamins (C + E), zinc and beta carotene can reduce significantly the risk of progression.[5,6] The following AREDS2 study demonstrated that lutein and zeaxanthin could be more appropriate than β-carotene in the nutritional supplementation of such patients without influencing the beneficial effect of AMD progression.[7] The same study showed no additional effects of omega-3 fatty acids (docosahexaenoic acid (DHA)/eicosapentaenoic acid (EPA))[7] and similar findings have been also observed in the other prospective interventional studies NAT-2.[8] These results did not support the prevailing view based on several lines of evidence from laboratory and epidemiological studies that suggested omega-3 fatty acid intake might have a protective role in AMD progression.[9] A possible explanation for such discrepancy is that the study design may not have permitted the prophylactic potential of omega-3 to be demonstrated.[9] Therefore, the role of omega-3 fatty acids in the nutritional supplementation of dry AMD is still object of debate and additional clinical data are needed. In this study, we report the results of a 2-year, placebo-controlled, intervention study aiming to analyse the effect of a commercially available combination of carotenoids, vitamins and omega-3 fatty acids on the progression of intermediate AMD according to AREDS Classification.

Materials and methods

This was a multicenter, double-masked, placebo-controlled, prospective clinical study performed by the GOAL group (Gruppo Oculisti Ambulatoriali Liberi – Scientific Association of Italian Ophthalmologists operating in Eye Primary Care) involving eight centres (see Appendix 1). Main inclusion criteria for patients were as follows: (1) age of 55–80 years; (2) diagnosis of intermediate AMD, according to the AREDS Research Group classification;[10] (3) presence of medium (⩾63 μm, <125 μm) and/or large drusens (⩾125 μm) and/or small areas of non-central retinal atrophy in both eyes (Figure 1); (4) best-corrected visual acuity (BCVA) for distance ⩾20/32 Snellen decimal (LogMAR 0.2) and a minimum number of 43 letters read at the ETDRS (Early Treatment Diabetic Retinopathy Study) chart; (5) BCVA for near ⩾20/32 Snellen decimal (LogMAR 0.2) at the MNREAD chart. MNREAD Acuity Charts were developed at the Minnesota Laboratory for Low-Vision Research, University of Minnesota and the print size steps between successive sentences follow a logarithmic progression.

In case of bilateral AMD, eye with the best visual acuity (VA) was selected for the study; if both eyes had the same VA, the right eye was chosen. The fellow eye was always monitored in case of bilateral AMD. Main exclusion criteria were as follows: (1) presence myopia >3 dioptres, (2) presence of any other disorder of the macula and (3) history of eye surgery in the 3 months prior to enrolment in the study. Written informed consent was obtained for all patients. The research followed the tenets of the Declaration of Helsinki and was approved by local Ethics Committees.

Eligible patients were randomized (2:1 ratio) to receive (1 tablet/daily) for 24 months either a food supplement (Azyr Mega, SIFI S.p.A, Italy) containing a mixture of carotenoids (lutein 10 mg, astaxanthin 4 mg, zeaxanthin 2 mg) antioxidants (vitamin C 90 mg, vitamin E 30 mg, zinc 22.5 mg plus copper 1 mg) and omega-3 fatty acids (fish oil 500 mg, containing EPA 185 mg and DHA 140 mg) or placebo. Food supplement and placebo were packaged in identical containers and indistinguishable in terms of external appearance.

Study visits were conducted at baseline and after 6–12–18–24 months. Patients received a complete ophthalmic examination including mydriatic retinography (performed by digital fundus camera), BCVA for distance measured by ETDRS Chart, BCVA for near measured by MN Read Chart.

The primary outcome of the study was the evaluation of AMD progression by retinography. Retinography was coded as worsened when increased atrophic areas not necessarily in the centre of the macula or when showed macular haemorrhages suggesting neovascular AMD; stable (no change) or improved (reduction of number of drusen or atrophic areas). In addition, fluorescein and indocyanine green angiography were always done in case of new macular haemorrhages, to confirm the diagnosis of neovascular AMD. An independent reading centre judged all the photographs taken during the clinical trial.

VA for distance was coded as worsened (loss ⩾ 5 ETDRS letters), stable (no change) or improved (gain ⩾ 5 ETDRS letters); VA for near was coded as worsened (loss > 1 sentence), stable (no change) or improved (gain > 1 sentence).

The statistical analysis was performed by SPARC Consulting S.r.l. (Milan, Italy). Continuous variables were

Table 1. Demographic and baseline characteristics (full analysis subset).

	Overall (N = 74)	Food supplement (N = 48)	Placebo (N = 26)
Age, years			
Mean ± SD	71.9 ± 6.2	71.4 ± 6.5	72.7 ± 5.5
Median	73	72.5	73
Minimum–maximum	55–82	55–82	64–80
Gender, n (%)			
Male	23 (31.8)	17 (35.4)	6 (23.1)
Female	51 (68.9)	31 (64.6)	20 (76.9)
Drusen type, (%)			
Hard	33 (44.6)	22 (45.8)	11 (42.3)
Soft	41 (55.4)	26 (54.2)	15 (57.7)
Visual acuity (ETDRS letters)			
Mean ± SD	48.8 ± 4.4	49.4 ± 4	47.6 ± 4.4
Range	43–60	43–60	43–55
Median	49	49	46

SD: standard deviation; ETDRS: Early Treatment Diabetic Retinopathy Study.

Table 2. AMD progression.

Outcome	Food supplement (N = 48)	Placebo (N = 26)	p-value[a]
Retinography			
Worsened, n (%)	1 (2.1)	4 (15.4)	**0.05**
Stable or improved, n (%)	47 (97.9)	22 (84.6)	
Visual acuity (distance)			
Worsened, n (%)	7 (14.6)	5 (19.2)	0.74
Stable or improved, n (%)	41 (85.4)	21 (80.8)	
Visual acuity (near)			
Worsened, n (%)	8 (16.7)	9 (34.6)	0.08
Stable or improved, n (%)	40 (83.3)	17 (65.4)	
Combination of retinography, visual acuity for near and visual acuity for distance worsened			
Yes, n (%)	0	3 (11.5)	**0.04**
No, n (%)	48 (100)	23 (88.5)	

AMD: age-related macular degeneration.
[a]Fisher's exact p-value calculated only for the worsened group.

summarized by descriptive statistics (number of cases, mean, standard deviation (SD), median, minimum and maximum). Categorical variables were summarized using counts of patients and percentages. For categorical end-points, differences between treatment arms were tested using the chi-square test or Fisher's exact test, were applicable, at the 5% significance level. Relative risk (RR) and attributable risk (AR) were also calculated. All statistical tests were performed with a two-sided approach and were conducted at the $\alpha = 0.05$ significance level. Statistical analysis was performed using the SAS System version 9.4 under Windows 10 Pro.

Results

Eighty patients with intermediate AMD were screened and randomized. Only patients without major protocol violations were included in the statistical analysis (full analysis subset); this subset included 74 patients (48 treated with food supplement and 26 with placebo). Demographics and baseline characteristics of these 74 patients are displayed in Table 1. AMD progression was evaluated by retinography, VA for distance and for near and by a combination of all three parameters (Table 2). At the study end-point (24 months), only 5 out of 74 (6.8%) patients showed an AMD progression according to retinography (Table 2 and Figure 2). Three of these had an increase in atrophy whereas two patients developed CNV. Four out of these five patients were in the placebo arm (15.4%) and only one in the group treated with the food supplement (2.1%).

The comparison between the groups performed by means of Fisher's exact test showed a statistically significant difference ($p = 0.049$). A logistic regression analysis conducted to predict worsening of retinography using treatment group as predictor, demonstrated that this was a significant predictor ($p = 0.05$). The odds ratio for treatment

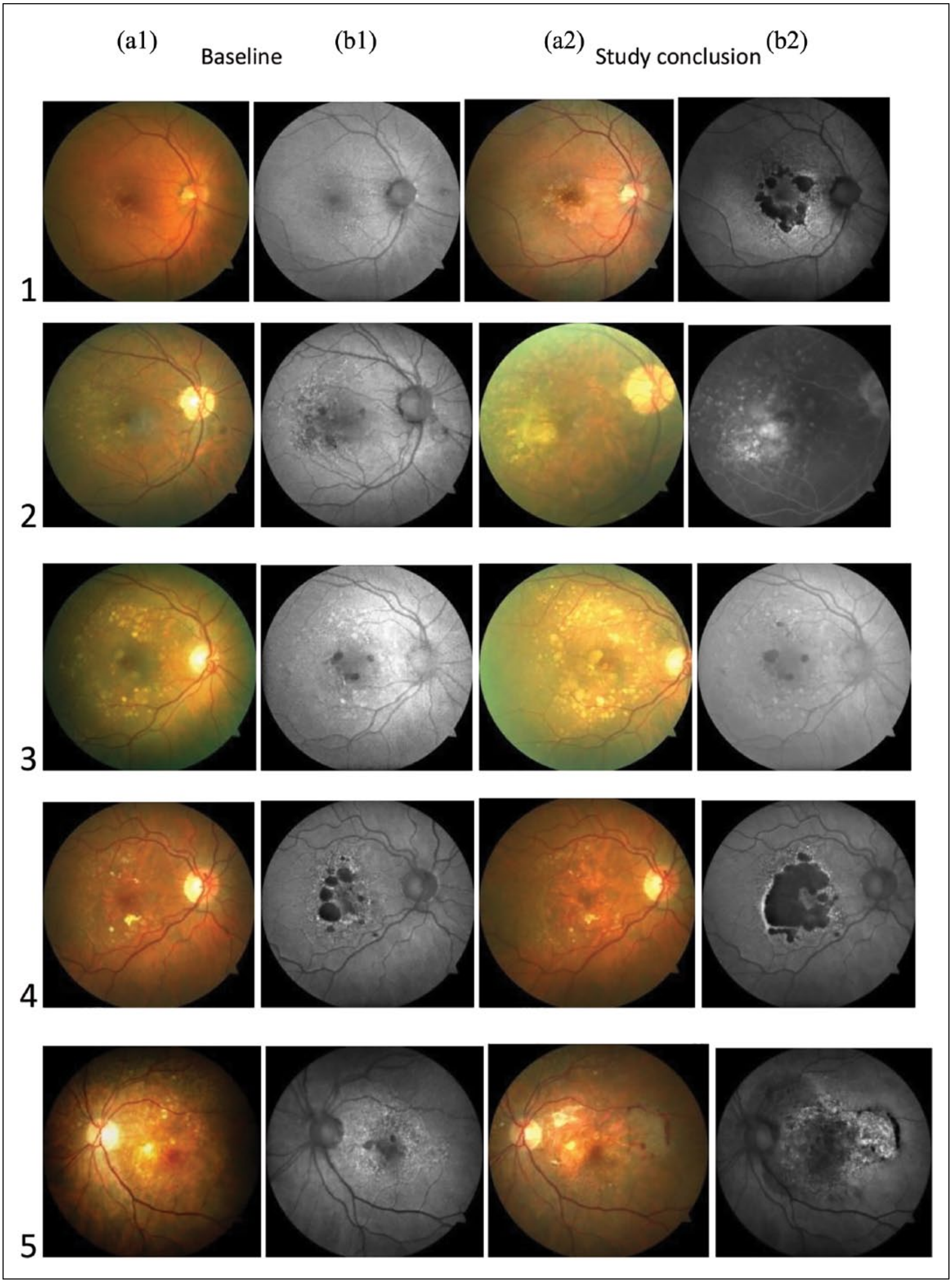

Figure 2. Colour photographs (a) and autofluorescence images (b) of the five patients with progressed retinography.
Baseline: a1–b1; study conclusion: a2–b2

group was 8.54 (95% confidence interval: 0.90–81.04), that is, patients in the placebo arm were nearly eight more times likely to have an AMD progression than patients in the food supplement arm. Moreover, VA (either at a distance or at near) was related to AMD progression only when analysed in combination with retinography (comparison between the two groups showed a statistically significant difference, $p = 0.04$, performed by means of Fisher's exact test) and not alone (Table 2). During the entire study, no significant adverse events were recorded.

Discussion

The high concentration of carotenoids in the macula generated the hypothesis that higher antioxidants intakes can prevent or delay the progression of age macular degeneration (AMD). Several epidemiological data provide the rationale for using antioxidants or other nutrients in treatment of AMD. The long-term, large population clinical trials of NEI (National Eye Institute) denominated AREDS I and II showed that in people with Intermediate AMD, the combination of vitamins C, vitamin E, carotenoids, zinc, copper and fatty acids omega-3 (AREDS II) reduced the risk over 5 years of severe visual loss by 25%.[7,10] In the Carotenoids in Age-Related Maculopathy Italian Study (CARMIS) Study,[11] an Italian multicenter prospective open-label randomized clinical trial, subjects receiving a supplementation of carotenoids (lutein, zeaxanthin, astaxanthin) did better in overall visual function (VA, contrast sensitivity, NEI Visual Functioning Questionnaire (VFQ)) than controls whose did not receive any supplementation.

The aim of GOALinAMD Study was to evaluate the effects of a long-term supplementation of a specific product containing a mixture of carotenoids, antioxidants and omega-3 fatty acids on intermediate AMD progression. For the first time, we evaluate the successful of the nutritional supplementation monitoring, as functional test, BCVA for near using a logarithmic chart, MN READ Test. While BCVA for distance depends on several factors as cataract or other diseases of anterior segment, BCVA for near is strictly connected with macular function and is a very strong predictive factor evaluating macular disease.

In GOALinAMD trial, subjects who received oral supplementation showed a reduction of risk of severe visual loss over 2 years of follow-up: no patients worsened in treatment group in all the three tests (BCVA for distance, for near and retinography) when were analysed together, whereas in the placebo arm three patients worsened in all three parameters. The comparison shows a statistically significant difference ($p = 0.04$) between two groups. Moreover, retinography alone showed that patients in the placebo arm were nearly eight more times likely to have an AMD progression after 24 months than patients in the food supplement arm. These results are according to AREDS data, even if daily oral supplementation was lower than AREDS. Lower daily dose has enabled the absence of significant adverse events.

There are some limitations to our study: the short follow-up and relatively small number of patients for the statistical analysis; of course, further studies are needed to evaluate the long-term effects of antioxidants on patients with intermediate AMD.

Declaration of conflicting interests

The author(s) declared following potential conflicts of interest with respect to the research, authorship, and/or publication of this article: A.P. received consulting fees from Allergan, Bayer, Novartis and SIFI. D.M. received consulting fees from SIFI. All other authors have nothing to disclose.

Ethical approval

Our study was approved by Ethics Committee of San Luigi Hospital (Turin, Italy) on 22 April 2013 and was conducted in accordance with the tenets of the Declaration of Helsinki on biomedical research involving human subjects.

Funding

The author(s) received no financial support for the research, authorship and/or publication of this article.

References

1. Colijn JM, Buitendijk GHS, Prokofyeva E, et al.; EYE-RISK Consortium; European Eye Epidemiology (E3) Consortium. Prevalence of age-related macular degeneration in Europe: the past and the future. *Ophthalmology* 2017; 124(12): 1753–1763.
2. Velez-Montoya R, Oliver SC, Olson JL, et al. Current knowledge and trends in age-related macular degeneration: genetics, epidemiology, and prevention. *Retina* 2014; 34(3): 423–441.
3. Holz FG, Schmitz-Valckenberg S and Fleckenstein M. Recent developments in the treatment of age-related macular degeneration. *J Clin Invest* 2014; 124(4): 1430–1438.
4. Sin HP, Liu DT and Lam DS. Lifestyle modification, nutritional and vitamins supplements for age-related macular degeneration. *Acta Ophthalmol* 2013; 91(1): 6–11.
5. Evans JR and Lawrenson JG. Antioxidant vitamin and mineral supplements for slowing the progression of age-related macular degeneration. *Cochrane Database Syst Rev* 2017; 7: CD000254.
6. Chew EY, Clemons TE, Agrón E, et al.; Age-Related Eye Disease Study Research Group. Long-term effects of vitamins C and E, β-carotene, and zinc on age-related macular degeneration: AREDS report no. 35. *Ophthalmology* 2013; 120(8): 1604–1611.
7. Age-Related Eye Disease Study 2 Research Group. Lutein + zeaxanthin and omega-3 fatty acids for age-related macular degeneration: the Age-Related Eye Disease Study 2 (AREDS2) randomized clinical trial. *JAMA* 2013; 309(19): 2005–2015.

8. Souied EH, Delcourt C, Querques G, et al.; Nutritional AMD Treatment 2 Study Group. Oral docosahexaenoic acid in the prevention of exudative age-related macular degeneration: the Nutritional AMD Treatment 2 study. *Ophthalmology* 2013; 120(8): 1619–1631.
9. Souied EH, Aslam T, Garcia-Layana A, et al. Omega-3 fatty acids and age-related macular degeneration. *Ophthalmic Res* 2015; 55(2): 62–69.
10. Age-Related Eye Disease Study Research Group. The Age-Related Eye Disease Study (AREDS): design implications. AREDS report no. 1. *Control Clin Trials* 1999; 20(6): 573–600.
11. Piermarocchi S, Saviano S, Parisi V, et al. Carotenoids in Age-Related Maculopathy Italian Study (CARMIS). Two years results of a randomized study. *Eur J Ophthalmol* 2012; 22(2): 216–225.

Appendix 1

The GOAL (Gruppo Oculisti Ambulatoriali) group: Alberto Piatti MD, ASL TO5 di Moncalieri; Antonella Croce MD, ASL Città di Torino; Giovanni Traina MD, ASL Toscana Nord Ovest; Lina Ambrosino MD, ASL Napoli 1 Centro; Luigi Boni MD, ASL Toscana Sud Est; Luca Lisi MD, ASL Roma 2; Maria Caterina Cascella MD, ASL di Bari, presidio F. Iaia; Alberto Grunberger MD, ATS Sardegna.
