# Supplementary material for: Insights into the nutritional prevention of macular degeneration based on a comparative topic modeling approach
Source: PeerJ Comput Sci. 2024 Mar 20;10:e1940. doi: 10.7717/peerj-cs.1940 (PMC11042009; doi:10.7717/peerj-cs.1940)
Supplement: Supplemental Information 2 [file peerj-cs-10-1940-s002.zip › positive_result_reports/new2.pdf]

AMERICAN ACADEMY OF OPHTHALMOLOGY®

Adherence to the Mediterranean Diet and Progression to Late Age-Related Macular Degeneration in the Age-Related Eye Disease Studies 1 and 2

*Tiarnán D. Keenan, BM BCh, PhD,[1,‡] Elvira Agrón, MA,[1,‡] Julie Mares, PhD,[2] Traci E. Clemons, PhD,[3] Freekje van Asten, MD, PhD,[4] Anand Swaroop, PhD,[4] Emily Y. Chew, MD,[1] for the Age-Related Eye Disease Studies (AREDS) 1 and 2 Research Groups**

Purpose: To determine whether closer adherence to a Mediterranean diet (and its individual components) was associated with altered risk of progression to late age-related macular degeneration (AMD) and large drusen. Additional objectives were to assess interactions with AMD genotype.

Design: Retrospective analysis of 2 controlled clinical trial cohorts: Age-Related Eye Disease Study (AREDS) and AREDS2.

Participants: Eyes with no late AMD at baseline in AREDS participants (n = 4255) and AREDS2 participants (n = 3611): total of 13 204 eyes (7756 participants). Mean age was 71 years (standard deviation, 6.6); 56.5% were female.

Methods: Color fundus photographs were collected at annual study visits and graded centrally for late AMD. The modified Alternative Mediterranean Diet Index (aMedi) score was calculated for each participant from food frequency questionnaires.

Main Outcome Measures: Progression to late AMD, geographic atrophy (GA), and neovascular AMD; progression to large drusen.

Results: Over a median follow-up of 10.2 years, of the 13 204 eyes, 34.0% progressed to late AMD. Hazard ratios (HRs) for progression in aMedi tertile 3 versus 1 were 0.78 (95% confidence interval [CI], 0.71–0.85, $P < 0.0001$) for late AMD, 0.71 (0.63–0.80, $P < 0.0001$) for GA, and 0.84 (0.75–0.95, $P = 0.005$) for neovascular AMD. For fish consumption, HRs for late AMD in quartile 4 versus 1 were 0.69 (0.58–0.82, $P < 0.0001$; AREDS) and 0.92 (0.78–1.07, $P = 0.28$; AREDS2). In AREDS, both aMedi and its fish component interacted with *CFH* rs10922109 for late AMD ($P = 0.01$ and $P = 0.0005$, respectively); higher aMedi and fish intake were each associated with decreased risk only in participants with protective alleles. In separate analyses (n = 5029 eyes of 3026 AREDS participants), the HR for progression to large drusen in aMedi tertile 3 versus 1 was 0.79 (0.68–0.93, $P = 0.004$).

Conclusions: Closer adherence to a Mediterranean-type diet was associated with lower risk of progression to late AMD and to large drusen. The signal was greater for GA than neovascular AMD. Fish intake contributed to this protective association. *CFH* genotype strongly influenced these relationships. These findings may help inform evidence-based dietary recommendations. *Ophthalmology 2020;127:1515-1528 Published by Elsevier on behalf of the American Academy of Ophthalmology*

Supplemental material available at www.aaojournal.org.

Age-related macular degeneration (AMD) is the leading cause of legal blindness in developed countries.[1,2] It is classified into early, intermediate, and late stages.[3] Late AMD occurs in 2 forms: geographic atrophy (GA) and neovascular disease. The disease arises from a complex interplay among aging, genetics, and environmental factors, including dietary factors.[4-6] No known therapies slow progression from early to intermediate AMD. However, oral supplements of antioxidants and minerals decrease progression from intermediate to late AMD, particularly neovascular AMD.[7,8] No drug therapies are known to decrease progression to GA or restore lost vision to these areas; therefore, GA prevention is an essential research priority.

The role of diet as a potentially modifiable factor has received interest.[6,9,10] The results of many observational studies suggest that a higher intake of individual dietary vitamins and minerals is associated with a lower risk of early or late AMD, although inconsistencies exist between studies.[10,11] Differences in other nutrients or non-nutritive components in the same foods may help explain the inconsistencies. Non-nutritive components are molecules,

https://doi.org/10.1016/j.ophtha.2020.04.030
ISSN 0161-6420/20

other than carbohydrates, fats, and proteins, present in food. These include vitamins, minerals, water, and fiber. For example, certain fruits and vegetables contain not only vitamin C and carotenoids but also non-nutritive phytochemicals such as flavonoids and nitrates;[12,13] these might be partly responsible for the associations between intake of the more common nutrients and decreased prevalence of AMD. Also, certain vegetables and fruit and vegetable-rich diets are increasingly recognized to promote a healthy gut microbiome.[14-16] Examining relationships with dietary patterns, rather than with individual nutrients, has advantages; it enables researchers to account simultaneously not only for the intake of common nutrients in foods but also for the intake of other bioactive phytochemicals in food components, as well as their potential interactions (which are not well understood).

Definitions of the Mediterranean diet were initially developed to characterize the diets of people living on Crete because they had low rates of coronary artery disease mortality.[17] Modifications to these definitions have since been made for different populations.[18] Adherence to Mediterranean-type diets is associated with multiple health benefits.[19] Few studies have examined whether such diets are associated with altered AMD prevalence or progression.[20-25] Only 2 studies have analyzed longitudinal data.[23,25] In addition, interactions between diet and genotype have been investigated,[21,23,26,27] but few datasets combine dietary and genetic data with sufficient power to address this.

The Age-Related Eye Disease Study (AREDS) and AREDS2 were multicenter phase III randomized controlled trials (RCTs) designed to assess the effects of nutritional supplementation on AMD progression.[28,29] In both, the primary outcome was progression to late AMD. However, important differences exist between the 2 study cohorts: AREDS2 participants all had at least intermediate AMD in both eyes at baseline and were at high risk of progression to late AMD, whereas AREDS participants had a wide spectrum of baseline disease severity, including many participants with no AMD or early AMD. The AREDS2 participants also had higher mean age and higher genetic risk of late AMD.[30]

The aims of this report were to use AREDS and AREDS2 to (1) examine potential associations between Mediterranean diet adherence (overall and by component) and progression to late AMD (GA or neovascular AMD); (2) assess interactions between Mediterranean diet adherence and AMD genotype in progression to late AMD; and (3) analyze potential associations between Mediterranean diet adherence and progression to large drusen.

Methods

Study Populations

The study designs for AREDS and AREDS2 have been described previously.[28,29] In the AREDS, 4757 participants (55–80 years) were recruited (1992–1998) at 11 US retinal specialty clinics and enrolled into AMD categories (no AMD to unilateral late AMD). In AREDS2, 4203 participants (50–85 years) with bilateral large drusen or unilateral late AMD were recruited (2006–2008) at 82 US retinal specialty clinics. Institutional Review Board approval was obtained at each site, and written informed consent was obtained from all participants. The research was conducted under the Declaration of Helsinki and for AREDS2 complied with the Health Insurance Portability and Accessibility Act.

Study Procedures

The AREDS participants were randomly assigned to placebo, antioxidants, zinc, or the combination. The RCT lasted 5 years. The AREDS2 participants were randomly assigned to receive the supplements that lowered risk of AMD progression in the AREDS (1) alone or with additional (2) lutein/zeaxanthin, (3) docosahexaenoic acid (DHA) plus eicosapentaenoic acid (EPA), or (4) the combination. Likewise, the RCT lasted 5 years. In both studies, at baseline and annual visits, eye examinations were performed and color fundus photographs were captured and graded centrally at the Fundus Photographic Reading Center (University of Wisconsin).[31]

In AREDS, after close-out at 5 years, epidemiologic follow-up started immediately in 3549 of the 4203 surviving AREDS participants. In AREDS2, after close-out at 5 years, 2923 participants underwent reassessment at 10 years (AREDS2 10-year follow-on study). Progression to late AMD (including GA/neovascular subtype) was defined by fundus photograph grades in the AREDS and AREDS2 5-year study.[28,29] However, for eyes with no late AMD at AREDS2 5-year close-out, progression to late AMD by 10 years was defined as described in the Supplementary Methods (available at www.aaojournal.org); this was without subtype analysis (i.e., in AREDS2, late AMD was assessed to 10 years, but GA and neovascular AMD to 5 years).

In AREDS, this included the independent assessment of GA and neovascular AMD. Thus, if an eye progressed to neovascular AMD, it was still assessed for additional progression to GA at subsequent study visits (and vice versa). By contrast, in AREDS2, this was not the case. In AREDS2, if an eye progressed to neovascular AMD, it was no longer assessed for additional progression to GA (or vice versa).

Modified Alternative Mediterranean Diet Index Score

In both studies, food frequency questionnaires (FFQs) were administered to all participants at randomization. The AREDS FFQ, a 90-item, semiquantitative modified Block FFQ, and its validation have been described.[32] The AREDS2 FFQ, a 131-item, semiquantitative Harvard FFQ, and its validation have been described.[33,34] In both FFQs, participants were asked how often, on average, they had consumed each food/beverage item during the preceding year.

The FFQs were used to determine the number of medium-sized servings of each food item consumed per week (or gram/day for alcohol). These data were summed for each participant to obtain the intake for each of the 9 components that define adherence to the Mediterranean diet: whole fruits, vegetables, whole grains, nuts, legumes, red meat, fish, monounsaturated fatty acid : saturated fatty acid ratio (MUFA:SFA), and alcohol. The intake distributions were plotted for each component separately for (1) AREDS and AREDS2, and (2) men and women. Because the intake distributions were significantly different between AREDS and AREDS2 for most components, all subsequent component analyses were performed on each cohort separately. Next, for each component, sex-specific intake quartiles (1–4) were calculated (separately for AREDS and AREDS2), with quartile 4 representing highest intake.

Alcohol intake was converted into binary format: 4 for intake within the specified intervals (5−15 g/day [female] or 10−15 g/day [male]) and 1 for intake above or below the specified intervals.[35] The quartiles for red meat were reversed (i.e., quartile 4 with highest intake scored 1, as least modified Alternative Mediterranean Diet Index [aMedi] adherent, and quartile 1 with lowest intake scored 4).

Next, to calculate the aMedi score for each participant, the quartile values for the 9 components were summed (range 9−36). The aMedi distributions were plotted separately for (1) AREDS and AREDS2, and (2) men and women. Because the aMedi distributions were similar in AREDS and AREDS2, subsequent aMedi analyses were performed using (1) a combined AREDS/AREDS2 cohort, (2) AREDS alone, and (3) AREDS2 alone.

For the components, assessment of adherence level by quartile ranks (rather than above or below the median, as in another version of the aMedi[36]) was used to capture more accurately the variation observed in these cohorts.[37] A similar method (based on assigning points for component intakes in the top quartile, rather than above the median) was used in another study population: the Women's Health Initiative, Carotenoids in Age-Related Eye Disease Study.[24] In this study population, high versus low aMedi scores (6−9 vs. 0−1) were associated with lower AMD prevalence 6 years later, as well as with lower mean serum C-reactive protein (3.5 mg/l vs. 5.1 mg/l; $P = 0.005$) and serum triglycerides (1.6 vs. 1.8 mmol/l; $P = 0.02$), and higher densities of macular lutein and zeaxanthin (0.41 vs. 0.34 optical density units; $P < 0.0001$).

In the current study, in sensitivity analyses, a different method was used to construct the aMedi score components according to the methodology used in some previous studies.[23,36] For these analyses, intake was considered above or below the median for each component, and these grades were summed to produce the aMedi (range, 0−9). This method was not used in the primary analyses because of the potential for loss of detailed information at the component level. For the aMedi, assessment of adherence level was analyzed by tertiles, as in many previous studies,[20,23,25] to provide interpretable hazard ratios (HRs).

Genotype Analysis

As part of AREDS and AREDS2, 2889 (AREDS) and 1826 (AREDS2) participants consented to genotype analysis. The single nucleotide polymorphisms (SNPs) were analyzed using a custom Illumina HumanCoreExome array.[38] The AMD Genetic Risk Score (GRS), a weighted risk score for late AMD, was calculated for each participant.[38] Two SNPs at 2 loci (with highest attributable risk of late AMD) were also selected: *ARMS2* rs10490924 and *CFH* rs10922109 (the lead variant at this locus in a large genome-wide association study [GWAS] of late AMD[38]).

Participant Cohorts and Statistical Methods

The eligibility criteria were eyes without late AMD at baseline in White participants with at least 2 study visits. Participants were excluded if they had missing data for the outcomes or for any covariate (described next), or if the FFQ was absent. For the reasons described earlier, the aMedi analyses were performed using the combined AREDS/AREDS2 cohort, as well as each cohort separately, but the component analyses were performed on each cohort separately. In all cases, eyes were divided into 2 groups: (1) those who progressed to late AMD during follow-up and (2) nonprogressors. In unadjusted analyses, these 2 groups were compared (chi-square test) according to aMedi tertiles.

Multivariable proportional hazards regression was performed for progression to (1) late AMD, (2) GA, and (3) neovascular AMD, according to aMedi tertiles (with tertile 1 as reference). Significance was set at $P = 0.017$ (Bonferroni). The proportional hazards assumption was tested in all cases. In the one situation where the assumption was not met (in the combined cohort analysis for the cohort variable), stratified proportional hazards regression was performed instead.[39] The regression analyses were also performed with (1) adjustment for treatment assignment (i.e., the oral supplements or placebo to which participants were randomized) and (2) including the interaction term between aMedi and treatment assignment. In AREDS, this included antioxidants as the main effect and zinc as the main effect, and, in AREDS2, this included DHA/EPA as the main effect and lutein/zeaxanthin as the main effect. For these analyses, follow-up was limited to the duration of the treatment assignment (5 years).

Next, the regression analyses were repeated, including the interaction term between aMedi and genotype. Significance was set at $P = 0.006$ (Bonferroni). Further regression analyses were performed for the 9 aMedi components: Separate models were made for each component (component x), adjusting for the modified total index score that did not include the respective component (modified total index score = total index score − component x). Significance was set at $P = 0.002$ (Bonferroni).

In all cases, the unit of analysis was the eye and analyses were adjusted for age, sex, smoking, total calorie intake, body mass index (BMI) (for AREDS only), and correlation between eyes. Age and total calorie intake were treated as continuous variables, and sex, smoking (assessed by self-report), and BMI were treated as categoric variables (as defined in Table 1). Adjustment for correlation between eyes was made in SAS (SAS Institute Inc., Cary, NC) by using the robust sandwich estimate for the covariance matrix in the Wald tests.[40] In analyses of the combined AREDS/AREDS2 cohort, adjustment was also made for the cohort. The analyses were conducted using SAS version 9.4.

Results

Participant Cohorts: Baseline Characteristics and Unadjusted Analyses

The combined cohort contained 13 204 eligible eyes of 7756 participants. Their characteristics are shown in Table 1, and their dietary intake is shown in Tables S1 and S2 (available at www.aaojournal.org). The numbers that progressed to late AMD, GA, or neovascular AMD by final follow-up (median 10.2 years) were 4487 (34.0%), 2194 (16.6%), and 2018 (15.3%), respectively. In AREDS and AREDS2 considered separately, the numbers that developed late AMD were 1655 (22.0%; median 10.1 years) and 2866 (48.6%; median 10.4 years), respectively.

Proportional Hazards Regression Analyses: Progression to Late Age-Related Macular Degeneration According to the Modified Alternative Mediterranean Diet Index Score

The results of multivariable proportional hazards regression are shown in Figure 1 and Table S3 (available at www.aaojournal.org). The proportional hazards assumption was met in all cases; the only exception was for the cohort variable in the combined cohort analysis, so, for these analyses, the results of stratified proportional hazards regression are shown instead. In the combined cohort, HRs for late AMD were 0.87 (tertile 2; 95% confidence interval [CI], 0.80−0.94, $P = 0.001$) and 0.78 (tertile 3; 95% CI, 0.71−0.85, $P < 0.0001$). Those for GA were 0.80

Table 1. Participant Demographics at Baseline

	AREDS Cohort	AREDS2 Cohort	Combined Cohort (AREDS and AREDS2)
Participants	4255	3611	7756*
Mean age (yrs)	69 (SD 5.1)	73 (SD 7.7)	71 (SD 6.6)
Female: n (%)	2388 (56.1)	2058 (57.0)	4385 (56.5)
Smoking status: n (%)			
Never	1914 (45.0)	1558 (43.1)	3415 (44.0)
Former	2035 (47.8)	1823 (50.5)	3809 (49.1)
Current	306 (7.2)	230 (6.4)	532 (6.9)
BMI: n (%)		–	–
≤25 kg/m^2	1405 (33.0)		
>25 and ≤30 kg/m^2	1791 (42.1)		
>30 kg/m^2	1059 (24.9)		
AMD severity category 3–4 (AREDS) or ≥7 in worse eye (AREDS2): n (%)		–	–
No	1982 (46.6)	845 (23.4)	
Yes	2273 (53.4)	2763 (76.6)	
aMedi tertiles: n (%)			
1	1349 (31.7)	1224 (33.9)	2542 (32.8)
2	1436 (33.7)	1101 (30.5)	2497 (32.2)
3	1470 (34.5)	1286 (35.6)	2717 (35.0)
Mean follow-up time (yrs)	9.2 (SD 2.8) Median 10.1	8.6 (SD 3.2) Median 10.4	8.9 (SD 3.0) Median 10.2
Participants with genetic data	2722	1669	4297
GRS group: n (%)			
Low risk group†	886 (32.5)	204 (12.2)	1069 (24.9)
Quartiles 1–2	1063 (39.1)	596 (35.7)	1623 (37.8)
Quartiles 3–4	773 (28.4)	869 (52.1)	1605 (37.4)
CFH protective alleles (rs10922109): n (%)			
0 (CC)	1259 (46.3)	1119 (67.0)	2332 (54.3)
1 (CA)	1125 (41.3)	472 (28.3)	1562 (36.3)
2 (AA)	338 (12.4)	78 (4.7)	403 (9.4)
ARMS2/HTRA1 risk alleles (rs10490924): n (%)			
0 (GG)	1379 (50.7)	645 (38.6)	1979 (46.1)
1 (GT)	1056 (38.8)	718 (43.0)	1739 (40.5)
2 (TT)	287 (10.5)	306 (18.3)	579 (3.5)

AMD = age-related macular degeneration; aMedi = Alternative Mediterranean Diet Index; AREDS = Age-Related Eye Disease Study; *ARMS2* = *age-related maculopathy susceptibility 2* gene; BMI = body mass index; *CFH* = *complement factor H* gene; GA = geographic atrophy; GRS = Genetic Risk Score; *HTRA1* = *high-temperature requirement A1* gene; SD = standard deviation.
*In the combined cohort, the 110 participants who were in both cohorts were counted only once (using their data from AREDS2).
†Low-risk GRS group: participants whose GRS was less than or equal to the mean GRS of a control population without late AMD.

(95% CI, 0.71–0.90, $P = 0.0002$) and 0.71 (95% CI, 0.63–0.80, $P < 0.0001$), respectively, and for neovascular AMD were 0.90 (95% CI, 0.80–1.01, $P = 0.08$) and 0.84 (95% CI, 0.75–0.95, $P = 0.005$). Thus, higher aMedi was associated with a significantly lower risk of late AMD, with dose-response associations. The HR differences were greater and more highly significant for GA than neovascular AMD. The results for separate analysis of the cohorts are also shown (Fig 1). These were relatively similar for GA; for neovascular AMD, however, a stronger signal was observed in AREDS than AREDS2.

Similar analyses that included adjustment for treatment assignment did not demonstrate different results in AREDS or AREDS2. The HRs for progression to late AMD (aMedi tertile 3 vs. 1), including adjustment, were 0.78 (95% CI, 0.66–0.92) in AREDS and 0.82 (95% CI, 0.73–0.93) in AREDS2. In addition, analyses that included the interaction term between aMedi and treatment assignment showed no significant interactions for any outcome in AREDS or AREDS2, even at the nominal level. The *P* interaction values for progression to late AMD were $P = 0.63$ (antioxidants), $P = 0.19$ (zinc), and $P = 0.95$ (combination) in AREDS, and $P = 0.75$ (DHA/EPA), $P = 0.51$ (lutein/zeaxanthin), and $P = 0.53$ (combination) in AREDS2.

The analyses were repeated using a different version of the aMedi score, constructed as 0–9 (above or below the median for each component)[36] instead of 9–36. In AREDS, the HRs for aMedi tertile 3 (with tertile 1 as reference) were 0.86 (95% CI, 0.73–1.00; $P = 0.049$), 0.83 (95% CI, 0.69–1.00; $P = 0.050$), and 0.89 (95% CI, 0.73–1.08; $P = 0.23$) for late AMD, GA, and neovascular AMD, respectively. In AREDS2, the equivalent HRs were 0.84 (95% CI, 0.74–0.95, $P = 0.006$), 0.81 (95% CI, 0.67–0.96, $P = 0.016$), and 0.89 (95% CI, 0.75–1.05, $P = 0.15$), respectively. Thus, the results showed a similar pattern in all cases but were partially attenuated compared with the original analyses, presumably through loss of information caused by binary assignment above or below the median. The results of the other sensitivity analyses are available in the Supplementary Methods (available at www.aaojournal.org).

In analyses of interactions between aMedi and genotype, in AREDS, overall *P* interaction values for *CFH* rs10922109 were 0.01 (late AMD), 0.003 (GA), and 0.026 (neovascular AMD), that

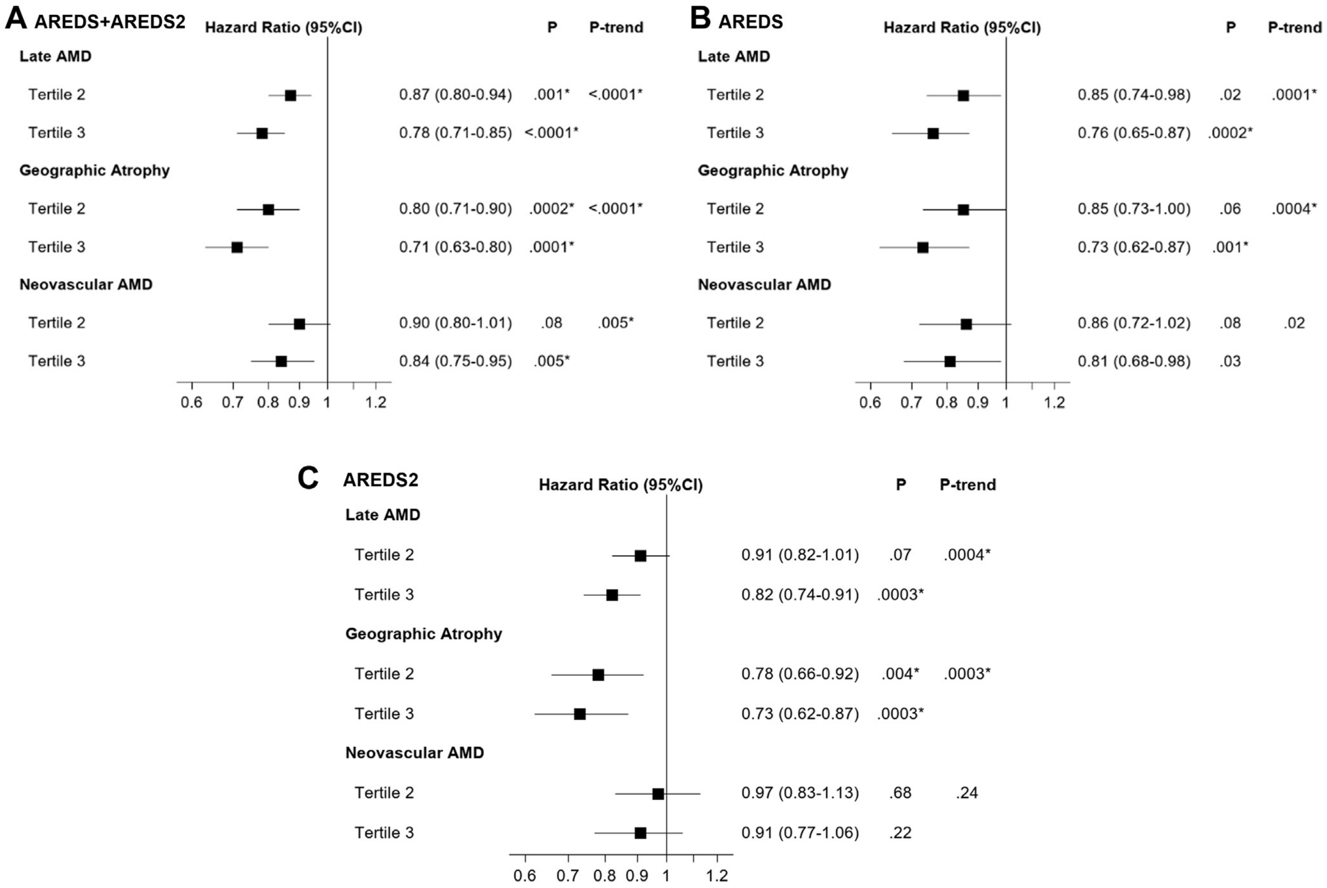

Figure 1. Results of the proportional hazards regression modeling of progression to late age-related macular degeneration (AMD) outcomes according to tertiles of the modified Alternative Mediterranean Dietary Index (aMedi): **(A)** Age-Related Eye Disease Study (AREDS) and AREDS2 (combined cohort); **(B)** AREDS; **(C)** AREDS2. Results are shown in comparison to tertile 1 (reference) after adjustment for age, sex, smoking status, total calorie intake, body mass index (for AREDS participants only), and correlation between eyes. Significant results (according to Bonferroni-corrected significance level of P = 0.017) are shown with an asterisk. **A** and **C,** For the data from AREDS2, the results for late AMD are based on events over the full study period (i.e., including the AREDS2 10-year follow-on study), whereas the results for geographic atrophy (GA) and neovascular AMD are based on events up until the AREDS2 close-out (median 5 years). This is because in the AREDS2 10-year follow-on study, late AMD subtype information was not captured from all data sources. CI = confidence interval.

is, significant at the Bonferroni level for GA and at the nominal level for late and neovascular AMD. In participants with no *CFH* protective alleles, higher aMedi was not associated with altered risk of late AMD (Table 2). In those with 1 protective allele, higher aMedi was associated with decreased risk of late AMD outcomes; for GA, HRs were as low as 0.52 (95% CI, 0.34−0.77, P = 0.001) for aMedi tertile 3 versus 1. The number of participants with 2 protective alleles was very low, because it is rare to be homozygous for this allele. As expected, the number of progression events in these individuals was extremely low because it is unusual for individuals with 2 protective alleles at this locus to progress to late AMD (irrespective of diet). Thus, although the HRs for progression to late AMD were low for aMedi tertiles 2 and 3, these results were not statistically significant. In similar analyses of AREDS2, no significant interactions were present. The likely reasons for the differing results between AREDS and AREDS2 are discussed next. In similar analyses of *ARMS2* rs10490924, no significant interactions were present for any late AMD outcome in AREDS or AREDS2. For the AMD GRS, again, no significant interactions were present for any outcome in either cohort.

Proportional Hazards Regression Analyses: Progression to Late Age-Related Macular Degeneration According to Intake of Individual Components

The results for individual aMedi components are shown in Figure 2 and Table S4 (available at www.aaojournal.org) (separately for AREDS and AREDS2, because intake distributions differed between the 2 studies). The magnitudes of protective associations with fish (and to a lesser extent vegetables and whole grains) and adverse associations with MUFA:SFA are described next.

Fish Intake

In AREDS, higher fish intake was associated with decreased risks of late AMD, GA, and neovascular AMD. In AREDS2, HRs for higher fish intake were numerically lower than 1 for GA but similar to 1 for neovascular AMD; none was significantly different to 1 at the Bonferroni level, although several met nominal significance. Further analyses, including assignment to DHA/EPA supplement intervention group as main effect, did not alter the results for

Table 2. Results of Proportional Hazards Regression of the Progression to Late Age-Related Macular Degeneration Outcomes in the AREDS (n = 4992 eyes of 2722 participants), According to Interactions between Participant Genotype at *CFH* rs10922109 and the Modified Alternative Mediterranean Diet Index

Outcome	*CFH* Alleles (rs10922109): A = Protective; C = Wild-type	aMedi Tertile (Reference = 1)	No. of Eyes	No. of Events	HR (95% Confidence Limits)	*P* Value
Late AMD	0 (CC)	2	768	246	0.91 (0.74−1.13)	0.39
		3	779	247	0.92 (0.74−1.15)	0.48
	1 (CA)	2	742	101	0.69 (0.51−0.93)	0.01
		3	757	97	0.61 (0.43−0.85)	0.003
	2 (AA)	2	232	13*	0.76 (0.32−1.79)	0.53*
		3	245	12*	0.65 (0.23−1.85)	0.42*
GA	0 (CC)	2	768	172	0.94 (0.73−1.21)	0.61
		3	779	179	0.91 (0.71−1.18)	0.49
	1 (CA)	2	742	78	0.68 (0.48−0.97)	0.03
		3	757	60	0.52 (0.34−0.77)	0.001
	2 (AA)	2	232	9*	0.63 (0.24−1.65)	0.35*
		3	245	9*	0.61 (0.21−1.78)	0.36*
Neovascular AMD	0 (CC)	2	768	137	0.89 (0.69−1.15)	0.37
		3	779	142	0.98 (0.74−1.29)	0.88
	1 (CA)	2	742	53	0.63 (0.43−0.93)	0.02
		3	757	55	0.59 (0.39−0.89)	0.01
	2 (AA)	2	232	6*	†	†
		3	245	7*	†	†

AMD = age-related macular degeneration; aMedi = modified alternative Mediterranean Diet Index; *CFH* = *complement factor H* gene; GA = geographic atrophy; HR = hazard ratio.
*Very low event numbers because of very low likelihood of progression to late AMD in those with 2 protective alleles (irrespective of diet), thus no statistical significance despite HRs <1.
†No meaningful results because of very low numbers of events.

AREDS2, and there was no significant interaction between fish intake and DHA/EPA supplementation (*P* interaction = 0.64 for late AMD). Likely reasons for the weaker association between fish intake and progression to late AMD in AREDS2 include lower median fish intake, substantially higher median baseline AMD status, and lower prevalence of *CFH* rs10922109 protective alleles (33% vs. 54%) in AREDS2 than AREDS; these are considered in more detail next.

In analyses of interactions between fish intake and genotype, in AREDS, highly significant interactions were observed for *CFH* rs10922109 (*P* interaction = 0.0005, 0.0008, and 0.009 for late AMD, GA, and neovascular AMD, respectively). In participants with no *CFH* protective alleles, higher fish intake was not associated with altered risk of late AMD (Table 3). However, in those with protective alleles, higher fish intake was associated with substantially decreased risk of late AMD outcomes. The number of participants with 2 protective alleles was very low. In similar analyses of AREDS2, no significant interactions were present. This was unsurprising, given the weak signals for overall associations between fish intake and late AMD outcomes. Indeed, the reasons for the differing results between AREDS and AREDS2 are likely the same as those for the overall results for fish. In addition, an interaction was observed in AREDS between GRS and fish intake, for neovascular AMD (*P* = 0.007; Table 3), but not late AMD (*P* interaction = 0.07) or GA (*P* interaction = 0.91). No significant interactions were found with *ARMS2*.

Monounsaturated : Saturated Fatty Acid Intake

Higher MUFA:SFA intake quartiles were associated with increased risk of late AMD in AREDS (*P* trend = 0.001), but not in AREDS2 (*P* trend = 0.36). In exploratory analyses, we found that the association was limited to women; in AREDS, the HRs for late AMD in MUFA:SFA quartiles 4 versus 1 were 1.41 (1.14−1.74; *P* = 0.001) in women and 1.15 (0.91−1.46; *P* = 0.24) in men. The likely reasons for differences between the results for AREDS and AREDS2 are discussed in the Supplement Methods (available at www.aaojournal.org).

Proportional Hazards Regression Analyses: Progression to Large Drusen

In separate analyses of eyes with no large drusen or late AMD at baseline (n = 5029 of 3026 AREDS participants), HRs for the development of large drusen, according to aMedi tertiles, were 0.87 (tertile 2; 0.75−1.01, *P* = 0.07) and 0.79 (tertile 3; 0.68−0.93, *P* = 0.004). In similar analyses according to fish intake quartiles, the HRs were not significantly different to 1. No genetic interaction was observed between aMedi and *CFH* rs10922109 status (*P* interaction = 0.21).

Discussion

Main Findings, Interpretation, and Comparison with Literature

Higher aMedi adherence was associated with a lower risk of late AMD. This was true for late AMD overall, for GA, and (with a weaker association) for neovascular AMD. Similar results for late AMD were observed in both AREDS and AREDS2, despite the substantial differences between the 2 cohorts (including decade of study, participant age, genetic risk profile, dietary habits, FFQ used, as well as the very

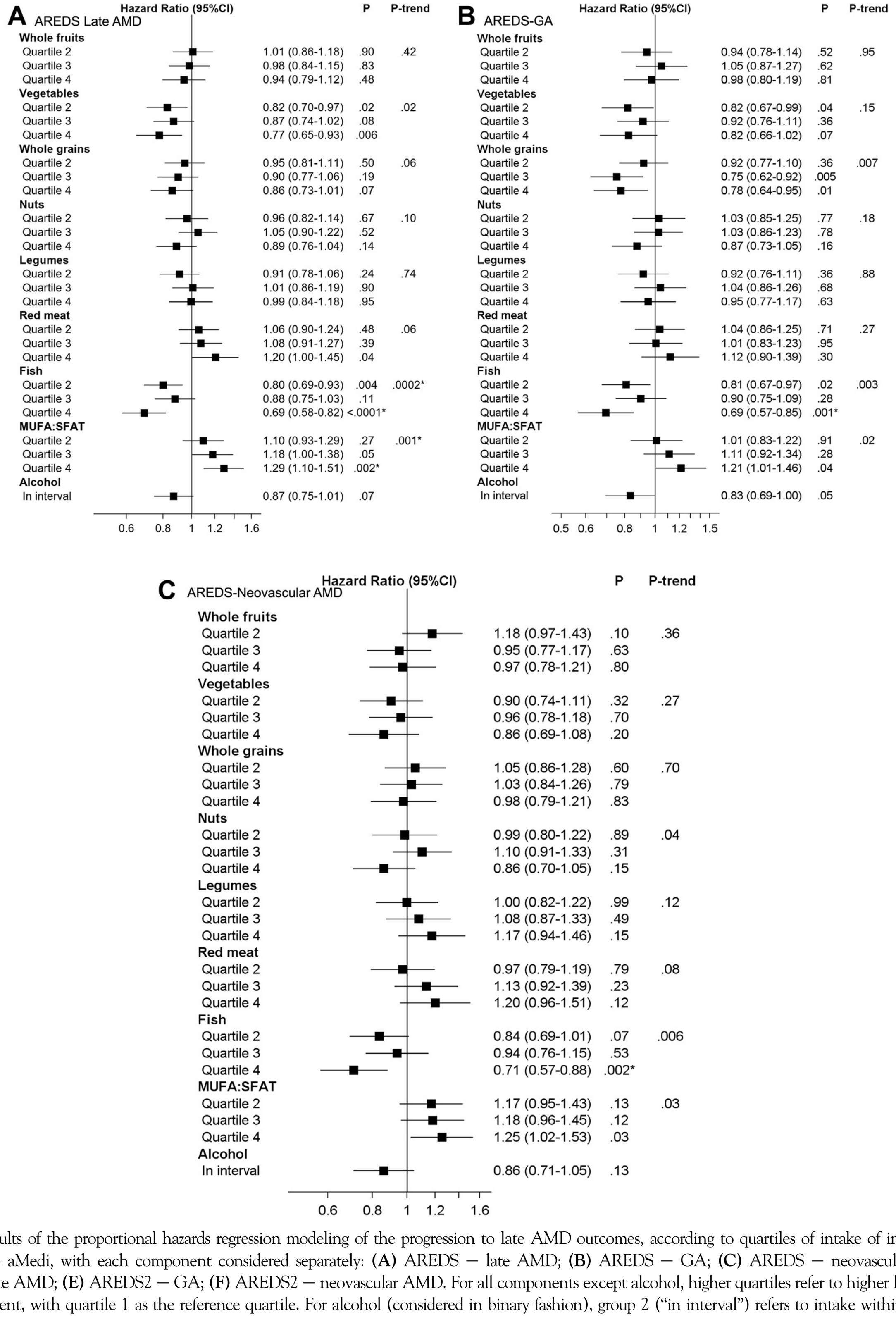

Figure 2. Results of the proportional hazards regression modeling of the progression to late AMD outcomes, according to quartiles of intake of individual components of the aMedi, with each component considered separately: **(A)** AREDS – late AMD; **(B)** AREDS – GA; **(C)** AREDS – neovascular AMD; **(D)** AREDS2 – late AMD; **(E)** AREDS2 – GA; **(F)** AREDS2 – neovascular AMD. For all components except alcohol, higher quartiles refer to higher levels of intake of the component, with quartile 1 as the reference quartile. For alcohol (considered in binary fashion), group 2 ("in interval") refers to intake within the specified interval (i.e., adherent to the aMedi), and group 1 (reference) refers to intake above or below the specified interval. For red meat, higher quartiles refer to higher levels of intake, which is less adherent to the modified alternative Mediterranean diet. For monounsaturated fatty acid : saturated fatty acid (MUFA:SFA), higher quartiles refer to higher ratios of MUFA:SFA intake, which is considered more adherent to the aMedi. Significant results (according to Bonferroni-corrected significance level of $P = 0.002$) are shown with an asterisk. AREDS2 **(D–F)**: The results for late AMD **(D)** are based on events over the full study period (i.e., including the AREDS2 10-year follow-on study), whereas the results for GA and neovascular AMD **(E–F)** are based on events up until the AREDS2 close-out (median 5 years). This is because, in the AREDS2 10-year follow-on study, late AMD subtype information was not captured from all data sources. CI = confidence interval.

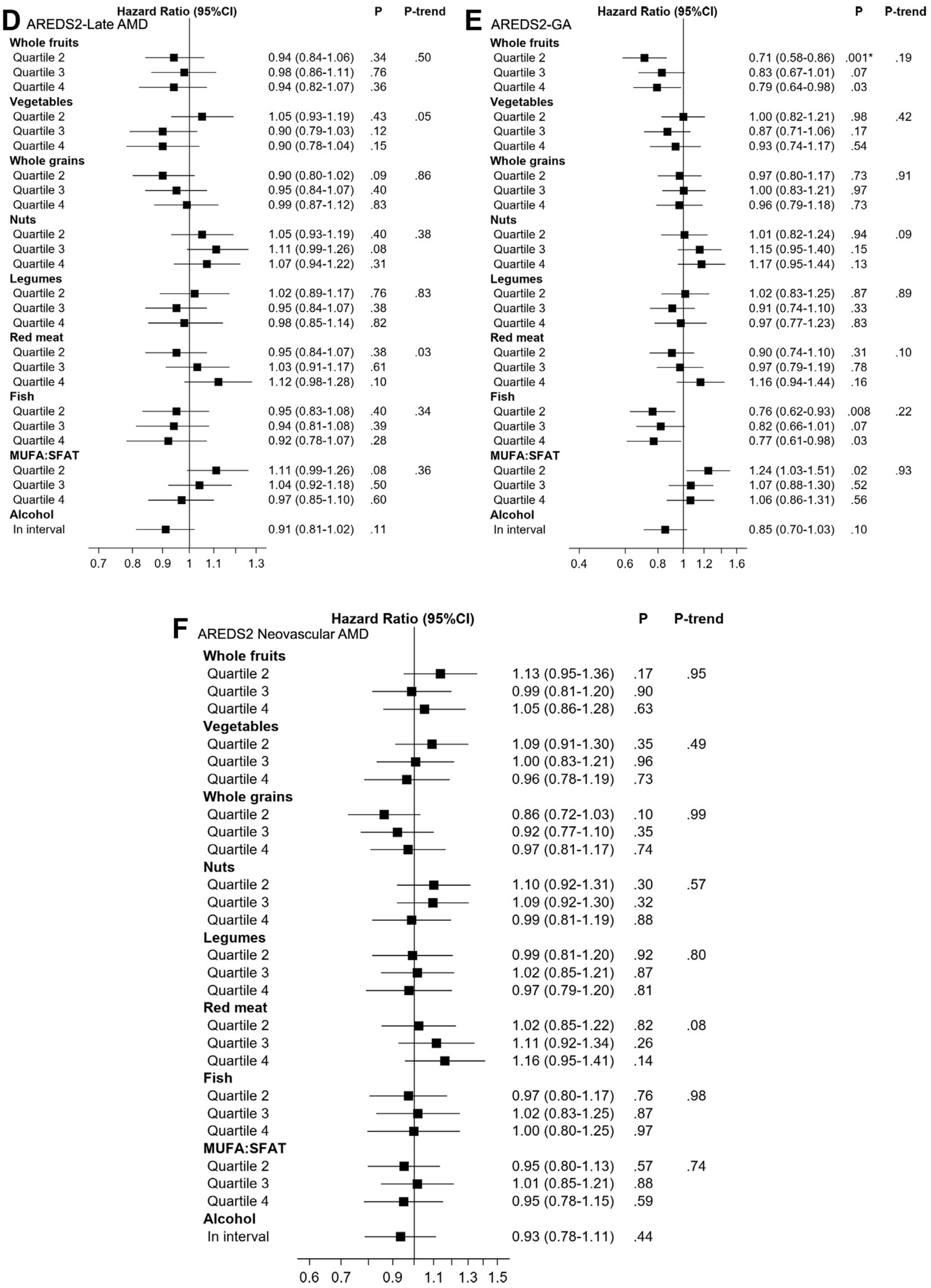

Figure 2. (Continued.)

high level of baseline disease severity in AREDS2). The findings were consistent with dose-response effects and robust to multiple sensitivity analyses, including construction of the aMedi by a different method used in some previous studies.[23,36] The evidence for GA was highly consistent between datasets, but for neovascular AMD was apparent only in AREDS. Higher adherence was also associated with lower risk of large drusen development; this represents a novel and important finding, because (aside from smoking cessation) no interventions are available to decrease progression to this highly prevalent disease stage. Notably, this suggests that the

Table 3. Results of Proportional Hazards Regression of the Progression to Late Age-Related Macular Degeneration Outcomes in the AREDS (n = 4992 Eyes of 2722 Participants), According to Interactions between Fish Intake and Participant Genotype at (i) *CFH* rs10922109 and (ii) Age-Related Macular Degeneration Genetic Risk Score

Outcome	*CFH* Alleles (rs10922109): A = Protective; C = Wild-type	Fish Intake Quartiles (Reference = 1)	No. of Eyes	No. of Events	HR (95% CL)	*P* Value
Late AMD	0 (CC)	2	609	187	0.94 (0.74–1.20)	0.63
		3	562	195	1.05 (0.82–1.34)	0.72
		4	535	166	0.94 (0.72–1.23)	0.67
	1 (CA)	2	587	85	0.74 (0.52–1.04)	0.08
		3	458	72	0.81 (0.56–1.17)	0.26
		4	602	74	0.63 (0.43–0.92)	0.02
	2 (AA)	2	195	13	0.67 (0.28–1.61)	0.37
		3+4*	319	10	0.19 (0.06–0.58)	0.003
GA	0 (CC)	2	609	127	0.84 (0.63–1.12)	0.24
		3	562	141	1.02 (0.76–1.36)	0.90
		4	535	121	0.89 (0.65–1.23)	0.49
	1 (CA)	2	587	63	0.73 (0.49–1.08)	0.12
		3	458	46	0.68 (0.44–1.05)	0.08
		4	602	51	0.63 (0.41–0.97)	0.04
	2 (AA)	2	195	10	0.14 (0.06–0.37)	<.0001
		3+4*	319	8	0.20 (0.06–0.67)	0.01
Neovascular AMD	0 (CC)	2	609	104	1.02 (0.76–1.38)	0.89
		3	562	104	1.06 (0.77–1.45)	0.71
		4	535	100	0.99 (0.71–1.37)	0.94
	1 (CA)	2	587	50	1.06 (0.68–1.66)	0.80
		3	458	48	1.32 (0.82–2.11)	0.25
		4	602	41	0.84 (0.51–1.37)	0.48
	2 (AA)	3+4†	319	3	0.16 (0.03–0.83)	0.03

Outcome	AMD GRS Group	Fish Intake Quartiles (Reference = 1)	No. of Eyes	No. of Events	HR (95% CL)	*P* Value
Neovascular AMD	0	2	496	16	0.54 (0.24–1.24)	0.15
		3	363	12	1.00 (0.38–2.65)	1.00
		4	473	11	0.78 (0.29–2.07)	0.61
	1	2	553	55	0.76 (0.51–1.13)	0.17
		3	478	51	0.83 (0.54–1.28)	0.40
		4	506	34	0.50 (0.31–0.80)	0.004
	2	2	342	90	1.22 (0.87–1.71)	0.25
		3	329	90	1.18 (0.84–1.67)	0.34
		4	327	98	1.30 (0.91–1.86)	0.16

AMD = age-related macular degeneration; *CFH* = *complement factor H* gene; CL = confidence limits; GA = geographic atrophy; GRS = Genetic Risk Score; HR = hazard ratio.
*Quartiles 3 and 4 combined because of low numbers of events.
†Quartiles 3 and 4 combined (reference = quartiles 1 and 2 combined) because of low numbers of events.

Mediterranean diet may be protective both against late AMD (even at the intermediate AMD stage) and against intermediate disease itself.

These results are partially consistent with a previous study of an AREDS subset: higher aMedi was associated with decreased risk of late AMD.[23] However, late AMD subtype analysis was not performed in this study. This is important, because our study revealed distinct patterns for each subtype; indeed, GA carries a large public health burden and represents a vital research priority because no treatments are known to prevent GA or restore vision to affected areas. Only one other study has reported prospectively obtained data.[25] In this European study, higher MED score (i.e., using the original Mediterranean Dietary Index,[41] rather than one alternative version[36] or the other alternative version used here) was associated with decreased late AMD risk (*P* trend = 0.04). Two European studies have reported cross-sectional associations between Mediterranean-type diets and AMD (although interpretation of cross-sectional associations is limited by susceptibility to reverse causation). One observed that higher MED was associated with lower prevalence of neovascular AMD but not GA.[21] The other found an association between higher MED score and lower AMD prevalence (without late AMD analysis).[20]

Indeed, we consider that more accurate findings might have been observed in the previous studies if they had used methods similar to ours; together with lower power, this may explain some borderline significant results in these previous studies. This idea is supported by the observation that similar patterns of results were obtained, but with attenuated HRs and significance levels, when we

reanalyzed our data with the aMedi constructed in the other way.

Since the original development of a traditional Mediterranean diet score,[41] adaptations have been developed to reflect the partially different dietary intakes and cultures outside the Mediterranean region, while still capturing evidence for the health benefits of its components: diets rich in plant foods, low in red or processed meat, with regular consumption of foods that provide omega-3 fatty acids (i.e., fish, nuts, whole grains, and legumes), and moderate alcohol intake (mostly as wine).[18,42] Likewise, methods for scoring adherence to Mediterranean diet-type patterns or components have been adapted to best capture the levels and distributions in specific populations, as discussed previously.[18,42] The aMedi score was a modification of the traditional Mediterranean Diet Index score, which was developed to examine the dietary patterns found on the island of Crete.[17,41] The modifications were made to reflect its use in other European and North American populations, according to dietary patterns that were consistently associated with lower risks of chronic disease. The modifications comprised excluding potatoes from the vegetable group, adding a nut category (i.e., separate from fruits), limiting the grain category to whole grains (i.e., not refined grains), limiting the meat group to red and processed meat only, and eliminating the dairy group.[18,42] Despite these modifications, there is broader evidence for the benefits of Mediterranean-type patterns across many scoring methods and study samples across the world, in terms of decreased risks of age-related chronic diseases that share risk factors with AMD, and of decreased levels of biomarkers of inflammation.[19] The aMedi score, expressed as high versus low quintiles, was associated with lower risk for cardiovascular disease mortality in a sample of large American cohorts.[37] Likewise, C-reactive protein levels have consistently been observed to be lower with Mediterranean-type diet patterns in both RCTs[43] and observational studies[36] across many countries.

In the present study, the results did not differ according to treatment assignment group in AREDS or AREDS2, and no interactions were observed between the aMedi and the treatment assignment. Thus, the protective association between aMedi and progression to late AMD is independent of any of the oral supplements administered in either study, comprising antioxidants, zinc, lutein/zeaxanthin, and DHA/EPA. This suggests that the Mediterranean diet (which was associated preferentially with decreased risk of GA) might play a complementary role alongside oral supplements (which preferentially decrease the risk of neovascular AMD[7,8]), rather than a competitive role. Likewise, the results were not affected by multivitamin intake in AREDS participants and persisted despite almost universal multivitamin intake in AREDS2 participants.

Interactions between Mediterranean Diet and Age-Related Macular Degeneration Genotype

Evidence for interactions between aMedi and genotype, specifically *CFH* rs10922109, was observed in AREDS. The association between higher aMedi and decreased late AMD was found only in those with *CFH* protective alleles. Of note, this suggests that individuals with this variant (which helps define a protective haplotype at *CFH/CFHR*) may have the most to gain from Mediterranean diets; if they do not follow a Mediterranean diet, they might not be taking full advantage of their genotype. Additional preventative strategies may be required for individuals without protective alleles.

As described earlier, this finding was observed in participants with 1 protective allele. Although similar HRs were observed for late AMD and GA in participants with 2 protective alleles, these results were not statistically significant. This was expected: It is rare to be homozygous for the *CFH* protective allele and very rare to be homozygous and to progress to late AMD (demonstrated by the extremely low numbers of events in Table 2). These rare individuals are almost always highly protected against developing late AMD, irrespective of diet. Thus, it would require extremely high power to demonstrate a significant difference in progression to late AMD according to Mediterranean diet adherence within this group.

Both previous longitudinal studies analyzed genetic interactions.[23,25] The European study[25] observed no significant interaction between dietary index and *CFH* or *ARMS2*, whereas the AREDS study[23] reported an interaction with *CFH* ($P = 0.04$). However, these studies may have missed relevant signals by selecting *CFH* rs1061170 (risk variant) rather than rs10922109. Absence of rs1061170 risk alleles may have been an imperfect proxy for presence of rs10922109 protective alleles. Indeed, we reanalyzed using rs1061170 and observed P interaction $= 0.05$ (GA in AREDS), similar to the previous result.[23]

We applied a 52-SNP GRS to these analyses; this risk score encompasses in a single metric the overall genetic load of late AMD for an individual by including all the risk and protective variants identified in the largest GWAS of late AMD to date.[38] This novel application demonstrated an important distinction through comparison of the results for the GRS and *CFH*. It suggests that the *CFH* status, but not the overall genetic load for late AMD, is important in determining whether closer adherence to the Mediterranean diet is associated with decreased risk. In public health terms, this suggests that different interventions might be required according to individual genotype.

Individual Components of the Modified Alternative Mediterranean Dietary Index

An innovative methodology was used to isolate the contribution of each separate component to the overall results by analyzing according to each component while simultaneously adjusting for a tailored aMedi that excludes that component. This technique is well validated in other fields[44] but novel in AMD studies. The approach demonstrated that fish intake was particularly important. Higher fish intake was significantly associated with decreased risk of GA and neovascular AMD in AREDS; with nominal significance, similar patterns were observed for GA in AREDS2.

The finding of a protective association with fish intake may raise a question regarding meat intake: Individuals with higher fish intake are likely to have lower red meat intake, so uncovering the true association becomes important. First, it is unlikely that higher fish intake is associated purely as a proxy for lower red meat intake because the strength and significance of the associations were greater for fish than for red meat. Second, the regression analyses for each component included adjustment for a modified aMedi index excluding that particular component to isolate as much as possible the signal for each particular component (against the background of the overall aMedi). For example, all analyses for fish were adjusted for the modified aMedi index constructed from the other 8 components (including red meat). Third, additional analyses were performed where 4 groups of participants were selected from the AREDS cohort, according to high (quartile 4) or low (quartile 1) intake of fish and red meat. All 4 combinations were considered (low/low; low/high; high/low; high/high) and HRs for progression to late AMD were calculated. Comparison of the low fish/low meat versus the high fish/low meat groups showed a large difference in HRs. Likewise, comparison of the low fish/high meat versus the high fish/high meat groups showed a difference in HRs. Thus, by comparing groups of participants with similar red meat intake but differing fish intake, we continued to observe a protective association between fish intake and progression to late AMD.

Likely reasons for the differences in results between AREDS and AREDS2 are considered here. Indeed, these same factors are likely to explain the partially different findings between AREDS and AREDS2 for 3 results: (1) fish intake-*CFH* interactions; (2) aMedi-*CFH* interactions, likely driven by (1); and (3) fish intake and progression to late AMD, affected by (1). These factors include (a) the lower median weekly fish intake in AREDS2 (e.g., 1.0 medium servings/week vs. 1.8 in AREDS, in men), (b) the lower proportion of participants with *CFH* protective alleles in AREDS2 (33% vs. 54% in AREDS), (c) the degree of relatively advanced AMD at study baseline in AREDS2, and (d) differences in food items listed on the FFQs and the underlying databases. In addition, participants in AREDS2 appear to have been better nourished at baseline than those in AREDS, including higher intakes of DHA, EPA, and alpha-linolenic acid omega-3 fatty acids, despite reporting fewer weekly fish servings. In AREDS participants, the mean estimated intake of DHA and EPA (59 mg/day) was 3-fold lower than in AREDS2 participants and less than the Institute of Medicine recommendations for men (160 mg) and women (110 mg).[45] The higher intake of these long-chain omega-3 fatty acids in AREDS2 could reflect a greater proportion of fish intake from fish that were rich in omega-3 in this more recent study.

The findings for fish intake are consistent with some previous studies. The previous AREDS study detected an association ($P = 0.04$) between fish intake and decreased late AMD.[23] A cross-sectional European study observed decreased risk of neovascular AMD with high fish intake,[46] and an American case-control study found an association between fish intake and decreased AMD risk when dietary linoleic acid is low.[47] A 2008 meta-analysis concluded that consumption of fish (and foods rich in omega-3 fatty acids) may be associated with lower risk of AMD.[48]

Compelling evidence was observed for a highly significant interaction between fish intake and genotype, specifically *CFH* rs10922109. The novelty and strength of this finding were achieved by isolating the genetic interaction in 3 dimensions: to the fish component of the aMedi, the *CFH* rs10922109 variant of the AMD genotype, and the GA subtype of late AMD. Again, the association between fish intake and decreased late AMD was found only in those with *CFH* protective alleles. Thus, individuals with protective haplotypes may have the most to gain from fish intake. The combination of a protective haplotype and higher fish intake appears particularly powerful. This has important biological and public health implications. The strong genotype dependence argues that the biological mechanism underlying potential protection from fish is intimately involved with the complement system. Further research to investigate interactions between fish nutrients and complement regulation is appropriate.

These results have implications for findings from previous studies. The odds ratios of late AMD associated with *CFH* rs10922109 minor allele (0.38 by GWAS[38]) are likely dependent on the dietary status of the study population. Likewise, previous studies of diet and AMD were likely influenced by *CFH/CFHR* genotypes. The results may help explain geographical differences in AMD and highlight the partially distinct nature of *CFH*-related and *ARMS2/HTRA1*-related AMD. For example, given the findings of this study, we would expect countries where the population consume a lot of fish (e.g. Japan) to have a lower prevalence of late AMD, particularly GA. This is indeed the case.[49-51] As regards specific nutrient(s) involved, the AREDS2 findings suggest these may not be related solely to DHA/EPA. Fish is also rich in B vitamins; deficiencies of folate and vitamin B12 have been associated with increased AMD,[52] and daily supplementation of these vitamins (and B6) decreased the incidence of visually significant AMD in an RCT.[53] In addition, very long chain polyunsaturated fatty acids (i.e., with carbon chains longer than DHA and EPA), which are present in fish but not in fish oil supplements, could also be relevant.[54]

Components other than fish were likely associated with decreased late AMD, such as vegetables, whole grains, and fruit. The association was driven by findings for GA, a pattern that differed from fish. Indeed, the presence of distinct patterns of findings for different aMedi components (GA vs. neovascular AMD and AREDS vs. AREDS2) suggests that more than 1 biological mechanism may be responsible for the beneficial associations of the Mediterranean diet, with each mechanism dependent on a subset of components/nutrients, although more complex interactions may also be relevant (e.g., altered gastrointestinal microbiome[55-57]). The results for MUFA:SFA ratio are discussed in the Supplementary Methods (available at www.aaojournal.org).

Study Strengths and Limitations

Strengths include the use of 2 datasets, both with large size and long follow-up, which provided a high event rate. The datasets benefit from standardized collection of information, reading center grading, and genetic data in most participants. Limitations include post hoc hypothesis generation, the possibility of residual or unmeasured confounding (e.g., physical activity), and differences in variables between the cohorts (e.g., BMI). In addition, diet assessment by FFQ is known to contain nondifferential measurement error, although energy adjustment may partially address this error.[58,59] Because of inherent differences in AREDS and AREDS2 FFQs, differences exist in assignment of food items to the modified aMedi components. The study may have limited generalizability to populations in whom diets, and genotypes, differ.

In conclusion, in the United States, adherence to a Mediterranean-type diet is associated with decreased progression to large drusen and to late AMD. This includes both subtypes of late disease, particularly GA. The potential benefits apply across a wide spectrum of disease severity. The association is present even in relatively established disease (i.e., intermediate AMD). This suggests it may rarely be too early or late to adopt a healthy diet. For progression from intermediate AMD to GA, this is important, because (other than smoking cessation) no interventions are available. The diet is less associated with decreased progression from intermediate to neovascular disease, but AREDS/AREDS2 supplements are beneficial here. Thus, diet and oral supplementation may play complementary roles, with a Mediterranean diet acting preferentially against atrophy and supplementation preferentially against neovascularization. Fish intake is an important driver in the associations. As regards *CFH* status, adopting a Mediterranean diet with fish intake might be considered as exploiting protective alleles, rather than "eating away genetic risk." However, in progression to large drusen and from intermediate AMD to GA, potential benefits appear less dependent on AMD genotype. Further research may uncover the mechanisms involved toward the generation of additional oral supplements. In addition, an RCT of the Mediterranean diet on AMD progression would be essential, ideally encompassing a broad spectrum of disease severity, genotypes, and phenotypes.

Acknowledgment

The authors thank Jill Reedy, PhD, from the National Cancer Institute/National Institutes of Health, for advice on the analyses of the components of the alternative Mediterranean Diet.

References

1. Quartilho A, Simkiss P, Zekite A, et al. Leading causes of certifiable visual loss in England and Wales during the year ending 31 March 2013. *Eye (Lond)*. 2016;30:602−607.
2. Congdon N, O'Colmain B, Klaver CC, et al. Causes and prevalence of visual impairment among adults in the United States. *Arch Ophthalmol*. 2004;122:477−485.
3. Ferris 3rd FL, Wilkinson CP, Bird A, et al. Clinical classification of age-related macular degeneration. *Ophthalmology*. 2013;120:844−851.
4. Fritsche LG, Fariss RN, Stambolian D, et al. Age-related macular degeneration: genetics and biology coming together. *Annu Rev Genomics Hum Genet*. 2014;15:151−171.
5. Chakravarthy U, Wong TY, Fletcher A, et al. Clinical risk factors for age-related macular degeneration: a systematic review and meta-analysis. *BMC Ophthalmol*. 2010;10:31.
6. Chapman NA, Jacobs RJ, Braakhuis AJ. Role of diet and food intake in age-related macular degeneration: a systematic review. *Clin Exp Ophthalmol*. 2019;47:106−127.
7. Chew EY, Clemons TE, Agron E, et al. Long-term effects of vitamins C and E, beta-carotene, and zinc on age-related macular degeneration: AREDS Report No. 35. *Ophthalmology*. 2013;120:1604−1611.
8. Age-Related Eye Disease Study Research Group. A randomized, placebo-controlled, clinical trial of high-dose supplementation with vitamins C and E, beta carotene, and zinc for age-related macular degeneration and vision loss: AREDS report no. 8. *Arch Ophthalmol*. 2001;119:1417−1436.
9. Carneiro A, Andrade JP. Nutritional and lifestyle interventions for age-related macular degeneration: a review. *Oxid Med Cell Longev*. 2017;2017:6469138.
10. Gorusupudi A, Nelson K, Bernstein PS. The Age-Related Eye Disease 2 Study: micronutrients in the treatment of macular degeneration. *Adv Nutr*. 2017;8:40−53.
11. Zampatti S, Ricci F, Cusumano A, et al. Review of nutrient actions on age-related macular degeneration. *Nutr Res*. 2014;34:95−105.
12. Gopinath B, Liew G, Kifley A, et al. Dietary flavonoids and the prevalence and 15-y incidence of age-related macular degeneration. *Am J Clin Nutr*. 2018;108:381−387.
13. Gopinath B, Liew G, Kifley A, et al. Association of dietary nitrate intake with the 15-year incidence of age-related macular degeneration. *J Acad Nutr Diet*. 2018;118:2311−2314.
14. Kaczmarek JL, Liu X, Charron CS, et al. Broccoli consumption affects the human gastrointestinal microbiota. *J Nutr Biochem*. 2019;63:27−34.
15. Graf D, Di Cagno R, Fak F, et al. Contribution of diet to the composition of the human gut microbiota. *Microb Ecol Health Dis*. 2015;26:26164.
16. Nagpal R, Neth BJ, Wang S, et al. Modified Mediterranean-ketogenic diet modulates gut microbiome and short-chain fatty acids in association with Alzheimer's disease markers in subjects with mild cognitive impairment. *EBioMedicine*. 2019;47:529−542.
17. Menotti A, Kromhout D, Blackburn H, et al. Food intake patterns and 25-year mortality from coronary heart disease: cross-cultural correlations in the Seven Countries Study. The Seven Countries Study Research Group. *Eur J Epidemiol*. 1999;15:507−515.
18. Davis C, Bryan J, Hodgson J, Murphy K. Definition of the Mediterranean Diet; a literature review. *Nutrients*. 2015;7:9139−9153.
19. Dinu M, Pagliai G, Casini A, Sofi F. Mediterranean diet and multiple health outcomes: an umbrella review of meta-analyses of observational studies and randomised trials. *Eur J Clin Nutr*. 2018;72:30−43.
20. Nunes S, Alves D, Barreto P, et al. Adherence to a Mediterranean diet and its association with age-related macular degeneration. The Coimbra Eye Study-Report 4. *Nutrition*. 2018;51-52:6−12.

21. Hogg RE, Woodside JV, McGrath A, et al. Mediterranean Diet Score and its association with age-related macular degeneration: The European Eye Study. *Ophthalmology*. 2017;124: 82−89.
22. Piermarocchi S, Tognetto D, Piermarocchi R, et al. Risk factors and age-related macular degeneration in a Mediterranean-Basin population: The PAMDI (Prevalence of Age-Related Macular Degeneration in Italy) Study–Report 2. *Ophthalmic Res*. 2016;55:111−118.
23. Merle BM, Silver RE, Rosner B, Seddon JM. Adherence to a Mediterranean diet, genetic susceptibility, and progression to advanced macular degeneration: a prospective cohort study. *Am J Clin Nutr*. 2015;102:1196−1206.
24. Mares JA, Voland RP, Sondel SA, et al. Healthy lifestyles related to subsequent prevalence of age-related macular degeneration. *Arch Ophthalmol*. 2011;129:470−480.
25. Merle BMJ, Colijn JM, Cougnard-Gregoire A, et al. Mediterranean Diet and incidence of advanced age-related macular degeneration: The EYE-RISK Consortium. *Ophthalmology*. 2019;126:381−390.
26. Meyers KJ, Liu Z, Millen AE, et al. Joint associations of diet, lifestyle, and genes with age-related macular degeneration. *Ophthalmology*. 2015;122:2286−2294.
27. Rowan S, Taylor A. Gene-diet interactions in age-related macular degeneration. *Adv Exp Med Biol*. 2016;854:95−101.
28. Age-Related Eye Disease Study Research Group. The Age-Related Eye Disease Study (AREDS): design implications. AREDS report no. 1. *Control Clin Trials*. 1999;20: 573−600.
29. AREDS2 Research Group, Chew EY, Clemons T, et al. The Age-Related Eye Disease Study 2 (AREDS2): study design and baseline characteristics (AREDS2 report number 1). *Ophthalmology*. 2012;119:2282−2289.
30. Ding Y, Liu Y, Yan Q, et al. Bivariate analysis of age-related macular degeneration progression using genetic risk scores. *Genetics*. 2017;206:119−133.
31. Danis RP, Domalpally A, Chew EY, et al. Methods and reproducibility of grading optimized digital color fundus photographs in the Age-Related Eye Disease Study 2 (AREDS2 Report Number 2). *Invest Ophthalmol Vis Sci*. 2013;54:4548−4554.
32. SanGiovanni JP, Chew EY, Clemons TE, et al. The relationship of dietary lipid intake and age-related macular degeneration in a case-control study: AREDS Report No. 20. *Arch Ophthalmol*. 2007;125:671−679.
33. Michaud DS, Giovannucci EL, Ascherio A, et al. Associations of plasma carotenoid concentrations and dietary intake of specific carotenoids in samples of two prospective cohort studies using a new carotenoid database. *Cancer Epidemiol Biomarkers Prev*. 1998;7:283−290.
34. Rimm EB, Giovannucci EL, Stampfer MJ, et al. Reproducibility and validity of an expanded self-administered semi-quantitative food frequency questionnaire among male health professionals. *Am J Epidemiol*. 1992;135:1114−1126. discussion 1127−1136.
35. Lopez-Garcia E, Rodriguez-Artalejo F, Li TY, et al. The Mediterranean-style dietary pattern and mortality among men and women with cardiovascular disease. *Am J Clin Nutr*. 2014;99:172−180.
36. Fung TT, Rexrode KM, Mantzoros CS, et al. Mediterranean diet and incidence of and mortality from coronary heart disease and stroke in women. *Circulation*. 2009;119:1093−1100.
37. Liese AD, Krebs-Smith SM, Subar AF, et al. The Dietary Patterns Methods Project: synthesis of findings across cohorts and relevance to dietary guidance. *J Nutr*. 2015;145:393−402.
38. Fritsche LG, Igl W, Bailey JN, et al. A large genome-wide association study of age-related macular degeneration highlights contributions of rare and common variants. *Nat Genet*. 2016;48:134−143.
39. Kleinbaum DG, Klein M. The stratified Cox procedure. In: *Survival Analysis: A Self-Learning Text*. Third ed. New York: Springer; 2012:201−227.
40. Wei LJ, Lin DY, Weissfeld L. Regression analysis of multivariate incomplete failure time data by modeling marginal distribution. *J Am Stat Assoc*. 1989;84:1065−1073.
41. Trichopoulou A, Kouris-Blazos A, Wahlqvist ML, et al. Diet and overall survival in elderly people. *BMJ*. 1995;311: 1457−1460.
42. Trichopoulou A, Martinez-Gonzalez MA, Tong TY, et al. Definitions and potential health benefits of the Mediterranean diet: views from experts around the world. *BMC Med*. 2014;12:112.
43. Neale EP, Batterham MJ, Tapsell LC. Consumption of a healthy dietary pattern results in significant reductions in C-reactive protein levels in adults: a meta-analysis. *Nutr Res*. 2016;36:391−401.
44. Reedy J, Krebs-Smith SM, Miller PE, et al. Higher diet quality is associated with decreased risk of all-cause, cardiovascular disease, and cancer mortality among older adults. *J Nutr*. 2014;144:881−889.
45. National Institutes of Health Office of Dietary Supplements. Omega-3 fatty acids fact sheet for health professionals. [Internet]. 2019. https://ods.od.nih.gov/factsheets/Omega3-FattyAcids-HealthProfessional/. Accessed February 14, 2019.
46. Augood C, Chakravarthy U, Young I, et al. Oily fish consumption, dietary docosahexaenoic acid and eicosapentaenoic acid intakes, and associations with neovascular age-related macular degeneration. *Am J Clin Nutr*. 2008;88: 398−406.
47. Seddon JM, Rosner B, Sperduto RD, et al. Dietary fat and risk for advanced age-related macular degeneration. *Arch Ophthalmol*. 2001;119:1191−1199.
48. Chong EW, Kreis AJ, Wong TY, et al. Dietary omega-3 fatty acid and fish intake in the primary prevention of age-related macular degeneration: a systematic review and meta-analysis. *Arch Ophthalmol*. 2008;126:826−833.
49. Wong WL, Su X, Li X, et al. Global prevalence of age-related macular degeneration and disease burden projection for 2020 and 2040: a systematic review and meta-analysis. *Lancet Glob Health*. 2014;2:e106−e116.
50. Obata R, Yanagi Y, Inoue T, et al. Prevalence and factors associated with age-related macular degeneration in a southwestern island population of Japan: the Kumejima Study. *Br J Ophthalmol*. 2018;102:1047−1053.
51. Yasuda M, Kiyohara Y, Hata Y, et al. Nine-year incidence and risk factors for age-related macular degeneration in a defined Japanese population the Hisayama study. *Ophthalmology*. 2009;116:2135−2140.
52. Gopinath B, Flood VM, Rochtchina E, et al. Homocysteine, folate, vitamin B-12, and 10-y incidence of age-related macular degeneration. *Am J Clin Nutr*. 2013;98:129−135.
53. Christen WG, Glynn RJ, Chew EY, et al. Folic acid, pyridoxine, and cyanocobalamin combination treatment and age-related macular degeneration in women: the Women's Antioxidant and Folic Acid Cardiovascular Study. *Arch Intern Med*. 2009;169:335−341.
54. Do KV, Kautzmann MI, Jun B, et al. Elovanoids counteract oligomeric beta-amyloid-induced gene expression and protect photoreceptors. *Proc Natl Acad Sci U S A*. 2019;116: 24317−24325.

55. Zinkernagel MS, Zysset-Burri DC, Keller I, et al. Association of the intestinal microbiome with the development of neovascular age-related macular degeneration. *Sci Rep*. 2017;7:40826.
56. Rinninella E, Mele MC, Merendino N, et al. The role of diet, micronutrients and the gut microbiota in age-related macular degeneration: new perspectives from the Gut(-)Retina Axis. *Nutrients*. 2018;10.
57. Andriessen EM, Wilson AM, Mawambo G, et al. Gut microbiota influences pathological angiogenesis in obesity-driven choroidal neovascularization. *EMBO Mol Med*. 2016;8:1366−1379.
58. Kipnis V, Subar AF, Midthune D, et al. Structure of dietary measurement error: results of the OPEN biomarker study. *Am J Epidemiol*. 2003;158:14−26.
59. Subar AF, Kipnis V, Troiano RP, et al. Using intake biomarkers to evaluate the extent of dietary misreporting in a large sample of adults: the OPEN study. *Am J Epidemiol*. 2003;158:1−13.

Footnotes and Financial Disclosures

Originally received: January 6, 2020.
Final revision: April 16, 2020.
Accepted: April 20, 2020.
Available online: April 26, 2020. Manuscript no. D-20-00031.

[1] Division of Epidemiology and Clinical Applications, National Eye Institute, National Institutes of Health, Bethesda, Maryland.

[2] Department of Ophthalmology and Visual Sciences, University of Wisconsin School of Medicine and Public Health, Madison, Wisconsin.

[3] The Emmes Company LLC, Rockville, Maryland.

[4] Neurobiology-Neurodegeneration & Repair Laboratory, National Eye Institute, National Institutes of Health, Bethesda, Maryland.

Presented in part at: Association for Research in Vision and Ophthalmology Annual Meeting, Honolulu, Hawaii, April 29 to May 3, 2018.

*The AREDS and AREDS2 Research Groups appear online (available at www.aaojournal.org).

‡T.D.K. and E.A. contributed equally.

Financial Disclosure(s):

The author(s) have no proprietary or commercial interest in any materials discussed in this article.

Supported by intramural program funds and contracts (AREDS (Contract NOI-EY-0-2127) and AREDS2 (Contract HHS-N-260-2005-00007-C; ADB Contract N01-EY-5-0007) from the National Eye Institute/National Institutes of Health, Department of Health and Human Services, Bethesda, Maryland. Funds were generously contributed to these contracts by the following National Institutes of Health institutes: Office of Dietary Supplements; National Center for Complementary and Alternative Medicine; National Institute on Aging; National Heart, Lung, and Blood Institute; National Institute of Neurological Disorders and Stroke. The sponsor and funding organization participated in the design and conduct of the study, data collection, management, analysis, and interpretation, and preparation, review, and approval of the manuscript.

HUMAN SUBJECTS: Human subjects were included in this study. Institutional review board approval was obtained at each site and written informed consent was obtained from all participants. The research was conducted under the Declaration of Helsinki and, for AREDS2, complied with the Health Insurance Portability and Accessibility Act.

No animal subjects were used in this study.

Author Contributions:

Conception and design: Keenan, Mares, Clemons, Swaroop, Chew

Data collection: Agrón, Clemons, Chew

Analysis and interpretation: Keenan, Agrón, Mares, van Asten, Swaroop, Chew

Obtained funding: N/A; Study was performed as part of the authors regular employment duties. No additional funding was provided.

Overall responsibility: Keenan, Agrón, Mares, Clemons, van Asten, Swaroop, Chew

Abbreviations and Acronyms:

AMD = age-related macular degeneration; **aMedi** = modified Alternative Mediterranean Diet Index; **AREDS** = Age-Related Eye Disease Study; **BMI** = body mass index; **CI** = confidence interval; **DHA** = docosahexaenoic acid; **EPA** = eicosapentaenoic acid; **FFQ** = food frequency questionnaire; **GA** = geographic atrophy; **GRS** = Genetic Risk Score; **GWAS** = genome-wide association study; **MUFA** = monounsaturated fatty acid; **RCT** = randomized controlled trial; **SFA** = saturated fatty acid; **SNP** = single nucleotide polymorphism.

Correspondence:

Emily Y. Chew, MD, NIH, Building 10, CRC, Room 3-2531, 10 Center Drive, MSC 1204, Bethesda, MD 20892-1204. E-mail: echew@nei.nih.gov.
