# Supplementary material for: Insights into the nutritional prevention of macular degeneration based on a comparative topic modeling approach
Source: PeerJ Comput Sci. 2024 Mar 20;10:e1940. doi: 10.7717/peerj-cs.1940 (PMC11042009; doi:10.7717/peerj-cs.1940)
Supplement: Supplemental Information 2 [file peerj-cs-10-1940-s002.zip › positive_result_reports/new6.pdf]

Journal of the American College of Nutrition

ISSN: 0731-5724 (Print) 1541-1087 (Online) Journal homepage: https://www.tandfonline.com/loi/uacn20

The Potential Role of Dietary Antioxidant Capacity in Preventing Age-Related Macular Degeneration

Sedat Arslan, Sibel Kadayifçilar & Gülhan Samur

To cite this article: Sedat Arslan, Sibel Kadayifçilar & Gülhan Samur (2019) The Potential Role of Dietary Antioxidant Capacity in Preventing Age-Related Macular Degeneration, Journal of the American College of Nutrition, 38:5, 424-432, DOI: 10.1080/07315724.2018.1538830

To link to this article: https://doi.org/10.1080/07315724.2018.1538830

Published online: 20 Dec 2018.

Submit your article to this journal

Article views: 624

View related articles

View Crossmark data

Citing articles: 14 View citing articles

Full Terms & Conditions of access and use can be found at
https://www.tandfonline.com/action/journalInformation?journalCode=uacn21

JOURNAL OF THE AMERICAN COLLEGE OF NUTRITION
2019, VOL. 38, NO. 5, 424–432
https://doi.org/10.1080/07315724.2018.1538830

Taylor & Francis
Taylor & Francis Group

Check for updates

The Potential Role of Dietary Antioxidant Capacity in Preventing Age-Related Macular Degeneration

Sedat Arslan[a], Sibel Kadayifçilar[b], and Gülhan Samur[c]

[a]Nutrition and Dietetics, Kastamonu University, Kastamonu, Turkey; [b]Department of Ophthalmology, Hacettepe University, Ankara, Turkey; [c]Nutrition and Dietetics, Hacettepe University, Ankara, Turkey

ABSTRACT

Objectives: Age-related macular degeneration (AMD) is a progressive disorder among people aged ≥50 years. Some dietary factors associated with the susceptibility to AMD include dietary glycemic index and glycemic load, as well as intake of antioxidants and other nutrients, such as vitamins, minerals, and dietary fatty acids.

Methods: This case-control study was conducted between July 2015 and February 2016 on 100 case subjects with AMD and 100 healthy controls without AMD. The participants were recruited from the Department of Ophthalmology of Hacettepe University Hospitals in Ankara, Turkey. Dietary intake was estimated from a 3-day food intake record and food frequency questionnaire, and anthropometric measurements were recorded. The relationship between nutritional factors and AMD was assessed using logistic regression.

Results: Dietary total antioxidant intake of AMD group was found to be lower ($p < 0.05$) than that of healthy individuals. In a multivariate analysis, smoking, daily red meat intake, omega-6 intake, and higher glycemic index were identified as risk factors for AMD development. Meanwhile, daily fruit intake, daily fish intake, omega-3 intake, and zinc intake were associated with a protective effect. However, no difference was found in dietary total antioxidant capacity.

Conclusions: In this study, a high dietary intake of carotenoids, vitamins C and E, zinc, and omega-3, as well as maintaining optimal waist circumference; were found to substantially reduce the risk of developing AMD in people aged >50 years. By contrast, in addition to smoking and old age, obesity, high red meat intake, and omega-6 intake might increase the risk of developing AMD. Therefore, a better understanding of nutritional risk factors is necessary for preventing AMD.

ARTICLE HISTORY
Received 3 August 2018
Accepted 15 October 2018

KEYWORDS
Dietary total antioxidant capacity; nutrition; age-related macular degeneration; nutrient intake; glycemic index

Introduction

There is an increasing global concern about the issues related to eye health. In 2010, World Health Organization (WHO) defined the primary causes of visual impairment: uncorrected refractive errors (43%), cataract (33%), glaucoma (2%), age-related macular degeneration (AMD) (3%), diabetic retinopathy (1%), and undetermined natural reasons (18%) (1). This report also outlined the three major reasons for blindness: cataract (51%), glaucoma (8%), and AMD (5%) (1). AMD is a leading cause of progressive, bilateral blindness in the elderly, and it can be classified as non-exudative (dry) or exudative (wet) (2).

Nutrition plays a vital role in sustaining a healthy life. Researchers with the Age-Related Eye Disease Study (AREDS) reported in 2001 that a nutritional supplement called AREDS formulation can reduce the risk of developing advanced AMD. The original AREDS formulation contained vitamin C, vitamin E, beta-carotene, zinc, and copper (3). A subsequent study completed in 2012, AREDS-2, showed that removing beta-carotene and decreasing the zinc level in the formulation did not exacerbate the AMD progression rate. The study also showed that incorporating beta-carotene in a group of past smokers (who quit at least a year ago) led to a substantially high rate of lung cancer development. Hence, the researchers introduced carotenoids, namely, lutein and zeaxanthin, as substitutes to beta-carotene in the new formulation. Although lutein and zeaxanthin did not further decrease the risk of AMD progression, they were found to be safer and appropriate, especially for former smokers to mitigate the incidence of lung cancer (4).

The findings of some studies on the effect of increased carotenoid and antioxidant intake on the risk of AMD are inconsistent (5,6). Likewise, the epidemiological evidence between dietary fat intake and AMD is inconsistent (7). One study has shown that consuming fatty fish, which increases polyunsaturated fatty acid intake, decreases the risk of developing AMD (8). However, other studies have reported that a high rate of fat intake is a risk factor for AMD (6,9). Moreover, another study has found no significant relationship between dietary fat intake and AMD development after the correction of other variables (10). In addition, the

CONTACT Gülhan Samur gsamur@hacettepe.edu.tr Nutrition and Dietetics, Hacettepe University, Turkey: Hacettepe Üniversitesi Sıhhıye Kampüsü Sağlık Bilimleri Fakültesi, Beslenme ve Diyetetik Bölümü, Altındağ/Ankara, Turkey.
Color versions of one or more of the figures in the article can be found online at www.tandfonline.com/uacn.
© 2018 American College of Nutrition

interventional AREDS-2 study has reported that the additional intake of long-chain omega-3 polyunsaturated fatty acids does not confer any beneficial effect against AMD (6). Despite these disparities, a combination of proper dietary nutrition intake (e.g., vitamin, mineral, and supplement) and other precautionary strategies are consequential in the effort of preventing AMD progression, the risk of which compounds with age.

Some evidence in America has indicated that the likelihood of developing AMD is more closely associated with waist-to-hip ratio than with body mass index (BMI) (11). In a cohort of middle-aged patients, after a 6-year follow-up, a group of researchers reported that the decrease of waist-to-hip ratio reduced the risk of AMD; the same trend was observed for waist circumference reduction, although the evidence was weak (12). Similarly, another study has reported that the increase in waist-to-hip ratio or waist circumference increases AMD progression (13).

This study aimed to evaluate dietary total antioxidant capacity, food and nutrient intake, and some anthropometric measurements of individuals with AMD. The possible effect of nutritional factors on the occurrence of AMD was evaluated by comparing the dietary total antioxidant capacity and some anthropometric measurements of healthy individuals with those of individuals with AMD.

Materials and methods

Participants

This was a descriptive and cross-sectional study including case and control groups. It was conducted in the Department of Ophthalmology of Hacettepe University Hospitals in Ankara, Turkey, between July 2015 and February 2016. The research sample comprised volunteers aged >50 years who met the study criteria.

While benefiting from the results of the previous studies, the sample size of the research was type 1 error level $\alpha = 0.05$ and type 2 level $\beta = 0.20$. The power of the test was taken as $1 - \beta = 0.80$, and the power analysis was statistically calculated using NCCS PAS 11 program. Two hundred individuals voluntarily participated in this study: 100 case patients (50 women, 50 men) and 100 control patients (50 women, 50 men). Controls were age matched with the cases and had general status similar to the cases. Individuals aged <50 years or who had special nutrition conditions, cardiovascular diseases, or eye surgery history were excluded.

A written informed consent was obtained from every participant. The proposed research study was evaluated and ethically approved on July 10, 2015, by the Hacettepe University Clinical Research Ethical Board with project number GO 15/628 and decision number GO 15/628-23.

Data on demographics and dietary intake of different food groups were collected from standardized face-to-face interviewer-assisted questionnaires. The sociodemographic attributes of the individuals (gender, age, occupation, educational status, and health conditions) were recorded, and additional information on whether they smoke or use drugs was confidentially obtained.

Body weight of each participant was measured using a calibrated electronic scale with an accuracy of 0.1 kg (TANITA HD 366) and conducted when the patient was hungry, wearing lightweight clothes and no shoes. Body height was measured by a researcher (S.A.) using a tape measure when each participant was standing upright in the Frankfurt Plane position (i.e., ear canal and the lower margin of the orbit are horizontal while facing forward) (14). BMI was calculated using the formula body weight (kg)/body length(2) (m^2) and categorized based on the WHO classification (15). According to this classification, a BMI of <18.5 kg/m^2 is thin, 18.5–24.9 kg/m^2 is normal, 25.0–29.9 kg/m^2 is overweight, and ≥30 kg/m^2 is obese (15). Waist circumference was also measured using a tape measure by circling the waist at the midpoint between the lowest rib and the umbilicus. If the waist circumference is found to be ≥88 cm for women and ≥102 cm for men, this is indicative of high risk of developing chronic diseases (16). Hip circumference was subsequently measured using a tape measure at the highest point of the hip while standing at the left side of the individual. If the waist-to-hip ratio is ≥0.85 for women and ≥0.90 for men, the risk of developing chronic diseases increases (16).

Assessment of dietary intake and dietary total antioxidant capacity

The dietary intake of participants was assessed using a validated quantitative food frequency questionnaire (QFFQ) (17). The QFFQ was designed to measure a "typical" diet by asking questions about the frequency and amount of food consumption. It comprised 110 food items. Foods were classified into the following categories: milk and dairy products, meat and meat products, fruits, vegetables, bread and cereals, beverages, and desserts. Intake frequencies included eight categories, ranging from never/once a month to more than one per day. Trained interviewers inquired how often the participants had consumed one portion of each food item. To compute the total amount of food intake per day, the reported frequency of consumption for each food item was multiplied by the portion size. The total food intake was then converted to total nutrient intake based on the food's nutrient profile. Standardized food recipes for Turkey (18) and the Nutrition Information System (BEBIS) program, (19) which is a food composition database for nutrient estimation, were used to determine the average daily energy and nutrient intake for each participant. These values were subsequently compared with the recommended daily allowance values (20) to determine the status of meeting energy and nutrient requirements. Thereafter, the percentages meeting the requirements were calculated.

Meanwhile, to assess dietary antioxidant capacity from the QFFQ, each food's contribution to Ferric Reducing Antioxidant Power (FRAP) was calculated based on the Antioxidant Food Table published by the Institute of Nutrition Research at the University of Oslo, which comprises data on >3,000 food items (21).

Table 1. General Characteristics and Statistical Information of Participants.

	Case group ($n = 100$)		Control group ($n = 100$)		Total ($n = 200$)		
Sociodemographic characteristics	n	%	n	%	N	%	p
Gender							
Female	50	50.0	50	50.0	100	50.0	
Male	50	50.0	50	50.0	100	50.0	
Age groups (years)							
50–65	38	38.0	39	39.0	77	38.5	0.884[a]
≥65	62	62.0	61	61.0	123	61.5	
Age (mean) (years)	68.1 ± 8.4		67.3 ± 8.5		67.7 ± 8.4		0.503[b]
Marital status							0.020[a]
Married	74	74.0	87	87.0	161	80.5	
Single/divorced/widowed	26	26.0	13	13.0	39	19.5	
Education							
<High school	65	65.0	75	75.0	140	70.0	0.123[a]
≥High school	35	35.0	25	25.0	60	30.0	
Total education period (mean; years)	7.0 ± 4.9		5.3 ± 4.7		6.2 ± 4.9		0.012[c]
Occupation							
Retired	85	85.0	78	78.0	163	81.5	0.202[a]
Still working	15	15.0	22	22.0	37	18.5	
Smoking status							
Ever	54	54.0	24	24.0	78	39.0	0.000[a]
Never	46	46.0	76	76.0	122	61.0	
Smoking period (mean; years)	30.6 ± 14.6		29.3 ± 12.2		30.2 ± 13.9		0.673[b]
Body mass index (BMI; $\bar{X}$ ± SD) (kg/m^2)							
Female BMI	30.4 ± 4.9		27.8 ± 4.3		29.1 ± 4.8		0.005[b]
Male BMI	27.8 ± 2.9		26.0 ± 3.5		26.9 ± 3.3		0.006[b]
Waist circumference (WC; $\bar{X}$ ± SD) (cm)							
Female WC	100.2 ± 9.7		96.5 ± 10.0		98.4 ± 9.9		0.066[b]
Male WC	99.2 ± 9.1		94.0 ± 11.7		96.6 ± 10.7		0.016[b]
Waist-to-hip ratio (W/H; $\bar{X}$ ± SD) (cm)							
Female W/H	0.95 ± 0.08		0.94 ± 0.05		0.94 ± 0.06		0.354[c]
Male W/H	0.99 ± 0.06		0.98 ± 0.06		0.99 ± 0.61		0.257[c]

[a]Pearson chi-squared test.
[b]Independent two-sample t-test.
[c]Mann–Whitney U test ($p < 0.05$).

Statistical analysis

For statistical evaluation of data obtained in this study, the Statistical Package for Social Science 22.0 program (IBM, USA) was used. The average, standard deviation, lowest value, and highest value of the data set collected were computed. The data distribution by numbers was given as number–percentage tables. For evaluation of the continuous data, the Kolmogorov–Smirnov test was used to determine whether the set followed a normal distribution. The normally distributed data were analyzed using parametric statistical tests (independent two-sample t-test and Pearson correlation), whereas nonnormally distributed data were analyzed using nonparametric statistical tests (Pearson chi-squared, Mann–Whitney U, and Spearman correlation). Odds ratios (OR) and 95% confidence intervals (CI) were calculated using a univariate logistic regression analysis independently for each criterion (food group, BMI, adjusted age, and gender) before combining them in a multivariate logistic regression. The OR and 95% CI values were then used to determine the risk factors for developing AMD. Furthermore, a stepwise multivariate regression analysis was also performed, with strict inclusions of the set with $p < 0.05$ in the model.

Results

A summary of the study population's characteristics and statistical information is shown in Table 1. The age distribution of the participants was found to be 38.5% in the 50–65 years age group and 61.5% in the ≥65 eyars age group ($p > 0.05$). The average age of the case group was 68.09 ± 8.41 years and that of the control group was 67.29 ± 8.45 years, with no statistically significant difference between the groups ($p > 0.05$). However, a statistically significant difference was noted between these two groups in terms of marital status ($p < 0.05$). The average education period in the case group (7.0 ± 4.9 years) was significantly higher than that in the control group (5.3 ± 4.7 years; $p < 0.05$). No statistically significant difference was found between the two groups in terms of occupation ($p > 0.05$).

The case group comprised 54.0% smokers and 46.0% nonsmokers, whereas the control group comprised 24.0% smokers and 76.0% nonsmokers. The two groups were statistically different in terms of smoking, and the smoking period was higher in the case group than in the control group ($p < 0.001$).

In the study, 66.0% of women had wet-type AMD and 34.0% had dry-type AMD. Meanwhile, 74.0% men had wet-type AMD and 26.0% had dry-type AMD. In total, 70.0% of participants with AMD had wet-type AMD and 30.0% had dry-type AMD. In this study, the average time for AMD diagnosis was 4.7 ± 2.8 years in women and 5.3 ± 2.2 years in men in the case group.

An overview of the anthropometric measures is given in Table 1. The average BMI in the case group was significantly higher than that in the control group ($p < 0.001$). Further,

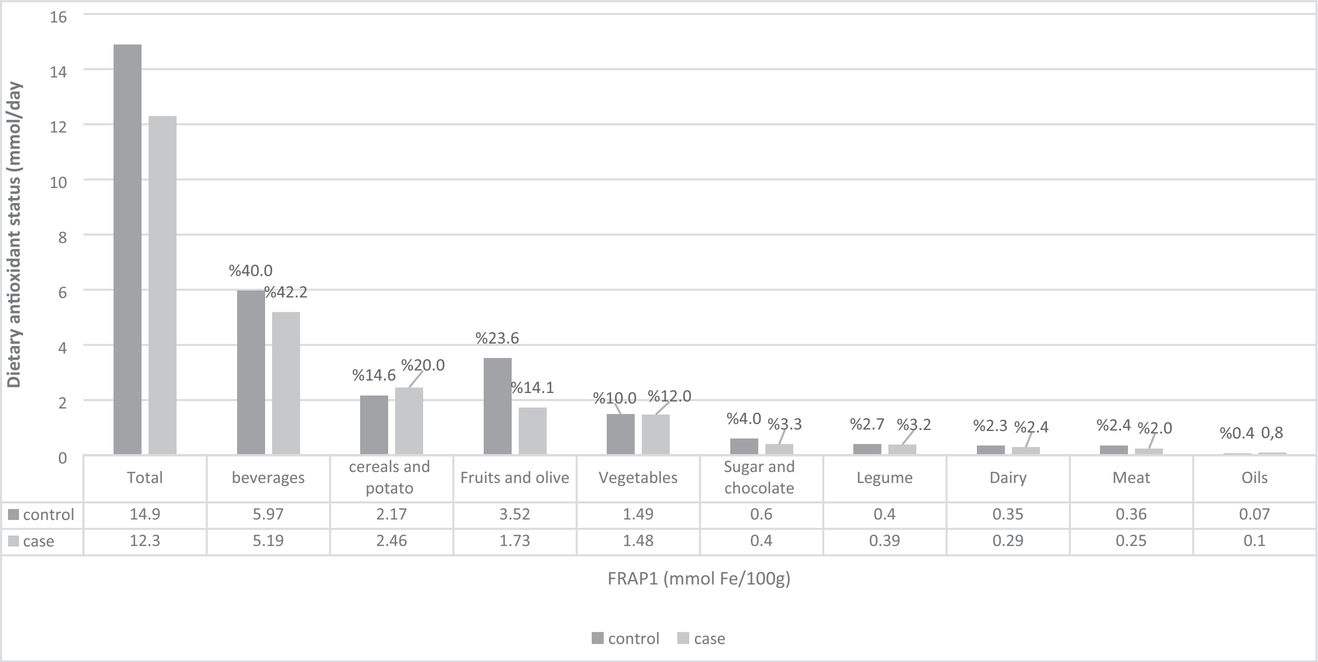

Figure 1. The dietary total antioxidant capacity and contribution of each nutrient groups to dietary total antioxidant capacity (dTAC) values calculated by Ferric Reducing Antioxidant Power (FRAP1) (Carlsen et al.) (% and mmol/d).

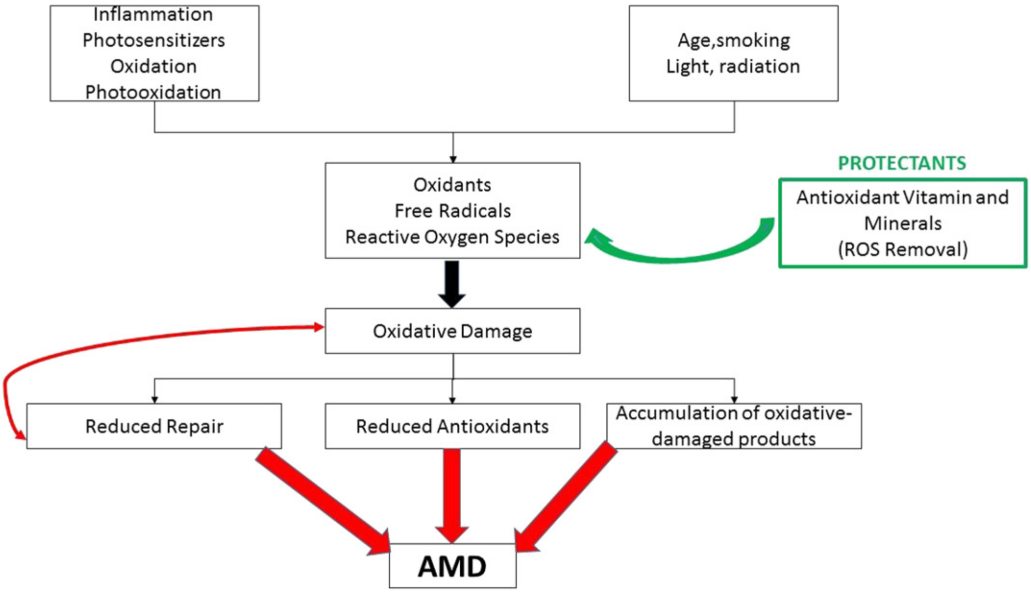

Figure 2. Schematic representation of development of AMD and protective mechanism of antioxidants. AMD: Age-related macular degeneration.

the average waist circumference was significantly higher in the case group than in the control groups, particularly among the men ($p < 0.05$). Meanwhile, no statistically significant difference was observed between the two groups in terms of waist-to-hip ratio ($p > 0.05$).

Based on the WHO's BMI classification, (15) in the case group, 12.0% participants were normal, 55.0% were slightly obese, 28.0% were class I obese, 3.0% were class II obese, and 2.0% were class III obese. Furthermore, in the control group, 25.0% participants were normal, 54.0% were slightly obese, 17.0% were class I obese, 2.0% were class II obese, and 1.0% were class III obese. Thus, no statistically significant difference was found between the control and case groups in terms of the BMI distributions ($p > 0.05$). The average BMI was 30.4 ± 4.9 in women and 27.8 ± 2.9 in men in the case group and 29.1 ± 4.8 in women and 26.9 ± 3.3 in men in the control group, indicating a statistically significant difference between the groups ($p < 0.05$).

Similarly, the waist circumference was analyzed according to WHO guideline (16). Both groups had a significant portion of participants in the AMD-risky category: 69.0% in the case group and 54.0% in the control group, but the difference was statistically significant between the two groups ($p < 0.05$). Meanwhile, the evaluation of waist-to-hip ratio showed that 67.0% of participants in the case group and 62% in the control group were in the AMD-risky category,

Table 2. Relationships Between Dietary Total Antioxidant Capacity and Anthropometric Measurements.

	Dietary total antioxidant capacity			
	Case group		Control group	
	r	*p*	*r*	*p*
Body mass index (kg/m^2)	0.058	0.569[a]	0.222	**0.027**[a]*
Waist circumference	0.072	0.478[b]	0.370	**0.000**[b]**
Waist-to-hip ratio	0.035	0.731[b]	0.044	0.661[a]

Note. Boldface data show significant difference.
[a]Spearman correlation.
[b]Pearson correlation.
*$p < 0.05$.
**$p < 0.001$.

indicating no statistically significant difference between the groups ($p > 0.05$).

The mean and 95% CI of dietary antioxidant status for each nutrient group in both case and control groups are shown in Figure 1. The antioxidant capacity levels from the nutrients obtained from fish and water products, egg and egg products, fresh legumes, dry legumes, Mediterranean fruits, olives, vegetables, white bread, whole-wheat bread, cake, pasta, cookies, coffee, tea, herbal tea, wine and similar drinks, sugar, honey, and candies were not statistically different between the case and control groups ($p > 0.05$). Meanwhile, the antioxidant capacity levels from the nutrient groups of fruits and olive and beverages were significantly higher in the control group than in the case group ($p < 0.05$): 1.7 ± 0.9 and 5.2 ± 2.9 mmol/d, respectively, in the case group and 3.5 ± 1.7 and 5.9 ± 4.0 mmol/d, respectively, in the control group. The antioxidant capacity levels from oils, cereals, and potatoes were significantly lower in the control group than in the case group ($p < 0.05$). However, the antioxidant capacity levels from meat, dairy, legume, sugar, and chocolate products were not significantly different between the groups ($p > 0.05$).

The approximate dietary total antioxidant capacity levels were determined based on the food intake records. The dietary total antioxidant capacity was statistically higher in the in the control group (14.9 ± 5.0 mmol/d) than in the case group (12.3 ± 3.4 mmol/d; $p < 0.05$; Figure 1).

In Table 2, the relationships between dietary total antioxidant capacity and anthropometric measures (i.e., BMI, waist circumference, and waist-to-hip ratio) are summarized. No significant relationship was found between the dietary total antioxidant capacity and any anthropometric measure in the case group ($p > 0.05$). However, the relationship between dietary total antioxidant capacity and BMI in the control group was an "unimportant correlation" ($p < 0.05$). Moreover, the relationship of dietary total antioxidant capacity with waist circumference in the control group was a "mid-level correlation" ($p < 0.001$).

Table 3 presents the results of logistic regression analysis of dietary antioxidant capacity, energy and nutrient intake, dietary glycemic index/load, and some risk factors for AMD in both case and control groups. It was found that the individuals who consumed fruits every day (OR: 0.56, $p < 0.05$) or 3–5 times in a week (OR: 0.75; $p < 0.05$) and those who consumed vegetables every day (OR: 0.80, $p > 0.05$) or 3–5 times in a week (OR: 0.96, $p > 0.05$) had a low risk of developing AMD. Similarly, the likelihood of developing AMD was lower in individuals who consumed fish every day (OR: 0.35, $p < 0.05$) or 3–5 times in a week (OR: 0.68, $p < 0.05$) than in those who consumed it only once in a week or less often. By contrast, the participants who consumed red meat every day had 1.2 times higher risk of developing AMD than the individuals who consumed it once in a week or less often ($p < 0.05$). No difference was observed in the risk of developing AMD between individuals

Table 3. Univariate Binary Logistic Regression Analysis for AMD Versus No AMD, Adjusted for Age and Gender.

	OR	95% CI	*p*
Energy	1.01	1.0–1.02	**0.015**
Protein	0.96	0.88–1.05	0.297
Carbohydrates	1.37	1.13–1.62	**0.030**
Fiber	0.96	0.76–1.10	0.694
Fat	1.41	1.09–1.81	**0.009**
Omega-3	0.12	0.04–0.35	**0.000**
Omega-6	1.65	1.35–2.0	**0.000**
Vitamin A	1.0	0.99–1.0	**0.011**
Lutein + zeaxantin	1.0	0.99–1.0	**0.003**
Carotenoids	0.70	0.61–0.93	**0.036**
Vitamin C	0.97	0.80–1.03	**0.000**
Vitamin E	0.76	0.63–0.88	0.130
Vitamin B_{12}	0.87	0.48–1.10	**0.017**
Folic acid	0.99	0.98–1.05	0.583
Vitamin B_6	0.77	0.55–0.91	0.124
Zinc	0.38	0.20–0.50	**0.002**
Selenium	1.49	0.22–98.3	0.853
Glycemic index			
<70*			0.371
≥70	2.03	0.30–2.21	**0.040**
Glycemic load			
<120			0.145
≥120	2.76	0.43–17.87	0.286
Dietary antioxidant capacity	1.0	0.99–1.00	0.094
Fruit intake			
Everyday	0.56	0.40–0.76	**0.028**
3–5 times a week	0.75	0.60–0.93	**0.020**
<Once a week*			0.183
Vegetable intake			
Everyday	0.80	0.71–1.01	0.060
3–5 times a week	0.96	0.92–0.99	0.125
<Once a week*			0.415
Fish intake			
Everyday	0.35	0.2–0.50	**0.010**
3–5 times a week	0.68	0.55–1.00	**0.002**
<Once a week*			0.083
Red meat intake			
Everyday	1.2	0.80–1.40	**0.042**
3–5 times a week	1.0	0.97–1.00	0.315
<Once a week			0.615
Tobacco use			
Non-smoker*			**0.019**
Smoker	6.59	1.34–32.5	**0.021**
Given up	1.48	0.2–11.3	0.703
Body mass index (BMI) (kg/m^2)			
18.5–24.9 (normal)*			1.000
25.0–29.9 (Overweight)	6.46	0.03–17.36	0.213
30.0–34.9 (Obese class I)	3.37	0.01–7.26	0.295
35.0–39.9 (Obese class II)	15.2	0.1–23.26	0.142
>40 (Obese class III)	25.24	0.02–32.236	0.233
Waist circumference			
Female <88 (Normal)*			0.186
≥88 (Risky)	1.09	0.99–1.20	**0.03**
Male <102 (Normal)*			0.04
≥102 (Risky)	1.12	1.06–1.30	**0.012**

Note. Boldface data show significant difference. OR: odds ratio; CI: confidence interval.
*Because the "reference" category for the categorical variables was the first category, their values were not calculated.

who consumed red meat 3–5 times in a week and those who consumed less (OR: 1.0, $p > 0.05$).

Compared with the disease risk of individuals with normal BMI, slightly obese people had 6.46 times higher risk, class I obese people had 3.37 times higher risk, class II obese people had 15.2 times higher risk, and class III obese people had 25.24 times higher risk; however, these results were not statistically significant ($p > 0.05$). Furthermore, compared with healthy women, women who were classified at risk in relation to the waist circumference analysis had 1.09 times higher risk of developing AMD ($p < 0.05$). Similarly, compared with healthy men, men who were at risk in terms of waist circumference had 1.12 times higher risk of developing AMD ($p < 0.05$). Meanwhile, participants who consumed high glycemic index and glycemic load diet had 2.3 times ($p < 0.05$) and 2.76 times ($p > 0.05$) higher risk of developing AMD, respectively. Nonetheless, no significant association was found between dietary total antioxidant capacity and the likelihood of AMD development (OR: 1.0, $p > 0.05$).

Discussion

Age is the primary risk factor for AMD development. According to the International Classification System, 50 years was the minimum age for investigating drusen occurrence and other abnormalities for diagnosing AMD (22). In a meta-analysis, a definite correlation was found between increasing age and AMD in all studies (23). Although AMD incidence increases by 0.21% in the 55–64 years age group and by 0.85% in the 60–74 years age group, it substantially increases by 4.59% in the 75–84 years age group and by 13.05% in the ≥85 years age group (24). In this study, by eliminating age as the risk factor, the average age values between the groups were found to be similar; the average age of AMD patients was 68.09 ± 8.41 years.

The socioeconomic conditions and education have not yet been determined as risk factors for AMD (25). In the Beaver Dam Eye Study, no correlation was noted between income level, education level, marital status, and AMD development (26). However, our study discovered a statistically significant difference between the case and control groups in terms of marital status ($p = 0.042$). Nevertheless, this could be because of the small number of participants, only two of whom were single in the case group and none in the control group.

Although the pathophysiological mechanism between obesity and AMD is still unclear, researchers have hypothesized that both systematic oxidative stress and hyperleptinemia cause AMD (27,28). Moreover, the relationship between AMD and BMI has been analyzed in several population-based studies, which have produced inconsistent results. In the Blue Mountains Eye Study (BMES) and Physicians Health Study research, the risk of developing early dry-type AMD was increased in the individuals who did not have normal BMI (29,30). In the POLA study conducted in France, comparison between obese individuals (BMI ≥30 kg/m^2) and thin individuals revealed a relationship between wet-type AMD and pigment abnormalities (31). Although the Beaver Dam Eye Study reported a connection between early development of AMD and BMI in women, no such relationship was found in men (30). At the same time, the AREDS cohort study reported a correlation between high BMI and the prevalence of wet-type AMD (32). In a case-control study of 130 participants in Iran, no statistical difference in BMI was observed between the groups (33). In another POLA study (34), the risk of developing AMD was 2.29 times higher in obese individuals than in normal individuals. In addition, the Blue Mountains Eye Study (35) showed that this risk was 1.78 times higher for early AMD and 1.92 times higher for late AMD. Meanwhile, our results, which are consistent with the previous findings, showed that the average BMI was considerably higher in the case group than in the control group ($p < 0.001$). Compared with the disease risk of the individuals with normal BMI, slightly obese people had 6.46 times higher risk, class I obese people had 3.37 times higher risk, class II obese people had 15.2 times higher risk, and class III obese people had 25.24 times higher risk; however, these results were not statistically significant ($p > 0.05$).

In the study by Seddon (36) wherein 1198 patients were examined for an average of 4.6 years, it was found that high waist circumference doubled the progression rate of the disease, whereas high waist-to-hip ratio increased it by 1.84 times. Furthermore, in our study, the average waist and hip circumferences were significantly higher in the control group than in the case group ($p < 0.05$). However, the waist-to-hip circumference ratio was not significantly different between the two groups ($p > 0.05$). Moreover, the results of logistic regression analysis revealed that in terms of waist circumference, the women who were categorized at risk had 1.09 times higher risk of developing AMD than healthy women ($p < 0.05$). Similarly, the men who were categorized as at risk had 1.12 times higher risk of developing AMD than the healthy men ($p < 0.05$).

In another study, fruits were shown to contribute to a lower risk of developing AMD, particularly in the individuals who consumed 3 or more portions daily compared with those who consumed 1.5 portions or less. The study reported a similar trend in men and women in terms of vegetable intake, although the difference was not significant (37). Similarly, our study revealed that consuming more fruits and vegetables lowered the risk of developing AMD in both men and women. The individuals in the case group who consumed fruits every day or 3–5 times in a week as well as those who consumed vegetables every day or 3–5 times in a week had a low risk of developing AMD. Moreover, a daily intake of pome fruits, citrus fruits, and green leafy vegetables proved to be beneficial in lowering the disease risk in individuals in the case group.

Zinc and copper are essential trace metals that are packed in the photoreceptors and the retinal pigment epithelium (RPE) of the human eye. Zinc and copper work as cofactors in a few visual chemicals, including copper-zinc superoxide dismutase, a part of the primary antioxidant system used to regulate oxidative stress. Oxidative stress and decreased antioxidant capacity have been implicated in the pathogenesis and progression of several age-related disorders, including

age-related macular degeneration (AMD); however, no definitive link has been established. The retina and the RPE are especially vulnerable to oxidative stress as a result of raised oxygen tension, high polyunsaturated lipid substance, and high exposure to sun and light. Although there is no reliable systemic index of intracellular zinc and copper status, several lines of evidence suggest that an accumulated zinc and copper deficiency in ocular tissue is involved in AMD, possibly by increasing oxidative stress (Figure 2). Our results show that individuals with higher intake of zinc have lower risk of developing AMD (OR: 0.20–0.50, $p < 0.05$).

For a long time, taking dietary antioxidants has been recommended because they are helpful in preventing AMD development and progression. However, in one study, a diet consisting of A-C-E vitamins, zinc, lutein, zeaxanthin, alpha-carotene, beta-carotene, lycopene, and beta-cryptoxanthin was analyzed, which revealed that apart from vitamin E (OR: 0.83, 95% CI: 0.69–1.01), other nutrients had minimal effect (OR: 0.91–1.11) or no effect in AMD prevention (38). Meanwhile, our results, which are consistent with the AREDS results, showed a clear difference in dietary total antioxidant capacity between the case and control groups, wherein the latter exhibited higher capacity ($p < 0.001$). The dietary total antioxidant capacity was 12.3 ± 3.4 mmol/d in the case group and 14.9 ± 5.0 mmol/d in the control group. In addition, the relationship between dietary total antioxidant capacity and waist circumference in the control group was a "positively low and mid-level correlation" ($p < 0.001$) (Table 2). Based on that, further work is needed to better understand the relationship between waist circumference and dietary total antioxidant capacity in the development of AMD.

Lipids and lipid metabolites have for some time been known to playing biological roles that go past energy store and membrane structure. In AMD, dysregulation of lipid digestion is nearly connected with malady beginning and progression. The points of lipoprotein adjustment and omega-3 unsaturated fat admission get extraordinary consideration. Omega-6 and omega-3 are polyunsaturated unsaturated fats and they are fundamental in light of the fact that our living being can't deliver them and we must get them from sustenance. More often than not while humans have been on earth we have eaten sustenance containing an omega-6/omega-3 proportion of around 1– 2:1. Be that as it may, in the course of the most recent 50 years in Western eating routine, the proportion has changed to 10–20:1 (39). Our eating routine presently incorporates colossal measures of oils that are separated from plants and utilized for cooking or in arranged nourishment. These oils (e.g., sunflower and corn oil) are principally omega-6. Omega-3 is found in fatty fish, and in entire grains and beans. In this way, today we have exorbitant measures of omega-6 unsaturated fats contrasted and eat fewer carbohydrates, on which individuals involved and their hereditary examples were built up. Intemperate measures of omega-6 and a high omega-6/omega-3 proportion, as is found in the present Western diet, advance the pathogenesis of numerous endless ailments, including cardiovascular, malignancy, and immune system sicknesses, while expanded levels of omega-3 apply a suppressive impact. In our organism, omega-6 has proinflammatory and omega-3 anti-inflammatory impact. It is conceivable that ideal proportion may differ with the disease underthought. AMD is likewise a chronic illness, and it is multigenic and multifactorial. In our study we found that the omega-6/omega 3 ratio was 1:7 in the AMD group and 1:12 in the control group. Moreover, the results of logistic regression analysis revealed that in terms of higher omega-6 intake, the individuals who were categorized at risk had 1.65 times higher risk of developing AMD ($p < 0.05$). Contrary, in terms of higher omega-3 intake, individuals who were categorized as at risk had 0.12 times lower risk of developing AMD ($p < 0.05$).

A dietary constituent that may have remediating consequences for chronic illness is the polyunsaturated fatty acids (PUFA) in the omega-3 (n-3) class. Supplementation with the long-chain n-3 PUFA has appeared to diminish insulin obstruction, triglyceride levels, pulse, and circulatory strain, and increment HDL cholesterol levels. On the other hand, omega-6 (n-6) PUFA, which contend with n-3s for a few physiological procedures and are copious in the Western eating regimen, can increment inflammatory signals and have been related with AMD. Given the many restricting impacts of n-3s contrasted with n-6s, recent examinations have started to examine how the dietary proportions of these PUFA impact health and illness. While numerous n-6 inferred eicosanoids engender inflammatory signals, numerous n-3 determined eicosanoids are less incendiary and even mitigating by rivalry. Therefore, diets with higher n-6 to n-3 ratios may contribute to the pathology of metabolic syndrome through inflammatory processes and other currently unrecognized mechanisms. There should be attention to the fact that the connection between n-3 and n-6 PUFA is unpredictable and they are not generally in restriction. For instance, the n-6 determined lipoxins have anti-inflammatory impacts. In any case, the dietary-affected tissue proportions of n-3 to n-6 PUFA are vital in their connection to health and illness.

Our study concluded that BMI waist and hip circumferences of the individuals with AMD were higher and dietary total antioxidant intake was lower than those of healthy individuals. AMD is a multifactorial disorder, yet nutritional intake is one of the most modifiable risk factors. Our study demonstrated that excess body weight might act as a potential risk factor for AMD development. With the increasing prevalence of obesity, it is important to realize that an effort for reducing the risk of AMD development in terms of controlling body weight, especially in obese patients, can be an additional benefit.

Transparency declaration

The lead author affirms that this article is an honest, accurate, and transparent account of the study being reported. The reporting of this work is compliant with STROBE guidelines. The lead author affirms that no important aspects of the study have been omitted and that any discrepancies from the study as planned have been explained.

Ethics statement

The proposed research study was evaluated and ethically approved on July 10, 2015, by the Hacettepe University Clinical Research Ethical Board with project number GO 15/628, decision number GO 15/628-23, and clinical trials registration number NCT03326401. Data collection from the patients followed ethical standards by obtaining their written informed consent.

Conflicts of interest

The authors declare that they have no conflict of interests.

ORCID

Sedat Arslan http://orcid.org/0000-0002-3356-7332

References

1. Mariotti SP. Global data on visual impairments 2010. Germany: World Health Organization. 2012;20.
2. Zampatti S, Ricci F, Cusumano A, Marsella LT, Novelli G, Giardina E. Review of nutrient actions on age-related macular degeneration. Nutrit Res. 2014;34(2):95–105.
3. Group A-REDSR. Potential public health impact of Age-Related Eye Disease Study results: AREDS report no. 11. Arch Ophthalmol. 2003;121(11):1621.
4. Chew EY, Clemons T, SanGiovanni JP, Danis R, Domalpally A, McBee W, Sperduto R, Ferris FL. The Age-Related Eye Disease Study 2 (AREDS2): study design and baseline characteristics (AREDS2 report number 1). Ophthalmology. 2012;119(11): 2282–2289.
5. Sin HP, Liu DT, Lam DS. Lifestyle modification, nutritional and vitamins supplements for age-related macular degeneration. Acta Ophthalmol. 2013;91(1):6–11.
6. Chew EY, Clemons TE, SanGiovanni JP, Danis R, Ferris FL, Elman M, et al. Lutein + zeaxanthin and omega-3 fatty acids for age-related macular degeneration. JAMA. 2013;309(19): 2005–2015.
7. Weikel KA, Chiu CJ, Taylor A. Nutritional modulation of age-related macular degeneration. Mol Asp Medicine. 2012;33(4): 318–375.
8. SanGiovanni JP, Chew EY, Agron E, Clemons TE, Ferris FL, Gensler G, et al. The relationship of dietary ω-3 long-chain polyunsaturated fatty acid intake with incident age-related macular degeneration: AREDS Report No. 23. Arch Ophthalmol. 2008; 126(9):1274–1279.
9. Swenor BK, Bressler S, Caulfield L, West SK. The impact of fish and shellfish consumption on age-related macular degeneration. Ophthalmol. 2010;117(12):2395–2401.
10. Heuberger RA, Mares-Perlman JA, Klein R, Klein BE, Millen AE, Palta M. Relationship of dietary fat to age-related maculopathy in the Third National Health and Nutrition Examination Survey. Arch Ophthalmol. 2001;119(12):1833–1838.
11. Peeters A, Magliano DJ, Stevens J, Duncan BB, Klein R, Wong TY. Changes in abdominal obesity and age-related macular degeneration: the Atherosclerosis Risk in Communities Study. Arch Ophthalmol. 2008;126(11):1554–1560.
12. Seddon JM, Cote J, Rosner B. Progression of age-related macular degeneration: association with dietary fat, transunsaturated fat, nuts, and fish intake. Arch Ophthalmol. 2003;121(12):1728–1737.
13. Adams MKM, Simpson JA, Aung KZ, Makeyeva GA, Giles GG, English DR, Hopper J, Guymer RH, Baird PN, Robman LD, et al. Abdominal obesity and age-related macular degeneration. Am J Epidemiol. 2011;173((11):1246–1255.
14. Pekcan G, ve diğ. Beslenme durumunun saptanması. Ayşe Baysal. (Ed.) Diyet El Kitabı (Yenilenmiş 5. Baskı, s. 67–141). Ankara: Hatipoğlu Yayınevi; 2008.
15. World Health Organization. BMI classification 2004. Available from: http://www.euro.who.int/en/health-topics/disease-prevention/nutrition/a-healthy-lifestyle/body-mass-index-bmi.
16. Organization WH. Waist circumference and waist-hip ratio: Report of a WHO expert consultation, Geneva, 8–11 December 2008. 2011.
17. Marventano S, Mistretta A, Platania A, Galvano F, Grosso G. Reliability and relative validity of a food frequency questionnaire for Italian adults living in Sicily, Southern Italy. Int J Food Sci Nutr. 2016;67(7):857–864.
18. Merdol T. Standart Yemek Tarifeleri (3. bs.). Ankara: Hatipoğlu Yayınevi; 2003.
19. Schmid M. Beslenme Bilgi Sistemi BeBİS 7.0 paket programı. Entwickelt an der Universital Hohenheim, Stuttgart. 2011.
20. Dietary Guidelines For Turkey. Dietary Guidelines For Turkey. In: Dietetics HUDoNa, editor. Ankara. 2014.
21. Carlsen MH, Halvorsen BL, Holte K, Bøhn SK, Dragland S, Sampson L, et al. The total antioxidant content of more than 3100 foods, beverages, spices, herbs and supplements used worldwide. Nutrit J. 2010;9(1):1.
22. Bird AC, Bressler NM, Bressler SB, Chisholm IH, Coscas G, Davis MD, de Jong PT, Klaver CC, Klein BE, Klein R, et al. An international classification and grading system for age-related maculopathy and age-related macular degeneration. The International ARM Epidemiological Study Group. Surv Ophthalmol. 1995;39(5):367–374.
23. Chakravarthy U, Wong TY, Fletcher A, Piault E, Evans C, Zlateva G, et al. Clinical risk factors for age-related macular degeneration: a systematic review and meta-analysis. BMC Ophthalmol. 2010;10(1):1.
24. van Leeuwen R, Klaver CC, Vingerling JR, Hofman A, de Jong PT. REVIEW: Epidemiology of age-related maculopathy: a review. Eur J Epidemiol. 2003;18(9):845–854.
25. Group EDC-CS. Risk factors for neovascular age-related macular degeneration. Arch Ophthalmol. 1992;110(12):1701.
26. Klein R, Klein BE, Jensen SC, Moss SE, Cruickshanks KJ. The relation of socioeconomic factors to age-related cataract, maculopathy, and impaired vision. The Beaver Dam Eye Study. Ophthalmol. 1994;101(12):1969–1979.
27. Suter PM, Locher R, Häsler E, Vetter W. Is there a role for the ob gene product leptin in essential hypertension? Am J Hypertens. 1998;11(11):1305–1311.
28. Tamburrino M, McGinnis R. Anorexia nervosa. A review. Panminerva Med. 2002;44(4):301–311.
29. Schaumberg DA, Christen WG, Hankinson SE, Glynn RJ. Body mass index and the incidence of visually significant age-related maculopathy in men. Arch Ophthalmol. 2001;119(9): 1259–1264.
30. Klein BE, Klein R, Lee KE, Jensen SC. Measures of obesity and age-related eye diseases. Ophthalmic Epidemiol. 2001;8(4):251–262.
31. Delcourt C, Michel F, Colvez A, Lacroux A, Delage M, Vernet M-H. Associations of cardiovascular disease and its risk factors with age-related macular degeneration: the POLA study. Ophthalmic Epidemiol. 2001;8(4):237–249.
32. Clemons T, Milton R, Klein R, Seddon J. Ferris 3rd F. Age-Related Eye Disease Study Research Group Risk factors for the incidence of advanced age-related macular degeneration in the age-related eye disease study (AREDS) AREDS report no. 19. Ophthalmol. 2005;112(4):533–539.
33. Moeini H, Masoudpour H, Ghanbari H. A study of the relation between body mass index and the incidence of age related macular degeneration. Br J Ophthalmol. 2005;89(8):964–966.
34. Defay R, Delcourt C, Ranvier M, Lacroux A, Papoz L, Group PS. Relationships between physical activity, obesity and diabetes mellitus in a French elderly population: the POLA study. Int J Obesity Rel Metab Dis. 2001;25(4):512–518.

35. Smith W, Assink J, Klein R, Mitchell P, Klaver CC, Klein BE, Hofman A, Jensen S, Wang JJ, de Jong PT, et al. Risk factors for age-related macular degeneration: pooled findings from three continents. Ophthalmol. 2001;108(4):697–704.
36. Seddon JM, Cote J, Davis N, Rosner B. Progression of age-related macular degeneration: association with body mass index, waist circumference, and waist-hip ratio. Arch Ophthalmol. 2003;121(6):785–792.
37. Cho E, Seddon JM, Rosner B, Willett WC, Hankinson SE. Prospective study of intake of fruits, vegetables, vitamins, and carotenoids and risk of age-related maculopathy. Arch Ophthalmol. 2004;122(6):883–892.
38. Chong EW, Wong TY, Kreis AJ, Simpson JA, Guymer RH. Dietary antioxidants and primary prevention of age related macular degeneration: systematic review and meta-analysis. BMJ. 2007;335(7623):755.
39. Čaljkušić Mance T, Kovačević D, Alpeza-Dunato Z, Novak Stroligo M, Brumini G. The role of omega6 to omega3 ratio in development and progression of age-related macular degeneration. Collegium Antropologicum. 2011;35(2):307–310.
