# Supplementary material for: Insights into the nutritional prevention of macular degeneration based on a comparative topic modeling approach
Source: PeerJ Comput Sci. 2024 Mar 20;10:e1940. doi: 10.7717/peerj-cs.1940 (PMC11042009; doi:10.7717/peerj-cs.1940)
Supplement: Supplemental Information 2 [file peerj-cs-10-1940-s002.zip › positive_result_reports/new4.pdf]

Dietary flavonoids and the prevalence and 15-y incidence of age-related macular degeneration

Bamini Gopinath,[1] Gerald Liew,[1] Annette Kifley,[1] Victoria M Flood,[2,4] Nichole Joachim,[1] Joshua R Lewis,[3,5,6] Jonathan M Hodgson,[5,6] and Paul Mitchell[1]

[1]Center for Vision Research, Department of Ophthalmology and Westmead Institute for Medical Research; [2]Faculty of Health Sciences; and [3]Center for Kidney Research, Westmead, School of Public Health, University of Sydney, Sydney, Australia; [4]Westmead Hospital, Western Sydney Local Health District, Westmead, Australia; [5]School of Medical and Health Sciences, Edith Cowan University, Perth, Australia; and [6]School of Medicine, University of Western Australia, Perth, Australia

ABSTRACT

Background: The majority of research performed to date has examined the effects of commonly known antioxidants such as vitamins C, E, and A and carotenoids on age-related macular degeneration (AMD) risk and progression. To date, there is limited research on promising phytochemicals with antioxidant and anti-inflammatory properties, including flavonoids.

Objective: In this exploratory study, we aimed to assess the independent associations between dietary intake of total flavonoids and common flavonoid classes with the prevalence and 15-y incidence of AMD.

Design: In this population-based cohort study, 2856 adults aged ≥49 y at baseline and 2037 followed up 15 y later were included in prevalence and incidence analyses, respectively. Dietary intake was assessed by using a semiquantitative food-frequency questionnaire (FFQ). Estimates of the flavonoid content of foods in the FFQ were assessed by using the USDA Flavonoid, Isoflavone, and Proanthocyanidin databases. AMD was assessed from retinal photographs.

Results: In cross-sectional analysis, each 1-SD increase in total overall flavonoid intake was associated with a reduced likelihood of any AMD (multivariable-adjusted OR: 0.76; 95% CI: 0.58, 0.99). Each 1-SD increase in dietary intake of total flavonols and total flavanones was associated with reduced odds of the prevalence of any AMD [multivariable-adjusted OR (95% CI): 0.75 (0.58, 0.97) and 0.77 (0.60, 0.99), respectively]. A marginally significant trend ($P = 0.05$) was observed between increasing the intake of total flavanone and hesperidin (from the first to the fourth quartile) and reduced likelihood of incident late AMD, after multivariable adjustment. Participants who reported ≥1 serving of oranges/d compared with those who never consumed oranges at baseline had a reduced risk of late AMD 15 y later (multivariable-adjusted OR: 0.39; 95% CI: 0.18, 0.85).

Conclusions: Our findings suggest an independent and protective association between dietary intake of flavonoids and the likelihood of having AMD. Additional prospective cohort studies are needed to validate these findings. *Am J Clin Nutr* 2018;108:381–387.

Keywords: age-related macular degeneration, flavonoids, Blue Mountains Eye Study, prevalence, incidence

INTRODUCTION

Age-related macular degeneration (AMD) is the leading cause of blindness and severe visual impairment in older adults (1). Current evidence suggests that patients with AMD should be given dietary advice to increase consumption of dark-green leafy vegetables, to consume low-glycemic-index diets, and to consume fish ≥2 times/wk (2–6). The Age-Related Eye Disease Study (AREDS) showed that taking a supplement containing high doses of vitamin C, vitamin E, β-carotene, zinc, and copper could reduce AMD progression by 25% (7–12). A follow-up study (AREDS 2) found that adding lutein and zeaxanthin (naturally occurring carotenoids) or omega-3 fatty acids to the original AREDS formulation (with β-carotene) had no overall effect on the risk of late AMD. However, the trial found that replacing β-carotene with a 5-to-1 mixture of lutein and zeaxanthin could help to further reduce the risk of late AMD, particularly among people

The Blue Mountains Eye Study was funded by the Australian National Health and Medical Research Council (grants 974159, 991407, 211069, and 262120) and the Westmead Institute for Medical Research. The salary of JMH was supported by a National Health and Medical Research Council (NHMRC) Senior Research Fellowship and a Royal Perth Hospital Medical Research Foundation Fellowship. The salary of JRL is supported by an NHMRC Career Development Fellowship (ID: 1107474).

Supplemental Figure 1 is available from the "Supplementary data" link in the online posting of the article and from the same link in the online table of contents at https://academic.oup.com/ajcn/.

Address correspondence to BG (e-mail: bamini.gopinath@sydney.edu.au).

Abbreviations used: AMD, age-related macular degeneration; AREDS, Age-Related Eye Disease Study; BMES, Blue Mountains Eye Study; FFQ, food-frequency questionnaire; SNP, single nucleotide polymorphism.

Received February 4, 2018. Accepted for publication May 1, 2018.

First published online July 6, 2018; doi: https://doi.org/10.1093/ajcn/nqy114.

 © 2018 American Society for Nutrition. All rights reserved.

who had a low background dietary intake of lutein and zeaxanthin (5, 6).

The majority of research performed to date has examined the effects of commonly known antioxidants such as vitamins C, E, and A and carotenoids (lutein and zeaxanthin) on AMD risk and progression. There is limited research on promising phytochemicals with antioxidant and anti-inflammatory properties, including flavonoids (13). Flavonoids are bioactive compounds found in foods such as tea, chocolate, red wine, fruit, and vegetables (14). Flavonoids found in foods can be divided into 6 main flavonoid classes: flavonols, flavan-3-ols, flavones, flavanones, anthocyanins, and isoflavones (14, 15). Flavonoids may have antioxidant and anti-inflammatory activities (15, 16). There is also strong evidence that flavonoids positively affect vascular health through improved endothelial function (17). Thus, the role of flavonoids seem promising for reversing oxidative stress and inflammation-associated damage and improving vascular function and thus possibly improving the clinical features of AMD (13).

However, additional research is needed to establish whether flavonoid intake is beneficially associated with the risk of AMD. Hence, we aimed to use a well-characterized large cohort of adults aged ≥49 y to explore the following: *1*) associations between dietary intake of total flavonoids with the prevalence and 15-y incidence of AMD (primary endpoints), independent of potential confounders; *2*) prospective relations between 6 common flavonoid classes (flavonols, flavan-3-ols, flavones, flavanones, anthocyanins, and isoflavones) and key individual flavonoids (quercetin and hesperidin) with the prevalence and 15-y incidence of AMD; and *3*) associations between the main foods and beverages contributing to total flavonoids (e.g., tea, apples, oranges, and orange juice) and both the prevalence and 15-y incidence of AMD.

METHODS

Study population

The Blue Mountains Eye Study (BMES) is a population-based cohort study of common eye diseases and other health outcomes in a suburban Australian population located west of Sydney. Study methods and procedures have been described elsewhere (18). Baseline examinations of 3654 residents aged ≥49 y were conducted during 1992–1994 (BMES-1; 82.4% participation rate). Selection bias at baseline was minimized after multiple call-back visits, including door-knocking, telephone reminders, and letters at recruitment. Surviving baseline participants were invited to attend examinations after 5 (1997–1999, BMES-2), 10 (2002–2004, BMES-3), and 15 (2007–2009, BMES-4) y, at which 2334 (75.1% of survivors), 1952 (75.6% of survivors), and 1149 (55.4% of survivors) participants were re-examined, respectively. Participants who did not return to the 5-y visit were also invited to the 10- or 15-y visits. For the current report, we analyzed data from BMES-1 through BMES-4. The University of Sydney and the Western Sydney Area Human Ethics Committees approved the study, including all methods that were performed, and written informed consent was obtained from all participants at each examination. All methods in this study were performed in accordance with the relevant guidelines and regulations.

Assessment of AMD

We took two 30° stereoscopic color retinal photographs of the macula of both eyes, which were graded for the presence of early and late AMD using the Wisconsin AMD Grading System (19, 20). Inter- and intragrader reliability showed good agreement for grading of specific AMD lesions, with quadratic weighted κ values ranging from 0.64 to 0.93 and 0.54 to 0.94, respectively (21). The detailed methodology of AMD ascertainment in this population has been previously reported (19, 20). Early AMD was defined as the absence of late AMD and the presence of either *1*) large (>125-μm diameter) indistinct soft or reticular drusen or *2*) both large distinct soft drusen and retinal pigmentary abnormalities (hyperpigmentation or hypopigmentation) in either eye (20). Similarly, late AMD was defined as the presence of neovascular AMD or geographic atrophy in either eye (20). Any AMD was defined as having early or late AMD. A retinal specialist (PM) adjudicated all uncertain retinal pathology and confirmed all late AMD cases.

Assessment of flavonoid intake

Dietary data were collected with the use of a 145-item self-administered food-frequency questionnaire (FFQ). The FFQ is modified for the Australian diet and vernacular from an early Willett FFQ (22) and includes reference portion sizes. Participants used a 9-category frequency scale to indicate the usual frequency of consumption of individual food items during the past year. Foods listed in the FFQ were categorized into major food categories and subcategories similar to those used for the 1995 Australian National Nutrition Survey (23). Estimates of the flavonoid content of foods in the FFQ were derived from the USDA Database for the Flavonoid Content of Selected Foods (24), the USDA Database for the Isoflavone Content of Selected Foods (25), and USDA Database for the Proanthocyanidin Content of Selected Foods (26).

The method of computing the flavonoid content of foods was similar to that outlined in Mink et al. (27) Specifically, for each food, we computed the intake of each individual flavonoid compound present in the food, the sum of assessed flavonoids for each flavonoid class, by summing the individual compounds of each flavonoid class, and the sum of all flavonoid intakes by summing the flavonoid classes. The flavan-3-ol content of foods was considered to represent the average of total flavan-3-ol and proanthocyanidin monomer contents. For foods in which only the flavan-3-ol or proanthocyanidin monomer content was available, the single value provided was used to represent the flavan-3-ol content. The proanthocyanidin content of foods was calculated by summing the proanthocyanidin dimers, trimers, 4–6mers, 7–10mers, and polymers. Where multiple varieties of a food listed in the FFQ were reported in the databases, the average flavonoid content of all similar varieties was computed, consistent with the descriptors used in the FFQ output. Foods in the FFQ that were not in the flavonoid databases were assumed to contain no flavonoids. Intakes of flavonoid classes (in milligrams per day) were calculated by multiplying the estimated intake (grams of edible portion per day) from the FFQ, with the flavonoid class content (milligrams of edible portion per day) of each food item on the questionnaire. Some of the food items on the FFQ with multiple ingredients (e.g., pizza) were

assigned a weighted value on the basis of a USDA standard recipe.

Assessment of covariates

Participants self-reported smoking status as never smoked, past smoker, or current smoker. We extracted separate data on the frequency of consuming fish (e.g., salmon, tuna, and sardines) and dietary intakes of lutein and zeaxanthin from the FFQ. The USDA Carotenoid Food Composition Database (28) was used to estimate the intakes of other combined lutein and zeaxanthin. Genotypic status was available for the complement factor H (*CFH*) single nucleotide polymorphism (SNP) rs1061170 in 2041 baseline participants who returned at BMES-2 and for the age-related maculopathy susceptibility gene 2 (*ARMS2*) SNP rs10490924 in 1893 baseline participants who returned at BMES-2. Two sources of genotypic information were used (29). TaqMan assays (Applied Biosystems) had been performed to provide specific genotyping of rs1061170 in 1925 individuals and rs10490924 in 638 individuals. In addition, BMES genotyping was also carried out for a genomewide association study using a custom array (Human 670-Quad, version 1; Illumina, Inc.) at the Wellcome Trust Center for Human Genetics, Sanger Institute, Cambridge, United Kingdom, as part of the Wellcome Trust Case Control Consortium 2. After quality control, genotype imputation was performed by using a genetic variation catalog (1000 Genomes, version 1) and IMPUTE software (Howie and Machini). Imputed genotypic status was available for rs1061170 in 1657 baseline participants who returned at BMES-2 and for rs10490924 in 1802 baseline participants who returned at BMES-2. This information on genotyping status from imputed data was used where TaqMan assays were not available for rs1061170 in 116 individuals and for rs10490924 in 1255 individuals. Concordance rates between typed and imputed SNP values were 99.6% for rs1061170 and 99.2% for rs10490924. Imputation data metrics were as follows: imputation R^2 values were 0.968 for rs1061170 and 0.996 for rs10490924, the proportion of the sample with missing SNP information was 8.8% for rs1061170 and 0.5% for rs10490924, Hardy-Weinberg equilibrium P values were 0.79 for rs1061170 and 0.95 for rs10490924, and minor allele frequencies were 0.39 for rs1061170 and 0.22 for rs10490924.

Statistical analysis

In exploratory analyses, we assessed associations with the prevalence and 15-y incidence of AMD, which were the primary endpoints. These primary endpoints did not change during the course of the present study or during post hoc analyses. SAS statistical software version 9.4 (SAS Institute) was used for analyses. Energy-adjusted dietary flavonoid intakes were transformed to normal scores by using the Blom method. Associations between energy-adjusted baseline dietary flavonoid intakes (study factor) and the prevalence of AMD (study outcome) were examined by using logistic regression analysis. Furthermore, associations between energy-adjusted baseline dietary flavonoid intakes and 15-y cumulative incidence of AMD were examined in discrete logistic regression models—that is, we conducted discrete-time survival analysis. The discrete logistic model refers to a survival model in which event times are treated as being genuinely discrete in truth rather than being on a continuous spectrum. The discrete time hazard is related to covariates by a logistic regression equation (30, 31). We have used its implementation in SAS in proc phreg, where a partial likelihood estimation method is used. Findings were also examined after accounting for the competing risk of death with the use of Fine and Gray's model (32) for cumulative incidence in the presence of competing risks. Regression analysis was first adjusted for age and sex, and then for covariates that have been found to be associated with the incidence of AMD in the BMES cohort: current smoking, fish consumption, intakes of lutein and zeaxanthin, and the presence of *CFH* and *ARMS2* SNPs rs1061170 and rs10490924, respectively. Genotype status was included as an adjustment factor in multivariable-adjusted models using 3 categories (no minor alleles, 1 minor allele only, or 2 minor alleles), based on an additive model for genetic effects. Further adjustments for BMI, hypertension, physical activity (in metabolic equivalents), and dietary vitamin C intake were also considered but did not appreciably change the observed estimates, so were not included in the main analysis. Findings from all analyses are expressed as adjusted ORs with 95% CIs.

RESULTS

Prevalence of AMD

Of the 3654 participants examined at baseline, 2856 who had complete dietary data as well as information on AMD lesions were included in the prevalence analysis (**Supplemental Figure 1**). Study characteristics of participants included in cross-sectional analysis are shown in **Table 1**. At baseline, there were 4.6% and 1.7% participants with early and late AMD, respectively (Table 1). After multivariable-adjustment, each 1-SD increase in intake of total flavonoids was associated with a reduced likelihood of any AMD (OR: 0.76; 95% CI: 0.58, 0.99). Each 1-SD increase in intake of total flavonols and total flavanones was associated with reduced odds of any AMD [OR (95% CI): 0.75 (0.58, 0.97) and 0.77 (0.60, 0.99), respectively]. Supplementary analysis involved key individual

TABLE 1
Baseline characteristics of participants involved in analysis of the prevalence and 15-y incidence of AMD[1]

Characteristics	Prevalence ($n = 2856$)	Incidence ($n = 2037$)
Age, y	65.3 ± 9.3	63.8 ± 8.3
Male sex, *n* (%)	1259 (44.1)	881 (43.3)
Current smokers, *n* (%)	393 (14.2)	247 (12.4)
Fish consumption (≥1 serving/wk), *n* (%)	1690 (59.8)	1200 (59.4)
Presence of 1 or 2 AMD risk alleles, *n* (%)		
CFH (rs1061170)	1077 (60.9)	1051 (60.6)
ARMS2 (rs10490924)	629 (38.4)	607 (37.9)
AMD type, *n* (%)		
Early	130 (4.6)	268 (15.3)
Late	47 (1.7)	84 (4.1)

[1]Values are means ± SDs unless otherwise indicated. P values were obtained by using t tests for continuous variables and chi-square analyses for categorical data. AMD, age-related macular degeneration; *ARMS2*, age-related maculopathy susceptibility gene 2; *CFH*, complement factor H.

TABLE 2
Associations between flavonoid intake and the prevalence of AMD in the Blue Mountains Eye Study[1]

Flavonoids, mg/d	Adjusted OR (95% CI)[2] Any AMD ($n = 177$)	Early AMD ($n = 130$)	Late AMD ($n = 47$)
All flavonoids			
First quartile (≤410.6)	1.0 (reference)	1.0 (reference)	1.0 (reference)
Second quartile (412.4–881.5)	0.63 (0.31, 1.29)	0.51 (0.23, 1.14)	1.37 (0.31, 6.13)
Third quartile (881.6–1232.3)	0.62 (0.31, 1.24)	0.63 (0.30, 1.32)	0.60 (0.11, 3.10)
Fourth quartile (≥1232.4)	0.52 (0.25, 1.06)	0.52 (0.24, 1.12)	0.45 (0.08, 2.60)
P-trend	0.08	0.12	0.25
Total flavonols			
First quartile (≤18.2)	1.0 (reference)	1.0 (reference)	1.0 (reference)
Second quartile (18.3–34.6)	0.43 (0.20, 0.90)*	0.46 (0.20, 1.07)	0.28 (0.06, 1.28)
Third quartile (34.6–46.0)	0.70 (0.36, 1.36)	0.82 (0.40, 1.69)	0.33 (0.08, 1.38)
Fourth quartile (≥46.0)	0.43 (0.21, 0.88)*	0.48 (0.22, 1.07)	0.22 (0.04, 1.02)
P-trend	0.05	0.16	0.05
Total flavanones			
First quartile (≤9.6)	1.0 (reference)	1.0 (reference)	1.0 (reference)
Second quartile (9.6–25.1)	0.51 (0.24, 1.06)	0.34 (0.14, 0.83)*	1.61 (0.40, 6.50)
Third quartile (25.2–47.1)	1.01 (0.55, 1.85)	1.05 (0.55, 1.99)	0.84 (0.18, 3.94)
Fourth quartile (≥47.2)	0.29 (0.13, 0.66)*	0.25 (0.10, 0.63)*	0.65 (0.10, 4.06)
P-trend	0.01	0.02	0.46
Total quercetin			
First quartile (≤12.3)	1.0 (reference)	1.0 (reference)	1.0 (reference)
Second quartile (12.3–20.8)	0.46 (0.22, 0.99)*	0.52 (0.23, 1.18)	0.26 (0.05, 1.48)
Third quartile (20.8–26.9)	0.73 (0.37, 1.43)	0.76 (0.36, 1.60)	0.65 (0.17, 2.52)
Fourth quartile (≥26.9)	0.49 (0.24, 1.00)	0.53 (0.24, 1.17)	0.27 (0.05, 1.39)
P-trend	0.12	0.21	0.20
Total hesperidin			
First quartile (≤5.5)	1.0 (reference)	1.0 (reference)	1.0 (reference)
Second quartile (5.5–16.0)	0.63 (0.31, 1.26)	0.49 (0.22, 1.09)	1.44 (0.36, 5.85)
Third quartile (16.0–30.1)	0.76 (0.40, 1.46)	0.78 (0.39, 1.54)	0.76 (0.15, 3.96)
Fourth quartile (≥30.2)	0.47 (0.23, 0.97)*	0.43 (0.19, 0.93)*	0.92 (0.18, 4.74)
P-trend	0.08	0.10	0.70

[1] $n = 2856$. *$P < 0.05$ compared with the reference group. AMD, age-related macular degeneration; *ARMS2*, age-related maculopathy susceptibility gene 2; *CFH*, complement factor H; SNP, single nucleotide polymorphism.

[2] Values were calculated by using logistic regression analyses and were adjusted for age, sex, current smoking, fish consumption, intakes of lutein and zeaxanthin, and *CFH* and *ARMS2* SNPs (rs1061170 and rs10490924).

flavonoids—quercetin (a flavonol) and hesperidin (flavanone)—and the prevalence of AMD. After adjustment for all potential confounders, each 1-SD increase in intake of quercetin was associated with reduced odds of any AMD (OR: 0.76; 95% CI: 0.58, 0.99). No significant linear associations were observed between hesperidin and the prevalence of AMD (data not shown).

Table 2 shows the association between quartiles of intake of flavonoids and the prevalence of AMD. Participants in the highest quartile of total flavanone intake compared with those in the lowest quartile of intake had reduced odds of any and early AMD. Those in the highest compared with the lowest quartile of total flavonol intake had a 57% reduced likelihood of any AMD, after multivariable adjustment. Participants in the highest quartile of total hesperidin intake compared with those in the lowest quartile of intake had reduced odds of any and early AMD (Table 2).

Additional analysis involved investigating the main foods and beverages contributing to total flavonoids, flavonols, and flavanones (i.e., apples, oranges, tea, and orange juice). Compared with participants who did not consume any oranges (reference group), those who reported consuming ≥1 serving of oranges/wk but <1 serving/d had reduced odds of any AMD (multivariable-adjusted OR: 0.42; 95% CI: 0.21, 0.84). Similarly, participants who reported ≥1 serving of oranges/d compared with the reference group had reduced odds of any AMD (OR: 0.42; 95% CI: 0.20, 0.89). In addition, compared with participants who did not consume any oranges, those who consumed ≥1 serving of oranges/wk but consumed <1 serving/d had 92% reduced odds of late AMD (OR: 0.08; 95% CI: 0.01, 0.76). Participants who consumed ≥1 serving of orange juice/d compared with those who never consumed orange juice had a reduced likelihood of having early AMD (multivariable-adjusted OR: 0.35; 95% CI: 0.14, 0.85). No significant associations were observed between the consumption of apples, tea, red wine, and beer and the prevalence of AMD (data not shown).

Incidence of AMD

Of the 2856 participants included in the prevalence analysis, 2037 with complete AMD and lifestyle data were re-examined 5, 10, and/or 15 y later (i.e., ≥1 follow-up examination), and were therefore included in incidence analysis (Supplemental Figure 1). Baseline characteristics of participants included in longitudinal analysis are shown in Table 1. There were 15.3% and 4.1% incident early and late AMD cases, respectively. No significant

TABLE 3
Associations between flavonoid intake and 15-y incidence of AMD in the Blue Mountains Eye Study[1]

	Adjusted OR (95% CI)	
Flavonoids, mg/d	Early AMD ($n = 268$)	Late AMD ($n = 84$)
All flavonoids		
First quartile (≤410.1)	1.0 (reference)	1.0 (reference)
Second quartile (413.0–881.5)	1.13 (0.75, 1.71)	0.72 (0.33, 1.58)
Third quartile (882.0–1232.3)	0.94 (0.62, 1.42)	1.17 (0.60, 2.29)
Fourth quartile (≥1232.4)	1.22 (0.82, 1.81)	1.00 (0.50, 2.00)
P-trend	0.35	0.65
Total flavones		
First quartile (≤0.64)	1.0 (reference)	1.0 (reference)
Second quartile (0.7–1.0)	0.97 (0.66, 1.44)	2.36 (1.13, 5.01)*
Third quartile (1.0–1.5)	0.83 (0.56, 1.23)	1.46 (0.66, 3.23)
Fourth quartile (≥1.5)	0.75 (0.50, 1.11)	1.52 (0.66, 3.49)
P-trend	0.10	0.97
Total flavanones		
First quartile (≤9.6)	1.0 (reference)	1.0 (reference)
Second quartile (9.6–25.1)	0.92 (0.62, 1.38)	1.15 (0.62, 2.11)
Third quartile (25.2–47.1)	0.97 (0.67, 1.41)	0.69 (0.36, 1.32)
Fourth quartile (≥47.2)	0.82 (0.55, 1.22)	0.55 (0.27, 1.09)
P-trend	0.30	0.05
Total hesperidin		
First quartile (≤5.5)	1.0 (reference)	1.0 (reference)
Second quartile (5.5–16.0)	1.03 (0.69, 1.53)	1.22 (0.65, 2.27)
Third quartile (16.0–30.1)	1.11 (0.76, 1.62)	0.88 (0.46, 1.68)
Fourth quartile (≥30.2)	0.85 (0.57, 1.26)	0.54 (0.26, 1.13)
P-trend	0.32	0.05

[1] $n = 2037$. Values were calculated by using discrete logistic regression models and were adjusted for age, sex, current smoking, fish consumption, intakes of lutein and zeaxanthin, and *CFH* and *ARMS2* SNPs (rs1061170 and rs10490924). *$P < 0.05$ compared with the reference group. AMD, age-related macular degeneration; *ARMS2*, age-related maculopathy susceptibility gene 2; *CFH*, complement factor H; SNP, single nucleotide polymorphism.

linear associations were observed between flavonoid intake and 15-y incidence of AMD (data not shown). A marginally significant trend was observed between increasing intake of total hesperidin (from the first to the fourth quartile) and lower 15-y incidence of late AMD, after multivariable adjustment (**Table 3**). Findings were essentially similar after accounting for the competing risk of death, except that the trend across quartiles of hesperidin became marginally nonsignificant ($P = 0.06$), whereas a significant trend emerged between quartiles of increasing flavonol intake and increased incidence of early AMD ($P = 0.03$). Participants who reported ≥1 serving of oranges/d compared with those who never ate oranges at baseline had a reduced risk of incident late AMD 15 y later (multivariable-adjusted OR: 0.39; 95% CI: 0.18, 0.85). No significant associations were observed between the consumption of apples, orange juice, tea, red wine, and beer with the 15-y incidence of AMD (data not shown).

DISCUSSION

This prospective cohort study in older adults provides epidemiologic evidence of an independent association between total flavonoid intake as well as the intake of specific flavonoid classes and AMD. Specifically, we observed significant and protective associations between the intake of total flavonoids as well as total flavonol and total flavanone intakes with AMD prevalence. Modest associations were also observed between the intakes of total flavones, flavanones, and hesperidin and the risk of incident late AMD 15 y later. Our study suggests that the consumption of oranges (a key contributor to total flavanones) is inversely and independently associated with both the prevalence and incidence of late AMD.

The median intake of total flavonoids in our cohort was 875 mg/d, which is higher than that previously reported in a Western Australia cohort (median intake of 696 mg/d in women aged >75 y) (14) and in an Australia-wide nutrition survey (median intake of 454 mg/d in those aged ≥19 y) (33). This difference is likely to be due to variations in age distribution, However, variations in food content databases and the different dietary assessment methods administered could also explain the differences in flavonoid intake observed between studies (14).

Higher total overall flavonoid intake and intakes of particular flavonoid subgroups (e.g., flavonol and flavanone) were associated with reduced odds of having AMD. This observed association is in line with existing evidence, because flavonoids are found in abundance in fruit and vegetables (15) and adequate consumption of fruit and vegetables has been established as being protective against AMD (2, 34). Our findings also concur with the existing published literature, which has shown that, after consumption, flavonoids may contribute to a variety of beneficial biological activities in humans (14). There are robust data now showing that flavonoids can preserve and enhance nitric oxide status and improve endothelial function (35, 36). There is also evidence that these compounds can minimize oxidative damage and inflammation (15, 16). Moreover, among the known angiogenesis inhibitors, flavonoids seem to play an important role (37). Although the mechanism behind the antiangiogenic effect of flavonoids is unclear, one proposed pathway is through the inhibition of protein kinases (2). Overall, these salutary effects of flavonoids may help to explain the influence these dietary compounds might have on AMD pathogenic processes—that is, the inflammatory, oxidative and angiogenic pathways (38).

The associations between flavonoid intake and both AMD prevalence and incidence appear to be class dependent. Specifically, participants with higher intakes of flavonols and flavanones had reduced odds of any AMD, whereas other flavonoid classes such as flavan-3-ols and isoflavones, did not show any significant associations with AMD prevalence. Similarly, differential associations with 15-y incidence of AMD were observed (e.g., flavone and flavanone intakes were inversely associated with the risk of incident AMD, whereas other flavonoid subgroups were not). The varying structures and bioactivities of the different flavonoid classes, as well as the ability to adequately assess intakes from foods, could explain the differential associations observed between the individual flavonoid classes and AMD prevalence and incidence (14, 39). Indeed, even a minor structural difference in flavonoids can have a large impact on their bioavailability (40, 41). Further studies are needed to confirm our findings and elucidate the influence of total flavonoids and flavonoid subclasses on the development and progression of AMD in older adults.

Our findings are promising, because BMES data show, for the first time to our knowledge, that flavonoids may be useful food compounds in protecting against AMD. The oral bioavailability of flavonoids, however, is known to be limited

by poor intrinsic transmembrane diffusion characteristics and poor solubility (42). Moreover, the activity of the flavonoid metabolites is not well established (42). Further research is also needed to establish whether systemic administration of flavonoids will yield much higher and effective concentrations of the parent flavonoids in the ocular tissues and at much lower doses (42). For the time being, it is reasonable that adequate intakes of fruit (particularly oranges), vegetables, and beverages (e.g., orange juice) containing flavonoids be recommended to patients, although it is too early to make recommendations on daily flavonoid intakes for the prevention of AMD (15). Strengths of this study include its prospective data collection, long-term follow-up of a population-based sample, use of a validated FFQ, and careful adjustment for confounders, including genetic risk. Hence, our findings are applicable to the general older Australian population and could also be applicable to older adults in other Western countries. In addition, this study uses high-quality stereoscopic retinal photography with validated grading to assess macular conditions and a detailed side-by-side comparison of the baseline and follow-up photographs to ensure negligible misclassification of incident AMD (4, 43, 44). However, this study has some noteworthy limitations. First, the database used for the estimation of flavonoid content of foods is based on US data only and therefore this approach might not have accounted for any variation in the flavonoid content of foods found in Australia (40). Second, we cannot discount the effect of residual confounding from unmeasured or unaccounted factors (e.g., inflammatory markers) on observed associations. Finally, the number of participants who developed incident AMD was small, and this might have reduced the power to detect modest associations with flavonoid intake.

In summary, we report independent associations between dietary intakes of total flavonoids, and some of the common flavonoid classes (e.g., flavonols and flavanones), and AMD among older adults. Furthermore, the consumption of oranges and orange juice, one of the main foods and beverages contributing to total flavanone intake, is also likely to independently influence the risk of AMD. These findings suggest that a habitual diet high in flavonoids could play a role in AMD prevention and progression. These associations, if confirmed in other epidemiologic and intervention studies, could have important public health implications.

The authors' responsibilities were as follows—BG and PM: designed the research; PM, BG, VMF, JMH, and JRL: conducted the research; AK: analyzed data or performed statistical analysis; BG, PM, NJ, and GL: wrote the manuscript; BG: had primary responsibility for final content; and all authors: read and approved the final manuscript. None of the authors declared a conflict of interest.

REFERENCES

1. Foran S, Wang JJ, Mitchell P. Causes of visual impairment in two older population cross-sections: the Blue Mountains Eye Study. Ophthalmic Epidemiol 2003;10:215–25.
2. Broadhead GK, Grigg JR, Chang AA, McCluskey P. Dietary modification and supplementation for the treatment of age-related macular degeneration. Nutr Rev 2015;73:448–62.
3. Gopinath B, Flood VM, Louie JC, Wang JJ, Burlutsky G, Rochtchina E, Mitchell P. Consumption of dairy products and the 15-year incidence of age-related macular degeneration. Br J Nutr 2014;111:1673–79.
4. Gopinath B, Flood VM, Rochtchina E, Wang JJ, Mitchell P. Homocysteine, folate, vitamin B-12, and 10-y incidence of age-related macular degeneration. Am J Clin Nutr 2013;98:129–35.
5. Chew EY, Clemons TE, SanGiovanni JP, Danis RP, Ferris FL III, Elman MJ, Antoszyk AN, Ruby AJ, Orth D, Bressler SB, et al. Secondary analyses of the effects of lutein/zeaxanthin on age-related macular degeneration progression: AREDS2 report no. 3. JAMA Ophthalmol 2014;132:142–49.
6. Age-Related Eye Disease Study 2 Research Group. Lutein + zeaxanthin and omega-3 fatty acids for age-related macular degeneration: the Age-Related Eye Disease Study 2 (AREDS2) randomized clinical trial. JAMA 2013;309:2005–15.
7. Age-Related Eye Disease Study Research Group. A randomized, placebo-controlled, clinical trial of high-dose supplementation with vitamins C and E, beta carotene, and zinc for age-related macular degeneration and vision loss: AREDS report no. 8. Arch Ophthalmol 2001;119:1417–36.
8. Bressler NM, Bressler SB, Congdon NG, Ferris FL III, Friedman DS, Klein R, Lindblad AS, Milton RC, Seddon JM. Potential public health impact of Age-Related Eye Disease Study results: AREDS report no. 11. Arch Ophthalmol 2003;121:1621–24.
9. Clemons TE, Milton RC, Klein R, Seddon JM, Ferris FL III. Risk factors for the incidence of advanced age-related macular degeneration in the Age-Related Eye Disease Study (AREDS): AREDS report no. 19. Ophthalmology 2005;112:533–39.
10. Age-Related Eye Disease Study Research Group. The Age-Related Eye Disease Study severity scale for age-related macular degeneration: AREDS report number 17. Arch Ophthalmol 2005;123:1484–98.
11. Age-Related Eye Disease Study Research Group. A simplified severity scale for Age-Related Macular Degeneration: AREDS report number 18. Arch Ophthalmol 2005;123:1570–4.
12. AREDS2 Research Group EY, Chew, Clemons T, SanGiovanni JP, Danis R, Domalpally A, McBee W, Sperduto R, Ferris FL. The Age-Related Eye Disease Study 2 (AREDS2): study design and baseline characteristics (AREDS2 report number 1). Ophthalmology 2012;119:2282–29.
13. Rhone M, Basu A. Phytochemicals and age-related eye diseases. Nutr Rev 2008;66:465–72.
14. Ivey KL, Hodgson JM, Croft KD, Lewis JR, Prince RL. Flavonoid intake and all-cause mortality. Am J Clin Nutr 2015;101:1012–20.
15. Nijveldt RJ, van Nood E, van Hoorn DE, Boelens PG, van Norren K, van Leeuwen PA. Flavonoids: a review of probable mechanisms of action and potential applications. Am J Clin Nutr 2001;74:418–25.
16. Middleton E Jr. Effect of plant flavonoids on immune and inflammatory cell function. Adv Exp Med Biol 1998;439:175–82.
17. Bondonno CP, Croft KD, Ward N, Considine MJ, Hodgson JM. Dietary flavonoids and nitrate: effects on nitric oxide and vascular function. Nutr Rev 2015;73:216–35.
18. Attebo K, Mitchell P, Smith W. Visual acuity and the causes of visual loss in Australia: the Blue Mountains Eye Study. Ophthalmology 1996;103:357–64.
19. Wang JJ, Rochtchina E, Lee AJ, Chia EM, Smith W, Cumming RG, Mitchell P. Ten-year incidence and progression of age-related maculopathy: the Blue Mountains Eye Study. Ophthalmology 2007;114:92–98.
20. Klein BE, Moss SE, Magli YL, Klein R, Hoyer C, Johnson J. Optic disc cupping: prevalence findings from the WESDR. Invest Ophthalmol Vis Sci 1989;30:304–9.
21. Mitchell P, Smith W, Attebo K, Wang JJ. Prevalence of age-related maculopathy in Australia: the Blue Mountains Eye Study. Ophthalmology 1995;102:1450–60.
22. Willett WC, Sampson L, Browne ML, Stampfer MJ, Rosner B, Hennekens CH, Speizer FE. The use of a self-administered questionnaire to assess diet four years in the past. Am J Epidemiol 1988;127:188–99.
23. McLennan W. Australian Statistician. National Nutrition Survey, Confidentialized Unit Record File. (4807.0). Canberra (Australia): Australian Bureau of Statistics; 1995.
24. Bhagwat S, Haytowitz DB, Holden JMR. USDA Database for the Flavinoid Content of Selected Foods, release 3.1. Maryland, USA: USDA, Agricultural Research Service; 2014.
25. Bhagwat S, Holden JM, Haytowitz DB. USDA Database for the Isoflavone Content of Selected Foods, release 2.0. Nutrient Data

Laboratory Home Page. USDA, Agricultural Research Service . 2008. Available from: http://www.ars.usda.gov/nutrientdata/isoflav. Accessed 04 September 2017.

26. Bhagwat S, Haytowitz DB. USDA Database for the Proanthocyanidin Content of Selected Foods. Maryland, USA: USDA, Agricultural Research Service; 2015.
27. Mink PJ, Scrafford CG, Barraj LM, Harnack L, Hong CP, Nettleton JA, Jacobs DR Jr. Flavonoid intake and cardiovascular disease mortality: a prospective study in postmenopausal women. Am J Clin Nutr 2007;85:895–909.
28. Chug-Ahuja JK, Holden JM, Forman MR, Mangels AR, Beecher GR, Lanza E. The development and application of a carotenoid database for fruits, vegetables, and selected multicomponent foods. J Am Diet Assoc 1993;93:318–23.
29. Holliday EG, Smith AV, Cornes BK, Buitendijk GHS, Jensen RA, Sim X, Aspelund T, Aung T, Baird PN, Boerwinkle E et al. Insights into the genetic architecture of early stage age-related macular degeneration: a genome-wide association study meta-analysis. PLoS One 2013;8:e53830.
30. Cox DR. Regression models and life-tables. J R Stat Soc Series B Methodol 1972;34:187–220.
31. Gopinath B, Liew G, Kifley A, Mitchell P. Thyroid dysfunction and ten-year incidence of age-related macular degeneration. Invest Ophthalmol Vis Sci 2016;57:5273–77.
32. Fine JP, Gray RJ. A proportional hazards model for the subdistribution of a competing risk. J Am Stat Assoc 1999;94: 496–509.
33. Johannot L, Somerset SM. Age-related variations in flavonoid intake and sources in the Australian population. Public Health Nutr 2006;9:1045–54.
34. Gopinath B, Liew G, Flood VM, Joachim N, Burlutsky G, Mitchell P. Combined influence of poor health behaviours on the prevalence and 15-year incidence of age-related macular degeneration. Sci Rep 2017;7:4359.
35. Loke WM, Hodgson JM, Proudfoot JM, McKinley AJ, Puddey IB, Croft KD. Pure dietary flavonoids quercetin and (-)-epicatechin augment nitric oxide products and reduce endothelin-1 acutely in healthy men. Am J Clin Nutr 2008;88:1018–25.
36. Schroeter H, Heiss C, Balzer J, Kleinbongard P, Keen CL, Hollenberg NK, Sies H, Kwik-Uribe C, Schmitz HH, Kelm M. (-)-Epicatechin mediates beneficial effects of flavanol-rich cocoa on vascular function in humans. Proc Natl Acad Sci USA 2006;103:1024–29.
37. Paper DH. Natural products as angiogenesis inhibitors. Planta Med 1998;64:686–95.
38. Klein R, Myers CE, Cruickshanks KJ, Gangnon RE, Danforth LG, Sivakumaran TA, Iyengar SK, Tsai MY, Klein BE. Markers of inflammation, oxidative stress, and endothelial dysfunction and the 20-year cumulative incidence of early age-related macular degeneration: the Beaver Dam Eye Study. JAMA Ophthalmol 2014;132:446–55.
39. Rice-Evans CA, Miller NJ. Antioxidant activities of flavonoids as bioactive components of food. Biochem Soc Trans 1996;24:790–5.
40. Ivey KL, Lewis JR, Prince RL, Hodgson JM. Tea and non-tea flavonol intakes in relation to atherosclerotic vascular disease mortality in older women. Br J Nutr 2013;110:1648–55.
41. Loke WM, Proudfoot JM, Stewart S, McKinley AJ, Needs PW, Kroon PA, Hodgson JM, Croft KD. Metabolic transformation has a profound effect on anti-inflammatory activity of flavonoids such as quercetin: lack of association between antioxidant and lipoxygenase inhibitory activity. Biochem Pharmacol 2008;75:1045–53.
42. Majumdar S, Srirangam R. Potential of the bioflavonoids in the prevention/treatment of ocular disorders. J Pharm Pharmacol 2010;62:951–65.
43. Kaushik S, Wang JJ, Flood V, Tan JS, Barclay AW, Wong TY, Brand-Miller J, Mitchell P. Dietary glycemic index and the risk of age-related macular degeneration. Am J Clin Nutr 2008;88:1104–10.
44. Tan JS, Wang JJ, Flood V, Mitchell P. Dietary fatty acids and the 10-year incidence of age-related macular degeneration: the Blue Mountains Eye Study. Arch Ophthalmol 2009;127:656–65.
