# Supplementary material for: Insights into the nutritional prevention of macular degeneration based on a comparative topic modeling approach
Source: PeerJ Comput Sci. 2024 Mar 20;10:e1940. doi: 10.7717/peerj-cs.1940 (PMC11042009; doi:10.7717/peerj-cs.1940)
Supplement: Supplemental Information 2 [file peerj-cs-10-1940-s002.zip › positive_result_reports/i1552-5783-54-14-ORSF42.pdf]

Special Issue

Nutrition Effects on Ocular Diseases in the Aging Eye

Emily Y. Chew

National Eye Institute, National Institutes of Health, Bethesda, Maryland

Correspondence: Emily Y. Chew, National Eye Institute, National Institutes of Health, Building 10, CRC Room 3-2531, 10 Center Drive, MSC 1204, Bethesda, MD 20892-1204; echew@nei.nih.gov.

Submitted: July 25, 2013
Accepted: August 22, 2013

Citation: Chew EY. Nutrition effects on ocular diseases in the aging eye. *Invest Ophthalmol Vis Sci*. 2013;54:ORSF42–ORSF47. DOI:10.1167/iovs.13-12914

Purpose. We reviewed the data from the clinical trials of nutritional supplements for the treatment of age-related cataract and age-related macular degeneration (AMD) to determine future directions of research and treatment.

Methods. Data from the controlled clinical trials are presented and reviewed for potential opportunities for further research into the treatment of cataracts and AMD.

Results. Two trials using daily multivitamins/minerals demonstrated a reduction in the progression of nuclear cataract, but increased the risk of posterior subcapsular cataract. For AMD, the Age-Related Eye Disease Study (AREDS) formulation (vitamins C, E, beta-carotene, zinc, and copper) reduced the risk of progression to advanced AMD by 25% at 5 years. Because beta-carotene is associated with increased lung cancer in former smokers, lutein/zeaxanthin could replace beta-carotene and provide an incremental increase in the beneficial effects beyond the effects of the AREDS formulation. In addition, a randomized clinical trial of B vitamins demonstrated a beneficial effect for AMD with the vitamin B complex.

Conclusions. Future evaluation may include additional assessments of nutrients for the treatment of progression of cataract and AMD. A modest reduction would have significant impact as the numbers of persons affected with these two leading causes of blindness are projected to double in the next decade. An important step would be to develop surrogate outcomes to increase efficiency in clinical trials. More detailed phenotyping, especially of AMD, is required as it appears to be not one disease, but a group of diseases. Genotype-phenotype analyses may help to target pathways that are important in AMD.

Keywords: age-related macular degeneration, cataract type, antioxidants

Age-related cataract and macular degeneration (AMD), two leading causes of blindness in the United States, are age-related ocular conditions in which nutritional risk factors have been evaluated extensively with observational and interventional studies. The results of a number of observational studies have provided the hypotheses and rationale for the interventional studies. The results of these interventional trials will be presented to identify potential opportunities for further research in these ocular conditions of significant public health importance. The Table gives a compilation of randomized controlled clinical trials for age-related cataract and AMD, including the nutrients evaluated and the study results.

Age-Related Cataracts

Age-related cataract is the leading cause of vision loss in the United States and its prevalence is rising; an estimated 30.1 million Americans will be affected by 2020.[1] Treatments that can delay the progression of lens opacities likely would reduce the burden of disease markedly. Numerous observational epidemiologic studies have demonstrated inverse relationships between dietary micronutrients and the development of age-related cataract or cataract surgery.[2–7] These studies have generated interest in micronutrients with antioxidant capabilities because of the potential importance of oxidative damage in cataract formation. Several controlled clinical trials have tested whether selected micronutrients with antioxidant characteristics or multivitamins affect cataract development.[8–14] The results of some of these studies will be discussed below and areas of future research will be considered.

Controlled Clinical Trials of Nutritional Supplements for Age-Related Cataract

The Linxian Cataract Trials

These trials were ancillary studies in a nutritionally deprived population at high risk of developing esophageal/gastric cancer living in Linxian, China.[10] Separate clinical trials tested multivitamins/minerals ($n = 2141$), and a mixture ($n = 3249$) containing retinol and zinc, riboflavin and niacin, vitamin C and molybdenum, selenium, vitamin E, and beta-carotene, using a factorial design with these four different vitamin/mineral combinations. In the studies, the risk of nuclear cataract progression was decreased by at least 36% and 44%, for multivitamins and mixture, respectively, in 5 to 6 years. However, there appeared to be an increased risk of posterior subcapsular cataract with the use of riboflavin/niacin. In this population, the results of the two trials suggest that vitamin/mineral supplements may be beneficial for slowing the progression of lens opacities. Further research is suggested in a less nutritionally deprived population before such results can be translated into general recommendations.

Copyright 2013 The Association for Research in Vision and Ophthalmology, Inc.

Investigative Ophthalmology & Visual Science

Table. Randomized Controlled Clinical Trials for Age-Related Cataract and AMD

Study	Participants	Nutrients Evaluated	Study Results
**Randomized, double-masked, placebo-controlled cataract trials**			
Linxian Cataract Trial[10] (China) Follow-up: 5–6 y	Study I: $n = 2{,}141$ Study II: $n = 3{,}249$ (ages 45–75)	I: Multivitamin/mineral<br>II: Retinal/zinc, riboflavin/niacin, vitamin C/molybdenum, vitamin E/beta-carotene	I: decrease by 36% in 65–74 y for nuclear cataract progression<br>II: decrease by 44% in 65–74 y nuclear cataract for riboflavin/niacin<br>Increased risk of posterior subcapsular cataract
Physicians' Health Study[15] Follow-up: 8 y	11,545 male physcians, ≥50 y	Vitamin E (440 IU every other day)/vitamin C (500 mg daily)	Vitamin E: HR:0.99 95% CI, 0.88–1.11<br>Vitamin C: HR 1.02 95% CI, 0.91–1.14 for incident cataract
Physicians' Health Study[19] Follow-up: 12 y	22,071 male physicians, ≥50 y	Beta-carotene (50 mg every other day)	RR 1.00, 95% CI, 0.91–1.09 for incident cataract<br>RR 1.00, 95% CI, 0.89–1.12 for cataract surgery
Women's Health Study[12] Follow-up: 9.7 y	39,876 female health professionals ≥45 y	Vitamin E (600 IU every other day)	RR 0.96, 95% CI, 0.88–1.04 for incident cataract
Age-Related Eye Disease Study (AREDS)[17] Follow-up: 6.3 y	4,629 men and women 55–80 y	Daily vitamins C (500 mg), E (400 IU), beta-carotene (15 mg), zinc (80 mg), copper (2 mg, AREDS formulation)	OR 0.97, 99% CI, 0.84–1.11 ($P = 0.55$) for any incident cataract event
Age-Related Eye Disease Study 2 (AREDS2)[18] Follow-up: 4.7 y	3,159 men and women 50–80 y	Lutein 10 mg and zeaxanthin 2 mg with the AREDS formulation	HR 0.95 95% CI, 0.83–1.09
Italian-American Clinical Trial of Nutritional Supplements (CNTS)[14] (Italy) Follow-up: 9 y	1,020 men and women 55–75 y	Multivitamin/mineral formulation: (Centrum; Lederle Laboratory, Inc., Pearl River, NY)	HR 0.82 95% CI, 0.68–0.98 ($P = 0.03$) for incident cataract<br>HR 0.66 95% CI, 0.50–0.88, ($P = 0.004$) for incident nuclear cataract<br>HR 2.00 95% CI, 1.35–2.98, ($P = 0.001$) for incident posterior subcapsular cataract
**Randomized, double-masked, placebo-controlled age-related macular degeneration trials**			
Randomized trial of Zinc[41] Follow-up: 12–24 m	151 with early AMD, 42–89 y	Zinc sulfate (100 mg); equivalent to 80 mg of elemental zinc	Visual acuity change (final compared to baseline vision) was significantly less likely to deteriorate in the zinc treated group ($P < 0.05$)
Age-Related Eye Disease Study (AREDS)[17] Follow-up: 6.3 y	3,640 men and women 55–80 y	Daily vitamins C (500 mg), E (400 IU), beta-carotene (15 mg), zinc (80 mg), copper (2 mg, AREDS formulation)	OR 0.72, 99% CI, 0.52–0.98 ($P = 0.002$) for progression to advanced AMD
Age-Related Eye Disease Study 2 (AREDS2)[18] Follow-up: 5.0 y	4,203 men and women 50–85 y	Lutein 10 mg and zeaxanthin 2 mg and omega-3 fatty acids (DHA and EPA 1 g) along with the AREDS formulation	Primary analyses using the entire study population did result in statistically significant differences. Main effects analyses of lutein/zeaxanthin vs. NO lutein/zeaxanthin: HR: 0.90, 95% CI, 0.82–0.99, ($P = 0.04$)
Women's Antioxidant and Folic Acid Cardiovascular Study Follow-up: 7.3 y	5,205 female health care professionals at risk or with preexisting cardiovascular disease ≥ 40 y	Folic acid 2.5 mg, pyridoxine hydrochloride or B_6 50 mg, cyancobalamin or B_{12} 1 mg	RR 0.66, 95% CI, 0.47–0.93 ($P = 0.02$) for development of advanced AMD

Investigative Ophthalmology & Visual Science

Physicians' Health Study

The Physicians' Health Study evaluated aspirin and a number of nutrients, including vitamins E, C, and beta-carotene, in a randomized, double-masked, placebo-controlled trial for cardiovascular disease and cancer. Secondary analyses included the ocular end-points of age-related cataracts and AMD in male physicians 50 years or older. These outcomes were obtained with self-reports and confirmed by medical records.

Vitamins E and C. In a randomized, double-masked, placebo-controlled trial of vitamin E (400 IU) every other day and vitamin C (500 mg) daily in 11,545 male physicians, the rates of incident cataract were investigated.[15] After 8 years of treatment and follow-up, there were no beneficial or harmful effects of this treatment regimen of vitamins E and C on the development of incident cataract in comparison of those treated versus the placebo group. The hazard ratios (HRs; 95% confidence intervals [CI]) were 0.99 (95% CI, 0.88–1.11) for vitamin E versus placebo and 1.02 (95% CI, 0.91–1.14) for vitamin C versus placebo.

Beta-Carotene. In a secondary study of a randomized controlled clinical trial of beta-carotene (50 mg on alternate days) versus placebo for cataract in 22,071 male physicians followed for a mean of 12 years, incident cataract and cataract surgery were the main outcomes.[9] There was no statistically significant difference between beta-carotene versus placebo on the incidence of cataract (relative risk [RR] of 1.00; 95% CI, 0.91–1.09) or cataract surgery (RR of 1.00; 95% CI, 0.89–1.12).

Women's Health Study

The Women's Health Study was a randomized, double-masked, placebo-controlled trial to investigate the effects of beta-carotene (50 mg every other day), and daily vitamin E and aspirin in the primary prevention of cancer and cardiovascular disease.[12] The study participants were 39,876 female health professionals aged at least 45 years. The main ocular outcome measures were visually-significant cataract and cataract extraction, based on self-reports with confirmation with medical records.

After a median follow-up of 2.1 years with beta-carotene, the randomization was stopped. There were neither statistically significant beneficial nor harmful effects of beta-carotene on the development of cataract. For the development of incident cataract, the RR was 0.95 (95% CI, 0.75–1.21) and for cataract extraction, the RR was 1.04 (95% CI, 0.78–1.39).

This study also evaluated vitamin E (600 IU on alternate days) versus placebo for a mean follow-up of 9.7 years for incident cataract.[16] Again, there was no significant difference between vitamin E and placebo for the development of cataract (RR of 0.96; 95% CI, 0.88–1.04). No differences also were noted for the subtypes of cataract or cataract surgery.

Age-Related Eye Disease Study (AREDS)

This was a randomized controlled clinical trial that evaluated antioxidant vitamins (500 mg C, 400 IU of E) and beta-carotene (15 mg) daily for the treatment of cataract in persons with varying degree of AMD.[17] The mean follow-up was 6.3 years in 4629 AREDS participants, both men and women aged 55 to 80 years. There was no effect on progression of the three types of cataract or the incidence of cataract surgery (odds ratio [OR], 0.97; 99% CI, 0.84–1.11; $P = 0.55$ for progression to any cataract event.

Age-Related Eye Disease Study 2 (AREDS2)

The AREDS2 is a randomized controlled clinical trial of lutein/zeaxanthin for the treatment of age-related cataract.[18] The 3159 AREDS2 participants, aged 50 to 80, were followed for a median of 4.7 years. The HR for progression to cataract surgery, the main outcome measure, was 0.95 (95% CI, 0.83–1.09; $P = 0.49$), comparing lutein/zeaxanthin versus no lutein/zeaxanthin. Lutein/zeaxanthin was not recommended for treatment of cataract, although those participants whose dietary intake of lutein/zeaxanthin was in the lowest quintile experienced a beneficial effect of the supplement. These data will need to be investigated further in similar studies of lutein/zeaxanthin for cataract progression.

The Italian-American Clinical Trial of Nutritional Supplements and Age-Related Cataract (CTNS)

This 13-year single-center clinical trial was designed to evaluate the role of multivitamin/mineral supplement (Centrum; Lederle Laboratory, Inc., Pearl River, NY) for the treatment of age-related cataracts. A total of 1020 participants was enrolled at the University of Parma, Parma, Italy, and followed for a mean of 9 years.[14] In general, there was a decrease in the total number of subjects with lens progression in those participants assigned to Centrum compared to those assigned to placebo (HR, 0.82; 95% CI, 0.68–0.98; $P = 0.03$). Progression to nuclear cataract was particularly decreased (HR, 0.66; 95% CI, 0.50–0.88; $P = 0.004$). Posterior subcapsular cataract (PSC) events, however, were increased with the use of Centrum compared to placebo (HR, 2.00; 95% CI, 1.35–2.98; $P = 0.001$). Because of these mixed results, that is, opposite effects on nuclear and PSC, no recommendations had been made by the investigators for the treatment of cataract.

Age-Related Macular Degeneration (AMD)

Age-related macular degeneration is the leading cause of blindness, accounting for more than 50% of blindness in the United States.[19] Therefore, this is an ocular disease of major public health importance. It is predicted that by 2020, there may be nearly 3 million individuals in the United States with advanced AMD.[20] Although anti-VEGF agents have reduced the risk of vision loss from neovascular AMD, to our knowledge there are no proven treatments for the atrophic (dry) form of AMD. The role of nutritional supplements has been raised by a number of observational and randomized clinical trials.

Controlled Clinical Trials of Nutritional Supplements for Treatment of AMD

The pathogenesis of AMD is unknown, although oxidative stress is considered to have a role. Since 1988, evaluation of the National Health and Nutrition Examination survey suggested that persons eating a diet with increased sources of vitamins A and C had an inverse association with AMD. Other observational studies also have implicated inverse dietary associations with AMD.[21–26] Observational data from AREDS and epidemiologic studies provide a rationale for examining the potential impact of other nutrients on the development of AMD, specifically higher dietary intake of lutein/zeaxanthin and/or omega-3 long-chain polyunsaturated fatty acids (LCPUFAs; docosahexanoic acid [DHA]/eicosapentanoic acid [EPA]) are associated with a decreased risk of developing advanced AMD.[27–40] These studies have provided data to support hypotheses that were tested in the controlled clinical trials of nutrients for the treatment of AMD presented below.

Investigative Ophthalmology & Visual Science

Randomized Trial of Zinc Supplements

This was a single center study of a randomized controlled clinical trial of zinc versus placebo in 151 individuals with early AMD.[41] A daily dose of zinc sulfate (100 mg) versus placebo was tested with 1:1 randomization. The outcome measurement was visual acuity change compared to baseline. There was a significantly positive treatment effect with zinc ($P < 0.05$). Importantly, the final visual acuity was significantly less likely to deteriorate in the zinc-treated group.

The AREDS

The AREDS was a randomized, double-masked, placebo-controlled clinical trial designed to evaluate vitamins C (500 mg), E (400 IU), and beta-carotene (15 mg), with or without zinc (zinc oxide 80 mg), with copper (2 mg cupric oxide), in a factorial design for the treatment of AMD.[42] The 3640 AREDS participants, aged 55 to 80 years, had varying severity of AMD and were followed for a mean of 6.3 years. The combination of the antioxidant vitamins with the minerals demonstrated a statistically significant odds reduction for the progression to advanced AMD (OR, 0.72; 99% CI, 0.52–0.98; $P = 0.002$). There was an approximately 25% reduction in risk of developing advanced AMD at 5 years. This treatment effect appeared to persist following 5 additional years of follow-up after the clinical trial was stopped.[43] The AREDS formulation became the standard of care for persons who are at high risk for AMD, either intermediate AMD or large drusen in both eyes, or advanced disease in one eye with large drusen in the fellow eye.

The AREDS2

The AREDS2,[44] a multicenter, randomized, double-masked, placebo-controlled clinical, was conducted in 4203 participants, aged 50 to 85 years, who had intermediate AMD (bilateral large drusen) or large drusen in 1 eye and advanced AMD in the fellow eye. They were assigned randomly to placebo, lutein (10 mg)/zeaxanthin (2 mg), omega 3 fatty acids (DHA 350 mg and EPA 650 mg), or the combination of lutein/zeaxanthin and omega-3 fatty acids. In addition, all the participants were administered either the original AREDS formulation (vitamins C, E, and beta-carotene, and zinc with copper) or some modification of the AREDS formulation (either elimination of beta-carotene, lowering of the zinc, or the combination of the two). The participants were followed for a median of approximately 5 years. Approximately 50% of the participants were former smokers and 7% were current smokers.

The primary analyses consisted of comparing each treatment group with the placebo group, using only half of the study population (~2000) for each of these analyses. These three treatment groups did not demonstrate additional beneficial effects beyond the effects of the AREDS formulation. However, in the analyses which used the entire population (4000+) to conduct the analysis of the main effects, that is, evaluating all those assigned to lutein/zeaxanthin ($n = 2123$) versus those not assigned lutein/zeaxanthin ($n = 2080$), there was an added 10% reduction in the risk of progression to advanced AMD (HR, 0.90; 95% CI, 0.82–0.99; $P = 0.04$). When analyses were restricted to those with lowest dietary intake of lutein/zeaxanthin, there was a further reduction in the risk of advanced AMD (HR, 0.75; 95% CI, 0.59–0.94; $P = 0.01$). When lutein/zeaxanthin with AREDS formulation minus the beta-carotene treatment group was compared to the AREDS (with beta-carotene and no lutein/zeaxanthin) treatment group, the HR was 0.82 (95% CI, 0.69–0.96, $P = 0.02$). The adverse effect associated with beta-carotene was the increased risk of lung cancer in former smokers. A number of other secondary analyses, some prespecified and others were post hoc, as well as the emergence of this important adverse effect contributes to the totality of the evidence that suggest adding lutein/zeaxanthin and eliminating beta-carotene from the AREDS formulation may result in an incremental increase beyond the beneficial effects of the AREDS formulation for reduction in the risk of developing advanced AMD.

Vitamin B Complex, the Women's Antioxidant, and Folic Acid Cardiovascular Study

The Women's Antioxidant and Folic Acid Cardiovascular Study was a randomized controlled clinical trial of female health care professionals ($n = 5442$) who were 40 years or older with preexisting cardiovascular disease, or who have three or more cardiovascular disease risk factors.[45] They were randomized to a combination of B vitamins (folic acid 2.5 mg, pyridoxine hydrochloride or B_6 at 50 mg, cyanocobalamin or B_{12} at 1 mg) or placebo daily. A total of 5205 women, who were not diagnosed with AMD at baseline, was followed for a period of 7.3 years. The outcome was incident AMD that was self-reported and confirmed by medical records. The results demonstrated that daily supplementation of the B vitamins, folic acid, pyridoxine, and cyanocobalamin may reduce the risk of AMD (RR, 0.66; 95% CI, 0.47–0.93; $P = 0.02$). Since these were secondary analyses, further studies are warranted before making clinical recommendations for use of B vitamins in the treatment of AMD.

Discussion: Unmet Needs and Opportunities for Future Research

Cataract

The data from randomized controlled clinical trials mostly have failed to demonstrate beneficial effects of nutritional supplements for the treatment of cataract. However, two randomized controlled clinical trials of multivitamins/minerals have suggested a beneficial role for the treatment of age-related cataracts, one in a nutritional deprived population in Linxian, China, and another in a well-nourished population in Parma, Italy. The beneficial effect for nuclear cataract was countered by the harmful effect of developing PSC cataract. One study suggested that riboflavin/niacin may be contributing to this deleterious effect. None of these studies has evaluated the individual nutrient that may be important in the multivitamin/mineral combination. Future studies may have the opportunity to provide clarification. However, such a trial may be extremely costly, requiring tremendous numbers of subjects and lengthy follow-up. Thus, an important area of future research is to find clinically meaningful surrogate outcomes that would allow clinical research to be conducted in a shorter interval of time with smaller number of participants. An example of this would be the potential use of the technology designed to detect precataractous lens protein changes clinically using dynamic light scattering.[46] The dynamic light-scattering device has been used to evaluate α-crystallin, a molecular chaperone protein that has been demonstrated to bind to damaged lens proteins, which prevent their aggregation. This measure of an α-crystallin index decrease was associated with increasing nuclear lens opacities ($P < 0.002$). Even in clinically clear lens, significant decreases in α-crystallin can be detected.

A small reduction in the progression of age-related cataract would have a profound effect on the costs of American health, as this condition currently is a significant public health

Investigative Ophthalmology & Visual Science

problem, with large numbers of people affected with the disease projected to increase markedly with increasing longevity. Currently, one of the most commonly performed procedures in the United States is cataract surgery. A successful preventive measure would reduce the cost of this condition to society, and to the individuals and their families.

Although the pathogenesis is not well understood, antioxidative properties of nutrient-based therapies are attractive for these chronic conditions. The therapeutic effects seen in those with the lowest dietary intake of lutein/zeaxanthin, for example, suggest that nutrient therapy may have a role in preventing cataract. The public health message of "You are what you eat" is important. Eating well, and having a diet replete with fish and many colored vegetables, especially the green leafy ones, are public health messages that might be particularly important to communicate to the entire population. This has implications for general well-being as well as potential reduction of ocular diseases.

Age-Related Macular Degeneration

The nutritional supplement, known as the AREDS formulation, has been demonstrated to reduce the progression to advanced AMD by 25% in 5 years in persons with intermediate AMD (bilateral large drusen) or having large drusen in one eye and advanced AMD in the fellow eye. There is an incremental increase in the efficacy of the AREDS formulation when beta-carotene is replaced by the addition of lutein/zeaxanthin to this formulation. Again, those in the lowest dietary intake group of lutein/zeaxanthin appear to have the greatest benefit. Thus, the public health message of proper diet is essential for prevention of chronic disease, such as AMD and other systemic conditions.

Aside from the beneficial effects of folic acid during pregnancy for the prevention of spina bifida and calcium for prevention of osteoporosis, the AREDS formulation is the only other nutritional supplement known to have beneficial effects on any disease. All the previous studies of nutritional supplements for cardiovascular disease and cancer have been proven to be negative. For example, nutritional studies of the B vitamins for cardiovascular disease were negative, while the ancillary studies in ocular disease demonstrated a beneficial effect for AMD. Ideally, it would be prudent to have a duplicate randomized, double-masked, controlled clinical trial to support the AREDS formulation and its effects on the retina. With limited resources, the feasibility of replicating the AREDS is unlikely as the AREDS formulation has become the standard of care for many of the individuals at risk for developing advanced AMD. One is reassured by the beneficial effects that persisted in the observational study that followed the clinical trial. At 10 years, there was still a 27% reduction in the risk of developing advanced AMD. In addition to the nutrients evaluated in AREDS2, should other vitamins be studied? Do we need further analyses with observational studies of nutritional intake? The Women's Antioxidant and Folic Acid Cardiovascular Study of B complex of vitamins suggested a beneficial effect for AMD. Would further randomized trial be feasible and be warranted, given the preliminary findings of a protective effect of the B vitamins for AMD? These are questions that yet must be answered.

An explosion of genetic information is occurring in the field of AMD.[47,48] Additional pathways have been emerging as potential disease mechanisms from studies of genetic associations. These include pathways that involve inflammatory, complement, and high-density lipoprotein (HDL) cholesterol pathways that potentially are part of AMD pathogenesis. The further analyses of these genetic associations with the dietary intake and the randomized trials of nutrient supplement should give us clues as to the potential for personalized medicine and potential targets for future treatment.

An important step in the research for AMD includes the need for better phenotyping, as AMD is likely to include a number of conditions that may be separated genetically. We also need a better understanding of the functional and structural correlations of the disease process. The technologies for imaging have developed rapidly, and may provide better tools for developing and improving our outcome measures for cataract and AMD outcomes for clinical trials.

Finally, it is important to understand that a modest protective effect on the progression of AMD (and cataract) still would have a significant impact on patient welfare and on cost control given the large and increasing numbers of patients affected. Areas of unmet medical need include finding effective therapy for geographic atrophy and for primary prevention of AMD. Prevention certainly would be favored over intravitreal injections, even though they may be successful in reducing the risk of vision loss. We believe the next decade will witness some major changes in the way we will treat AMD.

Acknowledgments

Disclosure: **E.Y. Chew**, None

References

1. Congdon N, Vingerling JR, Klein BE, et al. Prevalence of cataract and pseudophakia/aphakia among adults in the United States. *Arch Ophthalmol*. 2004;122:487–494.
2. Leske MC, Chylack LT Jr, Wu SY. The Lens Opacities Case-Control Study: risk factors for cataract. *Arch Ophthalmol*. 1991;109:244–251.
3. Seddon JM, Christen WG, Manson JE, et al. The use of vitamin supplements and the risk of cataract among US male physicians. *Am J Public Health*. 1994;84:788–792.
4. Leske MC, Chylack LT, He Q, et al. Antioxidant vitamins and nuclear opacities: the Longitudinal Study of Cataract. *Ophthalmology*. 1998;105:831–836.
5. Mares-Perlman JA, Lyle BJ, Klein R, et al. Vitamin supplement use and incident cataracts in a population-based study. *Arch Ophthalmol*. 2000;118:1556–1563.
6. Jacques PF, Chylack LT Jr, Hankinson SE, et al. Long-term nutrient intake and early age-related nuclear lens opacities. *Arch Ophthalmol*. 2001;119:1009–1019.
7. Kuzniarz M, Mitchell P, Cumming RG, Flood VM. Use of vitamin supplements and cataract: the Blue Mountains Eye Study. *Am J Ophthalmol*. 2001;132:19–26.
8. Teikari JM, Virtamo J, Rautalahti M, et al. Long-term supplementation with alpha-tocopherol and beta-carotene and age-related cataract. *Acta Ophthalmol Scand*. 1997;75:634–640.
9. Christen WG, Manson JE, Glynn RJ, et al. A randomized trial of beta carotene and age-related cataract in US physicians. *Arch Ophthalmol*. 2003;121:372–378.
10. Sperduto RD, Hu TS, Milton RC, et al. The Linxian cataract studies: two nutrition intervention trials. *Arch Ophthalmol*. 1993;111:1246–1253.
11. McNeil JJ, Robman L, Tikellis G, et al. Vitamin E supplementation and cataract. Randomized controlled trial. *Ophthalmology*. 2004;111:75–84.
12. Christen W, Glynn R, Sperduto R, et al. Age-related cataract in a randomized trial of beta-carotene in women. *Ophthalmic Epidemiol*. 2004;11:401–412.
13. Gritz DC, Srinivasan M, Smith SD, et al. The Antioxidants in Prevention of Cataracts Study: effects of antioxidant supplements on cataract progression in South India. *Br J Ophthalmol*. 2006;90:847–851.

14. Clinical Trial of Nutritional Supplements and Age-Related Cataract Study Group, Maraini G, Williams SL, Sperduto RD, et al. A randomized, double-masked, placebo-controlled clinical trial of multivitamin supplementation for age-related lens opacities. Clinical trial of nutritional supplements and age-related cataract report no. 3. *Ophthalmology.* 2008;115:599–607.
15. Christen WG, Glynn RJ, Sesso HD, et al. Age-related cataract in a randomized trial of vitamins E and C in men. *Arch Ophthalmol*. 2010;128:1397–1405.
16. Christen WG, Glynn RJ, Chew EY, Buring JE. Vitamin E and age-related cataract in a randomized trial of women. *Ophthalmology.* 2008;115:822–829.e1.
17. The Age-Related Eye Disease Study Research Group. A randomized, placebo-controlled clinical trial of high-dose supplementation with vitamins C and E and beta carotene for age-related cataract and vision loss. AREDS Report No. 9. *Arch Ophthalmol*. 2001;119:1439–1452.
18. The Age-Related Eye Disease Study Research Group 2. Randomized trial of lutein/zeaxanthin for age-related cataract. AREDS2 Report No. 4. *JAMA Ophthalmol*. 2013;119:1439–1452.
19. Congdon N, O'Colmain B, Klaver CC, et al. Causes and prevalence of visual impairment among adults in the United States. *Arch Ophthalmol*. 2004;122:477–485.
20. Friedman DS, O'Colmain BJ, Muñoz B, et al. Prevalence of age-related macular degeneration in the United States. *Arch Ophthalmol*. 2004;122:564–572.
21. Eye Disease Case-Control Study Group. Antioxidant status and neovascular age related macular degeneration. *Arch Ophthalmol*. 1993;111:104–109, Errata in 1993;111:1499, 1228, and 1366.
22. Seddon JM, Ajani UA, Sperduto RD, et al. Dietary carotenoids, vitamins A, C, and E, and advanced age-related macular degeneration. *JAMA*. 1994;272:1413–1420.
23. Vandenlangenberg GM, Mares-Perlman JA, Klein R, et al. Associations between antioxidant and zinc intake and the 5-year incidence of early age-related maculopathy in the Beaver Dam Eye Study. *Am J Epidemiol*. 1998;148:204–214.
24. Christen WG, Ajani UA, Glynn RJ, et al. Prospective cohort study of antioxidant vitamin supplement use and the risk of age-related maculopathy. *Am J Epidemiol*. 1999;149:476–484.
25. Flood V, Smith W, Wang JJ, et al. Dietary antioxidant intake and incidence of early age-related maculopathy. *Ophthalmology.* 2002;109:2272–2278.
26. Cho E, Seddon JM, Rosner B, et al. Prospective study of intake of fruits, vegetables, vitamins, and carotenoids and risk of age related maculopathy. *Arch Ophthalmol*. 2004;122:883–892.
27. Mares-Perlman JA, Fisher AI, Klein R, et al. Lutein and zeaxanthin in the diet and serum and their relation to age-related maculopathy in the third National Health and Nutrition Examination Survey. *Am J Epidemiol*. 2001;153:424–432.
28. Snellen EL, Verbeek AL, Van Den Hoogen GW, Cruysberg JR, Hoyng CB. Neovascular age-related macular degeneration and its relationship to antioxidant intake. *Acta Ophthalmol Scan*. 2002;80:368–371.
29. Moeller SM, Parekh N, Tinker L, et al. Associations between intermediate age related macular degeneration and lutein and zeaxanthin in the Carotenoids in Age related Eye Disease Study (CAREDS): ancillary study of the Women's Health Initiative. *Arch Ophthalmol*. 2006;124:1151–1162.
30. Sangiovanni JP, Chew EY, Clemons TE, et al. The relationship of dietary carotenoids, vitamin E, and vitamin C with age-related macular degeneration: a case-control study in the Age-Related Eye Disease Study. AREDS report number 22. *Arch Ophthalmol*. 2007;125:1225–1232.
31. Tan JS, Wang JJ, Flood V, Rochtchina E, Smith W, Mitchell P. Dietary antioxidants and the longer-term incidence of age-related macular degeneration: the Blue-Mountains Eye Study. *Ophthalmology.* 2008;115:334–341.
32. Seddon JM, Rosner B, Sperduto RD, et al. Dietary fat and risk for advanced age-related macular degeneration. *Arch Ophthalmol*. 2001;119:1191–1199.
33. Seddon JM, Cote J, Rosner B. Progression of age-related macular degeneration: association with dietary fat, transunsaturated fat, nuts, and fish intake. *Arch Ophthalmol*. 2003;121:1728–1737.
34. Seddon JM, George S, Rosner B. Cigarette smoking, fish consumption, omega-3 fatty acid intake, and associations with age-related macular degeneration: the US Twin Study of Age-Related Macular Degeneration. *Arch Ophthalmol*. 2006;124:995–1001.
35. Chua B, Flood V, Rochtchina E, et al. Dietary fatty acids and the 5-year incidence of age-related maculopathy. *Arch Ophthalmol*. 2006;124:981–986.
36. Augood C, Chakravarthy U, Young I, et al. Oily fish consumption, dietary docosahexaenoic acid and eicosapentaenoic acid intakes, and associations with neovascular age-related macular degeneration. *Am J Clin Nutr.* 2008;88:398–406.
37. Swenor BK, Bressler S, Caulfield L, West SK. The impact of fish and shellfish consumption on age-related macular degeneration. *Ophthalmology.* 2010;117:2395–2401.
38. SanGiovanni JP, Chew EY, Agron E, et al. The relationship of dietary lipid intake with incident age-related macular degeneration: AREDS report number 23. *Arch Ophthalmol*. 2008;126:1274–1279.
39. SanGiovanni JP, Agron E, Clemons TE, Chew EY. Omega-3 long chain polyunsaturated fatty acid intake inversely associated with 12-year progression to advanced age-related macular degeneration. *Arch Ophthalmol*. 2009;127:110–112.
40. SanGiovanni JP, Argon E, Meleth AD, et al. Omega-3 long-chain polyunsaturated fatty acid intake and 12-year incidence of neovascular age-related macular degeneration and central geographic atrophy: a prospective cohort study from the Age-Related Eye Disease Study. *Am J Clin Nutr.* 2009;90:1601–1607.
41. Newsome DA, Swartz M, Leone NC, et al. Oral zinc in macular degeneration. *Arch Ophthalmol*. 1988;106:192–198.
42. The Age-Related Eye Disease Study Research Group. A randomized, placebo-controlled, clinical trial of high-dose supplementation with vitamins C and E, beta carotene, and zinc for age-related macular degeneration and vision loss. AREDS Report No. 8. *Arch Ophthalmol*. 2001;119:1417–1436.
43. Chew EY, Clemons TE, Agrón E, et al. Age-Related Eye Disease Study Research Group. Long-term effects of vitamins c and e, β-carotene, and zinc on age-related macular degeneration: AREDS report no. 35. *Ophthalmology.* 2013;120:1604–1611.
44. Age-Related Eye Disease Study 2 Research Group. Lutein + zeaxanthin and omega-3 fatty acids for age-related macular degeneration: the Age-Related Eye Disease Study 2 (AREDS2) randomized clinical trial. *JAMA*. 2013;309:2005–2015.
45. Christen W, Glynn RJ, Chew EY, et al. Folic acid, pyridoxine, & cyanocobalamin combination treatment and age-related macular degeneration in women. The Women's Antioxidant and Folic Acid Cardiovascular Study. *Arch Intern Med*. 2009;169:335–341.
46. Datiles MB III, Ansari RR, Suh KI, et al. Clinical detection of precataractous lens protein changes using dynamic light scattering. *Arch Ophthalmol*. 2008;126:1687–1693.
47. Gorin MB. Genetic insights into age-related macular degeneration: controversies addressing risk, causality, and therapeutics. *Mol Aspects Med*. 2012;33:467–486.
48. Priya RR, Chew EY, Swaroop A. Genetic studies of age-related macular degeneration: lessons, challenges, and opportunities for disease management. *Ophthalmology.* 2012;119:2526–2536.
