# Supplementary material for: Insights into the nutritional prevention of macular degeneration based on a comparative topic modeling approach
Source: PeerJ Comput Sci. 2024 Mar 20;10:e1940. doi: 10.7717/peerj-cs.1940 (PMC11042009; doi:10.7717/peerj-cs.1940)
Supplement: Supplemental Information 2 [file peerj-cs-10-1940-s002.zip › positive_result_reports/1-s2.0-S0161642020308368-main.pdf]

AMERICAN ACADEMY OF OPHTHALMOLOGY®

Dietary Nutrient Intake and Progression to Late Age-Related Macular Degeneration in the Age-Related Eye Disease Studies 1 and 2

*Elvira Agrón, MA,[1] Julie Mares, PhD,[2] Traci E. Clemons, PhD,[3] Anand Swaroop, PhD,[4] Emily Y. Chew, MD,[1] Tiarnan D.L. Keenan, BM BCh, PhD,[1] for the AREDS and AREDS2 Research Groups**

Purpose: To analyze associations between the dietary intake of multiple nutrients and risk of progression to late age-related macular degeneration (AMD), its subtypes, and large drusen.

Design: Post hoc analysis of 2 controlled clinical trial cohorts: Age-Related Eye Disease Study (AREDS) and AREDS2.

Participants: Eyes with no late AMD at baseline among AREDS participants (n = 4504) and AREDS2 participants (n = 3738) totaled 14 135 eyes. Mean age was 71.0 years (standard deviation, 6.7 years), and 56.5% of patients were women.

Methods: Fundus photographs were collected at annual study visits and graded centrally for late AMD. Dietary intake of multiple nutrients was calculated from food frequency questionnaires.

Main Outcome Measures: Progression to late AMD, geographic atrophy (GA), neovascular AMD, and (separate analyses) large drusen.

Results: Over median follow-up of 10.2 years, of the 14 135 eyes, 32.7% progressed to late AMD. For 9 nutrients, intake quintiles 4 or 5 (vs. 1) were associated significantly ($P \leq 0.0005$) with decreased risk of late AMD: vitamin A, vitamin B6, vitamin C, folate, β-carotene, lutein and zeaxanthin, magnesium, copper, and alcohol. For 3 nutrients, quintiles 4 or 5 were associated significantly with increased risk: saturated fatty acid, monounsaturated fatty acid, and oleic acid. Similar results were observed for GA. Regarding neovascular AMD, 9 nutrients were associated nominally with decreased risk—vitamin A, vitamin B6, β-carotene, lutein and zeaxanthin, magnesium, copper, docosahexaenoic acid, omega-3 fatty acid, and alcohol—and 3 nutrients were associated with increased risk—saturated fatty acid, monounsaturated fatty acid, and oleic acid. In separate analyses (n = 5399 eyes of 3164 AREDS participants), 12 nutrients were associated nominally with decreased risk of large drusen.

Conclusions: Higher dietary intake of multiple nutrients, including minerals, vitamins, and carotenoids, is associated with decreased risk of progression to late AMD. These associations are stronger for GA than for neovascular AMD. The same nutrients also tend to show protective associations against large drusen development. Strong genetic interactions exist for some nutrient–genotype combinations, particularly omega-3 fatty acids and *CFH.* These data may justify further research into underlying mechanisms and randomized trials of supplementation. *Ophthalmology 2021;128:425-442 Published by Elsevier on behalf of the American Academy of Ophthalmology*

Supplemental material available at www.aaojournal.org.

Age-related macular degeneration (AMD) is the leading cause of legal blindness in developed countries.[1,2] It arises from a complex interplay among aging, genetic, and environmental factors, including dietary factors.[3–5] Late AMD, the stage with the potential for blindness, occurs in 2 forms: geographic atrophy (GA) and neovascular AMD. Neovascular AMD requires repeated intravitreal injection of anti–vascular endothelial growth factor drugs, whereas no treatments are available clinically for GA, either to slow down enlargement of the affected area or to restore lost vision. Hence, preventative approaches are attractive for both subtypes of late disease.

Oral supplementation with specific combinations of antioxidants and minerals is known to decrease progression from intermediate to late AMD, particularly neovascular AMD.[6,7] However, it is possible that alternative or additional protection against progression to late AMD may be derived from specific nutrients or other bioactive ingredients in food, which may comprise additional modifiable risk factors for AMD. Some observational studies have observed that higher intakes of particular dietary nutrients are associated with decreased or increased risk of early or late AMD.[5,8,9] However, inconsistencies exist among some of these studies. The most consistent findings have included decreased risk of late AMD associated with higher dietary intake of omega-3 fatty acids[10] and the carotenoids lutein and zeaxanthin.[11]

Recent analyses of the Age-Related Eye Disease Study (AREDS) and AREDS2 demonstrated that closer adherence

Published by Elsevier on behalf of the American Academy of Ophthalmology https://doi.org/10.1016/j.ophtha.2020.08.018
ISSN 0161-6420/20

to a Mediterranean dietary pattern, and to its fish component, was associated highly with decreased progression to late AMD, particularly GA.[12] Additional analysis of the biologically active nutrients may provide more insights into the molecular basis of disease, with implications for potential preventative strategies.

The AREDS and AREDS2 were multicenter phase 3 randomized controlled trials (RCTs) designed to assess the effects of nutritional supplementation on AMD progression.[13,14] Their primary outcome was progression to late AMD. In both studies, validated food frequency questionnaires (FFQs) were administered at study enrollment, which allowed the estimation of dietary intake levels for multiple nutrients. This permits a broad and unbiased approach to analyzing potential relationships between dietary intake and AMD progression for many nutrients. Owing to the comprehensive nature of this approach, some findings may be novel, and others may be consistent with those from previous studies.

The main aim of this study was to use data from the AREDS and AREDS2 to examine potential associations between the dietary intake of multiple nutrients and progression to late AMD, including separate analyses of GA and neovascular AMD. Additional aims included analyzing similar associations for the development of large drusen (using AREDS data) and examining potential interactions between nutrient intake levels and AMD genotype for progression to late AMD.

Methods

Study Populations

The study designs for the AREDS and AREDS2 have been described previously.[13,14] In the AREDS, 4757 participants (55−80 years of age) were recruited (from 1992−1998) at 11 United States retinal specialty clinics and enrolled into AMD categories (no AMD to unilateral late AMD). In the AREDS2, 4203 participants (50−85 years of age) with bilateral large drusen or unilateral late AMD were recruited (from 2006−2008) at 82 United States retinal specialty clinics. Institutional review board approval was obtained at each site, and written informed consent was obtained from all participants. The research adhered to the tenets of the Declaration of Helsinki and, for AREDS2, complied with the Health Insurance Portability and Accountability Act.

Study Procedures

The AREDS participants were assigned randomly to placebo, antioxidants, zinc, or the combination. The RCT component lasted 5 years. The AREDS2 participants were assigned randomly to receive the supplements that lowered risk of AMD progression in the AREDS, either (1) alone or with additional (2) lutein and zeaxanthin, (3) docosahexaenoic acid (DHA) plus eicosapentaenoic acid (EPA), or (4) the combination. Again, the RCT component lasted 5 years. In both studies, at baseline and annual visits, eye examinations were performed, and color fundus photographs were captured and graded centrally at the University of Wisconsin Fundus Photograph Reading Center.[15]

In the AREDS, after close-out at 5 years, epidemiologic follow-up started immediately for 3549 of the 4203 surviving AREDS participants. In the AREDS2, after close-out at 5 years, 2923 participants underwent reassessment at 10 years (AREDS2 10-year follow-on study). In the AREDS (for the duration) and the AREDS2 (for the first 5 years), progression to late AMD (including subtype of late AMD) was defined by fundus photograph grades, history of treatment for neovascular AMD, or both, as described previously.[13,14] In brief, progression to late AMD was defined as an eye that did not have late AMD at study baseline but did have late AMD at a follow-up study visit. Late AMD was defined as the presence of GA (central or noncentral) on fundus photography, the presence of neovascular AMD on fundus photography, or a history of treatment for neovascular AMD. In the AREDS, GA and neovascular AMD were assessed independently. Thus, if an eye progressed to neovascular AMD, it was still assessed for additional progression to GA at subsequent study visits, and vice versa. By contrast, in the AREDS2, this was not the case. In the AREDS2, if an eye progressed to GA, it was still assessed for additional progression to neovascular AMD; however, if an eye progressed to neovascular AMD, it was no longer assessed for additional progression to GA. Similarly, progression to large drusen was defined as an eye that did not demonstrate either late AMD or large drusen at study baseline but did demonstrate large drusen at a follow-up visit (by reading center grading of fundus photographs). No reversal was possible for any outcome. For example, if an eye progressed to GA during follow-up, subsequent grades of GA presence or absence for the same eye did not contribute to the analyses. In the AREDS2, for eyes with no late AMD at the 5-year close-out, progression to late AMD by 10 years was defined (without subtype analysis) as described in the Supplemental Materials (available at www.aaojournal.org).

Assessment of the Intake of Dietary Nutrients

In both studies, FFQs were administered to all participants at randomization. The AREDS FFQ, a 90-item, semiquantitative modified Block FFQ, and its validation have been described previously.[16] The AREDS2 FFQ, a 131-item, semiquantitative Harvard FFQ, and its validation have also been described previously.[17,18] In both FFQs, participants were asked how often, on average, they consumed each food or beverage item during the preceding year. The FFQs were processed at the University of Minnesota Nutrition Coordinating Center to estimate, for each participant, the daily dietary intake of 38 nutrients in the AREDS and 44 nutrients in the AREDS2. In the AREDS, these estimates excluded any additional intake from oral supplements. The same was true for most nutrients in AREDS2. However, for a minority of nutrients in AREDS2 (α-carotene, β-cryptoxanthin, lutein and zeaxanthin, lycopene, selenium, and those related to fatty acids, including DHA and EPA), the estimates included any additional intake from oral supplements. Each nutrient was divided by total calorie intake to represent nutrient intake density, and intake quintiles were calculated (separately for each cohort and for men and women), with quintile 5 representing the highest intake. Quintiles were used to provide easily interpretable hazard ratios.

Genotype Analysis

As part of AREDS and AREDS2, 2889 (AREDS) and 1826 (AREDS2) participants consented to genotype analysis. Single nucleotide polymorphisms were analyzed using a custom Illumina HumanCoreExome array (Illumina, Inc).[19] The AMD genetic risk score, a weighted risk score for late AMD, was calculated for each participant.[19] Four single nucleotide polymorphisms at 3 loci (with highest attributable risk of late AMD) were also selected: *ARMS2* rs10490924, *CFH* rs10922109 and rs1061170, and *C3* rs2230199.

Participant Cohorts and Statistical Methods

The eligibility criteria were: eyes without late AMD at baseline in participants with at least 2 study visits. In the combined AREDS and AREDS2 cohort, for each of the 33 nutrients for which intake was measured in both AREDS and AREDS2, multivariate proportional hazards regression analyses were performed for the outcomes of progression to late AMD, GA, and neovascular AMD, according to intake quintiles (with quintile 1 as the reference). *P* values for trend were calculated by regression using the energy-adjusted nutrient intake values (as continuous variables). The proportional hazards assumption was tested in all cases. In the one situation where the assumption was not met (for the cohort variable in the combined cohort analysis of neovascular AMD), stratified proportional hazards regression was performed instead.[20] These analyses were repeated for the individual AREDS (38 nutrients) and AREDS2 (44 nutrients) cohorts, considered separately. The Bonferroni level of significance was $P = 0.0005$ for the combined cohort. We considered that *P* values higher than that level could be the result of chance, given the large number of analyses; however, we did not disregard these associations, because higher *P* values for individual nutrients could also reflect how well dietary intake of those nutrients was captured on FFQs and the limitations of nutrient databases, rather than their physiologic importance.

The regression analyses were also performed (1) with adjustment for treatment assignment (i.e., the oral supplements or placebo to which the participants were randomized) and (2) including the interaction term between nutrient intake (treated continuously) and treatment assignment. In AREDS, this included antioxidants as main effect and zinc as main effect, and in AREDS2, this included DHA and EPA as main effect and lutein and zeaxanthin as main effect. For these analyses, follow-up was limited to the duration of the treatment assignment (5 years). The regression analyses were also repeated with adjustment for Centrum multivitamin use (in AREDS only, because Centrum (Pfizer Inc) use was almost universal in AREDS2).

In the AREDS, similar analyses were performed for the outcome of progression to large drusen. For these analyses, the eligibility criteria were: eyes without large drusen or late AMD at baseline in participants with at least 2 study visits.

For the nutrients with $P \leq 0.01$ for altered progression to late AMD outcomes in quintile 4 or 5 (versus quintile 1), the regression analyses were repeated, including the interaction term between nutrient intake (treated continuously) and genotype. For the nutrient−genotype combinations with *P* values for interaction of 0.01 or less, the regression analyses were performed separately for each level of the genetic characteristic.

In all cases, the unit of analysis was the eye and the analyses were adjusted for age, gender, smoking, total calorie intake, body mass index (BMI; for AREDS only), and correlation between eyes. Age, smoking, and BMI were included as risk factors known to be associated with progression to late AMD.[21,22] Gender was included as an important biological variable. Total calorie intake was included to decrease confounding and reduce extraneous variation from factors like physical activity and metabolic efficiency.[23,24] Age and total calorie intake were treated as continuous variables, whereas gender, smoking status (assessed by self-report), and BMI were treated as categorical variables (as defined in Table 1). Adjustment for correlation between eyes was made in SAS software version 9.4 (SAS Institute, Inc) by using the robust sandwich estimate for the covariance matrix in the Wald tests.[25] In analyses of the combined AREDS and AREDS2 cohort, adjustment was also made for the cohort. To avoid overadjustment, baseline AMD severity was not included in the models because it was associated highly with intake for most nutrients; however, additional analyses were conducted in which eyes with baseline AMD severity of 1 through 6 versus 7 or 8 on the AREDS 9-step scale[26] were considered separately.

To aid interpretation of the results, correlation analyses were conducted to explore potential correlations between the intake of each nutrient and every other nutrient using the Pearson correlation coefficient. All analyses were conducted using SAS software version 9.4 (SAS Institute, Inc).

Results

Participant Cohorts: Baseline Characteristics

The combined AREDS and AREDS2 cohort contained 14 135 eligible eyes of 8130 participants. Their characteristics are shown in Table 1, and their dietary intake is shown in Tables S1 and S2 (available at www.aaojournal.org). The numbers of eyes that progressed to late AMD, GA, or neovascular AMD by final follow-up (median, 10.2 years) were 4624 (32.7%), 2246 (16.1%), and 2100 (14.9%), respectively. Considered separately, the AREDS cohort comprised 8235 eyes of 4504 participants and the AREDS2 cohort comprised 6111 eyes of 3738 participants. The numbers of eyes that progressed to late AMD were 1744 (21.2%; median follow-up, 10.1 years) and 2914 (47.7%; median follow-up, 10.4 years), respectively.

For the analyses of progression to large drusen in the AREDS, the cohort contained 5399 eligible eyes of 3164 participants. In this cohort, the number of eyes that progressed to large drusen by final follow-up (median, 10.1 years) was 1431 (26.5%).

Proportional Hazards Regression Analyses: Progression to Late Age-Related Macular Degeneration According to the Intake of Individual Dietary Nutrients

The results of the proportional hazards regression analyses in the combined AREDS and AREDS2 cohort are shown in Figure 1 and Table 2. The proportional hazards assumptions were met in all cases; the only exception was for the cohort variable in the combined cohort analysis of neovascular AMD, so for these analyses, the results of stratified proportional hazards regression are shown instead. Regarding progression to late AMD, for 9 of the 33 nutrients examined, intake quintile 4, 5, or both (with quintile 1, indicating lowest intake, as reference) was associated significantly with decreased risk at the Bonferroni-adjusted level of $P = 0.0005$: vitamin A, vitamin B6, vitamin C, folate, β-carotene, lutein and zeaxanthin, magnesium, copper, and alcohol. For an additional 8 nutrients, association with decreased risk was present at the nominal level ($P > 0.0005$ and $P < 0.05$): thiamine, niacin, β-cryptoxanthin, calcium, iron, DHA, EPA, and omega-3 fatty acids (DHA and EPA). Conversely, for 3 of the nutrients, intake quintile 4, 5, or both was associated significantly with increased risk of late AMD at the Bonferroni-adjusted level: total saturated fatty acid, total monounsaturated fatty acid (MUFA), and oleic acid. For an additional 2 nutrients, association with increased risk was present at the nominal level: cholesterol and linoleic acid.

Regarding progression to GA, for 7 nutrients, intake quintile 4, 5, or both was associated significantly with decreased risk at the Bonferroni-adjusted level: vitamin A, vitamin C, folate, β-carotene, magnesium, copper, and alcohol. For an additional 13 nutrients, association with decreased risk was present at the nominal level: thiamin, riboflavin, niacin, vitamin B6, vitamin B12, β-cryptoxanthin, lycopene, calcium, iron, zinc, DHA, EPA, and omega-3 fatty acids. Conversely, for 2 of the nutrients, intake quintile 4, 5, or both was associated significantly with increased risk at the

Table 1. Participant Demographics at Baseline

	Combined Cohort	Age-Related Eye Disease Study Cohort	Age-Related Eye Disease Study 2 Cohort
No. of participants	8130*	4504	3738
Mean age (SD), yrs	71.0 (6.7)	69.3 (5.1)	72.9 (7.7)
Female gender, no. (%)	4593 (56.5)	2526 (56.1)	2129 (57.0)
Smoking status, no. (%)			
Never	3588 (44.1)	2021 (44.9)	1626 (43.5)
Former	3963 (48.7)	2142 (47.6)	1870 (50.0)
Current	579 (7.1)	341 (7.6)	242 (6.5)
Body mass index (kg/m^2), no. (%)			
≤25	–	1469 (32.6)	–
>25 and ≤30	–	1889 (41.9)	–
>30		1146 (25.4)	
AMD severity category 3-4 (AREDS) or ≥7 in worse eye (AREDS2), no. (%)			
No		2101 (46.6)	887 (23.7)
Yes	–	2403 (53.4)	2851 (76.3)
Follow-up time, yrs			
Mean (SD)	8.8 (3.0)	9.1 (2.8)	8.6 (3.2)
Median	10.2	10.1	10.4
No. of participants with genetic data	4476	2854	1718
Genetic risk score group, no. (%)			
Low risk group†	1183 (26.4)	979 (34.3)	226 (13.2)
Quartiles 1-2	1659 (37.1)	1086 (38.1)	610 (35.5)
Quartiles 3-4	1634 (36.5)	789 (27.6)	882 (51.3)
ARMS2 risk alleles (rs10490924), no. (%)			
0 (GG)	2071 (46.3)	1453 (50.9)	665 (38.7)
1 (GT)	1808 (40.4)	1106 (38.8)	737 (42.9)
2 (TT)	597 (13.3)	295 (10.3)	316 (18.4)
CFH protective alleles (rs10922109), no. (%)			
0 (CC)	2398 (53.6)	1300 (45.6)	1145 (66.6)
1 (CA)	1637 (36.6)	1187 (41.6)	486 (28.3)
2 (AA)	441 (9.9)	367 (12.9)	87 (5.1)
CFH risk alleles (rs1061170), no. (%)			
0 (TT)	1140 (25.5)	866 (30.3)	299 (17.4)
1 (TC)	1998 (44.6)	1305 (45.7)	734 (42.7)
2 (CC)	1338 (29.9)	683 (23.9)	685 (39.9)
C3 risk alleles (rs2230199), no. (%)			
0 (CC)	2542 (56.8)	1672 (58.6)	923 (53.7)
1 (CG)	1656 (37.0)	1036 (36.3)	658 (38.3)
2 (GG)	278 (6.2)	146 (5.1)	137 (8.0)

AMD = age-related macular degeneration; AREDS = Age-Related Eye Disease Study; *ARMS2* = age-related maculopathy susceptibility 2 gene; *CFH* = complement factor H gene; GA = geographic atrophy; SD = standard deviation.
*In the combined cohort, the 112 participants who were in both cohorts were counted only once (using the data from AREDS2).
†Low-risk genetic risk score group: participants whose genetic risk score was less than or equal to the mean genetic risk score of a control population without late AMD.

Bonferroni-adjusted level: saturated fatty acid and oleic acid. For an additional 2 nutrients, association with increased risk was present at the nominal level: MUFA and linoleic acid.

For progression to neovascular AMD, no nutrients had intake quintile 4, 5, or both associated significantly with altered risk at the Bonferroni-adjusted level. For 9 nutrients, association with decreased risk was present at the nominal level: vitamin A, vitamin B6, β-carotene, lutein and zeaxanthin, magnesium, copper, DHA, omega-3 fatty acid, and alcohol. Conversely, for 3 nutrients, association with increased risk was present at the nominal level: saturated fatty acid, monounsaturated fatty acid, and oleic acid.

The proportional hazards regression analyses were also performed separately on the AREDS cohort alone and the AREDS2 cohort alone. The results are shown in Figures 2 and 3 and in Table S3 (available at www.aaojournal.org). Of note, in the AREDS2, higher intake of the DHA plus docosapentaenoic acid (DPA) plus EPA variable was associated significantly with decreased risk of GA, with low hazard ratios, but not neovascular AMD. Intake of this variable was not measured in the AREDS (so is not included in the results of the combined cohort). In addition, the results for DHA, EPA, and DHA plus EPA were each significant in the AREDS (associated with decreased risk of late AMD, GA, and neovascular AMD) but not in the AREDS2. This may relate to the fact that DHA and EPA supplementation was assigned to half of the AREDS2 participants, other reasons discussed in detail below, or a combination thereof, including the potential importance of DPA, very long-chain polyunsaturated fatty acids (VLC-PUFAs), or both.

The analyses of the AREDS cohort alone were repeated separately for eyes with baseline AMD severity of 1 through 6 versus 7 or 8 on the AREDS 9-step scale (Fig S1, available at www.aaojournal.org). In general, for most nutrients with protective associations, the hazards ratios were stronger (further

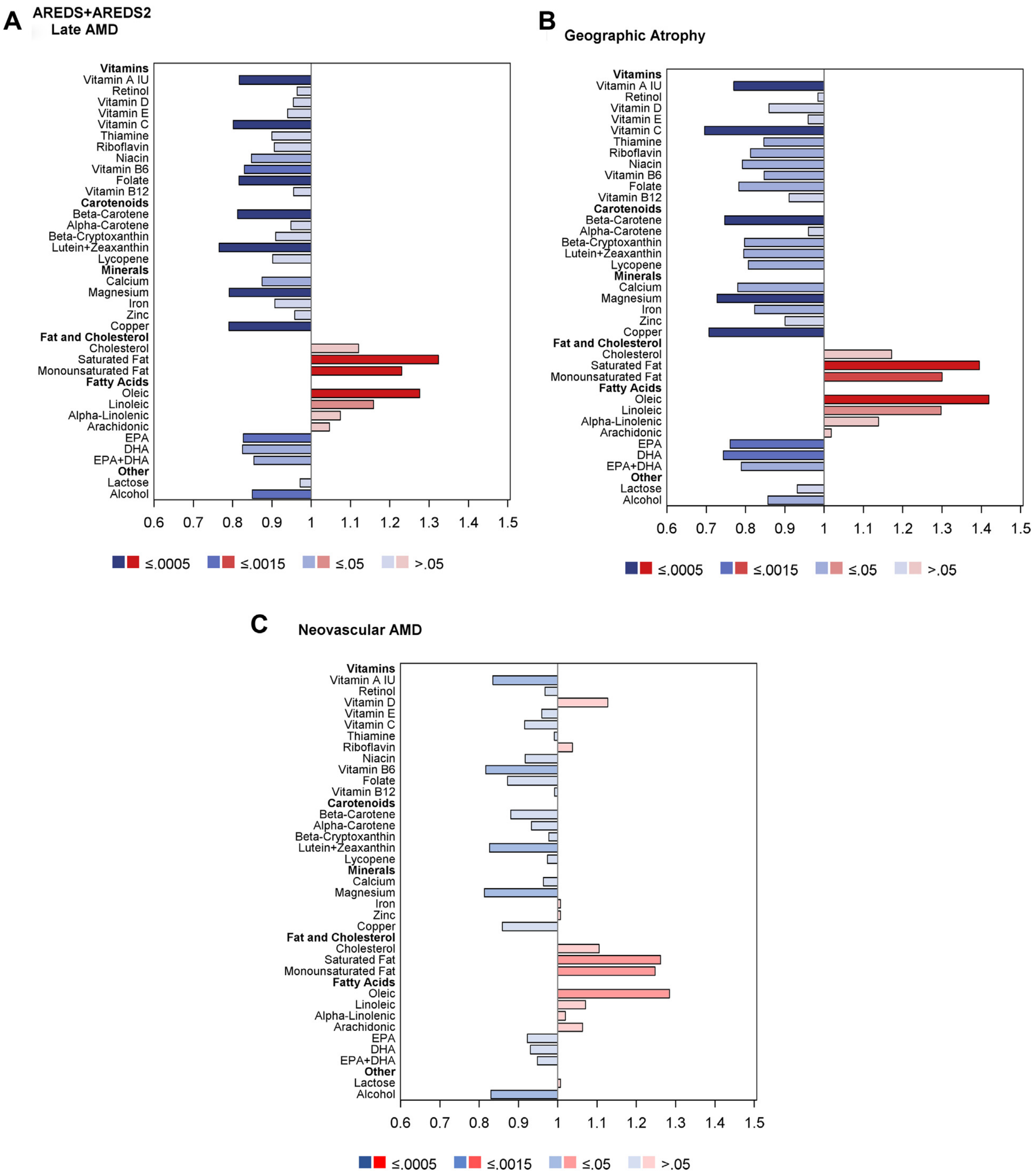

Figure 1. Butterfly plots showing proportional hazards regression modeling of progression to late age-related macular degeneration (AMD) outcomes in the combined Age-Related Eye Disease Study (AREDS) and AREDS2 cohort: (**A**) late AMD, (**B**) geographic atrophy, and (**C**) neovascular AMD. For each nutrient, the hazard ratio of dietary intake quintile 5 (with quintile 1 as reference) is shown on the x-axis, with protective associations in blue and harmful associations in red; smaller *P* values are denoted by darker colors. DHA = docosahexaenoic acid; EPA = eicosapentaenoic acid; IU = international unit.

from 1) and the *P* values more highly significant in the eyes with baseline severity of 1 through 6, whereas the hazard ratios were generally weaker (closer to 1) and the *P* values less highly significant in the eyes with baseline severity of 7 or 8. This was particularly true for DHA and EPA for the outcomes of late AMD and neovascular AMD.

Further analyses that included adjustment for treatment assignment demonstrated very similar results to the original analyses, for both the AREDS and AREDS2 cohorts considered separately. In addition, analyses that included the interaction term between nutrient intake and treatment assignment showed no significant interactions at the Bonferroni-adjusted level. Additional

Table 2. Results of the Proportional Hazards Regression Modeling of Progression to Late Age-Related Macular Degeneration Outcomes, According to Quintiles of Nutrient Intake in the Combined Age-Related Eye Disease Study and Age-Related Eye Disease Study 2 Cohort: Hazard Ratios and *P* Values

	Late AMD			Geographic Atrophy			Neovascular AMD		
**Nutrient**	*Q4 vs Q1**	*Q5 vs Q1**	*P*†	*Q4 vs Q1**	*Q5 vs Q1**	*P*†	*Q4 vs Q1**	*Q5 vs Q1**	P†
Vitamin A (IU)	0.80 (0.72, 0.89)<br><.0001	0.82 (0.74, 0.91)<br>0.0001	0.005	0.76 (0.65, 0.87)<br>0.0002	0.77 (0.66, 0.89)<br>0.0005	0.001	0.85 (0.73, 0.98)<br>0.022	0.83 (0.72, 0.96)<br>0.013	0.20
Retinol	0.97 (0.86, 1.08)<br>0.53	0.96 (0.86, 1.08)<br>0.54	0.61	0.97 (0.83, 1.14)<br>0.70	0.98 (0.84, 1.16)<br>0.85	0.61	0.98 (0.84, 1.14)<br>0.82	0.97 (0.83, 1.13)<br>0.68	0.75
Vitamin D	0.94 (0.84, 1.05)<br>0.25	0.95 (0.86, 1.06)<br>0.40	0.16	0.91 (0.78, 1.06)<br>0.21	0.86 (0.74, 1.00)<br>0.053	0.05	1.07 (0.92, 1.25)<br>0.36	1.13 (0.97, 1.31)<br>0.12	0.76
Vitamin E	0.97 (0.87, 1.10)<br>0.67	0.94 (0.83, 1.06)<br>0.33	0.89	1.02 (0.85, 1.21)<br>0.86	0.96 (0.80, 1.14)<br>0.64	0.51	1.03 (0.88, 1.21)<br>0.72	0.96 (0.81, 1.14)<br>0.63	0.78
Vitamin C	0.79 (0.71, 0.87)<br><.0001	0.80 (0.72, 0.89)<br><.0001	<.0001	0.71 (0.62, 0.83)<br><.0001	0.70 (0.60, 0.81)<br><.0001	<.0001	0.87 (0.75, 1.00)<br>0.056	0.92 (0.79, 1.06)<br>0.25	0.09
Thiamine	0.88 (0.79, 0.97)<br>0.015	0.90 (0.81, 1.00)<br>0.057	0.26	0.87 (0.75, 1.01)<br>0.068	0.85 (0.73, 0.99)<br>0.033	0.08	1.00 (0.86, 1.16)<br>0.99	0.99 (0.85, 1.16)<br>0.91	0.85
Riboflavin	0.91 (0.82, 1.01)<br>0.088	0.91 (0.81, 1.01)<br>0.078	0.56	0.81 (0.70, 0.94)<br>0.005	0.81 (0.70, 0.95)<br>0.008	0.07	1.07 (0.92, 1.25)<br>0.36	1.04 (0.89, 1.21)<br>0.64	0.54
Niacin	0.87 (0.78, 0.97)<br>0.012	0.85 (0.76, 0.95)<br>0.005	0.26	0.86 (0.74, 1.00)<br>0.044	0.79 (0.68, 0.93)<br>0.004	0.08	0.89 (0.77, 1.04)<br>0.14	0.92 (0.79, 1.07)<br>0.27	0.66
Vitamin B6	0.82 (0.73, 0.92)<br>0.0004	0.83 (0.74, 0.93)<br>0.001	0.05	0.79 (0.68, 0.92)<br>0.003	0.85 (0.73, 0.99)<br>0.035	0.03	0.85 (0.73, 0.98)<br>0.030	0.82 (0.70, 0.95)<br>0.010	0.10
Folate	0.82 (0.74, 0.92)<br>0.0004	0.82 (0.73, 0.91)<br>0.0002	0.005	0.71 (0.61, 0.82)<br><.0001	0.78 (0.67, 0.91)<br>0.002	0.0002	0.92 (0.80, 1.07)<br>0.29	0.87 (0.75, 1.02)<br>0.077	0.18
Vitamin B12	0.93 (0.83, 1.04)<br>0.19	0.96 (0.85, 1.07)<br>0.43	0.30	0.82 (0.70, 0.96)<br>0.011	0.91 (0.78, 1.07)<br>0.24	0.21	0.98 (0.84, 1.14)<br>0.77	0.99 (0.85, 1.15)<br>0.92	0.67
β-Carotene	0.78 (0.70, 0.87)<br><.0001	0.81 (0.73, 0.90)<br>0.0001	0.006	0.74 (0.63, 0.85)<br><.0001	0.75 (0.64, 0.87)<br>0.0001	0.001	0.83 (0.72, 0.97)<br>0.016	0.88 (0.76, 1.02)<br>0.088	0.23
α-Carotene	0.92 (0.83, 1.03)<br>0.14	0.95 (0.85, 1.05)<br>0.31	0.17	0.90 (0.77, 1.05)<br>0.18	0.96 (0.83, 1.11)<br>0.59	0.27	1.01 (0.87, 1.17)<br>0.92	0.93 (0.81, 1.08)<br>0.36	0.39
β-Cryptoxanthin	0.87 (0.78, 0.97)<br>0.011	0.91 (0.82, 1.01)<br>0.080	0.004	0.82 (0.70, 0.95)<br>0.009	0.80 (0.68, 0.93)<br>0.004	0.0003	0.94 (0.81, 1.09)<br>0.39	0.98 (0.84, 1.14)<br>0.77	0.38
Lutein and Zeaxanthin	0.74 (0.67, 0.83)<br><.0001	0.77 (0.69, 0.85)<br><.0001	0.08	0.77 (0.66, 0.90)<br>0.0007	0.80 (0.68, 0.93)<br>0.004	0.03	0.78 (0.68, 0.91)<br>0.0015	0.83 (0.71, 0.96)<br>0.013	0.42
Lycopene	0.96 (0.86, 1.07)<br>0.50	0.90 (0.81, 1.01)<br>0.064	0.005	0.82 (0.71, 0.95)<br>0.010	0.81 (0.70, 0.94)<br>0.005	0.002	1.03 (0.89, 1.19)<br>0.68	0.97 (0.84, 1.13)<br>0.73	0.58
Calcium	0.99 (0.89, 1.10)<br>0.83	0.87 (0.78, 0.98)<br>0.020	0.20	0.90 (0.77, 1.04)<br>0.15	0.78 (0.67, 0.91)<br>0.002	0.10	1.10 (0.95, 1.27)<br>0.22	0.96 (0.82, 1.13)<br>0.65	0.59
Magnesium	0.86 (0.77, 0.95)<br>0.005	0.79 (0.71, 0.88)<br><.0001	<.0001	0.83 (0.71, 0.96)<br>0.013	0.73 (0.62, 0.85)<br><.0001	<.0001	0.88 (0.76, 1.02)<br>0.084	0.81 (0.70, 0.95)<br>0.007	0.04
Iron	0.85 (0.77, 0.95)<br>0.004	0.91 (0.81, 1.01)<br>0.081	0.28	0.80 (0.69, 0.93)<br>0.004	0.82 (0.71, 0.96)<br>0.011	0.07	0.97 (0.83, 1.12)<br>0.66	1.01 (0.87, 1.17)<br>0.92	0.88
Zinc	0.95 (0.85, 1.05)<br>0.31	0.96 (0.86, 1.07)<br>0.45	0.89	0.86 (0.74, 0.99)<br>0.042	0.90 (0.77, 1.05)<br>0.17	0.47	1.05 (0.91, 1.22)<br>0.47	1.01 (0.87, 1.17)<br>0.93	0.70
Copper	0.83 (0.74, 0.93)<br>0.0015	0.79 (0.70, 0.89)<br>0.0001	0.07	0.77 (0.66, 0.90)<br>0.0009	0.71 (0.60, 0.83)<br><.0001	0.18	0.84 (0.72, 0.98)<br>0.027	0.86 (0.73, 1.01)<br>0.061	0.26
Cholesterol	1.12 (1.00, 1.26)<br>0.046	1.12 (1.00, 1.26)<br>0.053	0.01	1.14 (0.98, 1.34)<br>0.10	1.17 (1.00, 1.37)<br>0.051	0.07	1.05 (0.90, 1.23)<br>0.51	1.11 (0.95, 1.29)<br>0.21	0.07

Table 2. (Continued.)

Nutrient	Late AMD			Geographic Atrophy			Neovascular AMD		
	*Q4 vs Q1**	*Q5 vs Q1**	*P*†	*Q4 vs Q1**	*Q5 vs Q1**	*P*†	*Q4 vs Q1**	*Q5 vs Q1**	P†
Saturated fat	1.12 (1.01, 1.24)<br>0.038	1.32 (1.19, 1.48)<br><.0001	<.0001	1.17 (1.00, 1.36)<br>0.049	1.39 (1.20, 1.63)<br><.0001	<.0001	1.06 (0.91, 1.23)<br>0.44	1.26 (1.09, 1.46)<br>0.002	0.01
Monounsaturated fat	1.15 (1.03, 1.29)<br>0.012	1.23 (1.10, 1.38)<br>0.0002	0.001	1.23 (1.05, 1.43)<br>0.009	1.30 (1.11, 1.52)<br>0.0009	<.0001	1.16 (0.99, 1.35)<br>0.061	1.25 (1.07, 1.46)<br>0.005	0.02
Oleic acid	1.13 (1.01, 1.26)<br>0.029	1.28 (1.14, 1.43)<br><.0001	0.001	1.17 (1.00, 1.37)<br>0.057	1.42 (1.22, 1.66)<br><.0001	<.0001	1.15 (0.98, 1.34)<br>0.086	1.28 (1.10, 1.50)<br>0.002	0.02
Linoleic acid	1.03 (0.92, 1.15)<br>0.58	1.16 (1.03, 1.30)<br>0.014	0.02	1.11 (0.95, 1.30)<br>0.19	1.30 (1.10, 1.52)<br>0.002	0.003	0.97 (0.83, 1.12)<br>0.65	1.07 (0.92, 1.25)<br>0.39	0.50
α-Linolenic acid	1.07 (0.96, 1.19)<br>0.20	1.07 (0.96, 1.20)<br>0.20	0.03	1.06 (0.91, 1.23)<br>0.45	1.14 (0.98, 1.32)<br>0.089	<.0001	1.09 (0.94, 1.26)<br>0.26	1.02 (0.88, 1.18)<br>0.80	0.79
Arachidonic acid	1.01 (0.90, 1.14)<br>0.89	1.05 (0.93, 1.18)<br>0.46	0.37	1.01 (0.86, 1.19)<br>0.90	1.02 (0.86, 1.20)<br>0.84	0.89	0.95 (0.81, 1.12)<br>0.56	1.06 (0.90, 1.25)<br>0.46	0.26
EPA	0.85 (0.76, 0.96)<br>0.006	0.83 (0.74, 0.93)<br>0.0009	0.85	0.80 (0.69, 0.93)<br>0.004	0.76 (0.65, 0.89)<br>0.0008	0.87	0.91 (0.78, 1.06)<br>0.23	0.92 (0.79, 1.07)<br>0.30	0.06
DHA	0.84 (0.75, 0.95)<br>0.004	0.83 (0.73, 0.93)<br>0.002	0.38	0.78 (0.67, 0.92)<br>0.002	0.74 (0.63, 0.88)<br>0.0006	0.05	0.85 (0.73, 1.00)<br>0.044	0.93 (0.79, 1.09)<br>0.38	0.16
EPA and DHA	0.86 (0.76, 0.96)<br>0.008	0.85 (0.76, 0.96)<br>0.009	0.79	0.81 (0.70, 0.95)<br>0.01	0.79 (0.67, 0.93)<br>0.005	0.30	0.85 (0.72, 0.99)<br>0.038	0.95 (0.81, 1.11)<br>0.51	0.09
Lactose	1.02 (0.91, 1.14)<br>0.73	0.97 (0.87, 1.09)<br>0.61	0.79	0.98 (0.84, 1.15)<br>0.85	0.93 (0.79, 1.09)<br>0.39	0.58	1.11 (0.96, 1.29)<br>0.16	1.01 (0.86, 1.18)<br>0.93	0.91
Alcohol	0.79 (0.72, 0.88)<br><.0001	0.85 (0.77, 0.93)<br>0.0008	0.62	0.74 (0.65, 0.85)<br><.0001	0.86 (0.75, 0.98)<br>0.023	0.94	0.84 (0.73, 0.96)<br>0.009	0.83 (0.73, 0.95)<br>0.006	0.73

AMD = age-related macular degeneration; AREDS = Age-Related Eye Disease Study; CI = confidence interval; DHA = docosahexaenoic acid; EPA = eicosapentaenoic acid; HR = hazard ratio; IU = international units; Q = quintile.

*Hazard ratios and 95% confidence intervals, with accompanying *P* values shown underneath. The results are shown in comparison to quintile 1 (reference), following adjustment for age, sex, smoking status, total calorie intake, body mass index (for AREDS participants only), and correlation between eyes. For the AREDS2 data, the results for late AMD are based on events over the full study period (i.e., including the AREDS2 10-year follow-on study), while the results for geographic atrophy and neovascular AMD are based on events up until the AREDS2 close-out (median 5 years). This is because, in the AREDS2 10-year follow-on study, late AMD subtype information was not captured from all data sources.

†*P* trend values.

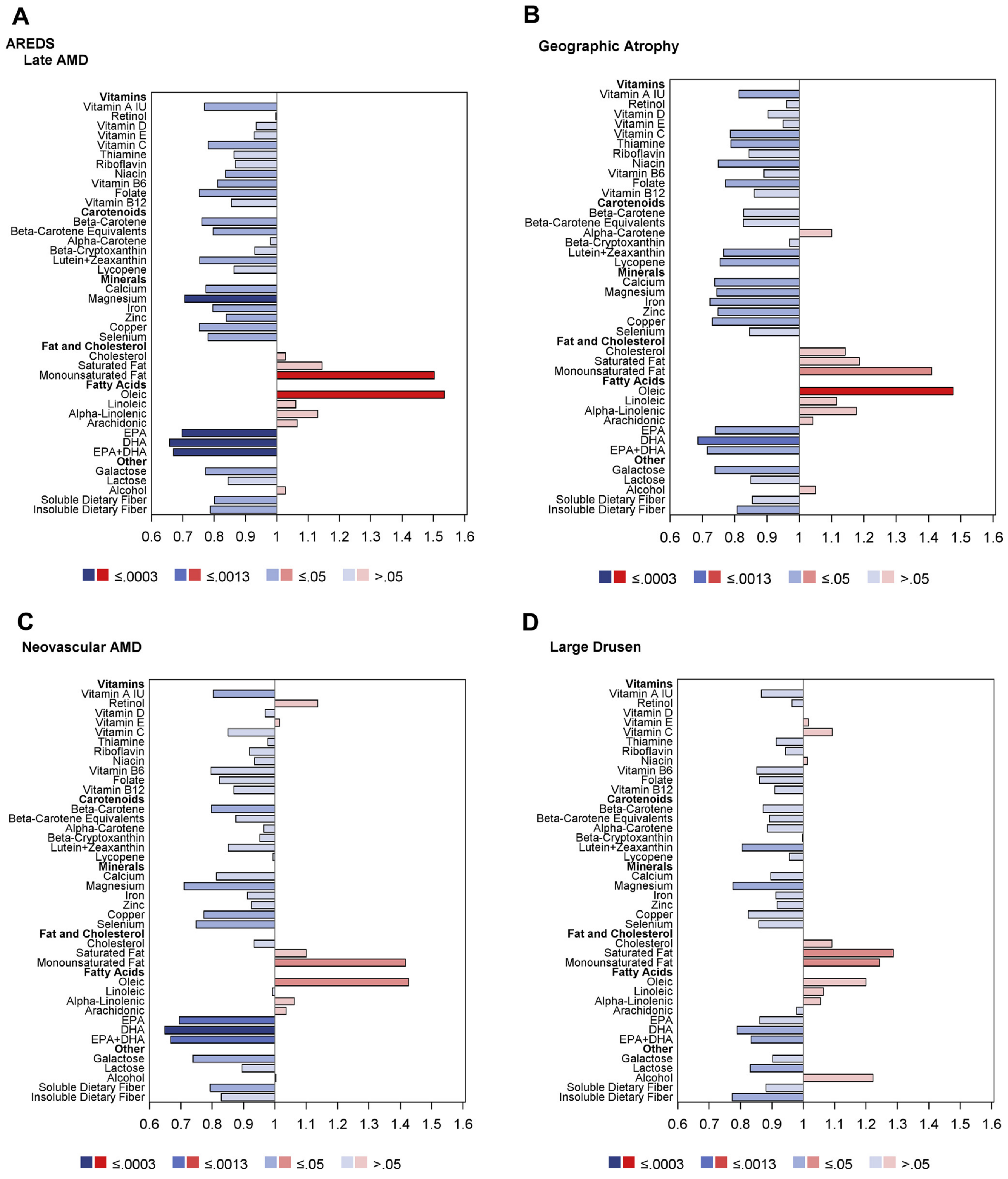

Figure 2. Butterfly plots showing proportional hazards regression modeling of progression to late age-related macular degeneration (AMD) outcomes and progression to large drusen in the Age-Related Eye Disease Study (AREDS) cohort: (**A**) late AMD, (**B**) geographic atrophy, (**C**) neovascular AMD, and (**D**) large drusen. For each nutrient, the hazard ratio of dietary intake quintile 5 (with quintile 1 as reference) is shown on the x-axis, with protective associations in blue and harmful associations in red; smaller *P* values are denoted by darker colors. DHA = docosahexaenoic acid; EPA = eicosapentaenoic acid; IU = international unit.

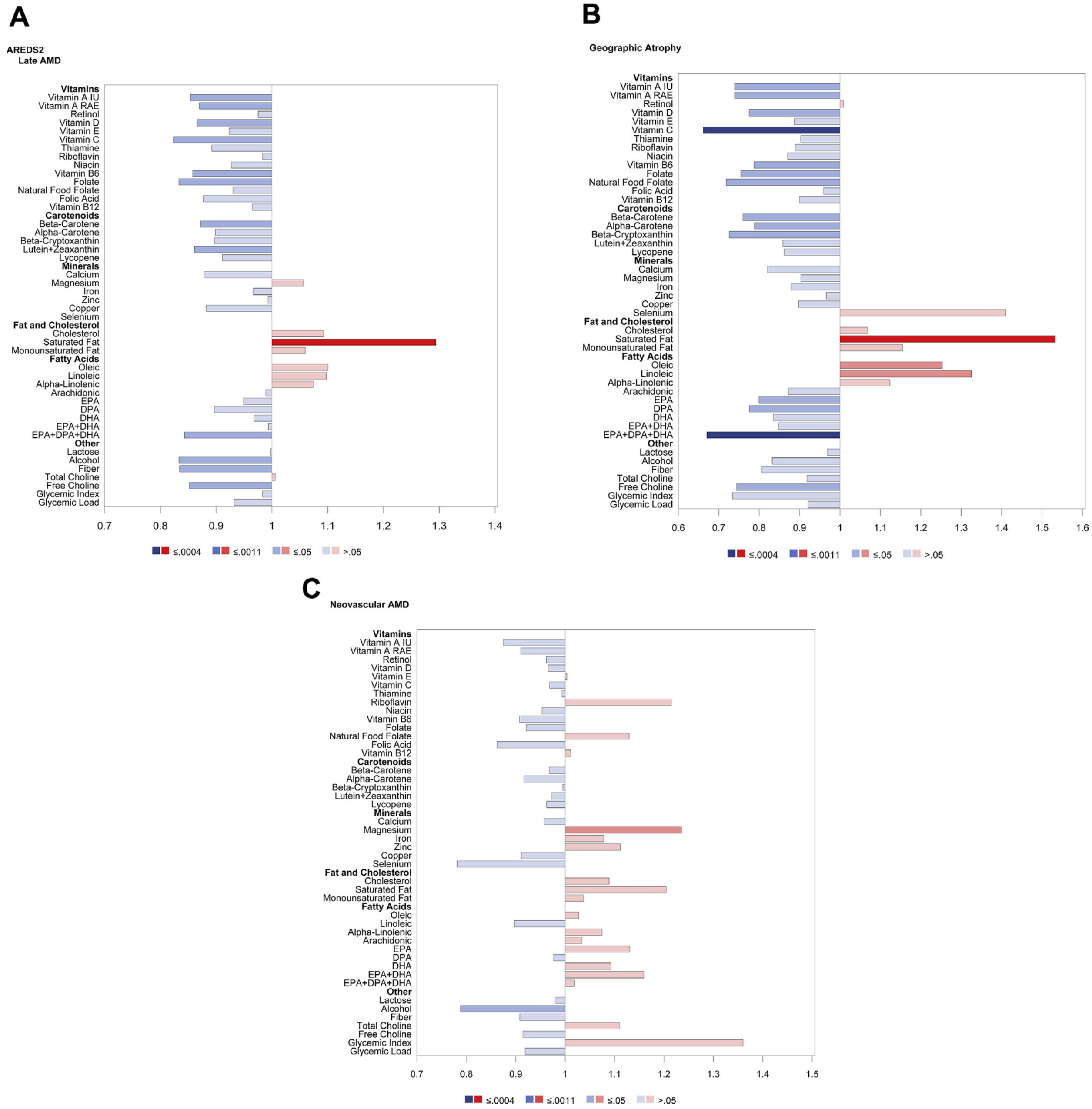

Figure 3. Butterfly plots showing proportional hazards regression modeling of progression to late age-related macular degeneration (AMD) outcomes in the Age-Related Eye Disease Study (AREDS) 2 cohort: (**A**) late AMD, (**B**) geographic atrophy, and (**C**) neovascular AMD. For each nutrient, the hazard ratio of dietary intake quintile 5 (with quintile 1 as reference) is shown on the x-axis, with protective associations in blue and harmful associations in red; smaller P values are denoted by darker colors. DHA = docosahexaenoic acid; DPA = docosapentaenoic acid; EPA = eicosapentaenoic acid; RAE = retinol activity equivalents; IU = international unit.

sensitivity analyses that included adjustment for Centrum multivitamin use in the AREDS cohort also demonstrated very similar results to the original analyses.

Proportional Hazards Regression Analyses: Progression to Large Drusen According to the Intake of Individual Dietary Nutrients

Proportional hazards regression analyses were performed for progression to large drusen using those eyes in the AREDS cohort without large drusen or late AMD at study baseline. The results are shown in Figure 2 and Table 3. The proportional hazards assumptions were met in all cases. Of the 38 nutrients examined, none had intake quintile 4, 5, or both associated significantly with either decreased or increased risk at the Bonferroni-adjusted level. For 12 nutrients, association with decreased risk was present at the nominal level: vitamin A, retinol, β-carotene, lutein and zeaxanthin, folate, copper, magnesium, DHA, omega-3 fatty acid, galactose, lactose, and insoluble dietary fiber. Conversely, for 3 nutrients, association with increased risk was present at the nominal level: saturated fatty acid, MUFA, and

Table 3. Results of the Proportional Hazards Regression Modeling of Progression to Large Drusen, According to Quintiles of Nutrient Intake in the AREDS Cohort

Nutrient	Q4 vs Q1*	Q5 vs Q1*	P†
Vitamin A (IU)	0.82 (0.68, 0.99) 0.043	0.87 (0.72, 1.04) 0.13	0.04
Retinol	1.02 (0.85, 1.23) 0.82	0.96 (0.80, 1.16) 0.70	0.19
Vitamin D	0.98 (0.81, 1.18) 0.83	1.00 (0.83, 1.21) 1.00	0.19
Vitamin E	0.97 (0.81, 1.16) 0.72	1.02 (0.84, 1.22) 0.86	0.46
Vitamin C	0.99 (0.82, 1.20) 0.92	1.09 (0.90, 1.33) 0.38	0.57
Thiamine	0.84 (0.70, 1.01) 0.064	0.91 (0.76, 1.10) 0.34	0.63
Riboflavin	0.85 (0.71, 1.02) 0.086	0.94 (0.79, 1.13) 0.53	0.45
Niacin	0.94 (0.78, 1.14) 0.53	1.01 (0.84, 1.23) 0.89	0.58
Vitamin B6	0.84 (0.70, 1.01) 0.067	0.85 (0.71, 1.03) 0.094	0.71
Folate	0.79 (0.65, 0.95) 0.0112	0.86 (0.71, 1.04) 0.1218	0.80
Vitamin B12	0.90 (0.75, 1.08) 0.27	0.91 (0.76, 1.09) 0.31	0.25
β-Carotene	0.82 (0.68, 0.99) 0.041	0.87 (0.73, 1.05) 0.15	0.11
β-Carotene equivalents	0.82 (0.68, 0.99) 0.041	0.89 (0.74, 1.07) 0.23	0.09
α-Carotene	0.97 (0.81, 1.18) 0.79	0.89 (0.73, 1.07) 0.20	0.09
β-Cryptoxanthin	1.01 (0.84, 1.22) 0.92	1.00 (0.82, 1.21) 0.98	0.97
Lutein and Zeaxanthin	0.90 (0.75, 1.09) 0.28	0.81 (0.66, 0.98) 0.028	0.61
Lycopene	0.91 (0.76, 1.10) 0.34	0.96 (0.80, 1.15) 0.62	0.96
Calcium	1.01 (0.83, 1.21) 0.95	0.90 (0.74, 1.08) 0.25	0.13
Magnesium	0.85 (0.70, 1.02) 0.088	0.78 (0.64, 0.94) 0.011	0.02
Iron	0.89 (0.74, 1.07) 0.20	0.91 (0.76, 1.10) 0.34	1.00
Zinc	0.87 (0.73, 1.04) 0.14	0.92 (0.76, 1.10) 0.36	0.59
Copper	0.81 (0.67, 0.98) 0.028	0.82 (0.68, 1.00) 0.053	0.01
Selenium	0.88 (0.73, 1.05) 0.15	0.86 (0.71, 1.03) 0.10	0.06
Cholesterol	1.23 (1.03, 1.47) 0.023	1.09 (0.91, 1.31) 0.35	0.70
Saturated fat	1.28 (1.06, 1.55) 0.011	1.29 (1.06, 1.56) 0.010	0.03
Monounsaturated fat	1.20 (1.00, 1.45) 0.049	1.24 (1.02, 1.51) 0.028	0.02
Oleic acid	1.17 (0.98, 1.41) 0.090	1.20 (0.99, 1.46) 0.064	0.03
Linoleic acid	0.90 (0.75, 1.09) 0.28	1.06 (0.89, 1.28) 0.50	0.93
α-Linolenic acid	0.93 (0.77, 1.11) 0.42	1.05 (0.88, 1.26) 0.56	0.76
Arachidonic acid	1.05 (0.88, 1.26) 0.56	0.98 (0.81, 1.18) 0.83	0.91
EPA	0.87 (0.73, 1.05) 0.14	0.86 (0.72, 1.03) 0.11	0.14
DHA	0.86 (0.72, 1.03) 0.10	0.79 (0.66, 0.95) 0.011	0.07
EPA and DHA	0.86 (0.71, 1.03) 0.099	0.83 (0.70, 1.00) 0.050	0.10
Galactose	0.83 (0.69, 1.00) 0.046	0.90 (0.75, 1.08) 0.27	0.77
Lactose	0.98 (0.82, 1.17) 0.81	0.83 (0.69, 1.00) 0.049	0.09
Alcohol	1.03 (0.83, 1.29) 0.79	1.22 (0.98, 1.52) 0.073	0.14
Soluble dietary fiber	0.97 (0.80, 1.16) 0.72	0.88 (0.73, 1.07) 0.20	0.18
Insoluble dietary fiber	0.78 (0.65, 0.95) 0.011	0.77 (0.64, 0.94) 0.009	0.04

DHA = docosahexaenoic acid; EPA = eicosapentaenoic acid; IU = international units; Q = quintile.
*Hazard ratios and 95% confidence intervals, with accompanying *P* values shown underneath. The results are shown in comparison to quintile 1 (reference), following adjustment for age, sex, smoking status, total calorie intake, body mass index, and correlation between eyes.
†*P* trend values.

cholesterol. Further analyses that included adjustment for treatment assignment demonstrated very similar results to the original analyses; additional analyses that included adjustment for Centrum multivitamin use were also very similar.

Genetic Interactions with Nutrient Intake

For the eligible nutrients, analyses of interactions between nutrient intake and genotype were performed for the 3 late AMD outcomes and for progression to large drusen. The number of nutrient–genotype combinations with *P* value for interaction of 0.01 or less was 9 in the AREDS and 2 in the AREDS2. For these 11 nutrient–genotype combinations, proportional hazards regression analyses were performed separately for each level of the genotype. The results are shown in Table 4. In the AREDS, regarding late AMD and GA, the combinations with significant results were DHA, EPA, or both with *CFH* genotype. By contrast, the relevant combinations for neovascular AMD were DHA, EPA, or both with *ARMS2* genotype. For late AMD and GA, higher DHA or EPA intake, or both, was associated with decreased risk preferentially in participants with low-risk genotypes at *CFH*. For example, for late AMD, in those with a protective allele at *CFH*, the hazard ratio associated with highest DHA intake was 0.45 (95% confidence interval, 0.30–0.67; $P < 0.0001$). By contrast, in those without protective alleles at *CFH*, no decreased risk was observed with high DHA intake. For neovascular AMD, higher DHA or EPA intake, or both, seemed to be associated with decreased risk preferentially in participants with low-risk genotypes at *ARMS2*, although the results were less consistent.

Correlations between the Dietary Intake of Different Nutrients

Pearson correlation coefficients were calculated for the dietary intake of each nutrient and every other nutrient to aid interpretation

Table 4. Results of Proportional Hazards Regression of the Progression to Late Age-Related Macular Degeneration Outcomes, According to Interactions between Nutrient Intake and Participant Genotype, Separately in the AREDS and AREDS2 Cohorts

		0		1		2	
**Nutrient**	**SNP**	*Q4 vs Q1**	*Q5 vs Q1**	*Q4 vs Q1**	*Q5 vs Q1**	*Q4 vs Q1**	*Q5 vs Q1**
AREDS							
Late AMD							
DHA	*CFH* rs10922109	1.22 (0.95, 1.58)	0.93 (0.71, 1.24)	0.81 (0.57, 1.15)	0.45 (0.30, 0.67)	0.86 (0.21, 3.60)	0.77 (0.20, 3.04)
	(0=CC; 1=CA; 2=AA)	0.12	0.63	0.24	<.0001	0.84[†]	0.71[†]
EPA	*CFH* rs10922109	1.01 (0.77, 1.31)	0.94 (0.71, 1.24)	0.68 (0.46, 1.01)	0.53 (0.35, 0.79)	0.70 (0.24, 2.00)	0.19 (0.04, 0.91)
	(0=CC; 1=CA; 2=AA)	0.96	0.67	0.056	0.002	0.50[†]	0.037[†]
EPA+DHA	*CFH* rs10922109	1.16 (0.90, 1.49)	0.90 (0.68, 1.19)	0.70 (0.48, 1.02)	0.49 (0.33, 0.73)	0.78 (0.25, 2.47)	0.49 (0.14, 1.66)
	(0=CC; 1=CA; 2=AA)	0.26	0.46	0.062	0.0005	0.67[†]	0.25[†]
Geographic atrophy							
DHA	*CFH* rs1061170	0.39 (0.20, 0.75)	0.50 (0.26, 0.93)	0.94 (0.65, 1.36)	0.65 (0.44, 0.95)	1.46 (0.97, 2.19)	0.99 (0.64, 1.53)
	(0=TT; 1=TC; 2=CC)	0.005	0.030	0.75	0.028	0.068	0.96
DHA	*CFH* rs10922109	1.34 (0.98, 1.83)	0.95 (0.68, 1.32)	0.59 (0.38, 0.91)	0.49 (0.31, 0.77)	0.90 (0.21, 3.77)	0.63 (0.14, 2.79)
	(0=CC; 1=CA; 2=AA)	0.069	0.75	0.016	0.002	0.88[†]	0.55[†]
EPA+DHA	*CFH* rs1061170	0.35 (0.18, 0.68)	0.51 (0.28, 0.93)	0.93 (0.64, 1.34)	0.70 (0.47, 1.02)	1.14 (0.76, 1.69)	0.96 (0.63, 1.47)
	(0=TT; 1=TC; 2=CC)	0.002	0.027	0.68	0.065	0.52	0.85
Neovascular AMD							
DHA	*ARMS2* rs10490924	0.96 (0.61, 1.51)	0.41 (0.23, 0.72)	1.35 (0.94, 1.94)	0.89 (0.60, 1.33)	1.07 (0.63, 1.84)	1.20 (0.68, 2.11)
	(0=GG; 1=GT; 2=TT)	0.86	0.002	0.11	0.58	0.79	0.54
EPA	*ARMS2* rs10490924	0.92 (0.58, 1.48)	0.46 (0.26, 0.81)	0.97 (0.67, 1.42)	0.90 (0.61, 1.32)	0.93 (0.54, 1.61)	1.33 (0.77, 2.30)
	(0=GG; 1=GT; 2=TT)	0.74	0.008	0.88	0.58	0.81	0.31
EPA+DHA	*ARMS2* rs10490924	0.91 (0.57, 1.47)	0.46 (0.26, 0.81)	1.11 (0.77, 1.60)	0.84 (0.57, 1.23)	1.14 (0.66, 1.96)	1.15 (0.67, 2.00)
	(0=GG; 1=GT; 2=TT)	0.71	0.007	0.56	0.36	0.64	0.61
AREDS2							
Late AMD							
Alcohol	C3 rs2230199	0.93 (0.73, 1.19)	1.08 (0.86, 1.35)	0.88 (0.67, 1.16)	0.66 (0.50, 0.88)	0.47 (0.23, 0.94)	0.51 (0.29, 0.91)
	(0=CC; 1=CG; 2=GG)	0.57	0.53	0.36	0.005	0.034	0.023
Geographic atrophy							
Copper	GRS group	0.31 (0.09, 1.07)	1.00 (0.41, 2.44)	0.59 (0.34, 1.05)	1.18 (0.73, 1.92)	0.76 (0.50, 1.14)	0.91 (0.61, 1.36)
		0.065	1.00	0.07	0.50	0.18	0.65

AMD = age-related macular degeneration; ARMS2 = age-related maculopathy susceptibility 2 gene; CFH = complement factor H gene; DHA = docosahexaenoic acid; EPA = eicosapentaenoic acid; GRS = genetic risk score; Q = quintile.

*Hazard ratios and 95% confidence intervals, with accompanying *P* values shown underneath. The results are shown in comparison to quintile 1 (reference), following adjustment for age, sex, smoking status, total calorie intake, body mass index (for AREDS participants only), and correlation between eyes. For the AREDS2 data, the results for late AMD are based on events over the full study period (i.e., including the AREDS2 10-year follow-on study), while the results for geographic atrophy and neovascular AMD are based on events up until the AREDS2 close-out (median 5 years).

[†]Very low events numbers, owing to very low likelihood of progression to late AMD in participants with two protective alleles (irrespective of nutrient intake), hence no statistical significance despite hazard ratios below one.

of the results. The results are shown in Table S4 (available at www.aaojournal.org).

Discussion

Main Findings and Interpretation

A detailed interrogation of multiple nutrients in the AREDS and AREDS2 has strengthened previous evidence for protective associations between the dietary intake of specific nutrients and risk of late AMD. In addition, it has suggested new associations with nutrients that might be studied further in observational cohorts and trials. Few previous studies have performed broad analyses of the relationships between dietary intake and risk of progression to late AMD using prospectively obtained data for a wide range of nutrients. However, multiple reports have investigated particular nutrients. This literature has been reviewed in detail.[5,8] As discussed below, the most consistent findings from previous prospective studies have been for DHA and EPA and for lutein and zeaxanthin.

In the current study, multiple dietary nutrients were associated with lower risk for progression to late AMD. This included higher intake of omega-3 long-chain polyunsaturated fatty acids (LC-PUFAs), minerals (e.g., copper, magnesium, and selenium), B vitamins, and antioxidant carotenoids (e.g., vitamin C, β-carotene, and lutein and zeaxanthin) and lower intake of saturated and monounsaturated fats. These findings were generally robust to sensitivity analyses. Most of these associations (except those for MUFA, discussed below) contribute to mounting evidence that a Mediterranean-like diet pattern,[27] or the intake of certain individual food components,[28] is associated with decreased incidence of late AMD. Indeed, the results are consistent with a recent report (using the same AREDS and AREDS2 cohorts) that showed that adherence to a Mediterranean-like diet pattern and its individual components was associated with decreased progression to late AMD.[12]

Fish, Fatty Acids, and Omega-3 Long-Chain Polyunsaturated Fatty Acids

One of the components of the Mediterranean diet pattern consistently linked to decreased late AMD is fish. A growing body of evidence suggests that these associations may be explained in part by the omega-3 LC-PUFAs found in fish: LC-PUFA intake has been related to lower risk of AMD in many, but not all, previous observational studies.[10,28–40] A meta-analysis in 2008 found that the highest dietary intake quintile of omega-3 LC-PUFAs had an odds ratio of 0.62 for late AMD.[10] In subsequent reports, a cohort study in Australia found no association, although power was low.[40] A cohort study of health professionals in the United States found no significant association between dietary DHA and EPA and visually significant late AMD[34]; however, the results of the current study (in which the associations were much stronger for GA) may be relevant, in that 96% of the events captured in the Health Professionals Study were neovascular AMD. Indeed, another cohort study of health professionals in the United States observed significant associations between DHA, EPA, and omega-3 LC-PUFA intake and decreased risk of visually significant AMD.[33]

The results of clinical trials of specific omega-3 LC-PUFAs have also been conflicting (as reviewed previously[41]). The AREDS2 comprised an RCT to evaluate the effect of adding 350 mg DHA and 650 mg EPA to the original AREDS formulation.[42] No significant risk reduction was observed. The negative results of this trial need to be contrasted with the positive results suggested by observational studies and one previous smaller trial.[43] One explanation could be differences in the dietary intake of omega-6 fatty acids (e.g., arachidonic and linoleic acids). High omega-6 fatty acid intake inhibits the anti-inflammatory effects of omega-3 LC-PUFAs, because they compete for the same enzymes in the cyclooxygenase and lipoxygenase pathways. The products of these pathways also differ: the omega-3 LC-PUFAs produce anti-inflammatory and anti-angiogenic metabolites, whereas the omega-6 fatty acids produce proinflammatory[44] and proangiogenic[45] metabolites. In addition, blood and retinal levels of omega-3 and omega-6 fatty acids, and their relationship to disease, may also depend on genetic polymorphisms (e.g., in the elongase genes of the *ELOVL* family and the desaturase genes *FADS1* and *FADS2*[46]).

It is also possible that nutrients other than DHA and EPA, but closely related to them, may be responsible. These may include DPA, VLC-PUFAs, or other unknown nutrients often consumed alongside DHA and EPA (presumably in fish) but not present in high concentrations in the AREDS2 DHA and EPA formulation. Fish is a major source of DPA. Docosapentaenoic acid can be a precursor for EPA, DHA, and other anti-inflammatory lipids.[47] Moreover, dietary fish oils, including DPA,[47] DHA, and EPA, can be precursors for VLC-PUFAs,[38] which also have anti-inflammatory properties. Very long-chain polyunsaturated fatty acids must be obtained from the diet (because they cannot be synthesized de novo in humans) and were not present in the AREDS2 DHA and EPA formulation. Interestingly, retinal membranes have a very high content of omega-3 VLC-PUFAs, and lower levels have been reported in the retinal tissue of eyes with AMD.[38]

B Vitamins

The protective associations observed between DHA and EPA intake and late AMD in the current study may also reflect, in part, the effects of other nutrients present in fish. For example, fish contains large quantities of B vitamins.[48] This idea is supported by significant correlations (Pearson ρ, 0.3–0.4; $P < 0.001$) between the intake of DHA and of several B vitamins (e.g., niacin, folate, and vitamins B6 and B12) in the combined AREDS and AREDS2 cohort. However, the protective associations between B vitamins and late AMD persisted after adjusting for DHA intake. Interestingly, a previous RCT containing 3 of these (folic acid and vitamins B6 and B12) observed a 34% reduction in incident late AMD.[49] Several prospective observational studies also suggest that some of these B vitamins may

have protective associations, but the evidence is conflicting.[32,50–53]

A mechanism by which adequate intake of folic acid and vitamins B6 and B12 may protect against AMD could be through prevention of elevated serum homocysteine. Homocysteine accumulates when B vitamin cofactors are missing for enzymes that catalyze intracellular reactions in amino acid and nucleotide metabolism. High serum homocysteine is an independent risk factor for neurodegenerative and cardiovascular diseases and has been associated with AMD.[54,55] However, the association seems to be complex and in one meta-analysis was limited to neovascular AMD.[55] This complexity may be explained by the variable status of the 3 B vitamin cofactors, other B vitamins, or common variants in methylenetetrahydrofolate reductase, a key enzyme in the conversion of homocysteine to methionine.[51,56]

A protective association between folate intake and progression to GA, but not neovascular AMD, was reported previously in a subset of the AREDS cohort.[53] The results of the current analyses were consistent between AREDS and AREDS2. Indeed, the results of the current analyses extend the evidence to include lower risk for the development of large drusen. However, in these cohorts, the associations of folate cannot be disentangled from those with other B vitamins and minerals (e.g., iron, magnesium, and zinc), given high levels of correlation between their intakes (Pearson ρ, >0.6). This may reflect the fact that fortified breakfast cereals and green vegetables usually contain high levels of both folate and these B vitamins and minerals.

Minerals

The intake of minerals present in fish may also explain, in part, the protective associations observed between omega-3 PUFA intake and late AMD. Several minerals also associated with decreased late AMD (copper, iron, magnesium, and selenium) had intake levels correlated with DHA and EPA intake (Pearson ρ, 0.3–0.4; $P < 0.001$). After adjustment for DHA or EPA intake, the associations between these minerals and decreased late AMD persisted in most cases.

Antioxidants

Fruits and vegetables contain abundant antioxidant nutrients associated with decreased late AMD in the combined cohort. This includes vitamin C, pro-vitamin A carotenoids (α-carotene, β-carotene, and β-cryptoxanthin), and lutein and zeaxanthin. A large body of evidence in other prospective studies is consistent with lower risk for AMD among those with higher intake of these nutrients.[5,9] Higher lycopene intake was also associated with decreased GA in the current study. Lycopene has not been observed in the retina, and the association may reflect the intake of other phytochemicals found in lycopene-rich foods. For example, tomatoes are rich in lycopene but are also an important source of nitrates. Indeed, previous studies have observed decreased incidence of AMD and glaucoma in individuals with higher intake of nitrates.[57,58]

Carotenoids: Lutein and Zeaxanthin

Multiple studies have observed that higher dietary intake of lutein and zeaxanthin is associated with decreased prevalence or incidence of late AMD,[11,59–63] consistent with the results from the current study. A meta-analysis in 2012 found that high lutein and zeaxanthin intake had an odds ratio of 0.74 for late AMD.[11] In a subsequent cohort study of United States health professionals, high dietary and supplement-based lutein and zeaxanthin intake was associated with decreased risk of visually significant late AMD (representing more than 96% neovascular AMD).[59] A similar pattern of results was observed for other carotenoids, including β-cryptoxanthin, α-carotene, and β-carotene. These findings are broadly consistent with the results of the current study, although their results likely would have differed if they had been able to identify incident GA cases. Finally, high-quality evidence for the effects of lutein and zeaxanthin comes from the AREDS2 RCT.[42] Adding lutein and zeaxanthin (10 mg and 2 mg, respectively) to the original AREDS formulation caused a borderline significantly decreased risk of progression to advanced AMD.

Monounsaturated Fatty Acid

Monounsaturated fatty acid intake was related to higher risk for progression to late AMD in the combined cohort and AREDS alone. Associations between MUFA intake and AMD in previous observational studies have been inconsistent. United States cohorts generally have demonstrated positive associations between MUFA intake and AMD,[64–66] whereas southern European cohorts generally have demonstrated negative associations.[40,67,68] These inconsistencies may reflect whether MUFA sources are from animal or plant foods and from high or low nutrient-dense foods. In the United States and northern European countries, the primary sources of MUFA are meat, dairy, and sugary foods, which are high in saturated fats and low in nutrient density.[69,70] Two other studies reported positive associations between MUFA intake and AMD[71,72]; however, these associations were reversed after adjustment for other dietary fats that were substantial sources of energy (i.e., PUFAs and saturated fatty acids). In the present study, MUFA intake was correlated highly with saturated fat intake (Pearson ρ, >0.7–0.8), suggesting that it may have come principally from meat and dairy food. Clinical trials indicate that replacing saturated fatty acid and carbohydrates with MUFA intake from plant and animal sources has beneficial effects on cardiovascular disease risk factors that have been related to AMD.[73]

Age-Related Macular Degeneration Stages and Subtypes

Subtype analysis of late AMD outcomes revealed overlapping but partially distinct results for GA and neovascular AMD. In general, more nutrients holding significant associations with decreased risk were observed for GA than for neovascular AMD. Of the 9 nutrients associated significantly with decreased risk of late AMD, most were

associated with decreased risk both of GA (either at the Bonferroni or the nominal level) and of neovascular AMD (at the nominal level). Conversely, of the 9 nutrients nominally associated with decreased risk of neovascular AMD, most were also associated with decreased risk of GA. Overall, these findings suggest some pathophysiologic pathways that are common to both GA and neovascular AMD and other pathways that are partially distinct. Interestingly, the results contrast sharply with those for the AREDS and AREDS2 supplements. In the current study, the associations were generally strongest for protection against GA, whereas the AREDS and AREDS2 supplements preferentially decrease the risk of neovascular AMD.[6,7] Hence, diet and oral supplementation potentially play complementary rather than competitive roles in decreasing the risk of late AMD.

The pattern of results observed in the AREDS and AREDS2 cohorts, considered separately, was relatively similar. This was despite the substantial differences between the two cohorts, including decade of study, participant age, genetic risk profile, dietary habits, and FFQ used, as well as the very high level of baseline disease severity in AREDS2. However, the levels of significance observed were generally higher in AREDS than in AREDS2. For example, of the nutrients with significant associations at the Bonferroni level in the combined cohort, many had associations at the Bonferroni level in the AREDS and at the nominal level in the AREDS2; almost all had at least nominal associations in both cohorts, in the same direction. The likely reasons for the differences and the generally higher level of significance observed in AREDS than AREDS2 may include: (1) the substantially higher proportion of participants with relatively advanced AMD at study baseline in AREDS2, (2) the higher median genetic risk score in AREDS2 participants,[21] (3) differences in food items listed on the FFQs and the underlying databases, and (4) the fact that AREDS2 participants seem to have been better nourished at baseline than those in AREDS (e.g., substantially higher intakes of DHA, EPA, and α-linolenic omega-3 fatty acids).

In general, the results did not differ substantially according to treatment assignment group in either AREDS or AREDS2, and no significant interactions were observed between nutrient intake and the treatment assignment. Hence, the protective associations between the intake of these nutrients and progression to late AMD seem largely to be independent of any of the oral supplements administered in either study, comprising antioxidants, zinc, lutein and zeaxanthin, and DHA and EPA. Similarly, the results were not affected by multivitamin intake in AREDS participants and persisted despite almost universal multivitamin intake in AREDS2 participants.

Regarding progression to large drusen, no nutrients demonstrated associations between higher intake and altered risk that were significant at the Bonferroni level. This presumably relates partly to the relatively low number of progression events, as expected for this earlier stage of a chronic age-related disease that takes decades to manifest. However, multiple nutrients had associations with decreased risk that were nominally significant. For some of these nutrients, this evidence was supported by nominal association for multiple quintiles (e.g., for folate, insoluble fiber, and lutein and zeaxanthin) or nominal associations with low hazard ratios in analyses as continuous variables (e.g., for copper and magnesium). The genuine existence of decreased risk of drusen progression through specific dietary nutrients would be an important finding because, aside from smoking cessation, no interventions are available to decrease progression to this highly prevalent disease stage. Of interest, recent analyses of the same dataset revealed an association between a Mediterranean dietary pattern and decreased risk of large drusen formation.[12]

It is interesting to examine to what extent the associations with progression to late AMD were similar or different to those with progression to large drusen. Overall, the pattern was similar. Of the 12 nutrients and molecules nominally associated with decreased risk of large drusen, all except 1 (lactose) were also associated (at least nominally) with decreased risk of late AMD. Conversely, of the 9 nutrients significantly associated (at the Bonferroni level) with decreased risk of late AMD in the combined cohort, all except 2 (alcohol and vitamin C) were also associated nominally with decreased risk of large drusen. In addition, the lipid nutrients associated with increased risk of late AMD were similar to those associated with increased risk of large drusen. Further studies will be required to examine more clearly the potential for nutrients selectively associated with decreased or increased progression risk in a stage-specific manner.

Interactions with Genotype

Importantly, the genetic analyses revealed strong interactions between omega-3 LC-PUFA intake and *CFH* genotype in AREDS. The association between higher intake and decreased late AMD and GA was found only in those with low-risk genotypes at *CFH*. Equivalent results were observed recently in analyses of the AREDS at the level of food components: similarly, a very strong interaction was observed between fish intake and *CFH* genotype.[12]

Regarding underlying mechanisms, oxidized lipids such as malondialdehyde accumulate in the retina in AMD, through oxidative stress, where they tend to provoke chronic local complement activation and inflammation.[74,75] *CFH* binds these oxidized lipids and is thought to block the proinflammatory effects. However, the AMD-associated *CFH* variant (402H) binds very poorly to malondialdehyde.[74] Hence, it is possible that CFH-directed AMD may arise through either a high-risk *CFH* genotype (causing poor *CFH* binding to oxidized lipids like malondialdehyde), low omega-3 LC-PUFA or VLC-PUFA levels in the retina (perhaps leading to stronger complement activation), or both. By contrast, the combination of a low-risk *CFH* genotype and high omega-3 LC-PUFA or VLC-PUFA levels in the retina may be strongly protective against local complement activation, which would explain the very low risk of late AMD and GA observed in these individuals.

Clinical Implications

The dietary nutrient intake levels observed to have protective associations against progression to late AMD can be

examined in Table S2, where they are displayed by quintile levels. On comparison with the Institute of Medicine recommended dietary allowances (RDAs),[76,77] different patterns emerged. For some vitamins and minerals (e.g., magnesium), individuals with dietary intakes meeting the RDAs would achieve the intake levels that had protective associations in this study (e.g., quintile 4). However, for other vitamins and minerals (e.g., vitamins B6 and C), the RDA levels fell modestly short of the intake levels with protective associations, that is, individuals with dietary intakes at the RDA levels would not achieve the intake levels that had protective associations in this study. For other nutrients (e.g., lutein and zeaxanthin), no RDA is available. Further information on the food sources of these nutrients is available from the National Institutes of Health Office of Dietary Supplements.[78] Given that this was a post hoc study of nutrient intake from dietary sources, we strongly recommend that individuals should not use the results to take nutritional supplements that have not undergone explicit testing of safety and efficacy by RCT. In addition, we strongly recommend that individuals should not consume nutritional supplements at levels higher than the Institute of Medicine tolerable upper intake levels.[76,77]

Strengths and Limitations

The combined use of 2 datasets, both large and with long follow-up, is an important strength in this study. The datasets benefit from standardized collection of information and reading center grading. Limitations include post hoc hypothesis generation, the possibility of residual or unmeasured confounding (e.g., physical activity), and differences in variables between the cohorts (e.g., BMI). In addition, diet assessment by FFQ is known to contain nondifferential measurement error, although energy adjustment may address this error partially.[79,80] Because of inherent differences in the FFQs used in AREDS and AREDS2, the assignment of food items to the nutrients analyzed had some differences between AREDS and AREDS2. Because genetic data were not available for many participants, the main analyses were not adjusted for the genetic risk score. The study may have limited generalizability to populations where diets and genotypes differ. Owing to the high degree of multiple testing, the results were presented according to Bonferroni levels of significance. However, the results with nominal significance may also be relevant, so were indicated separately. Of course, replication in prospective studies is important.

In conclusion, for multiple nutrients, higher dietary intake is associated with decreased risk of progression to late AMD. This includes nutrients in diverse classes, such as minerals, vitamins, and carotenoids. These associations apply to both subtypes of late AMD but are particularly strong for GA, for which no treatments are currently available. Because AREDS and AREDS2 supplements are protective against neovascular AMD preferentially, diet and oral supplementation may play complementary roles. For several nutrients (especially unsaturated and monounsaturated fats), higher intake is associated with increased risk of late AMD. The nutrients with protective associations against late AMD also, with a weaker level of evidence, tend to have protective associations against the development of large drusen. If genuine, this is important, because very few interventions are available to slow progression to this highly prevalent disease stage. For progression to late AMD and GA, strong genetic interactions exist for some nutrient−genotype combinations, particularly between omega-3 LC-PUFA intake and *CFH* genotype. This may provide important insights into the underlying biological pathways. Further research may shed light on the underlying mechanisms. In addition, these data may justify the organization of an RCT in which particular nutrients are tested in oral supplements.

Footnotes and Disclosures

Originally received: June 11, 2020.
Final revision: August 10, 2020.
Accepted: August 12, 2020.
Available online: August 25, 2020. Manuscript no. D-20-01642.

[1] Division of Epidemiology and Clinical Applications, National Eye Institute, National Institutes of Health, Bethesda, Maryland.

[2] Department of Ophthalmology and Visual Sciences, School of Medicine and Public Health, University of Wisconsin, Madison, Wisconsin.

[3] The Emmes Company LLC, Rockville, Maryland.

[4] Neurobiology-Neurodegeneration and Repair Laboratory, National Eye Institute, National Institutes of Health, Bethesda, Maryland.

*A complete listing of the members of the AREDS and AREDS2 Research Groups appear in the Appendix (available at www.aaojournal.org).

Disclosure(s):
All authors have completed and submitted the ICMJE disclosures form.
The author(s) have made the following disclosure(s): T.D.L.K.: Financial support − Bayer

Supported by the National Eye Institute, National Institutes of Health, Bethesda, Maryland (intramural program funds and contracts: AREDS [grant no.: NOI-EY-0-2127] and AREDS2 [grant nos.: HHS-N-260-2005-00007-C and ADB N01-EY-5-0007]). Funds were contributed to these contracts by the following National Institutes of Health institutes: Office of Dietary Supplements; National Center for Complementary and Alternative Medicine; National Institute on Aging; National Heart, Lung, and Blood Institute; and National Institute of Neurological Disorders and Stroke. The sponsor and funding organization participated in the design and conduct of the study; data collection, management, analysis, and interpretation; and preparation, review, and approval of the manuscript.

HUMAN SUBJECTS: Human subjects were included in this study. Institutional review board approval was obtained at each site and written informed consent was obtained from all participants. The research was conducted under the Declaration of Helsinki. All research complied with the Health Insurance Portability and Accountability Act (HIPAA) of 1996. All participants provided informed consent.

No animal subjects were included in this study.

Author Contributions:

Conception and design: Agrón, Mares, Clemons, Swaroop, Chew, Keenan

Analysis and interpretation: Agrón, Mares, Clemons, Swaroop, Chew, Keenan

Data collection: Agrón, Mares, Clemons, Swaroop, Chew, Keenan

Obtained funding: Agrón, Swaroop, Chew, and Keenan are NIH employees; Clemons is an Emmes employee; Mares is an employee of the University of Wisconsin; the study was performed as part of their regular employment duties.

Overall responsibility: Agrón, Mares, Clemons, Swaroop, Chew, Keenan

Abbreviations and Acronyms:
AMD = age-related macular degeneration; **AREDS** = Age-Related Eye Disease Study; **BMI** = body mass index; **DHA** = docosahexaenoic acid; **DPA** = docosapentaenoic acid; **EPA** = eicosapentaenoic acid; **FFQ** = food frequency questionnaire; **GA** = geographic atrophy; **LC-PUFA** = long-chain polyunsaturated fatty acid; **MUFA** = monounsaturated fatty acid; **RCT** = randomized controlled trial; **RDA** = recommended dietary allowance; **SD** = standard deviation; **VLC-PUFA** = very long-chain polyunsaturated fatty acid.

Keywords:
Age-related macular degeneration, Antioxidant, Carotenoid, Diet, Fatty acid, Geographic atrophy, Large drusen, Neovascular AMD, Mineral, Nutrient, Supplement, Vitamin.

Correspondence:
Tiarnan D.L. Keenan, BM BCh, PhD, National Institutes of Health, Building 10, CRC, Room 10D45, 10 Center Drive, MSC 1204, Bethesda, MD 20892-1204. E-mail: tiarnan.keenan@nih.gov; and Emily Y. Chew, MD, National Institutes of Health, Building 10, CRC, Room 3-2531, 10 Center Drive, MSC 1204, Bethesda, MD 20892-1204. E-mail: echew@nei.nih.gov.

References

1. Quartilho A, Simkiss P, Zekite A, et al. Leading causes of certifiable visual loss in England and Wales during the year ending 31 March 2013. *Eye (Lond)*. 2016;30(4):602−607.
2. Congdon N, O'Colmain B, Klaver CC, et al. Causes and prevalence of visual impairment among adults in the United States. *Arch Ophthalmol*. 2004;122(4):477−485.
3. Fritsche LG, Fariss RN, Stambolian D, et al. Age-related macular degeneration: genetics and biology coming together. *Annu Rev Genomics Hum Genet*. 2014;15:151−171.
4. Chakravarthy U, Wong TY, Fletcher A, et al. Clinical risk factors for age-related macular degeneration: a systematic review and meta-analysis. *BMC Ophthalmol*. 2010;10:31.
5. Chapman NA, Jacobs RJ, Braakhuis AJ. Role of diet and food intake in age-related macular degeneration: a systematic review. *Clin Exp Ophthalmol*. 2019;47(1):106−127.
6. Chew EY, Clemons TE, Agron E, et al. Long-term effects of vitamins C and E, beta-carotene, and zinc on age-related macular degeneration: AREDS report no. 35. *Ophthalmology*. 2013;120(8):1604−1611.
7. Age-Related Eye Disease Study Research Group. A randomized, placebo-controlled, clinical trial of high-dose supplementation with vitamins C and E, beta carotene, and zinc for age-related macular degeneration and vision loss: AREDS report no. 8. *Arch Ophthalmol*. 2001;119(10):1417−1436.
8. Gorusupudi A, Nelson K, Bernstein PS. The Age-Related Eye Disease 2 Study: micronutrients in the treatment of macular degeneration. *Adv Nutr*. 2017;8(1):40−53.
9. Zampatti S, Ricci F, Cusumano A, et al. Review of nutrient actions on age-related macular degeneration. *Nutr Res*. 2014;34(2):95−105.
10. Chong EW, Kreis AJ, Wong TY, et al. Dietary omega-3 fatty acid and fish intake in the primary prevention of age-related macular degeneration: a systematic review and meta-analysis. *Arch Ophthalmol*. 2008;126(6):826−833.
11. Ma L, Dou HL, Wu YQ, et al. Lutein and zeaxanthin intake and the risk of age-related macular degeneration: a systematic review and meta-analysis. *Br J Nutr*. 2012;107(3):350−359.
12. Keenan TD, Agron E, Mares J, et al. Adherence to the Mediterranean diet and progression to late age-related macular degeneration in the Age-Related Eye Disease Studies 1 and 2. *Ophthalmology*. 2020;127:1515−1528.
13. Age-Related Eye Disease Study Research Group. The Age-Related Eye Disease Study (AREDS): design implications. AREDS report no. 1. Control *Clin Trials*. 1999;20(6):573−600.
14. AREDS2 Research Group, Chew EY, Clemons T, et al. The Age-Related Eye Disease Study 2 (AREDS2): study design and baseline characteristics (AREDS2 report number 1). *Ophthalmology*. 2012;119(11):2282−2289.
15. Danis RP, Domalpally A, Chew EY, et al. Methods and reproducibility of grading optimized digital color fundus photographs in the Age-Related Eye Disease Study 2 (AREDS2 report number 2). *Invest Ophthalmol Vis Sci*. 2013;54(7):4548−4554.
16. SanGiovanni JP, Chew EY, Clemons TE, et al. The relationship of dietary lipid intake and age-related macular degeneration in a case-control study: AREDS report no. 20. *Arch Ophthalmol*. 2007;125(5):671−679.
17. Michaud DS, Giovannucci EL, Ascherio A, et al. Associations of plasma carotenoid concentrations and dietary intake of specific carotenoids in samples of two prospective cohort studies using a new carotenoid database. *Cancer Epidemiol Biomarkers Prev*. 1998;7(4):283−290.
18. Rimm EB, Giovannucci EL, Stampfer MJ, et al. Reproducibility and validity of an expanded self-administered semiquantitative food frequency questionnaire among male health professionals. *Am J Epidemiol*. 1992;135(10):1114−1126. discussion 1127−1136.
19. Fritsche LG, Igl W, Bailey JN, et al. A large genome-wide association study of age-related macular degeneration highlights contributions of rare and common variants. *Nat Genet*. 2016;48(2):134−143.
20. Kleinbaum DG, Klein M. The stratified Cox procedure. In: Gail M, Krickeberg K, Samet JM, et al., eds. *Survival Analysis: A Self-Learning Text*. Third ed. New York: Springer; 2012:201−227.
21. Ding Y, Liu Y, Yan Q, et al. Bivariate analysis of age-related macular degeneration progression using genetic risk scores. *Genetics*. 2017;206(1):119−133.
22. Seddon JM, Rosner B. Validated prediction models for macular degeneration progression and predictors of visual acuity loss identify high-risk individuals. *Am J Ophthalmol*. 2019;198:223−261.
23. Rhee JJ, Cho E, Willett WC. Energy adjustment of nutrient intakes is preferable to adjustment using body weight and physical activity in epidemiological analyses. *Public Health Nutr*. 2014;17(5):1054−1060.
24. Willett WC, Howe GR, Kushi LH. Adjustment for total energy intake in epidemiologic studies. *Am J Clin Nutr*. 1997;65(4 Suppl):1220S−1228S. discussion 1229S−1231S.

25. Wei LJ, Lin DY, Weissfeld L. Regression analysis of multivariate incomplete failure time data by modeling marginal distribution. *J Am Stat Assoc*. 1989;84:1065–1073.
26. Davis MD, Gangnon RE, Lee LY, et al. The Age-Related Eye Disease Study severity scale for age-related macular degeneration: AREDS report no. 17. *Arch Ophthalmol*. 2005;123(11):1484–1498.
27. Merle BMJ, Colijn JM, Cougnard-Gregoire A, et al. Mediterranean diet and incidence of advanced age-related macular degeneration: the EYE-RISK Consortium. *Ophthalmology*. 2019;126(3):381–390.
28. de Koning-Backus APM, Buitendijk GHS, Kiefte-de Jong JC, et al. Intake of vegetables, fruit, and fish is beneficial for age-related macular degeneration. *Am J Ophthalmol*. 2019;198: 70–79.
29. Merle B, Delyfer MN, Korobelnik JF, et al. Dietary omega-3 fatty acids and the risk for age-related maculopathy: the Alienor Study. *Invest Ophthalmol Vis Sci*. 2011;52(8): 6004–6011.
30. Seddon JM, George S, Rosner B. Cigarette smoking, fish consumption, omega-3 fatty acid intake, and associations with age-related macular degeneration: the US Twin Study of Age-Related Macular Degeneration. *Arch Ophthalmol*. 2006;124(7):995–1001.
31. Augood C, Chakravarthy U, Young I, et al. Oily fish consumption, dietary docosahexaenoic acid and eicosapentaenoic acid intakes, and associations with neovascular age-related macular degeneration. *Am J Clin Nutr*. 2008;88(2): 398–406.
32. SanGiovanni JP, Agron E, Meleth AD, et al. {omega}-3 Long-chain polyunsaturated fatty acid intake and 12-y incidence of neovascular age-related macular degeneration and central geographic atrophy: AREDS report 30, a prospective cohort study from the Age-Related Eye Disease Study. *Am J Clin Nutr*. 2009;90(6):1601–1607.
33. Christen WG, Schaumberg DA, Glynn RJ, Buring JE. Dietary omega-3 fatty acid and fish intake and incident age-related macular degeneration in women. *Arch Ophthalmol*. 2011;129(7):921–929.
34. Wu J, Cho E, Giovannucci EL, et al. Dietary intakes of eicosapentaenoic acid and docosahexaenoic acid and risk of age-related macular degeneration. *Ophthalmology*. 2017;124(5):634–643.
35. Tan JS, Wang JJ, Flood V, Mitchell P. Dietary fatty acids and the 10-year incidence of age-related macular degeneration: the Blue Mountains Eye Study. *Arch Ophthalmol*. 2009;127(5): 656–665.
36. Ho L, van Leeuwen R, Witteman JC, et al. Reducing the genetic risk of age-related macular degeneration with dietary antioxidants, zinc, and omega-3 fatty acids: the Rotterdam study. *Arch Ophthalmol*. 2011;129(6):758–766.
37. SanGiovanni JP, Agron E, Clemons TE, Chew EY. Omega-3 long-chain polyunsaturated fatty acid intake inversely associated with 12-year progression to advanced age-related macular degeneration. *Arch Ophthalmol*. 2009;127(1):110–112.
38. Gorusupudi A, Liu A, Hageman GS, Bernstein PS. Associations of human retinal very long-chain polyunsaturated fatty acids with dietary lipid biomarkers. *J Lipid Res*. 2016;57(3): 499–508.
39. Liu A, Chang J, Lin Y, et al. Long-chain and very long-chain polyunsaturated fatty acids in ocular aging and age-related macular degeneration. *J Lipid Res*. 2010;51(11): 3217–3229.
40. Chong EW, Robman LD, Simpson JA, et al. Fat consumption and its association with age-related macular degeneration. *Arch Ophthalmol*. 2009;127(5):674–680.
41. Souied EH, Aslam T, Garcia-Layana A, et al. Omega-3 fatty acids and age-related macular degeneration. *Ophthalmic Res*. 2015;55(2):62–69.
42. Age-Related Eye Disease Study 2 Research Group. Lutein + zeaxanthin and omega-3 fatty acids for age-related macular degeneration: the Age-Related Eye Disease Study 2 (AREDS2) randomized clinical trial. *JAMA*. 2013;309(19): 2005–2015.
43. Souied EH, Delcourt C, Querques G, et al. Oral docosahexaenoic acid in the prevention of exudative age-related macular degeneration: the Nutritional AMD Treatment 2 study. *Ophthalmology*. 2013;120(8):1619–1631.
44. Serhan CN. Resolution phase of inflammation: novel endogenous anti-inflammatory and proresolving lipid mediators and pathways. *Annu Rev Immunol*. 2007;25:101–137.
45. Sapieha P, Stahl A, Chen J, et al. 5-Lipoxygenase metabolite 4-HDHA is a mediator of the antiangiogenic effect of omega-3 polyunsaturated fatty acids. *Sci Transl Med*. 2011;3(69), 69ra12.
46. Corella D, Ordovas JM. Interactions between dietary n-3 fatty acids and genetic variants and risk of disease. *Br J Nutr*. 2012;107(Suppl 2):S271–S283.
47. Drouin G, Rioux V, Legrand P. The n-3 docosapentaenoic acid (DPA): a new player in the n-3 long chain polyunsaturated fatty acid family. *Biochimie*. 2019;159:36–48.
48. United States Department of Agriculture Agricultural Research Service. *FoodData central*; 2020. https://fdc.nal.usda.gov/fdc-app.html#/food-details/175163/nutrients. Accessed 14.05.20.
49. Christen WG, Glynn RJ, Chew EY, et al. Folic acid, pyridoxine, and cyanocobalamin combination treatment and age-related macular degeneration in women: the Women's Antioxidant and Folic Acid Cardiovascular Study. *Arch Intern Med*. 2009;169(4):335–341.
50. Gopinath B, Flood VM, Rochtchina E, et al. Homocysteine, folate, vitamin B-12, and 10-y incidence of age-related macular degeneration. *Am J Clin Nutr*. 2013;98(1):129–135.
51. Christen WG, Chasman DI, Cook NR, et al. Homocysteine, B vitamins, MTHFR genotype, and incident age-related macular degeneration. *Ophthalmol Retina*. 2018;2(5):508–510.
52. Christen WG, Cook NR, Chiuve SE, et al. Prospective study of plasma homocysteine, its dietary determinants, and risk of age-related macular degeneration in men. *Ophthalmic Epidemiol*. 2018;25(1):79–88.
53. Merle BM, Silver RE, Rosner B, Seddon JM. Dietary folate, B vitamins, genetic susceptibility and progression to advanced nonexudative age-related macular degeneration with geographic atrophy: a prospective cohort study. *Am J Clin Nutr*. 2016;103(4):1135–1144.
54. Huang P, Wang F, Sah BK, et al. Homocysteine and the risk of age-related macular degeneration: a systematic review and meta-analysis. *Sci Rep*. 2015;5:10585.
55. Pinna A, Zaccheddu F, Boscia F, et al. Homocysteine and risk of age-related macular degeneration: a systematic review and meta-analysis. *Acta Ophthalmol*. 2018;96(3): e269–e276.
56. Tanaka K, Nakayama T, Yuzawa M, et al. Analysis of candidate genes for age-related macular degeneration subtypes in the Japanese population. *Mol Vis*. 2011;17:2751–2758.
57. Gopinath B, Liew G, Kifley A, et al. Association of dietary nitrate intake with the 15-year incidence of age-related macular degeneration. *J Acad Nutr Diet*. 2018;118(12):2311–2314.

58. Kang JH, Willett WC, Rosner BA, et al. Association of dietary nitrate intake with primary open-angle glaucoma: a prospective analysis from the Nurses' Health Study and Health Professionals follow-up study. *JAMA Ophthalmol*. 2016;134(3):294−303.
59. Wu J, Cho E, Willett WC, et al. Intakes of lutein, zeaxanthin, and other carotenoids and age-related macular degeneration during 2 decades of prospective follow-up. *JAMA Ophthalmol*. 2015;133(12):1415−1424.
60. Age-Related Eye Disease Study Research Group, SanGiovanni JP, Chew EY, et al. The relationship of dietary carotenoid and vitamin A, E, and C intake with age-related macular degeneration in a case-control study: AREDS report no. 22. *Arch Ophthalmol*. 2007;125(9):1225−1232.
61. Seddon JM, Reynolds R, Rosner B. Associations of smoking, body mass index, dietary lutein, and the LIPC gene variant rs10468017 with advanced age-related macular degeneration. *Mol Vis*. 2010;16:2412−2424.
62. Seddon JM, Ajani UA, Sperduto RD, et al. Dietary carotenoids, vitamins A, C, and E, and advanced age-related macular degeneration. Eye Disease Case-Control Study Group. *JAMA*. 1994;272(18):1413−1420.
63. Chiu CJ, Milton RC, Klein R, et al. Dietary compound score and risk of age-related macular degeneration in the age-related eye disease study. *Ophthalmology*. 2009;116(5):939−946.
64. Mares-Perlman JA, Brady WE, Klein R, et al. Dietary fat and age-related maculopathy. *Arch Ophthalmol*. 1995;113(6):743−748.
65. Smith W, Mitchell P, Leeder SR. Dietary fat and fish intake and age-related maculopathy. *Arch Ophthalmol*. 2000;118(3):401−404.
66. Seddon JM, Rosner B, Sperduto RD, et al. Dietary fat and risk for advanced age-related macular degeneration. *Arch Ophthalmol*. 2001;119(8):1191−1199.
67. Roh M, Shin HJ, Lains I, et al. Higher intake of polyunsaturated fatty acid and monounsaturated fatty acid is inversely associated with AMD. *Invest Ophthalmol Vis Sci*. 2020;61(2):20.
68. Cougnard-Gregoire A, Merle BM, Korobelnik JF, et al. Olive oil consumption and age-related macular degeneration: the Alienor study. *PLoS One*. 2016;11(7):e0160240.
69. Linseisen J, Welch AA, Ocke M, et al. Dietary fat intake in the European Prospective Investigation into Cancer and Nutrition: results from the 24-h dietary recalls. *Eur J Clin Nutr*. 2009;63(Suppl 4):S61−S80.
70. O'Neil CE, Keast DR, Fulgoni VL, Nicklas TA. Food sources of energy and nutrients among adults in the US: NHANES 2003−2006. *Nutrients*. 2012;4(12):2097−2120.
71. Parekh N, Voland RP, Moeller SM, et al. Association between dietary fat intake and age-related macular degeneration in the Carotenoids in Age-Related Eye Disease Study (CAREDS): an ancillary study of the Women's Health Initiative. *Arch Ophthalmol*. 2009;127(11):1483−1493.
72. Cho E, Hung S, Willett WC, et al. Prospective study of dietary fat and the risk of age-related macular degeneration. *Am J Clin Nutr*. 2001;73(2):209−218.
73. Schwingshackl L, Hoffmann G. Monounsaturated fatty acids, olive oil and health status: a systematic review and meta-analysis of cohort studies. *Lipids Health Dis*. 2014;13(1):154.
74. Weismann D, Hartvigsen K, Lauer N, et al. Complement factor H binds malondialdehyde epitopes and protects from oxidative stress. *Nature*. 2011;478(7367):76−81.
75. Schutt F, Bergmann M, Holz FG, Kopitz J. Proteins modified by malondialdehyde, 4-hydroxynonenal, or advanced glycation end products in lipofuscin of human retinal pigment epithelium. *Invest Ophthalmol Vis Sci*. 2003;44(8):3663−3668.
76. Institute of Medicine. *Dietary Reference Intakes: The Essential Guide to Nutrient Requirements*. Washington, DC: Institute of Medicine; 2006.
77. Office of Dietary Supplements, National Institutes of Health. *Nutrient recommendations: dietary references intakes (DRI)*; 2011. https://ods.od.nih.gov/Health_Information/Dietary_Reference_Intakes.aspx. Accessed 16.07.20.
78. Office of Dietary Supplements, National Institutes of Health. *Dietary supplement fact sheets*; 2020. https://ods.od.nih.gov/factsheets/. Accessed 07/16/2020.
79. Kipnis V, Subar AF, Midthune D, et al. Structure of dietary measurement error: results of the OPEN biomarker study. *Am J Epidemiol*. 2003;158(1):14−21. discussion 22−16.
80. Subar AF, Kipnis V, Troiano RP, et al. Using intake biomarkers to evaluate the extent of dietary misreporting in a large sample of adults: the OPEN study. *Am J Epidemiol*. 2003;158(1):1−13.
