# Supplementary material for: Insights into the nutritional prevention of macular degeneration based on a comparative topic modeling approach
Source: PeerJ Comput Sci. 2024 Mar 20;10:e1940. doi: 10.7717/peerj-cs.1940 (PMC11042009; doi:10.7717/peerj-cs.1940)
Supplement: Supplemental Information 3 [file peerj-cs-10-1940-s003.zip › negative_result_reports/awh-et-al-2017-progression-from-no-amd-to-intermediate-amd-as-influenced-by-antioxidant-treatment-and-genetic-risk-an.pdf]

Check for updates

Original Manuscript

ASRS American Society of Retina Specialists

Journal of VitreoRetinal Diseases
2017, Vol. 1(1) 45-51
© The Author(s) 2017
Reprints and permission:
sagepub.com/journalsPermissions.nav
DOI: 10.1177/2474126416680931
jvrd.sagepub.com
SAGE

Progression From No AMD to Intermediate AMD as Influenced by Antioxidant Treatment and Genetic Risk: An Analysis of Data From the Age-Related Eye Disease Study Cataract Trial

Carl C. Awh, MD[1], Brent Zanke, MD, PhD[2,3], and Rafal Kustra, PhD[4]

Abstract

Purpose: To investigate the impact of antioxidant treatment and genetic risk on the development of intermediate age-related macular degeneration (AMD) in patients without baseline AMD, using data from the Age-Related Eye Disease Study (AREDS) Cataract Trial. **Methods:** Genetic risk and antioxidant treatment were analyzed as independent and interacting risk factors for the development of intermediate AMD in 554 AREDS individuals for whom genotyping was available. Genetic risk was determined using an allele dosage model based on the total number of complement factor H and age-related maculopathy sensitivity 2 risk alleles. **Results:** Overall, 14% of patients developed intermediate AMD over approximately 8 years. The risk of developing intermediate AMD varied from 6.5% for patients with 0 risk alleles to 39% for those with 3 or 4 risk alleles ($P < .0001$). Antioxidants had no impact on the development of intermediate AMD overall. However, antioxidant treatment had a significant impact on progression to intermediate AMD for patients with low or high genetic risk. Patients with 0 or 1 risk alleles had increased risk of progression to intermediate AMD (hazard ratio [HR] = 2.31, $P = .017$) if treated with antioxidants compared to placebo. Patients with 3 or 4 risk alleles had decreased risk of progression to intermediate AMD (HR = 0.27, $P = .0008$) if treated with antioxidants compared to placebo. **Conclusion:** On average, antioxidant treatment has no impact on the development of intermediate AMD in patients without AMD. However, antioxidant treatment may increase the risk of developing intermediate AMD in patients with low genetic risk and may reduce the risk of developing intermediate AMD in patients with high genetic risk. Since patients with high genetic risk have the greatest risk of progressing from intermediate to advanced AMD, genotype-directed antioxidant treatment of patients without AMD may ultimately lead to fewer cases of advanced AMD.

Keywords

age-related macular degeneration, antioxidants, AREDS, genetics, complement factor H, *ARMS2*, vitamins, environment, carotenoids, blindness, interaction

Introduction

Age-related macular degeneration (AMD) is the leading cause of severe vision loss in industrialized countries. Age-related macular degeneration progresses through well-characterized stages. Early AMD is characterized by the presence of small drusen; intermediate AMD by large drusen, pigment changes, and occasional mild vision loss; and advanced AMD by choroidal neovascularization or central geographic atrophy, typically with significant vision loss. Supplementation with zinc and antioxidants reduces the risk of progression from intermediate to advanced AMD in some patients. No identified means exist to decrease the development of early or intermediate AMD, although evidence suggests that lifestyle modifications such as smoking cessation and a healthy diet may reduce this risk.[1] Despite the lack of evidence of benefits of nutritional supplements for patients with minimal or no AMD, ocular nutritional supplements are widely marketed and used. In the United States, dietary supplements are not evaluated or regulated for efficacy or safety.[2] Supplements promoting "eye health" are readily available, and of the 59% of adults

[1] Tennessee Retina, PC, Nashville, TN, USA
[2] Faculty of Medicine, University of Toronto, Toronto, Ontario, Canada
[3] Arctic Diagnostics Inc, Toronto, Ontario, Canada
[4] Division of Biostatistics, Dalla Lana School of Public Health, University of Toronto, Toronto, Ontario, Canada

Corresponding Author:
Carl C. Awh, MD, Tennessee Retina, PC, 345 23rd Ave N Suite 350, Nashville, TN 37203, USA.
Email: carlawh@gmail.com

in the United States who take dietary supplements, 7% use a supplement for eye health.[2]

The Age-Related Eye Disease Study (AREDS) primary analysis of the influence of nutritional supplements on AMD progression included patients with categories 2 (minimal), 3 (intermediate), and 4 (advanced in the nonstudy eye) AMD. These patients were assigned to treatment with placebo, antioxidants (β-carotene, vitamin C, and vitamin E), zinc (80 mg daily), or antioxidants plus zinc. The AREDS investigators concluded that, for patients with category 3 or 4 AMD, antioxidants plus zinc reduced the 5-year risk of progression to advanced AMD by 25% and produced a 19% reduction in severe vision loss.[3] The Age-Related Eye Disease Study 2 (AREDS2) showed that omega-3 fatty acids or the alternate carotenoids, lutein and zeaxanthin, did not improve upon the original AREDS formulation.[4] However, lutein and zeaxanthin are safe and effective substitutes for β-carotene—a known promoter of lung cancer risk in smokers.[4] The AREDS or AREDS2 formulations are recommended currently for people with intermediate AMD in both eyes or with intermediate AMD in 1 eye and advanced AMD in the fellow eye.

The AREDS AMD trial was designed to study the progression of patients with established AMD. Patients in the AREDS trial with category 1 (no AMD, total drusen area <63 μm) were thought to be at low risk for vision loss from AMD and were not included in the AMD trial. These patients were enrolled in the AREDS cataract trial and were randomized to treatment with placebo or antioxidants to study the impact on cataract progression. Patients in the AREDS cataract trial were not treated with zinc or antioxidants plus zinc, because AREDS investigators had no reason to suspect that zinc would reduce the risk of progression of lens opacities.[3] Retinal outcomes were recorded, enabling a study of antioxidant treatment as primary prevention of AMD.

We previously reported the results of a genetic subgroup analysis of AREDS patients, concluding that complement factor H (*CFH*) and age-related maculopathy sensitivity 2 (*ARMS2*) genetic polymorphisms may predict response to antioxidants and/or zinc, the major components of the AREDS formulation.[5] Others have also studied the relationship between nutritional supplementation and genetic risk alleles in patients with AMD and have reached differing conclusions.[6-12] We identified an adverse response to the AREDS formulation, compared to placebo, in patients homozygous for *CFH* genetic risk and without *ARMS2* genetic risk. Based on this evidence, we concluded that genotype-directed nutritional therapy for individuals with intermediate AMD could lead to an overall reduction in the incidence of advanced AMD. A more recent analysis of over 4000 eyes of patients enrolled in the AREDS has confirmed the presence of a significant interaction between *CFH* and *ARMS2* genetic risk and treatment with antioxidants plus zinc for patients with intermediate to unilateral advanced AMD.[13]

Given evidence of the interaction between genetic risk factors and nutritional supplements in the progression of intermediate to advanced AMD, we now investigate the potential for interaction between antioxidants, genetic polymorphisms, and the de novo development of intermediate AMD among individuals enrolled in the AREDS cataract trial. The long and detailed observation and documentation of progression of these patients provide a useful data set with which the interaction of genetics, antioxidant therapy, and the development of intermediate AMD can be evaluated.

Dietary antioxidants have been shown to have a differential impact on the progression of AMD based on a conventional allele dosage model of total *CFH* and *ARMS2* risk allele number.[14] We chose to analyze the impact of antioxidant supplementation on AMD development using a similar approach, based on total *CFH* and *ARMS2* genetic risk allele number.

Methods

Patients were derived from the AREDS population. Study procedures have been reported previously.[15] Patient consent was given to permit genetic samples to be used for eye diseases only or for general research use. Patients were characterized by AREDS investigators at enrollment with time course retinal images classified by a central reading center, allowing determination of the time interval from study enrollment to AMD progression.[15] Patients with AREDS category 1 disease were enrolled only in the AREDS cataract trial and were randomized to treatment with either placebo or antioxidants (β-carotene 15 mg, vitamin C 500 mg, and vitamin E 400 IU). Retinal phenotype was documented with fundus photographs at 12-month intervals. Since genetic risk factors for AMD have been best characterized in white persons, we restricted our analysis to this group.

Genotyping

Targeted sequencing was performed on over 3000 samples from the AREDS and Universities of Michigan and Pennsylvania samples as described elsewhere.[16] The short-read sequences were matched to Genome Reference Consortium Build 37 (GRCh37) assembly before being deposited into the dbGaP database of the National Institutes of Health (NIH). We obtained the aligned sequences from dbGaP service using NIH's SRA toolkit (version 2.5.4). The read sequences for the *CFH* (chromosome 1) and *ARMS2* (chromosome 10) loci were processed using the SAMtools package (http://www.htslib.org) to deduce unphased genotypes at single-nucleotide polymorphic (SNP) variants in the *CFH* genomic region (rs1061170, rs3766405, rs1410996, rs412852) and 1 SNP (rs10490924) in the *ARMS2* region (http://www.htslib.org).

To validate the sequencing data and the deduced SNP genotypes obtained via dbGAP database, we identified 139 AREDS cases for which DNA was available from the Coriell Institute (Camden, New Jersey). Genotyping on this subset of cases was performed by Beckman Coulter Genomics (Danvers, Massachusetts) using bidirectional Sanger dideoxy sequencing. Genotypes from the same group of patients from these 2 sources were directly compared, resulting in complete concordance.

As described previously,[17] risk alleles at the *CFH* locus were determined by defining a 2 SNP haplotype that interrogates the 2 blocks of identified linkage disequilibrium.[18] To analyze the common genetic variability of the *CFH* locus, we selected a set of 5 polymorphisms for genotyping that were reported by Li et al to tag 4 common, disease-associated *CFH* haplotypes[18]: rs1048663, rs3766405, rs412852, rs11582939, and rs1066420 (previously rs1280514). rs1066420 was excluded from further analyses due to deviations from Hardy-Weinberg equilibrium in controls ($P < .001$). Linkage disequilibrium and tagging analysis of the remaining 4 SNPs revealed that any combination of 2 SNPs is sufficient to tag all common haplotypes (>1%) of this SNP haplotype block. We selected rs3766405 and rs412852 to tag the 2 major *CFH* haplotypes as has been done previously using linked SNPs rs2274700 ($r^2 = .86$ with rs3766405) and rs1061170 ($r^2 = .82$ with rs412852).[19,20] We defined the 2 SNP "high-risk" haplotype to be rs3766405 CC/rs412852 CC and the "medium-risk" haplotype to be rs3766405 CC/rs412852 CT or rs3766405 CT/rs412852 CT. All combinations not designated as "high" or "intermediate risk" were designated "low risk" at the *CFH* locus. Risk alleles at the *ARMS2* locus were defined by the single SNP rs10490924 with homozygous risk genotype (TT) combined with heterozygous genotype (TG) to define a high-risk group, and GG homozygous nonrisk genotype defining a low-risk group. This was done since TT genotype occurred in only 19 patients.

Clinical Outcome Determination

Participants in the AREDS cohort varied based on initial AMD status. Disease was classified based on the category of AMD in each eye—AREDS category 1 (no AMD): fewer than 5 small drusen with area <63 μm; category 2 (mild AMD): multiple small drusen, nonextensive intermediate drusen (63-124 μm), pigment abnormalities, or a combination thereof; category 3 (intermediate AMD): at least 1 large drusen (<125 μm), extensive intermediate drusen, or geographic atrophy not involving the center of the macula; and category 4 (advanced AMD in 1 eye only): central geographic atrophy or neovascular AMD or visual loss resulting from AMD regardless of lesion type. Only white patients with both eyes assessed as AMD category 1 at baseline and with genotyping available were eligible for our analysis (N = 554 patients). Patients with neither eye attaining category 3 by the last visit were considered censored, with the follow-up time determined by the date of the last visit. Event time for cases was determined as time to the first visit where 1 eye was assessed as progressing to category 3 AMD. Within this study set, 4 patients progressed directly to category 4 during the period of follow-up and were excluded from the analysis, resulting in 550 patients available for statistical analysis.

Statistical Analysis

We evaluated the quantitative interaction of the total number of *CFH* and *ARMS2* risk alleles (range, 0-4) with antioxidant therapy. The analysis of genetic and nutritional supplement effects, and their interaction, was done using Cox proportional hazards mode, which was also adjusted for previously reported potential confounders: body mass index, sex, age, and smoking status. All analyses were done using R statistical software (https://cran.r-project.org). The Wald test and χ^2 log-likelihood ratio tests were used for significance testing of coefficients and contrasts from the Cox model. Comparison of categorical data was done using the Pearson χ^2 test. The Mann-Whitney nonparametric test was used to compare continuous data.

Table 1. Comparison of Key Nongenetic Characteristics of Study Set Patients With All AREDS Cataract Trial Patients.

	Total	Age	BMI	High School (%)	Smoke (%)	AO (%)
Study set with DNA	554	67.8	27.4	521 (94)	283 (51)	269 (48.7)
AREDS cataract trial	1117	67.5	27.3	1050 (94)	547 (49)	534 (48.1)
P value	–	.14	.89	.93	.219	.84

Abbreviations: AO, antioxidants; AREDS, Age-Related Eye Disease Study; BMI, body mass index.

Results

The AREDS cataract trial enrolled 1117 patients with category 1 (bilateral "normal eye") designation at the study onset. Of these, 554 white individuals were represented in the targeted sequencing study set, with genotyping available at *CFH* and *ARMS2* sites. This subset with genetic information was comparable to the entire set of AREDS cataract trial patients with respect to age, body mass index, education level, smoking history, and randomization to antioxidants or placebo (Table 1).

We compared the frequency of *CFH* and *ARMS2* genotypes within our 554 patient study set with that of white persons in general. The 1000 Genomes Project (http://www.1000genomes.org) provides genotype frequencies at the *ARMS2* SNP, rs10490924, and for the 2 SNP *CFH* haplotype (rs412852 and rs3766405) for 503 individuals of European descent. The distribution of risk marker frequency was similar in both groups, indicating that our AREDS population sample was representative of the white population living in the United States with respect to *ARMS2* and *CFH* risk allele frequency (Table 2). The proportion of patients treated with antioxidants was similar across and between genetic risk groups (Table 3).

Excluding the 4 patients who progressed directly to advanced AMD, 14.2% of patients progressed to intermediate AMD at an average of 7.58 years. The AREDS cataract trial enrolled patients aged 55 to 80 years. The patients in the oldest tertile had a greater than average percentage of progression to intermediate AMD, but the difference among age groups was not statistically significant. Progression to intermediate AMD was documented at an average age of 75.9 years.

Table 2. Comparison of Distribution of *CFH* and *ARMS2* Genetic Risk in the Study Set With a Reference European Population From the 1000 Genomes Project.

Genetic Risk	AREDS (554)	EUR (503)	*P* Value
ARMS2			
Low	354 (0.639)	326 (0.648)	.813
Medium	182 (0.329)	158 (0.314)	
High	18 (0.032)	19 (0.038)	
CFH			
Low	168 (0.303)	178 (0.354)	.142
Medium	295 (0.532)	239 (0.475)	
High	90 (0.162)	86 (0.171)	

Abbreviations: AREDS, Age-Related Eye Disease Study; *ARMS2*, age-related maculopathy sensitivity 2; *CFH*, complement factor H; EUR, European descent.

Table 3. Distribution of Treatment Assignment Within Each Genetic Subgroup.

Risk Allele Number	AO Treatment	Placebo Treatment	Proportion of AO Treatment (95% CI)
0	49	60	0.45 (0.35-0.55)
1	128	114	0.53 (0.46-0.59)
2	83	92	0.47 (0.40-0.55)
3+4	10	18	0.36 (0.19-0.56)
Total	270	284	–

Abbreviation: AO, antioxidants.

Table 4. Incidence of Progression to Advanced Age-Related Macular Degeneration as a Function of Risk Allele Number.

Risk Allele Number	0	1	2	3 or 4
Subject number	108	242	172	28
Progress (%)	7 (6.48)	31 (12.8)	29 (16.9)	11 (39.3)
P (χ^2 test)	–	.12	.019	<.0001
Average time to progression	6.87	6.91	8.12	8.51
P (Mann-Whitney test)	–	.412	.643	.306
Censor time of nonprogressors	10.4	10.4	10.6	11.1

Table 5. The Impact of Antioxidant Treatment on the Risk of Progression to Advanced Age-Related Macular Degeneration, as Influenced by Total *CFH* and *ARMS2* Risk Allele Number.

Risk Allele Number	HR, AO vs PBO (95% CI)	*P* Value
0	3.98 (1.52-10.39)	.005
0 or 1	2.31 (1.64-4.59)	.017
2	0.67 (0.28-1.16)	.15
3 or 4	0.27 (0.11-0.71)	.008

Abbreviations: AO, antioxidants; *ARMS2*, age-related maculopathy sensitivity 2; *CFH*, complement factor H; HR, hazard ratio; PBO, placebo.

Progression to intermediate AMD increased with increasing total *CFH* and *ARMS2* risk alleles, ranging from 6.5% at a mean of 6.9 years for those without risk alleles to 39% at a mean of 8.5 years for those with 3 or 4 risk alleles (Table 4).

Impact of Antioxidant Supplementation on Progression to Intermediate AMD

Overall, treatment with antioxidants did not alter the progression from no AMD (AREDS category 1) to intermediate AMD (AREDS category 3; hazard ratio [HR] = 1.00; $P = .99$). Under a Cox model, the covariant of *CFH* and *ARMS2* total risk allele number (range, 0-4) was a significant determinant of progression risk (HR = 2.41; $P < .0001$) and interacted with treatment group (antioxidant vs placebo) with an HR of 0.41 ($P = .001$), demonstrating a genotype-influenced heterogeneity in antioxidant treatment response.

For patients treated with antioxidants compared to those treated with placebo, the HR for progression to intermediate AMD was increased in those with low *CFH* and *ARMS2* genetic risk and decreased in those with high *CFH* and *ARMS2* genetic risk in an allele dosage-dependent manner (Table 5). Among individuals with 0 total *CFH* and *ARMS2* risk alleles, antioxidant treatment appeared to increase the risk of progression to intermediate AMD, with an HR of 3.98 ($P = .005$). For patients with 0 or 1 total *CFH* and *ARMS2* risk alleles, an increased risk of developing intermediate AMD with antioxidant treatment was also observed (HR = 2.31; $P = .017$) (Figure 1). For patients with 2 risk alleles, antioxidant treatment had no significant impact (HR = 0.67; $P = .15$). For patients with 3 or 4 total *CFH* and *ARMS2* risk alleles, antioxidant treatment was associated with a highly significant reduction in progression to intermediate AMD (HR = 0.27; $P = .008$) (Figure 2). The opposing impact of antioxidant treatment on progression from no AMD (AREDS category 1) to intermediate AMD (AREDS category 3) as influenced by genetic risk is illustrated by comparing Figures 1 and 2.

Discussion

Our analysis of AREDS cataract trial data provides evidence that treatment with antioxidants may impact the de novo development of intermediate AMD as a function of *CFH* and *ARMS2* genetic risk. In persons without AMD, the genetic predisposition to develop AMD can be modified by antioxidant supplements. While the average progression risk for patients in this analysis was not impacted by antioxidant treatment, this average outcome appears to be the net result of dramatically different responses to antioxidant treatment; patients with low *CFH* or *ARMS2* genetic risk had significantly increased progression to intermediate AMD, while those with high *CFH* and *ARMS2* genetic risk had significantly decreased progression risk. Our analysis also demonstrated that a substantial percentage of patient with high *CFH* and *ARMS2* genetic risk developed intermediate AMD during the course of the trial (39% by 8.5 years).

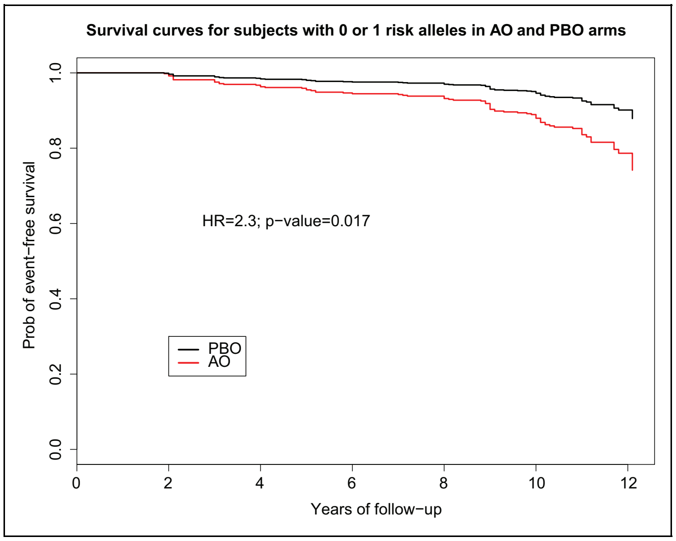

Figure 1. Estimated survival curves from Cox proportional hazards model of progression from no age-related macular degeneration (AMD) to intermediate AMD in patients with low genetic risk (0 or 1 complement factor H (*CFH*) or age-related maculopathy sensitivity 2 [*ARMS2*] risk alleles). Patients treated with antioxidants (AO) had increased progression risk (hazard ratio [HR] = 2.31, *P* = .017) compared to those treated with placebo (PBO).

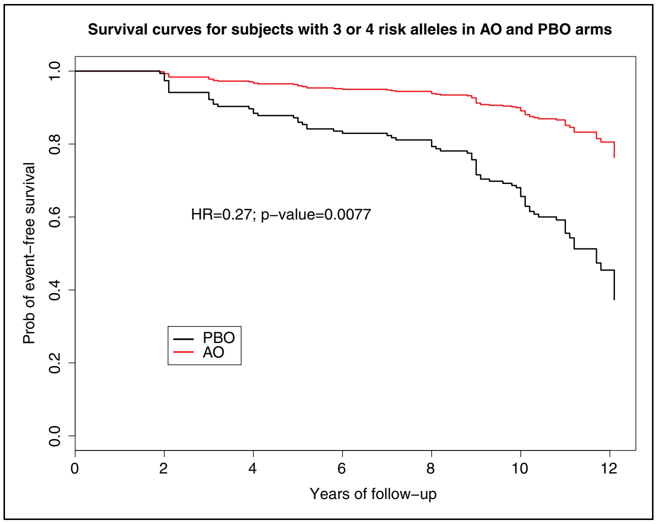

Figure 2. Estimated survival curves from Cox proportional hazards model of progression from no age-related macular degeneration (AMD) to intermediate AMD in patients with high genetic risk (3 or 4 complement factor H (*CFH*) and age-related maculopathy sensitivity 2 [*ARMS2*] risk alleles). Patients treated with antioxidants (AO) had reduced progression risk (hazard ratio [HR] = .27, *P* = .0077) compared to those treated with placebo (PBO).

Our observations are consistent with published findings from the Rotterdam Study, which reported that high dietary intake of nutrients with antioxidant properties reduced the risk of early AMD in those at high genetic risk but did not reduce progression in those with low genetic risk.[21] Patients with 2 *CFH* risk alleles in the lowest tertile of β-carotene intake had an HR for early AMD of 2.54, but those in the highest intake tertile had a reduction in HR to 1.47 (*P* = .05). Similar differences were observed for those in the highest versus lowest tertile of dietary lutein/zeaxanthin intake (HR = 2.63 vs 1.72; *P* = .05) and dietary eicosapentaenoic acid/docosahexaenoic acid (1.97 vs 1.3; *P* = .03). No benefit from any dietary antioxidant was demonstrated for patients without genetic risk, and there was a nonsignificant increase in AMD risk (HR = 1.25; *P* = .31) for patients without genetic risk in the highest tertile of β-carotene intake. Investigators from the Blue Mountain Eye Study and the Rotterdam Study also reported an interaction between genetic risk and dietary intake of lutein and zeaxanthin in a pooled data analysis (*P* = .0009), with high dietary intake attenuating the deleterious effects of genetic risk factors on the development of AMD.[14]

Other evidence exists that individuals with different genetic backgrounds respond differently to intended prophylactic treatment. Low-dose aspirin lowers the risk of developing colorectal cancer, but the protective effect is influenced by genetics.[22] A recent large case–control study found that although low-dose aspirin or nonsteroidal anti-inflammatory drug use was associated with an overall reduced risk of colorectal cancer, no protective effect was observed for 9% of patients, and an increased risk of cancer was observed in 4%, with differences attributed to genetic variations.[22]

The prevalence of dementia among individuals with AMD is increased over the population average.[23] This is consistent with the biochemical similarities between drusen and neurofibrillary plaques observed in the brains of patients with Alzheimer disease[24] and common SNPs associated with the development of each disease, such as those in the apolipoprotein E (*APOE*) gene.[16,25] Given evidence of genetically influenced variability in AMD progression associated with nutritional supplementation, it is reasonable to investigate the risk of dementia in these same groups. While the present data set does not allow such a comparison, detailed neuropsychiatric data were collected by AREDS investigators and may provide further insight into the relationship between genetics, supraphysiological dietary supplementation, and chronic neurodegenerative conditions.[23]

Treatment Implications

Since patients without AMD were randomized only to antioxidants or placebo in the AREDS cataract trial, we do not know how the zinc-containing AREDS or AREDS2 formulations impact patients without AMD. However, our prior analysis of patients with intermediate AMD revealed that patients with high *CFH* and no *ARMS2* risk alleles had increased progression to advanced AMD if treated with zinc or with antioxidants plus zinc.[5,17] Given the absence of evidence of benefit and the possibility of a similar adverse treatment response, patients without AMD should refrain from taking the AREDS or AREDS2 formulation, consistent with generally accepted recommendations.

This analysis is limited to antioxidant treatment with β-carotene, vitamin A, and vitamin E. The AREDS2 found that lutein and zeaxanthin are safe and effective substitutes for β-carotene. The AREDS2 outcomes, as well as dietary studies, suggest that lutein and zeaxanthin interact with *CFH* and *ARMS2* risk alleles similar to β-carotene.[21]

Persons with low genetic risk constitute the majority of the general population (63% in this study set). Therefore, indiscriminate treatment with antioxidants should be discouraged, as treatment may increase the risk of developing intermediate AMD in the average individual without AMD.

Five percent of patients in this analysis had 3 or 4 total *CFH* and *ARMS2* risk alleles. However, select populations (most notably, adult children of patients with advanced AMD) will contain a substantial percentage of patients with high genetic risk. In the AREDS AMD analysis, approximately 20% of patients with category 3 and 4 AMD at baseline had 3 or 4 *CFH* and *ARMS2* risk alleles.[17] Persons with high *CFH* and *ARMS2* genetic risk with intermediate AMD are at much higher than average risk of progressing to advanced AMD (approximately 75% over 10 years).[26] Therefore, decreasing the incidence of intermediate AMD in persons with high genetic risk should ultimately result in fewer cases of advanced AMD.

It is challenging to identify the effects of long-term, low-dose treatments such as nutritional supplements. The AREDS cataract trial data provide valuable insights into gene–environment interactions and their implications for antioxidant supplementation. Genotype-directed antioxidant treatment may lead to improved outcomes for persons without AMD. Our study raises interesting questions about whether the antioxidants used in the AREDS formulation may be harmful for the majority of people without AMD, who have low genetic risk, yet beneficial for those with high genetic risk. Pending validation studies, physicians, and patients should be mindful of the unproven benefit, and the potential risk, of antioxidant supplementation for most patients without AMD.

Declaration of Conflicting Interests

The author(s) declared the following potential conflicts of interest with respect to the research, authorship, and/or publication of this article: Carl C. Awh is a consultant and equity owner of ArcticDx. Brent Zanke is an employee and equity owner of ArcticDx.

Funding

The author(s) received no financial support for the research, authorship, and/or publication of this article.

References

1. Fletcher AE, Bentham GC, Agnew M, et al. Sunlight exposure, antioxidants, and age-related macular degeneration. *Arch Ophthalmol*. 2008;126(10):1396-1403.
2. Yong JJ, Scott IU, Greenberg PB. Ocular nutritional supplements: are their ingredients and manufacturers' claims evidence-based? *Ophthalmology*. 2015;122(3):595-599.
3. Age-Related Eye Disease Study Research Group. A randomized, placebo-controlled, clinical trial of high-dose supplementation with vitamins C and E, beta carotene, and zinc for age-related macular degeneration and vision loss: AREDS report no. 8. *Arch Ophthalmol*. 2001;119(10):1417-1436.
4. Chew EY, Clemons T, SanGiovanni JP, et al. The Age-Related Eye Disease Study 2 (AREDS2): study design and baseline characteristics (AREDS2 report number 1). *Ophthalmology*. 2012; 119(11):2282-2289.
5. Awh CC, Hawken S, Zanke BW. Treatment response to antioxidants and zinc based on CFH and ARMS2 genetic risk allele number in the Age-Related Eye Disease Study. *Ophthalmology*. 2015;122(1):162-169.
6. Klein ML, Francis PJ, Rosner B, et al. CFH and LOC387715/ARMS2 genotypes and treatment with antioxidants and zinc for age-related macular degeneration. *Ophthalmology*. 2008;115(6): 1019-1025.
7. Seddon JM, Reynolds R, Yu Y, Daly MJ, Rosner B. Risk models for progression to advanced age-related macular degeneration using demographic, environmental, genetic, and ocular factors. *Ophthalmology*. 2011;118(11):2203-2211.
8. Awh CC, Lane AM, Hawken S, Zanke B, Kim IK. Author reply. *Ophthalmology*. 2014;121(8):e39.
9. Chew EY, Klein ML, Clemons TE, et al; Age-Related Eye Disease Study Research Group. No clinically significant association between CFH and ARMS2 genotypes and response to nutritional supplements: AREDS report number 38. *Ophthalmology*. 2014; 121(11):2173-2180.
10. Chew EY, Klein ML, Clemons TE, Agrón E, Abecasis GR. Genetic testing in persons with age-related macular degeneration and the use of the AREDS supplements: to test or not to test? *Ophthalmology*. 2015;122(1):212-215.
11. Awh CC, Zanke B. Re: Chew et al.: genetic testing in persons with age-related macular degeneration and the use of AREDS supplements: to test or not to test? (Ophthalmology 2015;122: 212-215). *Ophthalmology*. 2015;122(10):e62-e63.
12. Awh CC, Zanke BW. Re: Chew et al.: no clinically significant association between CFH and ARMS2 genotypes and response to nutritional supplements: AREDS report number 38 (Ophthalmology 2014;121:2173-2180). *Ophthalmology*. 2015;122(8):e46.
13. Seddon JM, Silver RE, Rosner B. Response to AREDS supplements according to genetic factors: survival analysis approach using the eye as the unit of analysis [Published online July 28, 2016]. *Br J Ophthalmol*. 2016. pii: bjophthalmol-2016-308624.
14. Wang JJ, Buitendijk GH, Rochtchina E, et al. Genetic susceptibility, dietary antioxidants, and long-term incidence of age-related macular degeneration in two populations. *Ophthalmology*. 2014;121(3):667-675.
15. Age-Related Eye Disease Study Research Group. A randomized, placebo-controlled, clinical trial of high-dose supplementation with vitamins C and E and beta carotene for age-related cataract and vision loss: AREDS report no. 9. *Arch Ophthalmol*. 2001; 119(10):1439-1452.
16. Fritsche LG, Igl W, Bailey JN, et al. A large genome-wide association study of age-related macular degeneration highlights contributions of rare and common variants. *Nat Genet*. 2016;48(2): 134-143.

17. Awh CC, Lane AM, Hawken S, Zanke B, Kim IK. CFH and ARMS2 genetic polymorphisms predict response to antioxidants and zinc in patients with age-related macular degeneration. *Ophthalmology*. 2013;120(11):2317-2323.
18. Li M, Atmaca-Sonmez P, Othman M, et al. CFH haplotypes without the Y402H coding variant show strong association with susceptibility to age-related macular degeneration. *Nat Genet*. 2006; 38(9):1049-1054.
19. 1000 Genomes Project Consortium; Abecasis GR, Auton A, Brooks LD, et al. An integrated map of genetic variation from 1,092 human genomes. *Nature*. 2012;491(7422):56-65.
20. Maller J, George S, Purcell S, et al. Common variation in three genes, including a noncoding variant in CFH, strongly influences risk of age-related macular degeneration. *Nat Genet*. 2006;38(9): 1055-1059.
21. Ho L, van Leeuwen R, Witteman JC, et al. Reducing the genetic risk of age-related macular degeneration with dietary antioxidants, zinc, and omega-3 fatty acids: the Rotterdam study. *Arch Ophthalmol*. 2011;129(6):758-766.
22. Shiotani A, Murao T, Fujita Y, et al. Single nucleotide polymorphism markers for low-dose aspirin-associated peptic ulcer and ulcer bleeding. *J Gastroenterol Hepatol*. 2014;29(suppl 4): 47-52.
23. Clemons TE, Rankin MW, McBee WL; Age-Related Eye Disease Study Research Group. Cognitive impairment in the Age-Related Eye Disease Study: AREDS report no. 16. *Arch Ophthalmol*. 2006;124(4):537-543.
24. Williams MA, McKay GJ, Carson R, Craig D, Silvestri G, Passmore P. Age-related macular degeneration-associated genes in Alzheimer disease. *Am J Geriatr Psychiatry*. 2015;23(12): 1290-1296.
25. Liu CC, Kanekiyo T, Xu H, Bu G. Apolipoprotein E and Alzheimer disease: risk, mechanisms and therapy. *Nat Rev Neurol*. 2013; 9(2):106-118.
26. Klein R, Myers CE, Meuer SM, et al. Risk alleles in CFH and ARMS2 and the long-term natural history of age-related macular degeneration: the Beaver Dam Eye Study. *JAMA Ophthalmol*. 2013;131(3):383-392.
