# Supplementary material for: Insights into the nutritional prevention of macular degeneration based on a comparative topic modeling approach
Source: PeerJ Comput Sci. 2024 Mar 20;10:e1940. doi: 10.7717/peerj-cs.1940 (PMC11042009; doi:10.7717/peerj-cs.1940)
Supplement: Supplemental Information 3 [file peerj-cs-10-1940-s003.zip › negative_result_reports/nihms529465.pdf]

NIH Public Access
Author Manuscript
Ophthalmology. Author manuscript; available in PMC 2015 February 01.

Published in final edited form as:
Ophthalmology. 2014 February ; 121(2): 525–534. doi:10.1016/j.ophtha.2013.09.038.

A Multivitamin Supplement and Cataract and Age-related Macular Degeneration in a Randomized Trial of Male Physicians

Dr. William G. Christen, ScD, **Dr. Robert J. Glynn, ScD**, **Dr. JoAnn E. Manson, MD, DrPH**, **Ms. Jean MacFadyen, BA**, **Dr. Vadim Bubes, PhD**, **Dr. Miriam Schvartz, MD**, **Dr. Julie E. Buring, ScD**, **Dr. Howard D. Sesso, ScD, MPH**, and **Dr. J. Michael Gaziano, MD, MPH.**
Divisions of Preventive Medicine (Drs. Christen, Glynn, Manson, Bubes, Schvartz, Buring, Sesso, and Gaziano and Ms. MacFadyen), Aging (Drs. Buring, Sesso, and Gaziano), Pharmacoepidemiology and Pharmacoeconomics (Dr. Glynn), and Cardiovascular Disease (Dr. Gaziano) in the Department of Medicine, Brigham and Women's Hospital and Harvard Medical School; VA Boston Healthcare System (Dr. Gaziano); the Department of Population Medicine (Dr. Buring), Harvard Medical School, and the Departments of Epidemiology (Drs. Buring, Manson, and Sesso) and Biostatistics (Dr. Glynn), Harvard School of Public Health, all in Boston, MA

Abstract

Purpose—To test whether long-term multivitamin supplementation affects the incidence of cataract and/or age-related macular degeneration (AMD) in a large cohort of men.

Design—Randomized, double-blind, placebo-controlled trial.

Participants—Fourteen-thousand six hundred forty one United States male physicians aged ≥50 years.

Intervention—Daily multivitamin or placebo.

© 2013 American Academy of Ophthalmology, Inc. Published by Elsevier Inc. All rights reserved.

Corresponding author: William G. Christen, Sc.D., 900 Commonwealth Avenue East, Boston, MA 02215-1204. (617) 278-0795; Fax: (617) 278-2030; wchristen@rics.bwh.harvard.edu.

Dr. Christen had full access to all of the data in the study and takes responsibility for the integrity of the data and the accuracy of the data analyses.

Relevant Financial Disclosures:
Dr. Christen reported receiving research funding support from the National Institutes of Health and DSM Nutritional Products Inc. (formerly Roche Vitamins).
Dr. Glynn reported receiving investigator-initiated research funding from the National Institutes of Health, Bristol-Meyers Squibb, AstraZeneca, and Novartis, and signed a consulting agreement with Merck to give an invited talk.
Dr. Manson reported receiving investigator-initiated research funding from the National Institutes of Health, and assistance with study pills and packaging from BASF and Cognis Corporations for the Women's Antioxidant and Folic Acid Cardiovascular Study and from Pronova BioPharma and Pharmavite for the VITamin D and OmegA-3 TriaL, and funding from the non-profit Aurora Foundation.
Dr. Buring reported receiving investigator-initiated research funding from the National Institutes of Health, and assistance with study pills and packaging from Natural Source Vitamin E Association and Bayer Healthcare for the Women's Health Study.
Dr. Sesso reported receiving investigator-initiated research funding from the National Institutes of Health, the Tomato Products Wellness Council, and Cambridge Theranostics, Ltd.
Dr. Gaziano reported receiving investigator-initiated research funding from the National Institutes of Health the Veterans Administration, and the BASF Corporation, assistance with study agents and packaging from BASF Corporation and Pfizer (formerly Wyeth, American Home Products, and Lederle), and assistance with study packaging provided by DSM Nutritional Products, Inc. (formerly Roche Vitamins).
No other authors reported financial disclosures.

Publisher's Disclaimer: This is a PDF file of an unedited manuscript that has been accepted for publication. As a service to our customers we are providing this early version of the manuscript. The manuscript will undergo copyediting, typesetting, and review of the resulting proof before it is published in its final citable form. Please note that during the production process errors may be discovered which could affect the content, and all legal disclaimers that apply to the journal pertain.

NIH-PA Author Manuscript
NIH-PA Author Manuscript
NIH-PA Author Manuscript

NIH-PA Author Manuscript

NIH-PA Author Manuscript

NIH-PA Author Manuscript

Main Outcome Measures—Incident cataract and visually-significant AMD responsible for a reduction in best-corrected visual acuity to 20/30 or worse based on self-reports confirmed by medical record review.

Results—During an average of 11.2 years of treatment and follow-up, a total of 1,817 cases of cataract and 281 cases of visually-significant AMD were confirmed. There were 872 cataracts in the multivitamin group and 945 in the placebo group (hazard ratio [HR], 0.91; 95% confidence interval [CI], 0.83 to 0.99; p=0.04). For visually-significant AMD, there were 152 cases in the multivitamin group and 129 in the placebo group (HR, 1.19; 95% CI, 0.94 to 1.50; p=0.15).

Conclusions—These randomized trial data from a large cohort of middle-aged and older US male physicians indicate that long-term daily multivitamin use modestly and significantly decreased the risk of cataract, but had no significant effect on visually-significant AMD.

Trial registration—clinicaltrials.gov Identifier: NCT00270647

Nutritional factors are postulated to play a causal role in the development of cataract and age-related macular degeneration (AMD), two leading causes of visual impairment in older Americans.[1] Considerable observational evidence suggests that persons with higher dietary intake or blood levels of nutrients with antioxidant capabilities have lower rates of cataract and AMD.[2–7] Moreover, animal studies show that supplementation with antioxidants and other micronutrients can prevent or delay the formation of lens opacities,[3,8,9] and can reduce the damaging effects of reactive oxygen species on the retina.[10–12] However, results of nutritional supplementation trials in humans have been inconsistent.

For cataract, randomized trials conducted among older, generally well-nourished populations indicate that high-dose supplements of selected nutrients (most commonly vitamin E, vitamin C, and beta carotene), alone or in combination, for up to 10 years have little material impact on cataract occurrence.[13–21] Conversely, two trials testing a multivitamin supplement, one in a nutritionally-deficient population in China[22] and the other in a well-nourished population in Italy,[23] reported significantly lower rates of cataract in the multivitamin group. For AMD, the Age-related Eye Disease Study (AREDS) demonstrated that daily supplementation with zinc and high-dose antioxidants vitamin E, vitamin C, and beta carotene could reduce the risk of advanced AMD by 25% in persons with intermediate AMD or advanced AMD in one eye.[24] A second trial, in a population at high risk of cardiovascular disease (CVD), showed that daily folic acid, vitamin B_6, and vitamin B_{12} reduced AMD incidence by 35%–40%.[25] Five other trials testing high-dose vitamin E, vitamin C, and beta carotene, alone or in combination, reported no benefit on AMD.[26–30]

Herein, we present the final results for cataract and AMD from the multivitamin component of Physicians' Health Study II (PHS II).[31]

Methods

Study Design

The Physicians' Health Study (PHS II) was a randomized, double-blind, placebo-controlled, factorial trial evaluating a daily multivitamin (Centrum Silver), alternate day vitamin E (400 IU synthetic α-tocopherol), and daily vitamin C (500 mg synthetic ascorbic acid) in the prevention of cancer and CVD among 14,641 male physicians aged 50 years and older.[31] A fourth randomized component, alternate day beta-carotene (50 mg Lurotin), was terminated in March, 2003. The vitamin E and vitamin C components of the trial ended as scheduled on August 31, 2007.[18,30,32,33] Cataract and AMD were pre-specified secondary endpoints of PHS II.

The study design for PHS II has been described previously.[31] Briefly, recruitment, enrollment, and randomization of men into PHS II occurred in two phases (Figure 1). Phase 1 began in 1997 and included 7,641 willing and eligible participants from PHS I[20,29,34,35] who retained their original beta-carotene treatment assignment and were newly randomized to a multivitamin, vitamin C, and vitamin E. Phase 2 began in 1999 and consisted of 7,000 new physician participants who were independently randomized to each of a multivitamin, beta-carotene, vitamin C, and vitamin E, or their matching placebos. All participants provided written informed consent, and PHS II was approved by the institutional review board of the Brigham and Women's Hospital, Boston, Massachusetts.

Participants completed annual questionnaires providing information on compliance with pill taking, potential adverse events, updated risk factors, and the occurrence of any new endpoints including cataract and AMD. Treatment and follow-up continued in blinded fashion through June 1, 2011, the scheduled end of the multivitamin component. Morbidity and mortality follow-up were 98.2% and 99.9%, respectively.

Compliance with pill taking was based on self-report and defined as taking at least two thirds of the study agents. Adherence at 6 years was 73.6% for active multivitamin and 73.3% for placebo (P=0.68).

Ascertainment and Confirmation of Study Endpoints

Participants who reported cataract (n= 3,144) or AMD (n= 408) at baseline were excluded from analyses of the respective endpoint. For new reports of cataract or AMD, participants were asked to provide written consent to obtain medical records. Ophthalmologists/optometrists were contacted by mail and asked to provide information about the reported endpoint by completing a questionnaire or, alternatively, supplying copies of relevant medical records.

Cataract

The cataract questionnaire asked about the presence of lens opacities, diagnosis date, best-corrected visual acuity, cataract extraction, other ocular abnormalities, and cataract type and etiology.

The co-primary vision endpoint was incident cataract, defined as a confirmed lens opacity, diagnosed after randomization, but before June 1, 2011, age-related in etiology, and responsible for reduced best-corrected visual acuity to 20/30 or worse. Cataract extraction was a pre-specified secondary endpoint and was defined as the surgical removal of an incident cataract. A total of 11,497 participants did not report cataract at baseline and are included in this analysis. Of these, 5,736 men were in the multivitamin group and 5,761 in the placebo group (Figure 1).

Age-related Macular Degeneration

The AMD questionnaire requested information about diagnosis date, best-corrected visual acuity at diagnosis, and date when best-corrected visual acuity reached 20/30 or worse. Information was also requested about signs of AMD observed (drusen, retinal pigment epithelium [RPE] hypo/hyperpigmentation, geographic atrophy, RPE detachment, subretinal neovascular membrane, or disciform scar) when visual acuity was first noted to be 20/30 or worse, and the date exudative neovascular disease (defined by presence of RPE detachment, subretinal neovascular membrane, or disciform scar) was first noted. The questionnaire also asked whether there were other ocular abnormalities and, if so, whether the AMD, by itself, was significant enough to reduce best-corrected visual acuity to 20/30 or worse.

NIH-PA Author Manuscript NIH-PA Author Manuscript NIH-PA Author Manuscript

The co-primary vision endpoint was visually-significant AMD defined as confirmed AMD diagnosed after randomization, but before June 1, 2011, and best-corrected visual acuity loss to 20/30 or worse attributable to AMD. Two secondary endpoints were total AMD, comprised of all incident cases with or without vision loss, and advanced AMD, comprised of cases of exudative neovascular AMD plus geographic atrophy.

A total of 14,233 participants did not report AMD at baseline and are included in this analysis. Of these, 7,111 men were in the multivitamin group and 7,122 in the placebo group (Figure 1).

Statistical Analysis

In separate analyses of cataract and AMD, participants were classified according to randomized multivitamin treatment assignment and followed until the occurrence of that endpoint, death, or the end of the multivitamin component of PHS II, whichever came first. Estimated power for the primary study endpoints of incident cataract and visually-significant AMD was based on event rates observed in PHS I, and was estimated to be greater than 80% to detect a 10% reduction in cataract, and a 20% reduction in visually-significant AMD.

The distributions of baseline characteristics in the multivitamin and placebo groups were compared using 2-sample t-tests, chi-square tests for proportions, and tests for trend for ordinal categories. Kaplan-Meier curves estimated cumulative incidence over time by randomized group, and were compared using a crude log-rank test. Cox proportional-hazards models were used to estimate the hazard ratio (HR) of cataract and AMD among those in the multivitamin group compared to placebo after adjustment for age (years) at baseline, PHS cohort (original PHS I participant, new PHS II participant), and randomized beta carotene, vitamin E and vitamin C assignments. Models were also fit separately within three baseline age groups: 50–59, 60–69, ≥70 years. Tests of trend for the effect of age on the association between multivitamins and cataract or AMD were calculated by including a term for the interaction of multivitamins and age (with values 1 to 3 corresponding to the three age groups) in a proportional hazards model. The proportionality assumption was not violated for any cataract (diagnosis, p=0.99; extraction, p=0.75) or AMD endpoint (visually-significant AMD, p=0.40; total AMD, p=0.86; advanced AMD, p=0.98). Ninety-five percent confidence intervals (CI) and two-sided P value were calculated.

We analyzed subgroup data by categories of baseline variables, and by the other randomized treatment assignments. We explored possible effect modification by using interaction terms between subgroup indicators and multivitamin assignment.

We also considered the possibility that the apparent effect of multivitamins on one endpoint (e.g., cataract) reflected, at least in part, the effect of the intervention on the second endpoint (e.g., AMD). To address this, two separate proportional hazards models were fitted to estimate the effect of the intervention on one endpoint while adjusting for a diagnosis of the other as a time-varying covariate. Models were also fitted to estimate the effect of the intervention on one endpoint prior to, and after, diagnosis of the second endpoint.

Individuals were considered the unit of analysis because eyes were not examined independently, and were classified according to the status of the worse eye as defined by disease severity.[36,37]

Results

As expected in this large randomized trial, baseline characteristics had comparable distributions between the multivitamin and placebo groups (Table 1).

NIH-PA Author Manuscript NIH-PA Author Manuscript NIH-PA Author Manuscript

NIH-PA Author Manuscript

NIH-PA Author Manuscript

NIH-PA Author Manuscript

During a mean follow-up of 11.2 years (median [interquartile range], 11.2 [10.7–13.3] years; maximum, 13.8 years), 1,817 cataracts and 1,337 cataract extractions were confirmed. We also confirmed 538 cases of AMD, including 281 cases of visually-significant AMD and 144 cases of advanced AMD.

Cataract

Overall, there was a significant 9% lower risk of cataract in the multivitamin group compared to placebo (872 versus 945 cases; HR, 0.91; 95% CI, 0.83–0.99; p=0.04) (Table 2). For subtypes, there was a significant 13% reduced risk of nuclear sclerosis (NS) in the multivitamin group (800 versus 900 cases; HR, 0.87; 95% CI, 0.79–0.96; p=0.005). There was a non-significant reduction in cortical cataract (356 versus 387 cases; HR, 0.90; 95% CI, 0.78–1.04; p=0.17). There were similar numbers of posterior subcapsular (PSC) cataracts in the multivitamin and placebo groups (247 versus 248 cases; HR, 0.98; 95% CI, 0.82–1.17; p=0.85). The findings were similar for extraction of cataract and subtypes.

The benefits of daily multivitamin use were greater in older men, although no test of trend attained statistical significance for either cataract diagnosis or extraction (Table 2). For both endpoints, a beneficial effect of multivitamins began to emerge midway through follow-up and persisted throughout the remainder of the trial (crude log-rank P=0.05 for both endpoints) (Figure 2).

The effect of multivitamins on cataract did not differ markedly within categories of baseline characteristics (Table 3). However, the effect did appear to vary according to vitamin C treatment assignment (p interaction=0.04) with a significant benefit observed only among men in the vitamin C placebo group.

Age-related Macular Degeneration

Men in the multivitamin group had a 19% increased risk of visually-significant AMD that was not statistically significant (152 versus 129 cases; HR, 1.19; 95% CI, 0.94 to 1.50; p=0.15) (Table 4). There was also a significant 22% increased risk of total AMD (with or without vision loss) (294 versus 244 cases; HR, 1.22; 95% CI, 1.03 to 1.44; p=0.02), and a non-significant 22% increased risk of advanced AMD (79 versus 65 cases; HR, 1.22; 95% CI, 0.88 to 1.70; p=0.23) in the multivitamin group.

Hazard ratios for all AMD endpoints tended to be highest in the oldest age group, although no test of trend attained statistical significance. For the primary endpoint of visually-significant AMD, the curves appeared to diverge midway through follow-up but never attained statistical significance (crude log-rank P=0.18) (Figure 3). Curves for total AMD began to diverge earlier in the trial, and persisted throughout the trial (log-rank P=0.03). For advanced AMD, there was no apparent effect of a daily multivitamin at any point during the trial (crude log-rank P=0.25).

The effect of multivitamins on visually-significant AMD did not differ appreciably within categories of baseline characteristics (Table 3).

For both cataract and AMD, HR estimates for multivitamin treatment were not materially altered in analyses that accounted for diagnosis of the second endpoint. For example, the HR for cataract changed little (0.90; 95% CI, 0.82–0.99; P=0.03) after adjustment for a diagnosis of visually-significant AMD as a time-varying covariate. Similarly, the HR for visually-significant AMD changed little (1.18; 95% CI, 0.94–1.50; P=0.16) after adjustment for diagnosed cataract. We also compared HRs prior to and after diagnosis of the second endpoint. The HR for cataract was 0.92 (95% CI, 0.84–1.01) with no prior diagnosis of visually-significant AMD, and 0.69 (95% CI, 0.41–1.17) after a diagnosis of visually-

significant AMD. For visually-significant AMD, the HR was 1.31 (95% CI, 0.92–1.87) with no prior diagnosis of cataract, and 1.08 (95% CI, 0.79–1.48) after a diagnosis of cataract.

Discussion

In this large-scale randomized trial of middle-aged and older men, long-term daily multivitamin use modestly and significantly reduced the co-primary vision endpoint of cataract after more than ten years of treatment and follow-up. There was no significant benefit or risk of daily multivitamin use on visually-significant AMD, the second co-primary vision endpoint, although the HRs tended to be modestly elevated.

Our findings for cataract are consistent with results of two prior trials of multivitamin use in cataract prevention. In the Linxian Cataract Study, conducted in a nutritionally-deficient population in China, persons aged 65 to 74 years randomized to a daily supplement of 14 vitamins and 12 minerals at 2 to 3 times the United States (U.S) recommended dietary allowance (RDA), compared to placebo, had a significant 36% lower prevalence of nuclear cataract after 6 years of treatment.[22] There was no difference in the prevalence of cortical or PSC cataract in those aged 65 to 74 years, nor was there any difference in the prevalence of any cataract type in those aged 54–64 years. In the Italian American Clinical Trial of Nutritional Supplements and Age-related Cataract (CTNS), persons aged 55–75 years randomized to a daily multivitamin (Centrum), compared to placebo, had a significant 18% reduction in cataract development or progression after 9-years of treatment and follow-up.[23] Analyses of subtypes indicated a significant 34% reduction in nuclear (HR, 0.66; 95% CI, 0.50–0.88), a non-significant 22% reduction in cortical (HR, 0.78; 95% CI, 0.60–1.02), and a significant two-fold increased risk of PSC cataract (HR, 2.00; 95% CI, 1.35–2.98) in the multivitamin group. Neither the Linxian nor the CTNS study examined the effect of the intervention on AMD.

Taken together, our findings in PHS II and two prior trials indicate that long-term daily multivitamin use may have a small to moderate beneficial effect on risk of cataract, and particularly nuclear cataract. Given that an estimated 10 million adults in the U.S. have impaired vision due to cataract,[38,39] even a modest reduction in risk of cataract would have a large public health impact. It should also be noted that while the main trial results in AREDS indicated no benefit on lens opacity progression for daily treatment with high dose vitamin E (400 IU), vitamin C (500 mg), and beta carotene (15 mg), a propensity score analysis showed that self-selection for Centrum use (provided by AREDS) by approximately two-thirds of AREDS participants was associated with a significant reduction in lens opacity progression, particularly for nuclear opacities.[40]

PHS II is the first large-scale randomized trial to test a multivitamin supplement in AMD prevention. Our finding of no significant benefit appears to contrast with the benefits for other vitamin/mineral combinations in prior trials of AMD. For example, AREDS demonstrated that daily zinc (80 mg) and a high-dose antioxidant combination of vitamin E (400 IU), vitamin C (500 mg), and beta carotene (15 mg) significantly slowed the progression of AMD.[24] The multivitamin in PHS II included these nutrients at RDA levels (zinc [15 mg], vitamin E [45 IU], vitamin C [60 mg], beta-carotene [5000 IU vitamin A, 20% as beta carotene]), rather than the high-dose formulation tested in AREDS. In addition, AREDS tested a higher-risk population than PHS II (no reported diagnosis of AMD at baseline), and their primary endpoint was advanced AMD whereas in PHS II, the primary endpoint was visually-significant AMD, most commonly characterized by some combination of drusen and RPE changes. Thus, our endpoint represented an earlier stage of disease development than the advanced AMD endpoint in AREDS. It is worth noting that

NIH-PA Author Manuscript
NIH-PA Author Manuscript
NIH-PA Author Manuscript

NIH-PA Author Manuscript

NIH-PA Author Manuscript

NIH-PA Author Manuscript

AREDS reported no benefit of the zinc and antioxidant combination in participants with early stage AMD at baseline.[24]

Our findings also appear to contrast with the findings in the Women's Antioxidant and Folic Acid Cardiovascular Study (WAFACS), where combined treatment with folic acid, vitamin B_6, and vitamin B_{12} for 7.3 years reduced the risk of a diagnosis of AMD by 35% to 40%.[25] Both WAFACS and PHS II employed the same method of case ascertainment and the same diagnostic criteria for AMD, but differed in other important respects. WAFACS tested pharmacologic doses of folic acid (2.5 mg/d), vitamin B_6 (50 mg/d), and vitamin B_{12} (1 mg/d), whereas the multivitamin in PHS II contained RDA levels of these nutrients (folic acid [400 μg], vitamin B_6 [3 mg], vitamin B_{12} [25 μg]). In addition, WAFACS was comprised of women with preexisting CVD or 3 or more CVD risk factors, whereas PHS II was comprised primarily of apparently healthy men. It therefore seems possible that our finding of no benefit (and possible harm) may reflect, at least in part, the lower dosage of the PHS II multivitamin plus the generally lower risk profile of the PHS II population.

Several possible limitations of our study need to be considered. At the initiation of PHS II in 1997, we selected a commonly used multivitamin, Centrum Silver, to increase the potential generalizability of study findings. The same formulation was used throughout PHS II. However, after the inception of PHS II, the doses of several nutrients in Centrum Silver were changed and several nutrients were added including lutein (250 μg). Lutein has been shown to be of possible benefit against cataract and AMD in observational studies,[41–49] and in several small randomized trials,[50–55] although the recently completed Age-Related Eye Disease Study 2 reported no overall benefit on either endpoint.[56,57] However, the effect of adding lutein to the Centrum Silver formulation could not be addressed in our study. Compliance with study medication is a concern in any long-term randomized trial; however, compliance with the daily multivitamin remained high throughout follow-up. Finally, the PHS II population is comprised of generally well-nourished male physicians and our findings may not apply to women or to less well-nourished populations.

Several aspects of our methodology also deserve consideration. Case identification was based on participant reports, and thus some underascertainment of cataract and AMD is plausible. Such underascertainment would likely reduce study power, but is not associated with bias in randomized comparisons. Random misclassification was reduced by the use of medical records to confirm the participant reports. Non-random misclassification was unlikely since medical records were reviewed by an investigator (WGC) masked to treatment assignment, and study participants and treating ophthalmologists and optometrists were similarly unaware of treatment assignment. Finally, the equal distribution of baseline characteristics between the multivitamin and placebo groups indicates that confounding by measured factors is unlikely, and that other potential confounders, which were either unmeasured or unknown, were also likely to be evenly distributed between the two treatment groups.

In summary, the finding in this large-scale randomized trial of middle-aged and older men that long-term daily multivitamin use is associated with a modest but significant reduction in cataract, and in particular nuclear cataract, is consistent with results of previous trials of multivitamin use in cataract prevention. The finding of no significant benefit or harm for multivitamin use in prevention of visually-significant AMD needs to be confirmed in other populations of men and women.

Acknowledgments

Supported by grants CA 097193 (which included funding from the National Eye Institute and the National Institute on Aging), CA 34944, CA 40360, HL 26490, and HL 34595 from the National Institutes of Health (Bethesda, MD),

NIH-PA Author Manuscript

NIH-PA Author Manuscript

NIH-PA Author Manuscript

and an investigator-initiated grant from BASF Corporation (Florham Park, NJ). Study agents and packaging were provided by BASF Corporation and Pfizer (formerly Wyeth, American Home Products, and Lederle) (New York, NY), and study packaging was provided by DSM Nutritional Products, Inc. (formerly Roche Vitamins) (Parsippany, NJ).

Role of the Sponsors: NIH, BASF, Pfizer, and DSM Nutritional Products Inc had no role in the study design; conduct of the study; collection, management, analysis, and interpretation of the data; or preparation, review, or approval of the manuscript.

References

1. Congdon NG, West KP Jr. Nutrition and the eye. Curr Opin Ophthalmol. 1999; 10:464–73. [PubMed: 10662253]
2. Chiu CJ, Taylor A. Nutritional antioxidants and age-related cataract and maculopathy. Exp Eye Res. 2007; 84:229–45. [PubMed: 16879819]
3. Agte V, Tarwadi K. The importance of nutrition in the prevention of ocular disease with special reference to cataract. Ophthalmic Res. 2010; 44:166–72. AQ: duplicated with 10. [PubMed: 20829640]
4. Fletcher AE. Free radicals, antioxidants and eye diseases: evidence from epidemiological studies on cataract and age-related macular degeneration. Ophthalmic Res. 2010; 44:191–8. [PubMed: 20829643]
5. Krishnadev N, Meleth AD, Chew EY. Nutritional supplements for age-related macular degeneration. Curr Opin Ophthalmol. 2010; 21:184–9. [PubMed: 20216418]
6. Seddon JM. Multivitamin-multimineral supplements and eye disease: age-related macular degeneration and cataract. Am J Clin Nutr. 2007; 85:304S–7S. [PubMed: 17209215]
7. Cui YH, Jing CX, Pan HW. Association of blood antioxidants and vitamins with risk of age-related cataract: a meta-analysis of observational studies. Am J Clin Nutr. 2013; 98:778–86. [PubMed: 23842458]
8. Gerster H. Antioxidant vitamins in cataract prevention. Z Ernahrungswiss. 1989; 28:56–75. [PubMed: 2655316]
9. Taylor A. Role of nutrients in delaying cataracts. Ann N Y Acad Sci. 1992; 669:111–23. discussion 123–4. [PubMed: 1444018]
10. Organisciak DT, Wang HM, Li ZY, Tso MO. The protective effect of ascorbate in retinal light damage of rats. Invest Ophthalmol Vis Sci. 1985; 26:1580–8. [PubMed: 4055290]
11. Ham WT Jr, Mueller HA, Ruffolo JJ Jr, et al. Basic mechanisms underlying the production of photochemical lesions in the mammalian retina. Curr Eye Res. 1984; 3:165–74. [PubMed: 6690219]
12. Tso MO. Experiments on visual cells by nature and man: in search of treatment for photoreceptor degeneration. Friedenwald lecture. Invest Ophthalmol Vis Sci. 1989; 30:2430–54. [PubMed: 2687190]
13. Teikari JM, Rautalahti M, Haukka J, et al. Incidence of cataract operations in Finnish male smokers unaffected by alpha tocopherol or beta carotene supplements. J Epidemiol Community Health. 1998; 52:468–72. [PubMed: 9799882]
14. Age-Related Eye Disease Study Research Group. A randomized, placebo-controlled, clinical trial of high-dose supplementation with vitamins C and E and beta carotene for age-related cataract and vision loss: AREDS report no. 9. Arch Ophthalmol. 2001; 119:1439–52. [PubMed: 11594943]
15. Chylack LT Jr, Brown NP, Bron A, et al. REACT Group. The Roche European American Cataract Trial (REACT): a randomized clinical trial to investigate the efficacy of an oral antioxidant micronutrient mixture to slow progression of age-related cataract. Ophthalmic Epidemiol. 2002; 9:49–80. [PubMed: 11815895]
16. McNeil JJ, Robman L, Tikellis G, et al. Vitamin E supplementation and cataract: randomized controlled trial. Ophthalmology. 2004; 111:75–84. [PubMed: 14711717]
17. Christen WG, Glynn RJ, Chew EY, Buring JE. Vitamin E and age-related cataract in a randomized trial of women. Ophthalmology. 2008; 115:822–9. [PubMed: 18067963]

NIH-PA Author Manuscript
NIH-PA Author Manuscript
NIH-PA Author Manuscript

18. Christen WG, Glynn RJ, Sesso HD, et al. Age-related cataract in a randomized trial of vitamins E and C in men. Arch Ophthalmol. 2010; 128:1397–405. [PubMed: 21060040]
19. Christen W, Glynn R, Sperduto R, et al. Age-related cataract in a randomized trial of beta-carotene in women. Ophthalmic Epidemiol. 2004; 11:401–12. [PubMed: 15590586]
20. Christen WG, Manson JE, Glynn RJ, et al. A randomized trial of beta carotene and age-related cataract in US physicians. Arch Ophthalmol. 2003; 121:372–8. [PubMed: 12617708]
21. Gritz DC, Srinivasan M, Smith SD, et al. The Antioxidants in Prevention of Cataracts Study: effects of antioxidant supplements on cataract progression in South India. Br J Ophthalmol. 2006; 90:847–51. [PubMed: 16556618]
22. Sperduto RD, Hu TS, Milton RC, et al. The Linxian cataract studies. Two nutrition intervention trials. Arch Ophthalmol. 1993; 111:1246–53. [PubMed: 8363468]
23. Clinical Trial of Nutritional Supplements and Age-Related Cataract Study Group. A randomized, double-masked, placebo-controlled clinical trial of multivitamin supplementation for age-related lens opacities: Clinical Trial of Nutritional Supplements and Age-Related Cataract report no. 3. Ophthalmology. 2008; 115:599–607. [PubMed: 18387406]
24. Age-Related Eye Disease Study Research Group. A randomized, placebo-controlled, clinical trial of high-dose supplementation with vitamins C and E, beta carotene, and zinc for age-related macular degeneration and vision loss: AREDS report no. 8. Arch Ophthalmol. 2001; 119:1417–36. [PubMed: 11594942]
25. Christen WG, Glynn RJ, Chew EY, et al. Folic acid, pyridoxine, and cyanocobalamin combination treatment and age-related macular degeneration in women: the Women's Antioxidant and Folic Acid Cardiovascular Study. Arch Intern Med. 2009; 169:335–41. [PubMed: 19237716]
26. Teikari JM, Laatikainen L, Virtamo J, et al. Six-year supplementation with alpha-tocopherol and beta-carotene and age-related maculopathy. Acta Ophthalmol Scand. 1998; 76:224–9. [PubMed: 9591958]
27. Taylor HR, Tikellis G, Robman LD, et al. Vitamin E supplementation and macular degeneration: randomised controlled trial. BMJ. 2002; 325:11. [PubMed: 12098721]
28. Christen WG, Glynn RJ, Chew EY, Buring JE. Vitamin E and age-related macular degeneration in a randomized trial of women. Ophthalmology. 2010; 117:1163–8. [PubMed: 20153900]
29. Christen WG, Manson JE, Glynn RJ, et al. Beta carotene supplementation and age-related maculopathy in a randomized trial of US physicians. Arch Ophthalmol. 2007; 125:333–9. [PubMed: 17353403]
30. Christen WG, Glynn RJ, Sesso HD, et al. Vitamins E and C and medical record-confirmed age-related macular degeneration in a randomized trial of male physicians. Ophthalmology. 2012; 119:1642–9. [PubMed: 22503302]
31. Christen WG, Gaziano JM, Hennekens CH. Design of Physicians' Health Study II--a randomized trial of beta-carotene, vitamins E and C, and multivitamins, in prevention of cancer, cardiovascular disease, and eye disease, and review of results of completed trials. Ann Epidemiol. 2000; 10:125–34. [PubMed: 10691066]
32. Gaziano JM, Glynn RJ, Christen WG, et al. Vitamins E and C in the prevention of prostate and total cancer in men: the Physicians' Health Study II randomized controlled trial. JAMA. 2009; 301:52–62. [PubMed: 19066368]
33. Sesso HD, Buring JE, Christen WG, et al. Vitamins E and C in the prevention of cardiovascular disease in men: the Physicians' Health Study II randomized controlled trial. JAMA. 2008; 300:2123–33. [PubMed: 18997197]
34. Hennekens CH, Buring JE, Manson JE, et al. Lack of effect of long-term supplementation with beta carotene on the incidence of malignant neoplasms and cardiovascular disease. N Engl J Med. 1996; 334:1145–9. [PubMed: 8602179]
35. Steering Committee of the Physicians' Health Study Research Group. Final report on the aspirin component of the ongoing Physicians' Health Study. N Engl J Med. 1989; 321:129–35. [PubMed: 2664509]
36. Ederer F. Shall we count numbers of eyes or numbers of subjects? Arch Ophthalmol. 1973; 89:1–2. [PubMed: 4684894]

NIH-PA Author Manuscript NIH-PA Author Manuscript NIH-PA Author Manuscript

37. Glynn RJ, Rosner B. Accounting for the correlation between fellow eyes in regression analysis. Arch Ophthalmol. 1992; 110:381–7. [PubMed: 1543458]

38. Eye Diseases Prevalence Research Group. Prevalence of cataract and pseudophakia/aphakia among adults in the United States. Arch Ophthalmol. 2004; 122:487–94. [PubMed: 15078665]

39. National Eye Institute. [Accessed September 15, 2013.] National Plan for Eye and Vision Research: Lens and Cataract Program. Available at: http://www.nei.nih.gov/strategicplanning/np_lens.asp

40. Age-Related Eye Disease Study Research Group. Centrum use and progression of age-related cataract in the Age-Related Eye Disease Study: a propensity score approach. AREDS report no. 21. Ophthalmology. 2006; 113:1264–70. [PubMed: 16877067]

41. Chasan-Taber L, Willett WC, Seddon JM, et al. A prospective study of carotenoid and vitamin A intakes and risk of cataract extraction in US women. Am J Clin Nutr. 1999; 70:509–16. [PubMed: 10500020]

42. Moeller SM, Voland R, Tinker L, et al. CAREDS Study Group. Associations between age-related nuclear cataract and lutein and zeaxanthin in the diet and serum in the Carotenoids in the Age-Related Eye Disease Study, an ancillary study of the Women's Health Initiative. Arch Ophthalmol. 2008; 126:354–64. [PubMed: 18332316]

43. Mares-Perlman JA, Brady WE, Klein BE, et al. Serum carotenoids and tocopherols and severity of nuclear and cortical opacities. Invest Ophthalmol Vis Sci. 1995; 36:276–88. [PubMed: 7843899]

44. Brown L, Rimm EB, Seddon JM, et al. A prospective study of carotenoid intake and risk of cataract extraction in US men. Am J Clin Nutr. 1999; 70:517–24. [PubMed: 10500021]

45. Vu HT, Robman L, Hodge A, et al. Lutein and zeaxanthin and the risk of cataract: the Melbourne visual impairment project. Invest Ophthalmol Vis Sci. 2006; 47:3783–6. [PubMed: 16936087]

46. Lyle BJ, Mares-Perlman JA, Klein BE, et al. Antioxidant intake and risk of incident age-related nuclear cataracts in the Beaver Dam Eye Study. Am J Epidemiol. 1999; 149:801–9. [PubMed: 10221316]

47. Christen WG, Liu S, Glynn RJ, et al. Dietary carotenoids, vitamins C and E, and risk of cataract in women: a prospective study. Arch Ophthalmol. 2008; 126:102–9. [PubMed: 18195226]

48. Bone RA, Landrum JT, Mayne ST, et al. Macular pigment in donor eyes with and without AMD: a case-control study. Invest Ophthalmol Vis Sci. 2001; 42:235–40. [PubMed: 11133874]

49. Moeller SM, Parekh N, Tinker L, et al. CAREDS Research Study Group. Associations between intermediate age-related macular degeneration and lutein and zeaxanthin in the Carotenoids in Age-related Eye Disease Study (CAREDS): ancillary study of the Women's Health Initiative. Arch Ophthalmol. 2006; 124:1151–62. [PubMed: 16908818]

50. Olmedilla B, Granado F, Blanco I, Vaquero M. Lutein, but not alpha-tocopherol, supplementation improves visual function in patients with age-related cataracts: a 2-y double-blind, placebo-controlled pilot study. Nutrition. 2003; 19:21–4. [PubMed: 12507634]

51. Arnold C, Winter L, Frohlich K, et al. Macular xanthophylls and omega-3 long-chain polyunsaturated fatty acids in age-related macular degeneration: a randomized trial. JAMA Ophthalmol. 2013; 131:564–72. [PubMed: 23519529]

52. Piermarocchi S, Saviano S, Parisi V, et al. CARMIS Study Group. Carotenoids in Age-related Maculopathy Italian Study (CARMIS): two-year results of a randomized study. Eur J Ophthalmol. 2012; 22:216–25. [PubMed: 22009916]

53. Ma L, Yan SF, Huang YM, et al. Effect of lutein and zeaxanthin on macular pigment and visual function in patients with early age-related macular degeneration. Ophthalmology. 2012; 119:2290–7. [PubMed: 22858124]

54. Beatty S, Chakravarthy U, Nolan JM, et al. Secondary outcomes in a clinical trial of carotenoids with coantioxidants versus placebo in early age-related macular degeneration. Ophthalmology. 2013; 120:600–6. [PubMed: 23218821]

55. Berrow EJ, Bartlett HE, Eperjesi F, Gibson JM. The effects of a lutein-based supplement on objective and subjective measures of retinal and visual function in eyes with age-related maculopathy -- a randomised controlled trial. Br J Nutr. 2013; 109:2008–14. [PubMed: 23084077]

NIH-PA Author Manuscript
NIH-PA Author Manuscript
NIH-PA Author Manuscript

56. Chew EY, SanGiovanni JP, Ferris FL, et al. Age-Related Eye Disease Study 2 (AREDS2) Research Group. Lutein/zeaxanthin for the treatment of age-related cataract: AREDS2 randomized trial report no. 4. JAMA Ophthalmol. 2013; 131:843–50. [PubMed: 23645227]

57. Age-Related Eye Disease Study 2 Research Group. Lutein + zeaxanthin and omega-3 fatty acids for age-related macular degeneration: the Age-Related Eye Disease Study 2 (AREDS2) randomized clinical trial. JAMA. 2013; 309:2005–15. [PubMed: 23644932]

NIH-PA Author Manuscript
NIH-PA Author Manuscript
NIH-PA Author Manuscript

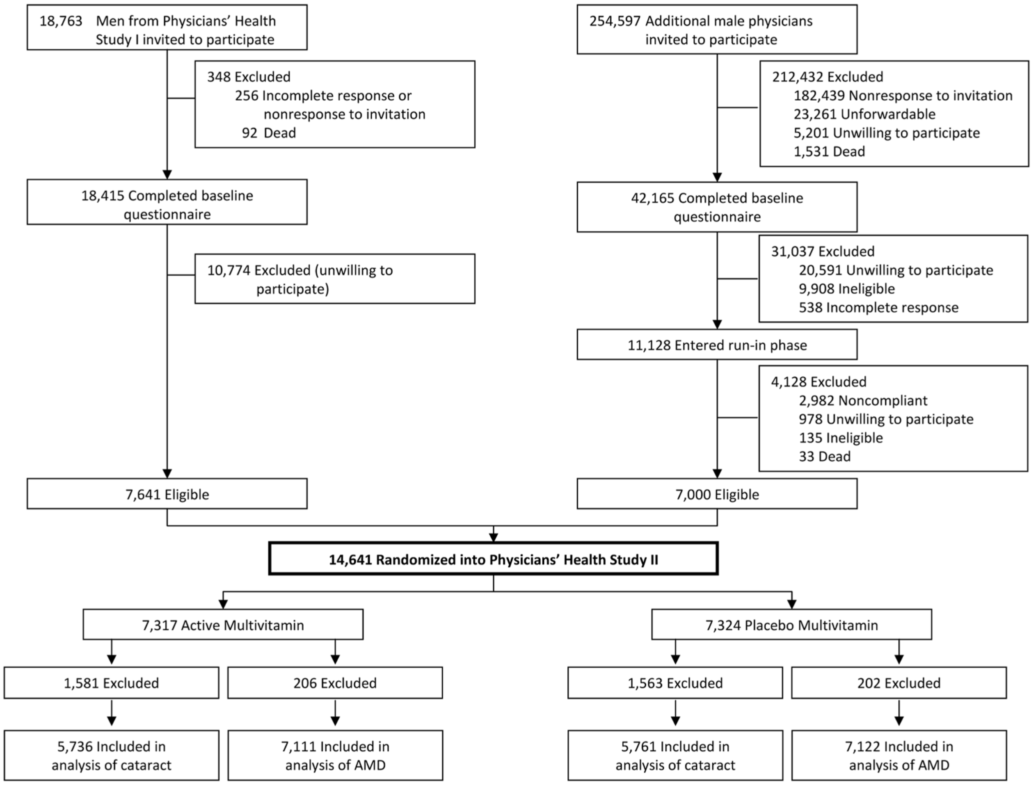

Figure 1.
Flow diagram of the multivitamin component of the Physicians' Health Study II. A total of 3,144 participants who reported a diagnosis of cataract at baseline, and 408 participants who reported a diagnosis of age-related macular degeneration at baseline, were excluded. Abbreviation: AMD, age-related macular degeneration.

NIH-PA Author Manuscript

NIH-PA Author Manuscript

NIH-PA Author Manuscript

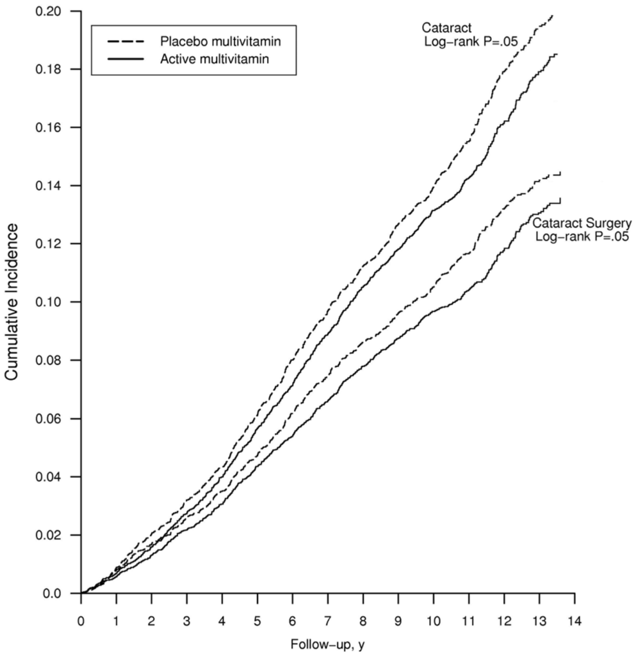

No. at Risk of Cataract

Placebo	5761	5687	5594	5483	5373	5212	5050	4897	4734	4588	4451	2758	1843	1615
Active	5736	5668	5582	5483	5376	5224	5100	4939	4805	4671	4536	2835	1905	1656

No. at Risk of Cataract Surgery

Placebo	5761	5691	5614	5516	5420	5290	5150	5017	4874	4749	4628	2901	1977	1746
Active	5736	5675	5598	5515	5429	5297	5195	5064	4951	4833	4712	2985	2023	1765

Figure 2.

Cumulative incidence rates of cataract in the multivitamin and placebo groups in the Physicians' Health Study II.

NIH-PA Author Manuscript
NIH-PA Author Manuscript
NIH-PA Author Manuscript

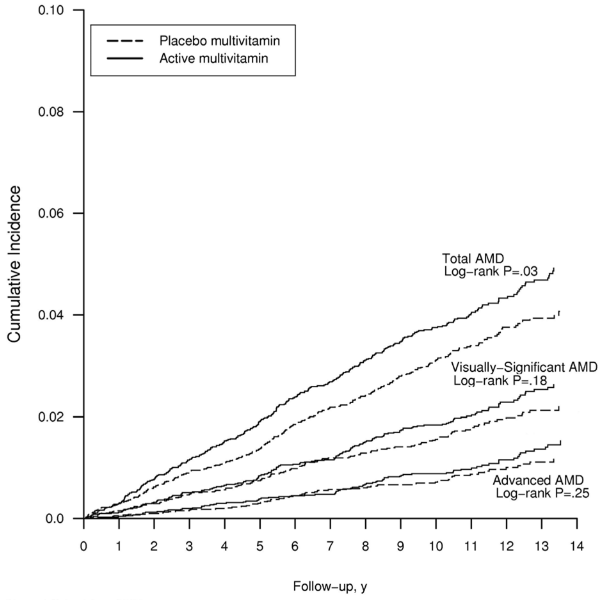

No. at Risk of Any AMD

Placebo	7122	7055	6979	6873	6784	6663	6537	6397	6257	6111	5956	3953	2768	2452
Active	7111	7043	6951	6865	6769	6646	6536	6404	6270	6132	5992	3979	2774	2461

No. at Risk of Visually−Significant AMD

Placebo	7122	7065	7002	6903	6819	6706	6596	6464	6331	6200	6053	4034	2840	2522
Active	7111	7055	6983	6910	6830	6720	6626	6506	6374	6242	6110	4078	2855	2536

No. at Risk of Advanced AMD

Placebo	7122	7072	7016	6925	6844	6736	6632	6505	6375	6246	6103	4076	2875	2555
Active	7111	7061	6996	6931	6853	6748	6664	6546	6424	6300	6170	4131	2893	2570

Figure 3.
Cumulative incidence rates of age-related macular degeneration in the multivitamin and placebo groups in the Physicians' Health Study II. Abbreviation: AMD, age-related macular degeneration.

NIH-PA Author Manuscript NIH-PA Author Manuscript NIH-PA Author Manuscript

Table 1

Baseline Characteristics by Multivitamin Randomized Treatment Assignment in Physicians' Health Study II.[a]

	Cataract			Visually-significant AMD		
	**No. Of Participants[b]**	**Multivitamin (n=5,736)**	**Placebo (n=5,761)**	**No. Of Participants**	**Multivitamin (n=7,111)**	**Placebo (n=7,122)**
Age (mean [SD], y)		61.9 (7.9)	62.0 (7.9)		63.9 (8.9)	64.0 (9.0)
Age, y						
50–59 years	5,6 06	49.1	48.5	5,865	41.2	41.2
60–69 years	3,914	33.8	34.3	4,647	32.8	32.5
≥70 years	1,977	17.2	17.2	3,721	26.0	26.3
Cigarette smoking						
Never	6,824	60.0	58.8	8,072	57.1	56.4
Former	4,264	36.7	37.6	5,642	39.4	39.9
Current	400	3.4	3.6	507	3.5	3.6
Alcohol use						
Rarely/never	2,108	18.9	18.0	2,650	19.2	18.3
≥1 drink/month	9,317	81.1	82.0	11,492	80.8	81.7
Body mass index (kg/m^2)						
<25	4,717	41.3	40.8	5,929	41.8	41.5
25 to <30	5,551	47.8	48.7	6,817	47.8	48.0
≥30	1,228	10.9	10.5	1,484	10.4	10.5
History of hypertension[c]						
Yes	4,426	38.1	39.4	5,896	41.0	42.3
No	6,998	61.9	60.6	8,258	59.0	57.7
History of high cholesterol[d]						
Yes	3,979	35.5	36.5	5,048	36.1	37.3
No	7,076	64.5	63.5	8,706	63.9	62.7
History of diabetes						
Yes	547	4.8	4.8	853	6.3	5.7
No	10,940	95.2	95.2	13,365	93.7	94.3
Aspirin use						

	Cataract			Visually-significant AMD		
	No. Of Participants[b]	Multivitamin (n=5,736)	Placebo (n=5,761)	No. Of Participants	Multivitamin (n=7,111)	Placebo (n=7,122)
Yes	8,742	77.7	76.6	10,868	77.7	77.2
No	2,589	22.3	23.4	3,158	22.3	22.8
Exercise ≥1 time/wk						
Yes	7,023	63.3	61.9	8,565	62.5	60.9
No	4,194	36.7	38.1	5,319	37.5	39.1
Self-reported history of CVD[e]						
Yes	454	3.7	4.2	717	5.0	5.0
No	11,043	96.3	95.8	13,516	95.0	95.0

Abbreviations: AMD, age-related macular degeneration; SD, standard deviation; CVD, cardiovascular disease.

[a]Data are given as percentage of participants unless otherwise noted

[b]For some variables, numbers do not total 11,497 for cataract and 14,233 for AMD because of missing data for that variable.

[c]History of hypertension was defined as self-reported systolic blood pressure of at least 140 mmHg, diastolic blood pressure of at least 90 mmHg, or past or current treatment for hypertension.

[d]History of high cholesterol was defined as self-reported total cholesterol level of at least 240 mg/dL or past or current treatment for high cholesterol.

[e]History of CVD included nonfatal myocardial infarction or nonfatal stroke.

NIH-PA Author Manuscript NIH-PA Author Manuscript NIH-PA Author Manuscript

NIH-PA Author Manuscript

NIH-PA Author Manuscript

NIH-PA Author Manuscript

Table 2

Confirmed Cases of Age-related Cataract and Cataract Extraction According to Multivitamin Randomized Treatment Assignment in Physicians' Health Study II.

	Cataract						Cataract Extraction					
	**Active (n=5,736)**	**Placebo (n=5,761)**	**HR[a]**	**95% CI**	**P Value**	**P Trend[b]**	**Active (n=5,736)**	**Placebo (n=5,761)**	**HR[a]**	**95% CI**	**P**	**P Trend[b]**
Total Cataract												
50–59 years	203	209	0.97	0.80–1.17	0.74	0.29	151	145	1.04	0.83–1.31	0.73	0.18
60–69 years	407	436	0.94	0.82–1.07	0.33		291	329	0.88	0.75–1.03	0.12	
≥70 years	262	300	0.85	0.72–1.00	0.06		194	227	0.84	0.69–1.02	0.08	
Subtotal	872	945	0.91	0.83–0.99	0.04		636	701	0.89	0.80–0.99	0.04	
Any NS												
50–59 years	175	187	0.93	0.76–1.15	0.51	0.46	132	129	1.02	0.80–1.30	0.85	0.29
60–69 years	374	425	0.88	0.77–1.01	0.08		272	319	0.85	0.72–1.00	0.05	
≥70 years	251	288	0.85	0.72–1.01	0.06		186	215	0.85	0.70–1.04	0.11	
Subtotal	800	900	0.87	0.79–0.96	0.005		590	663	0.87	0.78–0.98	0.018	
Any Cortical												
50–59 years	75	79	0.94	0.69–1.30	0.72	0.27	56	54	1.03	0.71–1.50	0.86	0.11
60–69 years	169	169	1.00	0.81–1.24	0.98		123	130	0.95	0.74–1.21	0.66	
≥70 years	112	139	0.79	0.61–1.01	0.06		78	107	0.72	0.54–0.96	0.027	
Subtotal	356	387	0.90	0.78–1.04	0.17		257	291	0.87	0.73–1.03	0.10	
Any PSC												
50–59 years	84	69	1.21	0.88–1.66	0.24	0.18	75	55	1.36	0.96–1.92	0.08	0.13
60–69 years	102	112	0.91	0.70–1.20	0.51		85	96	0.89	0.66–1.19	0.42	
≥70 years	61	67	0.89	0.63–1.26	0.50		53	56	0.93	0.64–1.35	0.69	
Subtotal	247	248	0.98	0.82–1.17	0.85		213	207	1.02	0.84–1.23	0.86	

Abbreviations: HR, hazard ratio; CI, confidence interval; NS, nuclear sclerosis; PSC, posterior subcapsular.

[a] Adjusted for age, Physicians' Health Study cohort, and vitamin C, vitamin E, and beta-carotene treatment assignment.

[b] Test for trend of the effect of age on the association between randomized treatment assignment and cataract.

NIH-PA Author Manuscript

Table 3

Relative Rates of Cataract and Visually-Significant Age-related Macular Degeneration by Randomized Treatment Assignment Within Subgroups in Physicians' Health Study II.[a]

	Cataract				Age-related Macular Degeneration			
	No.of Cataract/Total				No.of AMD/Total			
	Active	Placebo	HR (95% CI)[b]	P Interaction[c]	Active	Placebo	HR (95% CI)[b]	P Interaction[c]
Cigarette smoking								
Never	477/3437	504/3387	0.93 (0.82–1.05)	0.45	64/4055	57/4017	1.08 (0.76–1.55)	0.75
Former	366/2100	401/2164	0.91 (0.79–1.05)		82/2799	67/2843	1.30 (0.94–1.79)	
Current	29/192	40/208	0.70 (0.43–1.13)		6/249	5/258	1.16 (0.35–3.83)	
Alcohol use								
Rarely/never	180/1079	164/1029	0.99 (0.80–1.23)	0.43	37/1356	20/1294	1.62 (0.94–2.80)	0.17
≥1 drink/month	687/4618	776/4699	0.89 (0.80–0.99)		115/5705	107/5787	1.11 (0.85–1.44)	
Body mass index (kg/m^2)								
<25	374/2369	411/2348	0.89 (0.77–1.02)	0.71	66/2976	59/2953	1.11 (0.78–1.58)	0.87
25 to <30	399/2744	443/2807	0.90 (0.79–1.03)		73/3396	60/3421	1.24 (0.88–1.75)	
≥30	99/623	91/605	1.02 (0.77–1.35)		13/739	10/745	1.27 (0.56–2.91)	
History of hypertension								
Yes	399/2167	434/2259	0.94 (0.82–1.07)	0.63	75/2899	58/2997	1.33 (0.94–1.87)	0.35
No	470/3525	506/3473	0.89 (0.79–1.01)		77/4163	71/4095	1.07 (0.78–1.48)	
History of high cholesterol								
Yes	349/1958	361/2021	0.98 (0.84–1.13)	0.19	51/2485	45/2563	1.17 (0.78–1.75)	0.99
No	508/3560	572/3516	0.86 (0.76–0.97)		98/4395	83/4311	1.16 (0.87–1.56)	
History of diabetes								
Yes	56/273	55/274	0.98 (0.68–1.43)	0.41	8/446	9/407	0.85 (0.33–2.22)	0.56
No	816/5458	890/5482	0.90 (0.82–0.99)		144/6658	120/6707	1.21 (0.95–1.54)	
Exercise ≥1 time/wk								
Yes	503/3544	543/3479	0.91 (0.80–1.02)	0.98	83/4340	85/4225	0.97 (0.72–1.31)	0.055
No	361/2055	394/2139	0.91 (0.79–1.05)		67/2604	44/2715	1.56 (1.06–2.28)	
Self-reported history of CVD								
Yes	40/212	45/242	0.98 (0.64–1.50)	0.91	10/358	10/359	0.93 (0.39–2.24)	0.59

NIH-PA Author Manuscript NIH-PA Author Manuscript NIH-PA Author Manuscript

	Cataract				Age-related Macular Degeneration			
	No.of Cataract/Total				No.of AMD/Total			
	Active	Placebo	HR (95% CI)[b]	P Interaction[c]	Active	Placebo	HR (95% CI)[b]	P Interaction[c]
No	832/5524	900/5519	0.91 (0.82–1.00)		142/6753	119/6763	1.21 (0.95–1.54)	
Randomized to vitamin C								
Yes	455/2889	452/2879	1.00 (0.88–1.14)	0.04	75/3574	65/3575	1.15 (0.82–1.60)	0.79
No	417/2847	493/2882	0.82 (0.72–0.94)		77/3537	64/3547	1.22 (0.88–1.71)	
Randomized to vitamin E								
Yes	437/2869	479/2871	0.89 (0.78–1.01)	0.67	75/3537	69/3574	1.12 (0.81–1.55)	0.61
No	435/2867	466/2890	0.93 (0.81–1.06)		77/3574	60/3548	1.26 (0.90–1.77)	
Randomized to beta-carotene								
Yes	421/2885	439/2872	0.94 (0.82–1.07)	0.53	71/3591	60/3580	1.20 (0.85–1.69)	0.92
No	451/2851	506/2889	0.88 (0.78–1.00)		81/3520	69/3542	1.17 (0.85–1.62)	
Aspirin use								
Yes	708/4403	759/4339	0.89 (0.80–0.99)	0.50	121/5460	100/5408	1.22 (0.93–1.58)	0.61
No	162/1265	183/1324	0.97 (0.78–1.20)		30/1564	29/1594	1.03 (0.62–1.72)	

Abbreviations: HR, relative risk; CI, confidence interval; CVD cardiovascular disease; AMD, age-related macular degeneration.

[a] Baseline factors are defined as in Table 1.

[b] Adjusted for age, Physicians' Health Study cohort, and vitamin C, vitamin E, and beta-carotene treatment assignment.

[c] Test of the null hypothesis of no difference in treatment effect across risk factor subgroups.

NIH-PA Author Manuscript
NIH-PA Author Manuscript
NIH-PA Author Manuscript

Table 4

Confirmed Cases of Age-related Macular Degeneration According to Multivitamin Randomized Treatment Assignment in Physicians' Health Study II.

	Active (n=7,111)	Placebo (n=7,122)	HR[a]	95% CI	P	P-Trend[b]
Visually-Significant AMD						
50–59 years	6	8	0.76	0.26–2.18	0.61	0.43
60–69 years	41	36	1.14	0.73–1.78	0.58	
≥70 years	105	85	1.24	0.93–1.65	0.14	
Total	152	129	1.19	0.94–1.50	0.15	
Total AMD						
50–59 years	28	29	0.98	0.58–1.64	0.93	0.13
60–69 years	97	90	1.07	0.80–1.43	0.64	
≥70 years	169	125	1.38	1.09–1.73	0.007	
Total	294	244	1.22	1.03–1.44	0.02	
Advanced AMD						
50–59 years	3	7	0.43	0.11–1.67	0.22	0.14
60–69 years	21	18	1.15	0.61–2.17	0.66	
≥70 years	55	40	1.38	0.92–2.08	0.12	
Total	79	65	1.22	0.88–1.70	0.23	

Abbreviations HR, hazard ratio; CI, confidence interval; AMD, age-related macular degeneration.

[a]Adjusted for age, Physicians' Health Study cohort, and vitamin C, vitamin E, and beta-carotene treatment assignment.

[b]Test for trend of the effect of age on the association between randomized treatment assignment and AMD.
