# Supplementary material for: Insights into the nutritional prevention of macular degeneration based on a comparative topic modeling approach
Source: PeerJ Comput Sci. 2024 Mar 20;10:e1940. doi: 10.7717/peerj-cs.1940 (PMC11042009; doi:10.7717/peerj-cs.1940)
Supplement: Supplemental Information 3 [file peerj-cs-10-1940-s003.zip › negative_result_reports/755.full.pdf]

BMJ

RESEARCH

Dietary antioxidants and primary prevention of age related macular degeneration: systematic review and meta-analysis

Elaine W-T Chong, PhD candidate,[1] Tien Y Wong, professor of ophthalmology,[1] Andreas J Kreis, ophthalmology fellow,[1] Julie A Simpson, senior lecturer,[2] Robyn H Guymer, associate professor of ophthalmology[1]

[1]Centre for Eye Research Australia, University of Melbourne, Victoria 3002, Australia

[2]Centre for Molecular, Environmental, Genetic and Analytic Epidemiology, University of Melbourne

Correspondence to: T Y Wong, twong@unimelb.edu.au

doi:10.1136/bmj.39350.500428.47

ABSTRACT

Objective To evaluate the effectiveness of dietary antioxidants in the primary prevention of age related macular degeneration (AMD).

Design Systematic review and meta-analysis.

Data sources Search of seven databases without limits on year or language of publication, and retrieval of references in pertinent reviews and articles.

Methods Two reviewers independently searched the databases and selected the studies, using standardised criteria. Randomised clinical trials and prospective cohort studies were included. Of the 4192 abstracts initially identified, 12 studies (nine prospective cohort studies and three randomised clinical trials) met the selection criteria and were included. Data extraction and study quality evaluation were independently reviewed, using standardised criteria. Results were pooled quantitatively using meta-analytic methods.

Results The nine prospective cohort studies included 149 203 people, with 1878 incident cases of early AMD. The antioxidants investigated differed across studies, and not all studies contributed to the meta-analysis of each antioxidant. Pooled results from prospective cohort studies indicated that vitamin A, vitamin C, vitamin E, zinc, lutein, zeaxanthin, α carotene, β carotene, β cryptoxanthin, and lycopene have little or no effect in the primary prevention of early AMD. The three randomised clinical trials did not show that antioxidant supplements prevented early AMD.

Conclusions There is insufficient evidence to support the role of dietary antioxidants, including the use of dietary antioxidant supplements, for the primary prevention of early AMD.

INTRODUCTION

Age related macular degeneration (AMD) is the leading cause of severe visual loss in people aged over 50 in the developed world.[1-7] Early AMD is characterised clinically by yellow deposits known as drusen and changes in pigmentation of the retina. Late AMD develops when there is an ingrowth of new blood vessels that bleed into the subretinal space (exudative or "wet" type) or when the macula atrophies (geographic atrophy or "dry" type). Both these conditions usually lead to severe loss of central vision. The pathogenesis of AMD is unclear[8 9]; older age, genetic markers,[10 11] and cigarette smoking are the only risk factors consistently reported.[12-15] Although new treatments have emerged, they are suitable only for the small proportion of people with "wet" AMD.[16-19] No treatments are available for the "dry" form, and there is little to offer for the primary prevention of AMD in older people.

Dietary antioxidants have long been suggested as useful for preventing the development and progression of AMD.[20] The retina, with its high oxygen content and constant exposure to light, is particularly susceptible to oxidative damage.[21] A large randomised clinical trial, the age related eye disease study (AREDS),[22] showed that patients with intermediate AMD treated with high dose antioxidant supplements (vitamins C and E, zinc, and β carotene) had a 28% reduction in the risk of progression to advanced AMD compared with placebo (odds ratio 0.72, 99% confidence interval 0.52 to 0.98). That study did not specifically examine whether antioxidant supplements were effective for the primary prevention of early AMD in people without signs of this condition.[20]

Because oxidative damage could cause drusen to form,[23] antioxidants may be beneficial in the earliest stage of AMD. Randomised control trials and observational studies have been conducted in well nourished Western populations, but evidence of the role of dietary antioxidants as a primary preventive measure for AMD remains unclear. Some studies,[24 w1] but not others,[w2 w3] indicate that diets rich in antioxidants may protect against the development of signs of early AMD, and the common perception is that a diet rich in antioxidants can protect against AMD.[21 25-30]

We performed a systematic review and meta-analysis of the role of a range of dietary antioxidants—vitamins A, C, and E; zinc; lutein and zeaxanthin; α carotene; β carotene; β cryptoxanthin; and lycopene—in the primary prevention of AMD. We considered only randomised clinical trials and prospective cohort studies for inclusion.

METHODS

Data sources

We conducted a systematic review of seven databases, including PubMed (1950 to February 2007), Web of

BMJ: first published as 10.1136/bmj.39350.500428.47 on 8 October 2007. Downloaded from http://www.bmj.com/ on 17 July 2023 by guest. Protected by copyright.

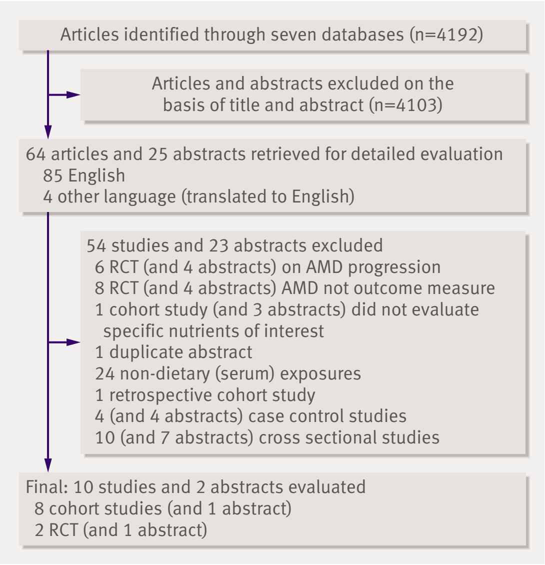

Fig 1 | **Flow chart of study selection process**

Science (1900 to February 2007), Embase (<1966 to February 2007), Medline (1950 to February 2007), Cochrane library (including the Cochrane Central Register of Controlled Trials, 1800 to February 2007), abstracts from the Association for Research in Vision and Ophthalmology (ARVO; 1962 to February 2007), and the National Institutes of Health clinical trial databases[31] (up to February 2007).

Systematic search of these databases used the terms "diet *or* nutrition *or* supplement* *or* carotenoids *or* antioxidants *or* trace elements *or* trace minerals *or* vitamin* *or* zinc *or* selenium *or* iron *or* copper *or* lutein *or* zeaxanthin *or* beta carotene* *or* carotene* *or* lycopene *or* vegetables *or* fruits" *and* "age-related macular degeneration *or* age related maculopathy *or* macular degeneration *or* retinal degeneration *or* drusen *or* choroidal neovascularization *or* geographic atrophy". The search strategy used both keywords and MeSH terms. No limits were placed on the year or language of publication. All articles in other languages were translated to English. References identified from bibliographies of pertinent articles or books were also retrieved.

Studies and participants

Randomised control trials and prospective cohort studies evaluating dietary antioxidants or antioxidant supplements in the primary prevention of AMD (that is, no disease to early or late AMD) were considered for inclusion. We specifically excluded studies in which all participants had early AMD, as these studies evaluated antioxidants for secondary prevention of AMD (progression of early to late AMD).

For studies to be included, five criteria needed to be met: a clear definition of exposure (dietary intake of vitamin A, vitamin C, vitamin E, zinc, lutein and zeaxanthin, α carotene, β carotene, β cryptoxanthin, lycopene); participant follow-up for one year or longer; clear definition of AMD as the outcome; appropriate statistical techniques to adjust for key potential confounders (for example, age and cigarette smoking); and estimates of odds ratio, relative risk, or the primary data to calculate these ratios. In studies that did not present an odds ratio or relative risk comparing highest to lowest fifth or fourth of intake, we contacted authors for this information.[w1 w4]

Outcome measures

The primary study outcome was early AMD (defined as soft drusen with or without retinal pigmentation changes), and late AMD (wet or dry AMD) was the secondary study outcome.

Selection of studies

Two reviewers (EW-TC and AJK) independently searched the seven databases, which included grey literature (unpublished work with limited distribution, such as conference abstracts; excluding grey literature that meets the pre-specified inclusion criteria from meta-analyses results in exaggerated effect sizes[32]). The search strategy found 4192 abstracts. We excluded studies if the title and abstract were not relevant, and obtained papers for all potentially relevant studies if the abstract contained insufficient information for exclusion.

Data extraction and study quality

Data extraction and evaluation of the study's quality were done independently by two reviewers (EW-TC and AJK). Data were extracted using a standardised extraction form, and methodological quality was assessed by using the validated Downs and Black instrument for cohort studies,[33 34] one of the best tools for assessing observational study designs.[35] The QUOROM statement checklist was used for randomised control trials.[36] The scores from these instruments were rated as high, moderate, or low quality. Disagreement between the reviewers was resolved by discussion with senior investigators (TW and RG). For two studies, the authors were contacted successfully to clarify details or to provide additional information about their study.[w1 w4]

Data synthesis

All meta-analyses were done with RevMan 4.2.8 software (www.cc-ims.net/RevMan), using fully adjusted odds ratio or relative risk in the meta-analyses. The standard error of the natural logarithm (ln) of the odds ratio was calculated from the 95% confidence intervals using the formula

(ln[upper limit of CI]–ln[lower limit of CI])/3.92).

Heterogeneity between studies was tested with the I^2 statistic.[37] If the I^2 statistic was ≤30% the fixed effect model was used to pool studies; otherwise, the random effects model was used. Sensitivity analyses, excluding unpublished abstracts and including only studies rated as high quality, were performed where possible. Where possible, we evaluated publication bias by

BMJ: first published as 10.1136/bmj.39350.500428.47 on 8 October 2007. Downloaded from http://www.bmj.com/ on 17 July 2023 by guest. Protected by copyright.

plotting a funnel plot[38]; publication bias is unlikely if the funnel plot shows a symmetrical inverted V shape.[39]

RESULTS

Description of studies

Of the 4192 abstracts screened, 89 were from potentially relevant studies, of which 77 were excluded because they did not meet the inclusion criteria (fig 1). The remaining 12 studies comprised nine prospective cohort studies (including one published only as an abstract) [w1-9] and three randomised control trials (including one abstract).[w10-w12] Reviewers agreed completely on study eligibility. Tables 1 and 2 summarise the design features and participants' characteristics in these studies.

Prospective cohort studies

When duplicate publications and abstracts were excluded, the nine prospective cohort studies selected comprised seven independent studies including 149 203 people and 1878 incident cases of early AMD. As the antioxidants investigated differed across the studies, not all studies contributed to the meta-analysis of every antioxidant. All of the cohort studies recruited participants between 1980 and 1994; in three studies,

Table 1 | Prospective cohort studies evaluating antioxidants and their association with the primary prevention of early age-related macular degeneration (AMD)

Author, year	Study	Follow-up	Population (sample size, age (years))	Definition of AMD	No of cases	Antioxidants investigated	Confounders adjusted for	Study quality
Christen, 1999[w5]	Physician health study 1	12.5 person years	Male doctors, USA (21 120, 40-84)	Drusen or pigment change, plus visual acuity ≤20/30	279	Brief questionnaire: Supplements	Age, smoking, treatment assignment group, body mass index, exercise, diabetes, family history of acute myocardial infarct, alcohol intake	Moderate
*Cho, 2001[w6]	Nurses health study and health professional follow-up study	8-10 year incidence	Health professionals, USA (104 208, ≥50)	Drusen or pigment change, plus visual acuity ≤20/30	195	Validated food frequency questionnaire: zinc	Age, smoking, energy, body mass index, exercise, lutein and zeaxanthin intake, 2 yr time period, hormone replacement therapy, blood pressure, cholesterol, alcohol intake	Moderate
Van Leeuwen, 2005[w1]	Rotterdam eye study	8 year incidence	Population based, Netherlands (4170, ≥55)	International classification	518	Validated food frequency questionnaire: lutein and zeaxanthin, vitamin E, vitamin C, vitamin A, zinc, β carotene, β cryptoxanthin, lycopene, α carotene	Age, smoking, sex, energy, fat intake, body mass index, blood pressure, cholesterol, alcohol	High
†Flood, 2002[w2]	Blue Mountain eye study	5 year incidence	Population based, Australia (1989, ≥49)	Wisconsin age related maculopathy grading system	192	Validated food frequency questionnaire: lutein and zeaxanthin, vitamin C, vitamin A, zinc, β carotene, β cryptoxanthin, lycopene, α carotene, supplements	Age, smoking, sex, energy, family history of AMD	High
Moeller, 2006[w7]	Carotenoid in age-related eye disease study	6 year prevalence	Women's Health Initiative: healthy volunteers (1787, 50-79) women only	AREDS modified	322	Validated food frequency questionnaire: lutein and zeaxanthin, fruit and vegetables	Age, smoking, energy, diabetes, family history of AMD, iris colour, cardiovascular disease, hormone replacement therapy	Moderate
Cho, 2004*[w3]	Nurses health study and health professional follow-up study	12-18 year incidence	Health professionals (118 428, ≥50)	Drusen or pigment change, plus visual acuity ≤20/30	464	Validated food frequency questionnaire: lutein and zeaxanthin, vitamin E, vitamin C, vitamin A, β carotene, β cryptoxanthin, lycopene, α carotene, fruit and vegetables, supplements	Age, smoking, energy, body mass index, exercise, fish intake, hormone replacement therapy, blood pressure, alcohol intake	Moderate
Van den Langenberg, 1998[w8]	Beaver Dam eye study	5 year incidence	Population based, USA (1709, 43-84)	Wisconsin age related maculopathy grading system	103	Validated food frequency questionnaire: lutein and zeaxanthin, vitamin E, C, β carotene, β cryptoxanthin, zinc, lycopene, α carotene, fruit and vegetables, supplements	Age, smoking, sex, energy, body mass index, exercise, diabetes, cardiovascular disease, beer intake	High
Flood, 2006†[w4]	Blue Mountain eye study	10 year incidence	Population based, Australia (2083, ≥49)	Wisconsin age related maculopathy grading system	220	Validated food frequency questionnaire: lutein and zeaxanthin	Age, smoking, sex, energy	Moderate
Chong, 2006[w9]	Melbourne collaborative cohort study	10 year prevalence	Volunteers for study (3605, ≥60)	International classification	1008	Validated food frequency questionnaire: lutein and zeaxanthin	Age, smoking, sex, energy, ethnicity, supplement use, total fat	Not applicable (abstract)

*The three studies that evaluated antioxidant intake and its associations with late AMD.
†Duplicate publication: Flood 2006 used in lutein and zeaxanthin pooled results instead of Flood 2002.

BMJ: first published as 10.1136/bmj.39350.500428.47 on 8 October 2007. Downloaded from http://www.bmj.com/ on 17 July 2023 by guest. Protected by copyright.

dietary data had been recorded before 1988 as they were a subset of another long term study.[w3 w5 w6] All studies were published in the past 10 years and were conducted in the United States or other Western countries. One cohort study included only women[w7] and another only men.[w5] In most studies, participants were 49 years or older, but two included participants in their early 40s.[w5 w8] Follow-up was 5-18 years (mean 9 years). Three studies were population based [w1 w2 w4 w8] and four included volunteers and health professionals. [w3 w5 w6 w7 w9] Most studies had initial participation rates of ≥80%; one study had a participation rate of 64% but reported no difference in prevalence of self reported AMD between participants and non-participants;[w7] another did not report the participation rates.[w5] The follow-up rates for most studies were over 75%. With the exception of the physicians' health study, which evaluated self reported use of antioxidant supplements (and did not contribute to the pooled results),[w5] all studies used previously validated food frequency questionnaires to evaluate intake of antioxidants.

The assessment and definition of AMD varied between studies (table 1). All studies adjusted for age and smoking in their analyses. Most studies analysed the risk of AMD by comparing the highest fifth or fourth of antioxidant intake to the lowest fifth or fourth; one study evaluated the risk of AMD per standard deviation increase of lutein and zeaxanthin intake, but the authors, when requested, provided the odds ratio for AMD comparing the highest fifth of lutein and zeaxanthin intake to the lowest fifth.[w4] The other author who we contacted provided us with a detailed spreadsheet of the hazard ratio of the various antioxidants that were investigated.[w1] The two reviewers agreed on the quality of the study in seven of the eight published cohort studies, and resolved disagreement by discussion.

Randomised controlled trials

Three randomised control trials, including one abstract, evaluated antioxidant supplementation in the primary prevention of AMD (table 2).[w10-w12] The vitamin E, cataract, and age related maculopathy trial (VECAT) evaluated vitamin E versus placebo supplementation in an Australian population, while the alpha tocopherol and beta carotene (ATBC) trial evaluated vitamin E or β carotene supplementation, or both, versus placebo in Finland. Neither of these trials found that antioxidant supplements were effective for primary prevention of AMD.

Dietary antioxidants and early AMD

Figures 2 and 3 show the point estimates for vitamin A, vitamin C, vitamin E, zinc, lutein and zeaxanthin, α carotene, β carotene, β cryptoxanthin, and lycopene in the different studies comparing the highest versus the lowest fifth or fourth of intake for early AMD.

For vitamin A, all three cohort studies that contributed to the pooled analysis reported null associations, and the pooled odds ratio of early AMD, in a comparison of the highest to the lowest vitamin A intake category, was 0.98 (95% confidence interval 0.81 to 1.18). For vitamin C, three of the four studies reported positive associations and one reported an inverse association. Because of heterogeneity between studies (P=0.15, I^2=43%), results were pooled by using the random effect model, and the pooled odds ratio was 1.11 (0.84 to 1.46). For vitamin E, of the three published cohort studies that contributed to the pooled results, two reported an inverse association and one a null association. The Rotterdam eye study,[w1] which reported a statistically significant finding, contributed 60% weight to the pooled result, an odds ratio of 0.83 (0.69 to 1.01). When we pooled results from the two high quality studies,[w1 w8] the odds ratio of vitamin E was 0.75 (0.59 to 0.94).

For zinc, two of the four studies reported positive associations, one reported a null association, and one an inverse association. The Rotterdam eye study reported a borderline significant finding and again dominated the pooled result (63% weight). The pooled

Table 2 | **Randomised controlled trials evaluating antioxidant supplementation and its association with the primary prevention of age related macular degeneration (AMD)**

Author, year	Study	Follow-up	Population (sample size, age (years))	Definition of AMD	No of cases	Antioxidants investigated	Relative risk (95% CI)	Randomisation and adequacy of allocation concealment
Taylor, 2002[w10]	Vitamin E, cataract, and age related maculopathy trial (VECAT)	4 year incidence	Population based, Australia (1193, 55-80)	Wisconsin age related maculopathy grading system (WARMGS) and international classification	69	Vitamin E 500 IU (335 mg/day) *v* placebo	Early AMD 1.05 (0.69 to 1.61); late AMD 1.36 (0.67 to 2.77)	High quality
Teikari, 1998[w11]	Alpha-tocopherol and beta-carotene study (ATBC)	6 year prevalence	Population based, Finland (941, ≥65)	Modified international classification	269	Vitamin E (50 mg/day), β carotene (20 mg/day) or both *v* placebo	Any AMD:Vitamin E only 1.27 (0.84 to 1.93); α carotene only 1.17 (0.76 to 1.79); both 1.14 (0.75 to 1.74)	Moderate quality
Christen, 2003[w12]	Physicians' health study	7-12 years	Male doctors, USA (21 216)	Drusen or pigment change, plus visual acuity ≤20/30	532	α carotene (50 mg every other day) *v* placebo	Any AMD 0.97 (0.82 to 1.15)	Insufficient information (abstract)

BMJ: first published as 10.1136/bmj.39350.500428.47 on 8 October 2007. Downloaded from http://www.bmj.com/ on 17 July 2023 by guest. Protected by copyright.

odds ratio of zinc for early AMD was 0.91 (0.74 to 1.11).

Six cohort studies contributed to the meta-analysis of lutein and zeaxanthin. Of these, four reported null associations, one a positive association, and one an inverse association. None of the findings in these studies was statistically significant, nor was the heterogeneity between studies (P=0.80, I^2=0%). Results were pooled by using the fixed effect model, and the odds ratio for participants in the highest relative to the lowest lutein and zeaxanthin intake category was 0.98 (0.86 to 1.13), and it was 0.96 (0.82 to 1.12) when we excluded results from the abstract from the model. The symmetrical shape of the funnel plot indicates that publication bias is unlikely (fig 4).[38 39]

Four published cohort studies evaluated the associations between α carotene, β carotene, β cryptoxanthin, and lycopene and early AMD and contributed to the pooled results of these antioxidants (fig 3). For α carotene, pooled results yielded an odds ratio of 1.05 (0.87 to 1.26). For β carotene, two of four studies reported null associations, one a positive association, and one an inverse association; none was significant. The Rotterdam eye study contributed greatly (53% weight) to an odds ratio of 1.04 (0.86 to 1.25). For β cryptoxanthin, the pooled odds ratio of four studies was 1.01 (0.85 to 1.22), and for lycopene it was 1.07 (0.90 to 1.28).

When sensitivity analyses including only population based, high quality studies for the nine investigated nutrients were performed the pooled odds ratio for the nutrients, apart from vitamin E, did not change greatly.

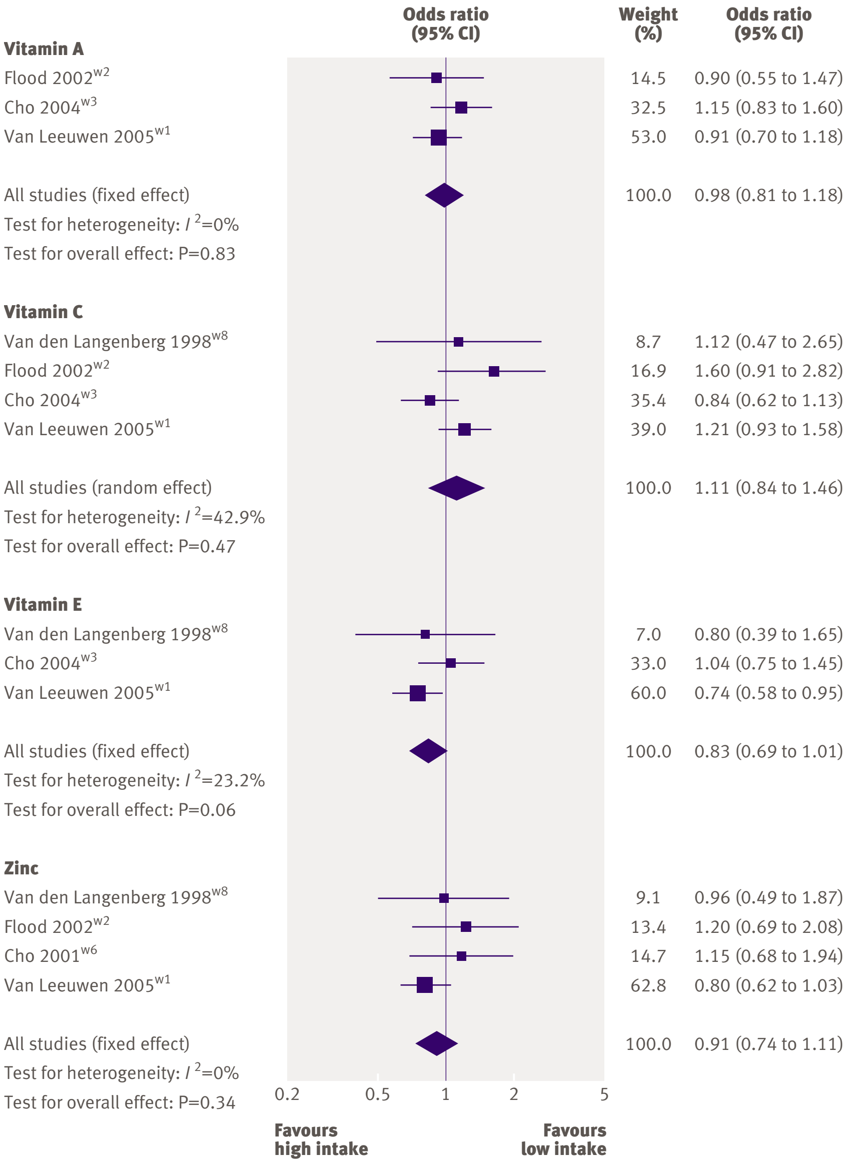

Fig 2 | Pooled odds ratio for early AMD (highest *v* lowest dietary intake categories of vitamins and zinc)

Dietary antioxidants and late AMD

Of the eight published cohort studies, only three provided point estimates for the risk of late AMD (table 1).[w3 w4 w6] As each evaluated different antioxidants, we were unable to pool these results. Cases were few and odds ratios had wide 95% confidence intervals.

DISCUSSION

Age related macular degeneration remains the leading cause of visual loss in the United Kingdom and other developed countries. Oxidative damage in the retina has been hypothesised as a key process involved in development of early AMD. Antioxidants are thought to prevent AMD by reducing the photo-oxidative damage from blue light in the oxygen filled environment of the retina, which is rich in polyunsaturated fatty acids that are highly susceptible to oxidation.[40]

Previous studies and reviews have largely focused on the role of dietary antioxidants and supplements in the secondary prevention of AMD—that is, preventing progression to late AMD in people with signs of early disease. Our analysis examined the role of dietary antioxidants and supplements in primary prevention and found that a range of dietary antioxidants, including vitamins A, C, and E, zinc, lutein and zeaxanthin, α carotene, β carotene, β cryptoxanthin, and lycopene, have little or no effect: pooled odds ratios ranged from 0.91 to 1.11, with the exception of vitamin E, which had a modest borderline protective association (0.83, 95% confidence interval 0.69 to 1.01).

Comparison with other studies

We found few randomised clinical trials, none of which found that vitamin E and β carotene supplements prevented early AMD.[w10-w12] The studies we evaluated were largely derived from populations in the United States or other developed Western nations, where participants are well nourished. Although we included both population based and volunteer based studies, the pooled odds ratio for the nutrients investigated, with the exception of vitamin E, did not change greatly in our sensitivity analyses of population based and high quality studies.

BMJ: first published as 10.1136/bmj.39350.500428.47 on 8 October 2007. Downloaded from http://www.bmj.com/ on 17 July 2023 by guest. Protected by copyright.

For vitamin E, the borderline significant pooled odds ratio, especially from the two high quality studies, suggests that vitamin E may be associated with a reduced risk of early AMD. Results from the two randomised control trials do not support a protective effect of vitamin E supplementation, given in doses 2.5-fold to 15-fold higher than the highest dietary range estimated from these cohort studies. The vitamin E, cataract, and age related maculopathy trial reported a relative risk for early AMD of 1.05 (0.69 to 1.61) comparing vitamin E intake of 335 mg/day versus placebo,[w10] and the alpha tocopherol and beta carotene trial reported an odds ratio for any AMD of 1.27 (0.84 to 1.93) for vitamin E intake of 50 mg/day versus placebo.[w11]

The alpha tocopherol and beta carotene trial reported an odds ratio for any AMD of 1.17 (0.76 to 1.79) for β carotene intake of 20 mg/day versus placebo,[w11] and the physicians' health study reported a relative risk of 0.97 (0.81 to 1.15) for β carotene (50 mg every other day) versus placebo.[w12] These results are consistent with data from prospective cohort studies. The pooled odds ratio of dietary β carotene intake for early AMD was 1.04 (0.85 to 1.27), comparing the highest (range of intake of 6.2-11.9 mg/day) to the lowest (2.1-2.3 mg/day) category of dietary β carotene.

Carotenoids have been shown to be good filters of harmful blue light, and their antioxidative properties have been demonstrated in vitro.[41] Two of these carotenoids, lutein and zeaxanthin, are found in the macula in concentrations higher than in other parts of the body.[42] However, results from our review suggest that high antioxidant levels in the healthy retina do little to prevent the development of early AMD. A Cochrane review showed that antioxidant supplements may have a role delaying the progression of early to late AMD.[43] These contrasting results could imply that uncontrolled oxidative chain reactions of reactive oxygen species may have begun in eyes with AMD at early or intermediate stage, and thus high antioxidant levels at this stage of the disease process may be effective in slowing progression of AMD. The Cochrane review reported a protective effect from multivitamin supplementation (pooled odds ratio 0.68, 99% confidence interval 0.49 to 0.93) and a protective effect of zinc supplementation (0.73, 95% confidence interval 0.58 to 0.93) in preventing the progression of AMD. Both findings were mainly based on data from the age related eye disease study.[22] No protective effect was seen for vitamin E supplementation (1.05, 0.80 to 1.55; derived only from the vitamin E, cataract, and age related maculopathy trial[w10]).[43]

Strengths and weaknesses of the study

We sought to be as comprehensive as possible, in accordance with guidelines for meta-analyses,[34 44] and performed an extensive search through seven databases, including grey literature, and did not limit our searches by language or time.[45 46] Inclusion of grey literature has also been shown to reduce the likelihood of publication bias[47] and of overestimating pooled estimates.[32] Although only six studies evaluated lutein and zeaxanthin, the funnel plot showed an inverted

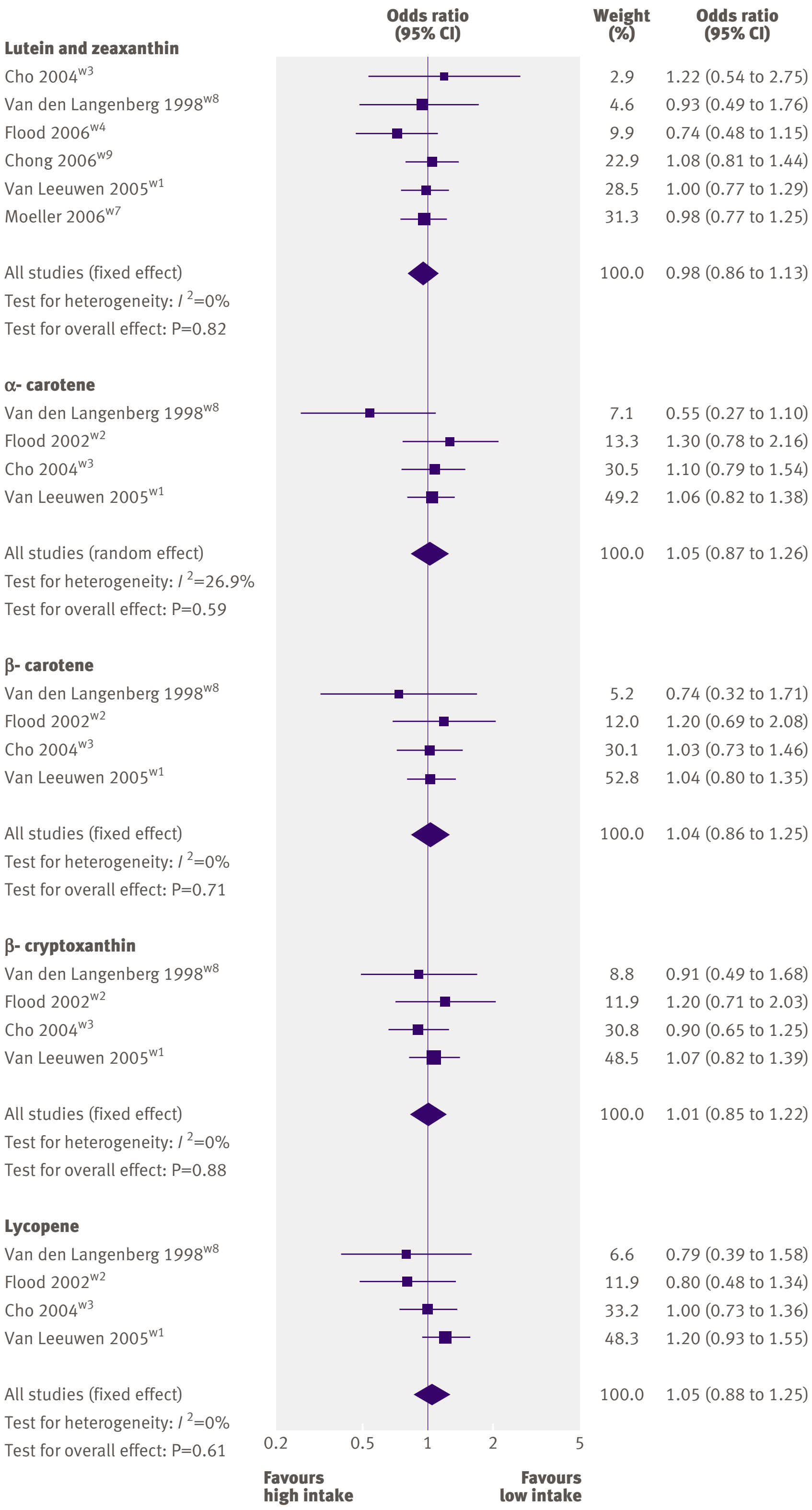

Fig 3 | Pooled odds ratio for early AMD (highest *v* lowest dietary intake categories of carotenoids)

BMJ: first published as 10.1136/bmj.39350.500428.47 on 8 October 2007. Downloaded from http://www.bmj.com/ on 17 July 2023 by guest. Protected by copyright.

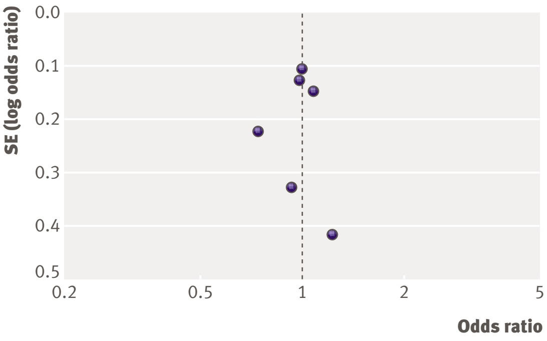

Fig 4 | **Funnel plot of the odds ratio (for highest versus lowest intake categories) versus the standard error of the log odds ratio for studies evaluating lutein and zeaxanthin. Dotted line crosses the x axis at the estimate of the pooled odds ratio for early AMD**

V shape, suggesting that publication bias was unlikely.[38 39] Furthermore, we had prespecified the inclusion criteria for the studies, and we specifically evaluated dietary antioxidant intakes and AMD outcomes. Studies that were included had adequate follow-up (most studies lasted a decade), had sound methods, and were of good quality. All studies with pooled risk estimates had been adjusted for age, cigarette smoking, and energy intake in the analyses and had results that were consistent with one another. Overall, there was also little heterogeneity between studies that were included in this review.

Limitations in published studies

Our review identified important limitations in the current literature. Firstly, we found few randomised controlled trials. Our meta-analysis did not include the results of the age related eye disease study, which evaluated the role of antioxidant supplements in the secondary prevention of AMD, a topic that is outside the scope of our review. Of the primary prevention trials, the two published randomised controlled trials evaluated only two potential antioxidants (vitamin E and β carotene) and had a relatively short follow-up of 4-6 years. Thus, prospective cohort studies provided the best currently available evidence regarding dietary antioxidants in the primary prevention of AMD. These prospective studies showed little heterogeneity, allowing us to conduct meta-analysis to derive pooled estimates of risk, but meta-analysis of observational data is known to have more biases than meta-analysis of randomised controlled trials.[48]

Secondly, we are unable to evaluate the effect of potential antioxidant synergism in our review. In the Rotterdam eye study, an above median dietary intake of combined antioxidant nutrients, similar to those found in the clinical trial formulation of the age related eye disease study, was associated with a larger reduced risk of AMD than for each antioxidant (vitamin C, vitamin E, β carotene, zinc) individually.[w1] We were unable to evaluate such potential synergistic effects with particular antioxidant intake combinations or ratios, as individual studies evaluated each antioxidant in isolation and we pooled the results for each antioxidant separately.

Thirdly, although the included studies had participation rates of greater than 80%, participants in some of these (healthcare professionals, for example) may not be representative of the wider community. Moreover, all studies were conducted in relatively well nourished populations in the United States and other developed Western countries, and results may not be generalisable to other countries.

Fourthly, the assessment and definition of AMD varied between studies (see table 1). Definitions ranged from those based on photographs to those that included visual acuity criteria. Studies that included visual acuity criteria may have included a smaller proportion of cases of early AMD than those studies with photographic documentation only.

Finally, most studies used food frequency questionnaires to assess dietary intakes of antioxidants, and these questionnaires were administered only once at study baseline. Non-differential misclassification of antioxidant intake may have occurred, which may bias the results towards the null.

Conclusion

Dietary intake of nine antioxidants evaluated in this systematic review had little or no effect in the primary prevention of early AMD in well nourished Western populations. There is insufficient evidence that antioxidants supplements prevent the onset of AMD. Cigarette smoking remains the only widely accepted modifiable risk factor for the primary prevention of AMD, and patients seeking advice on AMD prevention should be encouraged to stop smoking.

WHAT IS ALREADY KNOWN ABOUT THIS TOPIC

Age related macular degeneration (AMD) is the leading cause of visual loss in older people

Antioxidants have been hypothesised to reduce oxidative damage to the retina, but the effectiveness of dietary antioxidants in the primary prevention of AMD is unclear

WHAT THIS STUDY ADDS

Dietary antioxidants had little or no effect in the primary prevention of early AMD in well nourished Western populations

Cigarette smoking remains the only widely accepted modifiable risk factor for the primary prevention of AMD

We thank Rachel L McIntosh of the Centre for Eye Research Australia, the University of Melbourne, for her help in starting this systematic review and Liubov Robman, also of the centre, for comments and advice.

Contributors: All authors contributed to the concept, design and critical revision of the manuscript. EW-TC and AJK carried out the search, selected the articles and extracted the data. EW-TC carried out the statistical analyses with input from JAS. TYW had full access to all of the data in the study and takes responsibility for the integrity of the data and the accuracy of the data analysis.
Funding: EW-TC received a National Health and Medical Research Council public health scholarship, funded by the Federal Government of Australia. TYW and RHG are on advisory boards of Pfizer and Novartis and have received grants, honoraria, and travelling fees from these companies.
Competing interests: None declared.

BMJ: first published as 10.1136/bmj.39350.500428.47 on 8 October 2007. Downloaded from http://www.bmj.com/ on 17 July 2023 by guest. Protected by copyright.

Ethical approval: Not required.
Provenance and peer review: Not commissioned; externally peer reviewed.

1 Bressler NM. Age-related macular degeneration is the leading cause of blindness. *JAMA* 2004;291:1900-1.
2 Congdon NG, Friedman DS, Lietman T. Important causes of visual impairment in the world today. *JAMA* 2003;290:2057-60.
3 Van Newkirk MR, Nanjan MB, Wang JJ, Mitchell P, Taylor HR, McCarty CA. The prevalence of age-related maculopathy: the visual impairment project. *Ophthalmology* 2000;107:1593-600.
4 Mitchell P, Smith W, Attebo K, Wang JJ. Prevalence of age-related maculopathy in Australia. The Blue Mountains eye study. *Ophthalmology* 1995;102:1450-60.
5 Friedman DS, O'Colmain BJ, Munoz B, Tomany SC, McCarty C, de Jong PT, et al. Prevalence of age-related macular degeneration in the United States. *Arch Ophthalmol* 2004;122:564-72.
6 Klein R, Klein BE, Linton KL. Prevalence of age-related maculopathy. The Beaver Dam eye study. *Ophthalmology* 1992;99:933-43.
7 Owen CG, Fletcher AE, Donoghue M, Rudnicka AR. How big is the burden of visual loss caused by age related macular degeneration in the United Kingdom? *Br J Ophthalmol* 2003;87:312-7.
8 Arroyo JG. A 76-year-old man with macular degeneration. *JAMA* 2006;295:2394-406.
9 Chakravarthy U. Age related macular degeneration. *BMJ* 2006;333:869-70.
10 Haines JL, Hauser MA, Schmidt S, Scott WK, Olson LM, Gallins P, et al. Complement factor H variant increases the risk of age-related macular degeneration. *Science* 2005;308:419-21.
11 Baird PN, Richardson AJ, Robman LD, Dimitrov PN, Tikellis G, McCarty CA, et al. Apolipoprotein (APOE) gene is associated with progression of age-related macular degeneration (AMD). *Hum Mutat* 2006;27:337-42.
12 Thornton J, Edwards R, Mitchell P, Harrison RA, Buchan I, Kelly SP. Smoking and age-related macular degeneration: a review of association. *Eye* 2005;19:935-44.
13 Christen WG, Glynn RJ, Manson JE, Ajani UA, Buring JE. A prospective study of cigarette smoking and risk of age-related macular degeneration in men. *JAMA* 1996;276:1147-51.
14 Seddon JM, Willett WC, Speizer FE, Hankinson SE. A prospective study of cigarette smoking and age-related macular degeneration in women. *JAMA* 1996;276:1141-6.
15 Kelly SP, Thornton J, Lyratzopoulos G, Edwards R, Mitchell P. Smoking and blindness. *BMJ* 2004;328:537-8.
16 Eter N, Krohne TU, Holz FG. New pharmacologic approaches to therapy for age-related macular degeneration. *BioDrugs* 2006;20:167-79.
17 Gragoudas ES, Adamis AP, Cunningham ET Jr, Feinsod M, Guyer DR. Pegaptanib for neovascular age-related macular degeneration. *N Engl J Med* 2004;351:2805-16.
18 Rosenfeld PJ, Brown DM, Heier JS, Boyer DS, Kaiser PK, Chung CY, et al. Ranibizumab for neovascular age-related macular degeneration. *N Engl J Med* 2006;355:1419-31.
19 Brown DM, Kaiser PK, Michels M, Soubrane G, Heier JS, Kim RY, et al. Ranibizumab versus verteporfin for neovascular age-related macular degeneration. *N Engl J Med* 2006;355:1432-44.
20 Jampol LM, Ferris FL 3rd. Antioxidants and zinc to prevent progression of age-related macular degeneration. *JAMA* 2001;286:2466-8.
21 Seddon JM, Hennekens CH. Vitamins, minerals, and macular degeneration: promising but unproven hypotheses. *Arch Ophthalmol* 1994;112:176-9.
22 Age-Related Eye Disease Study Research Group. A randomized, placebo-controlled, clinical trial of high-dose supplementation with vitamins C and E, beta carotene, and zinc for age-related macular degeneration and vision loss: AREDS report No 8. *Arch Ophthalmol* 2001;119:1417-36.
23 Crabb JW, Miyagi M, Gu X, Shadrach K, West KA, Sakaguchi H, et al. Drusen proteome analysis: an approach to the etiology of age-related macular degeneration. *Proc Natl Acad Sci USA* 2002;99:14682-7.
24 Seddon JM, Ajani UA, Sperduto RD, Hiller R, Blair N, Burton TC, et al. Dietary carotenoids, vitamins A, C, and E, and advanced age-related macular degeneration. Eye Disease Case-Control Study Group. *JAMA* 1994;272:1413-20.
25 Mares JA, La Rowe TL, Blodi BA. Doctor, what vitamins should I take for my eyes? *Arch Ophthalmol* 2004;122:628-35.
26 Mares-Perlman JA, Millen AE, Ficek TL, Hankinson SE. The body of evidence to support a protective role for lutein and zeaxanthin in delaying chronic disease: overview. *J Nutr* 2002;132:518-524S.
27 Brody JE. Personal health: unveiling a new arsenal for eye health. *New York Times* 2001 March 13. http://query.nytimes.com/gst/fullpage.html?res=9F06EEDE103AF930A25750C0A9679C8B63&sec=health&pagewanted=2.
28 Horowitz JM. Your health. *Time Magazine* 2001 October 29. www.time.com/time/magazine/article/0,9171,1001096,00.html.
29 Chopdar A, Chakravarthy U, Verma D. Age related macular degeneration. *BMJ* 2003;326:485-8.
30 Hall NF, Gale CR. Prevention of age related macular degeneration. *BMJ* 2002;325:1-2.
31 National Institutes of Health. Clinical Trials Database. http://clinicaltrials.gov.
32 McAuley L, Pham B, Tugwell P, Moher D. Does the inclusion of grey literature influence estimates of intervention effectiveness reported in meta-analyses? *Lancet* 2000;356:1228-31.
33 Downs SH, Black N. The feasibility of creating a checklist for the assessment of the methodological quality both of randomised and non-randomised studies of health care interventions. *J Epidemiol Community Health* 1998;52:377-84.
34 Stroup DF, Berlin JA, Morton SC, Olkin I, Williamson GD, Rennie D, et al. Meta-analysis of observational studies in epidemiology: a proposal for reporting. Meta-analysis Of Observational Studies in Epidemiology (MOOSE) group. *JAMA* 2000;283:2008-12.
35 Deeks JJ, Dinnes J, D'Amico R, Sowden AJ, Sakarovitch C, Song F, et al. Evaluating non-randomised intervention studies. *Health Technol Assess* 2003;7:iii-x, 1-173.
36 Clarke M. The QUOROM statement. *Lancet* 2000;355:756-7.
37 Higgins JP, Thompson SG, Deeks JJ, Altman DG. Measuring inconsistency in meta-analyses. *BMJ* 2003;327:557-60.
38 Sterne JA, Egger M. Funnel plots for detecting bias in meta-analysis: guidelines on choice of axis. *J Clin Epidemiol* 2001;54:1046-55.
39 Egger M, Davey Smith G, Schneider M, Minder C. Bias in meta-analysis detected by a simple, graphical test. *BMJ* 1997;315:629-34.
40 Kirschfeld K. Carotenoid pigments: their possible role in protecting against photooxidation in eyes and photoreceptor cells. *Proc R Soc Lond B Biol Sci* 1982;216:71-85.
41 Britton G. Structure and properties of carotenoids in relation to function. *Faseb J* 1995;9:1551-8.
42 Schmitz HH, Poor CL, Gugger ET, Erdman JW Jr. Analysis of carotenoids in human and animal tissues. *Methods Enzymol* 1993;214:102-16.
43 Evans JR. Antioxidant vitamin and mineral supplements for slowing the progression of age-related macular degeneration. *Cochrane Database Syst Rev* 2006;(4):CD000254.
44 Moher D, Cook DJ, Eastwood S, Olkin I, Rennie D, Stroup DF. Improving the quality of reports of meta-analyses of randomised controlled trials: the QUOROM statement. Quality of Reporting of Meta-analyses. *Lancet* 1999;354:1896-900.
45 Egger M, Zellweger-Zahner T, Schneider M, Junker C, Lengeler C, Antes G. Language bias in randomised controlled trials published in English and German. *Lancet* 1997;350:326-9.
46 Moher D, Fortin P, Jadad AR, Juni P, Klassen T, Le Lorier J, et al. Completeness of reporting of trials published in languages other than English: implications for conduct and reporting of systematic reviews. *Lancet* 1996;347:363-6.
47 Dickersin K. Systematic reviews in epidemiology: why are we so far behind? *Int J Epidemiol* 2002;31:6-12.
48 Egger M, Schneider M, Davey Smith G. Spurious precision? Meta-analysis of observational studies. *BMJ* 1998;316:140-4.

Accepted: 30 July 2007

BMJ: first published as 10.1136/bmj.39350.500428.47 on 8 October 2007. Downloaded from http://www.bmj.com/ on 17 July 2023 by guest. Protected by copyright.
