# Supplementary material for: Insights into the nutritional prevention of macular degeneration based on a comparative topic modeling approach
Source: PeerJ Comput Sci. 2024 Mar 20;10:e1940. doi: 10.7717/peerj-cs.1940 (PMC11042009; doi:10.7717/peerj-cs.1940)
Supplement: Supplemental Information 3 [file peerj-cs-10-1940-s003.zip › negative_result_reports/Evans_et_al-2017-Cochrane_Database_of_Systematic_Reviews.pdf]

Cochrane Library

Cochrane Database of Systematic Reviews

Antioxidant vitamin and mineral supplements for preventing age-related macular degeneration (Review)

Evans JR, Lawrenson JG

Evans JR, Lawrenson JG.
Antioxidant vitamin and mineral supplements for preventing age-related macular degeneration.
Cochrane Database of Systematic Reviews 2017, Issue 7. Art. No.: CD000253.
DOI: 10.1002/14651858.CD000253.pub4.

www.cochranelibrary.com

Copyright © 2017 The Cochrane Collaboration. Published by John Wiley & Sons, Ltd.
WILEY

TABLE OF CONTENTS

HEADER 1
ABSTRACT 1
PLAIN LANGUAGE SUMMARY 2
SUMMARY OF FINDINGS 4
BACKGROUND 10
OBJECTIVES 10
METHODS 10
RESULTS 12
- Figure 1. 13
- Figure 2. 14
- Figure 3. 15

DISCUSSION 17
AUTHORS' CONCLUSIONS 17
ACKNOWLEDGEMENTS 18
REFERENCES 19
CHARACTERISTICS OF STUDIES 28
DATA AND ANALYSES 41
- Analysis 1.1. Comparison 1 Vitamin E versus placebo, Outcome 1 Any AMD. 41
- Analysis 1.2. Comparison 1 Vitamin E versus placebo, Outcome 2 Late AMD (either neovascular AMD or geographic atrophy or both). 42
- Analysis 1.3. Comparison 1 Vitamin E versus placebo, Outcome 3 Neovascular AMD or geographic atrophy separately. 42
- Analysis 2.1. Comparison 2 Beta-carotene versus placebo, Outcome 1 Any AMD. 43
- Analysis 2.2. Comparison 2 Beta-carotene versus placebo, Outcome 2 Late AMD (either neovascular AMD or geographic atrophy or both). 43
- Analysis 2.3. Comparison 2 Beta-carotene versus placebo, Outcome 3 Neovascular AMD or geographic atrophy separately. 43
- Analysis 3.1. Comparison 3 Vitamin C versus placebo, Outcome 1 AMD. 44
- Analysis 4.1. Comparison 4 Multivitamin versus placebo, Outcome 1 AMD. 44

ADDITIONAL TABLES 44
APPENDICES 45
WHAT'S NEW 50
HISTORY 50
CONTRIBUTIONS OF AUTHORS 50
DECLARATIONS OF INTEREST 50
SOURCES OF SUPPORT 51
DIFFERENCES BETWEEN PROTOCOL AND REVIEW 51
INDEX TERMS 51

Copyright © 2017 The Cochrane Collaboration. Published by John Wiley & Sons, Ltd.

[Intervention Review]

Antioxidant vitamin and mineral supplements for preventing age-related macular degeneration

Jennifer R Evans[1], John G Lawrenson[2]

[1]Cochrane Eyes and Vision, ICEH, London School of Hygiene & Tropical Medicine, London, UK. [2]Centre for Applied Vision Research, School of Health Sciences, City University of London, London, UK

Contact address: Jennifer R Evans, Cochrane Eyes and Vision, ICEH, London School of Hygiene & Tropical Medicine, Keppel Street, London, WC1E 7HT, UK. jennifer.evans@lshtm.ac.uk.

Editorial group: Cochrane Eyes and Vision Group.
Publication status and date: Edited (no change to conclusions), published in Issue 7, 2017.

Citation: Evans JR, Lawrenson JG. Antioxidant vitamin and mineral supplements for preventing age-related macular degeneration. *Cochrane Database of Systematic Reviews* 2017, Issue 7. Art. No.: CD000253. DOI: 10.1002/14651858.CD000253.pub4.

Copyright © 2017 The Cochrane Collaboration. Published by John Wiley & Sons, Ltd.

ABSTRACT

Background

There is inconclusive evidence from observational studies to suggest that people who eat a diet rich in antioxidant vitamins (carotenoids, vitamins C, and E) or minerals (selenium and zinc) may be less likely to develop age-related macular degeneration (AMD).

Objectives

To determine whether or not taking antioxidant vitamin or mineral supplements, or both, prevent the development of AMD.

Search methods

We searched the Cochrane Central Register of Controlled Trials (CENTRAL) (which contains the Cochrane Eyes and Vision Trials Register) (2017, Issue 2), MEDLINE Ovid (1946 to 29 March 2017), Embase Ovid (1947 to 29 March 2017), AMED (Allied and Complementary Medicine Database) (1985 to 29 March 2017), OpenGrey (System for Information on Grey Literature in Europe) (www.opengrey.eu/); searched 29 March 2017, the ISRCTN registry (www.isrctn.com/editAdvancedSearch); searched 29 March 2017, ClinicalTrials.gov (www.clinicaltrials.gov); searched 29 March 2017 and the WHO International Clinical Trials Registry Platform (ICTRP) (www.who.int/ictrp/search/en); searched 29 March 2017. We did not use any date or language restrictions in the electronic searches for trials.

Selection criteria

We included all randomised controlled trials (RCTs) comparing an antioxidant vitamin or mineral supplement (alone or in combination) to control.

Data collection and analysis

Both review authors independently assessed risk of bias in the included studies and extracted data. One author entered data into RevMan 5; the other author checked the data entry. We pooled data using a fixed-effect model. We graded the certainty of the evidence using GRADE.

Main results

We included a total of five RCTs in this review with data available for 76,756 people. The trials were conducted in Australia, Finland, and the USA, and investigated vitamin C, vitamin E, beta-carotene, and multivitamin supplements. All trials were judged to be at low risk of bias.

Four studies reported the comparison of vitamin E with placebo. Average treatment and follow-up duration ranged from 4 to 10 years. Data were available for a total of 55,614 participants. There was evidence that vitamin E supplements do not prevent the development of any AMD (risk ratio (RR) 0.97, 95% confidence interval (CI) 0.90 to 1.06; high-certainty evidence), and may slightly increase the risk of

Copyright © 2017 The Cochrane Collaboration. Published by John Wiley & Sons, Ltd.

late AMD (RR 1.22, 95% CI 0.89 to 1.67; moderate-certainty evidence) compared with placebo. Only one study (941 participants) reported data separately for neovascular AMD and geographic atrophy. There were 10 cases of neovascular AMD (RR 3.62, 95% CI 0.77 to 16.95; very low-certainty evidence), and four cases of geographic atrophy (RR 2.71, 95% CI 0.28 to 26.0; very low-certainty evidence). Two trials reported similar numbers of adverse events in the vitamin E and placebo groups. Another trial reported excess of haemorrhagic strokes in the vitamin E group (39 versus 23 events, hazard ratio 1.74, 95% CI 1.04 to 2.91, low-certainty evidence).

Two studies reported the comparison of beta-carotene with placebo. These studies took place in Finland and the USA. Both trials enrolled men only. Average treatment and follow-up duration was 6 years and 12 years. Data were available for a total of 22,083 participants. There was evidence that beta-carotene supplements did not prevent any AMD (RR 1.00, 95% CI 0.88 to 1.14; high-certainty evidence) nor have an important effect on late AMD (RR 0.90, 95% CI 0.65 to 1.24; moderate-certainty evidence). Only one study (941 participants) reported data separately for neovascular AMD and geographic atrophy. There were 10 cases of neovascular AMD (RR 0.61, 95% CI 0.17 to 2.15; very low-certainty evidence) and 4 cases of geographic atrophy (RR 0.31 95% CI 0.03 to 2.93; very low-certainty evidence). Beta-carotene was associated with increased risk of lung cancer in people who smoked.

One study reported the comparison of vitamin C with placebo, and multivitamin (Centrum Silver) versus placebo. This was a study in men in the USA with average treatment duration and follow-up of 8 years for vitamin C and 11 years for multivitamin. Data were available for a total of 14,236 participants. AMD was assessed by self-report followed by medical record review. There was evidence that vitamin C supplementation did not prevent any AMD (RR 0.96, 95% CI 0.79 to 1.18; high-certainty evidence) or late AMD (RR 0.94, 0.61 to 1.46; moderate-certainty evidence). There was a slight increased risk of any AMD (RR 1.21, 95% CI 1.02 to 1.43; moderate-certainty evidence) and late AMD (RR 1.22, 95% CI 0.88 to 1.69; moderate-certainty evidence) in the multivitamin group. Neovascular AMD and geographic atrophy were not reported separately. Adverse effects were not reported but there was possible increased risk of skin rashes in the multivitamin group.

Adverse effects were not consistently reported in these eye studies, but there is evidence from other large studies that beta-carotene increases the risk of lung cancer in people who smoke or who have been exposed to asbestos.

None of the studies reported quality of life or resource use and costs.

Authors' conclusions

Taking vitamin E or beta-carotene supplements will not prevent or delay the onset of AMD. The same probably applies to vitamin C and the multivitamin (Centrum Silver) investigated in the one trial reported to date. There is no evidence with respect to other antioxidant supplements, such as lutein and zeaxanthin. Although generally regarded as safe, vitamin supplements may have harmful effects, and clear evidence of benefit is needed before they can be recommended. People with AMD should see the related Cochrane Review on antioxidant vitamin and mineral supplements for slowing the progression of AMD, written by the same review team.

PLAIN LANGUAGE SUMMARY

Antioxidant vitamin and mineral supplements to prevent the development of age-related macular degeneration (AMD)

What is the aim of this review?
The aim of this Cochrane Review was to find out whether taking antioxidant vitamin and mineral supplements prevents the development of AMD. Cochrane researchers collected and analysed all relevant studies to answer this question and found five studies.

Key messages
Taking vitamin E or beta-carotene supplements will not prevent the onset of AMD in people who do not have signs of the condition. The same probably applies to vitamin C and multivitamin tablets. There is no evidence for other supplements, such as lutein and zeaxanthin.

What was studied in the review?
AMD is a condition of the central area (macula) of the back of the eye (retina). The macula degenerates with age. In some people, this deterioration happens more quickly, and is associated with a particular appearance at the back of the eye. In its earliest stage (early AMD), yellow spots (drusen) can be seen under the retina by an eye health professional on examining the eye. The affected person will probably be unaware that they have a problem. As AMD progresses, it can lead to the loss of the cells in the back of the eye, which are needed for vision. This is known as geographic atrophy. Sometimes, new (harmful) blood vessels grow in the macula. These new blood vessels may bleed and cause scarring. This is known as neovascular or wet AMD. Any damage to the macula can affect vision, particularly central vision. Neovascular AMD and geographic atrophy are known as late AMD.

It is possible that antioxidant vitamins may help to protect the macula against this deterioration and loss of vision. Vitamin C, E, beta-carotene, lutein, zeaxanthin, and zinc are examples of antioxidant vitamins commonly found in vitamin supplements.

The Cochrane researchers only looked at the effects of these supplements in healthy people in the general population who did not yet have AMD. There is another Cochrane Review on the effects of these supplements in people who already have AMD.

What are the main results of the review?

Copyright © 2017 The Cochrane Collaboration. Published by John Wiley & Sons, Ltd.

The Cochrane researchers found five relevant studies. The studies were large and included a total of 76,756 people. They took place in Australia, Finland, and the USA. The studies compared vitamin C, vitamin E, beta-carotene, and multivitamin supplements with placebo.

The review showed that, compared with taking a placebo:

· Taking vitamin E supplements made little or no difference to the chances of developing AMD (high-certainty evidence).
· Taking vitamin E supplements made little difference, or slightly increased, the chances of developing late AMD (moderate-certainty evidence).
· Taking beta-carotene made little or no difference to the chances of developing any AMD (high-certainty evidence) or late AMD (moderate-certainty evidence).
· Taking vitamin C made little or no difference to the chances of developing any AMD (high-certainty evidence) or late AMD (moderate-certainty evidence).
· Taking multivitamin tablets may slightly increase the chances of developing any AMD or late AMD (moderate-certainty evidence).
· Adverse effects were not consistently reported in these eye studies, but there is evidence from other large studies that beta-carotene increases the risk of lung cancer in people who smoke, or who have been exposed to asbestos.

None of the studies reported quality of life or resource use and costs.

How up-to-date is this review?
The Cochrane researchers searched for studies that had been published up to 29 March 2017.

Copyright © 2017 The Cochrane Collaboration. Published by John Wiley & Sons, Ltd.

SUMMARY OF FINDINGS

Summary of findings for the main comparison. Vitamin E versus placebo

Vitamin E versus placebo

Patient or population: general population
Setting: community
Intervention: vitamin E*
Comparison: placebo

Outcomes	Anticipated absolute effects (95% CI)		Relative effect (95% CI)	№ of participants (studies)	Certainty of the evidence (GRADE)	Comments
	Risk with placebo**	Risk with vitamin E				
Any AMD	150 per 1000	146 per 1000 (135 to 159)	RR 0.97 (0.90 to 1.06)	55,614 (4 RCTs)	⊕⊕⊕⊕ HIGH	Average duration of treatment and follow-up ranged from 4 years to 10 years
Late AMD (either neovascular AMD or geographic atrophy or both)	5 per 1000	6 per 1000 (4 to 8)	RR 1.22 (0.89 to 1.67)	55,614 (4 RCTs)	⊕⊕⊕⊝ MODERATE [1]	Average duration of treatment and follow-up ranged from 4 years to 10 years
Neovascular AMD	3 per 1000	11 per 1000 (2 to 51)	RR 3.62 (0.77 to 16.95)	941 (1 RCT)	⊕⊝⊝⊝ VERY LOW [2]	Average duration of treatment and follow-up was 6 years
Geographic atrophy	2 per 1000	6 per 1000 (1 to 57)	RR 2.71 (0.28 to 26.00)	941 (1 RCT)	⊕⊝⊝⊝ VERY LOW [2]	Average duration of treatment and follow-up was 6 years
Quality of life	-	-	-	-	-	Not reported
Adverse effects (AE)	-	-	-	-	⊕⊕⊝⊝ LOW[3]	Two trials reported similar numbers of AEs in vitamin E and placebo group. Another trial reported excess of haemorrhagic strokes in vitamin E group (39 vs 23 events, hazard ratio 1.74, 95% CI 1.04 to 2.91).
Resource use and costs	-	-	-	-	-	Not reported

* Dose of vitamin E used in studies were: 50 mg/day, 400 IU/alternate days, 600 IU/alternate days, and 500 IU/day

Copyright © 2017 The Cochrane Collaboration. Published by John Wiley & Sons, Ltd.

The risk in the placebo group is the median risk in the placebo groups in the included studies. **The risk in the intervention group** (and its 95% confidence interval) is based on the assumed risk in the comparison group and the **relative effect** of the intervention (and its 95% CI).
CI: Confidence interval; **RR:** Risk ratio

GRADE Working Group grades of evidence
High-certainty: We are very confident that the true effect lies close to that of the estimate of the effect
Moderate-certainty: We are moderately confident in the effect estimate: The true effect is likely to be close to the estimate of the effect, but there is a possibility that it is substantially different
Low-certainty: Our confidence in the effect estimate is limited: The true effect may be substantially different from the estimate of the effect
Very low-certainty: We have very little confidence in the effect estimate: The true effect is likely to be substantially different from the estimate of effect

[1] Downgraded one level for imprecision due to wide confidence intervals i.e. are below 0.8 or above 1.25.
[2] Downgraded one level for indirectness (only one trial in male smokers) and downgraded two levels for imprecision as very few cases (10 neovascular AMD, 4 geographic atrophy)
3 Downgraded one level for imprecision due to wide confidence intervals and lower confidence near 1 and downgraded one level for inconsistency as effect only reported by one trial.

Summary of findings 2. Beta-carotene versus placebo

Beta-carotene versus placebo

Patient or population: general population
Setting: community
Intervention: beta-carotene*
Comparison: placebo

Outcomes	Anticipated absolute effects (95% CI)		Relative effect (95% CI)	№ of participants (studies)	Certainty of the evidence (GRADE)	Comments
	Risk with placebo**	Risk with beta-carotene				
Any AMD	150 per 1000	150 per 1000 (132 to 171)	RR 1.00 (0.88 to 1.14)	22,083 (2 RCTs)	⊕⊕⊕⊕ HIGH	Average duration of treatment and follow-up was 6 years in one study and 12 years in the other study
Late AMD (either neovascular AMD or geographic atrophy or both)	5 per 1000	5 per 1000 (3 to 6)	RR 0.90 (0.65 to 1.24)	22,083 (2 RCTs)	⊕⊕⊕⊝ MODERATE [1]	Average duration of treatment and follow-up was 6 years in one study and 12 years in the other study
Neovascular AMD	3 per 1000	2 per 1000 (1 to 6)	RR 0.61 (0.17 to 2.15)	941 (1 RCT)	⊕⊝⊝⊝ VERY LOW [2]	Average duration of treatment and follow-up was 6 years

Copyright © 2017 The Cochrane Collaboration. Published by John Wiley & Sons, Ltd.

Geographic atrophy	2 per 1000	1 per 1000 (0 to 6)	RR 0.31 (0.03 to 2.93)	941 (1 RCT)	⊕⊝⊝⊝ VERY LOW [2]	Average duration of treatment and follow-up was 6 years
Quality of life	-	-	-	-	-	Not reported
Adverse effects	-		-	-	⊕⊕⊕⊕ HIGH	Beta-carotene associated with increased risk of lung cancer in people who smoke.
Resource use and costs	-	-	-	-	-	Not reported

* Dose of beta-carotene used was 20 mg/day in one study and 50 mg/alternate days in the other study.

****The risk in the placebo group** is the median risk in the control groups of the four included studies in Summary of findings for the main comparison. **The risk in the intervention group** (and its 95% confidence interval) is based on the assumed risk in the comparison group and the **relative effect** of the intervention (and its 95% CI).

CI: Confidence interval; **RR:** Risk ratio

GRADE Working Group grades of evidence
High-certainty: We are very confident that the true effect lies close to that of the estimate of the effect
Moderate-certainty: We are moderately confident in the effect estimate: The true effect is likely to be close to the estimate of the effect, but there is a possibility that it is substantially different
Low-certainty: Our confidence in the effect estimate is limited: The true effect may be substantially different from the estimate of the effect
Very low-certainty: We have very little confidence in the effect estimate: The true effect is likely to be substantially different from the estimate of effect

[1] Downgraded one level for imprecision due to wide confidence intervals i.e. are below 0.8 or above 1.25.
[2] Downgraded one level for indirectness (only one trial in male smokers) and downgraded two levels for imprecision as very few cases (10 neovascular AMD, 4 geographic atrophy)

Summary of findings 3. Vitamin C versus placebo

Vitamin C versus placebo

Patient or population: general population
Setting: community
Intervention: vitamin C*
Comparison: placebo

Outcomes	Anticipated absolute effects (95% CI)		Relative effect (95% CI)	№ of participants (studies)	Certainty of the evidence (GRADE)	Comments
	Risk with placebo**	Risk with vitamin C				

Copyright © 2017 The Cochrane Collaboration. Published by John Wiley & Sons, Ltd.

Any AMD	150 per 1000	144 per 1000 (119 to 177)	RR 0.96 (0.79 to 1.18)	14,236 (1 RCT)	⊕⊕⊕⊕ HIGH	Average duration of treatment and follow-up was 8 years
Late AMD (either neovascular AMD or geographic atrophy or both)	5 per 1000	5 per 1000 (3 to 7)	RR 0.94 (0.61 to 1.46)	14,236 (1 RCT)	⊕⊕⊕⊝ MODERATE [1]	Average duration of treatment and follow-up was 8 years
Neovascular AMD	-	-	-	-	-	Not reported
Geographic atrophy	-	-	-	-	-	Not reported
Quality of life	-	-	-	-	-	Not reported
Adverse effects	-		-	-	-	None reported
Resource use and costs	-	-	-	-	-	Not reported

* Dose of vitamin C used was 500 mg/day.

****The risk in the placebo group** is the median risk in the control groups of the four included studies in Summary of findings for the main comparison. **The risk in the intervention group** (and its 95% confidence interval) is based on the assumed risk in the comparison group and the **relative effect** of the intervention (and its 95% CI).

CI: Confidence interval; **RR:** Risk ratio

GRADE Working Group grades of evidence
High-certainty: We are very confident that the true effect lies close to that of the estimate of the effect
Moderate-certainty: We are moderately confident in the effect estimate: The true effect is likely to be close to the estimate of the effect, but there is a possibility that it is substantially different
Low-certainty: Our confidence in the effect estimate is limited: The true effect may be substantially different from the estimate of the effect
Very low-certainty: We have very little confidence in the effect estimate: The true effect is likely to be substantially different from the estimate of effect

[1] Downgraded one level for imprecision due to wide confidence intervals i.e. are below 0.8 or above 1.25.

Summary of findings 4. Multivitamin versus placebo

Multivitamin versus placebo for preventing AMD

Patient or population: general population
Setting: community
Intervention: multivitamin*
Comparison: placebo

Copyright © 2017 The Cochrane Collaboration. Published by John Wiley & Sons, Ltd.

Outcomes	Anticipated absolute effects (95% CI)		Relative effect (95% CI)	№ of participants (studies)	Certainty of the evidence (GRADE)	Comments
	Risk with placebo**	Risk with multivitamin				
Any AMD	150 per 1000	182 per 1000 (153 to 215)	RR 1.21 (1.02 to 1.43)	14,233 (1 RCT)	⊕⊕⊕⊝ MODERATE [1]	Average duration of treatment and follow-up was 11 years
Late AMD	5 per 1000	6 per 1000 (4 to 8)	RR 1.22 (0.88 to 1.69)	14,233 (1 RCT)	⊕⊕⊕⊝ MODERATE [1]	Average duration of treatment and follow-up was 11 years
Neovascular AMD	-	-	-	-	-	Not reported
Geographic atrophy	-	-	-	-	-	Not reported
Quality of life	-	-	-	-	-	Not reported
Adverse effects	-	-	-	-	⊕⊕⊕⊝ MODERATE [1]	"Those taking the active versus placebo multivitamin were more likely to have skin rashes (2111 and 1973 men in corresponding active and placebo multivitamin groups; HR 1.08, 95% CI 1.01 to 1.15; P = 0.016)". PHS II
Resource use and costs	-	-	-	-	-	Not reported

* Multivitamin used was Centrum Silver (zinc 15 mg, vitamin E 45 IU, vitamin C 60 mg, beta-carotene 5000 IU vitamin A, 20% as beta carotene, folic acid 2.5 mg, vitamin B6 50 mg, vitamin B12 1 mg)

****The risk in the placebo group** is the median risk in the control groups of the four included studies in Summary of findings for the main comparison. **The risk in the intervention group** (and its 95% confidence interval) is based on the assumed risk in the comparison group and the **relative effect** of the intervention (and its 95% CI).

CI: Confidence interval; **RR:** Risk ratio

GRADE Working Group grades of evidence
High-certainty: We are very confident that the true effect lies close to that of the estimate of the effect
Moderate-certainty: We are moderately confident in the effect estimate: The true effect is likely to be close to the estimate of the effect, but there is a possibility that it is substantially different
Low-certainty: Our confidence in the effect estimate is limited: The true effect may be substantially different from the estimate of the effect
Very low-certainty: We have very little confidence in the effect estimate: The true effect is likely to be substantially different from the estimate of effect

[1] Downgraded one level for imprecision

Copyright © 2017 The Cochrane Collaboration. Published by John Wiley & Sons, Ltd.

Copyright © 2017 The Cochrane Collaboration. Published by John Wiley & Sons, Ltd.

BACKGROUND

Description of the condition

Age-related macular degeneration (AMD) is a disease affecting the central area of the retina (macula). In the early stages of the disease, lipid material accumulates in deposits underneath the retinal pigment epithelium. These deposits are known as drusen, and can be seen as pale yellow spots on the retina. The pigment of the retinal pigment epithelium may become disturbed, with areas of hyperpigmentation and hypopigmentation. In the later stages of the disease, the retinal pigment epithelium may atrophy completely. This loss can occur in small focal areas or be widespread (geographic). In some cases, new blood vessels grow under the retinal pigment epithelium, and occasionally, into the subretinal space (exudative or neovascular AMD). Haemorrhage can occur, which often results in increased scarring of the retina.

The early stages of the disease are, in general, asymptomatic. In the later stages, there may be considerable distortion within the central visual field, leading to a complete loss of central visual function.

Population-based studies suggest that, in people 65 years and older, approximately 5% have advanced AMD (Owen 2012). It is the most common cause of blindness and visual impairment in industrialised countries. In the UK, for example, over 30,000 people are registered as blind or partially sighted annually, half of whom have lost their vision due to macular degeneration (Bunce 2006).

Description of the intervention

Photoreceptors in the retina are subject to oxidative stress throughout life, due to combined exposures to light and oxygen. It has been proposed that antioxidants may prevent cellular damage in the retina by reacting with free radicals produced in the process of light absorption (Christen 1996).

There are a number of non-experimental studies that have examined the possible association between antioxidant micronutrients and AMD, although few studies have examined supplementation specifically (Chong 2007; Evans 2001). Data on vitamin intake in observational studies should be considered cautiously, as people who have a diet rich in antioxidant vitamins and minerals, or who choose to take supplements regularly, are different in many ways from those who do not; these differences may not be adequately controlled by statistical analysis. The results of these observational studies have been inconclusive.

How the intervention might work

The underlying theory is that antioxidant vitamin and mineral supplements will protect the retina against oxidative stress, and that this protection will delay the onset of AMD.

Why it is important to do this review

Antioxidant vitamin and mineral supplements are increasingly being marketed for use in age-related eye disease, including AMD. The aim of this review was to examine the evidence on whether taking vitamin or mineral supplements prevents the development of AMD. See also the related Cochrane Review on antioxidant vitamin and mineral supplements for slowing the progression of AMD, which considered whether supplementation for people with AMD slowed down the progression of the disease (Evans 2017).

OBJECTIVES

To determine whether or not taking antioxidant vitamin or mineral supplements, or both, prevent the development of AMD.

METHODS

Criteria for considering studies for this review

Types of studies

We included randomised controlled trials (RCTs) that compared antioxidant vitamin or mineral supplements, (alone or in combination) with control (placebo or no treatment).

Types of participants

Participants in the trials were people in the general population, with or without diseases other than AMD. We excluded trials in which the participants were exclusively people with AMD. These trials were considered in a separate Cochrane Review that examined the effect of supplementation on progression of the disease (Evans 2017).

Types of interventions

We defined antioxidants as any vitamin or mineral that was known to have antioxidant properties in vivo or which was known to be an important component of an antioxidant enzyme present in the retina. We considered the following: vitamin C, vitamin E, carotenoids (including the macula pigment carotenoids lutein and zeaxanthin), selenium and zinc.

Types of outcome measures

We modified our protocol for the current update (2017) to include outcomes specified by the UK NICE macular degeneration guideline panel (NICE 2016) - see Differences between protocol and review.

We considered the following outcomes:

- Development of:
  - any AMD (early or late AMD, or both)
  - late AMD (neovascular AMD or geographic atrophy, or both)
  - neovascular AMD
  - geographic atrophy
- Quality of life
- Resource use and costs

Follow-up:

We considered the maximum follow-up identified in the studies.

Adverse effects

We considered any adverse effects reported by the included studies.

Search methods for identification of studies

Electronic searches

The Cochrane Eyes and Vision Information Specialist conducted systematic searches in the following databases for randomised controlled trials and controlled clinical trials. There were no language or publication year restrictions. The date of the search was 29 March 2017.

Copyright © 2017 The Cochrane Collaboration. Published by John Wiley & Sons, Ltd.

- Cochrane Central Register of Controlled Trials (CENTRAL; 2017, Issue 2) (which contains the Cochrane Eyes and Vision Trials Register) in the Cochrane Library (searched 29 March 2017) (Appendix 1);
- MEDLINE Ovid (1946 to 29 March 2017) (Appendix 2);
- Embase Ovid (1980 to 29 March 2017) (Appendix 3);
- AMED (Allied and Complementary Medicine Database) (1985 to 29 March 2017) (Appendix 4);
- OpenGrey (System for Information on Grey Literature in Europe) (www.opengrey.eu/; searched 29 March 2017) (Appendix 5);
- ISRCTN registry (www.isrctn.com/editAdvancedSearch; searched 29 March 2017) (Appendix 6);
- US National Institutes of Health Ongoing Trials Register ClinicalTrials.gov (www.clinicaltrials.gov; searched 29 March 2017) (Appendix 7);
- World Health Organization International Clinical Trials Registry Platform (www.who.int/ictrp; searched 29 March 2017) (Appendix 8).

For the 2012 and 2017 updates, we specifically looked for adverse effects, using a simple search aimed to identify systematic reviews of adverse effects of vitamin supplements, see Appendix 9 for search strategy.

Searching other resources

We searched the Science Citation Index and the reference lists of reports of trials that were selected for inclusion. We contacted the investigators of included and excluded trials to ask if they knew of any other relevant published or unpublished trials.

Data collection and analysis

Selection of studies

Our initial searches identified all trials of antioxidant supplements, and therefore, generated many citations. Each review author independently assessed half of the titles and abstracts resulting from the searches, and selected studies according to the definitions in the Criteria for considering studies for this review. To check that we were consistent, we both assessed a subset of 100 records and compared results. We obtained full copies of all reports referring to controlled trials that definitely or potentially met the inclusion criteria. We assessed the full copies and selected studies according to the inclusion criteria. We wrote to authors of trials for which there were no published outcome data on AMD, to ask whether they had collected any data on eye disease outcomes.

As none of the trial authors responded positively, i.e. gave us unpublished data on AMD, for further updates of this review we only considered trials with published data on AMD.

In updates to this review, both authors independently went through the titles and abstracts resulting from the searches, and resolved disagreements by discussion.

Data extraction and management

We extracted data using methods' forms developed by Cochrane Eyes and Vision (eyes.cochrane.org/resources-review-authors). We independently extracted data, and resolved disagreements by discussion. One author cut and pasted the data into Review Manager 5 (Review Manager 5 2014), and the other author checked that this had been done correctly.

For the 2017 update, we screened and extracted data using web-based review management software (Covidence 2015).

Assessment of risk of bias in included studies

Both authors independently assessed risk of bias using the Cochrane tool for assessing risk of bias, as described in Chapter 8 of the *Cochrane Handbook for Systematic Reviews of Interventions* (Higgins 2011). We resolved disagreements by discussion. The review authors were not masked to any trial details.

Measures of treatment effect

Our measure of treatment effect was the risk ratio (RR) for dichotomous outcomes and the mean difference (MD) for continuous outcomes. Currently the review only includes analysis of dichotomous outcomes.

Unit of analysis issues

By definition, the interventions were applied to the person, but as most people have two eyes, trials can analyse data from one or both eyes. In all the trials included in this review the unit of analysis was the person and severity was classified for the worse eye.

Assessment of heterogeneity

We assessed heterogeneity by visual inspection of the forest plot, the Chi^2 test for heterogeneity and the I^2 statistic.

Data synthesis

Where appropriate, we pooled data using a fixed-effect model, after testing for heterogeneity between trial results using a standard Chi^2 test.

Subgroup analysis and investigation of heterogeneity

We did not plan any subgroup analyses. Only five trials are included at present, which means that it is not possible to formally investigate heterogeneity.

Sensitivity analysis

We had planned to conduct sensitivity analyses to determine the impact of study quality on effect size. Currently there are only high-quality trials included in the review, and therefore, this is not relevant at present.

'Summary of findings' tables

We prepared separate 'Summary of findings' tables for the different types of vitamin supplement, i.e. vitamin C, vitamin E, beta-carotene, and multivitamin.

We assessed the certainty of the evidence (GRADE) for each outcome using customised software (GRADEpro 2014). JE did the initial assessment, which was checked by JL. We considered risk of bias, inconsistency, indirectness, imprecision, and publication bias when judging the certainty of the evidence (Schünemann 2011).

The 'Summary of findings' tables include an estimate of the risk of each outcome in the general population. We derived these from the median risk in the placebo group in four of the studies included in the review (ATBC 1998; PHS II 2012; VECAT 2002; WHS 2010). As

Copyright © 2017 The Cochrane Collaboration. Published by John Wiley & Sons, Ltd.

there was overlap in participants between PHS I 2007 and PHS II 2012, we just used data from PHS II 2012 for this estimate.

RESULTS

Description of studies

Results of the search

The initial searches resulted in 3178 titles and abstracts. Of these, 208 were potentially eligible trials reports. From these reports, we identified seven primary prevention trials of antioxidant vitamin or mineral supplements (ATBC 1994; CARET 1996; De Klerk 1998; LINXIAN 1993; Nambour 1995; PHS I 2007; WHS 2010). Investigators from three trials have confirmed that they did not collect data on AMD (CARET 1996; De Klerk 1998; Nambour 1995). We excluded these trials from the review. We did not receive a response from one trial author; we excluded this trial (LINXIAN 1993). Three trials had published data on AMD outcomes; we included them in this review (ATBC 1994; PHS I 2007; WHS 2010). Search of the National Eye Institute Clinical Research register identified one further ongoing trial that was collecting information on AMD - the Women's Antioxidant Cardiovascular Study (WACS). There are two trials that have recruited participants with and without AMD (AREDS 2001; VECAT 2002). We included VECAT 2002 in this review because 82% of participants did not have signs of AMD. We excluded AREDS 2001 because it did not include AMD outcomes for people without AMD at baseline; it is included in the Cochrane Review examining the effect of supplementation on progression of the disease (Evans 2017).

In our original search strategy, we identified all trials of antioxidant interventions and asked trialists if they had collected data on AMD. We wrote to the authors of 60 trials of antioxidant interventions in people with diseases other than AMD. We received 15 responses, and none had collected any relevant data. We included all 60 trials in the excluded studies section of this review. As this proved to be an inefficient way of identifying relevant trials, we included terms for AMD in subsequent searches. We found 367 reports of trials in May 2002, 343 in May 2005, and 64 reports in January 2006, but we did not identify any further trials that were relevant for this review. The results of the PHS I study were published in 2007.

We repeated the searches in August 2007, at which time we identified a total of 129 reports of studies. The Trial Search Co-ordinator (TSC) scanned the search results and removed 84 references that were not relevant to the scope of the review. We screened the titles and abstracts of the remaining 45 references and obtained full-text copies of four reports to assess for potential inclusion in the review. We identified one new report from the PHS I 2007 study to include in the review and excluded the three remaining studies. For reasons of exclusion, see the 'Characteristics of excluded studies' table.

An update search was done in January 2012 which yielded 477 titles and abstracts. The TSC scanned the search results and removed 206 references which were not relevant to the scope of the review. We screened the title and abstracts of the remaining 271 references. We rejected 267 abstracts as not eligible for inclusion in the review. We obtained full-text copies of four reports for further examination. One new report from the WHS 2010 study has been included in the review and three other studies were excluded. For reasons of exclusion, see Characteristics of excluded studies.

Update searches run in March 2017 yielded a further 872 records (Figure 1). After 157 duplicates were removed, the Cochrane Information Specialist (CIS; formerly the Trial Search Co-ordinator) screened the remaining 715 records and removed 562 references that were not relevant to the scope of the review. We screened the remaining 153 references and obtained one full-text report for further assessment, however, this study did not meet the inclusion criteria. We checked the status of the ongoing studies published in the previous version of this review. PHS II 2012 was now completed and we included it in this update. AREDS2 2008 was also completed, however, this study did not meet the inclusion criteria; see Characteristics of excluded studies for details.

Copyright © 2017 The Cochrane Collaboration. Published by John Wiley & Sons, Ltd.

Figure 1. Study flow diagram.

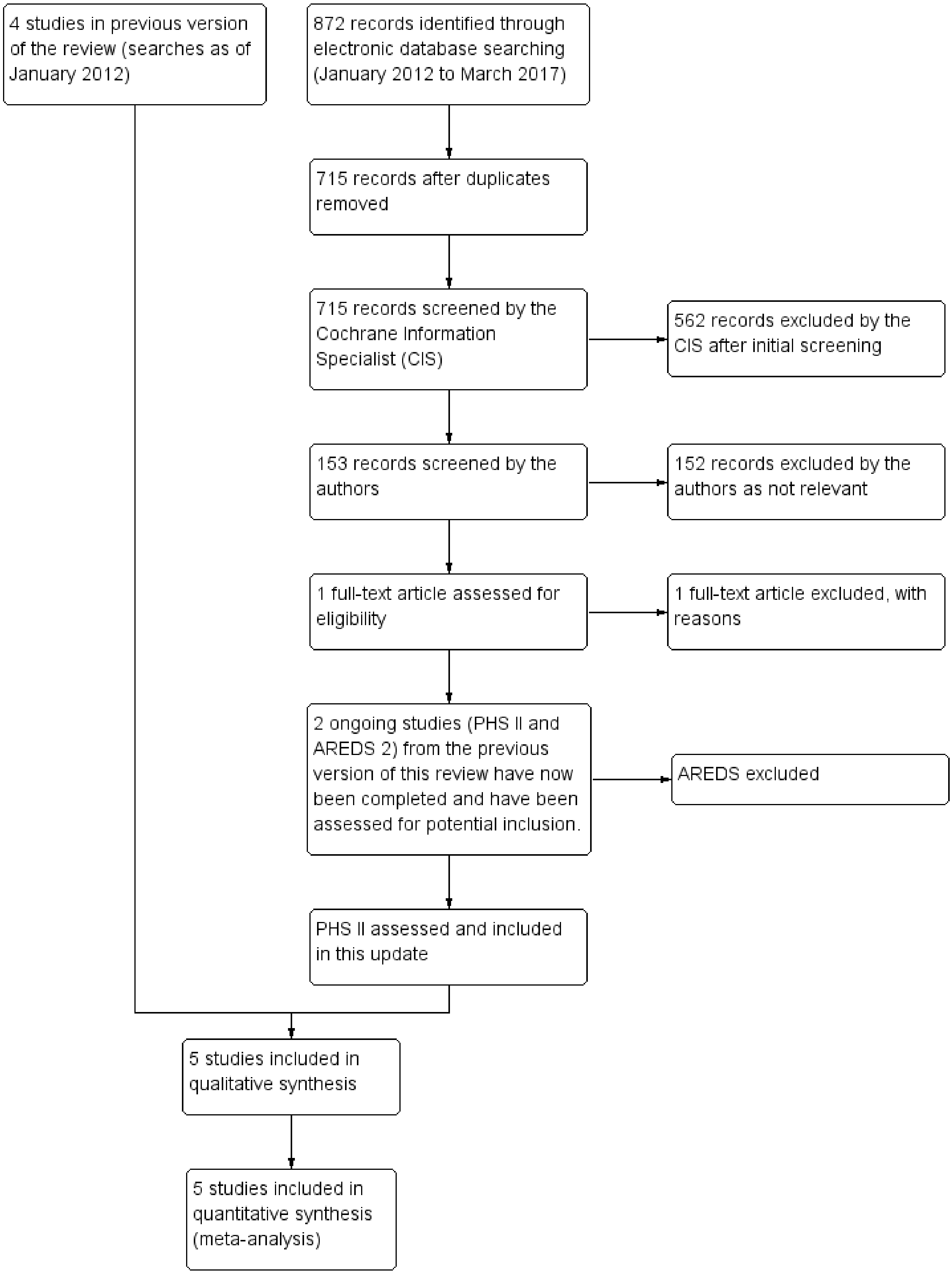

Copyright © 2017 The Cochrane Collaboration. Published by John Wiley & Sons, Ltd.

Included studies

See the 'Characteristics of included studies' table for more detailed information.

Types of participants

The studies took place in Australia, USA, and Finland. Three studies recruited men only (ATBC 1998; PHS I 2007; PHS II 2012), one study recruited women only (WHS 2010), and one study recruited both men and women (VECAT 2002). There was overlap of the participants in PHS I 2007 and PHS II 2012, as some participants in PHS II 2012 were recruited from PHS I 2007.

People taking part in the trials were identified from the general population. Participants in PHS I 2007 and PHS II 2012 were male physicians, and in WHS 2010 were female health professionals. In ATBC 1998, a random sample of 1035 men aged 65 years or older from the main study were invited to participate, with a response of 91% (941 men). In VECAT 2002, 18% of participants had AMD at baseline.

Types of intervention

In ATBC 1998, the groups received either alpha-tocopherol 50 mg per day alone, beta-carotene 20 mg per day alone, alpha-tocopherol and beta-carotene, or placebo. All formulations were coloured with quinoline yellow. Treatment duration was five to eight years (median 6.1 years). In VECAT 2002, participants were randomised to vitamin E (500 IU a day) or placebo. Supplementation continued for four years. In PHS I 2007, the groups received aspirin 325 mg every other day, beta-carotene 50 mg every other day, aspirin and beta-carotene, or placebo. Treatment duration averaged 12 years. In PHS II 2012, participants received vitamin C (500 mg daily), vitamin E (400 IU on alternate days), or daily multivitamin (Centrum Silver), or corresponding placebos. A beta-carotene arm was discontinued in 2003, and results not reported. In WHS 2010, participants received vitamin E (600 IU on alternate days) or placebo, and were followed up for 10 years.

Types of outcome measures

In ATBC 1998, three photographs of each eye were taken with a Canon fundus camera at 40- and 60-degree angles on Kodak Ektachrome 100 ASA slide film. These photographs were graded by one observer, masked to the participant's treatment group. The following grades of maculopathy were used: 0 = none; I = dry maculopathy with hard drusen, pigmentary changes, or both; II = soft macular drusen; III = disciform degeneration; IV = geographic atrophy.

In PHS I 2007, PHS II 2012, and WHS 2010, AMD was ascertained by self report: "Have you ever had macular degeneration diagnosed in your right or left eye?". If the participant answered yes to this question, permission was gained to contact their ophthalmologist or optometrist, and further details were obtained from the medical records.

In VECAT 2002, photographs were taken with a Nidek 3-DX fundus camera on Kodachrome 64 ASA colour film. The photographs were graded at baseline independently by two trained graders. Early AMD (the primary outcome) was defined as soft drusen (distinct or indistinct) or pigmentary changes (hyperpigmentation or hypopigmentation) on photographic grading. On clinical grading, this was large, soft drusen or non-geographical retinal pigment epithelium atrophy. VECAT 2002 used Bailey-Lovie visual acuity charts #4 and #5 (National Vision Research Institute, Australia).

Table 1 shows how the AMD outcome measures in the included studies were mapped onto the pre-specified review outcomes.

Excluded studies

See the 'Characteristics of excluded studies' table for further information.

Risk of bias in included studies

We considered all five trials to be at low risk of bias (Figure 2; Figure 3). See 'Risk of bias' tables for each included study for details of the assessment.

Figure 2. 'Risk of bias' graph: review authors' judgements about each risk of bias item presented as percentages across all included studies.

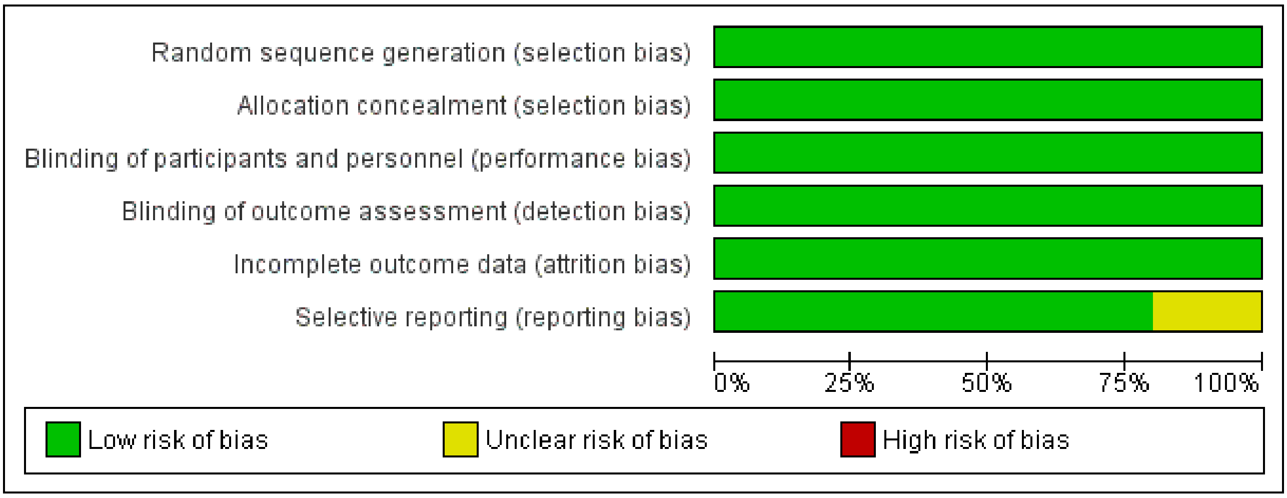

Copyright © 2017 The Cochrane Collaboration. Published by John Wiley & Sons, Ltd.

Figure 3. 'Risk of bias' summary: review authors' judgements about each risk of bias item for each included study.

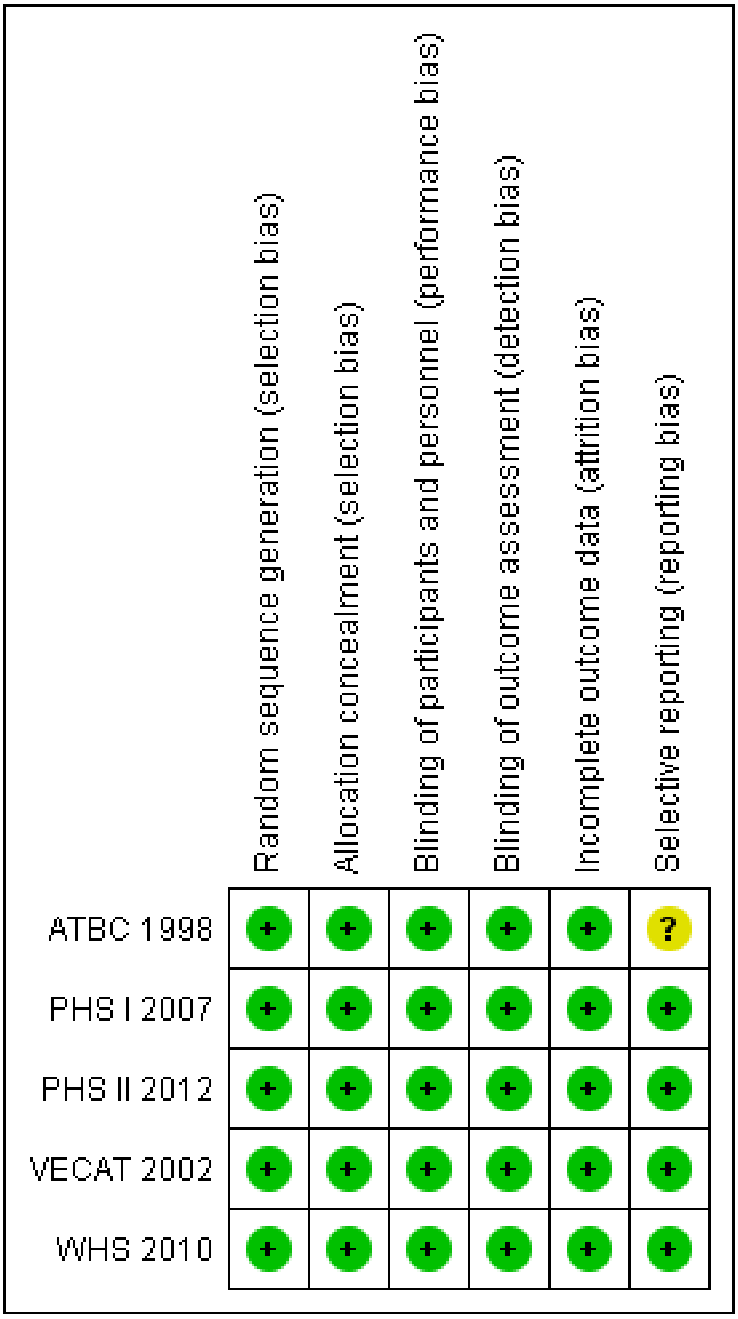

Effects of interventions

See: **Summary of findings for the main comparison** Vitamin E versus placebo; **Summary of findings 2** Beta-carotene versus placebo; **Summary of findings 3** Vitamin C versus placebo; **Summary of findings 4** Multivitamin versus placebo

See Table 1 for details of how the AMD outcomes reported in the trials map to the review outcomes reported here. None of the studies reported quality of life or resource use and costs. For details of the GRADE assessments, see the 'Summary of findings' tables (Summary of findings for the main comparison; Summary of findings 2; Summary of findings 3; Summary of findings 4).

Vitamin E versus placebo

Summary of findings for the main comparison

Four studies reported this comparison (ATBC 1998; PHS II 2012; VECAT 2002; WHS 2010). These studies took place in Australia (VECAT 2002), Finland (ATBC 1998), and USA (PHS II 2012; WHS 2010). Men (ATBC 1998; PHS II 2012), women (WHS 2010), or both (VECAT 2002) were enrolled. Average treatment and follow-up duration ranged from 4 years (VECAT 2002) to 10 years (WHS 2010). Data were available for a total of 55,614 participants.

Copyright © 2017 The Cochrane Collaboration. Published by John Wiley & Sons, Ltd.

The risk ratio for any AMD was 0.97 (95% CI 0.90 to 1.06; high-certainty evidence; Analysis 1.1) and for late AMD was 1.22 (95% CI 0.89 to 1.67; moderate-certainty evidence; Analysis 1.2).

Only one study (941 participants) reported data separately for neovascular AMD and geographic atrophy (ATBC 1998). There were 10 cases of neovascular AMD**(**RR 3.62, 9%% CI 0.77 to 16.95; very low-certainty evidence) and four cases of geographic atrophy (RR 2.71, 95% CI 0.28 to 26.0; very low-certainty evidence).

Beta-carotene versus placebo

Summary of findings 2

Two studies reported this comparison (ATBC 1998; PHS I 2007). These studies took place in Finland (ATBC 1998), and USA (PHS I 2007). Both trials enrolled men only. Average treatment and follow-up duration was six years (ATBC 1998), and 12 years (PHS I 2007). Data were available for a total of 22,083 participants.

The risk ratio for any AMD was 1.00 (95% CI 0.88 to 1.14; high-certainty evidence; Analysis 2.1), and 0.90 (95% CI 0.65 to 1.24; moderate-certainty evidence; Analysis 2.2) for late AMD.

Only one study (941 participants) reported data separately for neovascular AMD and geographic atrophy (ATBC 1998). There were 10 cases of neovascular AMD (RR 0.61, 95% CI 0.17 to 2.15; very low-certainty evidence) and four cases of geographic atrophy (RR 0.31 95% CI 0.03 to 2.93; very low-certainty evidence).

Vitamin C versus placebo

Summary of findings 3

One study reported this comparison (PHS II 2012). PHS II 2012 was a study on men in the USA, with average treatment duration and follow-up of eight years. Data were available for a total of 14,236 participants.

The risk ratio (RR) for any AMD was 0.96 (95% CI 0.79 to 1.18; high-certainty evidence) and for late AMD was 0.94 (0.61 to 1.46; moderate-certainty evidence; Analysis 3.1). The authors also reported similar hazard ratios (HR), which were adjusted for the other treatment assignments (vitamin E, beta-carotene, and multivitamin treatment assignments). These HRs were 0.96 (0.78 to 1.18) for any AMD, and 0.94 (0.60 to 1.4) for late AMD.

Neovascular AMD and geographic atrophy were not reported separately.

Multivitamin versus placebo

Summary of findings 4

One study reported this comparison (PHS II 2012). This study took place in the USA; they enrolled men only and followed them up for an average of 11 years. Data were available for a total of 14,233 participants.

The risk ratio for any AMD was 1.21 (95% CI 1.02 to 1.43; moderate-certainty evidence), and 1.22 (95% CI 0.88 to 1.69; moderate certainty evidence; Analysis 4.1) for late AMD. The authors also reported HRs, which were adjusted for the cohort (PHS I and PHS II) and other treatment assignments (vitamin C, vitamin E, and beta-carotene). These HRs were 1.22 (95% CI 1.03 to 1.44) for any AMD and 1.22 (95% CI 0.88 to 1.70) for late AMD.

Neovascular AMD and geographic atrophy were not reported separately.

Adverse effects

Included studies

In general, the publications that reported eye outcomes did not report adverse effects. We also looked at the reports of the main study results for those trials where eye outcomes were collected as part of a larger trial.

In VECAT 2002, it was noted that no serious adverse effects were seen. Similar numbers of people in the vitamin E and placebo groups withdrew due to adverse effects (four versus seven), reported any adverse effect (91 versus 83), or ocular adverse effect (105 versus 90).

The main ATBC 1998 trial found an increased risk of lung cancer associated with beta-carotene supplementation (ATBC 1994), a finding that was repeated in the large CARET trial (Omenn 1996). Beta-carotene supplementation is contra-indicated in people who smoke or have been exposed to asbestos.

In the main WHS 2010 trial, "We examined whether vitamin E increased adverse effects due to bleeding (gastrointestinal bleeding, hematuria, easy bruising, epistaxis) because of the potential for vitamin E to inhibit platelet function, gastrointestinal symptoms (gastric upset, nausea, diarrhea, constipation), or fatigue. There were no differences between reported adverse effects for any of these variables among women in the two groups, apart from a small, but significant, increase in the risk of epistaxis (RR 1.06, 95% CI 1.01 to 1.11; P = 0.02)" (Lee 2005).

In PHS II 2012, "An excess number of hemorrhagic strokes was observed among those assigned to vitamin E compared with placebo (39 versus 23 events; HR 1.74, 95% CI 1.04 to 2.91)" (Gaziano 2009), and"We observed no significant differences in adverse effects, including hematuria, easy bruising, and epistaxis, for active vitamin E or C compared with placebo"(Sesso 2008). For multivitamins "...no significant effects on gastrointestinal tract symptoms (peptic ulcer, constipation, diarrhea, gastritis, and nausea), fatigue, drowsiness, skin discoloration, and migraine (all P > 0.05). Those taking the active versus placebo multivitamin were more likely to have skin rashes (2111 and 1973 men in corresponding active and placebo multivitamin groups; HR 1.08, 95% CI 1.01 to 1.15; P = 0.016). In addition, there were inconsistent findings for daily multivitamin use on minor bleeding, with a reduction in hematuria (998 and 1105 men in corresponding active and placebo multivitamin groups; HR 0.89, 95% CI 0.81 to 0.97; P = 0.006), an increase in epistaxis (1216 and 1106 men in corresponding active and placebo multivitamin groups; HR 1.11, 95% CI 1.02 to 1.20; P = 0.016), and no effect on easy bruising/ other bleeding (1927 and 1902 men in corresponding active and placebo multivitamin groups; HR 1.02, 95% CI 0.96 to 1.08; P = 0.59)" (Gaziano 2012).

Other systematic reviews

Reviews identified by the search in Appendix 9, for example, Huang 2006, did not identify any consistent adverse effects of mineral and vitamin supplements, but only included nine RCTs in their review. A subsequent Cochrane Review including 78 trials with 296,707 participants concluded "We found no evidence to support

Copyright © 2017 The Cochrane Collaboration. Published by John Wiley & Sons, Ltd.

antioxidant supplements for primary or secondary prevention. Beta-carotene and vitamin E seem to increase mortality, and so may higher doses of vitamin A" (Bjelakovic 2012).

DISCUSSION

Summary of main results

This review provides evidence that people who take vitamin E or beta-carotene supplements do not reduce their risk of developing age-related macular degeneration (AMD; Summary of findings for the main comparison; Summary of findings 2). There is more limited evidence on vitamin C (Summary of findings 3), and one multivitamin (Centrum Silver, Summary of findings 4), but at present, nothing to suggest that these supplements prevent AMD.

Overall completeness and applicability of evidence

This review includes five large, high-quality studies that randomised over 75,000 members of the population to antioxidant supplementation or placebo. Duration of supplementation in these studies ranged from 4 to 12 years.

In ATBC 1994, there was no association with the treatment group and development of early stages of the disease. If anything, there was a tendency for more cases to be present in the treatment rather than the placebo group. This was not statistically significant. One drawback of adding a maculopathy study to a trial of primary prevention is that we have no information on maculopathy status before supplementation. Therefore, we have to assume that (1) maculopathy was equally distributed across study groups at the start of the study, and (2) most observed events occurred during the study period. It is likely that this was true for a reasonable proportion of the events, as the maculopathy study began eight years after recruitment for the main trial, and randomisation should have ensured equal distribution of maculopathy between the two groups.

Supplementation in this study began at age 50 to 69 years and lasted for five to eight years. Currently, we do not know at what age antioxidant protection may be important. It may be that this was too late or too short a period of supplementation to show an effect. This study was conducted in Finnish male smokers, and we have to be cautious in extrapolating the findings to other geographical areas, to people in other age groups, to women, and to non-smokers. However, the incidence of AMD, particularly neovascular disease, is likely to be higher in smokers, which means that they provide a good population to demonstrate any potential protective effects of antioxidant supplementation (Klein 1993).

Similarly, the results of VECAT 2002 do not provide evidence of a benefit of supplementation in people with no, mild, or borderline AMD, although again, these studies have been underpowered to examine late-stage disease.

In the PHS I 2007, over 20,000 physicians received supplementation with beta-carotene over 12 years. There was little evidence of any benefit of beta-carotene supplementation. They used medical record review to ascertain AMD, and therefore may have been less accurate. However, there was no reason to suppose that the ascertainment would have been different in the treatment and control groups. The same method of ascertainment was used in PHS II 2012 and WHS 2010.

This review does not provide evidence on the effects of other antioxidant vitamin and mineral supplements on the development of AMD; in particular, it does not provide evidence on the effects of commonly marketed vitamin combinations.

There are additional ongoing studies, including the Women's antioxidant cardiovascular study (WACS), and SELECT study that are collecting data on AMD outcomes.

Although generally regarded as safe, antioxidant supplements may have harmful effects. Our review does not provide a systematic review of all possible adverse effects of supplements, but does highlight the fact that beta-carotene increases the risk of lung cancer and overall, there was some evidence of a small, increased risk of mortality in people who took beta-carotene or vitamin E.

Quality of the evidence

Overall, the certainty of the evidence was considered to be high for any AMD, moderate for late AMD (due to lower number of events), and very low for neovascular AMD or geographic atrophy, which were only reported disaggregated in one study.

Although the number of people randomised in these studies was large, there is still a degree of uncertainty in the pooled estimates. In the pooled analyses, the risk ratios were largely around the null value, or just above the null value.

Potential biases in the review process

We did not include the Age-related Eye Disease Study in this review. However, there were 2180 people recruited with no, mild, or borderline AMD (AREDS 2001a). The study reported no benefit of the study treatment for these people, however, the number of events was small.

Agreements and disagreements with other studies or reviews

A review of observational prospective studies in 2007 found little evidence of a protective effect of dietary antioxidants (Chong 2007). The only dietary antioxidant for which a reduction was seen was vitamin E, in contrast to the evidence from the trials included in this review. It is possible that natural vitamin E from dietary sources rather than artificial supplements has different effects, or alternatively, high levels of dietary vitamin E might be a marker for other nutrients.

There are many reviews focusing on the role of antioxidant vitamin and mineral supplements in people with AMD, but we have only identified one that considered specifically their role in healthy people (Zampatti 2014). The conclusions were similar to the current review that there is little evidence for any benefit in healthy people.

AUTHORS' CONCLUSIONS

Implications for practice

Taking vitamin E or beta-carotene supplements will not prevent or delay the onset of AMD. The same probably applies to vitamin C and the multivitamin (Centrum Silver) investigated in the one trial reported to date. There is no evidence with respect to other antioxidant supplements, such as lutein and zeaxanthin.

Copyright © 2017 The Cochrane Collaboration. Published by John Wiley & Sons, Ltd.

Implications for research

There are a number of unanswered questions in the prevention of AMD. The hypothesis that antioxidant micronutrients may protect against the disease is a reasonable one. We do not know at what stage the protective effect may be important, nor the potential interactions with genetic effects and other risk factors for the disease, such as smoking. The research to date suggests that vitamin supplements (at least those studied) do not prevent AMD. They do not provide conclusive evidence on safety. The small number of incident events in healthy people mean that any future trials need to be very large. Four large primary and secondary prevention trials in the field of cancer and cardiovascular disease have added an examination of eye disease. This would seem to be a cost-effective way forward in research in this area.

ACKNOWLEDGEMENTS

This work was undertaken in collaboration with the National Institute for Health and Care Excellence. The views expressed in this publication are those of the authors and not necessarily those of NICE.

We are grateful to:

- the Systematic Review Training Unit at the Institute of Child Health, London for advice on the protocol for this review;
- all the trialists who responded to requests for information;
- peer reviewers Andrew Ness and Usha Chakravarthy for comments on an earlier version of this review.
- Carol Mccletchie OBE who reviewed and commented on the plain language summary from the consumer perspective.

We thank Katherine Henshaw who was an author on the original review. The Cochrane Eyes and Vision editorial team prepared and executed the electronic searches for this review. We are grateful to Anupa Shah and Iris Gordon for their assistance with the review process.

Copyright © 2017 The Cochrane Collaboration. Published by John Wiley & Sons, Ltd.

REFERENCES

References to studies included in this review

ATBC 1998 *{published data only}*

Albanes D, Heinonen OP, Huttunen JK, Taylor PR, Virtamo J, Edwards BK, et al. Effects of alpha-tocopherol and beta-carotene supplements on cancer incidence in the Alpha-tocopherol beta-carotene cancer prevention study. *American Journal of Clinical Nutrition* 1995;**62**(6):1427S-30S.

Albanes D, Heinonen OP, Taylor PR, Virtamo J, Edwards BK, Rautalahti M, et al. Alpha-tocopherol and beta-carotene supplements and lung cancer incidence in the Alpha-tocopherol, beta-carotene cancer prevention study: effects of base-line characteristics and study compliance. *Journal of the National Cancer Institute* 1996;**88**(21):1560-70.

Albanes D, Virtamo J, Rautalahti M, Pikkarainen J, Taylor PR, Greenwald P, et al. Pilot study: The US-Finland lung cancer prevention trial. *Journal of Nutrition, Growth and Cancer* 1986;**3**(3):207-14.

Albanes D, Virtamo J, Taylor PR, Rautalahti M, Pietinen P, Heinonen OP. Effects of supplemental beta-carotene, cigarette smoking, and alcohol consumption on serum carotenoids in the Alpha-tocopherol, beta-carotene cancer prevention study. *American Journal of Clinical Nutrition* 1997;**66**(2):366-72.

Blumberg J, Block G. The Alpha-tocopherol, beta-carotene cancer prevention study in Finland. *Nutrition Review* 1994;**52**(7):242-5.

Buring JE, Hebert P, Hennekens CH. The Alpha-tocopherol, beta-carotene lung cancer prevention trial of vitamin E and beta-carotene: the beginning of the answers. *Annals of Epidemiology* 1994;**4**(1):75.

Glynn SA, Albanes D, Pietinen P, Brown CC, Rautalahti M, Tangrea JA, et al. Alcohol consumption and risk of colorectal cancer in a cohort of Finnish men. *Cancer Causes and Control* 1996;**7**(2):214-23.

Hartman TJ, Albanes D, Pietinen P, Hartman AM, Rautalahti M, Tangrea JA, et al. The association between baseline vitamin E, selenium, and prostate cancer in the Alpha-tocopherol, beta-carotene cancer prevention study. *Cancer Epidemiology, Biomarkers and Prevention* 1998;**7**(4):335-40.

Heinonen OP, Albanes D, Virtamo J, Taylor PR, Huttunen JK, Hartman AM, et al. Prostate cancer and supplementation with alpha-tocopherol and beta-carotene: incidence and mortality in a controlled trial. *Journal of the National Cancer Institute* 1998;**90**:440-6.

Laurila AL, Anttila T, Laara E, Bloigu A, Virtamo J, Albanes D, et al. Serological evidence of an association between *Chlamydia pneumoniae* infection and lung cancer. *International Journal of Cancer* 1997;**74**(1):31-4.

Liede K, Hietanen J, Saxen L, Haukka J, Timonen T, Hayrinen Immonen R, et al. Long-term supplementation with alpha-tocopherol and beta-carotene and prevalence of oral mucosal lesions in smokers. *Oral Diseases* 1998;**4**(2):78-83.

Liede KE, Alfthan G, Hietanen JH, Haukka JK, Saxen LM, Heinonen OP. Beta-carotene concentration in buccal mucosal cells with and without dysplastic oral leukoplakia after long-term beta-carotene supplementation in male smokers. *European Journal of Clinical Nutrition* 1998;**52**:872-6.

Liede KE, Haukka JK, Saxen LM, Heinonen OP. Increased tendency towards gingival bleeding caused by joint effect of alpha-tocopherol supplementation and acetylsalicylic acid. *Annals of Medicine* 1998;**30**(6):542-6.

Pietinen P, Rimm EB, Korhonen P, Hartman AM, Willett WC, Albanes D, et al. Intake of dietary fiber and risk of coronary heart disease in a cohort of Finnish men. The Alpha-tocopherol, beta-carotene cancer prevention study. *Circulation* 1996;**94**(11):2720-7.

Rapola JM, Virtamo J, Haukka JK, Heinonen OP, Albanes D, Taylor PR, et al. Effect of vitamin E and beta carotene on the incidence of angina pectoris. A randomized, double-blind, controlled trial. *JAMA* 1996;**275**(9):693-8.

Rapola JM, Virtamo J, Ripatti S, Haukka JK, Huttunen JK, Albanes D, et al. Effects of alpha tocopherol and beta carotene supplements on symptoms, progression, and prognosis of angina pectoris. *Heart* 1998;**79**(5):454-8.

Rapola JM, Virtamo J, Ripatti S, Huttunen JK, Albanes D, Taylor PR, et al. Randomised trial of alpha-tocopherol and beta-carotene supplements on incidence of major coronary events in men with previous myocardial infraction. *Lancet* 1997;**349**(9067):1715-20.

Teikari JM, Laatikainen L, Rapola JM, Virtamo J, Haukka J, Liesto K, et al. Retinal vascular changes following supplementation with alpha-tocopherol or beta-carotene. *Acta Ophthalmologica Scandinavica* 1998;**76**(1):68-73.

* Teikari JM, Laatikainen L, Virtamo J, Haukka J, Rautalahti M, Liesto K, et al. Six-year supplementation with alpha-tocopherol and beta-carotene and age-related maculopathy. *Acta Ophthalmologica Scandinavica* 1998;**76**(2):224-9.

Teikari JM, Rautalahti M, Haukka J, Jarvinen P, Hartman AM, Virtamo J, et al. Incidence of cataract operations in Finnish male smokers unaffected by alpha tocopherol or beta carotene supplements. *Journal of Epidemiology and Community Health* 1998;**52**(7):468-72.

Teikari JM, Virtamo J, Rautalahti M, Palmgren J, Liesto K, Heinonen OP. Long-term supplementation with alpha-tocopherol and beta-carotene and age-related cataract. *Acta Ophthalmologica Scandinavica* 1997;**75**(6):634-40.

The ATBC Cancer Prevention Study Group. The Alpha-tocopherol, beta-carotene lung cancer prevention study: design, methods, participant characteristics, and compliance. *Annals of Epidemiology* 1994;**4**(1):1-10.

The Alpha-tocopherol, Beta-carotene Cancer Prevention Study Group. The effect of vitamin E and beta carotene on the

Copyright © 2017 The Cochrane Collaboration. Published by John Wiley & Sons, Ltd.

incidence of lung cancer and other cancers in male smokers. *New England Journal of Medicine* 1994;**330**(15):1029-35.

Tornwall M, Virtamo J, Haukka JK, Aro A, Albanes D, Edwards BK, et al. Effect of alpha-tocopherol (vitamin E) and beta-carotene supplementation on the incidence of intermittent claudication in male smokers. *Arteriosclerosis, Thrombosis and Vascular Biology* 1997;**17**(12):3475-80.

Varis K, Taylor PR, Sipponen P, Samloff IM, Heinonen OP, Albanes D, et al. Gastric cancer and premalignant lesions in atrophic gastritis: a controlled trial on the effect of supplementation with alpha-tocopherol and beta-carotene. The Helsinki gastritis study group. *Scandinavian Journal of Gastroenterology* 1998;**33**(3):294-300.

Virtamo J, Rapola JM, Ripatti S, Heinonen OP, Taylor PR, Albanes D, et al. Effect of vitamin E and beta-carotene on the incidence of primary nonfatal myocardial infarction and fatal coronary heart disease. *Archives of Internal Medicine* 1998;**158**(6):668-75.

PHS I 2007 *{published data only}*

Belanger C, Buring JE, Cook N, Eberlein K, Goldhaber SZ, Gordon D, et al. Final report on the aspirin component of the ongoing Physicians' health study. *New England Journal of Medicine* 1989;**321**(3):129-35.

Berger K, Kase CS, Buring JE. Interobserver agreement in the classification of stroke in the Physicians' health study. *Stroke* 1996;**27**(2):238-42.

Buring JE, Hebert P, Romero J, Kittross A, Cook N, Manson J, et al. Migraine and subsequent risk of stroke in the Physicians' health study. *Archives of Neurology* 1995;**52**(2):129-34.

Buring JE, Hennekens CH. Cost and efficiency in clinical trials: The U.S. Physicians' health study. *Statistics in Medicine* 1990;**9**(1-2):29-33.

Cairns J, Cohen L, Colton T, DeMets DL, Deykin D, Friedman L, et al. Issues in the early termination of the aspirin component of the Physicians' health study. *Annals of Epidemiology* 1991;**1**(5):395-405.

Camargo CA Jr, Stampfer MJ, Glynn RJ, Gaziano JM, Manson JE, Goldhaber SZ, et al. Prospective study of moderate alcohol consumption and risk of peripheral arterial disease in US male physicians. *Circulation* 1997;**95**(3):577-80.

Christen WG, Ajani UA, Glynn RJ, Manson JE, Schaumberg DA, Chew EC, et al. Prospective cohort study of antioxidant vitamin supplement use and the risk of age-related maculopathy. *American Journal of Epidemiology* 1999;**149**(5):476-84.

Christen WG, Glynn RJ, Manson JE, Agani UA, Buring JE. A prospective study of cigarette smoking and risk of age-related macular degeneration in men. *JAMA* 1996;**276**(14):1147-51.

Christen WG, Manson JE, Glynn RJ, Ajani UA, Schaumberg DA, Sperduto RD, et al. Low-dose aspirin and risk of cataract and subtypes in a randomized trial of U.S. physicians. *Ophthalmic Epidemiology* 1998;**5**(3):133-42.

* Christen WG, Manson JE, Glynn RJ, Gaziano JM, Chew EY, Buring JE, et al. Beta carotene supplementation and age-related maculopathy in a randomized trial of US physicians. *Archives of Ophthalmology* 2007;**125**(3):333-9.

Christen WG, Manson JE, Seddon JM, Glynn RJ, Buring JE, Rosner B, et al. A prospective study of cigarette smoking and risk of cataract in men. *JAMA* 1992;**268**(8):989-93.

Fotouhi N, Meydani M, Santos MS, Meydani SN, Hennekens CH, Gaziano JM. Carotenoid and tocopherol concentrations in plasma, peripheral blood mononuclear cells, and red blood cells after long-term beta-carotene supplementation in men. *American Journal of Clinical Nutrition* 1996;**63**(4):553-8.

Gann PH, Manson JE, Glynn RJ, Buring JE, Hennekens CH. Low-dose aspirin and incidence of colorectal tumors in a randomized trial. *Journal of the National Cancer Institute* 1993;**85**(15):1220-4.

Glynn RJ, Christen WG, Manson JE, Bernheimer J, Hennekens CH. Body mass index. An independent predictor of cataract. *Archives of Ophthalmology* 1995;**113**(9):1131-7.

Guallar E, Hennekens CH, Sacks FM, Willett WC, Stampfer MJ. A prospective study of plasma fish oil levels and incidence of myocardial infarction in U.S. male physicians. *Journal of the American College of Cardiology* 1995;**25**(2):387-94.

Hebert PR, Rich Edwards JW, Manson JE, Ridker PM, Cook NR, O'Connor GT, et al. Height and incidence of cardiovascular disease in male physicians. *Circulation* 1993;**88**(4 (Pt 1)):1437-43.

Hennekens CH, Buring JE, Manson JE, Stampfer M, Rosner B, Cook NR, et al. Lack of effect of long-term supplementation with beta-carotene on the incidence of malignant neoplasms and cardiovascular disease. *New England Journal of Medicine* 1996;**334**(18):1145-9.

Hennekens CH, Eberlein K. A randomized trial of aspirin and beta-carotene among U.S. physicians. *Preventive Medicine* 1985;**14**(2):165-8.

Lang JM, Buring JE, Rosner B, Cook N, Hennekens CH. Estimating the effect of the run-in on the power of the Physicians' health study. *Statistics in Medicine* 1991;**10**(10):1585-93.

Lee IM, Hennekens CH, Berger K, Buring JE, Manson JE. Exercise and risk of stroke in male physicians. *Stroke* 1999;**30**(1):1-6.

Ma J, Stampfer MJ, Giovannucci E, Artigas C, Hunter DJ, Fuchs C, et al. Methylenetetrahydrofolate reductase polymorphism, dietary interactions, and risk of colorectal cancer. *Cancer Research* 1997;**57**(6):1098-102.

Manson JE, Buring JE, Satterfield S, Hennekens CH. Baseline characteristics of participants in the Physicians' health study: a randomized trial of aspirin and beta-carotene in U.S. physicians. *American Journal of Preventive Medicine* 1991;**7**(3):150-4.

Manson JE, Christen WG, Seddon JM, Glynn RJ, Hennekens CH. A prospective study of alcohol consumption and risk of cataract. *American Journal of Preventive Medicine* 1994;**10**(3):156-61.

Copyright © 2017 The Cochrane Collaboration. Published by John Wiley & Sons, Ltd.

Morris MC, Manson JE, Rosner B, Buring JE, Willett WC, Hennekens CH. Fish consumption and cardiovascular disease in the Physicians' health study: a prospective study. *American Journal of Epidemiology* 1995;**142**(2):166-75.

O'Donnell CJ, Ridker PM, Glynn RJ, Berger K, Ajani U, Manson JE, et al. Hypertension and borderline isolated systolic hypertension increase risks of cardiovascular disease and mortality in male physicians. *Circulation* 1997;**95**(5):1132-7.

Ridker PM, Cushman M, Stampfer MJ, Tracy RP, Hennekens CH. Plasma concentration of C-reactive protein and risk of developing peripheral vascular disease. *Circulation* 1998;**97**(5):425-8.

Ridker PM, Hennekens CH, Roitman Johnson B, Stampfer MJ, Allen J. Plasma concentration of soluble intercellular adhesion molecule 1 and risks of future myocardial infarction in apparently healthy men. *Lancet* 1998;**351**(9004):88-92.

Ridker PM, Manson JE, Gaziano JM, Buring JE, Hennekens CH. Low-dose aspirin therapy for chronic stable angina. A randomized, placebo-controlled clinical trial. *Annals of Internal Medicine* 1991;**114**(10):835-9.

Robbins AS, Manson JE, Lee IM, Satterfield S, Hennekens CH. Cigarette smoking and stroke in a cohort of U.S. male physicians. *Annals of Internal Medicine* 1994;**120**(6):458-62.

Salvini S, Stampfer MJ, Barbieri RL, Hennekens CH. Effects of age, smoking and vitamins on plasma DHEAS levels: a cross-sectional study in men. *Journal of Clinical Endocrinology and Metabolism* 1992;**74**(1):139-43.

Santos MS, Gaziano JM, Leka LS, Beharka AA, Hennekens CH, Meydani SN. Beta-carotene-induced enhancement of natural killer cell activity in elderly men: an investigation of the role of cytokines. *American Journal of Clinical Nutrition* 1998;**68**(1):164-70.

Santos MS, Leka LS, Ribaya Mercado JD, Russell RM, Meydani M, Hennekens CH, et al. Short- and long-term beta-carotene supplementation do not influence T cell-mediated immunity in healthy elderly persons. *American Journal of Clinical Nutrition* 1997;**66**(5):917-24.

Santos MS, Meydani SN, Leka L, Wu D, Fotouhi N, Meydani M, et al. Natural killer cell activity in elderly men is enhanced by beta-carotene supplementation. *American Journal of Clinical Nutrition* 1996;**64**:772-7.

Satterfield S, Greco PJ, Goldhaber SZ, Stampfer MJ, Swartz SL, Stein EA, et al. Biochemical markers of compliance in the Physicians' health study. *American Journal of Preventive Medicine* 1990;**6**(5):290-4.

Schaumberg DA, Hennekens CH. Effect of 12 years of beta-carotene supplementation on malignant non-melanoma skin cancer: results from the Physicians' health study. *American Journal of Epidemiology* 1996;**143**:S9.

Seddon JM, Christen WG, Manson JE, LaMotte FS, Glynn RJ, Buring JE, et al. The use of vitamin supplements and the risk of cataract among US male physicians. *American Journal of Public Health* 1994;**84**(5):788-92.

Stampfer MJ, Buring JE, Willett W, Rosner B, Eberlein K, Hennekens CH. The 2 x 2 factorial design: its application to a randomized trial of aspirin and carotene in U.S. physicians. *Statistics in Medicine* 1985;**4**(2):111-6.

Verhoef P, Hennekens CH, Malinow MR, Kok FJ, Willett WC, Stampfer MJ. A prospective study of plasma homocyst(e)ine and risk of ischemic stroke. *Stroke* 1994;**25**(10):1924-30.

Zee RY, Ridker PM, Stampfer MJ, Hennekens CH, Lindpaintner K. Prospective evaluation of the angiotensin-converting enzyme insertion/deletion polymorphism and the risk of stroke. *Circulation* 1999;**99**(3):340-3.

PHS II 2012 *{published data only}*

Christen WG, Gaziano JM, Hennekens CH. Design of Physicians' health study II - a randomized trial of beta-carotene, vitamins E and C, and multivitamins, in prevention of cancer, cardiovascular disease, and eye disease and review of results of completed trials. *Annals of Epidemiology* 2000;**10**(2):125-34.

Christen WG, Glynn RJ, Sesso HD, Kurth T, Macfadyen J, Bubes V, et al. Vitamins E and C and medical record-confirmed age-related macular degeneration in a randomized trial of male physicians. *Ophthalmology* 2012;**119**(8):1642-9.

VECAT 2002 *{published data only}*

Garrett SKM, McNeil JJ, Silagy C, Sinclair M, Thomas AP, Robman LD, et al. Methodology of the VECAT study: vitamin E intervention in cataract and age-related maculopathy. *Ophthalmic Epidemiology* 1999;**6**:195-208.

Robman LD, Tikellis G, Garrett SKM, Harper CA, McNeil JJ, Taylor HR, et al. Baseline ophthalmic findings in the vitamin E, cataract and age-related maculopathy (VECAT) study. *Australian and New Zealand Journal of Ophthalmology* 1999;**27**:410-6.

* Taylor H, Tikellis G, Robman L, McCarty C, McNeil J. Vitamin E supplementation and macular degeneration: randomised controlled trial. *BMJ* 2002;**325**:325-11.

Taylor HR, Tikellis G, Robman LD, McCarty CA, McNeil. Vitamin E supplementation and age-related maculopathy. *Investigative Ophthalmology and Visual Science* 2001;**42**:S311.

WHS 2010 *{published data only}*

Buring JE, Hennekens CH. Randomized trials of primary prevention of cardiovascular disease in women. An investigator's view. *Annals of Epidemiology* 1994;**4**(2):111-4.

Christen WG, Glynn RJ, Chew EY, Buring JE. Vitamin E and age-related macular degeneration in a randomized trial of women. *Ophthalmology* 2010;**117**(6):1163-8.

Cook NR, Lee IM, Gaziano JM, Gordon D, Ridker PM, Manson JE, et al. Low-dose aspirin in the primary prevention of cancer: the Women's health study: a randomized controlled trial. *JAMA* 2005;**294**(1):47-55.

Lee IM, Cook NR, Gaziano JM, Gordon D, Ridker PM, Manson JE, et al. Vitamin E in the primary prevention of cardiovascular

Copyright © 2017 The Cochrane Collaboration. Published by John Wiley & Sons, Ltd.

disease and cancer: the Women's health study: a randomized controlled trial. *JAMA* 2005;**294**(1):56-65.

Ridker PM, Cook NR, Lee IM, Gordon D, Gaziano JM, Manson JE, et al. A randomized trial of low-dose aspirin in the primary prevention of cardiovascular disease in women. *New England Journal of Medicine* 2005;**352**(13):1293-304.

References to studies excluded from this review

ADSC 1997 *{published data only}*

Sano M, Ernesto C, Thomas RG, Klauber MR, Schafer K, Grundman M, et al. A controlled trial of selegiline, alpha-tocopherol, or both as treatment for Alzheimer's disease. The Alzheimer's Disease Cooperative Study. *New England Journal of Medicine* 1997;**336**(17):1216-22.

Andrews 1969 *{published data only}*

Andrews J, Letcher M, Brook M. Vitamin C supplementation in the elderly: a 17-month trial in an old persons' home. *British Medical Journal* 1969;**2**(5654):416-8.

AREDS 2001 *{published data only}*

* Age-related Eye Disease Study Research Group. A randomized, placebo-controlled, clinical trial of high-dose supplementation with vitamins C and E, beta carotene, and zinc for age-related macular degeneration and vision loss: AREDS report no. 8. *Archives of Ophthalmology* 2001;**119**(10):1417-36.

AREDS2 2008 *{published data only}*

Hubbard LD, Danis RP, Neider MW, Thayer DW, Wabers HD, White JK, et al. Brightness, contrast, and color balance of digital versus film retinal images in the age-related eye disease study 2. *Investigative Ophthalmology and Visual Science* 2008;**49**(8):3269-82.

Benner 1994 *{published data only}*

Benner SE, Lippman SM, Hong WK. Retinoid chemoprevention of second primary tumors. *Seminars in Hematolology* 1994;**31**(4 Suppl 5):26-30.

Benton 1995 *{published data only}*

* Benton D, Fordy J, Haller J. The impact of long-term vitamin supplementation on cognitive functioning. *Psychopharmacology* 1995;**117**(3):298-305.

Berrow 2016 *{published data only}*

Berrow EJ, Bartlett HE, Eperjesi F. The effect of nutritional supplementation on the multifocal electroretinogram in healthy eyes. *Documenta Ophthalmologica* 2016;**132**(2):123-35.

Blok 1997 *{published data only}*

Blok WL, Deslypere JP, Demacker PN, Van der Ven Jongekrijg J, Hectors MP, Van der Meer JW, et al. Pro- and anti-inflammatory cytokines in healthy volunteers fed various doses of fish oil for 1 year. *European Journal of Clinical Investigation* 1997;**27**(12):1003-8.

Bogden 1990 *{published data only}*

Bogden JD, Oleske JM, Lavenhar MA, Munves EM, Kemp FW, Bruening KS, et al. Effects of one year of supplementation with zinc and other micronutrients on cellular immunity in the elderly. *Journal of the American College of Nutrition* 1990;**9**(3):214-25.

Bone 2010 *{published data only}*

Bone RA, Landrum JT. Dose-dependent response of serum lutein and macular pigment optical density to supplementation with lutein esters. *Archives of Biochemistry and Biophysics* 2010;**504**(1):50-5.

Brewer 1997 *{published data only}*

Brewer GJ, Johnson V, Kaplan J. Treatment of Wilson's disease with zinc: XIV. Studies of the effect of zinc on lymphocyte function. *Journal of Laboratory and Clinical Medicine* 1997;**129**(6):649-52.

Brown 1998 *{published data only}*

Brown BG, Zhao XQ, Chait A, Frohlich J, Cheung M, Heise N, et al. Lipid altering or antioxidant vitamins for patients with coronary disease and very low HDL cholesterol? The HDL-atherosclerosis treatment study design. *Canadian Journal of Cardiology* 1998;**14 Suppl A**:6A-13A.

Bussey 1982 *{published data only}*

Bussey HJ, DeCosse JJ, Deschner EE, Eyers AA, Lesser ML, Morson BC, et al. A randomized trial of ascorbic acid in polyposis coli. *Cancer* 1982;**50**(7):1434-9.

Caligiuri 1997 *{published data only}*

Caligiuri MP, Lohr JB, Rotrosen J, Adler L, Lavori P, Edson R, et al. Reliability of an instrumental assessment of tardive dyskinesia: results from VA cooperative study 394. *Psychopharmacology* 1997;**132**(1):61-6.

CARET 1996 *{published data only}*

* Omenn GS, Goodman GE, Thornquist MD, Balmes J, Cullen MR, Glass A, et al. Effects of a combination of beta-carotene and vitamin A on lung cancer and cardiovascular disease. *New England Journal of Medicine* 1996;**334**(18):1150-5.

CARMIS 2011 *{published data only}*

Piermarocchi S, Saviano S, Parisi V, Tedeschi M, Panozzo G, Scarpa G, et al. Carotenoids in age-related maculopathy Italian study (CARMIS): two-year results of a randomized study. *European Journal of Ophthalmology* 2011;**22**(2):216-25.

CCSG 1993 *{published data only}*

Roncucci L, Di Donato P, Carati L, Ferrari A, Perini M, Bertoni G, et al. Antioxidant vitamins or lactulose for the prevention of the recurrence of colorectal adenomas. Colorectal Cancer Study Group of the University of Modena and the Health Care District 16. *Diseases of the Colon and Rectum* 1993;**36**(3):227-34.

Chandra 1992 *{published data only}*

* Chandra RK. Effect of vitamin and trace-element supplementation on immune responses and infection in elderly subjects. *Lancet* 1992;**340**(8828):1124-7.

CHAOS 1996 *{published data only}*

* Stephens NG, Parsons A, Schofield PM, Kelly F, Cheeseman K, Mitchinson MJ, et al. Randomised controlled trial of vitamin E

Copyright © 2017 The Cochrane Collaboration. Published by John Wiley & Sons, Ltd.

in patients with coronary disease: Cambridge heart antioxidant study (CHAOS). *Lancet* 1996;**347**(9004):781-6.

Clausen 1989 *{published data only}*

Clausen J, Nielsen SA, Kristensen M. Biochemical and clinical effects of an antioxidative supplementation of geriatric patients. A double blind study. *Biological Trace Element Research* 1989;**20**(1-2):135-51.

Constans 1996 *{published data only}*

Constans J, Delmas Beauvieux MC, Sergeant C, Peuchant E, Pellegrin JL, Pellegrin I, et al. One-year antioxidant supplementation with beta-carotene or selenium for patients infected with human immunodeficiency virus: a pilot study. *Clinical Infectious Diseases* 1996;**23**(3):654-6.

Constantino 1988 *{published data only}*

Costantino JP, Kuller LH, Begg L, Redmond CK, Bates MW. Serum level changes after administration of a pharmacologic dose of beta-carotene. *American Journal of Clinical Nutrition* 1988;**48**(5):1277-83.

Cucinotta 1994 *{published data only}*

Cucinotta D, Senin U, Girardello R, Crepaldi G. Posatirelin effect on patients with senile dementia of Alzheimer type (SDAT): a double blind multicentre trial versus ascorbic acid and citicoline. *Journal of Neurology* 1994;**241**:S129.

DATATOP 1989 *{published data only}*

Shoulson I, Fahn S, Oakes D, Lang A, Langston JW, LeWitt P, et al. DATATOP: A multicenter controlled clinical trial in early Parkinson's disease. *Archives of Neurology* 1989;**46**(10):1052-60.

DeCosse 1989 *{published data only}*

DeCosse JJ, Miller HH, Lesser ML. Effect of wheat fiber and vitamins C and E on rectal polyps in patients with familial adenomatous polyposis. *Journal of the National Cancer Institute* 1989;**81**(17):1290-7.

De Klerk 1998 *{published data only}*

De Klerk NH, Musk AW, Ambrosini GL, Eccles JL, Hansen J, Olsen N, et al. Vitamin A and cancer prevention II: comparison of the effects of retinol and beta-carotene. *International Journal of Cancer* 1998;**75**:362-7.

Dobson 1984 *{published data only}*

Dobson HM, Muir MM, Hume R. The effect of ascorbic acid on the seasonal variations in serum cholesterol levels. *Scottish Medical Journal* 1984;**29**(3):176-82.

ECP-IM 1995 *{published data only}*

Reed PI, Johnston BJ. Primary prevention of gastric cancer - The ECP-IM intervention study. *Acta Endoscopica* 1995;**25**:45-54.

EUROSCAN 1994 *{published data only}*

Cianfriglia F, Iofrida RV, Calpicchio A, Manieri A. The chemoprevention of oral carcinoma with vitamin A and/or N-acetylcysteine. *Minerva Stomatologica* 1994;**43**(6):255-61.

Fairley 1996 *{published data only}*

Fairley CK, Tabrizi SN, Chen S, Baghurst P, Young H, Quinn M, et al. A randomized clinical trial of beta carotene vs placebo for the treatment of cervical HPV infection. *International Journal of Gynecological Cancer* 1996;**6**(3):225-30.

Falsani 2010 *{published data only}*

Falsini B, Piccardi M, Minnella A, Savastano C, Capoluongo E, Fadda A, et al. Influence of saffron supplementation on retinal flicker sensitivity in early age-related macular degeneration. *Investigative Ophthalmology and Visual Science* 2010;**51**(12):6118-24.

Fontham 1995 *{published data only}*

Fontham ET, Malcom GT, Singh VN, Ruiz B, Schmidt B, Correa P. Effect of beta-carotene supplementation on serum alpha-tocopherol concentration. *Cancer Epidemiology, Biomarkers and Prevention* 1995;**4**(7):801-3.

Galan 1997 *{published data only}*

Galan P, Preziosi P, Monget AL, Richard MJ, Arnaud J, Lesourd B, et al. Effects of trace element and/or vitamin supplementation on vitamin and mineral status, free radical metabolism and immunological markers in elderly long term-hospitalized subjects. Geriatric Network MIN. VIT. AOX. *International Journal for Vitamin and Nutrition Research* 1997;**67**(6):450-60.

Garawal 1995 *{published data only}*

Garewal H, Meyskens F, Katz RV, Friedman S, Morse DE, Alberts D, et al. Beta-carotene produces sustained remissions in oral leukoplakia: results of a 1 year randomized, controlled trial. Proceedings of the Annual Meeting of the American Society of Clinical Oncology. 1995; Vol. 14:ABS. A1623.

GISSI 1993 *{published data only}*

Marchioli R, Di Pasquale A. The biochemical, pharmacological and epidemiological reference picture of the GISSI-Prevention. The Gruppo Italiano per lo studio della Streptochinasi nell'Infarto miocardico [Il quadro di riferimento biochimico, farmacologico, epidemiologico del GISSI-Prevenzione]. *Giornale Italiano di Cardiologia* 1993;**23**(9):933-64.

HOPE 1996 *{published data only}*

Gerstein HC, Bosch J, Pogue J, Taylor DW, Zinman B, Yusuf S. Rationale and design of a large study to evaluate the renal and cardiovascular effects of an ACE inhibitor and vitamin E in high-risk patients with diabetes. The MICRO-HOPE Study. Microalbuminuria, cardiovascular, and renal outcomes. Heart outcomes prevention evaluation. *Diabetes Care* 1996;**19**(11):1225-8.

Johnson 1997 *{published data only}*

Johnson MA, Porter KH. Micronutrient supplementation and infection in institutionalized elders. *Nutrition Review* 1997;**55**(11):400-4.

Jyothirmayi 1996 *{published data only}*

Jyothirmayi R, Ramadas K, Varghese C, Jacob R, Nair MK, Sankaranarayanan R. Efficacy of vitamin A in the prevention of loco-regional recurrence and second primaries in head and

Copyright © 2017 The Cochrane Collaboration. Published by John Wiley & Sons, Ltd.

neck cancer. *European Journal of Cancer Part B Oral Oncology* 1996;**32B**(6):373-6.

Kuklinski 1994 *{published data only}*

Kuklinski B, Weissenbacher E, Fahnrich A. Coenzyme Q10 and antioxidants in acute myocardial infarction. *Molecular Aspects of Medicine* 1994;**Supplement**:S143-7.

Kvansakul 2006 *{published data only}*

Kvansakul J, Rodriguez-Carmona M, Edgar DF, Barker FM, Kopcke W, Schalch W, et al. Supplementation with the carotenoids lutein or zeaxanthin improves human visual performance. *Ophthalmic and Physiological Optics* 2006;**26**(4):362-71.

Leng 1997 *{published data only}*

Leng GC, Lee AJ, Fowkes FG, Horrobin D, Jepson RG, Lowe GD, et al. Randomized controlled trial of antioxidants in intermittent claudication. *Vascular Medicine* 1997;**2**(4):279-85.

Li 1992 *{published data only}*

Li WG. Preliminary observations on effect of selenium yeast on high risk populations with primary liver cancer. *Chung Hua Yu Fang I Hsueh Tsa Chih - Chinese Journal of Preventive Medicine* 1992;**26**(5):268-71.

LINXIAN 1993 *{published data only}*

Li JY, Li B, Blot WJ, Taylor PR. Preliminary report on the results of nutrition prevention trials of cancer and other common diseases among residents in Linxian, China. *Chung Hua Chung Liu Tsa Chih - Chinese Journal of Oncology* 1993;**15**(3):165-81.

Sperduto RD, Hu TS, Milton RC, Zhao JL, Everett DF, Cheng QF, et al. The Linxian cataract studies. Two nutrition intervention trials. *Archives of Ophthalmology* 1993;**111**(9):1246-53.

Mayne 1998 *{published data only}*

Mayne ST, Cartmel B, Silva F, Kim CS, Fallon BG, Briskin K, et al. Effect of supplemental beta-carotene on plasma concentrations of carotenoids, retinol, and alpha-tocopherol in humans. *American Journal of Clinical Nutrition* 1998;**68**(3):642-7.

McKeown 1988 *{published data only}*

McKeown Eyssen G, Holloway C, Jazmaji V, Bright See E, Dion P, Bruce WR. A randomized trial of vitamins C and E in the prevention of recurrence of colorectal polyps. *Cancer Research* 1988;**48**(16):4701-5.

Meyskens 1994 *{published data only}*

Meyskens FL Jr, Liu PY, Tuthill RJ, Sondak VK, Fletcher WS, Jewell WR, et al. Randomized trial of vitamin A versus observation as adjuvant therapy in high-risk primary malignant melanoma: a Southwest oncology group study. *Journal of Clinical Oncology* 1994;**12**(10):2060-5.

Munoz 1987 *{published data only}*

Munoz N, Wahrendorf J, Bang LJ, Crespi M, Thurnham DI, Day NE, et al. No effect of riboflavine, retinol, and zinc on prevalence of precancerous lesions of oesophagus. Randomised double-blind intervention study in high-risk population of China. *Lancet* 1985;**2**(8447):111-4.

Munoz 1996 *{published data only}*

Munoz N, Vivas J, Buiatti E, Kato I, Oliver W. Chemoprevention trial on precancerous lesions of the stomach in Venezuela: summary of study design and baseline data. *IARC Scientific Publications* 1996;**139**:125-33.

Nambour 1995 *{published data only}*

Ambler JS, Hirst LW, Clarke CV, Green AC. The Nambour study of ocular disease. I. Design, study population and methodology. *Ophthalmic Epidemiology* 1995;**2**(3):137-44.

NCT00000161 (WAFACS) *{published data only}*

NCT00000161. Randomized trials of vitamin supplements and eye disease. clinicaltrials.gov/ct2/show/NCT00000161 (accessed 7 February 2008).

NCT00718653 *{published data only}*

NCT00718653. Effects of antioxidants on human macular pigments. clinicaltrials.gov/ct2/show/NCT00718653 (accessed 21 September 2010).

NCT00893724 *{published data only}*

NCT00893724. Supplemental adjuvants for intracellular nutrition and treatment (SAINTS). clinicaltrials.gov/ct2/show/NCT00893724 (accessed 20 March 2012).

NCT01208948 *{published data only}*

NCT01208948. Clinical trial on alpha lipoic acid in diabetic macular edema (RETIPON). clinicaltrials.gov/ct2/show/NCT01208948 (accessed 17 January 2012).

Newsome, 2008 *{published data only}*

Newsome DA. A randomized, prospective, placebo-controlled clinical trial of a novel zinc-monocysteine compound in age-related macular degeneration. *Current Eye Research* 2008;**33**(7):591-8.

NPCSG 1996 *{published data only}*

Clark LC, Combs GF Jr, Turnbull BW, Slate EH, Chalker DK, Chow J, et al. Effects of selenium supplementation for cancer prevention in patients with carcinoma of the skin. A randomized controlled trial. Nutritional prevention of cancer study group. *JAMA* 1996;**276**(24):1957-63.

Pastorino 1991 *{published data only}*

Pastorino U, Soresi E, Clerici M, Chiesa G, Belloni PA, Ongari M, et al. Lung cancer chemoprevention with retinol palmitate. Preliminary data from a randomized trial on stage 1a non small-cell lung cancer. *Acta Oncologica* 1988;**27**(6b):773-82.

Pemp 2010 *{published data only}*

Pemp B, Polska E, Karl K, Lasta M, Minichmayr A, Garhofer G, et al. Effects of antioxidants (AREDS medication) on ocular blood flow and endothelial function in an endotoxin-induced model of oxidative stress in humans. *Investigative Ophthalmology and Visual Science* 2010;**51**(1):2-6.

Peng 1993 *{published data only}*

Peng YM, Peng YS, Lin Y, Moon T, Baier M. Micronutrient concentrations in paired skin and plasma of patients with actinic keratoses: effect of prolonged retinol

Copyright © 2017 The Cochrane Collaboration. Published by John Wiley & Sons, Ltd.

supplementation. *Cancer Epidemiology, Biomarkers and Prevention* 1993;**2**(2):145-50.

PPP 1996 *{published data only}*

Primary prevention project (PPP) in the Mario Negri Institute in Milan. *Ricerca E Pratica* 1996;**12**:175-218.

PPSG 1994 *{published data only}*

Greenberg ER, Baron JA, Tosteson TD, Freeman DH Jr, Beck GJ, Bond JH, et al. A clinical trial of antioxidant vitamins to prevent colorectal adenoma. Polyp Prevention Study Group. *New England Journal of Medicine* 1994;**331**(3):141-7.

Prasad 1995 *{published data only}*

Prasad MP, Mukundan MA, Krishnaswamy K. Micronuclei and carcinogen DNA adducts as intermediate end points in nutrient intervention trial of precancerous lesions in the oral cavity. *European Journal of Cancer Part B Oral Oncology* 1995;**31B**(3):155-9.

REACT 1995 *{published data only}*

Chylack LT Jr, Wolfe JK, Friend J, Tung W, Singer DM, Brown NP, et al. Validation of methods for the assessment of cataract progression in the Roche European-American anticataract trial (REACT). *Ophthalmic Epidemiology* 1995;**2**(2):59-75.

Recchia 1995 *{published data only}*

Recchia F, Sica G, de Filippis S, Discepoli S, Rea S, Torchio P, et al. Interferon-beta, retinoids, and tamoxifen in the treatment of metastatic breast cancer: a phase II study. *Journal of Interferon and Cytokine Research* 1995;**15**(7):605-10.

Rein 2007 *{published data only}*

Rein DB, Saaddine JB, Wittenborn JS, Wirth KE, Hoerger TJ, Narayan KM, et al. Cost-effectiveness of vitamin therapy for age-related macular degeneration. *Ophthalmology* 2007;**114**(7):1319-26.

Ret Pig 1993 *{published data only}*

Berson EL, Rosner B, Sandberg MA, Hayes KC, Nicholson BW, Weigel DiFranco C, et al. Randomized trial of vitamin A and vitamin E supplementation for retinitis pigmentosa. *Archives of Ophthalmology* 1993;**111**(6):761-72.

Norton EWD, Marmor MF, Clowes DD, Gamel JW, Barr CC, Fielder AR, et al. A randomized trial of vitamin A and vitamin E supplementation for retinitis pigmentosa (2). *Archives of Ophthalmology* 1993;**111**(11):1460-6.

Rodriguez-Carmona 2006 *{published data only}*

Rodriguez-Carmona M, Kvansakul J, Harlow JA, Köpcke W, Schalch W, Barbur JL. The effects of supplementation with lutein and/or zeaxanthin on human macular pigment density and colour vision. *Ophthalmic and Physiological Optics* 2006;**26**(2):137-47.

SCPS 1989 *{published data only}*

Greenberg ER, Baron JA, Stevens MM, Stukel TA, Mandel JS, Spencer SK, et al. The Skin cancer prevention study: design of a clinical trial of beta-carotene among persons at high risk for nonmelanoma skin cancer. *Controlled Clinical Trials* 1989;**10**(2):153-66.

Greenberg ER, Baron JA, Stukel TA, Stevens MM, Mandel JS, Spencer SK, et al. A clinical trial of beta carotene to prevent basal-cell and squamous-cell cancers of the skin. *New England Journal of Medicine* 1990;**323**(12):789-95.

SECURE 1996 *{published data only}*

Lonn EM, Yusuf S, Doris CI, Sabine MJ, Dzavik V, Hutchison K, et al. Study design and baseline characteristics of the study to evaluate carotid ultrasound changes in patients treated with ramipril and vitamin E: SECURE. *American Journal of Cardiology* 1996;**78**(8):914-9.

Shandong 1998 *{published data only}*

Gail MH, You WC, Chang YS, Zhang L, Blot WJ, Brown LM, et al. Factorial trial of three interventions to reduce the progression of precancerous gastric lesions in Shandong, China: design issues and initial data. *Controlled Clinical Trials* 1998;**19**(4):352-69.

Sharma 1989 *{published data only}*

Sharma YR, Vajpayee RB, Bhatnagar R, Mohan M, Azad RV, Kumar M, et al. Systemic aspirin and systemic vitamin E in senile cataracts: cataract V. *Indian Journal of Ophthalmology* 1989;**37**(3):134-41.

Steiner 1995 *{published data only}*

Steiner M, Glantz M, Lekos A. Vitamin E plus aspirin compared with aspirin alone in patients with transient ischemic attacks. *American Journal of Clinical Nutrition* 1995;**62**(6 Suppl):1381S-4S.

SUVIMAX 1997 *{published data only}*

Girodon F, Blache D, Monget AL, Lombart M, Brunet Lecompte P, Arnaud J, et al. Effect of a two-year supplementation with low doses of antioxidant vitamins and/or minerals in elderly subjects on levels of nutrients and antioxidant defence parameters. *Journal of the American College of Nutrition* 1997;**16**(4):357-65.

Hercberg S, Galan P, Preziosi P, Roussel AM, Arnaud J, Richard MJ, et al. Background and rationale behind the SU.VI.MAX Study, a prevention trial using nutritional doses of a combination of antioxidant vitamins and minerals to reduce cardiovascular diseases and cancers. SUpplementation en VItamines et Mineraux AntioXydants Study. *International Journal for Vitamin and Nutrition Research* 1998;**68**(1):3-20.

SWSCPSG 1997 *{published data only}*

Levine N, Moon TE, Cartmel B, Bangert JL, Rodney S, Dong Q, et al. Trial of retinol and isotretinoin in skin cancer prevention: a randomized, double-blind, controlled trial. Southwest Skin Cancer Prevention Study Group. *Cancer Epidemiology, Biomarkers and Prevention* 1997;**6**(11):957-61.

Takamatsu 1995 *{published data only}*

Takamatsu S, Takamatsu M, Satoh K, Imaizumi T, Yoshida H, Hiramoto M, et al. Effects on health of dietary supplementation with 100 mg d-alpha-tocopheryl acetate, daily for 6 years. *Journal of International Medical Research* 1995;**23**(5):342-57.

Copyright © 2017 The Cochrane Collaboration. Published by John Wiley & Sons, Ltd.

Tomeo 1995 *{published data only}*

Tomeo AC, Geller M, Watkins TR, Gapor A, Bierenbaum ML. Antioxidant effects of tocotrienols in patients with hyperlipidemia and carotid stenosis. *Lipids* 1995;**30**(12):1179-83.

Tsubono 1997 *{published data only}*

Tsubono Y, Okubo S, Hayashi M, Kakizoe T, Tsugane S. A randomized controlled trial for chemoprevention of gastric cancer in high-risk Japanese population; study design, feasibility and protocol modification. *Japanese Journal of Cancer Research* 1997;**88**(4):344-9.

Wahlqvist 1994 *{published data only}*

Wahlqvist ML, Wattanapenpaiboon N, Macrae FA, Lambert JR, MacLennan R, Hsu Hage BH. Changes in serum carotenoids in subjects with colorectal adenomas after 24 mo of beta-carotene supplementation. Australian Polyp Prevention Project Investigators. *American Journal of Clinical Nutrition* 1994;**60**(6):936-43.

Wong 2010 *{published data only}*

Wong WT, Kam W, Cunningham D, Harrington M, Hammel K, Meyerle CB, et al. Treatment of geographic atrophy by the topical administration of OT-551: results of a phase II clinical trial. *Investigative Ophthalmology and Visual Science* 2010;**51**(12):6131-9.

Wright 1985 *{published data only}*

Wright JP, Mee AS, Parfitt A, Marks IN, Burns DG, Sherman M, et al. Vitamin A therapy in patients with Crohn's disease. *Gastroenterology* 1985;**88**(2):512-4.

Yu 1991 *{published data only}*

Yu SY, Zhu YJ, Li WG, Huang QS, Huang CZ, Zhang QN, et al. A preliminary report on the intervention trials of primary liver cancer in high-risk populations with nutritional supplementation of selenium in China. *Biological Trace Element Research* 1991;**29**(3):289-94.

YUNNAN 1990 *{published data only}*

Yu SY, Mao BL, Xiao P, Yu WP, Wang YL, Huang CZ, et al. Intervention trial with selenium for the prevention of lung cancer among tin miners in Yunnan, China. A pilot study. *Biological Trace Element Research* 1990;**24**(2):105-8.

Zaridze 1993 *{published data only}*

Zaridze D, Evstifeeva T, Boyle P. Chemoprevention of oral leukoplakia and chronic esophagitis in an area of high incidence of oral and esophageal cancer. *Annals of Epidemiology* 1993;**3**(3):225-34.

References to ongoing studies

NCT01269697 (LIMPIA) *{published data only}*

NCT01269697. Lutein influence on macula of persons issued from AMD parents (LIMPIA). clinicaltrials.gov/ct2/show/NCT01269697 (accessed 6 April 2012).

SELECT *{published data only}*

Lippman SM, Klein EA, Goodman PJ, Lucia MS, Thompson IM, Ford LG, et al. Effect of selenium and vitamin E on risk of prostate cancer and other cancers: the Selenium and Vitamin E cancer prevention trial (SELECT). *JAMA* 2009;**301**(1):39-51.

WACS *{unpublished data only}*

Manson JE, Gaziano JM, Spelsberg A, Ridker PM, Cook NR, Buring JE, et al. A secondary prevention trial of antioxidant vitamins and cardiovascular disease in women. Rationale, design, and methods. The WACS Research Group. *Annals of Epidemiology* 1995;**5**(4):261-9.

Additional references

AREDS 2001a

Age-related Eye Disease Study Research Group. A randomized, placebo-controlled, clinical trial of high-dose supplementation with vitamins C and E, beta carotene, and zinc for age-related macular degeneration and vision loss: AREDS report no. 8. *Archives of Ophthalmology* 2001;**119**(10):1417-36.

ATBC 1994

The Alpha-tocopherol, Beta-carotene Cancer Prevention Study Group. The effect of vitamin E and beta carotene on the incidence of lung cancer and other cancers in male smokers. *New England Journal of Medicine* 1994;**330**(15):1029-35.

Bjelakovic 2012

Bjelakovic G, Nikolova D, Gluud LL, Simonetti RG, Gluud C. Antioxidant supplements for prevention of mortality in healthy participants and patients with various diseases. *Cochrane Database of Systematic Reviews* 2012, Issue 3. [DOI: 10.1002/14651858.CD007176.pub2]

Bunce 2006

Bunce C, Wormald R. Leading causes of certification for blindness and partial sight in England & Wales. *BMC Public Health* 2006;**6**(1):58.

Chong 2007

Chong EW-T, Wong TY, Kreis AJ, Simpson JA, Guymer RH. Dietary antioxidants and primary prevention of age-related macular degeneration: a systematic review and meta-analysis. *BMJ* 2007;**335**(7623):755.

Christen 1996

Christen WG, Glynn RJ, Hennekens CH. Antioxidants and age-related eye disease. Current and future perspectives. *Annals of Epidemiology* 1996;**6**(1):60-6.

Covidence 2015 [Computer program]

Veritas Health Innovation. Covidence systematic review software. Version accessed prior to 27 October 2016. Melbourne: Veritas Health Innovation, 2015.

Evans 2001

Evans JR. Risk factors for age-related macular degeneration. *Progress in Retinal Eye Research* 2001;**20**:227-53.

Copyright © 2017 The Cochrane Collaboration. Published by John Wiley & Sons, Ltd.

Evans 2017

Evans JR, Lawrenson JG. Antioxidant vitamin and mineral supplements for slowing the progression of age-related macular degeneration. *Cochrane Database of Systematic Reviews* 2017, Issue 7. [DOI: 10.1002/14651858.CD000254.pub4]

Gaziano 2009

Gaziano JM, Glynn RJ, Christen WG, Kurth T, Belanger C, MacFadyen J, et al. Vitamins E and C in the prevention of prostate and total cancer in men: the Physicians' health study II randomized controlled trial. *JAMA* 2009;**301**:52-62.

Gaziano 2012

Gaziano JM, Sesso HD, Christen WG, Bubes V, Smith JP, MacFadyen J, et al. Multivitamins in the prevention of cancer in men: the Physicians' health study II randomized controlled trial. *JAMA* 2012;**308**:1871-80.

Glanville 2006

Glanville JM, Lefebvre C, Miles JN, Camosso-Stefinovic J. How to identify randomized controlled trials in MEDLINE: ten years on. *Journal of the Medical Library Association* 2006;**94**(2):130-6.

GRADEpro 2014 [Computer program]

GRADE Working Group, McMaster University. GRADEpro. Version accessed 22 June 2016. Hamilton (ON): GRADE Working Group, McMaster University, 2014.

Higgins 2011

Higgins JP, Altman DG, Sterne JAC editor(s). Chapter 8: Assessing risk of bias in included studies. In: Higgins JP, Green S editor(s). Cochrane Handbook for Systematic Reviews of Interventions Version 5.1.0 (updated March 2011). The Cochrane Collaboration, 2011. Available from handbook.cochrane.org.

Huang 2006

Huang H-Y, Caballero B, Chang S, Alberg AJ, Semba RD, Schneyer CR, et al. The efficacy and safety of multivitamin and mineral supplement use to prevent cancer and chronic disease in adults: a systematic review for a National Institutes of Health State-of-the-Science Conference. *Annals of Internal Medicine* 2006;**145**(5):372-85.

Klein 1993

Klein R, Klein BEK, Linton KL, DeMets DL. The Beaver dam eye study: the relation of age-related maculopathy to smoking. *American Journal of Epidemiology* 1993;**137**:190-200.

Lee 2005

Lee IM, Cook NR, Gaziano JM, Gordon D, Ridker PM, Manson JE, et al. Vitamin E in the primary prevention of cardiovascular disease and cancer: the Women's health study: a randomized controlled trial. *JAMA* 2005;**294**:56-65.

NICE 2016

NICE. Macular degeneration - NICE guidelines. www.nice.org.uk/guidance/indevelopment/gid-cgwave0658/ (accessed 28 March 2016).

Omenn 1996

Omenn GS, Goodman GE, Thornquist MD, Balmes J, Cullen MR, Glass A, et al. Effects of a combination of beta carotene and vitamin A on lung cancer and cardiovascular disease. *New England Journal of Medicine* 1996;**334**(18):1189-90.

Owen 2012

Owen CG, Jarrar Z, Wormald R, Cook DG, Fletcher AE, Rudnicka AR. The estimated prevalence and incidence of late stage age related macular degeneration in the UK. *British Journal of Ophthalmology* 2012;**96**(5):752-6.

Review Manager 5 2014 [Computer program]

Nordic Cochrane Centre, The Cochrane Collaboration. Review Manager 5 (RevMan 5). Version 5.3. Copenhagen: Nordic Cochrane Centre, The Cochrane Collaboration, 2014.

Schünemann 2011

Schünemann HJ, Oxman AD, Vist GE, Higgins JP, Deeks JJ, Glasziou P, et al. Chapter 12: Interpreting results and drawing conclusions. In: Higgins JP, Green S editor(s). Cochrane Handbook for Systematic Reviews of Interventions Version 5.1.0 (updated March 2011). The Cochrane Collaboration, 2011. Available from handbook.cochrane.org.

Sesso 2008

Sesso HD, Buring JE, Christen WG, Kurth T, Belanger C, MacFadyen J, et al. Vitamins E and C in the prevention of cardiovascular disease in men: the Physicians' health study II randomized controlled trial. *JAMA* 2008;**300**:2123-33.

Zampatti 2014

Zampatti S, Ricci F, Cusumano A, Marsella L T, Novelli G, Giardina E. Review of nutrient actions on age-related macular degeneration. *Nutr Res* 2014;**34**:95-105.

References to other published versions of this review

Evans 1999

Evans JR, Henshaw K. Antioxidant vitamin and mineral supplements for preventing age-related macular degeneration. *Cochrane Database of Systematic Reviews* 1994, Issue 4. [DOI: 10.1002/14651858.CD000253]

Evans 2008

Evans JR, Henshaw KS. Antioxidant vitamin and mineral supplements for preventing age-related macular degeneration. *Cochrane Database of Systematic Reviews* 2008, Issue 1. [DOI: 10.1002/14651858.CD000253.pub2]

Evans 2012

Evans JR, Lawrenson JG. Antioxidant vitamin and mineral supplements for preventing age-related macular degeneration. *Cochrane Database of Systematic Reviews* 2012, Issue 6. [DOI: 10.1002/14651858.CD000253.pub3]

* Indicates the major publication for the study

Copyright © 2017 The Cochrane Collaboration. Published by John Wiley & Sons, Ltd.

CHARACTERISTICS OF STUDIES

Characteristics of included studies *[ordered by study ID]*

ATBC 1998

Methods	Method of allocation: random. Sponsor provided coded capsules.<br><br>Masking: participant: yes; provider: yes; outcome: yes<br><br>Exclusions after randomisation: no<br><br>Losses to follow-up: 31%. Random sample for maculopathy study: 9%.<br>2 x 2 factorial design. Maculopathy add-on random sample in 2 regions.
Participants	Country: Finland<br><br>Number of participants randomised: 29,133. Random sample of 1035 selected for maculopathy study.<br><br>Age: 50 to 69 years in 1984. Maculopathy study 1992-3 in people aged 65 plus.<br><br>Sex: male<br><br>Inclusion criteria: 5 or more cigarettes daily<br><br>Exclusion criteria: history of cancer or serious disease limiting ability to participate; those taking supplements vitamin E, A, or beta-carotene in excess of predefined doses; those treated with anticoagulants
Interventions	Intervention:<br><br>• alpha-tocopherol (50 mg/day) N = 237<br>• beta-carotene (20 mg/day) N = 234<br>• alpha-tocopherol (50 mg/day) and beta-carotene (20 mg/day) N = 257<br><br>Comparator:<br><br>• placebo N = 213<br><br>Duration: 5 to 8 years (median 6.1)
Outcomes	AMD: 4 grades:<br>Grade I: dry maculopathy with hard drusen, pigmentary changes, or both<br>Grade II: soft macular drusen<br>Grade III: disciform degeneration<br>Grade IV: geographic atrophy<br><br>Quote "A person was considered to have ARM if he had a class I or higher change in either eye, and severity was classified according to the worst eye."
Notes	Compliance with treatment excellent; 4/5 active participants took more than 95% of scheduled capsules. Drop-out rate and compliance similar between all 4 groups.<br><br>Funding source: Quote "This study was supported by the Juho Vainio Foundation, Helsinki, Finland. The ATBC study was supported by Public Health Service Contract N01-CN-45165 from the Division of Cancer Prevention and Control, National Cancer Institute of the United States."<br><br>Declarations of interest: NR<br><br>Date study conducted: Quote "The ophthalmological examination took place during their final follow-up trial visit between December 1992 and March 1993"<br><br>Trial id: NCT00342992

Copyright © 2017 The Cochrane Collaboration. Published by John Wiley & Sons, Ltd.

ATBC 1998 *(Continued)*

Risk of bias

Bias	Authors' judgement	Support for judgement
Random sequence generation (selection bias)	Low risk	Quote "The participants were randomly assigned to one of four treatment groups: AT alone, AT and BC, BC alone, or placebo in a complete 2 x 2 factorial design" and "Randomization was performed in blocks of eight within each of the study areas."
Allocation concealment (selection bias)	Low risk	Quote "A coded reserve supply of capsule packs..."<br><br>Not clearly stated that allocation concealed, but the study was described as being "double-blind"
Blinding of participants and personnel (performance bias)<br>All outcomes	Low risk	Judgement comment: placebo-controlled study.
Blinding of outcome assessment (detection bias)<br>AMD	Low risk	Quote "The retinal specialist [...] examined six photographs (three per eye) of each participant without knowledge of the subject's treatment group"
Incomplete outcome data (attrition bias)<br>All outcomes	Low risk	Quote "A total of 941 persons participated (91%) and non-participation rates were similar across the intervention groups."
Selective reporting (reporting bias)	Unclear risk	Visual acuity measured but not reported, but as the main results for AMD showed no difference between groups, it is not clear whether this was an example of selective reporting or whether, in fact, the investigators considered that visual acuity in this age group might be attributed to a variety of causes, and therefore, was not a relevant outcome.

PHS I 2007

Methods	Method of allocation: coded tablets<br><br>Masking: participant: yes; provider: yes; outcome: yes<br><br>99% follow-up<br><br>2 x 2 factorial design.
Participants	Country: USA<br><br>Number randomised: originally 22,071 men were randomised:<br>21,142 participants were followed up for at least 7 years and provided information on diagnoses of AMD made during the first 7 years of the trial<br><br>Age: 40 to 84 years in 1982<br><br>Sex: male<br><br>Inclusion criteria: physician aged 40 to 84 years in 1982 with no history of cancer, myocardial infarction, stroke, or transient cerebral ischaemia<br><br>Exclusion criteria: personal history of cardiovascular disease or cancer; contraindications or current use of study medication;

Copyright © 2017 The Cochrane Collaboration. Published by John Wiley & Sons, Ltd.

PHS I 2007 *(Continued)*

Interventions	Intervention:<br>• beta-carotene (50 mg/alternate days) N = 11,036 randomised, 10,585 followed up<br>Comparator:<br>• beta-carotene placebo N = 11,035 randomised, 10,557 followed up<br>There was also an aspirin arm (2 x 2 factorial arm), which was terminated early (January 1988)<br>Mean duration 12 years range (range 11.6 to 14.2 years)
Outcomes	Self report of AMD followed by medical record review and questionnaire to relevant ophthalmologist<br>Primary endpoint: visually significant AMD, defined as a self-report confirmed by medical record evidence of an initial diagnosis after randomisation, but before 31 December 1995, with a reduction in best-corrected visual acuity to 20/30 or worse attributable to AMD<br>Secondary endpoints: AMD with or without vision loss, composed of all incident cases; Advanced AMD, encompassed of cases of visually significant AMD with pathological signs of disciform scar, RPE detachment, geographic atrophy, or subretinal neovascular membrane<br>Quote "Individuals, rather than eyes, were the unit of analysis because eyes were not examined independently, and participants were classified according to the status of the worse eye as defined by disease severity. When the worse eye was excluded because of visual acuity loss attributed to other ocular abnormalities the fellow eye was considered for classification."
Notes	Funding source: Quote "Supported by research grants HL 26490, HL 34595, CA 34944, CA 40360, and EY 06633 from the National Institutes of Health"<br>Declarations of interest: NR<br>Date study conducted: August 1985 (from clinicaltrials.gov) to December 1995<br>Trial id: NCT00000500

Risk of bias

Bias	Authors' judgement	Support for judgement
Random sequence generation (selection bias)	Low risk	Quote "The PHS I was a randomised, double-masked, placebo controlled trial..."<br>"A total of 22,071 physicians were then randomised according to a two-by-two factorial design, with use of a computer-generated list of random numbers..."
Allocation concealment (selection bias)	Low risk	Quote "The PHS I was a randomised, double-masked, placebo controlled trial..."<br>Judgement Comment: Although this aspect of the trial was not well described, the placebo control was described (placebo and supplement identical appearance and packaging) and the study was described as double-blind
Blinding of participants and personnel (performance bias)<br>All outcomes	Low risk	Quote "The PHS I was a randomised, double-masked, placebo controlled trial..."<br>Judgement Comment: Although this aspect of the trial was not well described, the placebo control was described (placebo and supplement identical appearance and packaging) and the study was described as double-blind
Blinding of outcome assessment (detection bias)	Low risk	Quote "The PHS I was a randomised, double-masked, placebo controlled trial..."

Copyright © 2017 The Cochrane Collaboration. Published by John Wiley & Sons, Ltd.

PHS I 2007 *(Continued)*

AMD		Judgement Comment: Although this aspect of the trial was not well described, the placebo control was described and the study was described as double-blind. Diagnosis of AMD by self-report based on health questionnaire (confirmed by ophthalmologist or optometrist). Patients and researchers unaware of intervention.
Incomplete outcome data (attrition bias)<br>All outcomes	Low risk	Quote "At the end of 11 years of follow-up (the last year completed for all participants), 99.2% were still providing information on morbidity, and the follow-up for mortality was 99.9% complete. Eighty percent of participants in the beta-carotene group and in the placebo group were still taking the study pills, with a mean compliance among pill takers of more than 97%. Therefore, even after 11 years, 78% of the study pills assigned in the beta-carotene group were reported as still being taken. In the placebo group, 6% of participants reported taking supplemental beta carotene or vitamin A."
Selective reporting (reporting bias)	Low risk	Judgement comment: reported AMD outcomes as expected

PHS II 2012

Methods	Method of allocation: coded tablets<br><br>Masking: participant: yes; provider: yes; outcome: yes<br><br>95% follow-up<br><br>2 x 2 x 2 x 2 factorial design.
Participants	Country: USA<br><br>Number randomised: 14,236 with no diagnosis of AMD at baseline according to vitamin C/E paper; 14,233 with no diagnosis of AMD at baseline according to multivitamin paper.<br><br>Average age: 64 years<br><br>Sex: male<br><br>Inclusion criteria: US male physicians; 50 years and older; participants in PHS I and new physician participants; willing to forego use of supplements for new trial; for new participants, do not report personal history of cancer (except non-melanoma skin cancer). CVD, current liver disease, current renal disease, peptic ulcer or gout. Compliance with pill-taking regimen in run-in period.<br><br>Exclusion criteria:History of cirrhosis; active liver disease in past six months; participants reporting cataract or AMD at baseline.
Interventions	Intervention:<br><br>• vitamin E (400 IU/alternate days) N = 7112<br>• vitamin C (500 mg/day) N = 7149<br>• multivitamin (Centrum silver: zinc 15 mg, vitamin E 45 IU, vitamin C 60 mg, beta-carotene 5000 IU, vitamin A - 20% as beta carotene, folic acid 2.5 mg, vitamin B6 50 mg, vitamin B12 1 mg/ day) N = 7111<br><br>Comparator:<br><br>• separate placebos for each type of vitamin: vitamin E placebo N = 7124, vitamin C placebo N = 7087, multivitamin placebo N = 7122<br><br>Alternate day beta-carotene (50 mg) component terminated in March 2003.<br><br>Lutein (added to Centrum Silver during course of study (250 µg) and doses of other nutrients changed

Copyright © 2017 The Cochrane Collaboration. Published by John Wiley & Sons, Ltd.

PHS II 2012 *(Continued)*

	Follow-up: the multivitamin component had a longer duration.<br><br>"An average of 8 years of treatment and follow-up" for vitamin E and vitamin C<br><br>Median duration of treatment for multivitamin analyses 11.2 years, IQR 10.7 to 13.3
Outcomes	Age-related macular degeneration: reported diagnosis followed up by contact with treating ophthalmologist/optometrist<br><br>Quote "We considered individuals, rather than eyes, as the unit of analysis and we classified individuals according to the status of the worse eye as defined by disease severity. When the worse eye was excluded because of visual acuity loss attributed to other ocular abnormalities, the fellow eye was considered for classification."
Notes	Funding source: Grants from National Eye Institute, National Institute on Ageing and the National Institutes of Health. BASF and DSM provided study agents and packaging.<br><br>Declarations of interest: "The authors have no proprietary or commercial interest in any of the materials discussed in this article."<br><br>Date study conducted: July 1997 to June 2011 (from clinicaltrials.gov)<br><br>Trial id: NCT00270647

Risk of bias

Bias	Authors' judgement	Support for judgement
Random sequence generation (selection bias)	Low risk	Quote "Randomisation to the other agents, using a computer generated list of random numbers, will be stratified according to age"
Allocation concealment (selection bias)	Low risk	Judgement comment: central randomisation
Blinding of participants and personnel (performance bias)<br>All outcomes	Low risk	Quote "The Physicians' Health Study (PHS II) was a randomised, double-blind, placebo-controlled, factorial trial evaluating a daily multivitamin (Centrum Silver), alternate day vitamin E (400 IU synthetic α-tocopherol), and daily vitamin C (500 mg synthetic ascorbic acid) in the prevention of cancer and CVD among 14,641 male physicians aged 50 years and older."
Blinding of outcome assessment (detection bias)<br>AMD	Low risk	Quote "Random misclassification was reduced by the use of medical records to confirm the participant reports. Non-random misclassification was unlikely since medical records were reviewed by an investigator (WGC) masked to treatment assignment, and study participants and treating ophthalmologists and optometrists were similarly unaware of treatment assignment."
Incomplete outcome data (attrition bias)<br>All outcomes	Low risk	Quote "Morbidity and mortality follow-up were extremely high, at 95.3% and 97.9%, respectively."
Selective reporting (reporting bias)	Low risk	Judgement comment: reported AMD outcomes as expected

VECAT 2002

Methods	Method of allocation: coded bottles<br><br>Masking: participant: yes; provider: yes; outcome: yes

Copyright © 2017 The Cochrane Collaboration. Published by John Wiley & Sons, Ltd.

VECAT 2002 *(Continued)*

	Losses to follow-up: not known
Participants	Country: Australia<br><br>Number of participants randomised: 1204<br><br>Eyes: worse eye used as the study eye<br><br>Age: 55 to 80 years, mean 66<br><br>Sex: 56% female<br><br>Inclusion criteria: lens and retina of at least one eye available for documentation<br><br>Exclusion criteria: previous cataract surgery or advanced cataract in both eyes; steroid or anticoagulation use; serious disease; regular use or sensitivity to vitamin E
Interventions	Intervention:<br><br>• vitamin E (500 IU/day) natural vitamin E in soybean oil medium N = 595 randomised, 587 followed up<br><br>Comparator:<br><br>• placebo identical in sight, taste and smell N = 598 randomised, 592 followed up<br><br>Duration: 4 years
Outcomes	2 m logMAR visual acuity; clinical examination; colour stereoscopic fundus photographs graded using International Grading Scheme<br><br>Quote "Participants were categorised by their worse eye."
Notes	Funding source: Quote "The VECAT study was funded in part by grants from the National Health and Medical Research Council, the Jack Brockhoff Foundation, the Eirene Lucas Foundation, the Stoicesco Foundation, the Carleton Family Charitable Trust, JeHope Knell Trust Fund, Smith and Nephew, Australia, and Henkel Australia".<br><br>Declarations of interest: none declared<br><br>Date study conducted: January 1995 to April 1996<br><br>Trial id: NR

Risk of bias

Bias	Authors' judgement	Support for judgement
Random sequence generation (selection bias)	Low risk	Quote "This random allocation was performed by using a “permuted blocks” allocation scheme"
Allocation concealment (selection bias)	Low risk	Quote "Study numbers were allocated sequentially by the study coordinator as participants were enrolled in the study. Participants were then randomly allocated to treatment group. The allocation list was stored at a remote site."
Blinding of participants and personnel (performance bias)<br>All outcomes	Low risk	Quote "Participants randomly received either 500 IU natural vitamin E (335 mg dá tocopherol) in a soybean oil suspension encapsulated in gelatin or a matched placebo capsule containing only the soybean oil." [...] "Bulk medications were dispensed into labelled jars by a person not involved in the study. Vitamin E and placebo were dispensed on different days to avoid confusion. Identical containers were used. The jars were packed in numerical order and then dispensed by study personnel. Vitamin E and placebo capsules were of identical appearance and taste. Neither study staff nor examiners or partici-

Copyright © 2017 The Cochrane Collaboration. Published by John Wiley & Sons, Ltd.

VECAT 2002 *(Continued)*

		pants were aware of the treatment allocation, although all knew that participants would be randomly assigned to receive either vitamin E or placebo."
Blinding of outcome assessment (detection bias)<br>AMD	Low risk	Quote "Neither study staff nor examiners or participants were aware of the treatment allocation, although all knew that participants would be randomly assigned to receive either vitamin E or placebo." "At the end of the study we re-assessed the initial and final photographs for any change with a “side by side” comparison in a masked and randomised fashion".
Incomplete outcome data (attrition bias)<br>All outcomes	Low risk	Quote "From the 1906 people who were screened by telephone, 1289 (69%) were examined and 1204 (93%) of these were enrolled and randomised. We excluded 11 participants after randomisation as they were outside the required age range at enrolment."<br><br>"In the vitamin E group, eight people were excluded from final data analysis: six developed diabetic retinopathy, one had myopic degeneration, and one had missing data. Six people were excluded from the placebo group: two developed adult vitelliform macular degeneration and four had missing data."<br><br>Figure 3: 1204 randomised, 11 excluded after randomisation, 14 excluded from analysis: 8/595 vitamin E group and 6/598 placebo group.
Selective reporting (reporting bias)	Low risk	Judgement comment: AMD incidence and progression reported but no difference between groups; visual acuity not reported but "Analysis of best corrected visual acuity and visual function data showed no differences between the groups (data not shown)." Therefore, no evidence that outcomes with "better" results selectively reported.

WHS 2010

Methods	Method of allocation: random allocation of coded bottles<br><br>Masking: participant: yes; provider: yes; outcome: yes<br><br>Losses to follow-up: not known
Participants	Country: USA<br><br>Number of participants randomised: 39,876 women health professionals<br><br>Age: 45+<br><br>Sex: female<br><br>Inclusion/exclusion criteria: (a) Female; (b) aged 45 years or older; (c) postmenopausal or with no intention of becoming pregnant; (d) no reported personal history of cardiovascular disease, cancer (other than non-melanoma skin cancer), gout, peptic ulcer, chronic renal or liver disease, or other serious illness precluding participation; (e) no reported history of serious side effects to the study treatments; (f) not currently taking aspirin, aspirin containing medication, or nonsteroidal anti-inflammatory drugs (NSAIDs) more than 1 day per week or, if so doing, willing to forego use of these medications; (g) not currently taking individual supplements of vitamin E or beta-carotene more than 1 day per week; (h) not currently taking anticoagulants or corticosteroids
Interventions	Intervention:<br><br>• vitamin E (600 IU on alternate days) N = 19,697<br><br>Comparator:

Copyright © 2017 The Cochrane Collaboration. Published by John Wiley & Sons, Ltd.

WHS 2010 *(Continued)*

	• placebo N = 19,724
Outcomes	Self-report and review of medical records<br><br>Quote "Individuals, rather than eyes, were the unit of analysis because eyes were not examined independently, and participants were classified according to the status of the worse eye as defined by disease severity. When the worse eye was excluded because of visual acuity loss attributed to other ocular abnormalities, the fellow eye was considered for classification."
Notes	Funding source: Quote "Supported by research grants CA 47988, HL 43851, and EY 06633 from the National Institutes of Health, Bethesda, Md. Pills and packaging were provided by Bayer Healthcare and the Natural Source Vitamin E Association. Bayer Healthcare and the Natural Source Vitamin E Association had no role in the design and conduct of the study, in the collection, analysis, and interpretation of the data, or in the preparation, review, or approval of the manuscript."<br><br>Declarations of interest: Quote "The authors have no proprietary or commercial interest in any materials discussed in this article."<br><br>Date study conducted: August 1993 to March 2004<br><br>Trial id: NCT00000161

Risk of bias

**Bias**	**Authors' judgement**	**Support for judgement**
Random sequence generation (selection bias)	Low risk	Quote "The WHS was a randomised, double-blind, placebo-controlled, 2 x 2 factorial trial..." "Randomization used blocks of size 16 within 5-year age strata and took place from April 30, 1993, through January 24, 1996."
Allocation concealment (selection bias)	Low risk	Quote "The WHS was a randomised, double-blind, placebo-controlled, 2 x 2 factorial trial..."
Blinding of participants and personnel (performance bias)<br>All outcomes	Low risk	Quote "Study medications and end-point ascertainment were continued in a blinded fashion through the scheduled end of the trial (March 31, 2004)." "Pill taking and end point ascertainment were continued in blinded fashion through the scheduled end of the trial on March 31, 2004. Morbidity and mortality follow-up were 97.2% and 99.4% complete, respectively."
Blinding of outcome assessment (detection bias)<br>AMD	Low risk	Quote "Study medications and end-point ascertainment were continued in a blinded fashion through the scheduled end of the trial (March 31, 2004)." "Pill taking and end point ascertainment were continued in blinded fashion through the scheduled end of the trial on March 31, 2004. Morbidity and mortality follow-up were 97.2% and 99.4% complete, respectively."
Incomplete outcome data (attrition bias)<br>All outcomes	Low risk	Quote "Compliance (defined as taking at least two thirds of the study capsules) was 78.9% at 5 years and 71.6% at 10 years, and averaged 75.8% throughout the trial."<br><br>"Pill taking and end point ascertainment were continued in blinded fashion through the scheduled end of the trial on March 31, 2004. Morbidity and mortality follow-up were 97.2% and 99.4% complete, respectively."<br><br>Follow-up balanced across treatment groups. See figure 1.
Selective reporting (reporting bias)	Low risk	No evidence of selective reporting. The outcome was limited to the study design - medical record review. Primary and secondary outcomes were apparently defined a priori and were reported.

AMD: age-related macular degeneration

Copyright © 2017 The Cochrane Collaboration. Published by John Wiley & Sons, Ltd.

ETDRS: Early Treatment Diabetic Retinopathy Study
IU: international units
RPE: retinal pigment epithelium

Characteristics of excluded studies *[ordered by study ID]*

Study	Reason for exclusion
ADSC 1997	No published data on AMD. No response from author.
Andrews 1969	No published data on AMD. Unable to contact author.
AREDS 2001	Age-related maculopathy outcomes for people without age-related maculopathy at baseline were not reported
AREDS2 2008	Participants had AMD
Benner 1994	No published data on AMD. No response from author.
Benton 1995	No data on AMD collected
Berrow 2016	No data on AMD collected
Blok 1997	No data on AMD collected
Bogden 1990	No published data on AMD. No response from author.
Bone 2010	Biological availability study only
Brewer 1997	No published data on AMD. No response from author.
Brown 1998	No published data on AMD. No response from author.
Bussey 1982	No published data on AMD. No response from author.
Caligiuri 1997	No published data on AMD. No response from author.
CARET 1996	No data on AMD collected
CARMIS 2011	Study on people with AMD (non-advanced AMD), therefore, not on prevention in healthy people
CCSG 1993	No published data on AMD. No response from author.
Chandra 1992	No published data on AMD. No response from author.
CHAOS 1996	No data on AMD collected
Clausen 1989	No published data on AMD. No response from author.
Constans 1996	No published data on AMD. No response from author.
Constantino 1988	No data on AMD collected
Cucinotta 1994	No published data on AMD. No response from author.
DATATOP 1989	No published data on AMD. Unable to contact author.

Copyright © 2017 The Cochrane Collaboration. Published by John Wiley & Sons, Ltd.

Study	Reason for exclusion
De Klerk 1998	No data on AMD collected
DeCosse 1989	No published data on AMD. No response from author
Dobson 1984	No data on AMD collected
ECP-IM 1995	No published data on AMD. No response from author.
EUROSCAN 1994	No published data on AMD. No response from author.
Fairley 1996	No published data on AMD. No response from author.
Falsani 2010	Participants had early AMD
Fontham 1995	No data on AMD collected
Galan 1997	No published data on AMD. No response from author.
Garawal 1995	No published data on AMD. No response from author.
GISSI 1993	No published data on AMD. No response from author.
HOPE 1996	No data on AMD collected
Johnson 1997	No published data on AMD. No response from author.
Jyothirmayi 1996	No published data on AMD. No response from author.
Kuklinski 1994	No published data on AMD. No response from author.
Kvansakul 2006	No AMD outcomes
Leng 1997	No published data on AMD. Unable to contact author.
Li 1992	No published data on AMD. No response from author.
LINXIAN 1993	No published data on AMD. No response from author.
Mayne 1998	No data on AMD collected
McKeown 1988	No data on AMD collected
Meyskens 1994	No published data on AMD. No response from author.
Munoz 1987	No published data on AMD. No response from author.
Munoz 1996	No published data on AMD. No response from author.
Nambour 1995	No follow-up data on AMD collected
NCT00000161 (WAFACS)	Reported on folic acid, pyridoxine and cyanocobalamin combination treatment
NCT00718653	Study of macular pigment only
NCT00893724	Study on people with AMD

Copyright © 2017 The Cochrane Collaboration. Published by John Wiley & Sons, Ltd.

Study	Reason for exclusion
NCT01208948	No AMD outcomes
Newsome, 2008	Study on people with AMD
NPCSG 1996	No published data on AMD. No response from author.
Pastorino 1991	No published data on AMD. No response from author.
Pemp 2010	Study of ocular blood flow and endothelial function only in model of oxidative stress in health volunteers
Peng 1993	No published data on AMD. No response from author.
PPP 1996	No published data on AMD. No response from author.
PPSG 1994	No data on AMD collected
Prasad 1995	No published data on AMD. No response from author.
REACT 1995	No published data on AMD. No response from author.
Recchia 1995	No published data on AMD. No response from author.
Rein 2007	Study not a randomised controlled trial
Ret Pig 1993	No published data on AMD. No response from author
Rodriguez-Carmona 2006	No AMD outcomes
SCPS 1989	No data on AMD collected
SECURE 1996	No published data on AMD. No response from author.
Shandong 1998	No data on AMD collected
Sharma 1989	No published data on AMD. No response from author.
Steiner 1995	No published data on AMD. No response from author.
SUVIMAX 1997	No published data on AMD. No response from author.
SWSCPSG 1997	No data on AMD collected
Takamatsu 1995	No published data on AMD. No response from author.
Tomeo 1995	No published data on AMD. No response from author.
Tsubono 1997	No data on AMD collected
Wahlqvist 1994	No data on AMD collected
Wong 2010	Participants had geographic atrophy
Wright 1985	No published data on AMD. No response from author.

Copyright © 2017 The Cochrane Collaboration. Published by John Wiley & Sons, Ltd.

Study	Reason for exclusion
Yu 1991	No published data on AMD. No response from author.
YUNNAN 1990	No published data on AMD. No response from author.
Zaridze 1993	No published data on AMD. No response from author.

AMD: age-related macular degeneration

Characteristics of ongoing studies *[ordered by study ID]*

NCT01269697 (LIMPIA)

Trial name or title	Lutein influence on macula of persons issued from AMD parents
Methods	Multicentre, double-masked, randomised clinical trial of supplementation with 'Nutrof Total' (lutein and zeaxanthin) versus placebo
Participants	People at high genetic risk for AMD because their parents had AMD. Age 40 to 70 years.
Interventions	Nutrof Total or placebo
Outcomes	Primary outcome measure:<br>Macular pigment density at 6 months after supplementation<br>Secondary outcome measures:<br>Best corrected visual acuity 12 months<br>Cognitive ability 12 months<br>Plasma fatty acids 12 months<br>Macular pigment density during supplementation and after stopping supplementation
Starting date	-
Contact information	Jean-Francois Korobelnik<br>jean-francois.korobelnik@chu-bordeaux.fr
Notes	clinicaltrials.gov/show/NCT01269697<br>The principal Investigator was contacted in March 2016 and confirmed that the study should be published in the next year.

SELECT

Trial name or title	Selenium and Vitamin E Cancer Prevention Trial (SELECT) for prostate cancer
Methods	This is a randomised, double-masked, multi-centre study. Participants are randomised to one of 4 prevention arms:<br>Arm I: participants receive 2 different oral placebos once daily<br>Arm II: participants receive oral selenium and oral placebo once daily

Copyright © 2017 The Cochrane Collaboration. Published by John Wiley & Sons, Ltd.

SELECT *(Continued)*

	Arm III: participants receive oral vitamin E and oral placebo once daily<br><br>Arm IV: participants receive oral selenium and oral vitamin E once daily.<br><br>Treatment continues for 7 to 12 years in the absence of unacceptable toxicity or diagnosis of prostate cancer<br><br>Quality of life is assessed at baseline and then at 1, 3, 5, and 7 years<br><br>Participants are followed annually
Participants	Healthy male volunteers. A total of 32,400 participants (8100 per prevention arm) will be accrued for this study within 5 years
Interventions	Dietary supplement: selenium<br>Dietary supplement: vitamin E
Outcomes	Primary outcome measures:<br><br>Effect on the clinical incidence of cancer<br>Effect on cancer-free survival, overall survival and serious cardiovascular events<br>Quality of life<br>Association of biological molecular markers with cancer risk<br>Relationship between effects on cancer risk and genetic factors<br>Effects in terms of intake of other nutrients, foods and dietary supplements<br>Effect of other dietary nutrients and dietary patterns on cancer risk<br>Effects on the reduction of Alzheimer's disease incidence<br>Reduction in the risk of AMD or cataract
Starting date	July 2001
Contact information	-
Notes	clinicaltrials.gov/show/NCT00006392<br><br>We contacted the principal investigator in March 2016; data collection and analysis of AMD outcomes is still ongoing.

WACS

Trial name or title	Women's Antioxidant Cardiovascular Study
Methods	-
Participants	8171 female health professionals aged 40+ with pre-existing cardiovascular disease (CVD) or high risk for developing CVD
Interventions	2 x 2 x 2 x 2 factorial design:<br><br>Vitamin E (600 IU on alternate days)<br><br>Vitamin C (500 mg daily)<br><br>Beta-carotene (5 mg on alternate days)<br><br>Combination of folate (800 mg daily), vitamin B6 (25 mg daily) and vitamin B12 (1 mg daily)
Outcomes	Self-report and review of medical records

Copyright © 2017 The Cochrane Collaboration. Published by John Wiley & Sons, Ltd.

WACS *(Continued)*

Starting date	1993
Contact information	-
Notes	clinicaltrials.gov/show/NCT00000541<br>We contacted the principal investigator in March 2016; no reply as yet.

AMD: age-related macular degeneration

DATA AND ANALYSES

Comparison 1. Vitamin E versus placebo

Outcome or subgroup title	No. of studies	No. of participants	Statistical method	Effect size
1 Any AMD	4	55614	Risk Ratio (M-H, Fixed, 95% CI)	0.97 [0.90, 1.06]
2 Late AMD (either neovascular AMD or geographic atrophy or both)	4	55614	Risk Ratio (M-H, Fixed, 95% CI)	1.22 [0.89, 1.67]
3 Neovascular AMD or geographic atrophy separately	1		Risk Ratio (M-H, Fixed, 95% CI)	Totals not selected
3.1 Neovascular AMD	1		Risk Ratio (M-H, Fixed, 95% CI)	0.0 [0.0, 0.0]
3.2 Geographic atrophy	1		Risk Ratio (M-H, Fixed, 95% CI)	0.0 [0.0, 0.0]

Analysis 1.1. Comparison 1 Vitamin E versus placebo, Outcome 1 Any AMD.

Study or subgroup	Vitamin E n/N	Placebo n/N	Risk Ratio M-H, Fixed, 95% CI	Weight	Risk Ratio M-H, Fixed, 95% CI
ATBC 1998	148/494	121/447		14.06%	1.11[0.9,1.36]
PHS II 2012	176/7112	187/7124		20.68%	0.94[0.77,1.16]
VECAT 2002	280/504	279/512		30.64%	1.02[0.91,1.14]
WHS 2010	280/19697	313/19724		34.62%	0.9[0.76,1.05]
**Total (95% CI)**	**27807**	**27807**		**100%**	**0.97[0.9,1.06]**
Total events: 884 (Vitamin E), 900 (Placebo)					
Heterogeneity: Tau²=0; Chi²=3.34, df=3(P=0.34); I²=10.08%					
Test for overall effect: Z=0.65(P=0.51)					

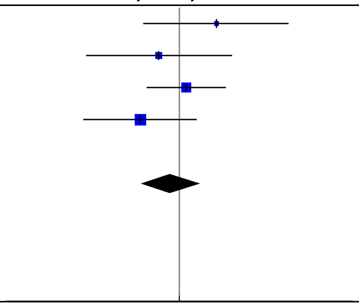

Copyright © 2017 The Cochrane Collaboration. Published by John Wiley & Sons, Ltd.

Analysis 1.2. Comparison 1 Vitamin E versus placebo, Outcome 2 Late AMD (either neovascular AMD or geographic atrophy or both).

Study or subgroup	Vitamin E n/N	Placebo n/N	Weight	Risk Ratio M-H, Fixed, 95% CI
ATBC 1998	11/494	3/447	4.43%	3.32[0.93,11.82]
PHS II 2012	42/7112	38/7124	53.42%	1.11[0.71,1.71]
VECAT 2002	5/504	4/512	5.58%	1.27[0.34,4.7]
WHS 2010	29/19697	26/19724	36.56%	1.12[0.66,1.9]
**Total (95% CI)**	**27807**	**27807**	**100%**	**1.22[0.89,1.67]**
Total events: 87 (Vitamin E), 71 (Placebo)				
Heterogeneity: Tau²=0; Chi²=2.68, df=3(P=0.44); I²=0%				
Test for overall effect: Z=1.23(P=0.22)				

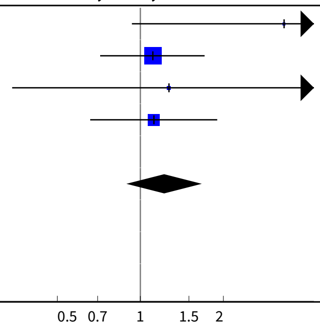

Favours vitamin E — Favours placebo

Analysis 1.3. Comparison 1 Vitamin E versus placebo, Outcome 3 Neovascular AMD or geographic atrophy separately.

Study or subgroup	Vitamin E n/N	Placebo n/N	Risk Ratio M-H, Fixed, 95% CI
**1.3.1 Neovascular AMD**			
ATBC 1998	8/494	2/447	3.62[0.77,16.95]
**1.3.2 Geographic atrophy**			
ATBC 1998	3/494	1/447	2.71[0.28,26]

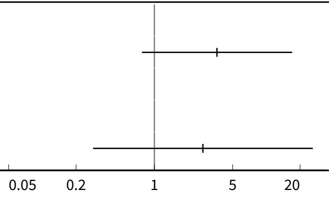

Favours vitamin E — Favours placebo

Comparison 2. Beta-carotene versus placebo

Outcome or subgroup title	No. of studies	No. of partici-pants	Statistical method	Effect size
1 Any AMD	2	22083	Risk Ratio (M-H, Fixed, 95% CI)	1.00 [0.88, 1.14]
2 Late AMD (either neovascular AMD or geographic atrophy or both)	2	22083	Risk Ratio (M-H, Fixed, 95% CI)	0.90 [0.65, 1.24]
3 Neovascular AMD or geographic atrophy separately	1		Risk Ratio (M-H, Fixed, 95% CI)	Totals not selected
3.1 Neovascular AMD	1		Risk Ratio (M-H, Fixed, 95% CI)	0.0 [0.0, 0.0]
3.2 Geographic atrophy	1		Risk Ratio (M-H, Fixed, 95% CI)	0.0 [0.0, 0.0]

Copyright © 2017 The Cochrane Collaboration. Published by John Wiley & Sons, Ltd.

Analysis 2.1. Comparison 2 Beta-carotene versus placebo, Outcome 1 Any AMD.

Study or subgroup	Beta-carotene n/N	Placebo n/N	Risk Ratio M-H, Fixed, 95% CI	Weight	Risk Ratio M-H, Fixed, 95% CI
ATBC 1998	141/491	128/450		32.74%	1.01[0.82,1.24]
PHS I 2007	275/10585	274/10557		67.26%	1[0.85,1.18]
**Total (95% CI)**	**11076**	**11007**		**100%**	**1[0.88,1.14]**
Total events: 416 (Beta-carotene), 402 (Placebo)					
Heterogeneity: Tau²=0; Chi²=0, df=1(P=0.95); I²=0%					
Test for overall effect: Z=0.06(P=0.95)					
	Favours beta-carotene		1	Favours placebo	

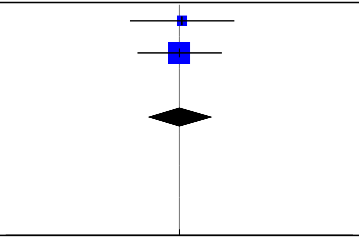

Analysis 2.2. Comparison 2 Beta-carotene versus placebo, Outcome 2 Late AMD (either neovascular AMD or geographic atrophy or both).

Study or subgroup	Beta-carotene n/N	Placebo n/N	Risk Ratio M-H, Fixed, 95% CI	Weight	Risk Ratio M-H, Fixed, 95% CI
ATBC 1998	5/491	9/450		12.44%	0.51[0.17,1.51]
PHS I 2007	63/10585	66/10557		87.56%	0.95[0.67,1.34]
**Total (95% CI)**	**11076**	**11007**		**100%**	**0.9[0.65,1.24]**
Total events: 68 (Beta-carotene), 75 (Placebo)					
Heterogeneity: Tau²=0; Chi²=1.16, df=1(P=0.28); I²=13.79%					
Test for overall effect: Z=0.65(P=0.51)					
	Favours beta-carotene		0.1 0.2 0.5 1 2 5 10	Favours placebo	

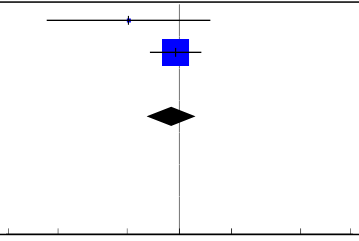

Analysis 2.3. Comparison 2 Beta-carotene versus placebo, Outcome 3 Neovascular AMD or geographic atrophy separately.

Study or subgroup	Beta-carotene n/N	Placebo n/N	Risk Ratio M-H, Fixed, 95% CI	Risk Ratio M-H, Fixed, 95% CI
**2.3.1 Neovascular AMD**				
ATBC 1998	4/491	6/450		0.61[0.17,2.15]
**2.3.2 Geographic atrophy**				
ATBC 1998	1/491	3/450		0.31[0.03,2.93]
		Favours beta-carotene	0.05 0.2 1 5 20	Favours placebo

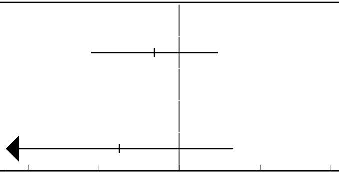

Comparison 3. Vitamin C versus placebo

Outcome or subgroup title	No. of studies	No. of participants	Statistical method	Effect size
1 AMD	1		Risk Ratio (M-H, Fixed, 95% CI)	Totals not selected
1.1 Any AMD	1		Risk Ratio (M-H, Fixed, 95% CI)	0.0 [0.0, 0.0]

Copyright © 2017 The Cochrane Collaboration. Published by John Wiley & Sons, Ltd.

Outcome or subgroup title	No. of studies	No. of participants	Statistical method	Effect size
1.2 Late AMD (either neovascular AMD or geographic atrophy or both)	1		Risk Ratio (M-H, Fixed, 95% CI)	0.0 [0.0, 0.0]

Analysis 3.1. Comparison 3 Vitamin C versus placebo, Outcome 1 AMD.

Study or subgroup	Vitamin C n/N	Placebo n/N	Risk Ratio M-H, Fixed, 95% CI	Risk Ratio M-H, Fixed, 95% CI
**3.1.1 Any AMD**				
PHS II 2012	179/7149	184/7087		0.96[0.79,1.18]
**3.1.2 Late AMD (either neovascular AMD or geographic atrophy or both)**				
PHS II 2012	39/7149	41/7087		0.94[0.61,1.46]
		Favours vitamin C		Favours placebo

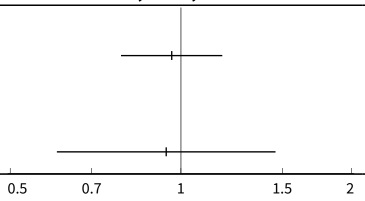

Comparison 4. Multivitamin versus placebo

Outcome or subgroup title	No. of studies	No. of participants	Statistical method	Effect size
1 AMD	1		Risk Ratio (M-H, Fixed, 95% CI)	Totals not selected
1.1 Any AMD	1		Risk Ratio (M-H, Fixed, 95% CI)	0.0 [0.0, 0.0]
1.2 Late AMD (either neovascular AMD or geographic atrophy or both)	1		Risk Ratio (M-H, Fixed, 95% CI)	0.0 [0.0, 0.0]

Analysis 4.1. Comparison 4 Multivitamin versus placebo, Outcome 1 AMD.

Study or subgroup	Multivitamin n/N	Placebo n/N	Risk Ratio M-H, Fixed, 95% CI	Risk Ratio M-H, Fixed, 95% CI
**4.1.1 Any AMD**				
PHS II 2012	294/7111	244/7122		1.21[1.02,1.43]
**4.1.2 Late AMD (either neovascular AMD or geographic atrophy or both)**				
PHS II 2012	79/7111	65/7122		1.22[0.88,1.69]
		Favours multivitamin		Favours placebo

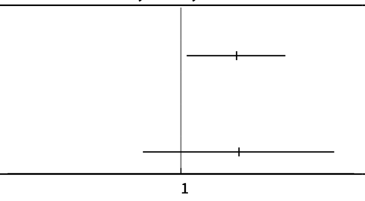

ADDITIONAL TABLES

Copyright © 2017 The Cochrane Collaboration. Published by John Wiley & Sons, Ltd.

Table 1. Mapping the definition of AMD used in included studies to the review outcomes

Definition of AMD used in this review	Study		
	ATBC 1998	PHS I 2007 PHS II 2012; WHS 2010	VECAT 2002
No AMD	0 = no ARM	Did not self-report or no signs listed below in medical records	-
Any AMD	I = dry maculopathy, with hard drusen, pigmentary changes, or both<br><br>II = soft macular drusen<br><br>III = disciform degeneration<br><br>IV = geographic atrophy.	Drusen, RPE hypo or hyperpigmentation, geographic atrophy, RPE detachment, subretinal neovascular membrane, or disciform scar	Early AMD 1: Soft intermediate or soft distinct or soft indistinct or pigment changes (hyperpigmentation or hypopigmentation)<br><br>Early AMD 2: Soft intermediate or soft distinct or soft indistinct and pigment changes (hyperpigmentation or hypopigmentation)<br><br>Early AMD 3: Soft distinct or soft indistinct or pigment changes (hyperpigmentation or hypopigmentation)<br><br>Early AMD 4: Soft distinct or soft indistinct and pigment changes (hyperpigmentation or hypopigmentation)<br><br>Late AMD: Serous or haemorrhagic detachment of the RPE or sensory retina, characteristic haemorrhages, or subretinal fibrous scars, central areolar zone of retinal pigment epithelial atrophy with visible choroidal vessels, at least 175 µm in diameter
Late AMD	III = disciform degeneration<br><br>IV = geographic atrophy.	Geographic atrophy, RPE detachment, subretinal neovascular membrane, or disciform scar	Serous or haemorrhagic detachment of the RPE or sensory retina, characteristic haemorrhages, or subretinal fibrous scars, central areolar zone of retinal pigment epithelial atrophy with visible choroidal vessels, at least 175 µm in diameter
Neovascular AMD	III = disciform degeneration	RPE detachment, subretinal neovascular membrane, or disciform scar	Serous or haemorrhagic detachment of the RPE or sensory retina, characteristic haemorrhages, or subretinal fibrous scars
Geographic atrophy	IV = geographic atrophy.	Geographic atrophy	Central areolar zone of retinal pigment epithelial atrophy with visible choroidal vessels, at least 175 µm in diameter, in the absence of signs of neovascular AMD in the same eye

RPE: retinal pigment epithelial
Method of detection: Grading of fundus photographs (ATBC 1998; VECAT 2002), and medical record review after self-report of AMD diagnosis (PHS I 2007; PHS II 2012; WHS 2010)

APPENDICES

Appendix 1. CENTRAL search strategy

#1 MeSH descriptor Macular Degeneration
#2 MeSH descriptor Retinal Degeneration
#3 MeSH descriptor Retinal Neovascularization
#4 MeSH descriptor Choroidal Neovascularization

Copyright © 2017 The Cochrane Collaboration. Published by John Wiley & Sons, Ltd.

#5 MeSH descriptor Macula Lutea
#6 macula* near lutea*
#7 (macula* or retina* or choroid*) near/4 degenerat*
#8 (macula* or retina* or choroid*) near/4 neovascul*
#9 maculopath*
#10 (#1 OR #2 OR #3 OR #4 OR #5 OR #6 OR #7 OR #8 OR #9)
#11 MeSH descriptor Vitamins
#12 vitamin*
#13 MeSH descriptor Vitamin A
#14 retinol*
#15 MeSH descriptor beta Carotene
#16 caroten*
#17 MeSH descriptor Ascorbic Acid
#18 ascorbic next acid
#19 MeSH descriptor Vitamin E
#20 MeSH descriptor alpha-Tocopherol
#21 alpha tocopherol*
#22 MeSH descriptor Vitamin B 12
#23 cobalamin*
#24 MeSH descriptor Antioxidants
#25 antioxidant* or anti oxidant*
#26 MeSH descriptor Carotenoids
#27 carotenoid*
#28 MeSH descriptor Zinc
#29 zinc*
#30 MeSH descriptor Riboflavin
#31 riboflavin*
#32 MeSH descriptor Selenium
#33 selenium*
#34 MeSH descriptor Lutein
#35 lutein*
#36 MeSH descriptor Xanthophylls
#37 xanthophyll*
#38 zeaxanthin*
#39 (#11 OR #12 OR #13 OR #14 OR #15 OR #16 OR #17 OR #18 OR #19 OR #20 OR #21 OR #22 OR #23 OR #24)
#40 (#25 OR #26 OR #27 OR #28 OR #29 OR #30 OR #31 OR #32 OR #33 OR #34 OR #35 OR #36 OR #37 OR #38)
#41 (#39 OR #40)
#42 (#10 AND #41)

Appendix 2. MEDLINE Ovid search strategy

1. exp clinical trial/ [publication type]
2. (randomized or randomised).ab,ti.
3. placebo.ab,ti.
4. dt.fs.
5. randomly.ab,ti.
6. trial.ab,ti.
7. groups.ab,ti.
8. or/1-7
9. exp animals/
10. exp humans/
11. 9 not (9 and 10)
12. 8 not 11
13. exp macular degeneration/
14. exp retinal degeneration/
15. exp retinal neovascularization/
16. exp choroidal neovascularization/
17. exp macula lutea/
18. maculopath$.tw.
19. ((macul$ or retina$ or choroid$) adj3 degener$).tw.
20. ((macul$ or retina$ or choroid$) adj3 neovasc$).tw.
21. (macula$ adj2 lutea).tw.

Copyright © 2017 The Cochrane Collaboration. Published by John Wiley & Sons, Ltd.

22. or/13-21
23. exp vitamins/
24. exp vitamin A/
25. vitamin A.tw.
26. retinol$.tw.
27. exp beta carotene/
28. caroten$.tw.
29. exp ascorbic acid/
30. ascorbic acid$.tw.
31. vitamin C.tw.
32. exp Vitamin E/
33. exp alpha tocopherol/
34. alpha?tocopherol$.tw.
35. alpha tocopherol$.tw.
36. vitamin E.tw.
37. exp Vitamin B12/
38. vitamin B12.tw.
39. cobalamin$.tw.
40. exp antioxidants/
41. ((antioxidant$ or anti) adj1 oxidant$).tw.
42. exp carotenoids/
43. carotenoid$.tw.
44. exp zinc/
45. zinc$.tw.
46. exp riboflavin/
47. riboflavin$.tw.
48. exp selenium/
49. selenium$.tw.
50. exp lutein/
51. lutein$.tw.
52. exp xanthophylls/
53. xanthophyll.tw.
54. zeaxanthin$.tw.
55. or/23-54
56. 22 and 55
57. 12 and 56

The search filter for trials at the beginning of the MEDLINE strategy is from the published paper by Glanville 2006.

Appendix 3. Embase Ovid search strategy

1. exp randomized controlled trial/
2. exp randomization/
3. exp double blind procedure/
4. exp single blind procedure/
5. random$.tw.
6. or/1-5
7. (animal or animal experiment).sh.
8. human.sh.
9. 7 and 8
10. 7 not 9
11. 6 not 10
12. exp clinical trial/
13. (clin$ adj3 trial$).tw.
14. ((singl$ or doubl$ or trebl$ or tripl$) adj3 (blind$ or mask$)).tw.
15. exp placebo/
16. placebo$.tw.
17. random$.tw.
18. exp experimental design/
19. exp crossover procedure/
20. exp control group/
21. exp latin square design/

Copyright © 2017 The Cochrane Collaboration. Published by John Wiley & Sons, Ltd.

22. or/12-21
23. 22 not 10
24. 23 not 11
25. exp comparative study/
26. exp evaluation/
27. exp prospective study/
28. (control$ or prospectiv$ or volunteer$).tw.
29. or/25-28
30. 29 not 10
31. 30 not (11 or 23)
32. 11 or 24 or 31
33. exp retina macula degeneration/
34. exp retina degeneration/
35. exp retina neovascularization/
36. exp subretinal neovascularization/
37. maculopath$.tw.
38. ((macul$ or retina$ or choroid$) adj3 degener$).tw.
39. ((macul$ or retina$ or choroid$) adj3 neovasc$).tw.
40. exp retina macula lutea/
41. (macula$ adj2 lutea$).tw.
42. or/33-41
43. exp vitamins/
44. exp Retinol/
45. vitamin A.tw.
46. retinol$.tw.
47. exp beta carotene/
48. caroten$.tw.
49. exp ascorbic acid/
50. ascorbic acid$.tw.
51. vitamin C.tw.
52. exp alpha tocopherol/
53. alpha?tocopherol$.tw.
54. alpha tocopherol$.tw.
55. vitamin E.tw.
56. vitamin B12.tw.
57. exp cyanocobalamin/
58. cobalamin$.tw.
59. exp antioxidants/
60. ((antioxidant$ or anti) adj1 oxidant$).tw.
61. exp carotenoid/
62. exp zinc/
63. zinc$.tw.
64. exp riboflavin/
65. riboflavin$.tw.
66. exp selenium/
67. selenium$.tw.
68. exp zeaxanthin/
69. zeaxanthin$.tw.
70. lutein$.tw.
71. xanthophyll.tw.
72. or/43-71
73. 42 and 72
74. 32 and 73

Appendix 4. AMED Ovid search strategy

1. exp eye disease/
2. exp vision disorders/
3. exp retinal disease/
4. maculopath$.tw.
5. ((macul$ or retina$ or choroid$) adj3 degenerat$).tw.
6. ((macul$ or retina$ or choroid$) adj3 neovasc$).tw.

Copyright © 2017 The Cochrane Collaboration. Published by John Wiley & Sons, Ltd.

7. or/1-6
8. exp vitamins/
9. vitamin A.tw.
10. retinol$.tw.
11. exp carotenoids/
12. caroten$.tw.
13. exp ascorbic acid/
14. ascorbic acid$.tw.
15. vitamin C.tw.
16. vitamin E.tw.
17. alpha tocopherol$.tw.
18. vitamin B12.tw.
19. cobalamin$.tw.
20. exp antioxidants/
21. ((antioxidant$ or anti) adj1 oxidant$).tw.
22. zinc/
23. zinc$.tw.
24. riboflavin$.tw.
25. selenium/
26. selenium$.tw.
27. lutein$.tw.
28. xanthophylls.tw.
29. zeaxanthin$.tw.
30. or/8-29
31. 7 and 30

Appendix 5. OpenGrey search strategy

(macular degeneration OR AMD) AND (antioxidant OR vitamin OR carotene OR selenium OR tocopherol)

Appendix 6. ISRCTN search strategy

(macular degeneration OR AMD) AND (antioxidant OR vitamin OR carotene OR selenium OR tocopherol)

Appendix 7. ClinicalTrials.gov search strategy

(Macular Degeneration OR AMD) AND (Antioxidant OR Vitamin OR Carotene OR Selenium OR Tocopherol)

Appendix 8. ICTRP search strategy

Macular Degeneration OR AMD = Condition AND Antioxidant OR Vitamin OR Carotene OR Selenium OR Tocopherol = Intervention

Appendix 9. MEDLINE Ovid adverse effects search strategy

1. exp retinal degeneration/
2. retinal neovascularization/
3. choroidal neovascularization/
4. exp macula lutea/
5. (macula$ adj2 lutea).tw.
6. ((macul$ or retina$ or choroid$) adj4 degener$).tw.
7. ((macul$ or retina$ or choroid$) adj4 neovasc$).tw.
8. (AMD or ARMD or CNV).tw.
9. maculopath$.tw.
10. or/1-9
11. exp vitamins/
12. vitamin A.tw.
13. retinol$.tw.
14. (caroten$ or betacaroten$).tw.
15. ascorbic acid$.tw.
16. vitamin C.tw.
17. alpha?tocopherol$.tw.
18. alpha tocopherol$.tw.
19. vitamin E.tw.
20. ((antioxidant$ or anti) adj1 oxidant$).tw.
21. zinc/

Copyright © 2017 The Cochrane Collaboration. Published by John Wiley & Sons, Ltd.

22. zinc$.tw.
23. or/11-22
24. 10 and 23
25. ae.fs.
26. 24 and 25
27. limit 26 to (meta analysis or randomized controlled trial or "review")

WHAT'S NEW

Date	Event	Description
30 July 2017	Amended	Minor amendment to the updated version of 2017: Cochrane Review Evans 2017 reference amended as updated version of this review published.

HISTORY

Protocol first published: Issue 3, 1997
Review first published: Issue 4, 1999

Date	Event	Description
29 March 2017	New search has been performed	Issue 7, 2017: Electronic searches were updated.
29 March 2017	New citation required but conclusions have not changed	Issue 7, 2017: One new trial (PHS II 2012), which has now been completed, was included in the review update.
19 April 2012	New citation required but conclusions have not changed	2012, Issue 6: The author byline has changed. Katherine Henshaw has been replaced by a new author, John Lawrenson.
19 April 2012	New search has been performed	2012, Issue 6: New searches yielded one new trial. New 'Risk of bias' grading and a 'Summary of findings' table have been included.
28 August 2008	Amended	Converted to new review format.
8 November 2007	New citation required and conclusions have changed	Substantive amendment. 2008, Issue 1: The results for PHS I were included. AREDS was previously included in this review but as no numerical data were available from the study on prevention of AMD, it was excluded from the review. The results of AREDS are presented in the review 'Antioxidants for slowing down the progression of AMD'.

CONTRIBUTIONS OF AUTHORS

JE assessed studies for inclusion and exclusion, assessed risk of bias, extracted data, entered data and wrote the text of the review.
JL assessed studies for inclusion and exclusion, assessed risk of bias, extracted data, and reviewed and commented on the text of the review.

DECLARATIONS OF INTEREST

None known.

Copyright © 2017 The Cochrane Collaboration. Published by John Wiley & Sons, Ltd.

SOURCES OF SUPPORT

Internal sources

- No sources of support supplied

External sources

- National Institute for Health Research (NIHR), UK.

    * Richard Wormald, Co-ordinating Editor for Cochrane Eyes and Vision (CEV) acknowledges financial support for his CEV research sessions from the Department of Health through the award made by the NIHR to Moorfields Eye Hospital NHS Foundation Trust and UCL Institute of Ophthalmology for a Specialist Biomedical Research Centre for Ophthalmology.
    * This review was supported by the NIHR, via Cochrane Infrastructure funding to the CEV UK editorial base which funds part of Jennifer Evans's salary.

  The views and opinions expressed therein are those of the authors and do not necessarily reflect those of the Systematic Reviews Programme, NIHR, NHS or the Department of Health.

DIFFERENCES BETWEEN PROTOCOL AND REVIEW

The original protocol was published in 1999 (Evans 1999). Since that time, there have been methodological improvements within Cochrane, and the methods have been updated to include assessment of risk of bias, 'Summary of findings' tables, GRADE assessment, and better consideration of unit of analysis issues.

For the 2017 update, we modified the outcome measures to ensure they were in line with those being used as part of the macular degeneration guidelines being prepared by NICE (NICE 2016). In previous versions of this review the outcomes were:

1. number of participants developing AMD;
2. number of participants with visual loss due to AMD;
3. quality of life measures;
4. any adverse outcomes reported.

In previous versions of this review, we pooled all antioxidants into one analysis. This was not possible in the current version because of overlapping participants in two trials.

INDEX TERMS

Medical Subject Headings (MeSH)

*Dietary Supplements; Antioxidants [*administration & dosage] [adverse effects]; Ascorbic Acid [administration & dosage] [adverse effects]; Drug Combinations; Macular Degeneration [*prevention & control]; Minerals [administration & dosage]; Randomized Controlled Trials as Topic; Vitamin E [administration & dosage] [adverse effects]; Vitamins [*administration & dosage] [adverse effects]; alpha-Tocopherol [administration & dosage]; beta Carotene [administration & dosage]

MeSH check words

Humans

Copyright © 2017 The Cochrane Collaboration. Published by John Wiley & Sons, Ltd.
