# Supplementary material for: Insights into the nutritional prevention of macular degeneration based on a comparative topic modeling approach
Source: PeerJ Comput Sci. 2024 Mar 20;10:e1940. doi: 10.7717/peerj-cs.1940 (PMC11042009; doi:10.7717/peerj-cs.1940)
Supplement: Supplemental Information 3 [file peerj-cs-10-1940-s003.zip › negative_result_reports/Biol_BC_2014_IvanAgtmaal.pdf]

The Role of Nutritional Supplements in the Progression of Age-related Macular Degeneration.

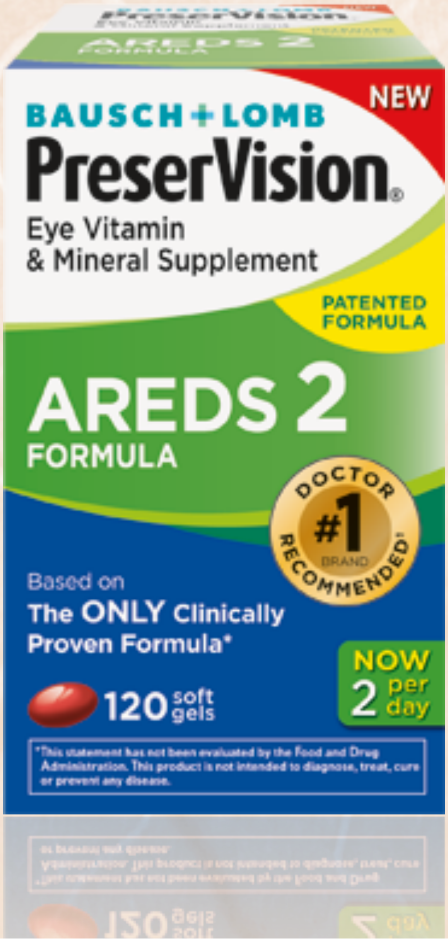

Irene van Agtmaal (2126435)
Supervisors: dr. R.P.H.M. Müskens, drs. E.A. Huiskamp
Supervisor 2: Prof. dr. N.M. Jansonius
Bachelor thesis of Biology (Biomedical Sciences)
Rijksuniversiteit Groningen, 7th of July 2014

Background picture provided by G. Dijkman, LUMC
Picture from: Bausch & Lomb (n.d.). New! Preservision AREDS 2 Formula. Retrieved from http://www.bausch.com/en/ecp/our-products/eye-vitamins/age-related-eye-vitamins-ecp/preservision2-areds-eye-vitamins/

- ABSTRACT -

Age-related macular degeneration (AMD) is the leading cause of legal blindness in people aged over 55 in Western countries. Because the proportion of the aged population is increasing and there are limited therapeutic options, AMD is becoming an important condition worldwide. This review describes the pathogenesis of AMD and current literature on the role of certain nutritional supplements in the progression of AMD.

Genetic factors, oxidative stress, apoptosis, angiogenesis and inflammation might be involved in the AMD pathogenesis. Genetic and environmental components that can influence the risk for developing AMD include age, smoking, BMI, and genetic variants like CFH Y402H, ARMS2 A69S and C3 R102G. Several small trials have investigated the association between diet, nutrient intake and AMD. The largest study investigating the effect of nutritional supplements on the progression of AMD is the Age-Related Eye Disease Study (AREDS). AREDS demonstrated that 5-year intake of a combination of oral supplements consisting of antioxidants (β-carotene, vitamin C, E), minerals, zinc and copper could reduce the risk of progression to advanced AMD by 25%. Lutein, zeaxanthin, vitamin B and the ω-3 fatty acids DHA and EPA have also been reported to decrease AMD progression. However, the AREDS2 study showed no overall improvement of the original AREDS formula when adding lutein, zeaxanthin, EPA and DHA. Recommendations in the current literature on whether or not people with AMD should take antioxidant or ω3-LCPUFA supplements are primarily based on the results of the AREDS study. Although other trials have been done, they have generally been small and of short duration, resulting in inconclusive results.

Although some results have been promising, there is insufficient evidence in the literature to recommend routine nutritional supplementation for slowing down AMD progression. Further large scale and sample randomised controlled trials need to be done in this area to provide sufficient evidence for the use of nutritional supplements in AMD.

- CONTENTS -

I. General Introduction .... *p. 3*

II. Age-related Macular Degeneration .... *p. 5*
IIa. Different stages in AMD .... *p. 5*
IIb. Different types of AMD .... *p. 6*
IIc. Detection techniques .... *p. 7*
IId. Current therapies .... *p. 8*

III. Pathogenic Mechanisms in AMD .... *p. 10*
IIIa. Genetics .... *p. 10*
IIIb. Oxidative stress .... *p. 11*
IIIc. Angiogenesis .... *p. 13*
IIId. Apoptosis .... *p. 14*
IIIe. Inflammation .... *p. 15*

IV. Nutritional Supplements and AMD .... *p. 18*
IVa. Antioxidants .... *p. 18*
IVc. Zinc .... *p. 19*
IVb. Omega 3 fatty acids .... *p. 20*
IVc. AREDS studies .... *p. 20*

V. Discussion .... *p. 25*

VI. Literature .... *p. 27*

VII. Appendix .... *p. 33*

I. GENERAL INTRODUCTION

Age-related macular degeneration (AMD) is the leading cause of legal blindness in people over the age of 55 years in Western countries (Klein et al. 2010). It is estimated to affect about 50 million people worldwide (Buentello-Volante et al. 2012). AMD is defined as an abnormality of the retinal pigment epithelium (RPE) that leads to degeneration of the overlying photoreceptors in the macula and consequent loss of central vision (Zhang et al. 2012). The macula lutea is a region of the retina that is packed with light-sensitive cells, called photoreceptors (Figure 1). The macula is responsible for central, high-resolution vision needed for e.g. reading, recognizing faces and driving. Damage leads to visual impairments like haziness, central scotoma or metamorphopsia in the central vision of patients (Figure 2) (as described in the NOG guideline for AMD, 2014). AMD has a chronic progressive course and can cause an extensive decline in the quality of life, often requiring lifelong observation and therapy (Pinazo-Durán et al. 2014a). Data pooled from several population-based studies (the Beaver Dam Eye Study, the Rotterdam Study, the Blue Mountains Eye study) have estimated the prevalence of advanced age-related macular degeneration to be 0·2% in individuals aged 55 to 64 years (Coleman et al. 2008, Vingerling et al. 1995, Klein et al. 2007, Mitchell et al. 2002). Currently, there are only a few effective treatments for AMD. Unfortunately, the acknowledged treatments are only effective in a small proportion of patients. Because the proportion of the aged population is continuously increasing, AMD is becoming a socioeconomic problem and important condition worldwide (Ferris et al. 2013, Pinazo-Durán et al. 2014a).

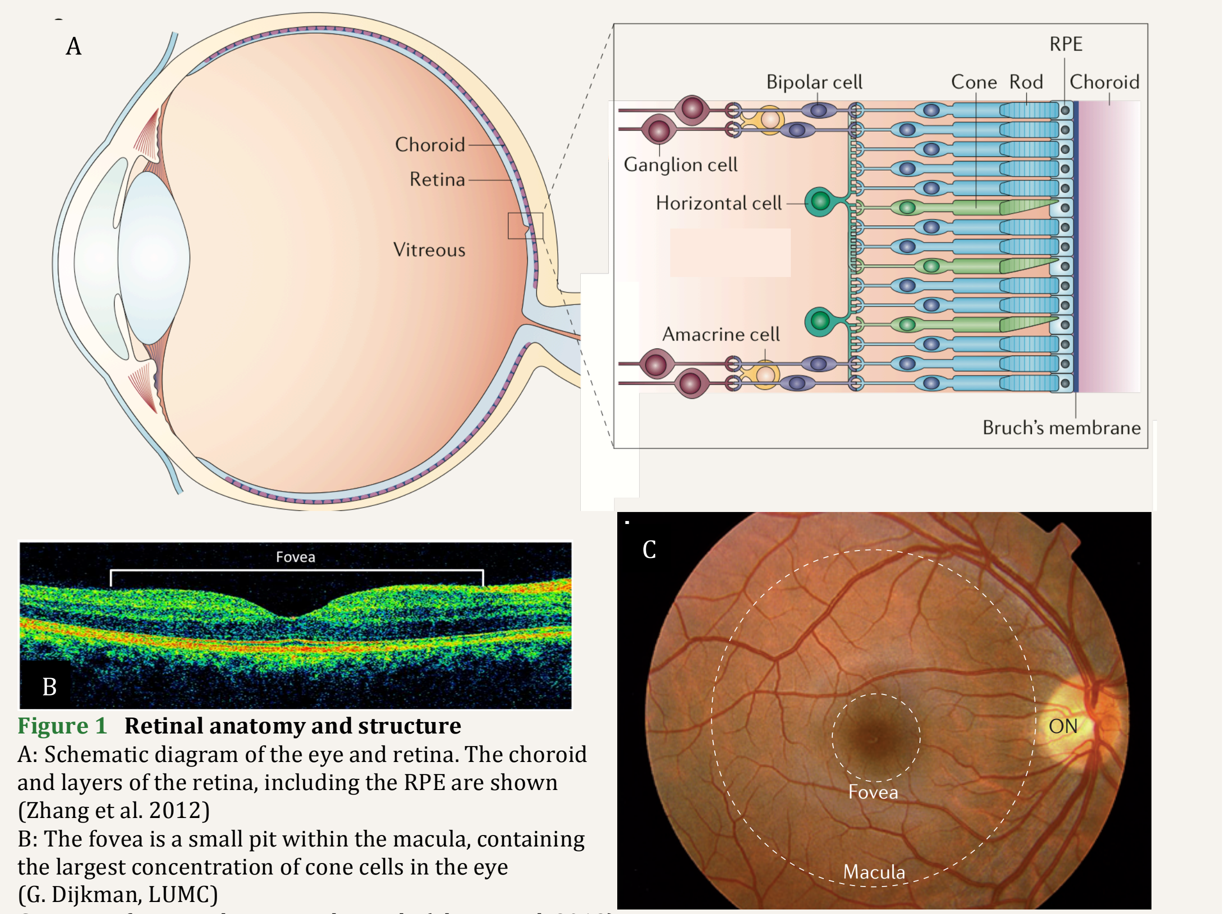

Figure 1 Retinal anatomy and structure
A: Schematic diagram of the eye and retina. The choroid and layers of the retina, including the RPE are shown (Zhang et al. 2012)
B: The fovea is a small pit within the macula, containing the largest concentration of cone cells in the eye (G. Dijkman, LUMC)
C: Image of a normal retina and macula (Zhang et al. 2012)

AMD appears to be a complex disease with demographic, environmental and genetic risk factors (Ding et al. 2009), of which age is considered to be the strongest risk factor (Tombran-Tink and Barnstable 2006, Coleman et al. 2008). Increasing evidence suggests that there are genetic factors involved in AMD. Studies have demonstrated an increased risk of AMD when a first-degree family member is affected and approximately 20% of the AMD patients have a positive family history (Tombran-Tink and Barnstable 2006). Currently, the most important genes associated with AMD are complement factor H (CFH) on chromosome 1q32 and LOC387715(ARMS2)/HtrA1 on chromosome 10q26 (Coleman et al. 2008). All forms of AMD are more prevalent in the white population than in more darkly pigmented races like

Blacks, Asians and Hispanics (Ferris et al. 2013). Female sex may also be a risk factor in individuals aged over 75 years (Smith et al. 2001).

Cigarette smoking is considered a strong oxidative stressor and the most preventable risk factor for AMD (Rickman et al. 2013, Eye Disease Case-Control Study Group 1992). The Rotterdam Study showed a dose-response relationship between smoking and AMD (Vingerling et al. 1996). Also, vascular risk factors have been hypothesized to be important pathogenic factors for the development of AMD, although reports have shown conflicting results (Tombran-Tink and Barnstable 2006, Ambati et al. 2003). High levels of exposure to blue or visible light might cause ocular damage, but so far reports have been conflicting.

Gene environment studies of the CFH locus provide evidence that modifiable factors can alter genetic susceptibility. In a study by Seddon et al. (2006a), susceptibility to advanced AMD associated with CFH Y402H was modified by BMI, and both BMI and smoking increased risk of advanced AMD.

Important advances in the understanding of AMD pathogenesis generated a foundation for further epidemiological and interventional studies focussing on the role of diet and nutritional supplements in the incidence and progression of AMD (Pinazo-Durán et al. 2014a). The Age-Related Eye Disease Study (AREDS) investigated the effect of high doses of zinc, vitamin A and C and β-carotene on the progression of AMD. The results were promising: 5-year intake of the nutritional supplements reduced the risk of progression to advanced AMD by 25% and the risk of moderate vision loss by 19% (AREDS Research Group 2001). In 2013, the preliminary results of the AREDS2 study were published. In AREDS2, the zinc-dose was reduced, β-carotene was replaced by lutein and zeaxanthin and long-chain omega-3 fatty acids were added to the original AREDS nutritional supplement, but the results of AREDS2 showed no overall improvement compared to the original AREDS formula (AREDS2 Research Group 2013).

This growing interest has led to numerous studies examining the role of diet and nutrition in the development of AMD. However, this research is still in its early stages and has so far led to different results, giving rise to the question: *'Could certain nutritional supplements slow down the progression of age-related macular degeneration?'*

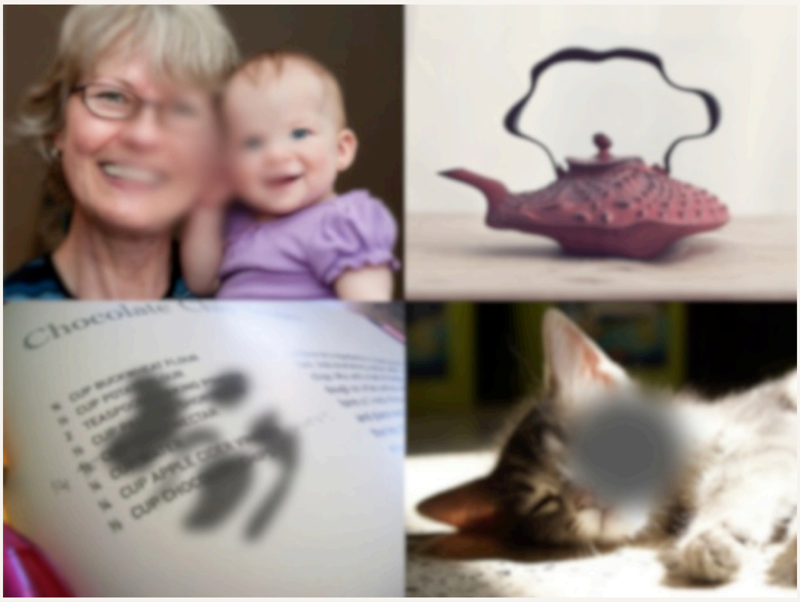

Figure 2 Vision of AMD patients. AMD-patients may notice blurry or dark spots in their central vision (scotoma), difficulties distinguishing sharp details and/or may have the feeling there is insufficient light when reading. Patients may also experience metamorphopsia (when lines appear wavy and shapes are distorted) (Picture provided by the Angiogenesis Foundation).

II. AGE-RELATED MACULAR DEGENERATION

IIa. Different stages of AMD

In the aging eye, subretinal extracellular deposits composed of lipids and glycoproteins accumulate between the basement membrane of the RPE and Bruch's membrane as discrete accumulations, called drusen (Ding et al. 2009, Rickman et al. 2013) (Figure 3). The clinical hallmark of AMD is the appearance of these drusen (Ambati et al. 2003). Based on the appearance of the macula, patients are currently classified as having early AMD, intermediate AMD and late or advanced AMD (Rickman et al. 2013). The appearance of small drusen (<63µm in diameter), or drupelets, is a normal age-related change in the eye and does not implicate an increased risk of developing late AMD (Donoso et al. 2006, Rickman et al. 2013, Ferris et al. 2013). However, when there are multiple small and intermediate drusen (63-125µm) present in the retina but no pigmentary abnormalities related to AMD, persons should be considered to have early AMD. Persons with large drusen (>125µm) or pigmentary abnormalities associated with drusen are considered to have intermediate AMD (Ferris et al. 2013). In early and intermediate AMD, the visual function of patients is often affected (Rickman et al. 2013). The appearance of lesions associated with neovascular AMD of geographic atrophy (GA) is considered to be an indication for late AMD (Ferris et al. 2013). Central vision is often severely affected in late or advanced stages of AMD and patients can experience progressive loss of central vision (Rickman et al. 2013).

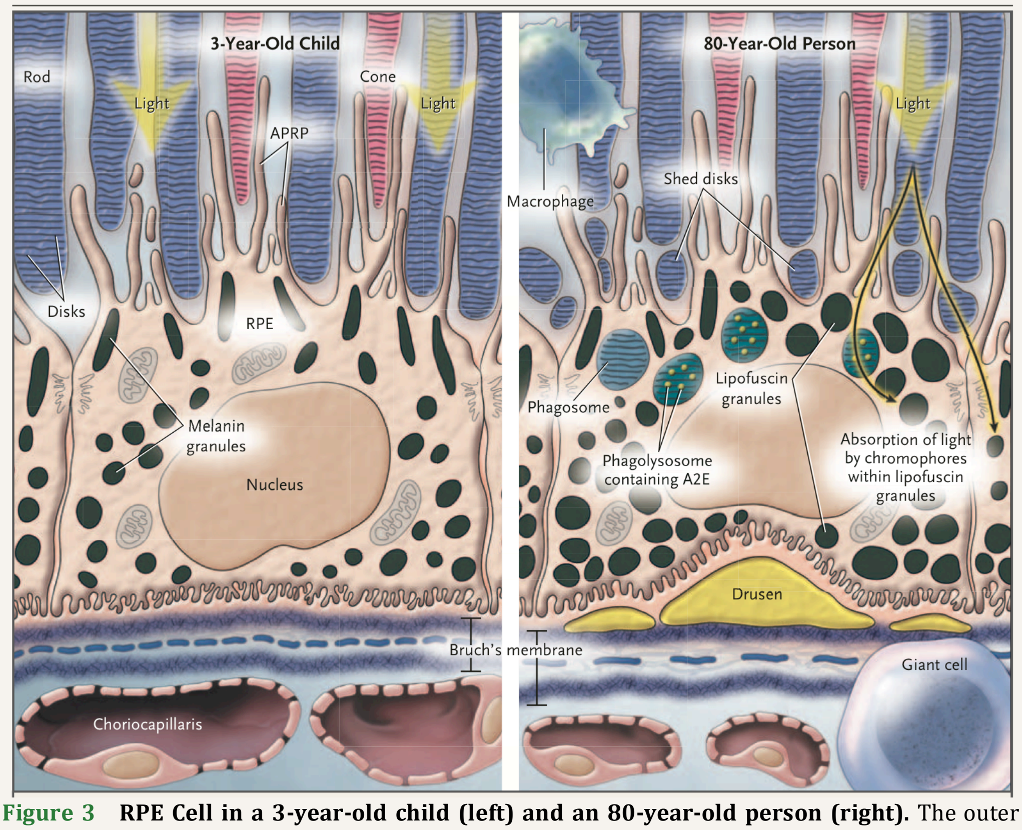

Figure 3 RPE Cell in a 3-year-old child (left) and an 80-year-old person (right). The outer segments of the rods and cones are embedded in the inter-photoreceptor matrix (blue-grey areas) and partially surrounded by apical pseudopodial RPE processes (APRP). Phagosomes can encapsulate disks and digest them in phagolysosomes in the cell cytoplasm of the RPE (right). Macrophages and giant cells (fused macrophages) remove cellular debris around the cell. Light-induced toxicity occurs as light is absorbed by the various chromophores (photosensitive compounds) in the lipofuscin granules. This damages DNA and cell membranes and causes inflammation and apoptosis. The right hand panel shows enlarged lipofuscin granules, thickened Burch's membrane by the formation of drusen and attenuation of the choriocappilaris. The central elastic lamina in Bruch's membrane becomes more porous in old age (de Jong 2006).

IIb. Different types of AMD

Drusen are located between the basement membrane of the RPE and Bruch's membrane and the formation of drusen can be caused by RPE dysfunction or by a change in the composition of permeability (to nutrients) of Bruch's membrane (Ambati et al. 2003, Zhang et al. 2012). They are most frequently found as clusters within the macular region and can vary in size, colour and shape, and tend to increase in number with advancing age. In early AMD stages, drusen are opthalmoscopically visible as yellow-white deposits (Ding et al. 2009).

Currently AMD is divided into two basic subtypes: 'dry' AMD (90% of the cases) and 'wet' or neovascular AMD (10% of the cases) (Singer 2014). Early or intermediate AMD with simple drusen can progress to geographic atrophy (GA), the 'dry' advanced form of AMD, or to neovascular AMD, which is the 'wet' advanced form of AMD (Figure 4). Neovascular AMD can eventually lead to the formation of scar tissue, which in turn is considered 'dry' AMD (Ferris et al. 2013).

There are two distinct types of deposits in the eye: basal laminar deposits (BlamD) and basal linear deposits (BlinL). The combination of these deposits with secondary changes can lead to the formation of drusen in the RPE (Coleman et al. 2008) (Figure 4A). Drusen are clinically classified as 'hard' or 'soft' (Figure 5). Hard drusen are relatively common in elderly patients with or without AMD and are - in small numbers - not considered an important risk factor for the development of AMD (Ding et al. 2013, Ambati et al. 2003). Soft drusen are associated with the detachment of the retinal pigment epithelium (RPE) and abnormal Bruch's membrane alterations (Coleman et al. 2008).

The composition of drusen has been investigated. Many different molecules have been identified, including glycoproteins, (a)lipoproteins B and E, lipids, vitronectin and complement factors (Coleman et al. 2008, Russel et al. 2005, Hageman and Mullins 1999). Macrophages have been detected in regressing drusen, suggesting macrophages are recruited to eliminate the deposits within the Bruch's membrane. Activated microglia also accumulate in AMD (Coleman et al. 2008). Although many studies have investigated the composition and characteristics of human drusen, further research is needed to elucidate its significance for AMD prevention (Pinazo-Durán et al. 2014a).

Geographic atrophy

GA is the advanced non-neovascular form of AMD, which involves the centre of the macula (Damico et al. 2012, Coleman et al. 2008). GA refers to the presence of discrete areas of retinal depigmentation (≥175μm in diameter) and visible choroidal vessels in the absence of neovascular AMD in the same eye (Figure 4B). GA results from continued RPE-loss, which in turn can lead to the development of areas with total loss of the retina, RPE and choriocapillaris (Coleman et al. 2008). GA generally leads to slow progression of visual loss, because photoreceptors are possibly metabolically dependent on the underlying RPE cells (Ambati et al. 2003). GA accounts for 35% of all cases of advanced MD and 20% of legal blindness caused by AMD (Damico et al. 2012).

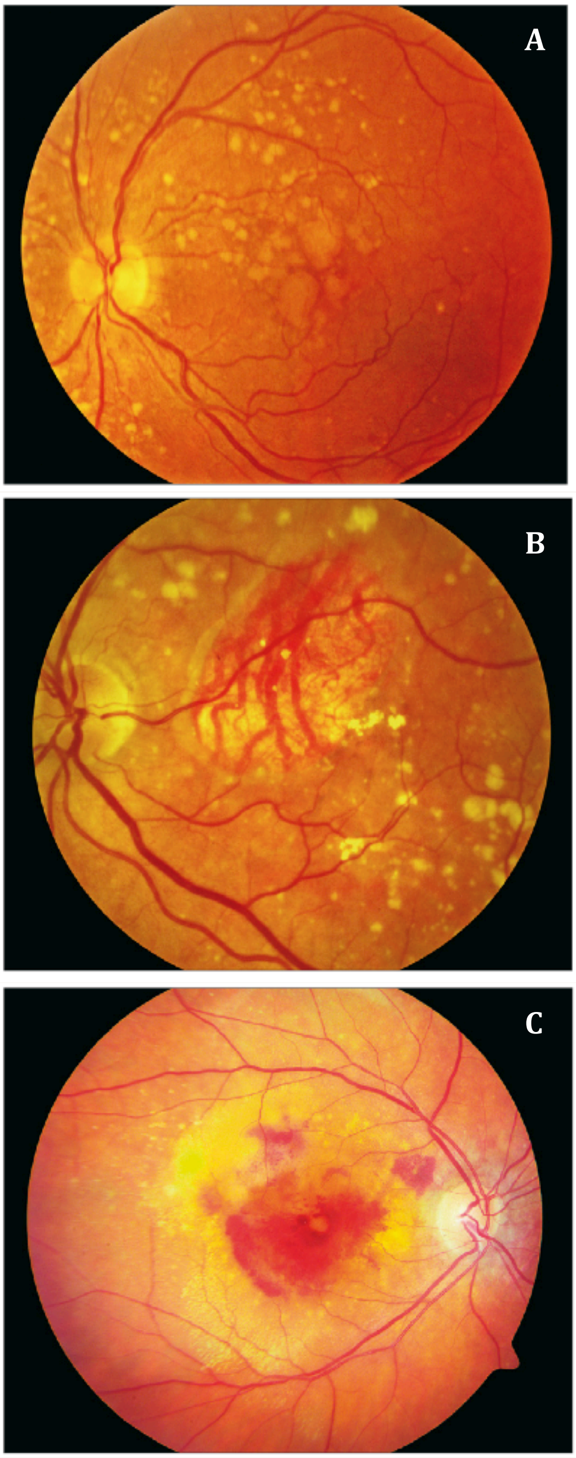

Figure 4 Different types of AMD. A: Left eye of a patient with intermediate age-related macular degeneration with large drusen. B: Geographic atrophy involving the centre of the fovea, with sharply demarcated loss of normal RPE and evidence of deeper larger choroidal vessels. C: Neovascular AMD, with retinal haemorrhage, lipids, or retinal hard exudate and subretinal fluid (Coleman et al. 2008).

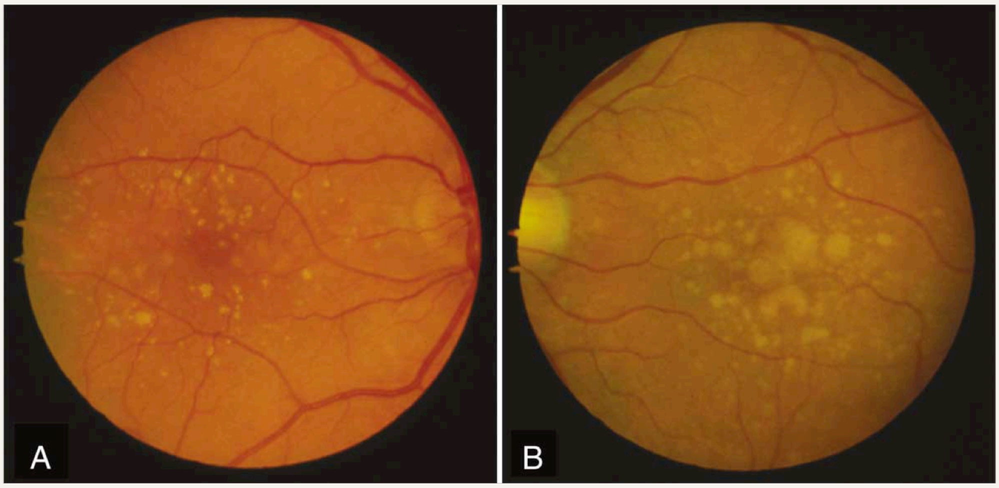

Figure 5 **Comparison of hard and soft drusen.** A: Hard drusen appear as small (<63µm), yellow-white deposits with relatively distinct margins. B: Soft drusen are larger, typically have less distinct borders and have a more diffuse and paler appearance (Hageman et al. 2001)

Neovascular AMD

Neovascular AMD, exudative or 'wet' AMD is the most common cause of severe central visual loss (Vingerling et al. 1995) and the onset of vision loss in neovascular AMD is acute. Choroidal neovascularisation (CNV) refers to the growth of new blood vessels from the choroid and (Figure 4C). In AMD, early neovascularisation can eventually break through the RPE and enter the subretinal space to develop exudative, haemorrhagic or disciform AMD (Ambati et al. 2003, Coleman et al. 2008). Repeated leakage of blood, lipid and serum can lead to fibrovascular and fibroglial tissue and disciform scarring (Ambati et al. 2003, Tombran-Tink and Barnstable 2006). Disciform AMD can cause severe impairment of the outer nuclear layer and can lead to a 70% reduction of photoreceptor length (Kim et al. 2002).

IIc. Detection techniques

Various ocular-imaging techniques have evolved over the past years and are currently being used in the diagnosis of AMD (Rickman et al. 2013). Ocular coherence tomography (OCT) and the fluorescein angiography (FA) are important tools in the examination of retinal diseases and many situations require both imaging techniques for a correct diagnosis and treatment plan (Chhablani and Sudhalkar 2014).

OCT of spectral domain OCT (SD-OCT) is a medical imaging technology similar to ultrasound, and has had a profound impact on early detection, monitoring of progression and treatment efficacy evaluation of wet AMD (Rickman et al. 2013). Highly vascular regions, such as the retinal pigment epithelium (RPE) and choroid, are visible in an OCT image as highly scattering structures (Figure 6) (Fujimoto et al. 2000).

Fluorescein angiography (FA) uses a special dye (fluorescein solution) and camera to visualize the vascular system in the retina and choroid after injection (Chopdar and Aung 2014). FA reveals important pathological features of vascular conditions such as vascular leakage or neovascularization (Pinhas et al. 2013), as CNV characteristically leaks fluorescein (Ambati et al. 2003, Chhablani and Sudhalkar 2014). The fluorescein angiographic leakage patterns of CNV are classified as either 'classic' or 'occult' (Figure 7).

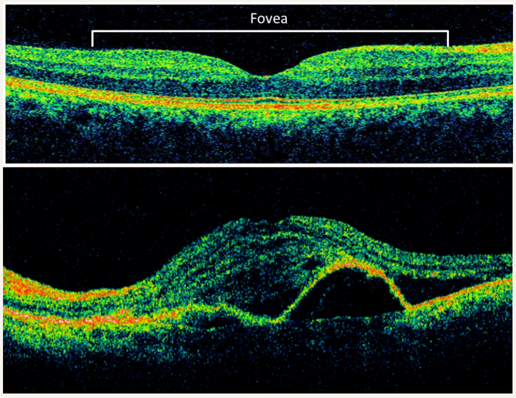

Figure 6 **Ocular Coherence Tomography** images of a normal retina (top) and a retina with pigmented epithelium detachment due to CNV in wet AMD (bottom) (Picture provided by G. Dijkman, LUMC)

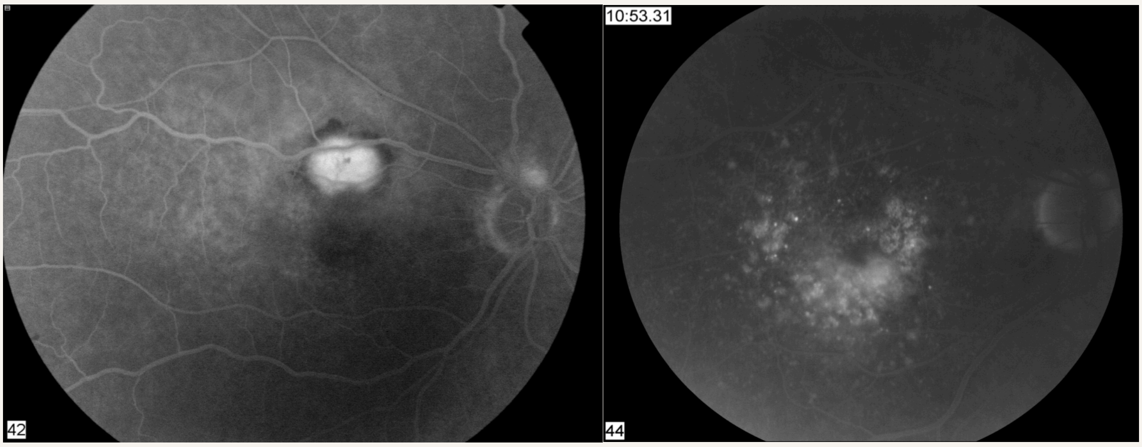

Figure 7 Fluorescent Angiography images
Left: Fluorescein angiogram of a patient with classic CNV, characterized by discrete hyperfluorescent areas. Right: Fluorescein angiogram of a patient with occult CNV, which appears as irregular stippled hyperfluorescent patterns (Ambati et al. 2003) (Picture provided by G. Dijkman, LUMC).

IId. Current therapies

Most of the current therapies and emerging treatments are directed at CNV and can halt the experienced visual impairments. There are currently no established effective treatments for dry AMD (Nowak et al. 2006). At present, there are three acknowledged therapies: thermal laser treatment, photodynamic therapy (PDT) and the intravitreal injection of anti-VEGF medications.

Thermal laser photocoagulation (TLP) uses a laser to cauterize extrafoveal vessels in CNV, halting subretinal fluid accumulation and preventing progression of vision loss. TLP is a simple and relatively inexpensive treatment, suitable for elimination of extrafoveal vessels/lesions. However, the incidence of recurrent and persistent CNV after laser treatment decreases the long-term effectiveness of TLP (Hanout et al. 2013, Nowak 2006).

Photodynamic therapy (PDT) was first approved in 2000 for subfoveal CNV and has since been a widely used treatment with generally positive therapeutic effects. PDT uses light-activated Verteporfin to damage fibrovascular tissue by inducing occlusion of new vessels, thereby temporarily stabilizing the existing leaky blood vessels. This makes PDT only a palliative therapy, which does not prevent the formation of new abnormal leaky vessels (Hanout et al. 2013, Nowak 2006). PDT may sometimes be used in combination with anti-VEGF medications (as described in the NOG guideline for AMD, 2014).

Over the last decade, several anti-VEGF medications have been developed for neovascular AMD. Vascular endothelial growth factor A (VEGFA) has been implicated in CNV and can result in loss of vision. It stimulates endothelial cell growth, promotes vascular permeability and induces dissociation of tight junction components (Zhang et al. 2012). Commonly used anti-VEGF medicines are ranibizumab (Lucentis) and bevacizumab (Avastin) (Figure 8). Ranibizumab and bevacizumab are closely related drugs that target all isotypes of VEGF (Waisbourd et al. 2007) and appear to be highly effective in stabilizing the majority of CNV-cases and even increase vision in a minority of CNV-cases (Singer 2014). Anti-VEGF medication should be used quite early in the onset of the disease (before scar formation has occurred) and should be administered by repeated, monthly intravitreal injections. Ranibizumab and bevacizumab are currently the most common therapies for neovascular AMD.

Ranibizumab (Lucentis) is a recombinant, humanized monoclonal antibody fragment that inhibits all active isoforms of VEGF-A (Hanout et al. 2013). Ranibizumab was approved by the FDA in 2006 and approved in Europa in 2007 (Waisbourd et al. 2007). Treatment with ranibizumab has a good safety profile and is associated with improved vision and decreased leakage from CNV (Nowak 2006). Bevacizumab (Avastin) was originally formulated as an intravenously administered drug for the treatment of metastatic colon cancer in combination with chemotherapy (Waisbourd et al. 2007). Bevacizumab is a full-length humanized monoclonal antibody that targets all isoforms of VEGF-A. In 2006, the cost of a single dose of 0,5mg (0,05mL) Ranibizumab was $1950 (US), whereas bevacizumab costs $17-50 (US) per injection (Waisbourd et al. 2007, Steinbrook 2006). This made bevacizumab a very attractive low-cost alternative treatment for neovascular AMD. The CATT research group was the first to compare the effects of ranibizumab and bevacizumab, and concluded both drugs had equivalent effects on visual acuity at 1 year (Martin et al. 2011). Martin et al. (2012) reported that bevacizumab and ranibizumab had similar effects over a 2-year period. Bevacizumab is currently the most widely used anti-

VEGF agent for treatment of neovascular AMD, due to its low costs, proper treatment schedule and similar efficacy compared to ranibizumab (Hanout et al. 2013 Martin 2011). According to the NOG-guidelines, intravitreal injection of 1,25mg bevacizumab (Avastin) is the first choice of treatment for patients with wet AMD (NOG guideline for AMD, 2014).

Treatment of CNV with radiotherapy has been widely investigated, since ionizing radiation preferentially damages mitotic tissue. Unfortunately conflicting reports, different radiation doses, type of radiation and dose fractions have made this an unsuccessful area up to now (Ambati et al. 2003).

Surgery may have favourable results in highly selected cases of wet AMD, but in general it has shown unimpressive results due to the complexity and risks of the surgery. The unimpressive results have been attributed to the entanglement of RPE with the CNV complex, making it almost obligatory to remove both structures, which leads to the loss of the underlying choriocapillaris (Ambati et al. 2003).

In the field of AMD, there are some new emerging and promising technologies focussing on e.g. small interfering RNA (siRNA) and other VEGF-antagonists like tyrosine kinase inhibitors (Hanout et al. 2013). In the development of possible treatments for dry AMD, a number of medicines are being investigated that utilize different mechanisms of action, e.g. neuroprotection, suppression of inflammation, stem cell replacement and complement inhibition (Singer 2014).

Currently, the use of AREDS-based vitamin supplements is the only approved treatment for dry AMD. It does not halt the vision loss, but may lower the risk of developing advanced stages of AMD and reduces visual loss in people at risk for the disease (Damico et al. 2012). According to the 2014 NOG guidelines, the AREDS-supplementation should be recommended to patients with intermediate or advanced AMD in one or both eyes (as described in the NOG guideline for AMD, 2014).

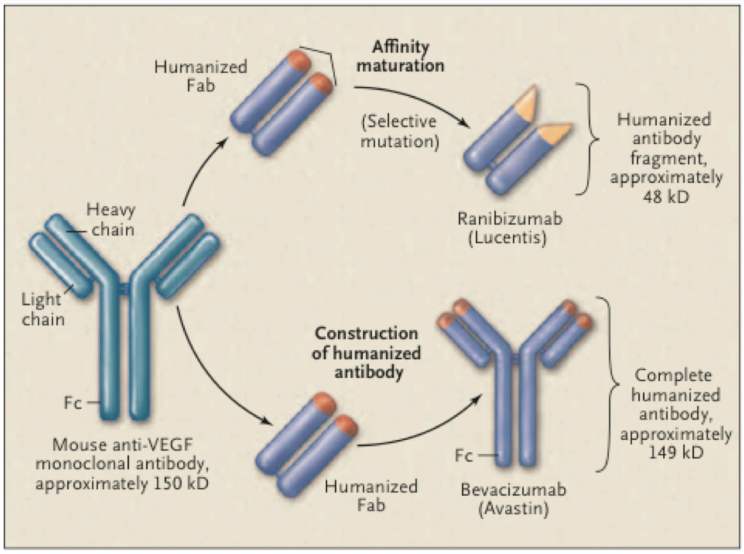

Figure 8 Schematic picture of the humanized antibodies Lucentis and Avastin (Steinbrook 2006)

III. PATHOGENIC MECHANISMS IN AMD

The following key processes are likely to play a role in AMD pathology: oxidative damage, lipofuscin accumulation and impaired function of RPE, increased apoptosis, abnormal immune system activation, senescent loss of homeostatic control and abnormalities in Bruch's membrane (Figure 9) (Zhang et al. 2012). Also, several risk-alleles associated with AMD have been identified in the past years.

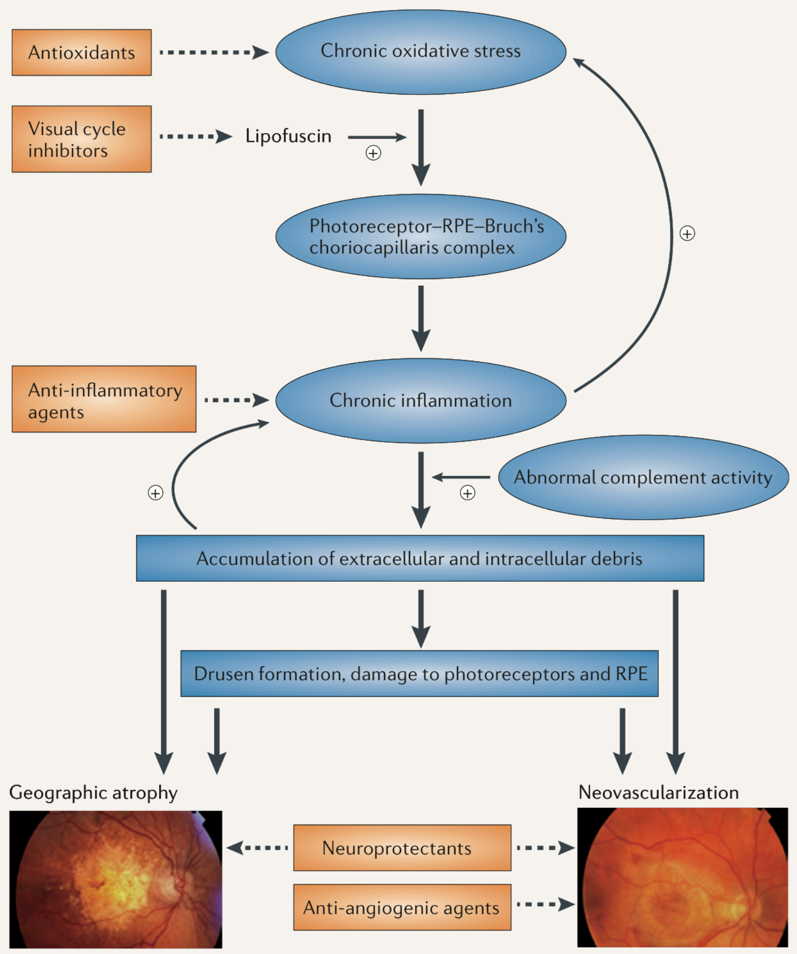

Figure 9 Proposed pathophysiology of AMD and locations in the pathway in which different therapeutic interventions might be effective (Zhang et al. 2012)

IIIa. Genetics

Both environmental and genetic factors play a role in the development of AMD (Buentello-Volante et al. 2012). A study by Klaver et al. (1998) showed that first-degree relatives of AMD-patients were three times more likely to develop wet AMD than control, and that more than 20% of the proportion of late AMD in the population could be attributed to genetic factors. The genetic heritability of AMD is estimated from 46% up to 71% (Seddon et al. 2005). However, only about 40% of the genetic variance of AMD can be explained by the genetic variants known to date (Sobrin et al. 2010).

In recent years, great advances have been made in the identification of several genetic regions that are involved in AMD pathogenesis. Among these are polymorphisms in proteins like complement factor H (CFH), complement component 2 (C2), complement component 3 (C3), complement factor B (CFB) and age-related maculopathy susceptibility 2 (ARMS2). Single nucleotide polymorphisms (SNPs) coding for CFH Y402H, ARMS2 A69S, and C3 R102G account for approximately 76% of the population-attributable risk of the development of AMD (Buentello-Volante et al. 2012), suggesting these three genetic variants are the most important in the AMD pathogenesis.

CFH, a serum glycoprotein which downregulates the activity of the alternative complement pathway, can be found in normal human RPE, Bruch's membrane and choroid (Buentello-Volante et al. 2012, Ding et al. 2009, Coleman et al. 2008). The CFH gene is located on chromosome 1q32. The alternative pathway of the complement system mediates antibody-independent recognition of pathogens and defence against

microbial infections (Johnson et al. 2006). CFH binds to C3b, stimulating the decay of the alternative pathway convertase C3b-Bb, or acts like a cofactor for complement factor I, another C3b inhibitor (Buentello-Volante et al. 2012). CFH dysfunction may lead to excessive inflammation and tissue damage involved in the pathogenesis of AMD (Johnson et al. 2006), but CFH is also suggested to mediate drusen formation (DeWan et al. 2006). The Y402H polymorphism in the CFH gene shows a very strong association with late AMD, especially in homozygous individuals (Buentello-Volante et al. 2012, Seddon et al. 2007). Other factors such as CFB, C2 and C3 also play central roles in the activation of complement pathway systems, indicating that AMD might involve a major inflammatory component (Coleman et al. 2008).

Another important locus that has been associated with both neovascular AMD and GA is LOC387715(ARMS2)/HtrA1 (high temperature requirement factor A1), located on chromosome 10q26 (Coleman et al. 2008, Seddon et al. 2007). These two genes seem to have different functions and expression patterns in the retina, but they are located extremely close and in strong linkage disequilibrium (Ding et al. 2009). Several studies have investigated the functional implications of the variants rs10490924 (ARMS A69S) and rs11200638 (HtrA1) and their association with AMD (Fritsche et al. 2008, Katta et al. 2007). The exact biological functions of ARMS2 and HtrA1 are still unclear, but they may contribute to AMD development through their effect on precursors, such as drusen or changes in RPE and Bruch-membrane (Coleman et al. 2008). HtrA1 is a secretory protein and inhibitor of the transforming growth factor β (TGF-β). The rs11200638 allele of HtrA1 has shown to cause increased expression of HtrA1 in AMD patients (DeWan et al. 2006, Seddon et al. 2007). A study showed that the ARMS2 protein localizes to the outer membrane of mitochondria, suggesting the ARMS2 A69S (rs10490924) variant might play a role in AMD through mitochondria-related pathways (Katta et al. 2009, Fritsche et al. 2008).

Variations in C3 have been associated with increased risk for AMD, of which the R102G polymorphism appears to be strongest AMD-associated variant. The R102G polymorphism is responsible for a smaller, but still substantial, portion of the AMD-cases in comparison to the CFH Y402H and LOC386615/ARMS2 A69S variants. The AMD-associated genes CFH and CFB are known to target the alternative complement cascade, of which C3 is a major component (Spencer et al. 2008). The R102G polymorphism generates the 'fast' and 'slow' electrophoretic allotypes of C3 (C3F and C3S). These allotypes affect binding to monocyte-complement receptor C3F, which is a risk variant for AMD (Buentello-Volante et al. 2012, Spencer et al. 2008). A study by Caire et al. (2014) suggested that the C3 R102G variant may play an important role in GA progression, but their findings were only able to show a tendency and no statistical significance was found.

IIIb. Oxidative stress

Oxidative stress (OS) is believed to be a key player in the initiation and progression of several ocular diseases, including AMD (Pinazo-Durán et al. 2014a, Tokartz et al. 2013, Justilien et al. 2007). OS results from the imbalance between oxidants and antioxidants - in favour of oxidants - leading to cellular damage and death caused by reactive oxygen species (ROS) (Tokartz et al. 2013, Pinazo-Durán et al. 2014a). ROS are partially reduced metabolites, including oxygen free radicals, hydrogen peroxide, singlet oxygen and their respective metabolic by products. Free radicals are molecules that contain unpaired electron(s) or have an open electron shell (Beatty et al. 2000). The chain reactions of ROS include hydrogen peroxide (H_2O_2), superoxide anion ($O_2{\bullet}^-$) and hydroxyl radical (•OH) (Figure 10). Singlet oxygen (O_2) and hydrogen peroxide (H_2O_2) have no unpaired electrons, but are in an unstable and reactive state (Beatty et al. 2000). ROS are by products of cellular metabolism and photochemical reactions (Ambati et al. 2003). Normally, radicals are effectively scavenged by cellular antioxidant defence systems, e.g. macular pigments, making their presence harmless (Tokartz et al. 2013).

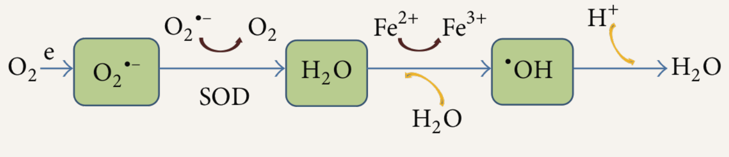

Figure 10 The chain reactions of reactive oxygen species (Pinazo-Durán et al. 2014a)
Abbreviations: e: electron, SOD: superoxide dismutase, Fe: iron

The retina is particularly vulnerable to oxidative stress because of its high polyunsaturated fatty acids (PUFAs) concentration in the photoreceptor outer segments (POS), elevated oxygen tension, high exposure to light and thus irradiation, presence of many chromophores (photosensitive compounds e.g. lipofuscin) and the generation of ROS by photoreceptor phagocytosis conducted by the RPE (Ambati et al. 2003, Ding et al. 2009, Tokartz et al. 2013). The PUFAs in cell membranes make them the main target for ROS induced damage, as their double bounds are an electron-source (Beatty et al. 2000). RPE cells are postmitotic (Campisi and d'Adda di Fagagna 2007). Therefore, damage in RPE cells accumulates during their life span and increases with age. The increasing concentration of ROS may cause damage to organelles, including lysosomes and mitochondria (Tokartz et al. 2013).

Macular pigments protect the macula against oxidative damage by constituting an optical filter that absorbs short-wavelength visible light (Tokarz et al. 2013, Pinazo-Durán et al. 2014a). The majority of light is absorbed by melanin, present in melanosomes. The remaining light is mainly absorbed by the hydroxycarotenoids, lutein and zeaxanthin (Tokartz et al. 2013). AMD patients have considerably less macular pigment in their eyes and therefore a greater risk of oxidative damage compared to healthy eyes (Wu et al. 2006). As mentioned before, AMD is more prevalent in the white population. It has been hypothesized that an increased amount of choroidal melanin in black patients' eyes could have a protective effect on the RPE, photoreceptors and Bruch's membrane, possibly through an antioxidant effect of its ability to absorb light rays that damage the posterior layers of the retina. Pigment or other factors in darkly pigmented RPE or choroid may also have an inhibitory effect on leakage, migration and proliferation of endothelial cells (Jampol and Tielsch 1992). Blue iris colour has also been implicated as another risk factor for AMD, because of lower pigment content in the retinal pigment epithelium compared to other iris colours (Coleman et al. 2008).

PUFA oxidation can lead to additional ROS generation in the retina. The major PUFA in the retina is docosahexaenoic acid (DHA22:6ω-3). In normal conditions, the RPE constantly phagocytizes the POS membranes, but in AMD the oxidized PUFAs are not correctly cleaved in the lysosomes of the RPE cells and therefore accumulate in the form of lipofuscin (Pinazo-Durán et al. 2014b, Blasiak et al. 2013). This is thought to be important in the formation of drusen (Beatty et al. 2000). Lipofuscin mainly consists of lipids, proteins and pigment derivates such as N-retinylidene-N-retinylethanolamine (A2E) (Tokartz et al. 2013). It is deposited into insoluble aggregates in RPE cells and functions as a photosensitiser, thereby evoking and enhancing OS in the retina (Pinazo-Durán et al. 2014b, Blasiak et al. 2013). Because lipofuscin accumulates with age, it is referred to as an 'age pigment' and considered a marker of cellular biological aging (Beatty et al. 2000).

Blue light seems to be the most dangerous to the RPE, since this light is the most energetic radiation reaching the RPE and it promotes photo-oxidation of lipofuscin. Photo-oxidation of lipofuscin generates reactive products including A2E, cell apoptosis and DNA oxidation (Sparrow et al. 2000). A2E is known to be an initiator of blue-light induced apoptosis in RPE cells (Sparrow et al. 2000). A2E accumulation leads to dysfunction of lysosomes in a dose dependent manner (Tokartz et al. 2013) and the generation of singlet oxygen may be involved in the mechanisms leading to apoptosis of A2E-containing RPE cells (Sparrow et al. 2002).

Mitochondria are major sources of ROS, as ROS are produced in their electron transport chain (Tokartz et al. 2013, Blasiak et al. 2013). Mitochondrial DNA (mtDNA) is more susceptible to oxidative damage than nuclear DNA (nDNA), because of its lack of protection by histones or other proteins, the lack of introns in some regions, high transcription rate and the less effective mtDNA repair systems in comparison to nuclear DNA (Blasiak et al. 2014). For these reasons, mtDNA rapidly accumulates mutations leading to generation of ROS (Cui et al. 2012). Increased ROS damages lipids, proteins and nucleic acids. A study by Blasiak et al. (2013) showed an increase in mtDNA damage and mutations, higher sensitivity to H_2O_2 and UV-radiation and a decrease in DNA repair efficacy in AMD patients. Their data suggested that the cellular response to both mtDNA and nDNA damage may be involved in AMD pathogenesis and that mtDNA accumulates more DNA lesions than nDNA in AMD. A study by Justilien et al. (2007) showed that knockdown of manganese superoxide (mnSOD), an antioxidant mitochondrial enzyme involved in replication and repair of mtDNA (Bakthavatchalu et al. 2012), stimulates long-term mitochondrial OS, increased $O_2^{\bullet-}$ and apoptosis, degeneration of RPE cells, thickening of Bruch's membrane, shortening and disorganisation of photoreceptor segments.

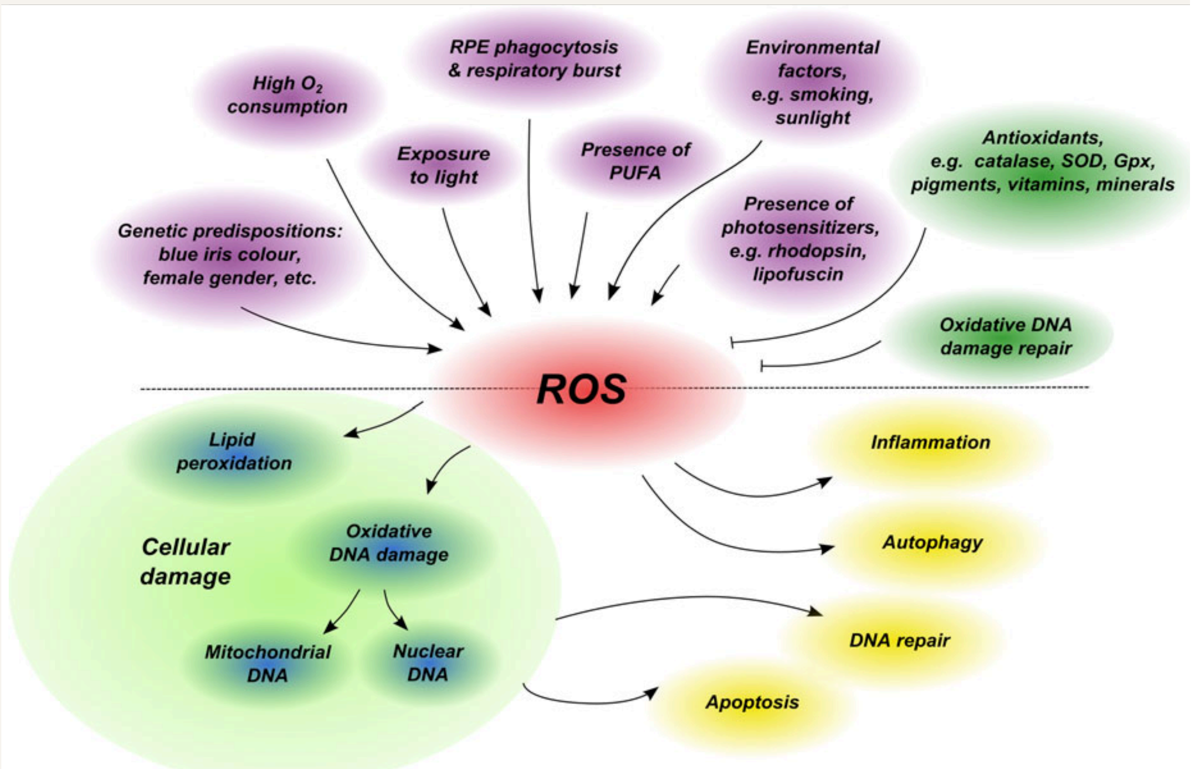

Figure 11 Schematic presentation of ROS involvement in AMD pathology (Tokartz et al. 2013)

IIIc. Apoptosis

Apoptosis, or programmed cell death, is a highly ordered and regulated cell suicide pathway that is essential for normal development and cell survival. In apoptosis, the permeability of the mitochondrial membrane increases and induces release of proapoptotic factors into the cytosol, such as procaspases, caspase activators and caspase-independent factors. This leads to cell death (Pinazo-Durán et al. 2014a). In apoptosis, chromatin is typically fragmented and caspase enzymes degrade the cell. Fragmented chromatin can be detected by the terminal deoxynucleotidyl transferase dUTP nick end labelling (TUNEL)-technique, labelling the terminal ends of nucleic acids (Negoescu et al. 1996). A study by Dunaief et al. (2002) suggested that cells of the RPE, photoreceptors and inner nuclear layer die by apoptosis in AMD. Their results showed a significant increase in TUNEL-positive cells in the inner choroid, RPE, photoreceptors and inner nuclear layers in macula's with AMD. Most TUNEL-positive RPE and photoreceptor cells were present near the edges of RPE and photoreceptor atrophy, the area predicted to be at risk of cell death. Moreover, photoreceptors in AMD eyes upregulated Fas, a mediator of apoptosis, suggesting the Fas/FasL may be involved in photoreceptor apoptosis.

Jun kinases (JNKs) may play a key role in the development of CNV (Du et al. 2013). JNKs regulate cell proliferation, migration, survival and cytokine production and can be activated by ROS (Kamata et al. 2005). JNK1 is involved in cell stress responses, apoptosis, inflammation and VEGF production. JNK1 deficiency or JNK inhibition leads to a decrease in apoptosis, VEGF expression and reduction of CNV in a murine model of wet AMD (Du et al. 2013). JNK inhibition might be a promising target in future treatment strategies for AMD.

Antioxidants and free radical scavengers have been demonstrated to inhibit or delay apoptosis (Matés 2000 and Salganik 2001), indicating ROS may be involved in the signal transduction pathways involved in apoptosis (Figure 12) (Pinazo-Durán et al. 2014a). ROS has been suggested to result in apoptosis of retinal ganglion cells in AMD eyes (Dunaief et al. 2002).

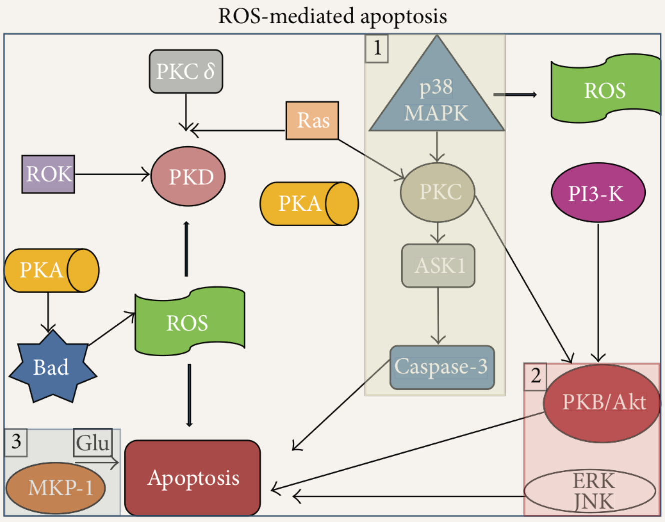

Figure 12 ROS-mediated apoptosis Cells have a wide spectrum of intracellular signal transduction systems to transmit ROS-mediated signals and accommodate to the OS. These systems include protein kinase cascades and many of these pathways are engaged in the route to apoptosis, including MAP kinases/ASK1 upstream regulator (1), PKB/Akt-ERK/JNK (2), MKP/1 and glutamate-induced cell death (3) (Pinazo-Durán 2014a).

IIId. Angiogenesis

Angiogenesis is the development of new capillaries from pre-existing vessel networks, which plays an important role in embryonic development, somatic growth and tissue repair (Ambati et al. 2003). However, angiogenesis can also be harmful and is involved in many diseases, including neovascular AMD. The key regulators of angiogenesis are the vascular endothelial growth factor (VEGF), pigment epithelium-derived growth factor (PEDF), fibroblast growth factor 2 (FGF2), angiopoietins and extracellular matrix (ECM) molecules. VEGF appears to serve as the molecular switch for a variety of neovascular conditions, because of its actions on the proliferation and survival of endothelial cells, vascular permeability and ocular inflammation (Table 1) (Eugene and Adamis 2005, Pinazo-Durán et al. 2014a).

Endothelial cells are particularly susceptible to exogenous and circulatory agents (Pinazo-Durán et al. 2014a). VEGF is a member of the platelet derived growth factor (PDGF) family that can activate endothelial cell growth and induce angiogenesis (neovascularization) by causing a variety of signalling cascades in the capillaries of the retina and choroid (Nakao et al. 2012, Eugene and Adamis 2005). The Blue Mountains Eye Study suggests that the relative risk for neovascular AMD among women is double that of men (1,2%vs.0,6%) (Mitchell et al. 2002). Some models suggest that female animals are more susceptible to CNV-induction, because of higher expression of kinase insert domain receptor (KDR), which is a receptor for VEGF (Tanemura et al. 2001).

PEDF functions as a neurotrophic factor and a potent angiogenic inhibitor in the retina by downregulating VEGF. It has been shown that there is a balance between VEGF and PEDF in the retina, which plays a critical role in the regulation of vascular permeability and angiogenesis (Zhang et al. 2006, Bhutto et al. 2005). In AMD, PEDF levels are significantly lowered in RPE cells, RPE basal lamina, Bruch's membrane and choroidal stroma (Bhutto et al. 2005). This decrease in PEDF in the retina is partially responsible for an increase in VEGF expression and disruption of the VEGF/PEDF balance (Gao et al. 2001). This disturbed balance correlates with the formation of CNV in AMD (Zhang et al. 2006, Bhutto et al. 2005).

Studies investigating the role of VEGF in ocular angiogenesis have led to the design of new therapeutic strategies for angiogenic eye disorders, such as anti-VEGF agents. As PEDF has been shown to protect the RPE and photoreceptors against cell death from various pathological insults, Popp et al. (2013) hypothesized that intravitreal injection of PEDF may decrease AMD-lesion progression in the eye. Their results showed that PEDF had a healing effect on AMD-lesions by its anti-inflammatory, anti-apoptotic and neuroprotective role and that PEDF may be a new potential treatment therapy for AMD.

Table 1 Properties of VEGF (Eugene and Adamis 2005)

Properties of VEGF
- Stimulator of angiogenesis
- Potent inducer of vascular permeability and fenestration
- Proinflammatory agent
- Neuroprotective agent
- Vessel survival factor

IIIe. Inflammation

The immune-inflammatory response (IIR) attempts to defend an organism from cell injury and its related effects. The IIR involves cells of the innate immunity (phagocytic cells such as neutrophils, monocytes, macrophages and NK cells) and cells of the adaptive immunity (lymphocytes T, B, and NK), interacting among them by cytokines, chemokines, nitric oxide (NO) etc. (Pinazo-Durán et al. 2014a). Antigen-presenting cells (APCs) function as a 'bridge' between the innate and adaptive immune response (Gourbeyre et al. 2009). Tissue damage results from uncontrolled chronic inflammation (Figure 13).

Increased inflammatory plasmatic markers and immune mediator molecules, such as TNF-alpha, E-selectin and interleukin 6 are positively correlated with age and contribute to a pro-inflammatory environment that helps to develop vascular dysfunction and promotes endothelial apoptosis (Ungvari et al. 2007). AMD lesions demonstrate signs of persistent chronic inflammatory damage, including infiltration and recruitment of macrophages, complement activation and pro-inflammatory cytokines or chemokines in AMD lesions and drusen. Furthermore, AMD occurrence is associated with microglial activation and accumulation in the macula (Ding et al. 2009, Pinazo-Durán et al. 2014a).

Drusen contain lipids, proteins and oxidation products for lipids and carbohydrates. These oxidative components in drusen, as well as retinal lesions in advanced AMD, can activate pattern recognition receptors that initiate an inflammatory and immune response, such as macrophage recruitment, microglial accumulation, complement activation, release of cytokines and chemokines and inflammatory oxidative stress (Tuo et al. 2012). Altered cytokine profiles of the RPE have been associated with the aging eye and AMD (Pinazo-Durán et al. 2014a). A study by Anderson et al. (2002) supported a role for local inflammation in drusen biogenesis. Their results indicated that cellular remnants and debris derived from degenerated RPE cells become isolated between the RPE basal lamina and Bruch's membrane. This cellular debris may constitute a chronic inflammatory stimulus and a potential 'nucleation' site for drusen formation. Hageman et al. (2001) reviewed the role of inflammatory, immune-, and cell-mediated events in drusen biogenesis. In their model, injured RPE serves as the most likely source of soluble cytokines or other stimulatory factors that recruit and activate dendritic cells (Figure 14).

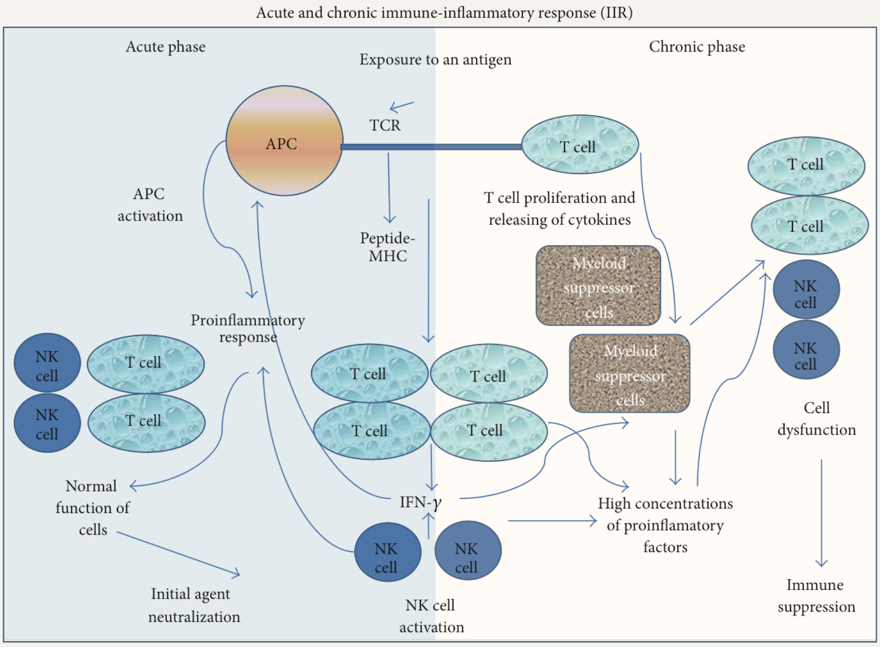

Figure 13 **The inflammatory-immune response (IIR)** Cytokines, interleukins (IL) IL-1 and IL-6 and TNF-alpha are inducers of the IIR through the regulation of monocytes. Macrophages, neutrophils, fibroblasts and endothelial cells are essential for an adequate immune system function. Uncontrolled chronic inflammation can cause tissue damage (Pinazo-Durán et al. 2014a)

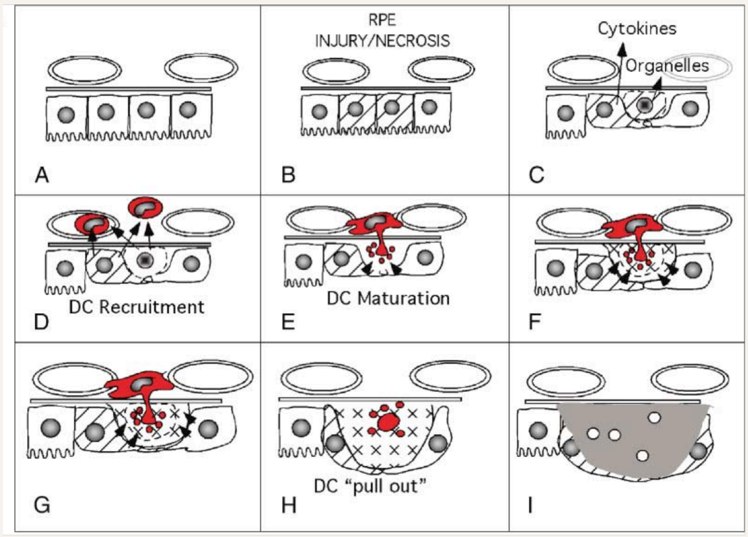

Figure 14 The integrated hypothesis proposed by Hageman et al, depicting the role of inflammatory-, immune- and cell-mediated events in drusen biogenesis. A: RPE, Bruch's membrane and choroid in a normal condition. B: RPE injury caused by gene mutations, light damage, oxidative stress, lipofuscin accumulation, complement mediated cell injury etc. Damage results in cytokine release and/or RPE 'debris' into Bruch's membrane. C: Adjacent RPE cells form a seal over the RPE debris and synthesize a novel basal lamina. D: Soluble molecules are released by injured RPE and serve as chemoattractants for choroidal or blood lymphocytes. The latter migrate to the site of injury (lesion) and develop into mature dendritic cells E: Formation of the drusen 'core'. F/G: Response of the underlying RPE, perhaps to reduce the growth of the druse. H: the mature choroidal dendritic cells withdraw their drusen-associated processes and migrate away from the lesion, I: leaving behind a composition of extracellular material (druse) (Hageman et al. 2001).

Pathogenic mechanisms of AMD related to inflammation include variations in complement factors, like CFH, CFB, C2 and C3 (Coleman et al. 2008). The complement system is part of the innate immune system and can be activated through three distinct pathways: the classical pathway, involving antigen-antibody complexes and complement, the alternative pathway, and lectin-mediated pathway, leading to the destruction of foreign proteins or damaged cells by the host defence system (Anderson et al. 2002, Donoso et al. 2006). As previously described, CFH plays a critical role in the alternative pathway of the complement system by binding and inactivating C3b (Johnson et al. 2006, Buentello-Volante et al. 2012). C3b is deposited on intact host cells, thereby preventing destruction of these cells but allowing destruction of foreign or damaged host cells. CFH dysfunction may lead to excessive inflammation and tissue damage involved in the pathogenesis of AMD. The Y2402H residue is located within the CFH complement activation locus, a site strongly associated with AMD (Figure 15) (Donoso et al. 2006, Seddon et al. 2007, Johnson et al. 2006).

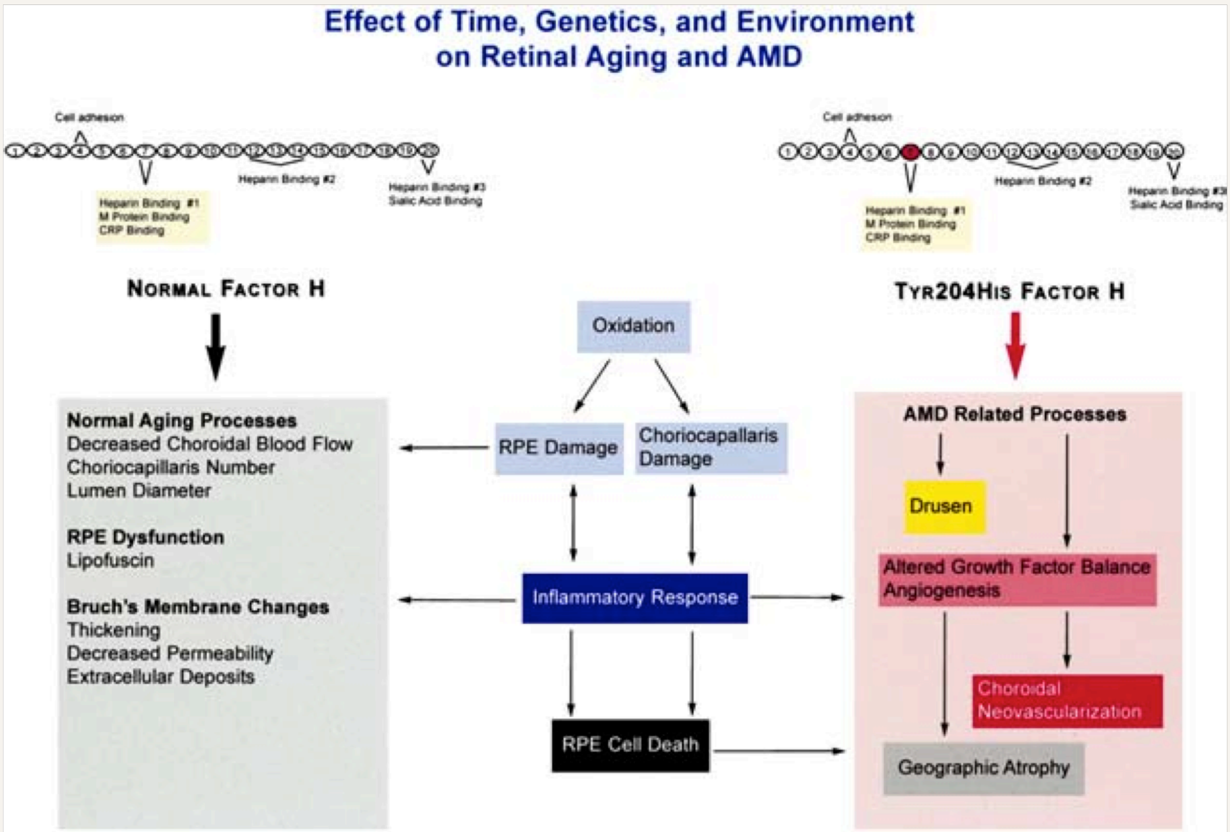

Figure 15 Complement Factor H and the effects of time, genetics and environment on retinal aging and AMD (Donoso et al. 2006)

IV. NUTRITIONAL SUPPLEMENTS AND AMD

The first study that evaluated the relationship between dietary intake, nutritional factors and AMD was performed in the Eye Disease Case-Control Study (EDCCS) in 1986 (Seddon et al. 1994). The hypothesis of this study was that antioxidants dietary and nutritional factors could influence AMD. Their hypothesis was based on the known influence of daily insults on free radical formation and oxidation, and on the assumption that the retina was a set up for these oxidative processes due to the abundance of PUFAs in the POS membranes.

IVa. Antioxidants (vitamins A, C and E)

The deposit of oxidized compounds in healthy tissue could lead to cell death and could theoretically lead to impaired function of the RPE and eventually to degeneration involving the macula. A review by Sobrin and Seddon (2014) proposed that dietary antioxidants could potentially block this damaging effect of oxidation and scavenge, decompose or reduce the formation of harmful compounds in the macula.

Vitamins (A, B, C and E)

Vitamin E is an antioxidant that exists in several forms in nature, of which the α-tocopherol is the most abundantly concentrated in both plasma and retinal tissue (Olson et al. 2011). Vitamin E is the major lipid-soluble antioxidant protecting lipids against peroxidative damage in plasma and red blood cells (Sies and Stahl 1995). Vitamin C, also known as L-ascorbic acid, is considered the most important antioxidant in extracellular fluids (Sies et al. 1992) and functions as a cofactor in the regeneration of Vitamin E in the retina. Vitamins C and E were included in the AREDS trials. AREDS reported a benefit from vitamin C and E, but this was in combination with the other supplements contained in the AREDS formulation. Vitamin C and E alone were not found to significantly affect the course of AMD (AREDS Research Group 2001). Two large, randomized controlled trials assessing the role of vitamin E in AMD prevention showed no effect (Teikari et al. 1998, Taylor et al. 2002). Interestingly, a meta-analysis combining both trials found a significant protective effect of Vitamin E in the prevention of early AMD (Chong et al. 2007). Yet, the overall conclusion of Chong et al. stated there was insufficient evidence to support the role of dietary antioxidants for the prevention of early AMD. The study by Seddon et al. (1994) investigated the effects of vitamins A, C and E and their results showed that intake of preformed vitamin A (retinal) was not associated with AMD and there was no significant association between vitamin C and E consumption and AMD. However, the researchers did suggest a possible lower risk for AMD among those with higher dietary intake of vitamin C. Although vitamin supplements are generally regarded as safe, they may have harmful effects. A high dose of vitamin A seems to be associated with increased risk of mortality, congestive heart failure, prostate cancer, and β-carotene with an increased risk of lung cancer among active smokers (Pinazo-Durán et al. 2014a). In AREDS, skin and subcutaneous tissue conditions (yellow skin) were more frequent in the antioxidant arms (P=0,03) (AREDS Research Group 2001).

Vitamin B is another antioxidant that is hypothesized to have a protective effect in the human retina (Olson et al. 2011). Hyperhomocystein is thought to induce vascular endothelial dysfunction, which may play a role in development of neovascular AMD (Axer-Siegel et al. 2004). Vitamin B supplementation has shown to decrease serum levels of homocystein, (Seddon et al. 2006b, Kamburoglu et al. 2006) and could thereby have a protective macular function. The Women's Antioxidant and Folic Acid Cardiovascular Study (WAFACS) showed an unexpected benefit of AMD development in women who received a combination of B-vitamins (Christen et al. 2009). However, this study had a number of limitations and further investigation into the role of vitamin B in AMD risk is needed.

Carotenoids (β-carotene, lutein, zeaxanthin)

Carotenoids are macular pigments that absorb light energy and act as antioxidants. There are over 600 known carotenoids, but only a few can be found in the human body (Olson et al. 2011). Lutein and zeaxanthin are the only two carotenoids present in the retina and are the main components of the human macular pigment, and are highly concentrated in the macula (Olson et al. 2011). Carotenoids reduce the amount of light reaching the photoreceptors by approximately 40% (Tokartz et al. 2013).

The carotenoid β-carotene is not present in the macula (Seddon et al. 1994), but since lutein and zeaxanthin have only been available for manufacturing in a research formulation for a few years, several studies have investigated the effect of β-carotene in AMD. However, in the study by Seddon et al. (1994) a higher dietary intake of β-carotene did not reduce the risk of advanced AMD. Several other studies demonstrated an increased incidence of lung cancer and mortality in smokers assigned to β-carotene supplementation (Omenn et al. 1996, ATBC Cancer Prevention Study Group 1994b).

The association between AMD risk and lutein and zeaxanthin supplementation has been explored in several large-scale epidemiological studies and prospective randomized controlled trials. Lutein and zeaxanthin can primarily be obtained from dark green, leafy vegetables (Seddon et al. 1994). Gale et al. (2003) studied 280 man and women and found that lower plasma levels of zeaxanthin could be associated with an increased risk of early AMD. The results of Seddon et al. (1994) showed that participants in the highest quintile of lutein and zeaxanthin intake (6mg) had a 43% lower risk for AMD compared to those in the lowest quintile. In 2006, the Carotenoids in Age-related Macular Degeneration Study (CAREDS) concluded that lutein- and zeaxanthin-rich diets may protect against intermediate AMD in female patients less than 75 years of age (Moeller et al. 2006). The Blue Mountain Eye Study reported that higher dietary lutein and zeaxanthin intake reduced the risk of incident AMD over 5 and 10 years (Tan et al. 2008). A large randomized controlled trial by Richer et al. (2004) reported that lutein had a protective effect in slowing the progression of AMD when used alone or in combination with other nutritional supplements (zinc, β-carotene, vitamin C and E). However, there are also studies that did not report any statistically significant correlation between lutein and AMD (Bartlett and Eperjesi 2007).

IVb. Zinc

High concentrations of zinc can be found in ocular tissues, particularly in the retina, pigment epithelium and choroid (Newsome et al. 1988). Zinc is a co-factor for many metabolically active enzymes within the eye, including superoxide dismutase (SOD) and catalase, which are important in protecting the retina from oxidative damage. It also fulfills a role as co-factor in the metabolism of vitamin A and in rhodopsin synthesis (Olson et al. 2011, Newsome et al. 1988). Zinc interacts with taurine and vitamin A in the retina, modifies plasma membranes in the photoreceptors, regulates the light rhodopsin reaction within the photoreceptor, modulates synaptic transmission and serves as an antioxidant in both the RPE and retina (Figure 16) (Grahn et al. 2001). Elderly are at higher risk of zinc deficiency, which might increase their risk of vision loss in AMD (Olson et al. 2011, Newsome et al. 1988).

Multiple studies have investigated the effects of zinc on AMD. However, studies have reported inconsistent associations between zinc and AMD. Reports range from zinc having no significant relationship with AMD, to serving a protective role. The Blue Mountains Eye Study initially reported no significant association between zinc intake and patients (Flood et al. 2002), but their 10-year data showed that high zinc intake significantly reduced the development of early AMD in individuals (Tan et al. 2008). AREDS did not find a statistically significant effect of zinc on the rates of AMD when zinc was analyzed individually (AREDS Research Group, 2001). AREDS also provided evidence on adverse effects of zinc. The main adverse effect was that people taking high-dose zinc were at increased risk for hospital admission due to genitourinary diseases or experienced circulatory adverse effects.

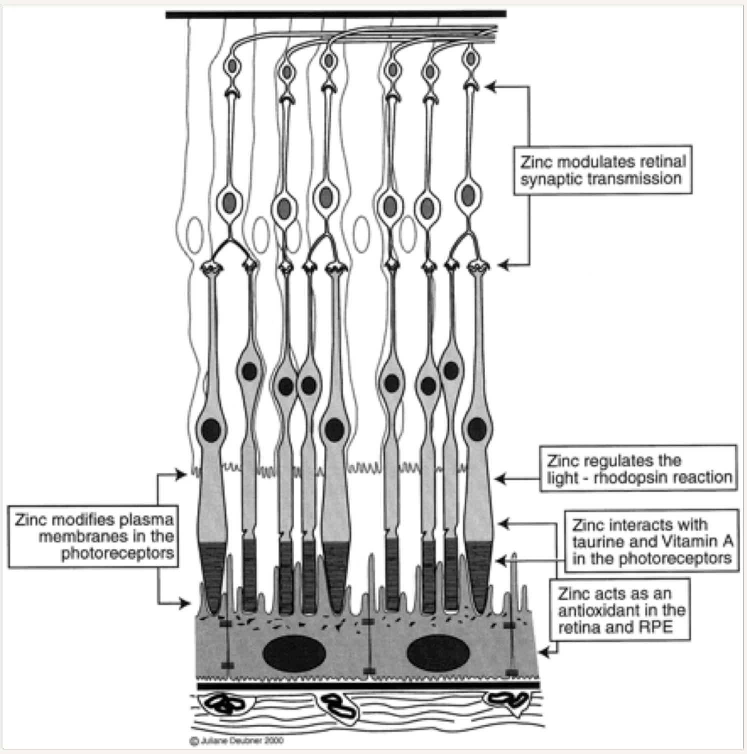

Figure 16 The functions of zinc in the retina and retinal pigment epithelium (Grahn et al. 2001)

IVc. Omega 3 fatty acids

Omega-3 fatty acids have been proposed to alter retinal cell membranes and confer protection against oxidative, inflammatory and vasogenic processes that play a key role in the pathogenesis of AMD (Evans and Lawrenson 2014). Docosahexaenoic acid (DHA) and its precursor, eicosapentaenoic acid (EPA), are examples of omega-3 long chain polyunsaturated acids (ω3-LCPUFA) (Olson et al. 2011). DHA accounts for 50-60% of the total fatty acid content of the outer segments of photoreceptors (Evans and Lawrenson 2014). DHA plays a key role in the homeostasis and function of photoreceptors and RPE (Pinazo-Duran et al. 2014b) and helps regulate gene expression, thereby initiating antioxidant, neuroprotective, anti-inflammatory and anti-angiogenic effects (Ramkumar et al. 2013, Mukherjee et al. 2007, Block et al. 2012). EPA might be involved in vascular permeability, inflammation, retinal neovascularization and suppression of VEGF (SanGiovanni and Chew 2005). The constant turnover of outer segment membranes requires continuous dietary supply of DHA and its precursors, since humans are unable to synthesize them de novo (Olson et al. 2011). A deficiency of ω3-LCPUFA may predispose the development of AMD.

Studies have reported that the consumption of ω3-LCPUFA and fish products could reduce the risk of developing advanced AMD. For example, Chua et al. (2006) and Cho et al. (2001a) reported a reduced risk of early AMD with higher intake of ω3-LCPUFA. A recent population-based cross-sectional study by Augood et al. (2008) identified an inverse association between the rate of dietary DHA and EPA consumption with neovascular AMD. Their results showed a 50% reduction in the risk of neovascular AMD in individuals that consumed oily fish at least once per weak, compared to those consuming oily fish less than once per week (Augood et al. 2008). A meta-analysis by Chong et al. (2008) pooled data from six observational studies and reported a 38% reduced rate of progression to late AMD in participants consuming the highest amount of dietary ω3-LCPUFA (Olson et al. 2011). However, although the authors found an inverse association in the progression of AMD, they concluded there was insufficient evidence from current literature to support a routine consumption of ω3-LCPUFA for AMD prevention (Chong et al. 2008). The Nutritional AMD Treatment 2 (NAT-2) study was a randomised controlled trial that specifically investigated whether ω3-LCPUFA supplementation could decrease the risk of developing advanced AMD. However, the trial provided high quality evidence that people taking ω3-LCPUFA supplements were not at a decreased or increased risk of developing advanced AMD (Souied et al. 2013, Evans and Lawrenson 2014).

Table 2 summarizes the main publications on the effects of several nutritional supplements for AMD. Although several trials have been done, they have generally been small and of short duration, resulting in inconclusive results.

IVd. Age-Related Eye Disease Study (AREDS)

AREDS1

Because of inconsistent evidence from observational studies and the public health concern regarding the widespread use of unproven, high-dose antioxidant and zinc supplements for AMD, the National Eye Institute (NEI) incorporated a clinical trial as part of the Age-Related Eye Disease Study. This 11-center double masked clinical trial is the largest study on the effects of nutritional supplements in AMD-patients and was published in 2001 (AREDS Research Group, 2001). The AREDS Research group aimed to evaluate the effect of high-dose vitamins C and E, β-carotene, and zinc supplement on AMD progression a visual acuity. The study randomized 3640 patients into four treatment groups, receiving daily tablets containing:

(1) Antioxidants (vitamin C, 500 mg, vitamin E, 400 IU and β-carotene, 15 mg)
(2) Zinc (80 mg zinc oxide) and copper (2mg cupric oxide, to prevent potential anaemia)
(3) Antioxidants plus zinc
(4) Placebo

In total, 4757 participants (aged 55-80 years) were enrolled in AREDS (Figure 17). Participants were enrolled if they had extensive small drusen, intermediate drusen, large drusen, non-central GA, or pigment abnormalities in 1 or both eyes, or advanced AMD or vision loss due to AMD in 1 eye. All participants had a best-corrected visual acuity of 20/32 or better in at least 1 eye (study eye). The participants were divided into 4 categories, determined by the size and extend of drusen and RPE abnormalities in each eye, the presence of advanced AMD and visual acuity (Appendix Figure 19):

Cat. 1 = Few or no drusen
Cat. 2 = Extensive small drusen, pigment abnormalities, or at least 1 intermediate size druse

Cat. 3 = Extensive intermediate drusen, GA not involving the centre of the macula, or at least 1 large druse
Cat. 4 = Advanced AMD or visual acuity less than 20/32 due to AMD in 1 eye

Because the effects of antioxidants in category 1 could not be assessed, the report focussed on the 3640 participants enrolled in the AMD clinical trial. There were no significant differences in demographics, socioeconomic status, smoking status or comorbidities noted between Category 2, 3, and 4 participants.

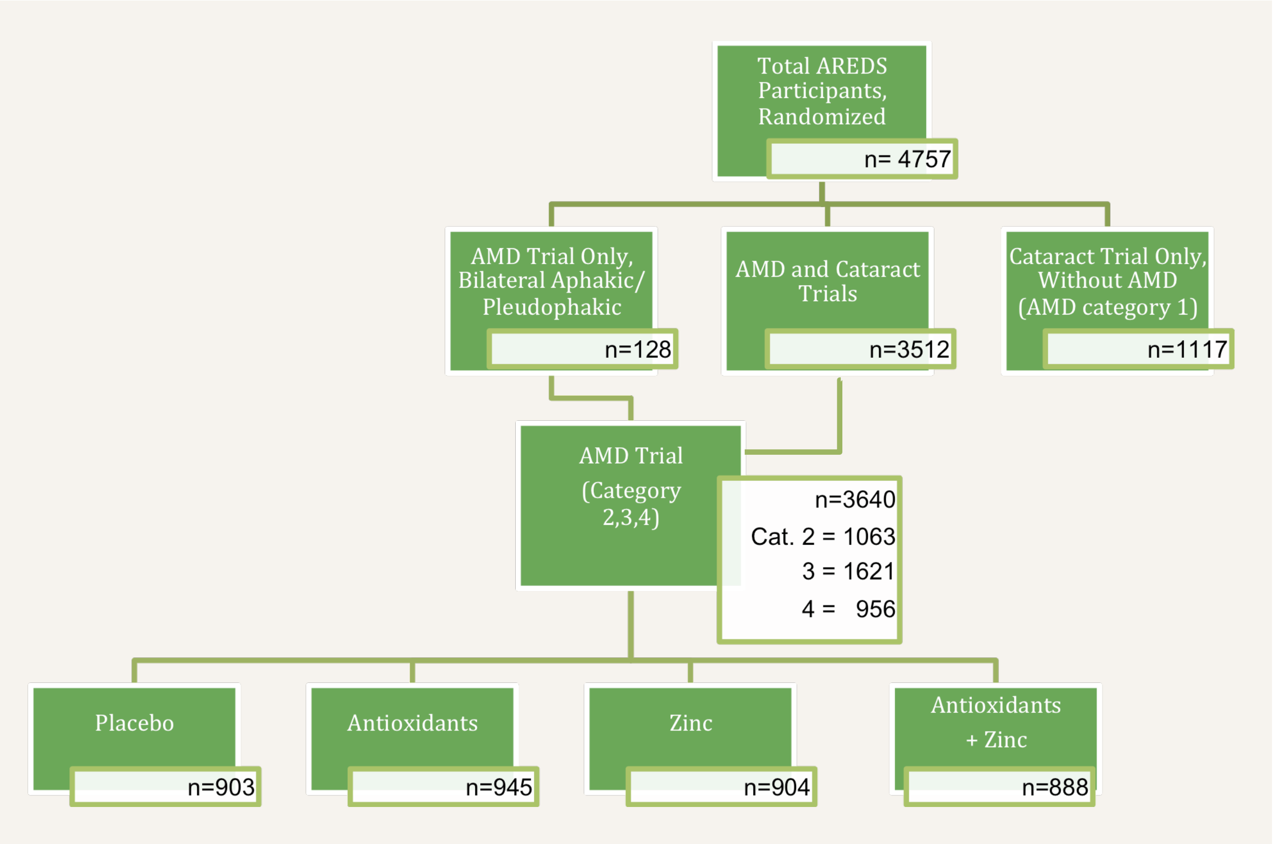

The primary outcome of this study was progression to advanced AMD (central GA or choroidal neovascularization) and at least moderate functional visual loss of ≥ 15 letters on an ETDRS logMAR chart. Serum levels of each of the vitamins or minerals and estimated pill counts suggested good adherence to the medication regimes. Because 75% of the participants wanted to take or continue to take their daily multivitamin during the AREDS screening examination, Centrum without lutein was provided. Hereby, these persons had an increase of approximately 100% of the RDA for each of the study ingredients, whether assigned to placebo or active intervention. Any increase in serum levels resulting form this intake was negligible compared with serum increases from the use of study supplements. The treatment effect of the study formulations was beneficial for both the group of participants supplementing with Centrum as for the group not choosing to use Centrum.

In Category 3, the probability of developing advanced AMD by 5 years among participants receiving placebo varied from about 6% to 27%. Participants in Category 4 had the highest probability of progression in the group receiving placebo, with an estimated probability of 43% at 5 years. Data from AREDS demonstrate that treatment with zinc alone or in combination with antioxidants reduced the risk of progression to advanced AMD in participants in Categories 3 and 4. Those taking antioxidants alone showed a risk reduction of 17%, those taking zinc alone showed a risk reduction of 21%. The risk reduction for those taking antioxidants plus zinc was 25% (probability of progression was 28% for placebo versus 20% for antioxidants plus zinc). These findings are partly supported by the effect on the visual acuity: only participants in Categories 3 and 4 assigned to antioxidants plus zinc had a statistically significant reduction (27%) in odds of a 15-letter of greater visual acuity decrease (OR, 0.73; 99% CI, 0.54–0.99.

There were too few advanced AMD events occurring in Category 2 participants to assess whether any treatments tested could slow the progression to advanced AMD. Participants in Category 2 only had a

1.3% 5-year probability of progression to advanced AMD. There was no statistically significant evidence of a benefit in delaying the progression of Category 2 eyes to more severe drusen pathology. The removal of this group provided more appropriate estimates of odds reduction within participants at risk for advanced AMD development.

No statistically significant serious adverse effect was associated with any of the formulations.

The AREDS study suggested that non-smokers with extensive intermediate size drusen, at least 1 large druse, noncentral GA in 1 or both eyes or advanced AMD or vision loss due to AMD in 1 eye should consider taking a supplement of antioxidants plus zinc similar to the supplement used in this study.

AREDS2

Several observational studies suggest that higher intake of lutein and zeaxanthin is associated with a decreased risk of progressing to advanced AMD. The carotenoids were considered for the original AREDS formula, but at the time neither carotenoid was available for manufacturing in a research formulation. Therefore β-carotene was in the formula. However, other studies using a similar dose of β-carotene in persons at high risk for lung cancer (smokers) have demonstrated an increased incidence of cancer and mortality in persons assigned to β-carotene supplementation (Omenn et al. 1996, ATBC Cancer Prevention Study Group 1994b).

As mentioned earlier, epidemiologic studies and observational data suggest that increased dietary intake of the ω3-LCPUFAs DHA and EPA is associated with a decreased risk of developing advanced AMD (SanGiovanni and Chew 2005). Furthermore, the high doses of zinc used in the AREDS study might not be necessary, because date suggests that the systemic absorption of zinc is limited to about 25 mg per day (Krishnadev et al. 2010). This information provides biological bases for the testing of these nutrients in a follow-up study. The National Institute of Health (NIH) commissioned the NEI to conduct the Age-Related Eye Disease Study 2 (AREDS2) (AREDS2 Research Group, 2012, Pinazo-Durán et al. 2104a).

AREDS2 is a multicentre phase-3 randomized controlled clinical trial conducted in 2006 to 2012. The aim of the study was to evaluate the efficacy and safety of lutein+zeaxanthin (L+Z) an/or omega-3 long chain polyunsaturated fatty acids (LCPUFA) supplementation in reducing the risk of developing advanced AMD. The study also evaluated the effect of lowering zinc component and/or eliminating β-carotene from the original AREDS formulation (AREDS2 Research Group, 2012).

All 4203 participants (aged 50-85) had bilateral intermediate AMD or advanced AMD in one eye. They were randomly divided into 4 groups (Figure 18):

(1) Placebo
(2) Lutein (10mg) + Zeaxanthin (2mg)
(3) ω3-LCPUFA
- 650mg EPA
- 350 mg DHA
(4) Lutein (10mg) + Zeaxanthin (2mg) + EPA (650 mg) + DHA (350 mg)

All participants were also asked to take the original AREDS formulation or accept a secondary randomization to 4 variations of the AREDS formulation, including elimination of β-carotene, lowering of zinc dose, or both (Appendix Figure 20, Appendix Figure 21).

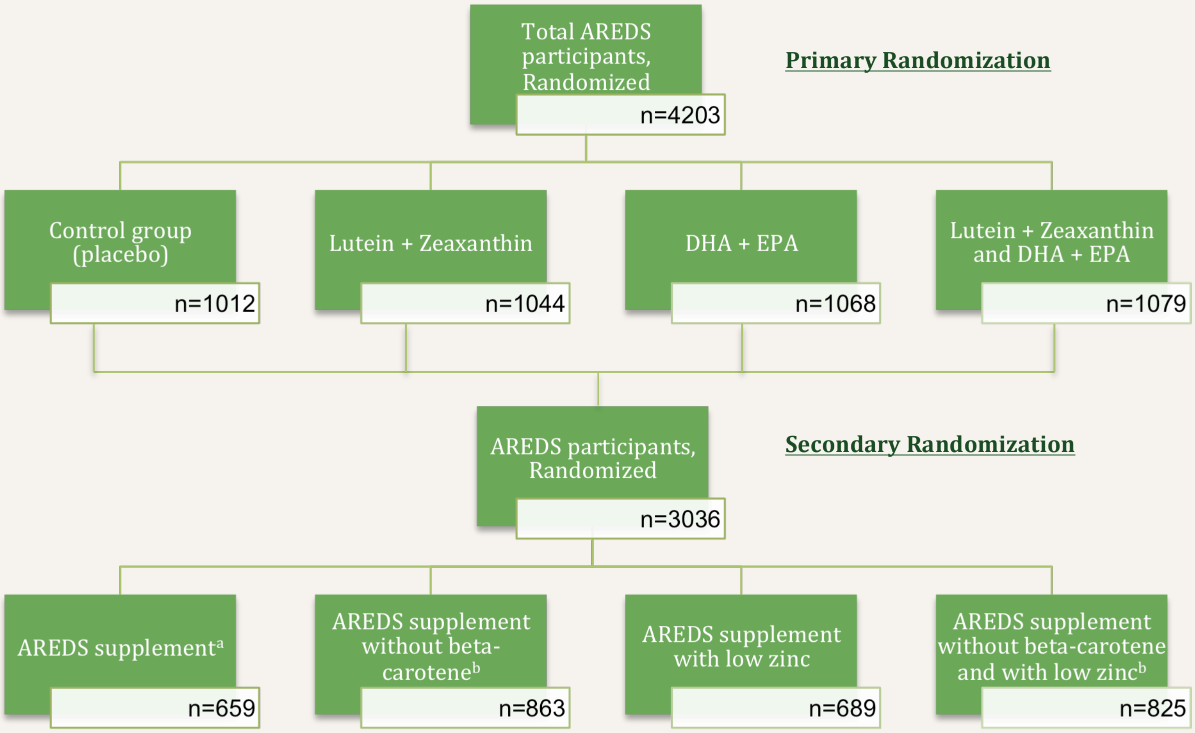

Figure 18 **Age-related Eye Disease Study 2 (AREDS2) study design**
DHA: docosahexanoic acid, EPA: eicosapentaneoic acid
[a] The original AREDS supplement: vitamin C (500 mg), vitamin E (400 IU), β-carotene (15 mg), zinc (80 mg zinc oxide) and copper (2 mg cupric oxide)
[b] β-carotene: smokers were randomly assigned to one of these two groups without β-carotene in the formulation

After a median follow-up of 5 years, 1940 study eyes (1608 participants) progressed to advanced AMD. Kaplan-Meier probabilities of progression to advanced AMD by 5 years were 31% for placebo, 29% for lutein+zeaxanthin, 31% for DHA+EPA and 30% for lutein+zeaxanthin and DHA+EPA. Comparison with placebo in the primary analyses demonstrated no statistically significant reduction in the progression to advanced AMD ((hazard ratio [HR], 0.90 [98.7% CI, 0.76- 1.07]; *P* = .12 for lutein+zeaxanthin; 0.97 [98.7% CI, 0.82-1.16]; *P* = .70 for DHA+EPA; 0.89 [98.7% CI, 0.75-1.06]; *P*= .10 for lutein+zeaxanthin and DHA+EPA). There was some benefit to the subgroup of patients who did not get β-carotene and to patients who were given lutein and zeaxanthin, but only when the patients' normal diets were deficient in these nutrients (Singer et al. 2014). There was no apparent effect of β-carotene elimination or lower-dose of zinc on progression to advanced MD. However, more lung cancers were noted in the β-carotene vs. no β-carotene group (23 [2.0%] vs. 11 [0.9%], nominal P=0.04), mostly in former smokers.

The AREDS2 trial results were not able to show overall improvement of the original AREDS formula by adding lutein, zeaxanthin and ω3-LCPUFA. However, because of a potential increased incidence of lung cancer in smokers and former smokers, lutein and zeaxanthin could be an appropriate carotenoid substitute in the AREDS formulation.

Table 2 Publication on the effects of antioxidants, ω3-LCPUFAs and zinc supplements for AMD

	Antioxidants	***Omega 3 LPUFA***	***Zinc***
Positive effects	AREDS Research Group 2001<br>AREDS2 Research Group 2013<br>Beatty et al. 2013<br>Moeller et al. 2006<br>Newsome et al 1988<br>Newsome 2008<br>Richer 1996<br>Richer et al. 2004<br>Seddon et al. 1994<br>Stur et al. 1996<br>Tan et al. 2008<br>West et al. 1994	Augood et al. 2008<br>Cho et al. 2001a<br>Chong et al. 2008<br>Christen et al. 2011<br>Chua et al. 2006<br>Feher et al. 2005<br>SanGiovanni et al. 2008<br>SanGiovanni et al. 2009a<br>SanGiovanni et al. 2009b<br>Seddon et al. 2001<br>Seddon et al. 2003<br>Seddon et al. 2006a<br>Swenor et al. 2010<br>Tan et al. 2009	van Leeuwen et al. 2005<br>Mares-Perlman et al. 1996<br>Newsome et al. 1988<br>Newsome 2008<br>Tan et al. 2008
Without effects	AREDS 2 Research Group 2013<br>Bartlett et al. 2007<br>Cho et al. 2004<br>Chong et al. 2007<br>Christen et al. 2014<br>Flood et al. 2002<br>van den Langenberg et al. 1998<br>van Leeuwen et al. 2005<br>Moeller et al. 2006<br>Omenn et al. 1996<br>Seddon et al. 1994<br>Smith et al. 1999<br>Taylor et al. 2002<br>Teikari et al. 1998<br>West et al. 1994	AREDS2 Research Group 2013<br>Hodge et al. 2006<br>Olson et al. 2011<br>Souied et al. 2013<br>The ATBC Cancer Prevention Study Group 1994a	Cho et al. 2001b<br>Flood et al. 2002<br>van den Langenberg 1998<br>Smith et al. 1999<br>Stur et al. 1996<br>Eye Disease Case-Control Study Group 1992
Negative effects	AREDS Research Group 2001<br>Goodman et al. 1993<br>Omenn et al. 1996		AREDS Research Group 2001

V. DISCUSSION

The AMD pathogenesis involves a complex interaction of cellular and molecular factors, which may be induced by light damage, oxidative stress, apoptosis, angiogenesis and inflammation. There is increasing evidence that modifiable risk factors, such as smoking and possibly diet, can alter genetic susceptibility for AMD (Sobrin and Seddon 2014). Since current therapies are not sufficient enough to treat all forms of AMD, AMD is a growing field of interest and many studies have investigated the importance of nutritional supplements in AMD progression. The effects of antioxidants, zinc and ω3-LCPUFA on AMD progression have been discussed (Table 2). Numerous small trials have investigated the role of dietary antioxidants, ω3-LCPUFA and zinc in AMD. Although several studies have reported beneficial results, there are also data demonstrating no effects or even negative effects of nutritional supplements. The AREDS study was one of the major clinical studies in this area and clinical recommendations have primarily been based on the data of this study.

Several factors should be considered when interpreting the AREDS-data. First, the population participating in the AREDS studies may differ from the general population, since the participants were relatively well-nourished, and the education level was high (32% and 45% of the participants had a bachelor's degree in AREDS1 and AREDS2 resp.). In both trials, 96% of the study population was white. Dietary habits, environmental factors, medical conditions, lifestyles, and genetic background are different in the European countries from the American population involved in the AREDS. A European-based study would be very helpful to determine the effect of nutritional supplements on AMD progression.

Also, the population studied in AREDS1 and AREDS2 differs in some aspects. In AREDS2, the rate of diabetes was higher (13%) compared to AREDS (6%). Also, 44% of the AREDS2 participants were taking statin-class cholesterol lowering drugs while only 9% of the AREDS2 participants took drugs of this kind.

Furthermore, AREDS was not capable to assess the treatment effect in all AMD patient-groups. The results of AREDS1 did not demonstrate beneficial effects of the AREDS formula in Categories 1 and 2 participants, while for Americans older than age 70 around 80% falls in these low-risk groups (Klein et al. 2007). Participants in these categories had low rates of progression to advanced AMD (≤1,3% in 5 years) and therefore the study had very low power to assess the effect of these treatments on the development of advanced AMD. The study was also not designed to address whether supplementation benefits persons who already have advanced neovascular AMD in both eyes.

Besides, the AREDS data suggest that 7 years of follow-up combination therapy in participants confers a treatment benefit, but it is not known how long someone at risk for advanced AMD should use the supplements to confer a beneficial effect.

The AREDS results seem very promising, but one should be careful with the interpretation of the given results. For example, the researchers suggested that removal of category 1 and 2 patients would provide more appropriate estimates of odds reduction within participants at risk for advanced AMD. In some cases, these categories are not taken into account when results are given. Also, the AREDS presents many beneficial results, but does not always mention whether they are significant or not.

A misconception about AREDS is that the study claims to provide evidence of a preventative effect of nutritional supplements in AMD. However, the AREDS study was never designed to determine whether supplements could prevent the onset of AMD, but aimed to evaluate the effect of nutritional supplements on the progression of AMD. To test the preventative effects of dietary patterns, observational (non-randomized) studies need to be done. The interpretation of observational studies can however be problematic since these study designs are more prone to bias and confounding.

The 2014 NOG-guidelines recommend ophthalmologists to advise a nutritional supplement consisting of vitamin C, E, zeaxanthin, zinc, cupper and lutein to AREDS category 3 or 4 patients or patients with severe AMD in one or two eyes, noncentral GA in one eye or severe AMD or vision loss because of AMD. The guideline also advises to discourage smoking patients to supplement with β-carotene. Some studies have shown beneficial effects of these nutrients indeed, but so far supplementation has only been effective in a small proportion of patients. Harmful secondary effects of not only β-carotene, but also high dose of zinc have been reported. Moreover, the AREDS formula and similar products are quite expensive and are not covered by most patients' health insurances. Taking these notes into consideration, the current guidelines can be questioned.

However, a healthy lifestyle is always recommended. A balanced diet consisting of enough ω3-LCPUFA and dark green leafy vegetables, no smoking, a healthy weight and enough exercise might be important key players in a healthy lifestyle and could possibly influence susceptibility of various diseases.

In conclusion, current recommendations are primarily based on the results of AREDS. Although other trials have been done, they have generally been small and of short duration, resulting in inconclusive results. Although some results have been very promising, there is still insufficient evidence in the literature to recommend routine nutritional supplementation for slowing down AMD progression. Further large scale and sample randomised controlled trials need to be done in this area to provide sufficient evidence for the use of nutritional supplements in AMD.

VI. LITERATURE

Age-Related Eye Disease Research Group (2001) A randomized, placebo-controlled, clinical trial of high-dose supplementation with vitamins C and E, beta carotene, and zinc for age- related macular degeneration and vision loss: AREDS report no. 8. Archives of Ophthalmology 119: 1417–1436

Age-Related Eye Disease Study Research Group (2005) Risk factors for the incidence of Advanced Age-Related Macular Degeneration in the Age-Related Eye Disease Study (AREDS) AREDS report no. 19. Ophthalmology 112(4): 533-539

Age-related Eye Disease Study 2 Research Group (2012) The Age-Related Eye disease study (AREDS2): study design and baseline characteristics (AREDS2 Report Number 1). Ophthalmology 119(11): 2282-2289

Age-Related Eye Disease Study 2 Research Group (2013) Lutein + zeaxanthin and omega-3 fatty acids for age-related macular degeneration: the Age-Related Eye Disease Study 2 (AREDS2) randomized clinical trial. JAMA 309: 2005–2015

Ambati J, Ambati BK, Yoo SH, Ianchulev S, Adamis AP (2003) Age-Related Macular Degeneration: Etiology, Pathogenesis, and Therapeutic Strategies. Survey of Ophthalmology 48 (3): 257-293

Anderson DH, Mullins RF, Hageman GS, Johnson LV (2002) A role for local inflammation in the formation of drusen in the aging eye. American Journal of Ophthalmology 134(3): 411-431

Augood C, Chakravarthy U, Young I, Vioque J, de Jong PT, Bentham G, Rahu M, Seland J, Soubrane G, Tomazzoli L, Topouzis F, Vingerling JR, Fletcher AE (2008) Oily fish consumption, dietary docosahexaenoic acid and eicosapentaenoic acid intakes, and associations with neovascular age-related macular degeneration. The American Journal of Clinical Nutrition 88: 398–406

Axer-Siegel R, Bourla D, Ehrlich R, Dotan G, Benjamini Y, Gavendo S, Weinberger D, Sela BA (2004) Association of neovascular age-related macular degeneration and hyperhomocysteinemia. American Journal of Ophthalmology 137(1): 84-89

Bakthavatchalu V, Dey S, Xu Y, Noel T, Jungsuwadee P, Holley AK, Dhar SK, Batinic-Haberle I, St Clair DK (2012) Manganese superoxide dismutase is a mitochondrial fidelity protein that protects Polγ against UV-induced inactivation. Oncogene 31(17): 2129-2139

Bartlett HE, Eperjesi F (2007) Effect of lutein and anti-oxidant dietary supplementation on contrast sensitivity in age-related macular disease: A randomized controlled trial. European Journal of Clinical Nutrition 61: 1121–1127

Beatty S, F, Koh HH, Phil M, Henson D, Boulton M (2000) The Role of Oxidative Stress in the Pathogenesis of Age-Related Macular Degeneration. Survey of Ophthalmology 45(2): 115-134

Beatty S, Chakravarthy U, Nolan JM, Muldrew KA, Woodside JV, Denny F, Stevenson MR (2013) Secondary outcomes in a clinical trial of carotenoids with coantioxidants versus placebo in early age-related macular degeneration. Ophthalmology 120(3): 600-606

Bhutto IA, McLeod SD, Hasegawa T, Kim SY, Merges C, Tong P, Lutty G (2006) Pigment epithelium-derived factor (PEDF) and vascular endothelial growth factor (VEGF) in aged human choroid and eyes with age-related macular degeneration. Experimental Eye Research 82: 99–110

Blasiak J, Glowacki S, Kauppinen A, Kaarniranta K (2013) Mitochondrial and nuclear DNA damage and repair in age-related macular degeneration. International Journal of Molecular 14(2): 2996–3010

Blasiak J, Petrovski G, Veréb Z, Facskó A, Kaarniranta K (2014) Oxidative stress, hypoxia, and autophagy in the neovascular processes of age-related macular degeneration. BioMed Research International 2014: 768026

Block RC, Dier U, Calderonartero P, Shearer GC, Kakinami L, Larson MK, Harris WS, Georas S, Mousa SA (2012) The Effects of EPA+DHA and Aspirin on Inflammatory Cytokines and Angiogenesis Factors. World Journal of Cardiovascular Diseases 2(1): 14-19

Buentello-Volante B, Rodriguez-Ruiz G, Miranda-Duarte A, Pompa-Mera EN, Graue-Wiechers F, Bekker-Méndez C, Ayala-Ramirez R, Quezada C, Rodríguez-Loaiza JL, Zenteno JC (2012) Susceptibility to advanced age-related macular degeneration and alleles of complement factor H, complement factor B, complement component 2, complement component 3, and age-related maculopathy susceptibility 2 genes in a Mexican population. Molecular Vision 18: 2518-2525

Caire J, Recalde S, Velazquez-Villoria A, Garcia-Garcia L, Reiter N, Anter J, Fernandez-Robredo P, Alfredo García-Layana; Spanish Multicenter Group on AMD (2014) Growth of geographic atrophy on fundus autofluorescence and polymorphisms of CFH, CFB, C3, FHR1-3, and ARMS2 in age-related macular degeneration. JAMA Ophthalmology 132(5): 528-534

Campisi J and d'Adda di Fagagna F (2007) Cellular senescence: when bad things happen to good cells. Nature Reviews Molecular Cell Biology 8(9): 729-740

Chhablani J and Sudhalkar D (2014) Fluorescein angiography and optical coherence tomography correlation in various retinal diseases. Retina Today jan/feb: 77-80

Cho E, Hung S, Willett WC, Spiegelman D, Rimm EB, Seddon JM, Colditz GA, Hankinson SE (2001a) Prospective study of dietary fat and the risk of age-related macular degeneration. The American Journal of Clinical Nutrition 73(2): 209–218

Cho E, Stampfer MJ, Seddon JM, Hung S, Spiegelman D, Rimm EB, Willett WC, Hankinson SE (2001b) Prospective study of zinc intake and the risk of age-related macular degeneration. Annals of Epidemiology 11:3 28–336

Cho E, Seddon JM, Rosner B, Willett WC, Hankinson SE (2004) Prospective study of intake of fruits, vegetables, vitamins, and carotenoids and risk of age-related maculopathy. Archives of Ophthalmology 122(6): 883–892

Chong EW, Wong TY, Kreis AJ, Simpson JA, Guymer RH (2007) Dietary antioxidants and primary prevention of age related macular degeneration: systematic review and meta-analysis. British Medical Journal 335(7623): 755-759

Chong EW, Kreis AJ, Wong TY, Simpson JA, Guymer RH (2008) Dietary ω-3 fatty acid and fish intake in the primary prevention of age-related macular degeneration: a systematic review and meta-analysis. Archives of Ophthalmology 126(6): 826–833

Christen WG, Schaumberg DA, Glynn RJ, Buring JE (2011) Dietary ω-3 fatty acid and fish intake and incident age-related macular degeneration in women. Archives of Ophthalmology 129(7): 921–929

Christen WG, Glynn RJ, Manson JE, Macfadyen J, Bubes V, Schvartz M, Buring JE, Sesso HD, Gaziano JM (2014) Effects of multivitamin supplement on cataract and age-related macular degeneration in a randomized trial of male physicians. Ophthalmology 121: 525–534

Chua B, Flood V, Rochtchina E, Wang JJ, Smith W, Mitchell P (2006) Dietary fatty acids and the 5-year incidence of age-related maculopathy. Archives of Ophthalmology 124(7): 981–986

Chopdar A and Aung T (2014) Multimodal Retinal Imaging. London: JP Medical Publishers

Coleman HR, Chan CC, Ferris FL, Chew EY (2008) Age-related macular degeneration. Lancet 2008; 372: 1835–1845

Cui H, Kong Y, Zhang H (2012) Oxidative Stress, Mitochondrial Dysfunction, and Aging. Journal of Signal Transduction: 646354

Damico FM, Gasparin F, Ramos Scolari M, Sampaio Pedral L, Takahashi BS (2012) New approaches and potential treatments for dry age-related macular degeneration. Arquivos Brasileiros de Oftalmologia 75(1): 71-75

DeWan A, Liu M, Hartman S, Zhang SS, Liu DT, Zhao C, Tam PO, Chan WM, Lam DS, Snyder M, Barnstable C, Pang CP, Hoh J (2006) HTRA1 promoter polymorphism in wet age-related macular degeneration. Science 314: 989–992

Donoso LA, Kim D, Frost A, Callahan A, Hageman G (2006) The Role of Inflammation in the Pathogenesis of Age-related Macular Degeneration. Survey of Ophthalmology 51(2): 137-152

Du H, Sun X, Guma M, Luo J, Ouyang H, Zhang X, Zeng J, Quach J, Nguyen DH, Shaw PX, Karin M, Zhang K (2013) JNK inhibition reduces apoptosis and neovascularization in a murine model of age-related macular degeneration. PNAS 110(6): 2377-2392

Dunaief JL, Dentchev T, Ying GS, Milam AH (2002) The role of apoptosis in age-related macular degeneration. Archives of Ophthalmology 120: 1435-1442

Evans JR and Lawrenson JG (2012) Antioxidant vitamin and mineral supplements for preventing age-related macular degeneration. Cochrane Database of Systematic Reviews 6: CD000253

Eye Disease Case-Control Study Group (1992) Risk factors for neovascular age-related macular degeneration. Archives of Ophthalmology 110(12): 1701-1708

Feher J, Kovacs B, Kovacs I, Schvoller M, Papale A, Gabrieli CB (2005) Improvement of visual functions and fundus alterations in early age-related macular degeneration treated with a combination of acetyl-L-carnitine, ω-3 fatty acids, and coenzyme Q10. Ophthalmologica 219(3): 154–166

Ferris FL, Wilkinson CP, Bird A, Chakravarthy U, Chew E, Csaky K, Sadda SR (2013) Clinical Classification of Age-related Macular Degeneration. Ophthalmology 120(4): 844–851

Flood V, Smith W, Wang JJ, Manzi F, Webb K, Mitchell P (2002) Dietary antioxidant intake and incidence of early age-related maculopathy: The Blue Mountains Eye Study. Ophthalmology 109; 2272–2278

Fritsche LG, Loenhardt T, Janssen A, Fisher SA, Rivera A, Keilhauer CN, Weber BH (2008) Age-related macular degeneration is associated with an unstable ARMS2 (LOC387715) mRNA. Nature Genetics 40(7): 892-896

Fujimoto JG, Pitris C, Boppart SA, Brezinskiz ME (2000) Optical Coherence Tomography: An Emerging Technology for Biomedical Imaging and Optical Biopsy. Neoplasia 2: 9-25

Gale CR, Hall NF, Phillips DI, Martyn CN (2003) Lutein and zeaxanthin status and risk of age-related macular degeneration. Investigative Ophthalmology & Visual Science 44(6): 2461-2465

Gao G, Li Y, Zhang D, Gee S, Crosson C, Ma J (2001) Unbalanced expression of VEGF and PEDF in ischemia-induced retinal neovascularization. FEBS Letters 489(2-3): 270-276

Goodman GE, Omenn GS, Thornquist MD, Lund B, Metch B, Gylys-Colwell I (1993) The Carotene and Retinol Efficacy Trial (CARET) to prevent lung cancer in high-risk populations: pilot study with cigarette smokers, Cancer Epidemiology Biomarkers and Prevention 2(4): 389–396

Gourbeyre P, Denery S, Bodinier M (2011) Probiotics, prebiotics, and synbiotics: impact on the gut immune system and allergic reactions. Journal of Leukocyte Biology 89: 685 - 695

Grahn BH, Paterson PG, Gottschall-Pass KT, Zhang Z (2001) Zinc and the eye. The Journal of the American College of Nutrition 20(suppl): 106–118

Grupo de Estudos da Retina (GER), revised by Bandello F (2010) Age-related Macular Degeneration Book. Loures: Théa Portugal

Hageman GS and Mullins RF (1999) Molecular composition of drusen as related to substructural phenotype. Molecular Vision 5:28

Hageman GS, Luthert PJ, Victor Chong NH, Johnson LV, Anderson DH, Mullins RF (2001) An integrated hypothesis that considers drusen as biomarkers of immune-mediated processes at the RPE-Bruch's membrane interface in aging and age-related macular degeneration. Progress in Retinal and Eye Research 20(6): 705-732

Hanout M, Ferraz D, Ansari M, Maqsood N, Kherani S, Sepah YJ, Rajagopalan N, Ibrahim M, Do DV, Nguyen QD (2013) Therapies for Neovascular Age-Related Macular Degeneration: Current Approaches and Pharmacologic Agents in Development. BioMed Research International: 830837

Hodge WG, Schachter HM, Barnes D, Pan Y, Lowcock EC, Zhang L, Sampson M, Morrison A, Tran K, Miguelez M, Lewin G (2006) Efficacy of ω-3 fatty acids in preventing age-related macular degeneration: a systematic review. Ophthalmology 113(7): 1165–1173

Jampol LM, Tielsch J (1992) Race, macular degeneration and the Macular Photocoagulation Study. Archives of Ophthalmology 110: 1699–700

Johnson PT, Betts KE, Radeke MJ, Hageman GS, Anderson DH, Johnson LV (2006) Individuals homozygous for the age-related macular degeneration risk-conferring variant of complement factor H have elevated levels of CRP in the choroid. PNAS 103(46): 17456–17461

de Jong PT (2006) Age-related macular degeneration. The New English Journal of Medicine 355: 1474–1485

Justilien V, Pang JJ, Renganathan K, Zhan X, Crabb JW, Kim SR, Sparrow JR, Hauswirth WW, Lewin AS (2007) SOD2 knockdown mouse model of early AMD. Investigative Ophthalmology & Visual Science 48: 4407–4420

Kamata H, Honda S, Maeda S, Chang L, Hirata H, Karin M (2005) Reactive oxygen species promote TNFalpha-induced death and sustained JNK activation by inhibiting MAP kinase phosphatases. Cell 120(5): 649–661

Kamburoglu G, Gumus K, Kadayifcilar S, Eldem B (2006) Plasma homocysteine, vitamin B12 and folate levels in age-related macular degeneration. Graefe's Archive of Clinical and Experimental Ophthalmology 244(5): 565-569

Katta S, Kaur I, Chakrabarti S (2009) The molecular genetic basis of age-related macular degeneration: an overview. Journal of Genetics 88: 425–449

Kim SY, Sadda S, Pearlman J, Humayun MS, de Juan E Jr, Melia BM, Green WR (2002) Morphometric analysis of the macula in eyes with disciform age-related macular degeneration. Retina 22: 471–477

Klaver CC, Wolfs RC, Assink JJ, van Duijn CM, Hofman A, de Jong PT (1998) Genetic risk of age-related maculopathy. Population-based familial aggregation study. Archives of Ophthalmology 116: 1646-1651

Klein R, Klein BE, Knudtson MD, Meuer SM, Swift M, Gangnon RE (2007) Fifteen-year cumulative incidence of age-related macular degeneration: the Beaver Dam Eye Study. Ophthalmology 114: 253–262

Klein R, Cruickshanks KJ, Nash SD, Krantz EM, Nieto FJ, Huang GH, Pankow JS, Klein BE (2010) The Prevalence of Age-Related Macular Degeneration and Associated Risk Factors. Archives of Ophthalmology 128(6): 750-758

Krishnadev N, Meleth AD, Chew EY (2010) Nutritional supplements for age-related macular degeneration. Current Opinion in Ophthalmology 21(3): 184–189

van den Langenberg GM, Mares-Perlman JA, Klein R, Klein BE, Brady WE, Palta M (1998) Associations between antioxidant and zinc intake and the 5-year incidence of early age-related maculopathy in the Beaver Dam Eye Study. American Journal of Epidemiology 148(2): 204–214

van Leeuwen R, Boekhoorn S, Vingerling JR, Witteman JC, Klaver CC, Hofman A, de Jong PT (2005) Dietary intake of antioxidants and risk of age-related macular degeneration. JAMA 294(24): 3101–3107

Mares-Perlman JA, Klein R, Klein BE, Greger JL, Brady WE, Palta M, Ritter LL (1996) Association of zinc and antioxidant nutrients with age-related maculopathy. Archives of Ophthalmology 114: 991–997

Martin DF, Maguire MG, Ying GS, Grunwald JE, Fine SL, Jaffe GJ (The CATT Research Group) (2011) Ranibizumab and bevacizumab for neovascular age-related macular degeneration. New England Journal of Medicine 364: 1897–1908

Martin DF, Maguire MG, Fine SL, Ying GS, Jaffe GJ, Grunwald JE, Toth C, Redford M, Ferris FL III (The CATT Research Group) (2012) Ranibizumab and bevacizumab for treatment of neovascular age-related macular degeneration: two-year results - Comparison of Age-related Macular Degeneration Treatments Trials (CATT) Research Group. Ophthalmology 119(7): 1388-1398

Matés JM (2000) Effects of antioxidant enzymes in the molecular control of reactive oxygen species toxicology. Toxicology 153: 83–104

Michael Singer (2014) Advances in the management of macular degeneration. F1000Prime Reports 6:29

Mitchell P, Wang JJ, Foran S, Smith W. (2002) Five-year incidence of age-related maculopathy lesions: the Blue Mountains Eye Study. Ophthalmology 109: 1092–1097

Moeller SM, Parekh N, Tinker L, Ritenbaugh C, Blodi B, Wallace RB, Mares JA; CAREDS Research Study Group (2006) Associations between intermediate age-related macular degeneration and lutein and zeaxanthin in the Carotenoids in Age-Related Eye Disease Study (CAREDS): ancillary study of the Women's Health Initiative. Archives of Ophthalmology 124(8): 1151–1162

Mukherjee PK, Marcheselli VL, de Rivero Vaccari JC, Gordon WC, Jackson FE, Bazan NG (2007) Photoreceptor outer segment phagocytosis attenuates oxidative stress-induced apoptosis with concomitant neuroprotectin D1 synthesis. Proceedings of the National Academy of Sciences of the United States of America 104(32): 13158–13163

Nakao S, Arima M, Ishikawa K, Kohno R, Kawahara S, Miyazaki M, Yoshida S, Enaida H, Hafezi-Moghadam A, Kono T, Ishibashi T (2012) Intravitreal Anti-VEGF Therapy Blocks Inflammatory Cell Infiltration and Re-Entry into the Circulation in Retinal Angiogenesis. Investigative Ophthalmology & Visual Science 53(7): 4323-4328

Nederlands Oogheelkundig Gezelschap (NOG) (2014) Richtlijn Leeftijdsgebonden Maculadegeneratie

Negoescu A, Lorimier P, Labat-Moleur F, Drouet C, Robert C, Guillermet C, Brambilla C, Brambilla E (1996) In situ apoptotic cell labeling by the TUNEL method: improvement and evaluation on cell preparations. Journal of Histochemistry & Cytochemistry 44(9): 959-968

Newsome DA, Swartz M, Leone NC, Elston RC, Miller E (1988) Oral zinc in macular degeneration. Archives of Ophthalmology 106(2): 192-198

Ng EWM, Adamis AP (2005) Targeting angiogenesis, the underlying disorder in neovascular age-related macular degeneration. Canadian Journal of Ophthalmology 40: 352– 368

Nowak JZ (2006) Age-related macular degeneration (AMD): pathogenesis and therapy. Pharmacological Reports 58: 353-363

Olson JH, Erie JC, Bakri SJ (2011) Nutritional supplementation and age-related macular degeneration. Seminars in Ophthalmology 26(3): 131–136

Omenn GS, Goodman GE, Thornquist MD, Balmes J, Cullen MR, Glass A, Keogh JP, Meyskens FL, Valanis B, Williams JH, Barnhart S, Hammar S (1996) Effects of a combination of beta-carotene and vitamin A on lung cancer and cardiovascular disease. New England Journal of Medicine 334: 1150–1155

Pinazo -Durán MD, Gómez-Ulla F, Arias L, Araiz J, Casaroli-Marano R, Gallego-Pinazo R, García-Medina JJ, López-Gálvez MI, Manzanas L, Salas A, Zapata M, Diaz-Llopis M, García-Layana A (2014a) Do Nutritional Supplements Have a Role in Age Macular Degeneration Prevention? Journal of Opthalmology 2014: 901686

Pinazo-Durán MD, Gallego-Pinazo R, García-Medina JJ, Zanón-Moreno V, Nucci C, Dolz-Marco R, Martínez-Castillo S, Galbis-Estrada C, Marco-Ramírez C, López-Gálvez MI, Galarreta DJ, Díaz-Llópis M (2014b) Oxidative stress and its downstream signaling in aging eyes. Clinical interventions in Aging 11(9): 637-52

Pinhas A, Dubow M, Shah N, Chui TY, Scoles D, Sulai YN, Weitz R, Walsh JB, Carroll J, Dubra A, Rosen RB (2013) In vivo imaging of human retinal microvasculature using adaptive optics scanning light ophthalmoscope fluorescein angiography. Biomedical Optics Express 4(8): 1305-1317

Popp M, Chu XK, Shen D, Tuo J, Chan CC (2013) Evaluating Potential Therapies in a Mouse Model of Focal Retinal Degeneration with Age-related Macular Degeneration (AMD)-Like Lesions. Journal of Clinical & Experimental Ophthalmology 4(5): 1000296

Ramkumar HL, Tuo J, Shen de F, Zhang J, Cao X, Chew EY, Chan CC (2013) Nutrient supplementation with n3 polyunsaturated fatty acids, lutein, and zeaxanthin decrease A2E accumulation and VEGF expression in the retinas of Ccl2/Cx3cr1-deficient mice on Crb1rd8 background. Journal of Nutrition 143(7): 1129-1135

Richer S (1996) Multicenter ophthalmic and nutritional age-related macular degeneration study—part 2: antioxidant intervention and conclusions. Journal of the American Optometric Association 67(1): 30–49

Richer S, Stiles W, Statkute L, Pulido J, Frankowski J, Rudy D, Pei K, Tsipursky M, Nyland J (2004) Double-masked, placebo-controlled, randomized trial of lutein and antioxidant supplementation in the intervention of atrophic age-related macular degeneration: The Veterans LAST study (Lutein Antioxidant Supplementation Trial). Optometry 75: 216–230

Rickman CW, Farsiu S, Toth CA, Klingeborn M (2013) Dry Age-Related Macular Degeneration: Mechanisms, Therapeutic Targets, and Imaging. Investigative Ophthalmology & Visual Science 54: ORSF68–ORSF80

Salganik RI (2001) The Benefits and Hazards of Antioxidants: Controlling Apoptosis and Other Protective Mechanisms in Cancer Patients and the Human Population. Journal of the American College of Nutrition 20(5): 464–472

SanGiovanni JP, Chew EY (2005) The role of omega-3 long-chain polyunsaturated fatty acids in health and disease of the retina. Progress in Retinal and Eye Research 24: 87–138

SanGiovanni JP, Chew EY, Agrón E, Clemons TE, Ferris FL III, Gensler G, Lindblad AS, Milton RC, Seddon JM, Klein R, Sperduto RD; Age-Related Eye Disease Study Research Group (2008) The relationship of dietary omega-3 long-chain polyunsaturated fatty acid intake with incident age- related macular degeneration: AREDS report no. 23. Archives of Ophthalmology 126: 1274–1279

SanGiovanni JP, Agrón E, Clemons TE, Chew EY (2009a) Omega-3 long chain polyunsaturated fatty acid intake inversely associated with 12-year progression to advanced age-related macular degeneration [letter]. Archives of Ophthalmology 127: 110–112

SanGiovanni JP, Agrón E, Meleth AD, Reed GF, Sperduto RD, Clemons TE, Chew EY; Age-Related Eye Disease Study Research Group (2009b) Omega-3 long-chain polyunsaturated fatty acid intake and 12-y incidence of neovascular age-related macular degeneration and central geographic atrophy: AREDS report 30, a prospective cohort study from the Age-Related Eye Disease Study. The American Journal of Clinical Nutrition 90: 1601–1607

Seddon JM, Ajani UA, Sperduto RD, Hiller R, Blair N, Burton TC, Farber MD, Gragoudas ES, Haller J, Miller DT, Yannuzzi LA, Willett W; Eye Disease Case-Control Study Group (1994) Dietary carotenoids, vitamins A, C, and E and advanced age-related macular degeneration. JAMA 272: 1413–1420

Seddon JM, Rosner B, Sperduto RD, Yannuzzi L, Haller JA, Blair NP, Willett W (2001) Dietary fat and risk for advanced age-related macular degeneration. Archives of Ophthalmology 119: 1191–1199

Seddon JM, Cote J, Rosner B (2003) Progression of age-related macular degeneration: association with dietary fat, transunsaturated fat, nuts, and fish intake. Archives of Ophthalmology 121: 1728–1737

Seddon JM, Cote J, Page WF, Aggen SH, Neale MC (2005) The US twin study of age-related macular degeneration: relative roles of genetic and environmental influences. Archives of Ophthalmology 123: 321-327

Seddon JM, George S, Rosner B, Klein ML (2006a) CFH gene variant, Y402H, and smoking, body mass index, environmental associations with advanced age-related macular degeneration. Human Heredity 61(3): 157-165

Seddon JM, Gensler G, Klein ML, Milton RC (2006b) Evaluation of plasma homocysteine and risk of age-related macular degeneration. American Journal of Ophthalmology 141(1): 201-203

Seddon JM, Francis PJ, George S, Schultz DW, Rosner B, Klein MI (2007) Association of CFH Y402H and LOC387715 A69S with progression of age-related macular degeneration. JAMA 297: 1793–800

Sies H, Stahl W (1995) Vitamins E and C, beta-carotene, and other carotenoids as antioxidants. American Journal of Clinical Nutrition 62(6): 1315S-1321S

Sies H, Stahl W, Sundquist AR (1992) Antioxidant functions of vitamins. Vitamins E and C, beta-carotene, and other carotenoids. Annals of the New York Academy of Sciences 669: 7-20

Smith W, Assink J, Klein R, Mitchell P, Klaver CC, Klein BE, Hofman A, Jensen S, Wang JJ, de Jong PT (2001) Risk factors for age-related macular degeneration: pooled findings from three continents. Ophthalmology 108: 697–704

Smith W, Mitchell P, Webb K, Leeder SR (1999) Dietary antioxidants and age-related maculopathy: The Blue Mountains Eye Study. Ophthalmology 106: 761–767

Sobrin L, Maller JB, Neale BM, Reynolds RC, Fagerness JA, Daly MJ, Seddon JM (2010) Genetic profile for five common variants associated with age-related macular degeneration in densely affected families: a novel analytic approach. European Journal of Human Genetics 18: 496-501

Sobrin L, Seddon JM (2014) Nature and nurture- genes and environment- predict onset and progression of macular degeneration. Progress in Retinal and Eye Research 40: 1-15

Souied EH, Delcourt C, Querques G, Bassols A, Merle B, Zourdani A, Smith T, Benlian P; Nutritional AMD Treatment 2 Study Group (2013) Nutritional AMD treatment 2 study group. Oral docosahexaenoic acid in the prevention of exudative age-related macular degeneration: the nutritional AMD treatment 2 study. Ophthalmology 120(8):1619–1631

Sparrow JR, Nakanishi K, Parish CA (2000) The lipofuscin- fluorophore A2E mediates blue light-induced damage to retinal pigmented epithelial cells. Investigative Ophthalmology & Visual Science 41: 1981–1989

Sparrow JR, Zhou J, Ben-Shabat S, Vollmer H, Itagaki Y, Nakanishi K (2002) Involvement of oxidative mechanisms in blue-light-induced damage to A2E-laden RPE. Investigative Ophthalmology & Visual Science 43: 1222–1227

Spencer KL, Olson LM, Anderson BM, Schnetz-Boutaud N, Scott WK, Gallins P, Agarwal A, Postel EA, Pericak-Vance MA, Haines JL (2008) C3 R102G polymorphism increases risk of age-related macular degeneration. Human Molecular Genetics 17 (12): 1821-1824

Steinbrook R (2006) The Price of Sight — Ranibizumab, Bevacizumab, and the Treatment of Macular Degeneration . New England Journal of Medicine 355; 1409-1421

Stur M, Tittl M, Reitner A, Meisinger V (1996) Oral zinc and the second eye in age-related macular degeneration. Investigative Ophthalmology & Visual Science 37: 1225–1235

Swenor BK, Bressler S, Caulfield L, West SK (2010) The impact of fish and shellfish consumption on age-related macular degeneration. Ophthalmology 117: 2395–2401

Tan JS, Wang JJ, Flood V, Rochtchina E, Smith W, Mitchell P (2008) Dietary antioxidants and the long-term incidence of age-related macular degeneration: The Blue Mountains Eye study. Ophthalmology 115; 334–341

Tan JS, Wang JJ, Flood V, Mitchell P (2009) Dietary fatty acids and the 10-year incidence of age-related macular degeneration: the Blue Mountains Eye Study. Archives of Ophthalmology 127: 656–665

Tanemura M, Miyamoto N, Mandai M, Honda Y (2001) Estrogen may enhance VEGFR-2 expression in the process of laser- induced CNV formation. Investigative Ophthalmology & Visual Science 42: S96

Taylor HR, Tikellis G, Robman LD, McCarty CA, McNeil JJ (2002) Vitamin E supplementation and macular degeneration: randomised controlled trial. British Medical Journal 325(7354): 11–14

Teikari JM, Laatikainen L, Virtamo J, Haukka J, Rautalahti M, Liesto K, Albanes D, Taylor P, Heinonen OP (1998) Six-year supple-mentation with alpha-tocopherol and beta-carotene and age- related maculopathy, Acta Ophthalmologica Scandinavica 76 (2): 224–229

The ATBC Cancer Prevention Study Group (1994a) The alpha-tocopherol, beta-carotene lung cancer prevention study: design, methods, participant characteristics, and compliance. Annals of Epidemiology 4(1): 1–10

The ATBC Cancer Prevention Study Group (1994b) The effect of vitamin E and beta carotene on the incidence of lung cancer and other cancers in male smokers. New England Journal of Medicine 330: 1029–1035

Tokarz P, Kaarniranta K, Blasiak J (2013) Role of antioxidant enzymes and small molecular weight antioxidants in the pathogenesis of age-related macular degeneration (AMD). Biogerontology 14: 461–482

Tombran-Tink J and Barnstable CJ (2006) Ocular Angiogenesis: Diseases, Mechanisms, and Therapeutics. Towota: Humana Press

Tuo J, Grob S, Zhang K, Chan CC (2012) Genetics of immunological and inflammatory components in age-related macular degeneration. Ocular Immunology and Inflammation 20(1): 27-36

Ungvari Z, Orosz Z, Rivera A, Labinskyy N, Xiangmin Z, Olson S, Podlutsky A, Csiszar A (2007) Resveratrol increases vascular oxidative stress resistance. American Journal of Physiology - Heart and Circulatory Physiology 292(5): 2417-2424

Vingerling JR, Dielemans I, Hofman A, Grobbee DE, Hijmering M, Kramer CF, de Jong PT (1995) The prevalence of age-related maculopathy in the Rotterdam Study. Ophthalmology 102: 205–210

Vingerling JR, Hofman, A, Grobbee DE, de Jong PT (1996) Age-Related Macular Degeneration and Smoking - The Rotterdam Study. Archives of Ophthalmology 114(10): 1193-1196

Waisbourd M, Loewenstein A, Goldstein M, Leibovitch I (2007) Targeting Vascular Endothelial Growth Factor - A Promising Strategy for Treating Age-Related Macular Degeneration. Drugs Aging 24(8): 643-662

West S, Vitale S, Hallfrisch J, Muñoz B, Muller D, Bressler S, Bressler NM (1994) Are antioxidants or supplements protective for age-related macular degeneration? Archives of Ophthalmology 112: 222–227

Wu J, Seregard S, Algvere PV (2006) Photochemical damage of the retina. Survey of Ophthalmology 51(5): 461–481

Zhang SX, Wang JJ, Gao G, Parke K, Ma J (2006) Pigment epithelium-derived factor downregulates vascular endothelial growth factor (VEGF) expression and inhibits VEGF–VEGF receptor 2 binding in diabetic retinopathy. Journal of Molecular Endocrinology 37: 1–12

VII. APPENDIX

AMD Eligibility Categories

AMD Category	First Eye[*]			Second Eye
	Drusen Size[†]	Drusen Area[†]	Pigment Abnormalities[‡]	
1	None or small (<63 µm)	<125 µm diameter circle (≈5–15 drusen) small	None	Same as first eye
2	Small (<63 µm)	≥125 µm diameter circle (about 1/150 disc area)	Absent or present, but GA absent	Same as first eye or Category 1
	Or intermediate (≥63, <125 µm)	At least 1 druse		
	Or none required if pigment abnormalities present			
3a	Intermediate (≥63, <125 µm)	≥360 µm diameter circle (about 1/16 disc area) if soft indistinct drusen are present (≈20 intermediate drusen)	Absent or present, but central GA[†] absent	Same as first eye or Category 1 or 2
		≥656 µm diameter circle (about 1/5 disc area), if soft indistinct drusen are absent (≈65 intermediate drusen)		
	Or large (≥125 µm)	At least 1 druse		
	Or none required, if noncentral GA[†] is present			
3b	First eye same as Category 3a			VA <20/32 not due to AMD[§], or uniocular disqualifying disorder is present[‖]
4a	First eye same as Category 1, 2, or 3a			Advanced AMD[¶]
4b	First eye same as Category 1, 2, or 3a			VA <20/32 due to AMD, but advanced AMD[¶] not present[§]

[*] Must have visual acuity (VA) ≥20/32, no advanced age-related macular degeneration (AMD), and no disqualifying lesions.

[†] Drusen and geographic atrophy (GA) are assessed within 2 disc diameters (3000 µm)[33] of the center of the macula.

[‡] Pigment abnormalities (increased pigmentation or depigmentation) within 1 disc diameter of the center of the macula.

[§] Eye not eligible for VA event.

[‖] Eye not eligible for AMD event.

[¶] The GA involving center of macula or signs of choroidal neovascularization (presence beneath the retinal pigment epithelium or sensory retina of fluid, blood, or fibrovascular or fibrous tissue).

Figure 19 AMD Eligibility Categories in AREDS1 (AREDS Research Group 2001)

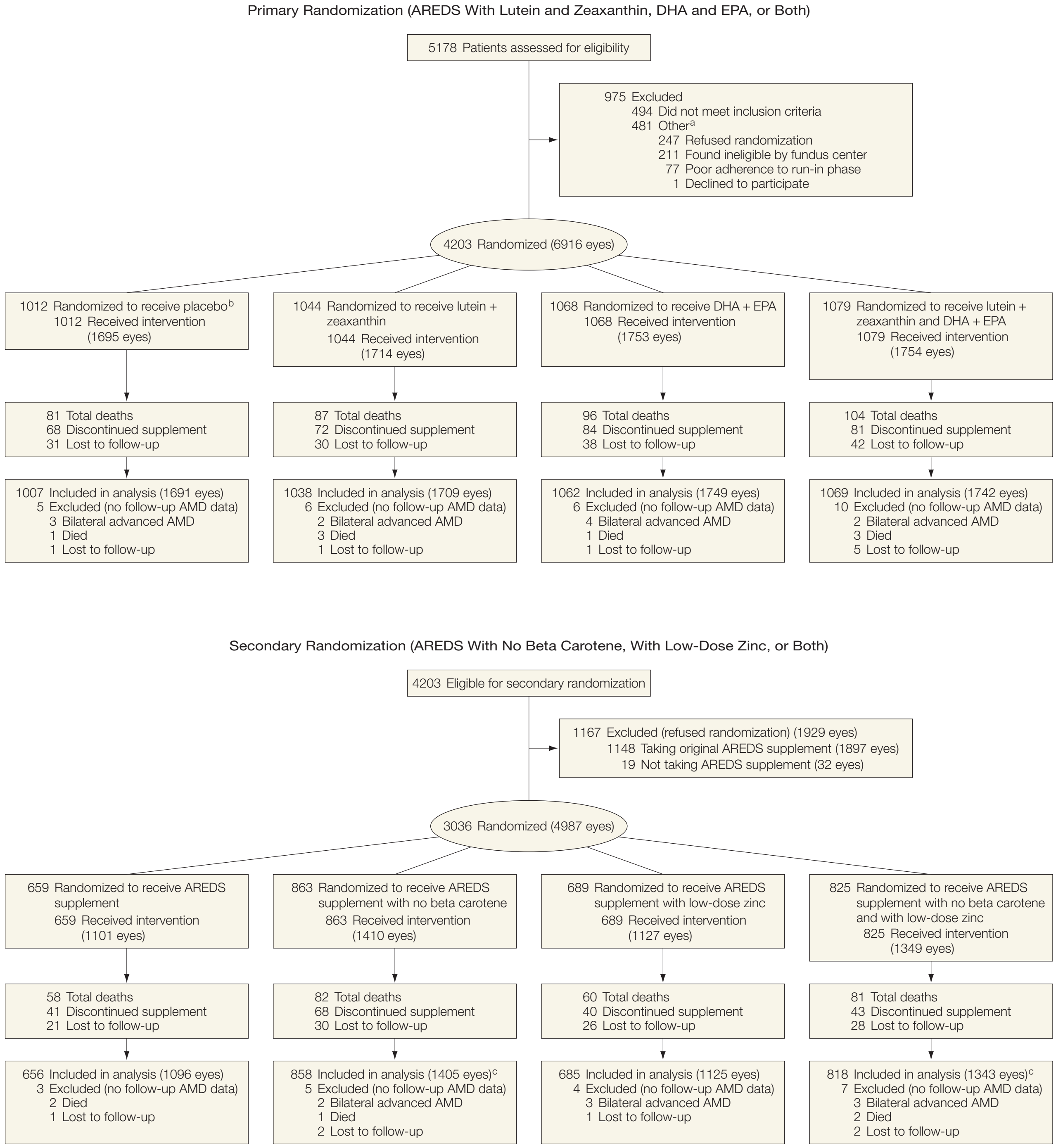

The original Age-Related Eye Disease Study (AREDS) supplement comprised vitamin C (500 mg), vitamin E (400 IU), beta carotene (15 mg), zinc (80 mg, as zinc oxide), and copper (2 mg, as cupric oxide). DHA indicates docosahexaenoic acid; EPA, eicosapentaenoic acid.

[a] Patients could be excluded for more than 1 reason.

[b] The participants assigned to the placebo group were also given the AREDS supplement either within or outside of the secondary randomization for the 4 variations of the AREDS supplements; thus, there is no true placebo group.

[c] Smokers were not randomized to groups receiving beta carotene (n=181, AREDS with no beta carotene; n =166, AREDS with no beta carotene and with low-dose zinc).

Figure 20 AREDS2 study flow and randomization (primary and secondary) (AREDS Research Group 2013)

Table 2

Four alternative AREDS formulations being tested in the secondary randomization of AREDS2

Formulations	Vitamin C	Vitamin E	Beta Carotene	Zinc Oxide	Cupric Oxide
1	500 mg	400 IU	15 mg	80 mg	2 mg
2	500 mg	400 IU	0 mg	80 mg	2 mg
3	500 mg	400 IU	15 mg	25 mg	2 mg
4	500 mg	400 IU	0 mg	25 mg	2 mg

Figure 21 Overview of the four alternative AREDS formulations used in the secondary randomization of AREDS2 (Krishnadev et al. 2010)
