# Supplementary material for: Insights into the nutritional prevention of macular degeneration based on a comparative topic modeling approach
Source: PeerJ Comput Sci. 2024 Mar 20;10:e1940. doi: 10.7717/peerj-cs.1940 (PMC11042009; doi:10.7717/peerj-cs.1940)
Supplement: Supplemental Information 3 [file peerj-cs-10-1940-s003.zip › negative_result_reports/s10792-019-01140-6.pdf]

Int Ophthalmol (2019) 39:2975–2983
https://doi.org/10.1007/s10792-019-01140-6

REVIEW

The value of nutritional supplements in treating Age-Related Macular Degeneration: a review of the literature

Sabrina Mukhtar · Balamurali K. Ambati

Received: 17 November 2018 / Accepted: 6 July 2019 / Published online: 16 July 2019
© Springer Nature B.V. 2019

Abstract
Purpose To describe and evaluate the value of nutritional supplements in the management of age-related macular degeneration (AMD) through a review of the current literature.
Methods An extensive literature search was performed, and key research articles exploring AREDS and AREDS-2 formulations, genetics, omega fatty acids, calcium and folic acid in high-risk women were reviewed. PubMed and Web of Science databases were used for generating articles to review.
Results The AREDS and AREDS-2 trials, while difficult to validate, show support for antioxidant supplementation in reducing AMD progression in Caucasian populations. While genetic guided personalized medicine has been studied mainly with complement factor H and age-related maculopathy susceptibility 2 risk alleles, the data have not been reproducible. Women at a higher risk of cardiovascular disease may benefit from antioxidant therapies in preventing AMD. Omega 3 fatty acid supplementation has been widely supported through observational studies; however, randomized controlled trials have not shown benefit in disease progression. Calcium exposure has been linked to increased mechanisms in cell death and may be detrimental to older individuals with AMD.
Conclusion The data regarding nutritional supplements in preventing AMD progression are inconclusive, and therefore recommendations should be based on risk factors and demographic data.

Keywords Macular degeneration · Nutrition · Public health · Retina

S. Mukhtar (✉)
University of Pittsburgh Medical Center, 203 Lothrop Street, Pittsburgh, PA 15213, USA
e-mail: mukhtars@upmc.edu

B. K. Ambati
Pacific Clear Vision Institute, 112 Darlene Lane, Eugene, OR 97401, USA
e-mail: bambati@pcvi.com

Introduction

Age-related macular degeneration (AMD) is the leading cause of blindness in adults aged 55 and older in the western world [1]. There are 7.3 million people diagnosed with early AMD and 1.75 million Americans suffering from advanced AMD [2]. Early stages of the disease manifest as retinal pigment epithelium abnormalities and drusen near the fovea, while late AMD presents with geographic atrophy or choroidal neovascularization [3]. Though therapies exist for neovascular AMD, no effective treatment is currently available for early and geographic atrophy-related AMD. As a consequence, affected individuals experience significant limitations to independent

functioning without current treatment options. Given the prevalence and impact of the disease on quality of life, The National Eye Institute has identified developing new AMD treatments as an important goal for vision research [4].

In the last decade, many groups aimed to define modifying risk factors, specifically how to address the oxidative changes in the retina during disease progression. Positive behavioral modifications include smoking cessation [5], exercise, and healthy diet; there have been investigations of the utility of supplements to slow down progression of AMD. By identifying nutritional supplements that can help slow progression of non-exudative AMD, the clinician is provided another tool to provide patients with this sight threatening disease. However, consensus on dosage, side effects, baseline dietary intake and long-term results makes it difficult to draw conclusions and recommendations for patients.

This review article aims to identify and evaluate the efficacy of nutritional supplements in slowing progression of AMD and discern the utility for genotype guided nutritional recommendations. Literature search was performed using key words AREDS, AREDS-2, nutritional supplements and acute macular degeneration, genetics, omega fatty acids, calcium and folic acid. PubMed and Web of Science databases were searched from January 1988 to December 2017.

Nutritional supplements and their mechanisms of actions

Oxidative stress seems to play an integral role in disease progression. With age, production of free radicals increase and endogenous defenses decrease [6], creating an environment conducive to damage. The retinal antioxidant network is extensive; however, vitamins C and E, carotenoids, lutein and zeaxanthin are most important in protecting against oxidative stress [6]. Anatomically, alpha- and beta-carotene are concentrated in the macular outer plexiform layer and outer segments of rods and cones. Zeaxanthin is located in the central fovea and lutein resides in the periphery. The major structural lipid in the retina, especially in cones, is docosahexaenoic acid (DHA) [7–9]. More specifically, DHA is involved in membrane permeability and fluidity, and its anti-inflammatory actions inhibit formation of new choroidal vessels, a mechanism that is compromised in neovascular AMD [9].

Additionally, folic acid, vitamin B6 and vitamin B12 lower homocysteine levels, known to cause endothelial dysfunction, and therefore are thought to have a protective effect against progression of AMD [10]. Furthermore, calcium triggers caspase-dependent cell death [11, 12], which can lead to further progressing.

Prevalence of AMD is higher in Caucasians compared to non-white groups suggesting a genetic component [13]. Literature investigating the complement factor H (CFH) and age-related maculopathy susceptibility 2 (ARMS2) genotypes is at the forefront of personalized medicine conversations. CFH binds zinc and neutralizes its ability to inactivate component 3b [14–16]. ARMS2 genotype is linked to mitochondria and affects oxidative phosphorylation processes [17, 18].

Age-related eye disease study (AREDS) and AREDS-2: a hallmark for utility of antioxidant therapy in AMD

Both AREDS and AREDS2 (Table 1) studies aimed to elucidate the protective effects of antioxidants in preventing cell damage in the retina. Physiologically, antioxidants can react with the free radicals produced in the process of light absorption. Theoretically, consumption of higher quantities of antioxidants may reduce the risk of AMD progression [19].

AREDS investigated the utility of antioxidants native to healthy retinas and the effect of their supplementation on progression of AMD. The randomized controlled trial consisted of 4757 patients aged 55–80 years who either took AREDS supplements (vitamin C, vitamin E, beta-carotene, zinc and copper) or placebo [20]. Participants who received the AREDS supplements were diagnosed with at least early AMD. They were split into four categories ranging form early AMD to advanced AMD with vision loss in one eye but good vision in the other eye. After mean follow-up of 6.3 years, AREDS supplementation had strong prevention in disease progression compared to placebo [OR 0.68, 95% CI 0.53–0.87] [19]. Participants taking supplements were less likely to lose 15 letters of vision [OR 0.77, 95% CI 0.62–0.96] [19]. During the study, 407 patients

Table 1 Findings from major trials in patients with Age Related Macular Degeneration

Study/author (year)	Demographics	Study design	Results
AREDS Group (2001)	3640 patients<br>55–80 years old	RCT using placebo, high-dose supplementation with vitamins C and E, beta-carotene, and zinc	Zinc and antioxidants demonstrated significant odds reduction for development of AMD
AREDS2 Group (2013)	4203 patients<br>50–85 years old	RCT using lutein + zeaxanthin, DHA + EPA, lutein + zeaxanthin and DHA + EPA, or placebo in addition to AREDS formulation	Addition of lutein + zeaxanthin, DHA + EPA or both to the AREDS formulation did not reduce the risk of progression to advanced AMD. lutein + zeaxanthin could serve as a carotenoid substitute due to a potential increased incidence of lung cancer in former smokers
Blue Mountain Eye Study (2006)	3654 patients<br>49 years or older	Population-based prospective study	Diet high in omega-3 polyunsaturated fat (fish) protects against early and late ARM
Nutritional AMD Treatment 2 Trial (NAT2) (2013)	263 patients<br>55–85 years old	RCT using DHA + EPA or placebo	DHA was not significantly better than placebo in reducing CNV in unilateral exudative AMD<br>CNV was reduced in DHA patients showing a high EPA + DHA over 3 years (using RBCM measurements)
Women's Antioxidant and Folic Acid Cardiovascular Study (WAFACS) (2008)	5442 women<br>42 years or older	RCT using folic acid + vitamin B6 + B12	35–40% decreased risk of developing AMD

RCT randomized controlled trial, *AMD* acute macular degeneration, *DHA* docosahexaenoic acid, *EPA* eicosapentaenoic acid, *ARM* age-related maculopathy, *CNV* choroid neovascularization, *RBCM* red blood cell membrane

without geographic atrophy at baseline developed at least moderate geographic atrophy [6]. Zinc supplementation, alone or in combination with antioxidants, reduced the risk of progression of Category 3/4 to advanced AMD, with a 25% five year risk reduction compared to placebo; however, this finding was not statistically significant [6]. It is important to note that the concentrations of AREDS vitamin supplements were much higher compared to dietary recommendations. While vitamin C was included in AREDS, the link between vitamin C intake and AMD was not supported in the Pathologies Oculaires Liées á l'Age (POLA) Study or Eye Disease Case Control Study [21–23]. Additionally, beta-carotene supplementation in prevention of AMD is not strongly supported [21]. Importantly, beta-carotene's link to increased risk of cancer in smokers led to the initiation of the AREDS-2 clinical trial.

The AREDS-2 clinical trial aimed to improve the efficacy and safety profile of the AREDS formulation. The original formulation was replaced with lutein and zeaxanthin, which are concentrated in the fovea, and DHA and EPA. The four treatment groups included lutein and zeaxanthin supplementation, DHA and EPA, all four supplements, and placebo group. A total of 4203 participants were enrolled, aged 50–85, who were at risk of progression to advanced AMD with bilateral drusen or large drusen in one eye and with advanced AMD in the other. In the group assigned to the carotenoid supplements, there was a 10% reduction in the progression to advanced AMD compared to placebo, while the group with just DHA and EPA supplementation did not show this effect [3]. Adverse effects were also studied, and lutein and zeaxanthin were not found to increase incidence of lung cancer in smokers; hence, their supplementation could be a better option for people with a history of smoking. While AREDS-2 did not confidently demonstrate the protective effects of lutein and zeaxanthin supplementation on AMD progression, secondary analysis did show protective effects in the subgroup with the lower dietary intake of these carotenoids [3]. These

findings support the possibility of adequate intake of antioxidants through dietary measures.

These trials were conducted in the well-nourished American population, and therefore the results are not generalizable. Additionally, other trials with shorter follow-up, less than 2 years, did not show that these supplements were beneficial [19, 24]. It is crucial to note that because there is a lack of similar independent data of the AREDS trial, it is difficult to validate the study's findings.

Women's Antioxidant and Folic Acid Cardiovascular Study (WAFACS) and AMD

The direct association between blood homocysteine levels and risk of AMD has been well documented in the literature [25–34]. The WAFACS (Table 1) aimed to elucidate benefits of the homocysteine-reducing effect of folic acid and vitamins B6 and B12 on the progression of AMD. Hyperhomocysteinemia is defined as a plasma concentration of greater than 15 mol/L [34–36]. Hyperhomocysteinemia induces vascular endothelial dysfunction, impairs vascular reactivity and promotes inflammatory processes leading to atherosclerosis and cardiovascular disease [37–46]. Studies show that folic acid, vitamin B6 and vitamin B12 lower homocysteine levels and subsequently attenuate endothelial dysfunction [10].

WAFACS is a randomized clinical trial that aimed to examine the incidence of AMD in participants receiving folic acid, vitamins B6 and B12. A total of 5205 female health professionals aged 40 and older with pre-existing cardiovascular disease or three or more cardiovascular risk factors and no evidence of AMD at baseline participated. After 7.3 years of follow-up, a total of 137 AMD cases were documented, 55 of which were in the treatment group and 82 in the placebo group (RR 0.66, 95% CI 0.47–0.93, $p = 0.02$). Additionally, 26 cases in the treatment group and 44 cases in the placebo group developed visually significant AMD (RR 0.59, 95% CI 0.36–0.95, $p = 0.03$) [47]. Two years after treatment, there was a 35–40% decreased risk of AMD in the supplement receiving group compared to placebo, which persisted throughout the trial [47]. The difference in AMD incidence between antioxidant and placebo groups was stratified by age and presence of modifiable risk factors (hypertension, hyperlipidemia, diabetes, prior cardiovascular disease, current hormone replacement therapy use, current multivitamin use, and aspirin use); however, the relative risk reduction did not differ between strata, showing that antioxidant therapy independently attenuates AMD development in high-risk women.

To better determine whether these supplements effectively reduced homocysteine levels, Albert et al. tested mean plasma homocysteine levels in participants receiving the antioxidant treatment. Those in the treatment group had 18.5% less homocysteine levels compared to placebo group (95% CI 12.5–24.1, $p < 0.001$) [48]. However, Bazzano et al. did not find that lowering homocysteine levels in people with pre-existing vascular disease reduced adverse cardiovascular events [49]. While both follow-up studies examined the relationship between homocysteine levels and cardiovascular outcomes, they did not report reducing atherosclerosis which is the main link to AMD [50, 51]. It will be important to investigate whether reduction of homocysteine levels is reflected in subsequent reduction of atherosclerotic markers. The question then becomes whether these supplements are attenuating endothelial dysfunction directly from homocysteine reduction or whether independent processes are also taking place.

Additionally, the subset of the population studied in WAFACS is very limited to high-risk women. Further investigation of the effect of these supplements in both genders and individuals without cardiovascular risk factors is warranted.

Calcium supplementation and AMD

Detailed analysis of subretinal pigment epithelial deposit compositions of patients with known AMD shows high concentrations of calcium [52]. The role of calcium regulation in AMD progression continues to be studied and is supported by the current research showing that calcium dysregulation plays an important role in neurodegenerative diseases like glaucoma, Alzheimer's, Huntington and Parkinson's disease, where excessive calcium has been found to trigger caspase-dependent cell death [11, 12]. Recent studies show similarities between Alzheimer's disease and AMD as both disease processes involve amyloidogenesis, plaque formation, inflammation, oxidative stress and glial functional change [53–56]. Similar

calcium-dependent pathologic mechanisms established in the Alzheimer's disease model can occur in AMD, a key characteristic being age as a predisposing factor for progression.

To better assess the link between dietary calcium intake and AMD progression, Kaigi et al. investigated the effect of self-reported calcium intake and AMD diagnosis in a cross sectional study. Calcium consumption and fundus evaluation for AMD were recorded across 3191 participants aged 40 and older who participated in the National Health and Nutrition Examination Survey (NHANES) [57]. A total of 248 participants (7.1%) were diagnosed with AMD. After adjustment for confounding variables, patients with self-reported calcium consumption greater than 800 mg had a higher odds of AMD (OR 1.85, 95% CI 1.25–2.75) [57]. A dose relationship between calcium consumption and an AMD diagnosis was not seen; however, a threshold phenomenon was observed. Of note, upon age stratification, there was a stronger association between calcium consumption and AMD in older (aged 68 and older) compared to younger individuals (aged 40–67) (OR 2.63, 95% CI 1.52–4.54) [57]. This observation may be secondary to longer periods of calcium exposure in older individuals and age-related dysfunction of cell membranes that maintain calcium homeostasis [12].

While Kaigi et al. provided a convincing argument for the possibility of calcium supplementation being linked to the higher prevalence of AMD, there is limited literature showing this link and therefore more studies are warranted to validate their findings. Furthermore, the 800 mg/d cut off as the highest quintile of calcium supplementation is still lower than the total daily intake of calcium for men and women in the USA [58]. Calcium supplementation as a risk factor for AMD progression needs to be studied further to better guide patients with predisposition for AMD.

Omega 3 supplementation and AMD

Omega 3 fatty acids act through their renewing capabilities in RPE cells and, when deficient, can lead to photoreceptor degradation and accumulation of lipid filled drusen in the RPE and sub-RPE space [3]. High DHA levels in oily fish have been associated with decreased risk of neovascular AMD [59, 60]. The Eye Disease Case Control Study demonstrated an association between the higher intake of n-3 fatty acids and the lower risk of AMD among individuals with the lower linoleic acid (omega 6 fatty acid) consumption [61, 62]. The Blue Mountains Eye Study (Table 1) observed protective effects of n-2 fatty acids in late AMD [63]. The Nutritional AMD Treatment 2 (NAT2) Trial (Table 1) demonstrated that individuals with high content of eicosaphentaenoic acid (EPA) and DHA in their red blood cell membranes were significantly protected against AMD compared to the their counterparts with lower concentrations [59, 60]. Georgiou et al. showed that daily high doses of EPA (3.4 g) and DHA (1.6 g) for six months were associated with improved visual acuity in patients with dry AMD [64]. However, Querques et al. reported that DHA supplementation alone for 3 years had no effect on the drusen remodeling process [65]. Additionally, a 2015 Cochrane meta-analysis concluded that there is no evidence to support that omega 3 PUFA supplementation prevents or slows progression of AMD with the AREDS-2 trial data [66, 67].

Overall, while the literature may show a positive effect of omega 3 fatty acid supplementation as a preventative measure of AMD progression, most studies are observational and unsupported by randomized controlled trials [66, 67]. It would be reasonable for the clinician to advise patients to consume fatty fish or omega 3 fatty acid supplements as being possibly beneficial in reducing AMD progression while informing patients that most data are based on observational studies.

The role of genetics in nutritional supplementation

Analyses related to the differential effect of AREDS supplementation among genotype groups have been inconclusive. Individuals with homozygous CFH or ARMS2 risk alleles have a higher rate of disease progression compared to subjects homozygous for non-risk alleles (HR 1.64, 95% CI 1.3–2.97, $p < 0.0001$) and (HR 2.44, CI 95% 1.96–3.02, $p < 0.001$), respectively [68]. While there are many genotype subgroups associated with AMD progression, rare risk alleles (C2, CFB) and small effect sizes (ATP binding cassette family, lipoprotein lipase) were not identified in the literature as significant predictors of risk in the AREDS study population. Awh et al. reported that over an average of 10.2 years,

participants with one or two CFH risk alleles derived maximum benefit from antioxidant therapy alone (in the AREDS formulation) compared to no beneficial effect seen with zinc supplementation [69]. Furthermore, zinc supplementation with antioxidants had a hazard ratio of 1.83 ($p < 0.001$) in developing disease progression compared to subjects without CFH risk alleles [69]. As aforementioned, zinc binds with CFH and hinders its ability to inactivate component 3b [14–16]. Increased progression of disease was found in antioxidant treatment alone in individuals with an ARMS2 risk allele, $p < 0.001$ [69]. The authors suggest that the benefit of antioxidants completely disappears in the presence of two ARMS2 risk alleles, which can be explained by the localization to mitochondria and subsequent effect on oxidative phosphorylation [17, 18]. The study concluded that based on distribution of genetic risk alleles, the average 10-year progression rate for patient populations treated with placebo was 47%, AREDS formulation was 40.5% and with genotype directed therapy was 31.7%, suggesting utility in genotype directed genotype nutritional therapy [69].

The data hold promise for patients with CFH risk alleles to undergo antioxidant therapy only, and ARMS2 risk allele groups to utilize non-antioxidant therapies in AMD prevention. However, several limitations exist. First, the study group does not investigate zinc alone in ARMS2 groups and antioxidants in CFH groups, partly limited by previously established AREDS data. As such, direct correlation cannot be extrapolated. Second, genetics of AMD have mostly been studied in Caucasian populations, limiting generalization to other races. Furthermore, colleagues could not reproduce the data generated by Awh et al. Chew et al. repeated statistical analysis and found no benefit of genotype directed therapy with AREDS formulation and interaction analysis found no evidence of relative benefits with varied genotypes [70]. However, Chew and colleagues ran statistical analysis on more subgroups and therefore the study lacked power in their analysis [71]. The Rotterdam Study supported Awh et al., reporting that dietary intake of antioxidants reduces risk of early AMD in those at high CFH and ARMS2 genetic risk [72].

Since all AREDS2 formulations contained zinc, analysis of the study's data could not be reported. However, it is important to note that AREDS2 found no significant difference between lower or higher doses of zinc on AMD progression, suggesting that the effect on zinc on CFH risk allele genotypes is not dose related [69].

Furthermore, genetic differences may explain 67% of variability in macular pigment optical density and 27% of variability in response to lutein and zeaxanthin supplementation over a course of 6 months in women participants of the AREDS Study [73–75]. Therefore, concentrations of AREDS or antioxidants, mainly lutein and zexanthin, vary more than tenfold among individuals [74]. No genetic studies investigating the differences of omega 3 fatty acids, calcium supplementation and vitamins B6 and B12 have been published during this literature review to draw further conclusion of the utility of genotyping when making these nutritional recommendations. Interestingly, methylenetetrahydrofolate reductase gene (*MTHFR* C677T) has been associated with elevated homocysteine serum levels [76–78].

Overall, the role of genetics in personalized medicine for AMD is inconclusive and limited. When data are not collected rigorously enough, erroneous conclusions can be drawn regarding an unknown biological process. At this point, pharmacogenomic recommendations remain difficult to determine based on genetic profile.

Conclusion

With AMD being a significant public health issue, it is important to understand and identify therapies to attenuate disease progression. Unfortunately, there is no supplementary regimen supported with strong data to prevent the progression of AMD. Antioxidant supplementation, based on the AREDS and AREDS-2 trials, may have better utility in malnourished communities. However, current evidence-based recommendations should be limited to Caucasians with AMD. Due to inconclusive data and cost, recommendations based on genetic variants are limited; therefore, genetic testing may be unwarranted at this time. Women with AMD and high cardiovascular risk factors based on family and demographic data can be offered a combination of vitamins B6, B12 and folic acid. Lastly, of the supplements that have been investigated, omega 3 fatty acids, especially EPA and DHA, are supported via observational studies, but the lack of support via randomized controlled trials

argues against their beneficial effect. After reviewing the literature, no one nutritional supplement can be attributed to confidently slowing the progression of AMD. It is to the discretion of the clinician to determine the population subset their patient falls under and patient motivation to advise accordingly with emphasis to inform patients of the limitations of the current literature.

Compliance with ethical standards

Conflict of interest The authors declare that they have no conflict of interest.

References

1. Rojas-Fernandez CH, Tyber K (2016) Benefits, potential harms, and optimal use of nutritional supplementation for preventing progression of age-related macular degeneration. Ann Pharmacol 51:1–7
2. Friedman DS, O'Colmain BJ, Munoz B, Tomany SC, McCarty C, de Jong P, Nemesure B, Mitchell P, Kempen J (2004) Eye DIsease prevalence group. Arch Ophthalmol 122(4):564–572
3. Carneiro A, Andrade JP (2017) Nutritional and lifestyle interventions for age related macular degeneration: a review. Oxid Med Cell Longev 2017:6469138
4. National Eye Institute (2006) National plan for eye and vision research: retinal disease program. Eye Vision Res 1–43
5. Chakravarthy U, Augood C, Bentham GC et al (2007) Cigarette smoking and age-related macular degeneration in the EUREYE Study. Ophthalmology 358(24):2606–2617
6. Carneiro A (2015) Anti-ageing nutrients evidence-based prevention of age-related diseases. Nutrition and the ageing eye. Wiley, Hoboken, NJ
7. Sparrow JR, Vollmer-Snarr HR, Zhou J, Jang YP, Jockusch S, Itagaki Y, Nakanishi K (2003) A2E-epoxides damage DNA in retinal pigment epithelial cells. Vitamin E and other antioxidants inhibit A2E-epoxide formation. J Biol Chem 278(20):18207–18213
8. Conner WE, Neuringer M (1988) The effects of n-3 fatty acid deficiency and repletion upon the fatty acid composition and function of the brain and retina. Prog Clin Biol Res 282:275–294
9. SanGiovanni JP, Chew EY (2005) The role of omega-3 long-chain polyunsaturated fatty acids in health and disease of the retina. Prog Retin Eye Res 24(1):87–138. https://doi.org/10.1016/j.preteyeres.2004.06.002
10. Homocysteine Lowering Trialists' Collaboration (2005) Dose-dependent effects of folic acid on blood concentrations of homocysteine: a meta-analysis of the randomized trials. Am J Clin Nutr 82(4):806–812
11. Crish SD, Calkins DJ (2011) Neurodegeneration in glaucoma. Neuroscience 176(1):1–11
12. Wojda U, Salinska E, Kuznicki J (2008) Calcium ions in neuronal degeneration. IUBMB Life 60(9):575–590
13. Chen Y, Bedell M, Zhang K (2010) Age-related macular degeneration: genetic and environmental factors of disease. Mol Interv 10(5):271–281. https://doi.org/10.1124/mi.10.5.4
14. Nan R, Gor J, Lengyel I, Perkins SJ (2008) Uncontrolled zinc- and copper-induced oligomerisation of the human complement regulator factor H and its possible implications for function and disease. J Mol Biol 384(5):1341–1352. https://doi.org/10.1016/j.jmb.2008.10.030
15. Perkins SJ, Nan R, Li K, Khan S, Miller A (2012) Complement factor H-ligand interactions: self-association, multivalency and dissociation constants. Immunobiology 217(2):281–297. https://doi.org/10.1016/j.imbio.2011.10.003
16. Nan R, Farabella I, Schumacher FF, Miller A, Gor J, Martin AC, Jones DT, Lengyel I, Perkins SJ (2011) Zinc binding to the Tyr402 and His402 allotypes of complement factor H: possible implications for age-related macular degeneration. J Mol Biol 408(4):714–735. https://doi.org/10.1016/j.jmb.2011.03.006
17. Calder PC, Albers R, Antoine JM, Blum S, Bourdet-Sicard R, Ferns GA, Folkerts G, Friedmann PS, Frost GS, Guarner F, Lovik M, Macfarlane S, Meyer PD, M'Rabet L, Serafini M, van Eden W, van Loo J, Vas Dias W, Vidry S, Winklhofer-Roob BM, Zhao J (2009) Inflammatory disease processes and interactions with nutrition. Br J Nutr 101(Suppl 1):S1–45. https://doi.org/10.1017/S0007114509377867
18. Fritsche LG, Loenhardt T, Janssen A, Fisher SA, Rivera A, Keilhauer CN, Weber BH (2008) Age-related macular degeneration is associated with an unstable ARMS2 (LOC387715) mRNA. Nat Genet 40(7):892–896. https://doi.org/10.1038/ng.170
19. Evans JR, Lawrenson JG (2012) Antioxidant vitamin and mineral supplements for slowing the progression of age-related macular degeneration. Cochrane Database Syst Rev. https://doi.org/10.1002/14651858.CD000254.pub3
20. Age Related Eye Disease Study Research Group (2001) A randomized, placebo-controlled, clinical trial of high-dose supplementation with vitamins C and E, beta carotene, and zinc for age-related macular degeneration and vision loss: AREDS report no. 8. Arch Ophthalmol 119(10):1417–1436
21. Zampatti S, Ricci F, Cusumano A, Marsella LT, Novelli G, Giardina E (2013) Review of nutrient actions on age-related macular degeneration. Nutr Res 34(2):95–105
22. Delcourt C, Carriere I, Delage M, Barberger-Gateau P, Schalch W, POLA Study Group (2006) Plasma lutein and zeaxanthin and other carotenoids as modifiable risk factors for age-related maculopathy and cataract: the POLA study. Invest Ophthalmol Vis Sci 47(6):2329–2335
23. Eye Disease Case-Control Study Group (1993) Antioxidant status and neovascular age-related macular degeneration. Arch Ophthalmol 111:104–109
24. Chong EW, Wong TY, Kreis AJ, Simpson JA, Guymer RH (2007) Dietary antioxidants and primary prevention of age related macular degeneration: systematic review and meta-analysis. BMJ 335(7623):755
25. Heuberger RA, Al Fisher, Jacques PF et al (2002) Relation of blood homocystein and its nutritional determinants to age-related maculopathy in the third National Health and Nutrition Examination Survey. Am J Clin Nutr 76(4):897–902

26. Axer-Siegal R, Bourla D, Ehrlich R et al (2004) Association of neovascular age-related macular degeneration and hyperhomocysteinemia. Am J Ophthalmol 137(1):84–89
27. Rochtchina E, Wang JJ, Flood VM, Mitchell P (2007) Elevated serum homocysteine, low serum vitamin B12, folate, and age-related macular degeneration: the blue mountains eye study. Am J Ophthalmol 143(2):344–346
28. Nowak M, Swietochowska E, Wielkoszynski T et al (2005) Homocysteine, vitamin B12, and folic acid in age-related macular degeneration. Eur J Ophthalmol 15(6):764–767
29. Vine AK, Stader J, Branham K, Musch DC, Swaroop A (2005) Biomarkers of cardiovascular disease as risk factors for age-related macular degeneration. Ophthalmology 112(12):2076–2080
30. Coral K, Raman R, Rathi S et al (2006) Plasma homocysteine and total thiol content in patients with exudative age-related macular degeneration. Eye 20(2):203–207
31. Kamburoglu G, Gumus K, Kadayifcilar S, Eldem B (2006) Plasma homocysteine, vitamin B12 and folate levels in age-related macular degeneration. Graefes Arch Clin Exp Ophthalmol 244(5):565–569
32. Seddon JM, Gensler G, Klein ML, Milton RC (2006) Evaluation of plasma homocystein and risk of age-related macular degeneration. Am J Ophthalmol 141(1):201–203
33. Refsum H, Ueland PM, Nygard O, Vollset SE (1998) Homocysteine and cardiovascular disease. Annu Rev Med 49:31–62
34. Ueland PM, Refsum H, Stabler SP, Malinow MR, Andersson A, Allen RH (1993) Total homocysteine in plasma or serum: methods and clinical applications. Clin Chem 39(9):1764–1779
35. Welch GN, Loscalzo J (1998) Homocysteine and atherothrombosis. N Engl J Med 338(15):1042–1050
36. Chambers JC, Obeid OA, Kooner JS (1999) Physiological increments in plasma homocysteine induce vascular endothelial dysfunction in normal human subjects. Arterioscler Thromb Vasc Biol 19(12):2922–2927
37. Domagala TB, Undas A, Libura M, Szczeklik A (2000) Pathogenesis of vascular disease in hyperhomocysteinaemia. J Cardiovasc Risk 5(4):239–247
38. McDowell IF, Lang D (2000) Homocysteine and endothelial dysfunction: a link with cardiovascular disease. J Nutr 130(2Suppl):369S–372S
39. Homocysteine Studies Collaboration (2001) Homocysteine and risk of ischemic heard disease and stroke: a meta analysis. JAMA 288(16):2015–2022
40. Wald DS, Law M, Morris JK (2002) Homocysteine and risk of ischemic heart disease and stroke: a meta-analysis. JAMA 288(16):2015–2022
41. Nappo F, De Rosa N, Marfella R et al (1999) Impairment of endothelial functions by acute hyperhomocysteinemia and reversal by antioxidant vitamins. JAMA 281(22):2113–2118
42. Upchurch GR, Welch GN, Fabian AJ et al (1997) Homocyst(e)ine decreases bioavailable nitric oxide by a mechanism involving glutathione peroxidase. J Biol Chem 272(20):17012–17017
43. Stuhlinger MC, Tsao PS, Her JH, Kimoto M, Balint RF, Cooke JP (2001) Homocysteine impairs the nitric oxide synthase pathway: role of asymmetric dimethylglargine. Circulation 104(21):2569–2575
44. Silverman MD, Tumuluri RJ, Davis M, Lopez G, Rosenbaum JT, Lelkes PI (2002) Homocysteine upregulates vascular cell adhesion molecule-1 expression in cultured human aortic endothelial cells and enhances monocyte adhesion. Arterioscler Thromb Vasc Biol 22(4):587–592
45. Tsai JC, Perrella MA, Yoshizumi M et al (1994) Promotion of vascular smooth muscle cell growth by homocysteine: a link to atherosclerosis. Proc Natl Acad Sci USA 91(14):6369–6373
46. Dudman NP, Temple SE, Guo XW, Fu W, Perry MA (1999) Homocysteine enhances neutrophil–endothelial interactions in both cultured human cells and rats in vivo. Circ Res 84(4):409–416
47. Christen WG, Glynn RJ, Chew EY, Albert CM, Manson JE (2009) Folic acid, vitamin B6, and vitamin B12 in combination and age-related macular degeneration in a randomized trial of women. Arch Intern Med 169:335341
48. Albert CM, Cook NR, Gaziano JM et al (2008) Effect of folic acid and B vitamins on risk of cardiovascular events and total mortality among women at high risk for cardiovascular disease: a randomized trial. JAMA 299(17):2027–2036
49. Bazzano LA, Raeynolds K, Holder KN, He J (2006) Effect of folic acid supplementation on risk of cardiovascular disease: a meta analysis of randomized controlled trials. JAMA 296(22):2720–2726
50. Vingerling JR, Dielmans I, Bots ML, Hofman A, Grobbee DE, de Jong PT (1995) Age-related macular degeneration is associated with atherosclerosis. The Rotterdam Study. Am J Epidemiol 142(4):404–409
51. Snow KK, Seddon JM (1999) Do age-related macular degeneration and cardiovascular disease share common antecedents? Ophthalmol Epidemiol 6(2):125–143
52. Flinn JM, Kakalec P, Tapero R, Jones B, Lengyel I (2014) Correlations in distribution and concentration of calcium, copper and iron with zinc in isolated extracellular deposits associated with age-related macular degeneration. Metallomics 6(7):1223–1228
53. Logue MW, Schu M, Vardarajan BN et al (2014) Search for age-related macular degeneration risk variants in Alzheimer disease genes and pathways. Neurobiol Aging 35(6):1510.e1517–1510.e1518
54. Zhao Y, Battacharjee S, Jones BM et al (2015) Beta-amyloid precursor protein (BAPP) processing in Alzheimer's disease (AD) and age-related macular degeneration (AMD). Mol Neurobiol 52(1):533–544
55. Sivak JM (2013) The aging eye. Invest Ophthalmol Vis Sci 54(1):871–880
56. Harvey H, Durant S (2014) The role of glial cells and the complement system in retinal diseases and Alzheimer's disease. Exp Brain Res 232(11):3363–3377
57. Kaigi CL, Sing K, Wang SY, Enanoria WT, Lin SC (2015) Self-reported calcium supplementation and age-related macular degeneration. JAMA Ophthalmol 133(7):746–754
58. Bilezikian JP (1994) NIH consensus development panel on optimal calcium intake. NIH consensus conference. JAMA 272(24):1942–1948
59. Souied EH, Delcourt C, Querques G et al (2013) Oral docosahexaenoic acid in the prevention of exudative age-related macular degeneration: the nutritional AMD treatment 2 study. Ophthalmology 120(8):1619–1631

60. Querques G, Souied EH (2014) The role of omega-3 and micronutrients in age-related macular degeneration. Surv Ophthalmol 59(5):532–539
61. Aoki A, Inoue M, Nguyen E et al (2016) Dietary n-3 fatty acid, alpha-tocopherol, zinc, vitamin D, vitamin C, and Beta-carotene are associated with age-related macular degeneration in Japan. Sci Rep 6:20723
62. Seddon JM, George S, Rosner B (2006) Cigarette smoking, fish consumption, omega-3 fatty acid intake, and associations with age-related macular degeneration: the US twin study of age-related macular degeneration. Arch Ophthalmol 124(7):995–1001
63. Chua B, Flood V, Rochtchina E, Wang JJ, Smith W, Mitchell P (2006) Dietary fatty acids and the 5 year incidence of age-related maculopathy. Arch Ophthalmol 124(7):981–986
64. Georgiou T, Neokleous A, Nicolaou D, Sears B (2014) Pilot study for treating dry age-related macular degeneration (AMD) with high-dose omega-3 fatty acids. PharmaNutrition 2(1):8–11
65. Querques G, Merle BMJ, Pumariega NM et al (2016) Dynamic drusen remodelling in participants of the nutritional AMD treatment-2 (NAT-2) randomized trial. PLoS ONE 11(2):e149
66. Lawrenson JG, Evans JR (2012) Omega 3 fatty acids for preventing or slowing the progression age-related macular degeneration. Cochrane Database Syst Rev. https://doi.org/10.1002/14651858.CD010015.pub3
67. Lawrenson JG, Evans JR (2015) Omega 3 fatty acids for preventing or slowing progression of age-related macular degeneration. Cochrane Database Syst Rev. https://doi.org/10.1002/14651858.CD010015.pub2
68. Seddon JM, Silver RE, Rosner B (2016) Response to AREDS supplements according to genetic factors: survival analysis approach using the eye as the unit of analysis. Br J Ophthalmol 100(12):1731–1737. https://doi.org/10.1136/bjophthalmol-2016-308624
69. Awh CC, Lane AM, Hawken S, Zanke B, Kim IK (2013) CFH and ARMS2 genetic polymorphisms predict response to antioxidants and zinc in patients with age-related macular degeneration. Ophthalmology 120(11):2317–2323. https://doi.org/10.1016/j.ophtha.2013.07.039
70. Chew EY, Klein ML, Clemons TE, Agron E, Ratnapriya R, Edwards AO, Fritsche LG, Swaroop A, Abecasis GR, Age-Related Eye Disease Study Research Group (2014) No clinically significant association between CFH and ARMS2 genotypes and response to nutritional supplements: AREDS report number 38. Ophthalmology 121(11):2173–2180. https://doi.org/10.1016/j.ophtha.2014.05.008
71. Awh CC, Hawken S, Zanke BW (2015) Treatment response to antioxidants and zinc based on CFH and ARMS2 genetic risk allele number in the age-related eye disease study. Ophthalmology 122(1):162–169. https://doi.org/10.1016/j.ophtha.2014.07.049
72. Ho L, van Leeuwen R, Witteman JC, van Duijn CM, Uitterlinden AG, Hofman A, de Jong PT, Vingerling JR, Klaver CC (2011) Reducing the genetic risk of age-related macular degeneration with dietary antioxidants, zinc, and omega-3 fatty acids: the Rotterdam study. Arch Ophthalmol 129(6):758–766. https://doi.org/10.1001/archophthalmol.2011.141
73. Liew SH, Gilbert CE, Spector TD, Mellerio J, Marshall J, van Kuijk FJ, Beatty S, Fitzke F, Hammond CJ (2005) Heritability of macular pigment: a twin study. Invest Ophthalmol Vis Sci 46(12):4430–4436. https://doi.org/10.1167/iovs.05-0519
74. Meyers KJ, Johnson EJ, Bernstein PS et al (2013) Genetic determinants of macular pigments in women of the carotenoids in age related eye disease study. Invest Ophthalmol Vis Sci 54:2333–2345
75. Hammond CJ, Liew SH, Van Kuijk FJ, Beatty S, Nolan JM, Spector TD, Gilbert CE (2012) The heritability of macular response to supplemental lutein and zeaxanthin: a classic twin study. Invest Ophthalmol Vis Sci 53(8):4963–4968. https://doi.org/10.1167/iovs.12-9618
76. Rodrigo R, Passalacqua W, Araya J, Orellana M, Rivera G (2003) Homocysteine and essential hypertension. J Clin Pharmacol 43(12):1299–1306. https://doi.org/10.1177/0091270003258190
77. Casas JP, Hingorani AD, Bautista LE, Sharma P (2004) Meta-analysis of genetic studies in ischemic stroke: thirty-two genes involving approximately 18,000 cases and 58,000 controls. Arch Neurol 61(11):1652–1661. https://doi.org/10.1001/archneur.61.11.1652
78. Stankovic S, Majkic-Singh N (2010) Genetic aspects of ischemic stroke: coagulation, homocysteine, and lipoprotein metabolism as potential risk factors. Crit Rev Clin Lab Sci 47(2):72–123. https://doi.org/10.3109/10408361003791520

Publisher's Note Springer Nature remains neutral with regard to jurisdictional claims in published maps and institutional affiliations.
