# Supplementary material for: Insights into the nutritional prevention of macular degeneration based on a comparative topic modeling approach
Source: PeerJ Comput Sci. 2024 Mar 20;10:e1940. doi: 10.7717/peerj-cs.1940 (PMC11042009; doi:10.7717/peerj-cs.1940)
Supplement: Supplemental Information 3 [file peerj-cs-10-1940-s003.zip › negative_result_reports/nutrients-13-01253-v2.pdf]

nutrients

MDPI

Article

A Randomized Study of Nutritional Supplementation in Patients with Unilateral Wet Age-Related Macular Degeneration

Alfredo García-Layana [1,2,3], Sergio Recalde [1,2,3,*], Maria Hernandez [1,2,3], Maximino J. Abraldes [3,4], João Nascimento [5], Emiliano Hernández-Galilea [6], Begoña Olmedilla-Alonso [7], Jose Juan Escobar-Barranco [8], Miguel Angel Zapata [9], Rufino Silva [10,11,12], Mariana Caballero Arredondo [13], María Carmen Lopez-Sabater [13], Silvia Mendez-Martínez [14,15], Nieves Pardiñas-Barón [14,15], Pilar Calvo [14,15] and Patricia Fernández-Robredo [1,2,3]

1 Retinal Pathologies and New Therapies Group, Experimental Ophthalmology Laboratory, Department of Ophthalmology, Clínica Universidad de Navarra, Av. de Pío XII, 36, 31008 Pamplona, Spain; aglayana@unav.es (A.G.-L.); mahersan@unav.es (M.H.); pfrobredo@unav.es (P.F.-R.)
2 Navarra Institute for Health Research, IdiSNA, C/Irunlarrea, 3, 31008 Pamplona, Spain
3 Red Temática de Investigación Cooperativa Sanitaria en Enfer-Medades Oculares (Oftared), Instituto de Salud Carlos III, Av. de Monforte de Lemos 5, 28029 Madrid, Spain; maxiabraldes@gmail.com
4 Department of Ophthalmology, Complexo Hospitalario Universitario de Santiago de Compostela, Universidad de Santiago de Compostela, 15706 Santiago de Compostela, Spain
5 Retinal Department, Ophthalmology, Retina Lisbon Institut, Av. Duque de Loulé Nº5, 1050-085 Lisbon, Portugal; joaocnascimento@sapo.pt
6 Department of Ophthalmology, University of Salamanca, University Hospital of Salamanca, Paseo de San Vicente 182, 37007 Salamanca, Spain; egalilea@usal.es
7 Department of Metabolism and Nutrition, Institute of Food Science, Technology and Nutrition (ICTAN-CSIC), José Antonio Novais 10, 28040 Madrid, Spain; BOlmedilla@ictan.csic.es
8 Department of Ophthalmology, Hospital Dos de Maig, Carrer del Dos de Maig 301, 08025 Barcelona, Spain; escobarjou@yahoo.es
9 Department of Ophthalmology, Hospital Vall Hebron, Passeig de la Vall d'Hebron 119, 08035 Barcelona, Spain; zapatavictori@hotmail.com
10 Faculty of Medicine, Coimbra Institute for Clinical and Biomedical Research (iCBR), University of Coimbra, Paço das Escolas, 3004-531 Coimbra, Portugal; rufino.silva@oftalmologia.co.pt
11 Ophthalmology Department, Centro Hospitalar e Universitário de Coimbra (CHUC), Praceta Professor Mota Pinto, 3004-561 Coimbra, Portugal
12 Association for Innovation and Biomedical Research on Light and Image (AIBILI), Azinhaga Sta. Comba, 3000-548 Coimbra, Portugal
13 Nutrition, Food Science and Gastronomy, University of Barcelona, Joan XXIII, 27-31, 08028 Barcelona, Spain; mariana.cbllro@gmail.com (M.C.A.); mclopez@ub.edu (M.C.L.-S.)
14 Department of Ophthalmology, Miguel Servet University Hospital, Paseo Isabel la Católica 1-3, 50009 Zaragoza, Spain; silviamendezmartinez@hotmail.com (S.M.-M.); npardinasb@yahoo.com (N.P.-B.); xenatrance@yahoo.es (P.C.)
15 Miguel Servet Ophthalmology Research Group (GIMSO), Aragon Health Research Institute (IIS Aragon), University of Zaragoza, Avda. De Juan Bosco 13, 50009 Zaragoza, Spain
* Correspondence: srecalde@unav.es; Tel.: +34-948425600 (ext. 806499)

check for updates

Citation: García-Layana, A.; Recalde, S.; Hernandez, M.; Abraldes, M.J.; Nascimento, J.; Hernández-Galilea, E.; Olmedilla-Alonso, B.; Escobar-Barranco, J.J.; Zapata, M.A.; Silva, R.; et al. A Randomized Study of Nutritional Supplementation in Patients with Unilateral Wet Age-Related Macular Degeneration. *Nutrients* **2021**, *13*, 1253. https://doi.org/10.3390/nu13041253

Academic Editor: Jean Christopher Chamcheu

Received: 3 March 2021
Accepted: 7 April 2021
Published: 10 April 2021

Publisher's Note: MDPI stays neutral with regard to jurisdictional claims in published maps and institutional affiliations.

Copyright: © 2021 by the authors. Licensee MDPI, Basel, Switzerland. This article is an open access article distributed under the terms and conditions of the Creative Commons Attribution (CC BY) license (https://creativecommons.org/licenses/by/4.0/).

Abstract: The purpose of this study is evaluate the efficacy and safety of medicinal products containing the original Age-Related Eye Disease group (AREDS) formulation at doses approved in Europe (EU, control group; $n = 59$) with a product that adds DHA, lutein, zeaxanthin, resveratrol and hydroxytyrosol to the formula (intervention group; $n = 50$). This was a multicenter, randomized, observer-blinded trial conducted in patients aged 50 years or older diagnosed with unilateral exudative Age related Macular Degeneration AMD. At month 12, the intervention did not have a significant differential effect on visual acuity compared with the control group, with an estimated treatment difference in Early Treatment Diabetic Retinopathy Study (ETDRS) of −1.63 (95% CI −0.83 to 4.09; $p = 0.192$). The intervention exhibited a significant and, in most cases, relevant effect in terms of a reduction in some inflammatory cytokines and a greater improvement in the fatty acid profile and serum lutein and zeaxantin concentration. In patients with unilateral wet AMD, the addition

of lutein, zeaxanthin, resveratrol, hydroxytyrosol and DHA to the AREDS EU recommended doses in the short-term did not have a differential effect on visual acuity compared to a standard AREDS EU formula but, in addition to improving the fatty acid profile and increasing carotenoid serum levels, may provide a beneficial effect in improving the proinflammatory and proangiogenic profile of patients with AMD.

Keywords: age-related macular degeneration; AREDS; Theavit®; Retilut®; carotenoids; polyunsaturated fatty acids; inflammatory markers; angiogenic factors at month 12

1. Introduction

Age-related macular degeneration (AMD) is a leading cause of loss of vision and is associated with a substantial burden for the individual. Globally, the prevalence of AMD has been estimated to be 8.7% in individuals aged 45 to 85 years [1]. In Europe, despite the observation of a decrease in the prevalence in the last two decades, it is expected that the number of affected subjects will increase, with AMD remaining a significant public health problem [2]. In 2015, AMD was the fourth cause of blindness globally and the third cause of moderate-to-severe visual impairment [1]. Individuals with AMD may exhibit an important deterioration in quality of life depending on visual acuity and other factors, such as the disease stage and comorbidities [3–5].

Advanced forms of AMD include geographic atrophy or dry AMD and choroidal neovascularization or wet AMD. Anti-vascular endothelial growth factor (anti-VEGF) agents are effective in patients with wet AMD in terms of maintaining visual acuity [6] and should therefore be considered a standard of care for these patients [7]. Despite its proven efficacy, due to the difficulties in implementing strict intravitreal treatment patterns in clinical practice, anti-VEGFs may not be associated with the expected outcomes [8]. In addition, dry AMD is a more resistant form of the disease, and no drug has yet been approved for its treatment. Therefore, there is considerable interest in identifying therapeutic options that could delay the occurrence of advanced forms of the disease.

The recognition of the role of oxidative stress in macular degeneration and the fact that the retina is particularly susceptible to it has led to the proposal of several antioxidants for reducing the risk of progression of AMD [9]. In this context, a large, multicenter, randomized trial, namely, the Age-Related Eye Disease Study (AREDS), demonstrated that a supplement containing vitamin C, vitamin E, beta-carotene and zinc compared to placebo reduced the five-year risk of developing AMD by 25% in patients at risk, a modest but significant benefit [10]. Afterward, in light of the potential antioxidant benefit of other micronutrients and potential risks of beta-carotene, the AREDS2 randomized trial was conducted to evaluate, using a factorial design, the efficacy and safety of the addition of lutein plus zeaxanthin and/or omega-3 long-chain polyunsaturated fatty acids (LCPUFAs) to the original formula and to assess the omission of beta-carotene and/or reduction in the dose of zinc from the formula [11]. The primary analysis of the trial did not find a further reduction in the risk of AMD with the addition of lutein plus zeaxanthin and/or omega-3 LCPUFAs. However, a secondary analysis showed that individuals randomized to lutein plus zeaxanthin and the AREDS formula without beta-carotene compared to those who received no lutein plus zeaxanthin and the AREDS formula containing beta-carotene exhibited a significant 18% reduction in the likelihood of progression to advanced AMD and a 22% reduction in the likelihood of developing neovascular AMD; there was no significant effect on the evolution of central geographic atrophy [12]. Further studies have demonstrated the beneficial role of lutein plus zeaxanthin in AMD [13–15]. PUFAs play a role in inflammation and its resolution [16,17] and have a beneficial effect in AMD [18,19], and their role in the maintenance of vision has been endorsed by approval from the European Food Safety Authority [20]. Other substances, such as resveratrol and hydroxytyrosol, have been investigated and have shown antioxidant and/or antiangiogenic properties

in cultured retinal pigment epithelial cells [21,22]. However, there are insufficient data on the role of docosahexaenoic acid [DHA]/eicosapentaenoic acid [EPA] combined with the recommended dietary allowances of vitamins and minerals or the addition of other micronutrients, such as resveratrol, vitamin B or vitamin D, as preventive strategies in AMD [20].

We report herein the results of a randomized, observer-blinded trial aimed at evaluating the efficacy and safety of medicinal products containing the original AREDS formulation at doses approved in Europe with a product that adds to the formula DHA, lutein, zeaxanthin, resveratrol and hydroxytyrosol.

2. Materials and Methods

This was a randomized, observer-blinded trial conducted at nine sites in Spain and Portugal between November 2014 and April 2018. Every patient provided written informed consent before performing any study procedure. The protocol was approved by the ethics committee of the Clínica Universidad de Navarra, and each participant site endorsed that approval. The study was conducted following the principles included in the Declaration of Helsinki. Trial registration: ClinicalTrials.gov identifier NCT04756310.

2.1. Study Subjects

We included patients aged 50 years or older diagnosed with unilateral choroidal neovascularization secondary to AMD or any of its sequelae (i.e., disciform scar, pigment epithelium detachment secondary to subretinal fluid, and/or subretinal hemorrhage (stage V of the modified AREDS classification)) with no exudative involvement in the contralateral eye (study eye) and who provided written informed consent.

Patients were excluded if they met any of the following criteria: had myopia of six diopters; had posterior pole abnormalities that could lead to choroidal neovascularization such as choroidal nevus, angioid streaks, central serous choroidopathy, inherited degenerative retinal diseases, and diabetic retinopathy; had coexisting media opacities that prevent assessment of the fundus; were considered to be at risk of becoming lost to follow-up based on the investigator's judgment; had participated in a therapeutic trial within the last three months; had received any nutritional supplement within one month of the study entry; had suspected or confirmed diagnosis of substance use disorder (illegal drugs); and/or were not able to understand the study procedures.

2.2. Interventions

Patients were randomized in a 1:1 ratio with a block design to receive a supplement containing the components of the AREDS original formulation (i.e., vitamin C, vitamin E, beta-carotene and zinc) manganese and selenium (Theavit®, laboratorios Mayoli Spindler, Barcelona, Spain; the control group) or a supplement containing the AREDS original formulation, except for beta-carotene, plus copper, DHA, lutein, zeaxanthin, resveratrol and hydroxytyrosol (Retilut®, laboratorios Thea, Barcelona, Spain; the intervention group). The doses of the AREDS formulations complied with European requirements for these supplements and are specified in Table S1. The boxes containing the control and intervention products were identical in appearance and were consecutively numbered according to the randomization schedule. Patients were instructed to receive two capsules daily regardless of the assigned group.

2.3. Evaluations and Outcomes

Patients were evaluated at the inclusion visit (baseline) and at 6 and 12 months.

At baseline, we recorded information on medical and ophthalmologic history, a brief nutritional questionnaire was applied, the best-corrected visual acuity (BCVA) was assessed in the study eye in a sitting position using Early Treatment Diabetic Retinopathy Study (ETDRS) testing charts, an anterior segment biomicroscopy was performed, and stereoscopic fundus evaluation and digital fundus photography were performed; in addition, blood samples were obtained for biochemical analysis (see below). During the follow-up, BCVA, biomicroscopy and fundus evaluation were performed every six months, while digital retinography and biochemical analysis were performed at baseline and 12 months. Adverse events were recorded at each study visit.

2.4. Biochemical Analyses

Biochemical analyses included the determination of several inflammation and oxidative stress markers, vascular endothelial growth factor and the fatty acid profile.

2.4.1. Determination and Analysis of Lutein and Zeaxanthin

The extraction of lutein and zeaxanthin from the serum samples (600 µL) was carried out with a mixture of hexane:dichloromethane (5:1) [23], and the extract was reconstituted with a mixture of methanol:methyl-tert-butyl-ether (50:50) and injected (20 µL) into the chromatograph. A duplicate analysis was performed on 20% of the patients' samples, and all of them contained an internal standard (tocopherol acetate).

Lutein and zeaxanthin analysis was performed by high-performance liquid chromatography (HPLC) using a kit consisting of a model 600 pump, a Rheodyne injector, and a diode array detector (PDA) (Waters, Milford, MA, USA). The system included a C30 YMC column (5 µm, 250 × 4.6 mm i.d.) and a precolumn (Aquapore ODS type RP-18). The mobile phase consisted of methanol with triethylamine (0.1%) and methyl-tert-butyl-ether in a linear gradient from 95:5 to 70:30 in 30 min as described in Olmedilla-Alonso et al. [24]. The flow rate was 0.9 mL/min. The response was recorded using the Empower 2 software application (Waters). The identification of the compounds was carried out by comparing the retention times with those of standard compounds and comparing the UV-VIS spectra online. Quantification was performed using a calibration curve. The repeatability of the response to the concentration of these carotenoids was verified by repeated injections of the standards on the same day and on different days.

2.4.2. Multiplex Cytokine Analysis of IL-1b, -6, -8, -9, -10, -12p70, IFN-γ, MCP1 and TNF-α

Cytokine analysis for IL-6, -8, -18, IFN-γ, MCP1 and TNF-α was performed using FirePlex Firefly® Analysis Workbench (Abcam), which is software for multiplex protein expression assays from Abcam Laboratories. A total of 100 µL of each sample (plasma) was assayed. All cytokines are expressed in pg/mL.

2.4.3. MMP-10 Analysis by ELISA

Plasma samples were assayed for MMP-10 levels using the BioAim ELISA kit (BioAim Scientific, Scarborough, ON, Canada) following the manufacturer's instructions. Data are presented as pg/mL.

2.4.4. VEGF Measurement by Western Blot

Western blotting for VEGF determination was performed as previously described [25]. Briefly, 2 µL of plasma samples (diluted 1:10) was mixed with Laemmli buffer (Bio-Rad), boiled for 5 min, separated on 10 to 12% SDS PAGE gels and transferred to a nitrocellulose membrane. After blocking with 5% skimmed milk (*w*/*v*), 0.1% Tween-20 (*w*/*v*) in TBS (1 h, RT), membranes were exposed to rat monoclonal anti-VEGF antibody (1:5000, 512808, BioLegend, San Diego, CA, USA) at RT for 1 h followed by incubation at RT for 1 h with a horseradish peroxidase-conjugated goat anti-rat IgG-peroxidase conjugated antibody

(1:5000, 31470, Pierce Biotechnology, Waltham, MA, USA). Signals were detected with an enhanced chemiluminescence (ECL) kit (ECL Prime Western blotting detection kit, GE Healthcare) and captured with ImageQuant 400 (GE Healthcare, Fairfield, CT, USA). The relative intensities of the immunoreactive bands were analyzed with ImageQuantTL software (GE Healthcare). The loading was verified by Ponceau S red, and the same blot was stripped and reblotted with an anti-β-actin monoclonal antibody (Sigma-Aldrich) to normalize the VEGF levels.

2.4.5. Fatty Acid Profile Analysis

For the fatty acid (FA) profile analysis, we used a method described elsewhere [26]. The plasma was initially subjected to a saponification step with sodium methylate and anhydrous methanol to obtain FAs in their free form. Then, FA methyl esters (or FAMEs) were obtained with the use of boron trifluoride and methanol, and finally, the FAMEs were extracted with hexane and injected into the gas chromatograph. Quantification was performed by normalization, expressing the results in relative amounts. Omega-3 polyunsaturated fatty acid (n-3 PUFA), omega-3 long-chain polyunsaturated fatty acid (n-3 LCPUFA), omega-6 polyunsaturated fatty acid (n-6 PUFA) and omega-6 long-chain polyunsaturated fatty acid (n-6 LCPUFA) sums were created by adding the individual FAs. Additionally, the ratios of n-6:n-3 PUFAs and n-6:n-3 LCPUFAs were calculated for the analysis.

2.5. Statistical Analysis

The primary outcome was the mean change from baseline in the BCVA in the non-affected eye by choroidal neovascularization secondary to AMD (study eye). Secondary outcomes included the mean change from baseline in the evaluated cytokines and lipids and the frequency of adverse reactions, mainly the development of choroidal neovascularization in the study eye.

To detect a mean difference between treatments in the BCVA of 4.6 letters (standard deviation 8.9), assuming a high correlation between the baseline assessment and the determination to be compared (minimum correlation of 0.8 between both), with a two-sided significance level of 5%, a power of 90%, and an anticipated number of dropouts of 10 to 20%, a sample size of 40 patients per treatment arm was required.

All efficacy outcomes were analyzed in the intent-to-treat population using a visit-wise approach. To compare the mean changes from baseline in the different efficacy outcomes, we used the unpaired Student's *t*-test or the Mann–Whitney U test. All tests were two-sided and considered significant if $p < 0.05$. Effect sizes for the difference in mean changes between the intervention and control groups were calculated using Cohen's d [27]. We considered Cohen's d of <0.20, 0.20 to 0.49, 0.50 to 0.79, and ≥0.80 to reflect trivial, small, moderate, and large effect sizes, respectively [27]. Effect sizes that were at least moderate were interpreted as relevant changes. All analyses were performed with SPSS 20.0.

3. Results

3.1. Patient Disposition and Baseline Characteristics

We randomly assigned 109 patients to treatment. Fifty patients received the intervention, and 59 received the control. Patients had a mean age of 77.1 years (standard deviation [SD]; 7.6) and were evenly distributed with respect to sex. Baseline characteristics were generally well-balanced between the intervention and the control, except for the AMD status, which showed the worst result in patients from the intervention group (Table 1).

Of the 109 randomized patients, 93 completed the trial. The number of participants discontinuing treatment prematurely was 5 (10%) with the intervention treatment and 11 (18.6%) with the control treatment (Figure S1).

3.2. Best Corrected Visual Acuity

At month 12, ETDRS letters had decreased with the intervention (N = 45; mean change −1.73, 95% CI −3.28 to −0.19) and in the control group (N = 48; mean change −0.10, 95% CI −2.03 to 1.83), for an estimated treatment difference between the intervention and control groups of –1.63 (95% CI –0.83 to 4.09; p = 0.192).

3.3. Carotenoids and Polyunsaturated Fatty Acids

The serum levels of the carotenoids lutein and zeaxanthin increased with the intervention and remained almost unchanged with the control treatment, and the difference between the intervention and control was statistically significant and showed a large effect size at month 12 (Table 2).

Table 1. Baseline characteristics.

Characteristic	Intervention N = 50	Control N = 59
Age (years), mean (SD)	78.4 (7.0)	76.0 (8.0)
Sex (women), n (%)	22 (44.0)	31 (52.5)
BCVA, mean (SD)	75.6 (11.1)	76.2 (11.7)
Corneal alterations (yes), n (%)	2 (4.0)	2 (3.4)
AMD status		
Presence of drusen, n (%)	41 (82.0)	42 (71.2)
Degree of drusen, n (%)		
1	8 (16.0)	14 (23.7)
2	17 (34.0)	15 (25.4)
3	10 (20.0)	6 (10.2)
4	6 (12.0)	7 (11.9)
Missing	8 (18.0)	17 (28.8)
Pigmentary alterations, n (%)		
Hyperpigmentation	15 (30.0)	13 (22.0)
Hypo-/hyperpigmentation	15 (30.0)	15 (25.4)
Hypopigmentation	3 (6.0)	5 (8.5)
No alterations	17 (34.0)	15 (42.4)
Geographic atrophy (yes), n (%)	9 (18.0)	4 (6.8)
LENS status		
Phakic	30 (60.0)	37 (62.7)
Pseudophakic	20 (40.0)	22 (37.3)

AMD, age-related macular degeneration; BCVA, best-corrected visual acuity; SD, standard deviation.

At month 12, the mean changes in the PUFAs were statistically significantly different between the intervention and control groups except for the total n-6 LCPUFAs; thus, DHA, total n-3 PUFAs and total n-3 LCPUFAs showed a greater increase with the intervention than with the control treatment, with an effect size that was moderate to large, while the total n-6 PUFAs, total n-6 LCPUFAs and the ratios of n-6/n-3 PUFAs and LCPUFAs showed a greater decrease in the intervention group than in the control group, also with moderate to large effect sizes (Table 2).

Table 2. Changes in carotenoids and polyunsaturated fatty acids at month 12.

	Intervention			Control			Mean Difference (Intervention-Control)	*p*-Value	95% CI		Effect Size
**Variable**	***N***	**Mean Change**	**SD**	***N***	**Mean Change**	**SD**		**Student's *t*-Test**	**Lower**	**Upper**	**Cohen's d**
**CAROTENOIDS (μg/dL)**											
**Lutein**	42	24.41 **	27.93	43	−1.57	6.58	26.0	<0.001	17.08	34.91	1.29
**Zeaxanthin**	42	2.88 **	3.52	43	−0.09	1.29	2.98	<0.001	1.83	4.13	1.13
**POLYUNSATURATED FATTY ACIDS (as % of total fatty acids in plasma)**											
**DHA**	34	0.74	0.59	35	0.04	0.66	0.701	<0.001	0.4	1.004	1.12
**Σ n-3 PUFAs**	34	0.82	1.1	35	0.05	1.04	0.763	0.004	0.248	1.279	0.72
**Σ n-6 PUFAs**	34	−0.97	4.4	35	2.47	7.28	−3.444	0.021	−6.347	−0.541	0.57
**Σ n-3 LCPUFAs**	34	0.79	1.11	35	0.08	1	0.715	0.006	0.207	1.222	0.67
**Σ n-6 LCPUFAs**	34	−0.35	1.15	35	0.3	2.19	−0.654	0.126	−1.5	0.188	0.37
**Ratio of n-6/n-3 PUFAs**	34	−2.18	2.59	35	1.05	3.35	−3.227	<0.001	−4.7	−1.785	1.08
**Ratio of LCn-6:LCn-3 PUFAs**	34	−0.6	0.64	35	0.18	0.89	−0.783	<0.001	−1.155	−0.412	1.00

DHA, docosahexaenoic acid; LCPUFA, long-chain polyunsaturated fatty acid; N, number of evaluable patients; PUFA, polyunsaturated fatty acid; SD: standard deviation; CI, confidence interval. * $p < 0.05$. ** $p < 0.001$.

3.4. Inflammatory and Oxidative Stress Markers and Vascular Endothelial Growth Factor

The intervention was associated with significant reductions in the levels of interferon-γ, IL-1β and tumor necrosis factor (TNF)-α, while changes from baseline in the levels of cytokines in the control group were not significant. The intervention treatment reduced the levels of some cytokines, such as interleukin IL-8, IL-1β and TNF-α, to a significantly greater extent than the control group, with the effect size being small for IL-8 and moderate for IL-1β and TNF-α (Figure 1a–k; Table S3). Both treatment groups reduced matrix metalloproteinase (MMP)-10 and VEGF to a similar extent, and the difference between the groups was not statistically significant with a trivial effect size (Figure 1j,k; Table S3).

3.5. Tolerability and Safety

Overall, there were 21 adverse events reported throughout the study–13 in the intervention group and eight in the control group. Of them, 17 were related to the eyes. The most common adverse events related to the eyes were the development/progression of cataracts (five cases in the intervention group and one case in the control group) (Table S2). An adverse event of special interest was the development of exudative AMD in the study eye, which occurred in three patients in the intervention group and two patients in the control group.

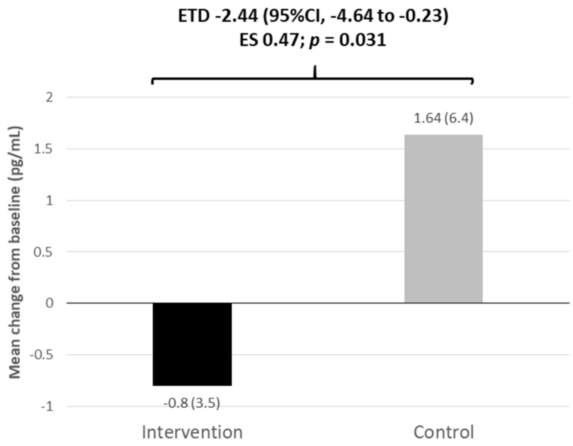

(**a**) Interleukin-8.

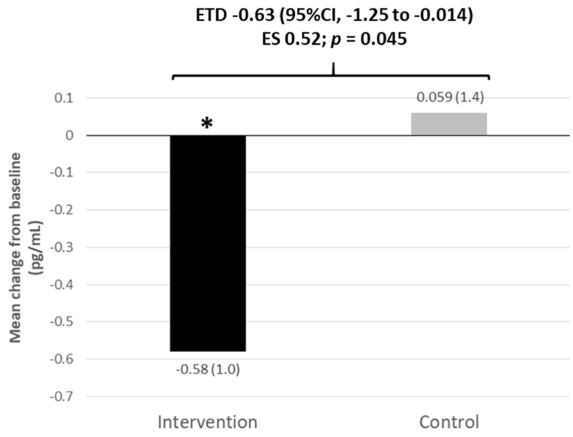

(**b**) Interleukin-1 β.

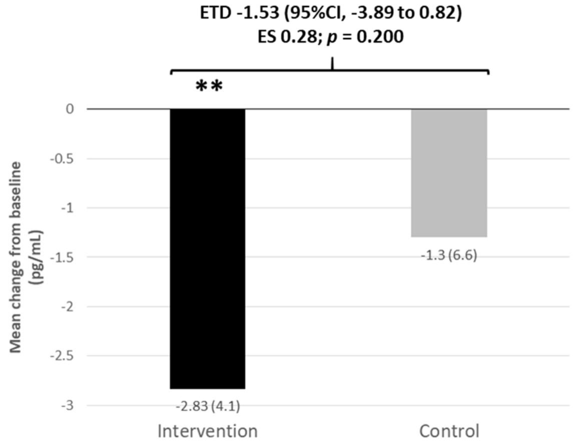

(**c**) Interferon-γ.

Figure 1. *Cont.*

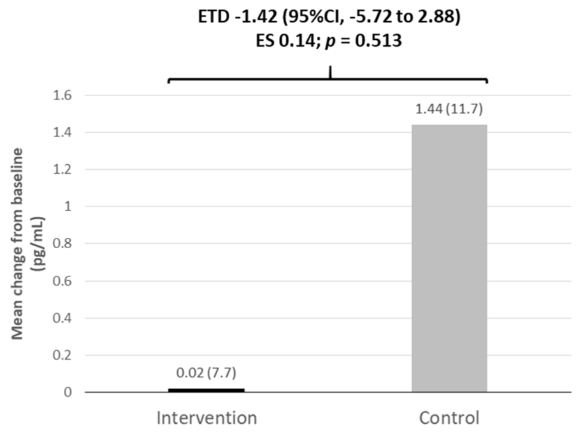

(**d**) 1g. Interleukin-9.

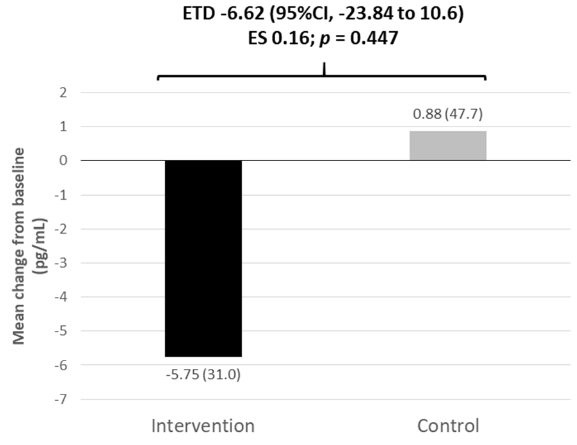

(**e**) Monocyte chemoattractant protein 1.

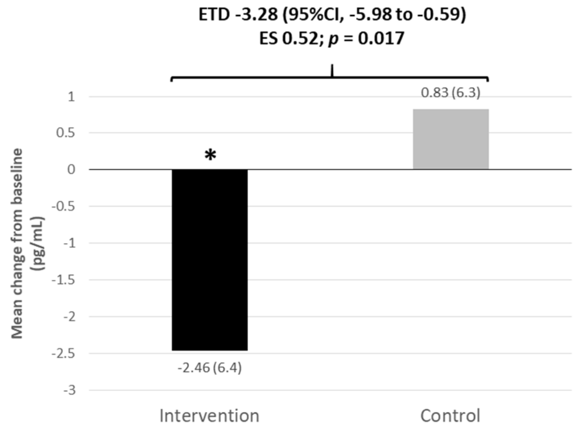

(**f**) Tumor necrosis factor-α.

Figure 1. *Cont.*

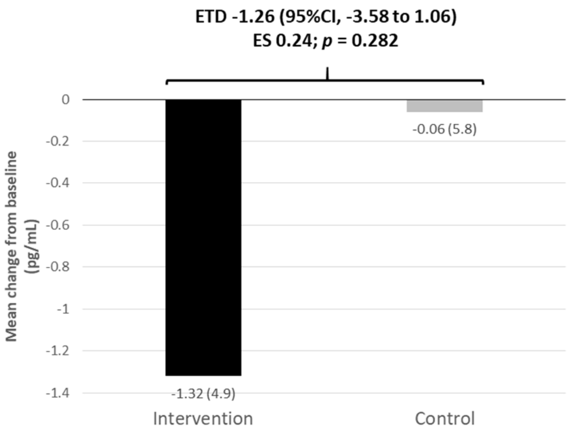

(**g**) Interleukin-10.

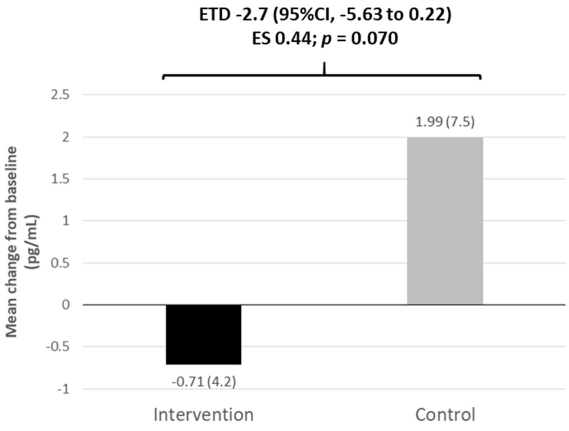

(**h**) Interleukin-6.

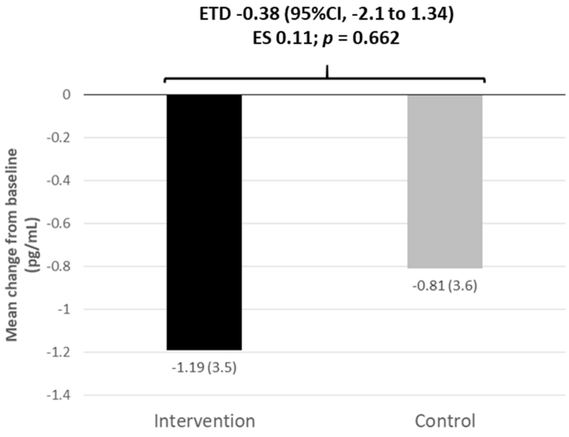

(**i**) Interleukin-12p70.

Figure 1. *Cont.*

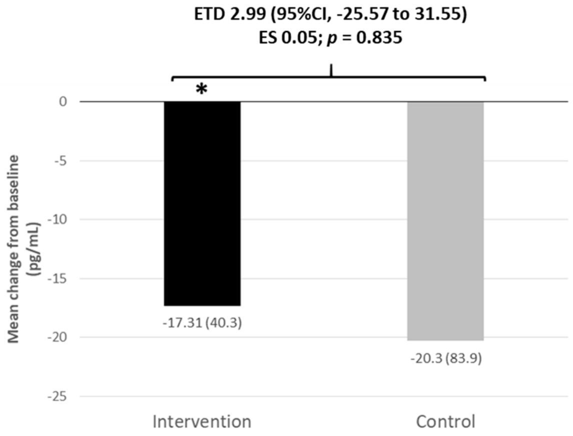

(**j**) Matrix metalloproteinase 10.

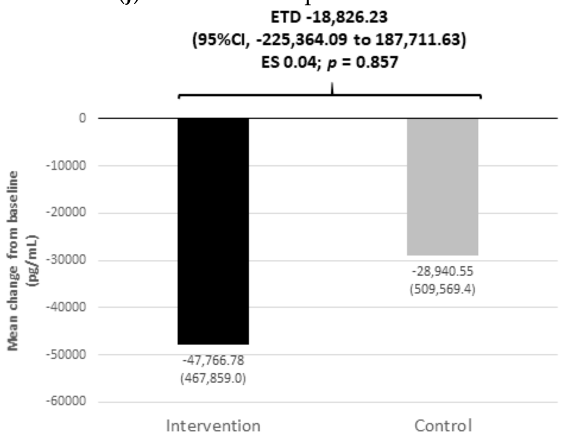

(**k**) Vascular endothelial growth factor.

Figure 1. (**a**–**k**). Inflammatory markers and angiogenic factors at month 12. CI, confidence interval; ETD, estimated treatment difference; ES, effect size (Cohen's d). Figures between brackets are standard deviations. * $p < 0.01$ vs. baseline; ** $p < 0.001$ vs. baseline.

4. Discussion

Overall, our results indicate that in patients with unilateral wet age-related macular degeneration, short-term treatment with the original AREDS formulation at doses approved in Europe supplemented with DHA, lutein, zeaxanthin, resveratrol and hydroxytyrosol has no significant differential effect on visual acuity compared with the original AREDS formulation at doses approved in Europe. The supplemented formula exhibits a significant and, in most cases, relevant effect in terms of reduction of some inflammatory cytokines and a greater improvement in the fatty acid profile and serum lutein concentration. Both formulations were generally well-tolerated.

Both supplements were associated with minimal changes in visual acuity, with no significant differences between them. Although difficult to compare, these results are consistent with those of the AREDS trial. In the AREDS trial, the median visual acuity score was maintained in the overall population, with a small reduction from a median of 86 to 85 after one or two years among patients with no or few drusen at baseline and no change in the same period among those with large drusen at baseline [28]. In the AREDS2, none of the AREDS formulations were associated with relevant worsening or improving of visual acuity [11].

Compared to the original AREDS formulation at doses approved in Europe, the supplemented formula was associated with a significant and relevant increase in DHA, total n-3 PUFAs and total n-3 LCPUFAs and a significant and relevant decrease in total n-6 PUFAs and the ratios of n-6/n-3 PUFAs and LCPUFAs. These results indicate that the

addition of DHA to the original formula has a positive and relevant impact on the fatty acid profile. However, there is great controversy about the beneficial effect of these fatty acids in AMD. Fish consumption appears to be associated with a significant reduction in the risk of developing AMD according to some meta-analyses [29,30]. However, a systematic review analyzing two placebo-controlled trials of omega 3 fatty acid supplements found that this supplementation in subjects with AMD for periods up to five years is not associated with a reduced risk of the progression of AMD or the development of relevant visual loss [31]. The results of the AREDS2 trial [11] support the results of that systematic review. However, an analysis of the Nutritional AMD Treatment 2 (NAT2) trial comparing the prophylactic effect of oral DHA with placebo showed that patients who maintained high levels of red blood cell membrane EPA/DHA had a reduced likelihood of choroidal neovascularization compared to those who maintained consistently low levels (hazard ratio 0.32, 95% CI 0.10 to 0.99) [18]. Bearing in mind the overall evidence, some authors consider that the beneficial effect of omega-3 fatty acids should be further evaluated using other formulations and/or populations of AMD patients [32].

Chronic inflammation is involved in the pathogenesis of AMD, as shown by the elevations in local and systemic proinflammatory markers in patients with AMD [33–35]. Chronic inflammation has been linked to a transformation of the tissue microenvironment into a senescence-associated secretory phenotype, releasing proinflammatory cytokines such as IL-1β, TNF-α, IL-6 and C-reactive protein [36,37]. In our study, the supplemented formula was also associated with a significantly greater reduction in inflammatory markers compared to the standard formula, more specifically with a significant reduction in IL-8, IL-1β and TNF-α and, albeit not significant, with a reduction in IL-6. All these changes were of moderate effect size with a Cohen's d of approximately 0.50. IL-1β is a potent proinflammatory cytokine whose upregulation induces angiogenesis and neuroinflammation [38] and has been reported to be an inflammatory mediator in the development of wet AMD, since these patients have shown increased plasma and vitreous levels of this cytokine [39,40]; in contrast, in animal models, its inhibition significantly reduced the development of subretinal neovascularization and has been shown to prevent choroidal neovascularization [39,41,42]. IL-6 has been associated with choroidal neovascularization [43,44], and IL-8 also plays a role in inflammation and angiogenesis [45]. High levels of IL-6 and IL-8 have been found in the aqueous humor of patients with AMD compared to patients with cataracts [46], and a recent meta-analysis confirmed that the levels of IL-8 are increased in patients with wet AMD [47]. A study that compared the cytokine profiles in aqueous humor in patients with neovascular AMD found positive correlations between interleukin IL-6 and IL-8 and monocyte chemoattractant protein (MCP) 1, a key chemokine that, in turn, has been associated with wet AMD [48,49]. TNF-α has also been involved in the pathogenesis of AMD [50]. In animal models, TNF-α contributes to laser-induced choroidal neovascularization formation [51], probably by upregulating VEGF production in retinal pigment epithelium cells [52], suggesting that TNF-α could be a therapeutic target for the prevention and treatment of AMD [51]. However, despite the initial interest in anti-TNF-α drugs for the treatment of retinal disease [53], in addition to safety concerns, anecdotal case reports provide controversial results on the use of anti-TNF-α drugs in patients with AMD [54,55]. Interestingly, resveratrol, a component of the supplemented formula, can decrease the secretion of proinflammatory cytokines such as IL-6, IL-8, and TNF-α [56] and, therefore, could have contributed to the observed effects on cytokines in our study in the intervention group.

The main limitation of our study is the short-term follow-up. A one-year follow-up is appropriate for detecting changes in biochemical parameters but not in visual acuity or in the occurrence of clinical events. Another possible limitation is the use of only systemic cytokines to evaluate the macular inflammatory and stress oxidation levels. The determination of these biomarkers in blood is an indirect determination and it has not been possible to demonstrate that they reflect what is happening at the macular level. However, as occurs with the main genetic factors related to factor H (fH)-related

complement activation, which takes place locally in the retina, our group has shown in previous studies that the concentration of fH variants in plasma varies between controls, AMD patients and aging patients, which can help explain the association of the fH-H402 protein with AMD [57]. These results indicate that the AMD pathology is not an exclusively ocular process.

Although there is no generalized agreement on which are the most important components of micronutrition, supplementation is currently included in the routine management of AMD by many ophthalmologists in Europe [58]. Our study shows that in patients with unilateral wet AMD, the addition of lutein, zeaxanthin, resveratrol, hydroxytyrosol and DHA to the AREDS EU recommended doses in the short term did not have a differential effect on visual acuity compared to a standard AREDS EU formula, but, in addition to improving the fatty acid profile and increasing carotenoid serum levels, it may provide a beneficial effect on improving the proinflammatory and proangiogenic profile of patients with AMD. The impact that these changes could have on the long-term progression of AMD to more advanced stages of the disease requires further investigation. It would be worth evaluating the long-term impact of this supplementation in other AMD subpopulations, such as those with intermediate AMD stages.

Supplementary Materials: The following are available online at https://www.mdpi.com/article/10.3390/nu13041253/s1, Table S1. Composition of Retilut® (intervention) and Theavit® (control); Table S2. Adverse events; Table S3. Changes in inflammatory markers and angiogenic factors at month 12; Figure S1. Patient disposition.

Author Contributions: Designed the clinical trial or Conceptualization and methodology: A.G.-L.; Laboratory assessments: S.R., M.H., P.F.-R., B.O.-A., M.C.L.-S.; Conducted the clinical trial: A.G.-L., J.N., E.H.-G., M.A.Z., P.C., N.P.-B., S.M.-M., M.J.A., R.S., J.J.E.-B., M.C.A.; Analyzed the data: A.G.-L., S.R., M.H., P.F.-R.; Review & Editing: A.G.-L., S.R., M.H., P.F.-R. All authors have read and agreed to the published version of the manuscript.

Funding: The research was funded by Laboratorios Théa (Barcelona, Spain) and by research grants from the "Instituto de Salud Carlos III/European Regional Development Fund (ERDF)" and RD16/0008/0011, OFTARED: Enfermedades oculares: "Prevención, detección precoz, tratamiento y rehabilitación de las patologías oculares".

Institutional Review Board Statement: The protocol was approved by the ethics committee of the Clínica Universidad de Navarra (Pamplona, Spain) and each participant site endorsed that approval. The ethical approval code is (Code-42/2014) of the Ethical Committee for Clinical Research of Navarra Government.

Informed Consent Statement: Every patient provided written informed consent before performing any study procedure.

Data Availability Statement: Data is available upon reasonable request from the corresponding authors.

Acknowledgments: The authors thank Fernando Rico-Villademoros (COCIENTE SL Madrid, Spain) for his editorial assistance. This assistance has been funded by Laboratorios Théa (Barcelona, Spain).

Conflicts of Interest: A.G.-L. has received consultant fees from Allergan, Bayer, Novartis, Roche, and Thea. S.R. declares no conflicts of interest. P.F.-R. declares no conflicts of interest. M.H. declares no conflicts of interest. M.J.A. has received consultant fees from Allergan, Bayer, Brill, Novartis, and Roche. J.N. has received consultant fees from Allergan, Novartis, and Zeiss. E.H.-G. declares no conflicts of interest. B.O.-A. declares no conflicts of interest. J.J.E.-B. has acted as the principal investigator in clinical trials from Roche, Novartis, and Kodiak. M.A.Z. declares no conflicts of interest. R.S. has received consultant fees from Allergan, Alimera, Bayer, Novartis, Roche, Thea, and NovoKordisk. M.C.A. declares no conflicts of interest. M.C.L.-S. declares no conflicts of interest. S.M.-M. declares no conflicts of interest. N.P.-B. declares no conflicts of interest. P.C. is a member of the advisory boards of Novartis and Bayer and a speaker for Novartis and Thea.

References

1. Jonas, J.B.; Cheung, C.M.G.; Panda-Jonas, S. Updates on the epidemiology of age-related macular degeneration. *Asia Pac. J. Ophthalmol.* **2017**, *6*, 493–497. [CrossRef]
2. Colijn, J.M.; Buitendijk, G.H.S.; Prokofyeva, E.; Alves, D.; Cachulo, M.L.; Khawaja, A.P.; Cougnard-Gregoire, A.; Merle, B.M.J.; Korb, C.; Erke, M.G.; et al. Prevalence of age-related macular degeneration in Europe: The past and the future. *Ophthalmology* **2017**, *124*, 1753–1763. [CrossRef]
3. Lane, J.; Rohan, E.M.F.; Sabeti, F.; Essex, R.W.; Maddess, T.; Dawel, A.; Robbins, R.A.; Barnes, N.; He, X.; McKone, E. Impacts of impaired face perception on social interactions and quality of life in age-related macular degeneration: A qualitative study and new community resources. *PLoS ONE* **2018**, *13*, e0209218. [CrossRef]
4. Elshout, M.; Webers, C.A.; van der Reis, M.I.; de Jong-Hesse, Y.; Schouten, J.S. Tracing the natural course of visual acuity and quality of life in neovascular age-related macular degeneration: A systematic review and quality of life study. *BMC Ophthalmol.* **2017**, *17*, 120. [CrossRef] [PubMed]
5. Chatziralli, I.; Mitropoulos, P.; Parikakis, E.; Niakas, D.; Labiris, G. Risk factors for poor quality of life among patients with age-related macular degeneration. *Semin. Ophthalmol.* **2017**, *32*, 772–780. [CrossRef] [PubMed]
6. Solomon, S.D.; Lindsley, K.; Vedula, S.S.; Krzystolik, M.G.; Hawkins, B.S. Anti-vascular endothelial growth factor for neovascular age-related macular degeneration. *Cochrane Database Syst. Rev.* **2019**, *3*, CD005139. [CrossRef]
7. Schmidt-Erfurth, U.; Chong, V.; Loewenstein, A.; Larsen, M.; Souied, E.; Schlingemann, R.; Eldem, B.; Mones, J.; Richard, G.; Bandello, F.; et al. Guidelines for the management of neovascular age-related macular degeneration by the European Society of Retina Specialists (EURETINA). *Br. J. Ophthalmol.* **2014**, *98*, 1144–1167. [CrossRef]
8. García-Layana, A.; García-Arumí, J.; Figueroa, M.S.; Arias Barquet, L.; Ruíz-Moreno, J.M.; Monclús-Arbona, L.; The Spanish AMD Multicenter Group. Management of wet age-related macular degeneration in Spain: Challenges for treat and extend implementation in routine clinical practice. *J. Ophthalmol.* **2019**, *2019*, 9821509. [CrossRef] [PubMed]
9. Gorusupudi, A.; Nelson, K.; Bernstein, P.S. The age-related eye disease 2 study: Micronutrients in the treatment of macular degeneration. *Adv. Nutr.* **2017**, *8*, 40–53. [CrossRef] [PubMed]
10. Age-Related Eye Disease Study Research Group. A randomized, placebo-controlled, clinical trial of high-dose supplementation with vitamins C and E, beta carotene, and zinc for age-related macular degeneration and vision loss: AREDS report no. 8. *Arch. Ophthalmol.* **2001**, *119*, 1417–1436. [CrossRef]
11. Age-Related Eye Disease Study 2 Research Group. Lutein + zeaxanthin and omega-3 fatty acids for age-related macular degeneration: The Age-Related Eye Disease Study 2 (AREDS2) randomized clinical trial. *JAMA* **2013**, *309*, 2005–2015. [CrossRef]
12. Age-Related Eye Disease Study 2 Research Group; Chew, E.Y.; Clemons, T.E.; Sangiovanni, J.P.; Danis, R.P.; Ferris, F.L., 3rd; Elman, M.J.; Antoszyk, A.N.; Ruby, A.J.; Orth, D.; et al. Secondary analyses of the effects of lutein/zeaxanthin on age-related macular degeneration progression: AREDS2 report no. 3. *JAMA Ophthalmol.* **2014**, *132*, 142–149. [CrossRef] [PubMed]
13. Huang, Y.M.; Dou, H.L.; Huang, F.F.; Xu, X.R.; Zou, Z.Y.; Lin, X.M. Effect of supplemental lutein and zeaxanthin on serum, macular pigmentation, and visual performance in patients with early age-related macular degeneration. *Biomed Res. Int.* **2015**, *2015*, 564738. [CrossRef]
14. Wu, J.; Cho, E.; Willett, W.C.; Sastry, S.M.; Schaumberg, D.A. Intakes of lutein, zeaxanthin, and other carotenoids and age-related macular degeneration during 2 decades of prospective follow-up. *JAMA Ophthalmol.* **2015**, *133*, 1415. [CrossRef] [PubMed]
15. Liu, R.; Wang, T.; Zhang, B.; Qin, L.; Wu, C.; Li, Q.; Ma, L. Lutein and zeaxanthin supplementation and association with visual function in age-related macular degeneration. *Invest. Ophthalmol. Vis. Sci.* **2014**, *56*, 252–258. [CrossRef] [PubMed]
16. Marion-Letellier, R.; Savoye, G.; Ghosh, S. Polyunsaturated fatty acids and inflammation. *IUBMB Life* **2015**, *67*, 659–667. [CrossRef] [PubMed]
17. Eynard, A.R.; Repossi, G. Role of ω3 polyunsaturated fatty acids in diabetic retinopathy: A morphological and metabolically cross talk among blood retina barriers damage, autoimmunity and chronic inflammation. *Lipids Health Dis.* **2019**, *18*, 114. [CrossRef] [PubMed]
18. Merle, B.M.; Benlian, P.; Puche, N.; Bassols, A.; Delcourt, C.; Souied, E.H.; Nutritional , A.M.D. Treatment Study Group. Circulating omega-3 Fatty acids and neovascular age-related macular degeneration. *Invest. Ophthalmol. Vis. Sci.* **2014**, *55*, 2010–2019. [CrossRef]
19. Liu, A.; Lin, Y.; Terry, R.; Nelson, K.; Bernstein, P.S. Role of long-chain and very-long-chain polyunsaturated fatty acids in macular degenerations and dystrophies. *Clin. Lipidol.* **2011**, *6*, 593–613. [CrossRef]
20. García-Layana, A.; Guirao Navarro, M. The European perspective of the nutritional supplements in AMD prevention. In Proceedings of the 12th ISOPT Clinical the International Symposium on Ocula Pharmacology and Therapeutics, Berlin, Germany, 9–12 July 2015; pp. 7–11.
21. Liu, Z.; Sun, L.; Zhu, L.; Jia, X.; Li, X.; Jia, H.; Wang, Y.; Weber, P.; Long, J.; Liu, J. Hydroxytyrosol protects retinal pigment epithelial cells from acrolein-induced oxidative stress and mitochondrial dysfunction. *J. Neurochem.* **2007**, *103*, 2690–2700. [CrossRef]
22. Nagineni, C.N.; Raju, R.; Nagineni, K.K.; Kommineni, V.K.; Cherukuri, A.; Kutty, R.K.; Hooks, J.J.; Detrick, B. Resveratrol suppresses expression of VEGF by human retinal pigment epithelial cells: Potential nutraceutical for age-related macular degeneration. *Aging Dis.* **2014**, *5*, 88–100. [CrossRef]

23. Olmedilla-Alonso, B.; Beltrán-de-Miguel, B.; Estévez-Santiago, R.; Cuadrado-Vives, C. Markers of lutein and zeaxanthin status in two age groups of men and women: Dietary intake, serum concentrations, lipid profile and macular pigment optical density. *Nutr. J.* **2014**, *13*, 52. [CrossRef]
24. Olmedilla-Alonso, B.; Estévez-Santiago, R.; Silván, J.M.; Sánchez-Prieto, M.; de Pascual-Teresa, S. Effect of Long-Term Xanthophyll and Anthocyanin Supplementation on Lutein and Zeaxanthin Serum Concentrations and Macular Pigment Optical Density in Postmenopausal Women. *Nutrients* **2018**, *10*, 959. [CrossRef]
25. Hernandez, M.; Recalde, S.; Garcia-Garcia, L.; Bezunartea, J.; Miskey, C.; Johnen, S.; Diarra, S.; Sebe, A.; Rodriguez-Madoz, J.R.; Pouillot, S.; et al. Preclinical evaluation of a cell-based gene therapy using the sleeping beauty transposon system in choroidal neovascularization. *Mol. Ther. Methods Clin. Dev.* **2019**, *15*, 403–417. [CrossRef]
26. Bondia-Pons, I.; Castellote, A.I.; López-Sabater, M.C. Comparison of conventional and fast gas chromatography in human plasma fatty acid determination. *J. Chromatogr. B Analyt. Technol. Biomed. Life Sci.* **2004**, *809*, 339–344. [CrossRef] [PubMed]
27. Kazis, L.E.; Anderson, J.J.; Meenan, R.F. Effect sizes for interpreting changes in health status. *Med. Care* **1989**, *27*, S178–S189. [CrossRef]
28. Chew, E.Y.; Clemons, T.E.; Agrón, E.; Sperduto, R.D.; Sangiovanni, J.P.; Davis, M.D.; Ferris, F.L. Ten-year follow-up of age-related macular degeneration in the age-related eye disease study. *JAMA Ophthalmol.* **2014**, *132*, 272. [CrossRef]
29. Zhu, W.; Wu, Y.; Meng, Y.F.; Xing, Q.; Tao, J.J.; Lu, J. Fish consumption and age-related macular degeneration incidence: A meta-analysis and systematic review of prospective cohort studies. *Nutrients* **2016**, *8*, 743. [CrossRef]
30. Dinu, M.; Pagliai, G.; Casini, A.; Sofi, F. Food groups and risk of age-related macular degeneration: A systematic review with meta-analysis. *Eur. J. Nutr.* **2019**, *58*, 2123–2143. [CrossRef]
31. Lawrenson, J.G.; Evans, J.R. Omega 3 fatty acids for preventing or slowing the progression of age-related macular degeneration. *Cochrane Database Syst. Rev.* **2015**, *2015*, CD010015. [CrossRef]
32. Souied, E.H.; Aslam, T.; Garcia-Layana, A.; Holz, F.G.; Leys, A.; Silva, R.; Delcourt, C. Omega-3 fatty acids and age-related macular degeneration. *Ophthalmic Res.* **2015**, *55*, 62–69. [CrossRef]
33. Čolak, E.; Ignjatović, S.; Radosavljević, A.; Žorić, L. The association of enzymatic and non-enzymatic antioxidant defense parameters with inflammatory markers in patients with exudative form of age-related macular degeneration. *J. Clin. Biochem. Nutr.* **2017**, *60*, 100–107. [CrossRef]
34. Haas, P.; Kubista, K.E.; Krugluger, W.; Huber, J.; Binder, S. Impact of visceral fat and pro-inflammatory factors on the pathogenesis of age-related macular degeneration. *Acta Ophthalmol.* **2015**, *93*, 533–538. [CrossRef]
35. Klein, R.; Myers, C.E.; Cruickshanks, K.J.; Gangnon, R.E.; Danforth, L.G.; Sivakumaran, T.A.; Iyengar, S.K.; Tsai, M.Y.; Klein, B.E.K. Markers of inflammation, oxidative stress, and endothelial dysfunction and the 20-year cumulative incidence of early age-related macular degeneration: The Beaver Dam Eye Study. *JAMA Ophthalmol.* **2014**, *132*, 446–455. [CrossRef]
36. Campisi, J. Cellular senescence: Putting the paradoxes in perspective. *Curr. Opin. Genet. Dev.* **2011**, *21*, 107–112. [CrossRef]
37. Freund, A.; Orjalo, A.V.; Desprez, P.Y.; Campisi, J. Inflammatory networks during cellular senescence: Causes and consequences. *Trends Mol. Med.* **2010**, *16*, 238–246. [CrossRef]
38. Liu, Y.; Biarnés Costa, M.; Gerhardinger, C. IL-1β is upregulated in the diabetic retina and retinal vessels: Cell-specific effect of high glucose and IL-1β autostimulation. *PLoS ONE* **2012**, *7*, e36949. [CrossRef]
39. Zhao, M.; Bai, Y.; Xie, W.; Shi, X.; Li, F.; Yang, F.; Sun, Y.; Huang, L.; Li, X. Interleukin-1β level is increased in vitreous of patients with neovascular age-related macular degeneration (nAMD) and polypoidal choroidal vasculopathy (PCV). *PLoS ONE* **2015**, *10*, e0125150. [CrossRef]
40. Subhi, Y.; Krogh Nielsen, M.; Molbech, C.R.; Oishi, A.; Singh, A.; Nissen, M.H.; Sørensen, T.L. Plasma markers of chronic low-grade inflammation in polypoidal choroidal vasculopathy and neovascular age-related macular degeneration. *Acta Ophthalmol.* **2019**, *97*, 99–106. [CrossRef]
41. Lavalette, S.; Raoul, W.; Houssier, M.; Camelo, S.; Levy, O.; Calippe, B.; Jonet, L.; Behar-Cohen, F.; Chemtob, S.; Guillonneau, X.; et al. Interleukin-1β inhibition prevents choroidal neovascularization and does not exacerbate photoreceptor degeneration. *Am. J. Pathol.* **2011**, *178*, 2416–2423. [CrossRef]
42. Zou, Y.; Xu, X.; Chiou, G.C. Effect of interleukin-1 blockers, CK112, and CK116 on rat experimental choroidal neovascularization in vivo and endothelial cell cultures in vitro. *J. Ocul. Pharmacol. Ther.* **2006**, *22*, 19–25. [CrossRef]
43. Barnes, T.C.; Anderson, M.E.; Moots, R.J. The many faces of interleukin-6: The role of IL-6 in inflammation, vasculopathy, and fibrosis in systemic sclerosis. *Int. J. Rheumatol.* **2011**, *2011*, 721608. [CrossRef]
44. Roh, M.I.; Kim, H.S.; Song, J.H.; Lim, J.B.; Koh, H.J.; Kwon, O.W. Concentration of cytokines in the aqueous humor of patients with naive, recurrent and regressed CNV associated with amd after bevacizumab treatment. *Retina* **2009**, *29*, 523–529. [CrossRef] [PubMed]
45. Ricci, F.; Staurenghi, G.; Lepre, T.; Missiroli, F.; Zampatti, S.; Cascella, R.; Borgiani, P.; Marsella, L.T.; Eandi, C.M.; Cusumano, A.; et al. Haplotypes in IL-8 gene are associated to age-related macular degeneration: A case-control study. *PLoS ONE* **2013**, *8*, e66978. [CrossRef]
46. Mimura, T.; Funatsu, H.; Noma, H.; Shimura, M.; Kamei, Y.; Yoshida, M.; Kondo, A.; Watanabe, E.; Mizota, A. Aqueous humor levels of cytokines in patients with age-related macular degeneration. *Ophthalmologica* **2019**, *241*, 81–89. [CrossRef] [PubMed]
47. Liu, J.; Tian, Z.; Li, J.; Zhao, G. Associations of IL-8 gene polymorphisms and IL-8 levels with predisposition to age-related macular degeneration: A meta-analysis. *Aging Clin. Exp. Res.* **2020**, *32*, 2703. [CrossRef] [PubMed]

48. Jonas, J.B.; Tao, Y.; Neumaier, M.; Findeisen, P. Monocyte chemoattractant protein 1, intercellular adhesion molecule 1, and vascular cell adhesion molecule 1 in exudative age-related macular degeneration. *Arch. Ophthalmol.* **2010**, *128*, 1281–1286. [CrossRef]
49. Suzuki, M.; Tsujikawa, M.; Itabe, H.; Du, Z.J.; Xie, P.; Matsumura, N.; Fu, X.; Zhang, R.; Sonoda, K.-h.; Egashira, K.; et al. Chronic photo-oxidative stress and subsequent MCP-1 activation as causative factors for age-related macular degeneration. *J. Cell Sci.* **2012**, *125*, 2407–2415. [CrossRef] [PubMed]
50. Touhami, S.; Beguier, F.; Augustin, S.; Charles-Messance, H.; Vignaud, L.; Nandrot, E.F.; Reichman, S.; Forster, V.; Mathis, T.; Sahel, J.-A.; et al. Chronic exposure to tumor necrosis factor alpha induces retinal pigment epithelium cell dedifferentiation. *J. Neuroinflammation* **2018**, *15*, 85. [CrossRef]
51. Lichtlen, P.; Lam, T.T.; Nork, T.M.; Streit, T.; Urech, D.M. Relative contribution of VEGF and TNF-alpha in the cynomolgus laser-induced CNV model: Comparing the efficacy of bevacizumab, adalimumab, and ESBA105. *Invest. Ophthalmol. Vis. Sci.* **2010**, *51*, 4738–4745. [CrossRef]
52. Wang, H.; Han, X.; Wittchen, E.S.; Hartnett, M.E. TNF-α mediates choroidal neovascularization by upregulating VEGF expression in RPE through ROS-dependent β-catenin activation. *Mol. Vis.* **2016**, *22*, 116–128.
53. Mirshahi, A.; Hoehn, R.; Lorenz, K.; Kramann, C.; Baatz, H. Anti-tumor necrosis factor alpha for retinal diseases: Current knowledge and future concepts. *J. Ophthalmic. Vis. Res.* **2012**, *7*, 39–44.
54. Fernández-Vega, B.; Fernández-Vega, Á.; Rangel, C.M.; Nicieza, J.; Villota-Deleu, E.; Vega, J.A.; Sanchez-Avila, R.M. Blockade of tumor necrosis factor-alpha: A role for adalimumab in neovascular age-related macular degeneration refractory to anti-angiogenesis therapy? *Case Rep. Ophthalmol.* **2016**, *7*, 154–162. [CrossRef]
55. Wu, L.; Arevalo, J.F.; Hernandez-Bogantes, E.; Regatieri, C.V.; Roca, J.A.; Farah, M.E. Intravitreal tumor necrosis factor-alpha inhibitors for neovascular age-related macular degeneration suboptimally responsive to antivascular endothelial growth factor agents: A pilot study from the pan american collaborative retina study group. *J. Ocul. Pharmacol. Ther.* **2013**, *29*, 366–371. [CrossRef]
56. Latruffe, N.; Lançon, A.; Frazzi, R.; Aires, V.; Delmas, D.; Michaille, J.J.; Djouadi, F.; Bastin, J.; Cherkaoui-Malki, M. Exploring new ways of regulation by resveratrol involving miRNAs, with emphasis on inflammation. *Ann. N. Y. Acad. Sci.* **2015**, *1348*, 97–106. [CrossRef]
57. Hakobyan, S.; Harris, C.L.; Tortajada, A.; Goicochea de Jorge, E.; García-Layana, A.; Fernández-Robredo, P.; Rodríguez de Córdoba, S.; Morgan, B.P. Measurement of factor H variants in plasma using variant-specific monoclonal antibodies: Application to assessing risk of age-related macular degeneration. *Invest. Ophthalmol. Vis. Sci.* **2008**, *49*, 1983–1990. [CrossRef]
58. Aslam, T.; Delcourt, C.; Holz, F.; García-Layana, A.; Leys, A.; Silva, R.M.; Souied, E. European survey on the opinion and use of micronutrition in age-related macular degeneration: 10 years on from the Age-Related Eye Disease Study. *Clin. Ophthalmol.* **2014**, *8*, 2045–2053. [CrossRef]
